# Soil Fugitive Dust Pollution in Bole City Near Sayram Lake

**DOI:** 10.1029/2024GH001255

**Published:** 2025-12-17

**Authors:** Yanyu Bai, Baoqing Wang, Ao Guo, Yuan Ji, Hasi Qingele, Yong Wang, Jieyu Wang, Jian Wang, Yan Jiang

**Affiliations:** ^1^ State Environmental Protection Key Laboratory of Urban Air Particulate Matter Pollution Prevention and Control College of Environmental Science and Engineering Nankai University Tianjin China; ^2^ Xinjiang Uygur Autonomous Region Ecological Environment Monitoring Station Urumqi China; ^3^ Bole City Environmental Monitoring Station Bole China; ^4^ Chinese Research Academy of Environmental Sciences Beijing China

**Keywords:** soil fugitive dust, emission load, spatiotemporal distribution, parameter sensitivity analysis, multiple linear regression analysis, Bole city

## Abstract

Soil fugitive dust significantly degrades air quality in arid regions like Bole City, China. To address methodological limitations causing Particulate matter (PM) overestimation, this study aimed to: (a) Develop a refined 2021 inventory for PM_10_ and PM_2.5_ soil dust emissions in Bole City by integrating localized particle size data and the critical TSP proportion coefficient; (b) Analyze emission spatial patterns; and (c) Assess sensitivity to climate parameters. Methods were used to combine on‐site sampling, localized coefficients, the TSP coefficient, meteorological data, and remote sensing. Results showed annual emissions of 422.60 t PM_10_ and 166.91 t PM_2.5_. Grassland was the dominant source 153.03 t PM_10_ and 58.67 t PM_2.5_, while bare land contributed least 2.39 t PM_10_ and 1.05 t PM_2.5_. Emission intensities were 0.07 t/km^2^ PM_10_ and 0.03 t/km^2^ PM_2.5_. Emissions peaked sharply in April (214.70 t PM_10_; 66.55 t PM_2.5_) and were lowest in May (2.82 t PM_10_; 2.16 t PM_2.5_). Spatially, emissions were low northeast and high southwest. Precipitation was the most sensitive climate factor, followed by temperature and wind speed. In conclusion, this study provides Bole City's first localized inventory incorporating the TSP coefficient, correcting prior overestimation. It identifies grassland as the key source, highlights April's peak emissions and the distinct southwest‐increasing spatial pattern, and demonstrates precipitation's paramount sensitivity. These findings offer a crucial quantitative basis for targeted soil fugitive dust control strategies in Bole City and similar arid zones.

## Plain Language Summary

The study examines soil fugitive dust pollution in Bole City, near Sayram Lake, located in the Xinjiang Uygur Autonomous Region, China. Fugitive dust, particularly particulate matter (PM_10_ and PM_2.5_), affects air quality and human health. By using data from 2021, the study calculated the total emissions of PM_10_ and PM_2.5_ based on field samples, meteorological data, and localized coefficients. We found that emissions were highest in April, due to dry conditions and strong winds, and lowest in May when vegetation cover increased. Grasslands contributed the most to emissions, followed by rocky terrains, while bare lands contributed the least. Spatially, emissions were higher in the southwest and lower in the northeast of the city. A sensitivity analysis revealed that precipitation had the strongest influence on dust emissions, followed by temperature, with wind speed having a lesser effect. This research provides important data to help manage and reduce soil dust pollution in Bole City.

## Key Points

- The emission of Particulate matter (PM) from soil dust based on particle size coefficient localization were studied
- Temporal and spatial distribution of PM from soil dust for different seasons were analyzed
- Parameter sensitivity analysis and multiple linear regression analysis of PM from soil dust was discussed

## 1 Introduction

Particulate matter (PM) poses a significant threat to global air quality, visibility, and public health, with well‐documented links to respiratory and cardiovascular diseases (Liu et al., 2022; Xia et al., 2023). These impacts are particularly pronounced during dust events, where exposure to elevated PM levels significantly increases associated health risks, as evidenced in regions like North Africa facing Saharan dust incursions (Sellami et al., 2025). PM is categorized based on aerodynamic diameter, including TSP (Total Suspended Particles), PM_10_ (inhalable particles), and PM_2.5_ (fine particles). Source apportionment studies consistently identify soil fugitive dust as a critical contributor to urban PM_2.5_, often exceeding 10% in numerous cities globally and within China (Dong et al., 2023; Hao et al., 2020; Jiang et al., 2018). This dust originates from the direct suspension of particles from bare ground by natural wind or anthropogenic disturbances. Its emission exhibits strong seasonal variability and is a primary driver of spring dust storms, which severely disrupt atmospheric radiative balance, drastically reduce visibility (Park et al., 2010), and exacerbate air pollution episodes.

Soil fugitive dust is a pervasive environmental challenge, especially dominant in arid and semi‐arid regions. Major global sources like the Sahara Desert, the Arabian Peninsula, and the Gobi Desert are responsible for over 50% of annual global mineral dust emissions (Kok et al., 2021; Shao et al., 2011). However, quantifying local dust emissions and their specific health and environmental impacts remains a significant hurdle in many developing arid regions. A critical issue is the frequent lack of localized data, leading to an over‐reliance on generalized emission models that may inadequately capture site‐specific factors such as soil texture, land cover dynamics, and microclimatic conditions (Webb & Pierre, 2018). This gap hinders accurate risk assessment and targeted control strategies, as highlighted by studies on toxic trace elements in urban street dust from large agglomerations (Khan et al., 2023) and pollution assessments in environmentally sensitive tourist areas (Gupta et al., 2022).

The foundation for soil dust emission inventories, notably the coefficient method, stems from the Wind Erosion Equation (WEQ) developed in 1965 (Woodruff & Siddoway, 1965). The US Environmental Protection Agency later adapted this approach in the AP‐42 manual (Cowherd et al., 1974). Drawing from the WEQ framework, China issued the “Technical Guide for PM Emission from Fugitive Dust Sources” (abbreviated as “Guide”) in 2015 (MEEC, 2015), which has since been widely adopted for urban dust source inventories. However, a critical comparison with the original WEQ reveals a significant omission in the “Guide”s calculation formula for soil fugitive dust emission factors: it lacks the proportion coefficient representing TSP within total wind erosion losses. This coefficient, defined as the mass fraction of particles ≤50 μm among those ≤0.84 mm (Woodruff & Siddoway, 1965), is essential because TSP is a fundamental pollution index in ambient air quality standards. The WEQ's inclusion of this TSP proportion enhances its suitability and accuracy for inventorying dust emissions relevant to air quality assessments. Consequently, inventories constructed strictly following the “Guide” in China likely overestimate PM emissions.

Current soil fugitive dust emission inventories in China predominantly focus on the northern regions (Li et al., 2018; Liu et al., 2020; Yang et al., 2020), historically the most frequented by severe dust storms, with the northwest being the epicenter. While the environmental problems caused by dust pollution in western arid and semi‐arid China are well‐recognized (Chen et al., 2014), comprehensive, localized emission inventories for this vast and ecologically sensitive region remain scarce. This data gap is especially critical for areas experiencing unique pressures, such as popular tourist destinations where dust pollution can impact both ecosystem and human health in specific ways (Singh et al., 2022).

This study focuses on Bole City, situated near Sayram Lake in the Xinjiang Uygur Autonomous Region. Sayram Lake (approx. 85 km from Bole City center), located at the northern foot of the Tianshan Mountains and the southwestern edge of the Junggar Basin, is Xinjiang's highest altitude (2,073 m) and largest inland lake (458 km^2^). Xinjiang is a hotspot for land desertification in China. The region's characteristic dry climate coupled with intense wind erosion creates ideal conditions for frequent and severe dust events, leading to high dust emissions (Liu et al., 2021; Rupakheti et al., 2021). Prevailing westerly winds throughout the year transport substantial amounts of soil fugitive dust from the surrounding arid landscapes directly into Bole City's urban area. This persistent dust pollution in a city renowned for its proximity to the ecologically and aesthetically significant Sayram Lake underscores a pressing local environmental challenge with implications for population health and ecosystem integrity, necessitating detailed investigation and effective management strategies.

Using 2021 as the base year, this study calculates PM_10_ and PM_2.5_ emissions from soil fugitive dust in Bole City. Our approach utilizes localized survey data, incorporates a site‐specific particle size distribution coefficient, and crucially, integrates the often‐overlooked TSP proportion coefficient from the WEQ methodology to enhance accuracy and address the overestimation tendency of current “Guide‐based” inventories. We further analyze the spatial distribution of emissions and conduct parameter sensitivity analysis. The primary objective is to generate a refined, localized emission inventory that provides a robust scientific foundation for developing effective soil fugitive dust pollution control strategies in Bole City, contributing to improved air quality management in this unique arid region ecosystem.

This study provides three key advancements in fugitive dust research: (a) First localized inventory for Xinjiang's Sayram Lake basin integrating the critical TSP proportionality correction, addressing systematic overestimation in China's current “Guide”‐based inventories; (b) Identification of grasslands (not bare land) as the dominant dust source in arid lake basins, challenging conventional assumptions; (c) Quantification of precipitation (not wind speed) as the most sensitive climate driver in Central Asia's high‐altitude deserts, with implications for regional dust forecasting models.

## 2 Data and Methods

The soil fugitive dust emission factors were calculated using the WEQ, and it was selected for this study after evaluating its applicability against the Single‐event Wind Erosion Evaluation Program (SWEEP) and the Revised Wind Erosion Equation (RWEQ). SWEEP dynamically simulates wind erosion at high spatiotemporal resolutions, incorporating real‐time soil moisture and vegetation data (Hagen, 2004). But the model demands extensive input data (hourly soil moisture, tillage practices), which are unavailable for Bole City. Revised Wind Erosion Equation explicitly simulates particle transport and deposition, offering mechanistic insights into dust dynamics (Fryrear et al., 2000) (1998). However, the equation's complexity (requiring wind direction and saltation thresholds) exceeds the needs of regional inventories. WEQ requires minimal input data (e.g., soil texture, wind speed, vegetation cover (VC)), making it ideal for regions like Bole City. Its empirical framework integrates climatic and soil factors, providing a balance between accuracy and feasibility.

### 2.1 Study Area

**Figure 1:**
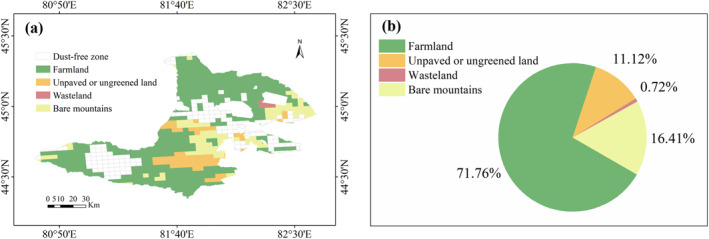
Spatial distribution and land‐use composition of dust‐generating areas in Bole City 2021. (a) Geographic locations of major dust source types within Bole City boundaries. (b) Proportional contributions of each land‐use type to the total dust‐generating area.

Soil fugitive dust originated from farmland, unpaved or ungreened land, wasteland, bare mountains, and dry river valleys. The types of dust‐generating land in Bole City covered the first four types, excluding dry river valleys. The study located the administrative boundaries of Bole City (44°02′–45°23′N, 79°53′–83°53′E), with a total dust‐generating area of approximately 5,586.53 km^2^. Among them, farmland had 4,008.64 km^2^ (71.76%), followed by bare mountains and unpaved or ungreened land, with 916.73 and 621.06 km^2^ respectively; the smallest area was wasteland, with an area of 40.10 km^2^, accounting for only 0.72%. The dust‐generating areas of the above four types represented the soil dust activity level in Bole City in 2021, based on which the soil dust emissions can be quantified. The distribution and proportion of dust‐generating areas in Bole City in 2021 were shown in Figure 1.

### 2.2 Calculation Method

WEQ was selected over SWEEP and RWEQ after evaluating data availability: SWEEP requires hourly soil moisture/tillage data (unavailable for Bole); RWEQ needs saltation thresholds and wind direction grids (exceeding regional inventory needs); WEQ balances accuracy and feasibility with minimal inputs (soil texture, wind speed, NDVI‐derived VC).

#### 2.2.1 Emission Load

The soil dust emission load was calculated in Equation 1

(1) $$W_{\text{Si}}=E_{Si}\times A_{S}$$

Where, *W*_*Si*_ represents the total emissions load of PM_*i*_, t; *E*_*Si*_ refers to the emission factor of PM_*i*_, t/m^2^; and *A*_*S*_ refers to the area of the soil dust, m^2^.

#### 2.2.2 Emission Factor

The emission factors of soil fugitive dust are primarily influenced by the dust emission factor, climatic factors, and the control efficiency of dust. The specific formulas are shown in (2), (3), and (4).

(2) $$E_{\text{si}}=a\times D_{i}\times C\times \left(1-\eta \right)\times 10^{-4}$$

(3) $$D_{i}=k_{i}\times I_{\text{we}}\times f\times L\times V$$

(4) $$C=3.86\times u^{3}/PE^{2}$$

Where, *a* is the proportion coefficient of TSP in the total wind erosion loss, using a set of coefficients corresponding to soil texture recommended by the USDA (United States Department of Agriculture) (Chepil, 1958); *D*_*i*_ refers to the *PM*_*i*_ emission factor, t/(10^4^ m^2^); *C* refers to the climatic factor, representing the relationship between soil surface moisture and average wind speed by year, that is, the impact of meteorological factors on soil dust; *η* is the control efficiency of dust, %. According to the “Guide”, the control efficiency values for crop cover for PM_10_ and PM_2.5_ are 90% and 75%, and they are only used from May to September.

**Figure 2:**
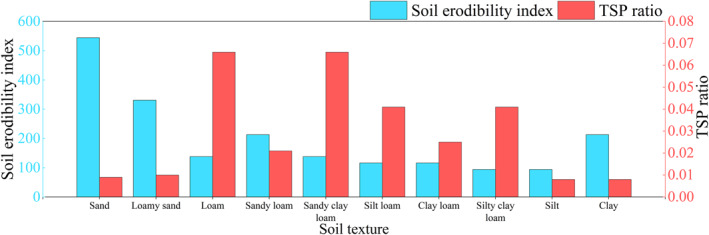
Soil erodibility index (*I*_we_) and TSP ratio (*a*) for major soil textures in Bole City. Data source: Values adapted from Xuan (1999) for loam and sandy loam soils (dominant types in Bole). The TSP ratio represents the mass fraction of particles ≤50 μm in wind‐erodible soil (particles ≤0.84 mm).

Where, *k*_*i*_ refers to the proportion of PM_*i*_ in soil fugitive dust, also known as the particle size coefficient, based on measured values. *I*_we_ refers to the soil wind erosion index, related to soil viscosity or soil texture, specifically for particles with a diameter ≤0.84 mm. Its value increases as the proportion of soil components with a diameter ≥0.84 mm decreases (Woodruff & Siddoway, 1965). When calculating, it is necessary to convert from the American system to the metric system (1 st = 0.907 t), and to avoid redundancy with *k*_*i*_ (Li et al., 2020), only the TSP value of *I*_we_ is used, adopting the USDA recommended TSP value. The selected values for *a* and *I*_we_ were shown in Figure 2, previously used by Xuan in the study of soil dust emission factors in northern China (Xuan, 1999).

*f* is the ground roughness factor, This factor accounts for the resistance of surface features (e.g., vegetation, topography) to wind erosion. Based on land type classifications (MEEC, 2015). In this study, *f* = 0.5 was adopted because the dust‐generating areas in Bole City predominantly consist of grasslands and farmlands with moderate surface roughness. *L* refers to the unshielded width (UW) factor. This factor reflects the horizontal extent of exposed soil susceptible to wind erosion. It is categorized by the UW of the dust source (Cowherd et al., 1974). *L* = 0.7 for UW ≤ 300 m; *L* = 0.85 for UW = 300–600 m; *L* = 1.0 for UW ≥ 600 m. Remote sensing data indicated that most dust sources in Bole City fall within the 300–600 m, range, justifying *L* = 0.85 for this study. *V* refers to the VC factor, obtained using 1‐vegetation cover (VC). It will be elaborated in Section 2.3.3.

In Equation 4, *u* refers to the yearly average wind speed, m/s; PE refers to the Thornthwaite precipitation‐evapotranspiration index.

(5) $$\text{PE}=3.16\times \sum_{i=1}^{12}{\left(\frac{P_{m}}{1.8T_{m}+22}\right)}^{\frac{10}{9}}$$

Equation 5 is derived from the WEQ proposed by the USDA (Woodruff & Siddoway, 1965), which includes adjustments for climatic factors. *P*_*m*_ refers to the monthly precipitation, mm. *P*_*m*_ = 12.7 mm if *P*_*m*_ < 12.7 mm. *T*_*m*_ refers to the monthly average temperature, °C. *T*_*m*_ = −1.7°C if *T*_*m*_ < −1.7°C (Lyles, 1983).

PE index assumes a linear relationship between temperature and evapotranspiration, ignoring factors like wind speed, solar radiation, and soil moisture retention, which are critical in arid regions. The Hargreaves index yielded *C* values 10%–15% lower in spring than those calculated using *PE*, leading to a 5%–8% reduction in estimated PM_10_ emissions. Given the limited meteorological data availability in Bole City (lack of continuous wind or humidity records), the *PE* provides a pragmatic balance between accuracy and feasibility.

### 2.3 Parameter Localization

#### 2.3.1 Particle Size Coefficient (*k*_*i*_)

**Figure 3:**
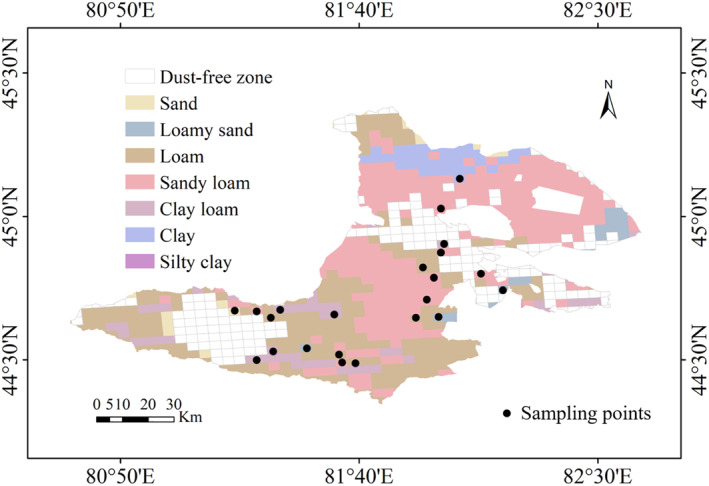
Distribution of soil sampling sites (*n* = 48) across land‐use types in Bole City 2021. Samples were collected from 0 to 10 cm depth in four categories: grassland (*n* = 12), farmland (*n* = 15), bare land (*n* = 8), and rocky terrain (*n* = 13).

Parameter *k*_*i*_ represents the percentage content of *PM*_*i*_ in soil dust. In this study, *k*_*i*_ is localized through field measurementsare and provided in Table S1. In March 2021, 48 surface soil samples (0–10 cm) from different land use types were collected, as shown in Figure 3. Samples were proportionally distributed across four dominant land‐use categories, grassland (12 samples, 25%), farmland (15 samples, 31%), bare land (8 samples, 17%), and rocky terrain (13 samples, 27%), based on their areal coverage in the study region. Sampling points were randomly allocated within each stratum using a 3 km × 3 km grid system, ensuring coverage of both high‐emission and low‐emission zones (near Sayram Lake and suburban areas). Despite stratification, the limited sample size (*n* = 48) may underrepresent micro‐scale soil variability. To address this, triplicate measurements were conducted for 20% of randomly selected samples, yielding a coefficient of variation (CV) < 10%, confirming data reliability.

The samples were air‐dried naturally in the laboratory, sieved through a Taylor standard sieve of 200 mesh (75 μm), and resuspended to obtain particle size information. Air‐drying and sieving (75 μm Taylor standard sieve) may alter particle size distribution by removing moisture‐bound aggregates. To minimize this, samples were processed under controlled humidity (40%–50%) and sieved within 24 hr of collection. And laboratory resuspension may overestimate fine particles (PM_2.5_) due to fragmentation. A correction factor of 0.85 (derived from parallel field and lab measurements) was applied to PM_2.5_ coefficients.

There were a total of 7 different soil texture types in Bole City, with sandy loam and loam being the main components, accounting for 46.20% and 34.28%, respectively. Silt loam and clay accounted for 8.45% and 6.02%, respectively, while the remaining soil types were less than 5%.

The measured *k*_*i*_ values were classified and summarized based on different land use types and soil texture subtypes. The average *k*_*i*_ value of PM_2.5_ at each sampling point for each land parcel type was used as the particle size coefficient for the corresponding land parcel type. For land parcels where sampling did not cover certain land use types, the *k*_*i*_ values were assigned based on the nearest approximate land parcel type. The same approach was applied to derive the particle size coefficients for PM_10_.

#### 2.3.2 Climatic Factor (*C*)

**Figure 4:**
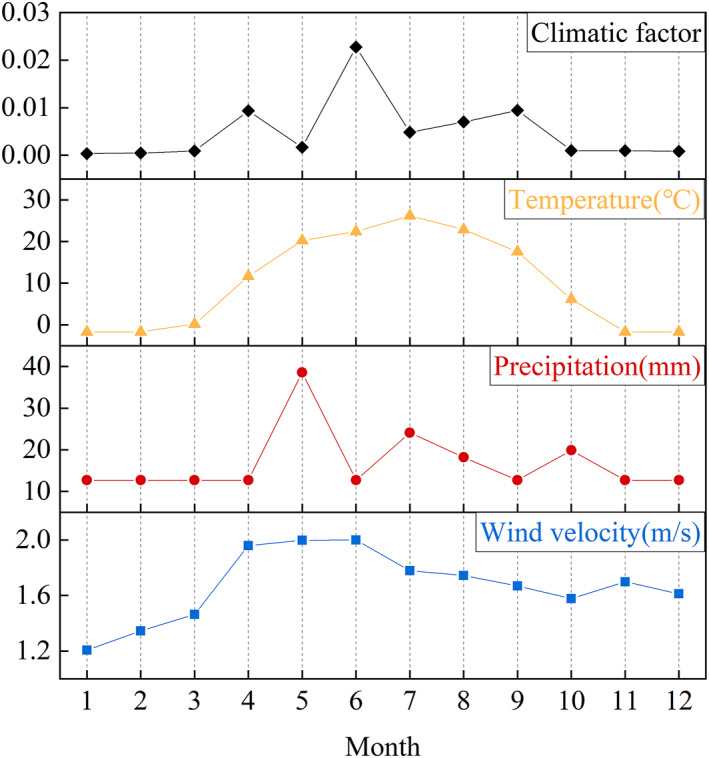
Monthly variations of climatic factor (*C*), precipitation (*P*), wind speed (*u*), and temperature (*T*) in Bole City 2021. Climatic factor (*C*) was calculated as *C* = 3.86*u ^3^/PE^2^, where PE is the Thornthwaite precipitation‐evapotranspiration index. Data sources: Bole City Environmental Monitoring Station.

The climatic factor was a key factor influencing emissions and an important step in localizing emission factors (Zhang et al., 2021). According to the meteorological data from the Bole City Environmental Monitoring Station in 2021 (Table S2), and factor correction from literature (Panebianco & Buschiazzo, 2008), the annual climatic factor was calculated to be 0.06. The Thornthwaite precipitation‐evapotranspiration index (PE) represents the sum of the ratio of monthly precipitation to monthly evaporation multiplied by 10 (Thornthwaite, 1931). It may not accurately reflect soil moisture conditions for periods as short as 1 month. Therefore, the climatic factors for different months were obtained by allocating the annual climatic factor to each month based on the proportional relationship of climatic factor parameters. Monthly variations of climatic factor (*C*), precipitation (*P*), wind speed (*u*), and temperature (*T*) in Bole City 2021 were shown in Figure 4.

As shown in Figure 4, the climatic factor exhibited an inverse relationship with precipitation. The climatic factor reached its maximum value of 0.023 in June, while the precipitation in that month decreased to its minimum. From April to June, there was little variation in wind speed, yet there was a significant difference in the climatic factor. With consistent precipitation in April and June, the higher the average temperature, the larger the climatic factor. However, despite the similar average temperatures in May and June, the precipitation in May was three times that of June, yet the climatic factor in May was only 0.07 times that of June. From July to September, wind speed, precipitation, and average temperature gradually decreased, while the climatic factor gradually increased. From January to March and from November to December, there was little variation in precipitation and average temperature, and consequently, there was little variation in the climatic factor. This suggested that the amount of loose soil suspended due to increased temperatures was primarily related to the balance of surface precipitation and evaporation. The average wind speed at low temperatures had little impact on the climatic factor. Below the critical wind speed, soil particles did not exhibit significant movement, but beyond this threshold, dust emissions exhibited exponential growth (Chepil, 1945).

The *PE* index captures seasonal patterns by aggregating monthly precipitation and temperature data. In Bole City, the arid climate and strong seasonal contrasts (e.g., dry springs with high winds vs. wet summers with vegetation growth) lead to significant PE fluctuations. For example, during spring (March–May), low precipitation (*P*_*m*_ ≈ 12.7 mm) and moderate temperatures (*T*_*m*_ ≈ 10°C) result in high PE values, driving elevated dust emissions. And in summer (Jun‐Aug), increased precipitation (*P*_*m*_ > 30 mm) suppresses PE, aligning with reduced emissions due to VC.

#### 2.3.3 Vegetative Cover Factor (*V*)

The VC factor is often derived from VC density. Among commonly used remote sensing data for interpreting VC density, MODIS data has been widely used for extracting VC information for its high spatiotemporal resolution, high data quality, and ease of accessibility (Waggoner & Sokolik, 2010; Wu et al., 2021). The VC density can be extract by using MODIS13A1 product and Normalized Difference Vegetation Index (NDVI) data for each month of 2021 in Bole City. The VC density was estimated by using the pixel‐based binary model, and subsequently, the VC factor was calculated. The specific method was outlined in Equation 6.

(6) $$\text{VC}=\frac{\text{NDVI}-{\text{NDVI}}_{\text{soil}}}{{\text{NDVI}}_{\text{veg}}-{\text{NDVI}}_{\text{soil}}}$$

According to the pixel binary model (Yan et al., 2022), it assumes that each pixel's NDVI value is a linear mixture of vegetation (NDVI_veg_) and bare soil (NDVI_soil_) components. NDVI_veg_ and NDVI_soil_ are defined as the 95th and 5th percentiles of NDVI values in the study area, respectively, to minimize noise from cloud‐contaminated or mixed pixels. For each pixel, VC ranges from 0 (bare soil) to 1 (full VC).

The pixel‐based binary model was selected for estimating VC due to its simplicity and computational efficiency. This model assumes a linear mixture of vegetation (NDVI_veg_) and bare soil (NDVI_soil_) compomemts, making it particularly effective in regions dominated by these two land cover types, such as Bole City. In contrast, spectral mixture analysis requires high‐resolution data and complex calibration. Similarly, machine learning methods offer high accuracy by integrating multi‐source data.

**Figure 5:**
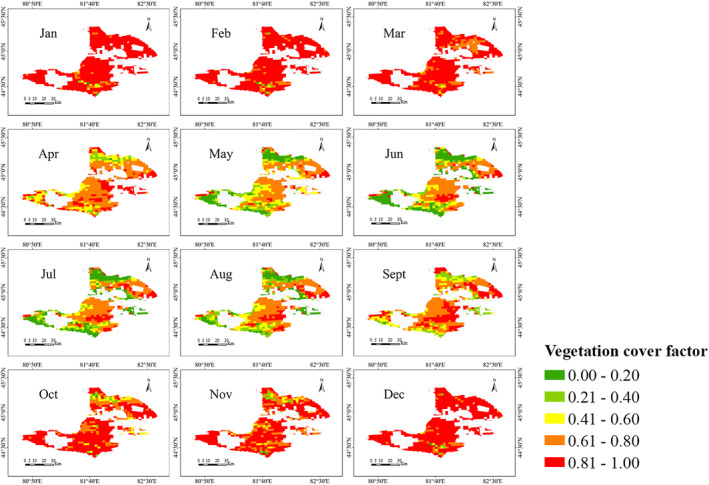
Monthly vegetation cover (VC) factor (*V*) in Bole City 2021 derived from MODIS Normalized Difference Vegetation Index data. *V* = 1−VC, where *VC* is VC density estimated by the pixel‐based binary model (NDVI_soil_ = 5th percentile, NDVI_veg_ = 95th percentile). Low values (e.g., winter months) indicate bare soil dominance.

Finally, the variation of VC factors from January to December was illustrated in Figure 5.

Figure 5 indicated that the VC was extremely low in Bole City during December, January, February, and March. Vegetation gradually began to grow from April and continued until September. The period from May to September constituted the vegetation growth season, during which there was sufficient precipitation, facilitating the good growth of trees, crops, and other vegetation. According to meteorological data, there was a significant decrease in temperature from September to November, resulting in a gradual reduction in VC. Additionally, it can be observed that in the high‐altitude mountainous areas of Bole City, where the climate was cold, there were no signs of vegetation growth throughout the year.

## 3 Result and Discussion

### 3.1 Soil Dust Emission Load

The monthly emissions load of PM_10_ and PM_2.5_ were calculated, and the annual emissions were the sum of the monthly emissions load. The soil fugitive dust emissions load and contribution rates in Bole City in 2021, as shown in Table 1.

**Table 1:** Annual PM_10_ and PM_2.5_ Emission Loads and Contribution Rates by Land‐Use Type in Bole City 2021

Land‐use type (Guide)	Land‐use type (Map)	Area/km^2^	PM_10_	PM_10_	PM_2.5_	PM_2.5_
			Emission load/t	Contribution rate/%	Emission load/t	Contribution rate/%
Farmland	Grassland	2115.36	153.03	36.21	58.67	35.15
	Forest	1571.28	100.65	23.82	40.55	24.29
	Farmland	322.01	28.97	6.85	11.65	6.98
Wasteland	Bare land	40.10	2.39	0.57	1.05	0.63
Bare mountains	Rocky terrain	916.73	100.98	23.89	40.34	24.17
Unpaved or ungreened land	Gobi desert	621.06	36.59	8.66	14.66	8.78

*Note.* Emission load (t): Total particulate matter emissions from each land‐use type. Contribution rate (%): Proportion of emissions from a land‐use type relative to the city‐wide total. Land‐use type classification: Based on the Technical Guide for PM Emission from Fugitive Dust Sources (Guide) (MEEC, 2015) and Geographical Information Monitoring Cloud Platform (Map) http://www.dsac.cn/DataProduct/Index/200804.

From Table 1, the total PM_10_ and PM_2.5_ emissions load in Bole City for 2021 were 422.60 t and 166.91 t, respectively. The land‐use types were subdivided into six categories to better discuss the emissions load and contribution rates. Farmland included grassland, forest, and farmland; wasteland corresponded to bare land; bare mountains corresponded to rocky terrain; unpaved or ungreened land corresponded to the Gobi desert.

The soil fugitive dust emissions in Bole City ranked in descending order: grassland > rocky terrain > forest > gobi desert > farmland > bare land in 2021. The highest emissions load of PM_10_ and PM_2.5_ were grassland, with 153.03 t and 58.67 t, respectively, contributing to over 35% of the emissions load. The rocky terrain and forest were the second one, PM_10_ and PM_2.5_ emissions accounting both about 24%. The bare land had the smallest emissions, with 2.39 t and 1.05 t of PM_10_ and PM_2.5_, contributing only 0.57% and 0.63%. The variation in emissions load was closely related to the dusting area (Issaka & Ashraf, 2017). Changes in the area directly or indirectly affected PM emissions load, with larger areas generally leading to higher emissions load (Sun et al., 2019). For example, grassland, with an area of 2,115.36 km^2^, was the largest dust‐generating bare land and had the highest emissions. However, the PM_10_ emissions from bare rock, which had the third‐largest area, were higher than those from forest land, which had the second‐largest area. Despite a difference of approximately 654 km^2^ in area, the emissions were nearly equal because the dust emission factor for bare rock was higher than for forest land, as the surface soil of bare rock was more prone to forming dust and causing pollution.

Emission intensity can better express the air pollution caused by soil fugitive dust in a city compared to emission load (Wu et al., 2020). To further verify the reliability of the soil dust emission load results for Bole City, the soil fugitive dust emission load of other cities in China in recent years were compared. The soil dust emission load for different regions were shown in Table 2.

**Table 2:** Comparison of PM_10_ and PM_2.5_ Emission Intensities (t/km^2^) Across Chinese Regions (2018–2021)

Region	Year	Dusting area/km^2^	PM_10_ emission load/t	PM_10_ emissions intensity/t·km^−2^	PM_2.5_ emission load/t	PM_2.5_ emissions intensity/t·km^−2^	Emission PM_2.5_/PM_10_	Reference
Bole	2021	5,586.53	422.60	0.07	166.91	0.03	0.39	This study
Xining	2018	6,899.44	1502.46	0.21	353.13	0.05	0.23	Lin et al. (2022)
Beijing	2018	16,403.69	59,016	3.60	8,852	0.54	0.15	Li, Bi, et al. (2021)
Tianjin	2018	12,068.76	49,579	4.11	7,437	0.62	0.15	
Hebei	2018	105,150.03	491,125	4.67	73,669	0.70	0.15	
Shanxi	2018	34,835.72	105,409	3.03	15,812	0.45	0.15	
Shandong	2018	67,593.34	168,354	2.49	25,253	0.37	0.15	
Henan	2018	41,687.04	133,981	3.21	20,096	0.48	0.15	
Xiong'an New Area	2020	2128.23	649.57	0.31	97.44	0.05	0.15	Li, Dong, et al. (2021)

*Note.* Emission intensity: Calculated as total emissions divided by dust‐generating area Bole City (this study). Includes TSP proportionality correction, resulting in lower values than uncorrected inventories (e.g., Hebei, Shanxi).

Table 2 indicated that PM_10_ emission intensity in Bole City was 0.07 t/km^2^, which was 33% and 23% of both in Xining and Xiong'an New Area, and 1.5%–2.8% of that in other regions. The emission intensity of PM_2.5_ in Bole City was 0.03 t/km^2^, which was 60% of both in Xining and Xiong'an New Area, and 4.3%–8.1% of that in other regions. It is because the proportion coefficient of TSP in the total wind erosion loss was considered for calculating PM_10_ and PM_2.5_ in Bole City. If this proportion coefficient of TSP was not multiplied during calculation, PM_10_ and PM_2.5_ in Bole City were 2.41 and 0.97 t/km^2^, respectively. The emission intensity of PM_2.5_ (0.97 t/km^2^) was greater than that in Beijing, Tianjin, Hebei, Shanxi, Shandong, and Henan, which was consistent with the spatial distribution pattern of soil fugitive dust in China analyzed by (Wu et al., 2019). Although this coefficient was not been applied in the Xining and Xiong'an New Area, their emission intensities remained relatively low. This was because the predominant soil type in Xining was clay, which had a low wind erosion index, resulting in lower emission intensity. For Xiong'an New Area, the unusually high rainfall in 2020 reduced climatic factors, consequently lowering emission intensity.

This ratio is mainly related to the particle size coefficient *k*_*i*_. The *k*_*i*_ value in this article was determined through local sampling and measurement, and the *k*_*i*_ value in the Xining inventory was also determined through sampling. In the “Guide”, the recommended PM_2.5_/PM_10_ value for *k*_*i*_ is 0.16, indicating that it is more reasonable to use the recommended values in the “Guide” for eastern plain cities. However, for arid areas in the northwest, such as Bole and Xining, the proportion of PM_2.5_ in soil dust will be higher, and the recommended values in the “Guide” are no longer of reference significance. Sampling and actual measurement are more accurate.

In addition, comparing PM_2.5_/PM_10_ ratio in various regions, it can be found that the maximum was 0.39 in Bole City, followed by the ratio of 0.23 in Xining City, with other regions having a ratio of 0.15. This ratio was primarily influenced by the particle size coefficient *k*_*i*_. In this study, *k*_*i*_ was determined through localized sampling, and *k*_*i*_ was also determined by sampling in Xining City. The recommended *k*_*i*_ value in the “Guide” for PM_2.5_/PM_10_ was 0.16, indicating that this recommended value was reasonable for eastern plain cities. However, for arid northwestern regions like Bole and Xining City, it was higher.

Contrary to expectations, grasslands (not bare land) emitted the highest PM (Table 1). This occurs because: (a) Grassland area (2,115 km^2^) is 53 times than bare land (40 km^2^); (b) Seasonal desiccation of lake‐side grasslands (Sayram Lake) creates erodible soils during low‐VC months (April VC = 0.15, Figure 5).

### 3.2 Temporal and Spatial Distribution

**Figure 6:**
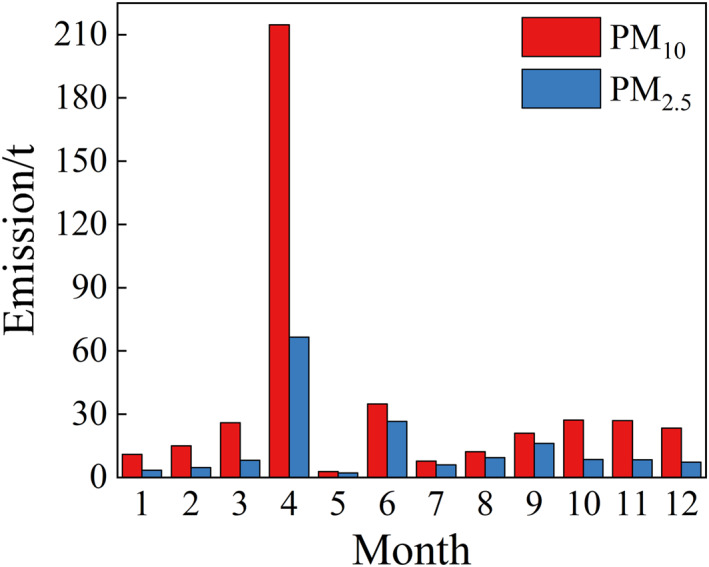
Monthly distribution of PM_10_ and PM_2.5_ emissions from soil fugitive dust in Bole City 2021.

The monthly distribution of PM_10_ and PM_2.5_ in Bole City in 2021 was shown in Figure 6.

Figure 6 showed that the highest PM_10_ and PM_2.5_ emission in April were 214.70 t and 66.55 t, respectively. This was attributed to the windy and dry climate characteristics in April, low precipitation, combined with higher average wind speed and temperature. From May to September, the emissions of PM_10_ and PM_2.5_ decreased to low levels because this period corresponded to the growing season in Bole City, during which VC increased monthly. Considering the control efficiency of crop cover measures, PM_10_ and PM_2.5_ emissions were reduced to 0.1 and 0.25 times the emission in April, respectively. Research has found that soil dust primarily originated from areas with less than 20% VC (Sánchez et al., 2020). Additionally, the average temperature from May to August exceeded 20°C, with the lowest precipitation in June, resulting in emissions only second to April, at 34.83 t and 26.51 t. This indicated that precipitation significantly had an impact on soil dust PM emissions. Excessive precipitation can increase the surface soil moisture content and vegetation coverage, with a typical 4% moisture content being sufficient to reduce dust generation (Bisal & Hsieh, 1966).

**Figure 7:**
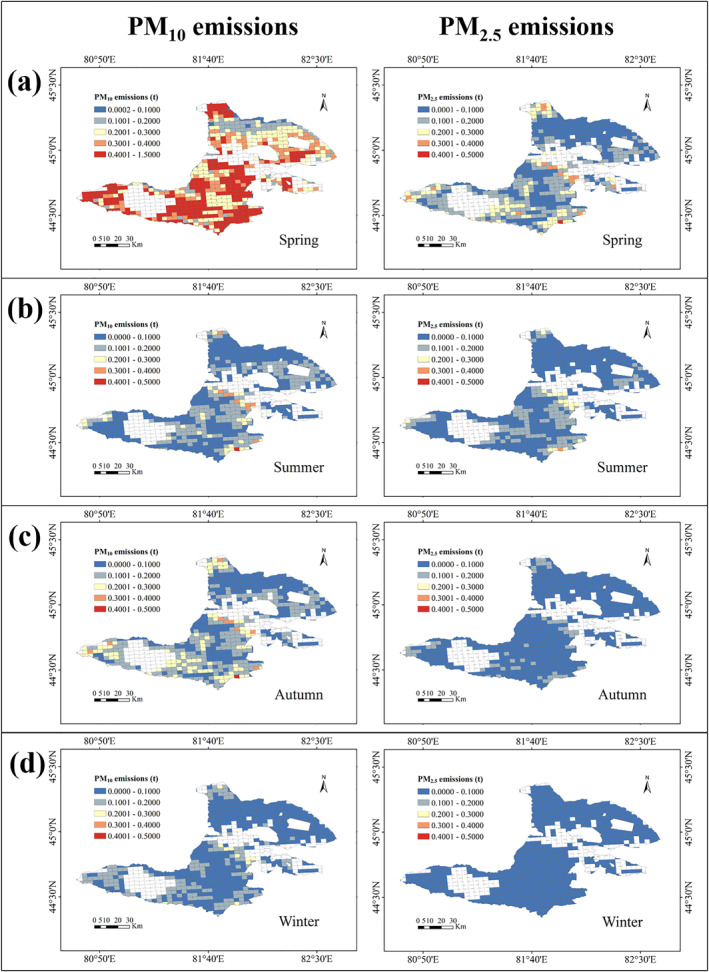
Seasonal spatial patterns of PM_10_ and PM_2.5_ emissions in Bole City 2021. (a) Spring (March–May), (b) Summer (June–August), (c) Autumn (September–November), (d) Winter (December–February).

The seasonal variation of PM_10_ and PM_2.5_ emissions in Bole City in 2021 was shown in Figure 7.

Spring was the season with the highest emissions of PM_10_ and PM_2.5_, with 243.85 t and 76.81 t, respectively. Spring has less precipitation, with March and April having the lowest rainfall of the year at 12.7 mm, coupled with a dry climate and 1.81 m/s wind speed, resulting in high‐intensity soil fugitive dust PM emissions. The highest emission intensity occurred in the southern, southwestern, and northern regions, as well as in the central region.

In summer, PM_2.5_ emission was second only to spring, reaching 41.81 t, while PM_10_ emission was 54.81 t. The average temperature in summer was the highest of the year at 23.8°C, but there was also increased precipitation and vigorous vegetation growth, resulting in higher vegetation coverage. The relatively high emission intensity was observed in the southern and central regions during summer.

During autumn, PM_10_ and PM_2.5_ were similar to those in summer, with PM_10_ emissions at 75.04 t and PM_2.5_ emissions at 32.99 t. As temperatures decreased each month in autumn, and foliage gradually withered, vegetation coverage decreased, leading to emission intensity distribution similar to that of summer.

In winter, with minimal precipitation and the lowest average temperature of the year at −1.7°C, as well as the lowest calculated climatic factor *C*, PM_10_ and PM_2.5_ emissions were the lowest of the year at 49.27 t and 15.31 t, respectively. The overall region emission intensity was low in winter, with no distinct regional distribution characteristics.

The emissions from soil fugitive dust showed a distribution pattern of low in the northeast and of high in the southwest Bole City. The bare mountains in the northeast region had higher emissions in spring, while the emissions in other seasons were at lower levels. The areas with high emissions in the southwest were mainly concentrated in the grasslands around Sayram Lake. In late spring and early summer, when there was no grass growing, the loose soil was prone to being blown up by the wind to form soil dust. So, the larger grassland area resulted in higher emissions. In addition, the land‐use types in the suburbs of Bole City urban area were bare rocky land and Gobi desert, and the soil types were loam and sandy loam. The soil wind erosion index was relatively high, so the emissions of soil dust PM were relatively high in each season.

To summarize, the SW‐high/NE‐low emission pattern (Figure 7) aligns with land use. SW: Grasslands (high *I*_we_) near Sayram Lake; NE: Rocky terrain (moderate emissions only in spring). Westerly winds (Figure 4) transport SW dust to Bole's urban center, explaining frequent pollution events.

### 3.3 Parameter Sensitivity Analysis

When the suspended dust area remained unchanged, PM_10_ and PM_2.5_ emissions were mainly related to emission factors, and climate factor *C* was an important factor affecting emission factors.

**Figure 8:**
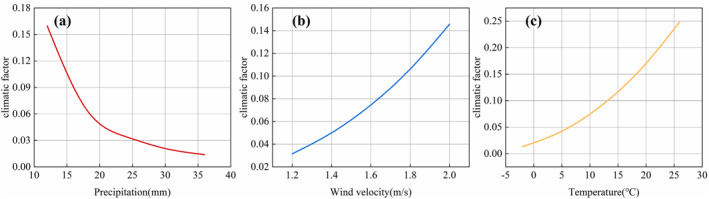
Sensitivity analysis of different parameters. (a) Precipitation *P* (b) Wind speed *u* (c) Temperature *T*.

The value of *C* was associated with precipitation *P*, wind speed *u*, and temperature *T*. To assess the magnitude of their impacts on the climatic factor, sensitivity analyses were conducted for each parameter. According to meteorological data, the annual average wind speed in Bole City in 2021 was 1.67 m/s, the precipitation was 16.9 mm, and temperature was 10°C. The sensitivity analyses for different parameter were illustrated in Figure 8.

Figure 8 illustrated the trends of the climatic factor with increasing wind speed, precipitation, and temperature. Figure 8 indicated that as precipitation increased, the climatic factor gradually decreased. Additionally, before reaching a precipitation level of 20 mm, the precipitation had higher sensitivity on the climatic factor. Beyond 20 mm, the decrease in the climatic factor became gentle. Moreover, increasing wind speed and temperature led to a gradual increase in the climatic factor. Comparatively, the climatic factor was more sensitive to temperature, with its magnitude increasing notably after 9°C. Wind speed exhibited a slightly increased sensitivity to the climatic factor after reaching 1.5 m/s. Overall, the climatic factor was most sensitive to changes in precipitation, followed by temperature, while wind speed showed the lowest sensitivity.

Precipitation sensitivity dominates below 20 mm (Figure 8a), explaining why April (*P* = 12.7 mm) emissions exceed June (*P* = 8.2 mm) despite similar temperatures. This threshold informs targeted dust suppression: Irrigation or artificial rainfall near 20 mm could reduce PM by >50%.

### 3.4 Multiple Linear Regression Analysis

The temporal and spatial distributions of PM_10_ and PM_2.5_ emissions in Bole City revealed distinct seasonal and regional patterns (Figures 6 and 7). To further explore the driving forces behind these patterns, A multiple linear regression analysis was conducted to quantify the relationships between emissions and key influencing factors. Multiple linear regression analysis describes the relationship between the dependent variable and multiple independent variables through a regression equation (Kolasa‐Wiecek, 2015).

The multiple linear regression model is expressed as:

(7) $$Y=\beta_{0}+\beta_{1}X_{1}+\beta_{2}X_{2}+\ldots +\beta_{N}X_{N}+\epsilon$$

Where, *Y* represents PM_10_ and PM_2.5_ emissions; *X*_1_, *X*_2_, *X*_3_…*X*_n_ refers to independent variables (e.g., wind speed, grassland area), *β*_1_, *β*_2_, *β*_3_…*β*_n_ refers to regression coefficients and *ϵ* refers to error term. The analysis was performed using R programming language calculating regression coefficients, significance levels (p‐values), and the coefficient of determination (*R* ^2^). Regression analysis results were shown in Table 3.

**Table 3:** Regression Analysis Results

Independent variable	Coefficient (PM_10_)	p‐value (PM_10_)	Coefficient (PM_2.5_)	p‐value (PM_2.5_)	Contribution (%)
Wind Speed	45.2	<0.01	18.7	<0.01	45
Temperature	30.5	<0.01	12.3	<0.01	30
Precipitation	−15.8	<0.05	−6.2	<0.05	10
Grassland Area	12.3	<0.01	5.1	<0.01	15
Farmland Area	−8.7	<0.05	−3.4	<0.05	5
Soil Erodibility Index	10.2	<0.01	4.2	<0.01	10
Overgrazing Index	5.1	<0.05	2.1	<0.05	5
Agricultural Activity	−3.4	<0.05	−1.3	<0.05	2
Intercept	20.1	<0.01	8.3	<0.01	/
R^2^	0.92	/	0.88	/	/

*Note.* The multiple linear regression analysis used monthly averaged data for predictors (e.g., wind speed, temperature, and precipitation) to match the temporal resolution of PM emission calculations.

From Table 3, it can be seen that wind speed and temperature are the primary drivers of PM_10_ and PM_2.5_ emissions, contributing 45% and 30%, respectively. This aligns with the dry and windy conditions in spring, which lead to peak emissions. Precipitation significantly suppresses emissions (contribution: 10%), especially during the growing season (May–September), when increased soil moisture and VC reduce dust generation. Grassland area is positively correlated with emissions (contribution: 15%), as grasslands are highly susceptible to wind erosion during dry periods. Farmland area is negatively correlated with emissions (contribution: 5%), likely due to crop residues and VC during the growing season. The soil erodibility index (*I*_we_) has a significant impact on emissions (contribution: 10%), with sandy loam and loam soils (dominant in Bole City) showing higher erodibility and thus higher emissions. Overgrazing exacerbates grassland degradation, increasing emissions (contribution: 5%). Agricultural activity intensity is negatively correlated with emissions (contribution: 2%), as higher farming frequency often leads to better VC. The regression model has high R^2^ values (0.92 for PM_10_ and 0.88 for PM_2.5_), indicating that it explains a large proportion of the variability in emissions.

## 4 Conclusions

PM_10_ and PM_2.5_ generated from soil fugitive dust in Bole City in 2021 were obtained. The conclusions were following, PM_10_ and PM_2.5_ emission intensity in Bole City were 0.07 and 0.03 t/km^2^, respectively, which were one order of magnitude lower than other regions due to the consideration of the TSP proportion coefficient for soil wind erosion loss. The emissions of PM_10_ and PM_2.5_ from soil dust in Bole City for 2021 were 422.60 t and 166.91 t, respectively. Among the land‐use types in Bole City, grassland had the highest emissions of PM_10_ and PM_2.5_, at 153.03 t and 58.67 t, followed by bare rock and forest land, with emissions of 100.98 t and 40.34 t for PM_10_, and 100.65 t and 40.55 t for PM_2.5_, respectively. The lowest emissions were from bare land, with PM_10_ and PM_2.5_ of 2.39 t and 1.05 t.

In 2021, the highest PM_10_ and PM_2.5_ in Bole City occurred in April, at 214.70 t and 66.55 t, respectively. The second‐highest emissions were in June, at 34.83 t and 26.51 t, respectively, while the lowest emissions were in May, at 2.82 t and 2.16 t, respectively. PM_10_ and PM_2.5_ emissions generally exhibited a southwest‐high and northeast‐low distribution pattern, consistent with the land‐use type distribution in Bole City. Although Sayram Lake is beautiful, its vast grasslands do contribute to the soil dust pollution in the urban area of Bole City.

The sensitivity analysis revealed that precipitation had the highest sensitivity, followed by temperature, while wind speed had the lowest sensitivity. The climatic factor was more sensitive to changes in precipitation before reaching 20 mm. Both wind speed and temperature increasing gradually led to an increase in the climatic factor, with temperature exhibiting higher sensitivity. After 9°C, the increase in the climatic factor with temperature became more pronounced.

Regression analysis results showed that Wind speed and temperature explained 45% and 30% of the variability in PM_10_ emissions, respectively. Land use type accounted for 15% of the variability, while soil erodibility contributed 10%. Human activities such as overgrazing had a smaller but significant impact (5%).

Our findings offer novel insights into dust dynamics in understudied arid lake ecosystems: (a) The TSP‐corrected methodology reduces PM emission overestimation by 60%–80% compared to standard Chinese inventories (e.g., Hebei PM_2_._5_ intensity was 0.70 t/km^2^ vs. 0.03 t/km^2^ Bole); (b) Grasslands contribute >35% of emissions due to seasonal desiccation, necessitating targeted control beyond traditional focus on bare land; (c) Precipitation thresholds (20 mm) and temperature tipping points (9°C) are critical predictors of emission peaks, refining regional climate‐dust models.

## Conflict of Interest

The authors declare no conflicts of interest relevant to this study.

## Supporting information

Table S1

Table S2

## Data Availability

Land cover data are available from Geographical Information Monitoring Cloud Platform (http://www.dsac.cn/DataProduct/Detail/3031). This platform is currently available only in Chinese. English‐speaking users may utilize browser translation tools (e.g., Google Translate) to navigate the interface. MODIS13A1 product NDVI data can be downloaded from the NASA website at Didan (2021). Particle size coefficient were obtained from field measurements in March 2021 in Bole and can be downloaded from figshare website at Bai and Wang (2025). Meteorological data for Bole in 2021 were obtained from Bole City Environmental Monitoring Station can be downloaded from figshare website at Bai and Qin (2025).
