# Supplementary material for: Soil Fugitive Dust Pollution in Bole City Near Sayram Lake
Source: Geohealth. 2025 Dec 17;9(12):e2024GH001255. doi: 10.1029/2024GH001255 (PMC12712225; doi:10.1029/2024GH001255)
Supplement: Supplementary file 1 — Table S1 [file GH2-9-e2024GH001255-s002.pdf]

GeoHealth

Supporting Information for

Soil fugitive dust pollution in Bole City near Sayram Lake

Yanyu Bai[1], Baoqing Wang[1], Ao Guo[1], Yuan Ji[2], Hasi Qingele[3], Yong Wang[3], Jieyu Wang[1], Jian Wang[4], Yan Jiang[4]

[1]State Environmental Protection Key Laboratory of Urban Air Particulate Matter Pollution Prevention and Control, College of Environmental Science and Engineering, Nankai University, Tianjin, China, [2]Xinjiang Uygur Autonomous Region Ecological Environment Monitoring Station, Urumqi, China, [3]Bole City Environmental Monitoring Station, Bole, China, [4]Chinese Research Academy of Environmental Sciences, Beijing, China

Contents of this file

Tables S1

Additional Supporting Information (Files uploaded separately)

Tables S2 Daily meteorological records for Bole City (2021)

Introduction

This document provides descriptions of the two primary data used to support the findings in the study “Soil fugitive dust pollution in Bole City near Sayram Lake. Table S1: Contains field-measured particle size coefficients (k_i) for PM_{10} and $PM_{2.5}$, derived from 48 surface soil samples collected across different land-use types in Bole City (March 2021). Table S2: Provides daily meteorological records (wind speed, temperature, and precipitation) for Bole City in 2021, sourced from the Bole City Environmental Monitoring Station.

Table S1. Particle size coefficients for PM10 and PM2.5 from surface soils in Bole City (March 2021)

Land use types	Soil types	$PM_{2.5}$/%	PM_{10}/%
Grassland	Sandy loam	8.01	28.61
	Silty clay	10	31.73
	Loamy sand	10.79	33.71
	Clay loam	9.55	30.79
Rocky terrain	Sandy loam	6.2	19.78
	Silty clay	5.97	21.94
	Loamy sand	7.19	23.83
	Loam	6.06	21.53
Farm land	Sandy loam	10.8	35.47
	Silty clay	13.17	39.31
	Loamy sand	12.87	38.6
	Clay	11.65	36.44
Bare land	Sandy loam	9.36	27.98
	Silty clay	9.14	19.64
	Loamy sand	9.14	29.42
	Sand	8.92	32.83
