# Supplementary material for: Soil Fugitive Dust Pollution in Bole City Near Sayram Lake
Source: Geohealth. 2025 Dec 17;9(12):e2024GH001255. doi: 10.1029/2024GH001255 (PMC12712225; doi:10.1029/2024GH001255)
Supplement: Supplementary file 2 — Table S2 [file GH2-9-e2024GH001255-s001.pdf]

Time	Temperature(℃)	Humidity (%)	Wind speed (m/s)	Wind scale	Wind direction (degree)	Wind direction	Air pressure(hpa)	Visibility (km)	The precipitation for this hour(mm)	Average total cloud cover(%)
2021-01-01 00:00:00	-20	76	2.4	2	265	west wind	972	13.1	0	93
2021-01-01 01:00:00	-21.5	75	1.1	1	333	northwest wind	972	12.8	0	93
2021-01-01 02:00:00	-22.1	81	0.5	1	273	west wind	971	11.4	0	93
2021-01-01 03:00:00	-22.5	80	0.7	1	313	northwest wind	971	9.1	0	93
2021-01-01 04:00:00	-22.7	80	0.5	1	288	west wind	972	10.8	0	93
2021-01-01 05:00:00	-22.2	79	0.9	1	306	northwest wind	971	10.2	0	93
2021-01-01 06:00:00	-23.2	79	1	1	253	west wind	971	12.7	0	93
2021-01-01 07:00:00	-23.1	80	0.7	1	324	northwest wind	971	11.9	0	82
2021-01-01 08:00:00	-24.4	77	0	0	0	north wind	971	11	0	93
2021-01-01 09:00:00	-23.3	81	1.2	1	358	north wind	971	9.2	0	93
2021-01-01 10:00:00	-23	82	0.8	1	342	north wind	971	5.3	0	93
2021-01-01 11:00:00	-21.3	83	0.7	1	306	northwest wind	971	2.9	0	82
2021-01-01 12:00:00	-19.7	81	1.2	1	309	northwest wind	971	3.3	0	93
2021-01-01 13:00:00	-18.1	78	1.3	1	280	west wind	971	5	0	93
2021-01-01 14:00:00	-16.8	73	1	1	251	west wind	970	6	0	93
2021-01-01 15:00:00	-15.4	70	1	1	302	northwest wind	970	6	0	82
2021-01-01 16:00:00	-14.8	65	1.1	1	121	southeast wind	970	7.7	0	23
2021-01-01 17:00:00	-14.9	64	1.2	1	21	north wind	970	7.6	0	23
2021-01-01 18:00:00	-17.4	75	0.9	1	118	southeast wind	970	5.4	0	23
2021-01-01 19:00:00	-19	79	0.6	1	202	south wind	971	4.3	0	23
2021-01-01 20:00:00	-19.3	83	0.8	1	319	northwest wind	971	4.6	0	82
2021-01-01 21:00:00	-21.3	81	0.5	1	238	southwest wind	971	4.7	0	82
2021-01-01 22:00:00	-21.2	81	0.8	1	270	west wind	971	5.5	0	93
2021-01-01 23:00:00	-21.5	80	1.9	2	254	west wind	971	6	0	82
2021-01-02 00:00:00	-20.8	80	0.9	1	283	west wind	971	7.3	0	82
2021-01-02 01:00:00	-19.8	80	1.9	2	251	west wind	971	7	0	93
2021-01-02 02:00:00	-19.7	80	1.4	1	252	west wind	971	7.8	0	82
2021-01-02 03:00:00	-20.1	80	1.2	1	265	west wind	971	7	0	93
2021-01-02 04:00:00	-21	81	1.6	2	265	west wind	971	10.4	0	23
2021-01-02 05:00:00	-21	80	1.5	1	262	west wind	971	19.4	0	82
2021-01-02 06:00:00	-22.2	78	1.1	1	258	west wind	971	20.1	0	82
2021-01-02 07:00:00	-21.9	78	1.3	1	249	west wind	972	19.7	0	93
2021-01-02 08:00:00	-21.3	79	2.2	2	262	west wind	973	16.5	0	93
2021-01-02 09:00:00	-21.7	77	1.4	1	266	west wind	973	11.5	0	93
2021-01-02 10:00:00	-21.4	79	2.2	2	260	west wind	974	12.3	0	82

2021-01-02 11:00:00	-20.2	77	2	2	256	west wind	974	12.8	0	82
2021-01-02 12:00:00	-17.1	73	1.9	2	259	west wind	974	15.7	0	82
2021-01-02 13:00:00	-14	66	1.4	1	252	west wind	974	14.7	0	23
2021-01-02 14:00:00	-10.6	61	0.7	1	288	west wind	974	18.1	0	1
2021-01-02 15:00:00	-10	58	2	2	6	north wind	973	17.9	0	1
2021-01-02 16:00:00	-11.1	61	1.8	2	44	northeast wind	974	16.3	0	1
2021-01-02 17:00:00	-11.6	62	1.7	2	46	northeast wind	974	13.6	0	1
2021-01-02 18:00:00	-12.9	69	1.5	1	36	northeast wind	975	10.2	0	23
2021-01-02 19:00:00	-14.6	75	1.4	1	41	northeast wind	976	8.8	0	1
2021-01-02 20:00:00	-17	79	1.1	1	79	east wind	976	6.8	0	23
2021-01-02 21:00:00	-18.3	84	1.2	1	319	northwest wind	977	3	0	82
2021-01-02 22:00:00	-19.9	81	0.9	1	269	west wind	977	4.3	0	82
2021-01-02 23:00:00	-19.6	82	0.5	1	290	west wind	977	3.1	0	82
2021-01-03 00:00:00	-19	83	0.4	1	255	west wind	977	3	0	23
2021-01-03 01:00:00	-18.9	83	0.3	1	333	northwest wind	977	3.2	0	23
2021-01-03 02:00:00	-19.1	83	0.4	1	302	northwest wind	977	3.4	0	23
2021-01-03 03:00:00	-19.3	83	0.7	1	274	west wind	976	3.8	0	23
2021-01-03 04:00:00	-20.4	82	1.3	1	265	west wind	976	7.7	0	93
2021-01-03 05:00:00	-22.8	78	1.8	2	253	west wind	976	9.2	0	93
2021-01-03 06:00:00	-23.5	78	2.1	2	253	west wind	976	4.2	0	93
2021-01-03 07:00:00	-23.2	78	1.5	1	265	west wind	976	4.8	0	93
2021-01-03 08:00:00	-23	78	1.7	2	264	west wind	977	3.7	0	93
2021-01-03 09:00:00	-22.4	78	1.8	2	270	west wind	977	4.4	0	82
2021-01-03 10:00:00	-21.8	79	1.1	1	199	south wind	977	2.7	0	82
2021-01-03 11:00:00	-21.1	79	1.5	1	262	west wind	978	5.1	0	23
2021-01-03 12:00:00	-19.1	77	0.9	1	233	southwest wind	978	6.1	0	23
2021-01-03 13:00:00	-16.8	74	0.8	1	350	north wind	978	5.2	0	82
2021-01-03 14:00:00	-15.5	68	1	1	1	north wind	977	5.7	0	82
2021-01-03 15:00:00	-15.4	70	1.5	1	29	northeast wind	977	4.3	0	82
2021-01-03 16:00:00	-15.6	70	1.3	1	27	northeast wind	978	4.4	0	82
2021-01-03 17:00:00	-15.9	69	1.3	1	65	northeast wind	979	3.7	0	82
2021-01-03 18:00:00	-17.1	74	0.9	1	234	southwest wind	980	3.6	0	82
2021-01-03 19:00:00	-18.7	78	0.6	1	319	northwest wind	980	3	0	82
2021-01-03 20:00:00	-21.2	80	0.6	1	275	west wind	981	3.3	0	93
2021-01-03 21:00:00	-22.7	79	1.6	2	256	west wind	981	4.5	0	93
2021-01-03 22:00:00	-23.4	76	1.8	2	263	west wind	981	5.6	0	93
2021-01-03 23:00:00	-24	77	1.3	1	272	west wind	981	6	0	93
2021-01-04 00:00:00	-24.5	76	1.6	2	261	west wind	982	6	0	93

2021-01-04 01:00:00	-24.7	77	1.7	2	264	west wind	982	6.6	0	93
2021-01-04 02:00:00	-25.2	77	2.2	2	258	west wind	982	6.6	0	93
2021-01-04 03:00:00	-25.3	78	2.1	2	261	west wind	982	6.6	0	93
2021-01-04 04:00:00	-25.3	76	1.9	2	251	west wind	982	3	0	93
2021-01-04 05:00:00	-24.4	77	2.2	2	258	west wind	983	4.5	0	93
2021-01-04 06:00:00	-24	77	1.5	1	263	west wind	983	2.9	0	93
2021-01-04 07:00:00	-23.9	77	1.2	1	266	west wind	984	4	0	93
2021-01-04 08:00:00	-23.6	78	0.9	1	250	west wind	984	4.7	0	82
2021-01-04 09:00:00	-23.1	78	1.4	1	258	west wind	985	5.3	0	82
2021-01-04 10:00:00	-23.1	78	0.7	1	301	northwest wind	986	3.8	0	82
2021-01-04 11:00:00	-22.9	78	1	1	257	west wind	986	5.2	0	82
2021-01-04 12:00:00	-21.7	75	1.1	1	248	west wind	986	5.8	0	93
2021-01-04 13:00:00	-19.8	69	1.1	1	224	southwest wind	986	5.7	0	93
2021-01-04 14:00:00	-17.6	64	1.2	1	148	southeast wind	985	5.6	0	93
2021-01-04 15:00:00	-18.1	67	1.6	2	107	east wind	985	5.7	0	93
2021-01-04 16:00:00	-18	68	1.2	1	85	east wind	985	4.7	0	93
2021-01-04 17:00:00	-18.7	70	0.9	1	78	east wind	985	5.3	0	82
2021-01-04 18:00:00	-19.8	73	1	1	25	northeast wind	986	5.1	0	82
2021-01-04 19:00:00	-21.4	77	0.6	1	260	west wind	986	4.2	0	93
2021-01-04 20:00:00	-23	80	0.5	1	272	west wind	986	4.4	0	93
2021-01-04 21:00:00	-25.4	78	1.1	1	251	west wind	986	4.1	0	93
2021-01-04 22:00:00	-26.6	77	1.3	1	263	west wind	985	5.3	0	93
2021-01-04 23:00:00	-26.8	77	1.4	1	267	west wind	985	5.8	0	93
2021-01-05 00:00:00	-27.9	75	1.1	1	279	west wind	984	7.3	0	93
2021-01-05 01:00:00	-27.4	76	1.9	2	268	west wind	984	7.6	0	93
2021-01-05 02:00:00	-27.8	75	1.6	2	263	west wind	984	7.4	0	93
2021-01-05 03:00:00	-28.1	74	1.5	1	261	west wind	983	8	0	93
2021-01-05 04:00:00	-28.2	75	1.9	2	271	west wind	983	7.6	0	93
2021-01-05 05:00:00	-28.4	75	1.4	1	263	west wind	983	8.2	0	93
2021-01-05 06:00:00	-28.9	74	1	1	288	west wind	982	7.9	0	93
2021-01-05 07:00:00	-28.9	75	1	1	275	west wind	982	8.6	0	93
2021-01-05 08:00:00	-29.8	73	0.6	1	271	west wind	982	6.5	0	93
2021-01-05 09:00:00	-30	75	0.6	1	277	west wind	981	5.5	0	93
2021-01-05 10:00:00	-28.9	77	1.4	1	268	west wind	981	4.8	0	93
2021-01-05 11:00:00	-28.2	76	1	1	265	west wind	981	6.3	0	93
2021-01-05 12:00:00	-25.1	75	0.3	1	286	west wind	981	5.6	0	93
2021-01-05 13:00:00	-22.6	74	0.6	1	235	southwest wind	980	6	0	93
2021-01-05 14:00:00	-21.6	71	1.3	1	343	north wind	980	7.4	0	93

2021-01-05 15:00:00	-21.2	70	1.1	1	322	northwest wind	979	7	0	93
2021-01-05 16:00:00	-20.9	69	1.5	1	39	northeast wind	978	6.9	0	93
2021-01-05 17:00:00	-21.2	70	1.6	2	31	northeast wind	978	8.2	0	82
2021-01-05 18:00:00	-22.3	72	1	1	6	north wind	979	8.4	0	93
2021-01-05 19:00:00	-24	77	1	1	175	south wind	979	4.8	0	93
2021-01-05 20:00:00	-24.9	80	0.3	1	358	north wind	979	6.2	0	93
2021-01-05 21:00:00	-26.6	78	0.5	1	327	northwest wind	978	5.2	0	93
2021-01-05 22:00:00	-27.2	77	0.5	1	267	west wind	978	4.6	0	93
2021-01-05 23:00:00	-28.2	76	1.3	1	260	west wind	978	4.5	0	93
2021-01-06 00:00:00	-29	74	0.5	1	281	west wind	978	4.7	0	93
2021-01-06 01:00:00	-28.3	76	1.8	2	259	west wind	979	5.3	0	93
2021-01-06 02:00:00	-28.1	76	1.3	1	254	west wind	978	6.1	0	93
2021-01-06 03:00:00	-28.4	75	0.7	1	282	west wind	978	6.5	0	93
2021-01-06 04:00:00	-29.5	73	0.6	1	276	west wind	979	8.1	0	93
2021-01-06 05:00:00	-29	76	0.9	1	269	west wind	979	6.6	0	93
2021-01-06 06:00:00	-28.2	76	1.4	1	260	west wind	979	6.2	0	93
2021-01-06 07:00:00	-28.6	76	1.2	1	266	west wind	979	5.7	0	93
2021-01-06 08:00:00	-29.3	74	0.7	1	268	west wind	979	7.4	0	93
2021-01-06 09:00:00	-29.4	74	1.1	1	270	west wind	979	6	0	93
2021-01-06 10:00:00	-29	75	1.3	1	267	west wind	979	6.4	0	93
2021-01-06 11:00:00	-27.6	75	0.9	1	249	west wind	979	5.3	0	93
2021-01-06 12:00:00	-24	75	0.8	1	290	west wind	979	4.9	0	93
2021-01-06 13:00:00	-20.1	69	1	1	148	southeast wind	979	5.4	0	93
2021-01-06 14:00:00	-18.9	64	1.7	2	163	south wind	978	6.2	0	93
2021-01-06 15:00:00	-18.4	62	1.5	1	71	east wind	978	7	0	93
2021-01-06 16:00:00	-18.9	66	1.3	1	49	northeast wind	978	7.4	0	82
2021-01-06 17:00:00	-18.5	62	1	1	340	north wind	978	7.1	0	93
2021-01-06 18:00:00	-19.4	66	0.7	1	78	east wind	978	5.7	0	93
2021-01-06 19:00:00	-20.9	72	1.1	1	119	southeast wind	978	4.5	0	93
2021-01-06 20:00:00	-21.9	79	0.9	1	352	north wind	978	4.1	0	93
2021-01-06 21:00:00	-22.9	78	0.2	0	0	north wind	978	3.9	0	93
2021-01-06 22:00:00	-23.5	79	1.3	1	350	north wind	978	4.5	0	93
2021-01-06 23:00:00	-23.9	80	0.2	0	0	north wind	977	3.8	0	93
2021-01-07 00:00:00	-24	80	1.7	2	267	west wind	977	4	0	93
2021-01-07 01:00:00	-24.2	80	1.8	2	255	west wind	977	4.2	0	93
2021-01-07 02:00:00	-24.1	79	1.8	2	261	west wind	977	4.8	0	93
2021-01-07 03:00:00	-23.5	79	1.7	2	254	west wind	977	4.9	0	93
2021-01-07 04:00:00	-23	79	1.3	1	279	west wind	977	4.9	0	93

2021-01-07 05:00:00	-23.3	77	1.3	1	42	northeast wind	977	4.9	0	93
2021-01-07 06:00:00	-24.5	78	0.8	1	326	northwest wind	977	5.6	0	
2021-01-07 07:00:00	-25.2	77	0.4	1	288	west wind	976	6.8	0	93
2021-01-07 08:00:00	-25.6	77	0.5	1	49	northeast wind	976	6.9	0	93
2021-01-07 09:00:00	-26	77	0.7	1	357	north wind	977	5	0	93
2021-01-07 10:00:00	-25.8	81	0.8	1	321	northwest wind	977	5.4	0	93
2021-01-07 11:00:00	-24.6	79	0.5	1	30	northeast wind	977	4.6	0	93
2021-01-07 12:00:00	-22.3	78	0.7	1	311	northwest wind	977	5.3	0	93
2021-01-07 13:00:00	-20.6	74	1.1	1	310	northwest wind	976	5.3	0	93
2021-01-07 14:00:00	-18.4	64	0.9	1	296	northwest wind	976	5.2	0	93
2021-01-07 15:00:00	-20.5	71	2.2	2	72	east wind	976	4.1	0	93
2021-01-07 16:00:00	-21.1	72	1.7	2	121	southeast wind	976	4.8	0	93
2021-01-07 17:00:00	-21.6	73	1.4	1	127	southeast wind	977	4.6	0	93
2021-01-07 18:00:00	-21.7	74	0.9	1	76	east wind	977	4.6	0	93
2021-01-07 19:00:00	-21.9	76	0.4	1	87	east wind	977	4.2	0	93
2021-01-07 20:00:00	-22.3	77	1.1	1	343	north wind	976	4.3	0	93
2021-01-07 21:00:00	-22.1	77	1.1	1	263	west wind	976	4.9	0	93
2021-01-07 22:00:00	-21.6	77	1.1	1	257	west wind	976	4	0	93
2021-01-07 23:00:00	-21.8	77	0.9	1	196	south wind	975	4.5	0	93
2021-01-08 00:00:00	-21.9	77	1.1	1	246	southwest wind	975	4.2	0	93
2021-01-08 01:00:00	-22.5	78	0.9	1	320	northwest wind	975	4.5	0	93
2021-01-08 02:00:00	-23	79	1.7	2	257	west wind	975	4.2	0	93
2021-01-08 03:00:00	-23.5	77	2	2	265	west wind	975	7.4	0	93
2021-01-08 04:00:00	-23.9	77	1.8	2	255	west wind	975	6.4	0	93
2021-01-08 05:00:00	-23.7	76	2.1	2	261	west wind	975	15.7	0	93
2021-01-08 06:00:00	-24.9	74	1.3	1	319	northwest wind	975	20.6	0	93
2021-01-08 07:00:00	-25.3	75	0.8	1	11	north wind	975	8.2	0	93
2021-01-08 08:00:00	-24.4	81	1.4	1	343	north wind	975	8.2	0	93
2021-01-08 09:00:00	-23.4	82	0.2	0	0	north wind	975	8.2	0	93
2021-01-08 10:00:00	-23.2	81	0.7	1	1	north wind	975	4.7	0	
2021-01-08 11:00:00	-21.8	80	0.4	1	312	northwest wind	976	4.9	0	90
2021-01-08 12:00:00	-20.5	74	0.7	1	302	northwest wind	976	6.7	0	90
2021-01-08 13:00:00	-18.9	69	0.7	1	345	north wind	975	7.2	0	90
2021-01-08 14:00:00	-17.7	66	0.8	1	164	south wind	975	6.5	0	90
2021-01-08 15:00:00	-16.1	62	0.4	1	261	west wind	975	6.7	0	90
2021-01-08 16:00:00	-15.4	59	0.8	1	3	north wind	975	5.9	0	90
2021-01-08 17:00:00	-16.3	68	1.6	2	120	southeast wind	975	4.6	0	90
2021-01-08 18:00:00	-16.6	69	1.3	1	123	southeast wind	976	4.1	0	90

2021-01-08 19:00:00	-17.9	76	1.1	1	40	northeast wind	976	4.2	0	90
2021-01-08 20:00:00	-18.7	79	0.8	1	54	northeast wind	977	4.2	0	90
2021-01-08 21:00:00	-18.9	80	1	1	291	west wind	977	3.7	0	90
2021-01-08 22:00:00	-19.5	81	0.7	1	57	northeast wind	976	3.5	0	90
2021-01-08 23:00:00	-20.1	82	0.8	1	348	north wind	976	3.3	0	90
2021-01-09 00:00:00	-21.5	81	0.3	1	20	north wind	976	3.4	0	90
2021-01-09 01:00:00	-22.2	83	0.8	1	318	northwest wind	976	3.3	0	90
2021-01-09 02:00:00	-22.9	82	1.1	1	331	northwest wind	976	3.5	0	90
2021-01-09 03:00:00	-23.5	81	0.9	1	250	west wind	976	4.7	0	90
2021-01-09 04:00:00	-24.4	79	0.4	1	294	northwest wind	977	8.9	0	90
2021-01-09 05:00:00	-23.6	79	0.8	1	288	west wind	977	10.3	0	90
2021-01-09 06:00:00	-22.9	79	0.9	1	100	east wind	977	4.7	0	90
2021-01-09 07:00:00	-22.4	83	1	1	37	northeast wind	977	3.2	0	90
2021-01-09 08:00:00	-22.3	83	0.8	1	334	northwest wind	978	3.1	0	90
2021-01-09 09:00:00	-22.1	82	0.3	1	184	south wind	978	3.3	0	90
2021-01-09 10:00:00	-22	82	0.6	1	45	northeast wind	979	3.1	0	90
2021-01-09 11:00:00	-21.3	82	1	1	335	northwest wind	979	3	0	90
2021-01-09 12:00:00	-19.8	79	0.8	1	225	southwest wind	979	3	0	82
2021-01-09 13:00:00	-18.9	74	0.9	1	146	southeast wind	980	3.6	0	82
2021-01-09 14:00:00	-15.5	60	1	1	159	south wind	979	3.6	0	82
2021-01-09 15:00:00	-15.3	61	0.9	1	136	southeast wind	978	3.5	0	82
2021-01-09 16:00:00	-15.3	62	1.2	1	90	east wind	978	3.4	0	82
2021-01-09 17:00:00	-15.1	63	1.6	2	123	southeast wind	979	3.3	0	82
2021-01-09 18:00:00	-16	64	0.2	0	0	north wind	979	3	0	23
2021-01-09 19:00:00	-18.7	75	0.7	1	310	northwest wind	980	2.7	0	23
2021-01-09 20:00:00	-20	78	0.8	1	329	northwest wind	981	2.2	0	23
2021-01-09 21:00:00	-21.4	81	0.6	1	322	northwest wind	981	2.2	0	82
2021-01-09 22:00:00	-22.7	81	0.4	1	288	west wind	981	2.8	0	82
2021-01-09 23:00:00	-23.5	79	0.6	1	347	north wind	981	2.5	0	82
2021-01-10 00:00:00	-24.3	80	0.6	1	11	north wind	980	3.3	0	82
2021-01-10 01:00:00	-25.3	78	0.7	1	264	west wind	981	2.4	0	82
2021-01-10 02:00:00	-24.3	83	1	1	349	north wind	981	1.9	0	82
2021-01-10 03:00:00	-26.3	80	0.5	1	196	south wind	980	2.6	0	82
2021-01-10 04:00:00	-25.8	79	0.4	1	290	west wind	980	3.1	0	82
2021-01-10 05:00:00	-26.4	77	0.9	1	290	west wind	980	3.4	0	82
2021-01-10 06:00:00	-26.7	78	1.1	1	255	west wind	980	2.7	0	82
2021-01-10 07:00:00	-26.8	76	0.9	1	300	northwest wind	980	2.7	0	82
2021-01-10 08:00:00	-26.5	77	2	2	257	west wind	980	2.5	0	82

2021-01-10 09:00:00	-26.4	77	1.4	1	257	west wind	981	2.8	0	90
2021-01-10 10:00:00	-26.1	78	1.5	1	259	west wind	981	2.7	0	90
2021-01-10 11:00:00	-25.5	78	1.9	2	254	west wind	981	2.9	0	90
2021-01-10 12:00:00	-22.7	79	1.2	1	249	west wind	981	3	0	90
2021-01-10 13:00:00	-20.3	76	1.9	2	253	west wind	980	3.3	0	90
2021-01-10 14:00:00	-18.2	70	1.4	1	217	southwest wind	979	4.1	0	90
2021-01-10 15:00:00	-16.1	64	1.1	1	189	south wind	978	4.3	0	82
2021-01-10 16:00:00	-17.1	64	1.9	2	75	east wind	978	3.7	0	82
2021-01-10 17:00:00	-18.4	70	1.5	1	124	southeast wind	978	3.3	0	82
2021-01-10 18:00:00	-18.6	72	0.4	1	237	southwest wind	978	3.1	0	82
2021-01-10 19:00:00	-19	76	0.9	1	162	south wind	978	3.2	0	82
2021-01-10 20:00:00	-19.8	78	0.4	1	74	east wind	978	2.8	0	82
2021-01-10 21:00:00	-21.1	79	1	1	344	north wind	977	2.1	0	90
2021-01-10 22:00:00	-22.1	82	0.6	1	293	northwest wind	977	2	0	90
2021-01-10 23:00:00	-22.8	81	1	1	290	west wind	976	2.4	0	90
2021-01-11 00:00:00	-23.4	80	1	1	238	southwest wind	975	2.4	0	90
2021-01-11 01:00:00	-23.8	79	0.2	0	0	north wind	975	3	0	90
2021-01-11 02:00:00	-23.9	80	0.5	1	38	northeast wind	974	3.1	0	90
2021-01-11 03:00:00	-24.2	79	0.9	1	282	west wind	974	5.7	0	90
2021-01-11 04:00:00	-24.6	79	0.7	1	272	west wind	973	5.5	0	90
2021-01-11 05:00:00	-25.5	77	0.7	1	287	west wind	973	7.7	0	90
2021-01-11 06:00:00	-25.2	77	0.6	1	279	west wind	973	7.6	0	90
2021-01-11 07:00:00	-24.8	78	1	1	271	west wind	973	6.1	0	90
2021-01-11 08:00:00	-25	78	1.2	1	251	west wind	972	4.3	0	90
2021-01-11 09:00:00	-24	79	1.2	1	257	west wind	972	4.6	0	90
2021-01-11 10:00:00	-23.8	80	1.6	2	264	west wind	972	5.9	0	90
2021-01-11 11:00:00	-21.6	78	1.7	2	248	west wind	972	6.5	0	90
2021-01-11 12:00:00	-18.9	75	1.5	1	255	west wind	971	7.3	0	90
2021-01-11 13:00:00	-15.5	69	1.1	1	301	northwest wind	970	8.2	0	90
2021-01-11 14:00:00	-13.7	59	0.8	1	8	north wind	969	8.9	0	90
2021-01-11 15:00:00	-13.2	59	1.8	2	107	east wind	968	6.3	0	82
2021-01-11 16:00:00	-15.2	67	2.4	2	79	east wind	967	3.9	0	82
2021-01-11 17:00:00	-15.5	68	1.3	1	85	east wind	967	4.6	0	82
2021-01-11 18:00:00	-16.6	72	1.4	1	112	east wind	966	4.1	0	82
2021-01-11 19:00:00	-18.3	77	0.7	1	331	northwest wind	965	3.1	0	82
2021-01-11 20:00:00	-19.6	80	0.6	1	21	north wind	965	2.8	0	82
2021-01-11 21:00:00	-20.4	83	0.9	1	287	west wind	965	2.2	0	82
2021-01-11 22:00:00	-21.3	80	1.3	1	248	west wind	964	3.3	0	82

2021-01-11 23:00:00	-21.5	81	1.7	2	255	west wind	964	3.5	0	82
2021-01-12 00:00:00	-22.2	78	0.6	1	274	west wind	963	3.8	0	82
2021-01-12 01:00:00	-23.2	78	0.5	1	285	west wind	962	4.2	0	82
2021-01-12 02:00:00	-23.7	78	0.7	1	284	west wind	962	5.5	0	82
2021-01-12 03:00:00	-23.7	80	0.8	1	257	west wind	961	6.8	0	82
2021-01-12 04:00:00	-23.5	79	0.7	1	270	west wind	961	6.5	0	82
2021-01-12 05:00:00	-24.4	76	0.4	1	281	west wind	960	7.1	0	82
2021-01-12 06:00:00	-24.1	79	0.9	1	259	west wind	959	8.2	0	82
2021-01-12 07:00:00	-23.7	79	1.4	1	260	west wind	958	9.3	0	82
2021-01-12 08:00:00	-23.1	79	1.7	2	270	west wind	958	6.4	0	82
2021-01-12 09:00:00	-24	78	0.6	1	268	west wind	959	5.2	0	82
2021-01-12 10:00:00	-23.5	79	0.7	1	277	west wind	959	6.4	0	82
2021-01-12 11:00:00	-20.6	79	0.9	1	273	west wind	959	6.4	0	82
2021-01-12 12:00:00	-16.5	72	0.4	1	158	south wind	959	6.7	0	82
2021-01-12 13:00:00	-14.3	65	1.2	1	178	south wind	959	8.3	0	
2021-01-12 14:00:00	-13.3	60	1.5	1	45	northeast wind	958	6.9	0	
2021-01-12 15:00:00	-13.9	65	1.5	1	91	east wind	958	4.9	0	
2021-01-12 16:00:00	-14	65	1.3	1	129	southeast wind	958	4.3	0	
2021-01-12 17:00:00	-14.8	70	1.1	1	105	east wind	957	5	0	
2021-01-12 18:00:00	-16	74	0.9	1	1	north wind	957	3.7	0	
2021-01-12 19:00:00	-17.5	78	1.4	1	77	east wind	957	3	0	
2021-01-12 20:00:00	-19.4	81	0.6	1	16	north wind	957	2.7	0	
2021-01-12 21:00:00	-20.1	81	1.1	1	245	southwest wind	957	2.3	0	
2021-01-12 22:00:00	-19.8	82	0.3	1	133	southeast wind	956	2.5	0	
2021-01-12 23:00:00	-19.3	82	0.7	1	348	north wind	955	2.9	0	
2021-01-13 00:00:00	-19.4	81	0.4	1	63	northeast wind	955	2.3	0	
2021-01-13 01:00:00	-19.8	84	1	1	7	north wind	955	2.2	0	
2021-01-13 02:00:00	-19.7	84	1.7	2	268	west wind	955	2.9	0	
2021-01-13 03:00:00	-19.7	81	1.1	1	214	southwest wind	954	3.3	0	
2021-01-13 04:00:00	-18.9	81	1.3	1	264	west wind	955	3.9	0	
2021-01-13 05:00:00	-18.8	79	1.2	1	334	northwest wind	955	4	0	
2021-01-13 06:00:00	-19.5	79	1.2	1	257	west wind	955	4.7	0	
2021-01-13 07:00:00	-17.8	81	0.6	1	187	south wind	955	4.7	0	
2021-01-13 08:00:00	-16.8	81	0.4	1	1	north wind	955	4.4	0	
2021-01-13 09:00:00	-17.1	80	0.7	1	77	east wind	955	3.8	0	
2021-01-13 10:00:00	-17.7	81	0.6	1	93	east wind	956	3.2	0	
2021-01-13 11:00:00	-15.7	81	1.8	2	274	west wind	957	5.9	0	
2021-01-13 12:00:00	-11.4	66	1.4	1	173	south wind	958	8.8	0	

2021-01-13 13:00:00	-6.1	60	0.9	1	129	southeast wind	958	9.3	0	
2021-01-13 14:00:00	-5.8	70	2.4	2	135	southeast wind	958	9.4	0	
2021-01-13 15:00:00	-4.9	66	1.4	1	153	southeast wind	958	10.7	0	
2021-01-13 16:00:00	-5.2	69	3.3	2	124	southeast wind	958	11.8	0	23
2021-01-13 17:00:00	-4.5	65	1.2	1	176	south wind	958	12.3	0	23
2021-01-13 18:00:00	-5.7	67	1.1	1	183	south wind	959	10.6	0	10
2021-01-13 19:00:00	-10	78	1.7	2	152	southeast wind	960	9.5	0	10
2021-01-13 20:00:00	-10.7	83	1.9	2	257	west wind	960	5.5	0	10
2021-01-13 21:00:00	-12.5	84	0.8	1	272	west wind	961	5.6	0	23
2021-01-13 22:00:00	-13.8	83	2	2	259	west wind	962	6.5	0	23
2021-01-13 23:00:00	-13.6	84	2.5	2	259	west wind	963	7.5	0	23
2021-01-14 00:00:00	-13.7	84	2.6	2	262	west wind	964	7.5	0	23
2021-01-14 01:00:00	-14	86	1.3	1	274	west wind	964	8.3	0	23
2021-01-14 02:00:00	-15.3	86	3.2	2	253	west wind	965	9.3	0	23
2021-01-14 03:00:00	-15.5	84	2.9	2	254	west wind	965	10.1	0	23
2021-01-14 04:00:00	-13.8	86	1.4	1	263	west wind	965	10.2	0	23
2021-01-14 05:00:00	-13.2	84	1.1	1	251	west wind	965	8.7	0	23
2021-01-14 06:00:00	-13.5	86	1.2	1	135	southeast wind	966	6.1	0	23
2021-01-14 07:00:00	-12.8	88	0.4	1	327	northwest wind	966	6	0	23
2021-01-14 08:00:00	-13.2	87	0.6	1	255	west wind	967	5.6	0	23
2021-01-14 09:00:00	-14.8	86	0.6	1	321	northwest wind	968	5.7	0	10
2021-01-14 10:00:00	-16	87	1	1	248	west wind	969	3.1	0	10
2021-01-14 11:00:00	-14.5	89	0.9	1	222	southwest wind	969	4.4	0	10
2021-01-14 12:00:00	-10.4	83	1.5	1	254	west wind	970	8.7	0	23
2021-01-14 13:00:00	-9	73	2.5	2	257	west wind	970	11.1	0	23
2021-01-14 14:00:00	-7.3	66	2	2	264	west wind	971	16.5	0	23
2021-01-14 15:00:00	-6.4	66	2.1	2	259	west wind	970	15.1	0	10
2021-01-14 16:00:00	-5.8	63	1.7	2	248	west wind	970	17.3	0	10
2021-01-14 17:00:00	-5.4	59	1.5	1	248	west wind	971	19.8	0	10
2021-01-14 18:00:00	-9	77	2.5	2	90	east wind	971	9.4	0	10
2021-01-14 19:00:00	-11	84	1.5	1	79	east wind	972	9.9	0	10
2021-01-14 20:00:00	-12.7	86	1.2	1	348	north wind	973	7.5	0	10
2021-01-14 21:00:00	-15.6	85	0.5	1	339	north wind	974	4.4	0	23
2021-01-14 22:00:00	-16.9	85	0.9	1	264	west wind	974	5.3	0	23
2021-01-14 23:00:00	-18.4	85	0.8	1	248	west wind	975	7	0	23
2021-01-15 00:00:00	-18.2	85	0.6	1	261	west wind	975	4.4	0	23
2021-01-15 01:00:00	-16.4	88	0.5	1	57	northeast wind	975	2.6	0	23
2021-01-15 02:00:00	-15.2	88	0.7	1	7	north wind	975	5	0	23

2021-01-15 03:00:00	-14.4	86	1.5	1	71	east wind	975	6.5	0	23
2021-01-15 04:00:00	-14.6	86	1.5	1	41	northeast wind	975	6.1	0	23
2021-01-15 05:00:00	-14.8	86	1.8	2	85	east wind	975	5.7	0	23
2021-01-15 06:00:00	-15.1	86	1.2	1	7	north wind	975	6.2	0	23
2021-01-15 07:00:00	-15.1	85	1.4	1	54	northeast wind	976	5.3	0	23
2021-01-15 08:00:00	-15.3	85	1.2	1	78	east wind	976	6.7	0	23
2021-01-15 09:00:00	-15.3	85	0.9	1	98	east wind	976	5.5	0	10
2021-01-15 10:00:00	-15.7	84	1.6	2	48	northeast wind	977	1.8	0	10
2021-01-15 11:00:00	-15.8	82	1.7	2	55	northeast wind	977	4.6	0	10
2021-01-15 12:00:00	-15.9	80	1.9	2	48	northeast wind	978	8.6	0	23
2021-01-15 13:00:00	-15.6	77	1.8	2	17	north wind	977	14.4	0	23
2021-01-15 14:00:00	-14.9	73	1.8	2	35	northeast wind	977	10.4	0	23
2021-01-15 15:00:00	-13.8	66	0.9	1	322	northwest wind	977	18.8	0	82
2021-01-15 16:00:00	-13.8	64	1.3	1	357	north wind	978	19.7	0	82
2021-01-15 17:00:00	-14.4	70	1.9	2	359	north wind	978	21.4	0	82
2021-01-15 18:00:00	-15	72	1.1	1	5	north wind	979	18.6	0	23
2021-01-15 19:00:00	-16.5	76	0.9	1	338	north wind	980	18.6	0	23
2021-01-15 20:00:00	-18.9	79	0.9	1	322	northwest wind	981	11.1	0	23
2021-01-15 21:00:00	-21.2	78	1	1	257	west wind	981	9.8	0	82
2021-01-15 22:00:00	-22.8	78	1	1	268	west wind	981	10.7	0	82
2021-01-15 23:00:00	-23.3	78	1.6	2	264	west wind	980	11	0	82
2021-01-16 00:00:00	-23.7	79	1.4	1	249	west wind	981	9.9	0	82
2021-01-16 01:00:00	-23.7	79	1.4	1	262	west wind	981	7	0	82
2021-01-16 02:00:00	-22.6	79	1.9	2	248	west wind	981	9.3	0	82
2021-01-16 03:00:00	-21.7	80	1.8	2	252	west wind	981	12.8	0	82
2021-01-16 04:00:00	-22.2	79	1.5	1	261	west wind	981	12.6	0	82
2021-01-16 05:00:00	-23.1	79	0.7	1	292	west wind	981	13.4	0	82
2021-01-16 06:00:00	-23.8	79	1.7	2	255	west wind	981	12.8	0	82
2021-01-16 07:00:00	-24	78	2.3	2	257	west wind	981	12.7	0	82
2021-01-16 08:00:00	-23.9	77	1.8	2	261	west wind	981	13	0	82
2021-01-16 09:00:00	-23.8	77	2.6	2	267	west wind	981	12.2	0	82
2021-01-16 10:00:00	-22.8	77	2.9	2	255	west wind	981	5.3	0	82
2021-01-16 11:00:00	-21.4	76	1.8	2	275	west wind	981	9.7	0	82
2021-01-16 12:00:00	-19	74	0.5	1	308	northwest wind	980	9.5	0	82
2021-01-16 13:00:00	-17.2	66	0.9	1	231	southwest wind	980	10.9	0	82
2021-01-16 14:00:00	-16.4	69	1.3	1	337	northwest wind	979	8.2	0	82
2021-01-16 15:00:00	-15.6	68	1.3	1	41	northeast wind	977	10.3	0	82
2021-01-16 16:00:00	-15.7	68	1.2	1	43	northeast wind	977	12.1	0	82

2021-01-16 17:00:00	-15.4	66	1.1	1	203	southwest wind	976	10.3	0	82
2021-01-16 18:00:00	-17	71	1.1	1	83	east wind	976	9.9	0	82
2021-01-16 19:00:00	-19	79	0.8	1	122	southeast wind	975	7.8	0	82
2021-01-16 20:00:00	-20	83	1	1	313	northwest wind	975	6.6	0	82
2021-01-16 21:00:00	-21.9	81	1	1	267	west wind	974	6.9	0	82
2021-01-16 22:00:00	-23.7	81	0	0	0	north wind	972	7.2	0	82
2021-01-16 23:00:00	-24.4	79	0.6	1	258	west wind	971	7.6	0	82
2021-01-17 00:00:00	-24.6	78	1.3	1	316	northwest wind	970	8.6	0	82
2021-01-17 01:00:00	-25.3	79	0.3	1	273	west wind	969	13.7	0	82
2021-01-17 02:00:00	-25.4	78	0.7	1	42	northeast wind	968	13	0	82
2021-01-17 03:00:00	-25	78	0.5	1	184	south wind	968	7.6	0	82
2021-01-17 04:00:00	-23.7	85	1	1	320	northwest wind	967	6.1	0	82
2021-01-17 05:00:00	-23.8	84	1.1	1	299	northwest wind	967	7.4	0	82
2021-01-17 06:00:00	-25.3	80	0.7	1	268	west wind	966	6.6	0	82
2021-01-17 07:00:00	-25.4	78	0.7	1	272	west wind	966	9.8	0	82
2021-01-17 08:00:00	-25.7	78	0.9	1	271	west wind	966	8	0	82
2021-01-17 09:00:00	-25.3	79	1.4	1	259	west wind	966	6.2	0	82
2021-01-17 10:00:00	-24	81	1.5	1	264	west wind	965	5.4	0	82
2021-01-17 11:00:00	-21.3	82	0.8	1	274	west wind	965	8.9	0	82
2021-01-17 12:00:00	-16.4	80	1.8	2	360	north wind	964	14.1	0	82
2021-01-17 13:00:00	-12.6	63	2.3	2	47	northeast wind	964	10.1	0	82
2021-01-17 14:00:00	-13.1	62	2	2	47	northeast wind	963	11.2	0	82
2021-01-17 15:00:00	-12.4	60	1.4	1	21	north wind	962	11.9	0	82
2021-01-17 16:00:00	-11.2	55	1	1	6	north wind	962	12.6	0	82
2021-01-17 17:00:00	-11.2	56	1.1	1	350	north wind	962	13.2	0	82
2021-01-17 18:00:00	-12.2	58	1	1	355	north wind	962	13.8	0	23
2021-01-17 19:00:00	-13.3	65	0.8	1	256	west wind	962	8.3	0	23
2021-01-17 20:00:00	-16.1	71	0.6	1	258	west wind	962	7.7	0	23
2021-01-17 21:00:00	-18.5	77	0.1	0	0	north wind	962	7.3	0	82
2021-01-17 22:00:00	-19	79	0.9	1	302	northwest wind	962	7.9	0	82
2021-01-17 23:00:00	-20.2	79	0.6	1	267	west wind	962	8.1	0	82
2021-01-18 00:00:00	-21.1	81	0.7	1	322	northwest wind	961	10.1	0	82
2021-01-18 01:00:00	-21.7	80	0.2	0	0	north wind	961	8.9	0	82
2021-01-18 02:00:00	-22.4	81	0.7	1	296	northwest wind	960	8.7	0	82
2021-01-18 03:00:00	-22.7	82	1	1	6	north wind	960	9.5	0	82
2021-01-18 04:00:00	-23.3	81	0.6	1	255	west wind	960	9.9	0	82
2021-01-18 05:00:00	-23.7	81	0.9	1	258	west wind	959	9.7	0	82
2021-01-18 06:00:00	-23.3	82	1.5	1	253	west wind	959	9.6	0	82

2021-01-18 07:00:00	-24	80	0.8	1	269	west wind	958	9.7	0	82
2021-01-18 08:00:00	-23.2	80	1.7	2	255	west wind	958	8.7	0	82
2021-01-18 09:00:00	-22.3	80	1.7	2	267	west wind	958	8.8	0	82
2021-01-18 10:00:00	-23	77	1.2	1	285	west wind	958	9	0	82
2021-01-18 11:00:00	-20.9	80	0.4	1	305	northwest wind	958	6.7	0	82
2021-01-18 12:00:00	-16.8	77	0.4	1	343	north wind	958	6.3	0	82
2021-01-18 13:00:00	-15.2	73	0.9	1	116	southeast wind	957	7.1	0	82
2021-01-18 14:00:00	-14.1	65	0.9	1	97	east wind	956	7.5	0	82
2021-01-18 15:00:00	-13.3	65	1.2	1	132	southeast wind	955	7.6	0	82
2021-01-18 16:00:00	-12.7	62	1	1	150	southeast wind	955	7.8	0	82
2021-01-18 17:00:00	-12	61	1.2	1	141	southeast wind	955	7.6	0	82
2021-01-18 18:00:00	-14.5	70	1.5	1	103	east wind	956	7.1	0	82
2021-01-18 19:00:00	-16.4	77	1	1	339	north wind	956	5.9	0	82
2021-01-18 20:00:00	-17.5	80	0.6	1	332	northwest wind	956	5.4	0	82
2021-01-18 21:00:00	-18.4	82	1.3	1	352	north wind	957	4.6	0	82
2021-01-18 22:00:00	-19.1	84	1.1	1	357	north wind	956	4.2	0	82
2021-01-18 23:00:00	-19.6	84	1	1	356	north wind	957	4.8	0	82
2021-01-19 00:00:00	-19.8	84	0.7	1	286	west wind	957	5.7	0	82
2021-01-19 01:00:00	-21	81	0.4	1	283	west wind	957	6	0	82
2021-01-19 02:00:00	-21.1	81	0.8	1	323	northwest wind	957	5.7	0	82
2021-01-19 03:00:00	-21.7	80	0.8	1	259	west wind	957	5.9	0	82
2021-01-19 04:00:00	-22.1	80	0.1	0	0	north wind	957	6.3	0	82
2021-01-19 05:00:00	-21.8	81	0.6	1	279	west wind	957	6.9	0	82
2021-01-19 06:00:00	-21.8	80	1.1	1	263	west wind	957	6.6	0	82
2021-01-19 07:00:00	-21.7	80	1.1	1	250	west wind	957	6.3	0	82
2021-01-19 08:00:00	-20.6	81	1.4	1	263	west wind	957	6.3	0	82
2021-01-19 09:00:00	-20	81	1.7	2	256	west wind	958	5.5	0	90
2021-01-19 10:00:00	-18.8	81	1.3	1	265	west wind	958	5.4	0	90
2021-01-19 11:00:00	-17.2	80	1.1	1	248	west wind	959	6.1	0	90
2021-01-19 12:00:00	-14.6	73	0.9	1	355	north wind	959	6.5	0	90
2021-01-19 13:00:00	-13.2	67	1	1	316	northwest wind	959	6.9	0	90
2021-01-19 14:00:00	-11.5	63	1.3	1	126	southeast wind	958	7.3	0	90
2021-01-19 15:00:00	-11.8	64	1.5	1	111	east wind	957	7.2	0	90
2021-01-19 16:00:00	-12.8	68	1.5	1	97	east wind	957	6.4	0	90
2021-01-19 17:00:00	-12.9	68	1.9	2	114	southeast wind	957	6.7	0	90
2021-01-19 18:00:00	-14	72	0.9	1	28	northeast wind	958	6.4	0	90
2021-01-19 19:00:00	-15.1	76	0.6	1	29	northeast wind	958	5.9	0	90
2021-01-19 20:00:00	-16.5	78	1.1	1	183	south wind	959	5.2	0	90

2021-01-19 21:00:00	-17.3	81	0.5	1	358	north wind	958	4.8	0	82
2021-01-19 22:00:00	-17.2	83	1.1	1	287	west wind	959	5.2	0	82
2021-01-19 23:00:00	-17.3	81	0.2	0	0	north wind	959	5.7	0	82
2021-01-20 00:00:00	-17.2	81	1.1	1	249	west wind	958	4.5	0	82
2021-01-20 01:00:00	-17.4	82	0.2	0	0	north wind	958	4	0	82
2021-01-20 02:00:00	-17.6	83	1.2	1	327	northwest wind	958	4.3	0	82
2021-01-20 03:00:00	-18.1	84	1	1	341	north wind	958	4.9	0	82
2021-01-20 04:00:00	-17.9	84	0.7	1	14	north wind	957	5.4	0	82
2021-01-20 05:00:00	-18.2	83	0.2	0	0	north wind	957	5.1	0	82
2021-01-20 06:00:00	-17.7	83	0.7	1	275	west wind	957	6.1	0	82
2021-01-20 07:00:00	-17.4	82	0.9	1	345	north wind	958	5.7	0	82
2021-01-20 08:00:00	-18.1	82	0.6	1	302	northwest wind	958	4.9	0	82
2021-01-20 09:00:00	-18	83	0.7	1	319	northwest wind	959	4.7	0	90
2021-01-20 10:00:00	-18.4	84	1.4	1	259	west wind	960	3.4	0	90
2021-01-20 11:00:00	-17.7	81	1	1	221	southwest wind	960	4.7	0	90
2021-01-20 12:00:00	-14.6	77	0.4	1	356	north wind	960	4.9	0	23
2021-01-20 13:00:00	-11.8	67	1	1	115	southeast wind	960	5.1	0	23
2021-01-20 14:00:00	-11.6	66	1.6	2	116	southeast wind	960	6.1	0	23
2021-01-20 15:00:00	-11.7	66	1.7	2	56	northeast wind	960	5.7	0	90
2021-01-20 16:00:00	-10.5	65	1.3	1	152	southeast wind	961	5.5	0	90
2021-01-20 17:00:00	-10.2	65	1.1	1	125	southeast wind	961	5.8	0	90
2021-01-20 18:00:00	-11.3	70	1.8	2	98	east wind	961	5.8	0	90
2021-01-20 19:00:00	-13.1	76	1.2	1	84	east wind	962	5.7	0	90
2021-01-20 20:00:00	-14.4	79	1.5	1	19	north wind	963	4.7	0	90
2021-01-20 21:00:00	-15.5	83	1	1	325	northwest wind	963	4.9	0	82
2021-01-20 22:00:00	-15.7	83	0.6	1	325	northwest wind	963	4	0	82
2021-01-20 23:00:00	-15.7	84	0.6	1	160	south wind	963	3.7	0	82
2021-01-21 00:00:00	-15.4	83	1	1	321	northwest wind	963	4	0	82
2021-01-21 01:00:00	-15.8	83	0.8	1	284	west wind	962	3.5	0	82
2021-01-21 02:00:00	-16.8	82	0.5	1	311	northwest wind	962	4.1	0	82
2021-01-21 03:00:00	-17	85	1	1	11	north wind	962	4.5	0	82
2021-01-21 04:00:00	-18.2	83	0.8	1	283	west wind	963	3.6	0	82
2021-01-21 05:00:00	-17.8	85	0.7	1	341	north wind	963	4.3	0	82
2021-01-21 06:00:00	-17.9	85	1.1	1	340	north wind	963	5.2	0	82
2021-01-21 07:00:00	-18.1	84	0.7	1	247	southwest wind	963	4.7	0	82
2021-01-21 08:00:00	-17.5	84	1.9	2	255	west wind	962	4	0	82
2021-01-21 09:00:00	-16.5	85	1.5	1	251	west wind	962	3.8	0	82
2021-01-21 10:00:00	-15.7	84	1	1	247	southwest wind	961	3.6	0	82

2021-01-21 11:00:00	-15.1	83	0.6	1	343	north wind	961	3.9	0	82
2021-01-21 12:00:00	-13.6	78	1	1	17	north wind	960	5	0	82
2021-01-21 13:00:00	-11.3	65	0.9	1	121	southeast wind	960	5.5	0	82
2021-01-21 14:00:00	-10.8	64	1.5	1	58	northeast wind	959	5.5	0	82
2021-01-21 15:00:00	-9.2	59	0.9	1	196	south wind	958	6	0	82
2021-01-21 16:00:00	-9.7	62	1.1	1	70	east wind	957	5.6	0	82
2021-01-21 17:00:00	-10.9	67	2.3	2	92	east wind	956	5.2	0	82
2021-01-21 18:00:00	-12.1	69	1	1	24	northeast wind	956	5.9	0	23
2021-01-21 19:00:00	-13.3	72	1.1	1	160	south wind	956	5.9	0	23
2021-01-21 20:00:00	-14	75	0.6	1	276	west wind	956	4.8	0	23
2021-01-21 21:00:00	-14.6	78	1.3	1	259	west wind	955	4.2	0	82
2021-01-21 22:00:00	-14.9	80	1.4	1	259	west wind	955	4.6	0	82
2021-01-21 23:00:00	-14.9	80	2.4	2	252	west wind	954	4.7	0	82
2021-01-22 00:00:00	-15	80	2.2	2	253	west wind	953	4	0	82
2021-01-22 01:00:00	-14.8	81	1.7	2	246	southwest wind	952	4	0	82
2021-01-22 02:00:00	-15	81	1.1	1	265	west wind	951	4.5	0	82
2021-01-22 03:00:00	-16.6	81	1.1	1	26	northeast wind	950	2.7	0	82
2021-01-22 04:00:00	-16.8	85	0.6	1	309	northwest wind	950	2.6	0	82
2021-01-22 05:00:00	-17.6	83	1.1	1	261	west wind	950	3.3	0	82
2021-01-22 06:00:00	-18.3	83	0.8	1	333	northwest wind	949	2.9	0	82
2021-01-22 07:00:00	-17.9	84	1.7	2	260	west wind	949	2.9	0	82
2021-01-22 08:00:00	-17.9	83	1.4	1	267	west wind	949	3.3	0	82
2021-01-22 09:00:00	-18.1	83	1	1	318	northwest wind	948	2.8	0	82
2021-01-22 10:00:00	-17.1	84	1.2	1	297	northwest wind	948	5.4	0	82
2021-01-22 11:00:00	-11.5	88	2.3	2	263	west wind	948	12.7	0	82
2021-01-22 12:00:00	-7.4	73	2.3	2	268	west wind	948	9.3	0	82
2021-01-22 13:00:00	-4.6	68	1.9	2	259	west wind	947	12.7	0	82
2021-01-22 14:00:00	-2.5	63	1	1	307	northwest wind	947	12	0	82
2021-01-22 15:00:00	-5	60	1.3	1	69	east wind	947	3.6	0	90
2021-01-22 16:00:00	-7	60	2.6	2	75	east wind	947	3.2	0	90
2021-01-22 17:00:00	-6.5	58	1.8	2	26	northeast wind	948	2.9	0	90
2021-01-22 18:00:00	-8.3	64	1	1	336	northwest wind	950	2.5	0	90
2021-01-22 19:00:00	-8	69	1.2	1	240	southwest wind	952	2.2	0	90
2021-01-22 20:00:00	-7.2	69	1.2	1	314	northwest wind	953	2.8	0	90
2021-01-22 21:00:00	-6.2	73	1.3	1	212	southwest wind	955	2.7	0	90
2021-01-22 22:00:00	-5.8	74	1	1	222	southwest wind	957	2.7	0	90
2021-01-22 23:00:00	-5.3	79	1.3	1	316	northwest wind	958	0.9	0	90
2021-01-23 00:00:00	-5.5	91	1	1	166	south wind	959	0.7	0	90

2021-01-23 01:00:00	-5.1	94	1.1	1	259	west wind	961	0.8	1.5	90
2021-01-23 02:00:00	-4.6	95	0.8	1	172	south wind	961	1.3	1	90
2021-01-23 03:00:00	-4.6	96	0.2	0			962	1.4	0.6	90
2021-01-23 04:00:00	-4.5	96	0.8	1	259	west wind	963	2	0.8	90
2021-01-23 05:00:00	-14.4	96	1.2	1	170	south wind	964	1.5	0.9	90
2021-01-23 06:00:00	-5.8	96	2.4	2	164	south wind	965	3.8	0.7	90
2021-01-23 07:00:00	-6.5	92	1.3	1	150	southeast wind	966	13.6	0.7	90
2021-01-23 08:00:00	-7.8	83	2.2	2	152	southeast wind	967	14.6	0.2	90
2021-01-23 09:00:00	-7.8	82	2.1	2	129	southeast wind	968	16.1	0	90
2021-01-23 10:00:00	-8.1	80	0.5	1	240	southwest wind	969	14.5	0	90
2021-01-23 11:00:00	-8	77	0.9	1	268	west wind	969	18.8	0	90
2021-01-23 12:00:00	-7.6	71	1	1	244	southwest wind	969	17.2	0	90
2021-01-23 13:00:00	-6.2	67	1.6	2	178	south wind	969	24.8	0	90
2021-01-23 14:00:00	-6.1	67	1.4	1	193	south wind	969	30	0	90
2021-01-23 15:00:00	-5.6	58	1.7	2	174	south wind	968	30	0	82
2021-01-23 16:00:00	-5.5	59	2	2	123	southeast wind	968	30	0	82
2021-01-23 17:00:00	-6.2	63	1.3	1	127	southeast wind	968	30	0	82
2021-01-23 18:00:00	-7.8	67	0.7	1	216	southwest wind	969	25.7	0	23
2021-01-23 19:00:00	-9.9	75	0.8	1	130	southeast wind	969	15.5	0	23
2021-01-23 20:00:00	-11.6	82	0.5	1	274	west wind	970	13.3	0	23
2021-01-23 21:00:00	-13.5	81	0.7	1	273	west wind	970	10.5	0	90
2021-01-23 22:00:00	-14.7	83	2.1	2	247	southwest wind	969	15.8	0	90
2021-01-23 23:00:00	-15.2	82	2.3	2	259	west wind	969	19.4	0	90
2021-01-24 00:00:00	-16.3	84	1.6	2	263	west wind	968	20.9	0	90
2021-01-24 01:00:00	-15.6	84	1.5	1	250	west wind	969	19	0	90
2021-01-24 02:00:00	-15.6	83	1.8	2	263	west wind	968	23.5	0	90
2021-01-24 03:00:00	-15.4	84	1.8	2	255	west wind	968	22.4	0	90
2021-01-24 04:00:00	-14.9	81	0.8	1	270	west wind	968	24.1	0	90
2021-01-24 05:00:00	-13.9	83	1.3	1	250	west wind	968	22	0	90
2021-01-24 06:00:00	-13.6	80	2.3	2	254	west wind	968	19.4	0	90
2021-01-24 07:00:00	-14.3	82	1.3	1	254	west wind	969	18.2	0	90
2021-01-24 08:00:00	-14	84	1	1	271	west wind	969	27	0	90
2021-01-24 09:00:00	-15.5	79	0.8	1	336	northwest wind	969	17.8	0	82
2021-01-24 10:00:00	-15.9	82	1.4	1	251	west wind	970	18.3	0	82
2021-01-24 11:00:00	-13.5	73	1.8	2	253	west wind	970	21.8	0	82
2021-01-24 12:00:00	-11.5	66	2	2	257	west wind	970	21.2	0	82
2021-01-24 13:00:00	-9.6	58	1.5	1	305	northwest wind	970	30	0	82
2021-01-24 14:00:00	-7.3	62	1.4	1	41	northeast wind	969	28	0	82

2021-01-24 15:00:00	-6.8	64	1.3	1	51	northeast wind	969	24.8	0	90
2021-01-24 16:00:00	-6.4	65	0.9	1	12	north wind	969	19.8	0	90
2021-01-24 17:00:00	-6.4	62	1.3	1	342	north wind	969	24.2	0	90
2021-01-24 18:00:00	-7	65	1.6	2	46	northeast wind	969	22.6	0	90
2021-01-24 19:00:00	-9.2	72	0.6	1	126	southeast wind	970	8.6	0	90
2021-01-24 20:00:00	-9.6	78	1.2	1	317	northwest wind	971	14.5	0	90
2021-01-24 21:00:00	-10.4	80	1	1	320	northwest wind	971	14.2	0	82
2021-01-24 22:00:00	-11.3	81	0.3	1	253	west wind	971	9.8	0	82
2021-01-24 23:00:00	-12.3	81	1.1	1	207	southwest wind	971	11.2	0	82
2021-01-25 00:00:00	-13.2	85	0.7	1	310	northwest wind	971	9.7	0	82
2021-01-25 01:00:00	-14.8	87	0.8	1	275	west wind	972	12.2	0	82
2021-01-25 02:00:00	-15.9	86	0.8	1	259	west wind	972	13	0	82
2021-01-25 03:00:00	-16.9	87	1.4	1	261	west wind	972	14.5	0	82
2021-01-25 04:00:00	-17.1	85	1.1	1	266	west wind	973	15.4	0	82
2021-01-25 05:00:00	-17.3	87	1.9	2	253	west wind	973	21.5	0	82
2021-01-25 06:00:00	-17.2	85	1.9	2	263	west wind	973	23.7	0	82
2021-01-25 07:00:00	-16.9	82	2.5	2	257	west wind	973	25.1	0	82
2021-01-25 08:00:00	-16.5	81	2.4	2	264	west wind	973	23.3	0	82
2021-01-25 09:00:00	-16.3	80	1.8	2	275	west wind	973	18.9	0	23
2021-01-25 10:00:00	-16.6	81	2.3	2	262	west wind	973	20.5	0	23
2021-01-25 11:00:00	-15.2	78	2.8	2	255	west wind	973	18.3	0	23
2021-01-25 12:00:00	-12.4	71	2.8	2	260	west wind	973	22.4	0	82
2021-01-25 13:00:00	-9.4	63	1	1	268	west wind	972	22.8	0	82
2021-01-25 14:00:00	-7.2	59	1.1	1	215	southwest wind	971	23.3	0	82
2021-01-25 15:00:00	-5.7	56	1	1	178	south wind	970	27.8	0	23
2021-01-25 16:00:00	-7.1	63	2.6	2	65	northeast wind	970	22.5	0	23
2021-01-25 17:00:00	-8	65	2.1	2	71	east wind	970	24.6	0	23
2021-01-25 18:00:00	-8.8	69	1.5	1	70	east wind	970	21.4	0	23
2021-01-25 19:00:00	-9.8	74	2.1	2	31	northeast wind	971	21.4	0	23
2021-01-25 20:00:00	-11.2	77	1.2	1	15	north wind	972	16.3	0	23
2021-01-25 21:00:00	-13.7	81	0.2	0			972	7.4	0	90
2021-01-25 22:00:00	-13.4	85	2.4	2	251	west wind	972	3.6	0	90
2021-01-25 23:00:00	-13.5	84	1.7	2	246	southwest wind	972	4.9	0	90
2021-01-26 00:00:00	-13.1	85	1.9	2	253	west wind	971	5.5	0	90
2021-01-26 01:00:00	-12.4	86	1.3	1	267	west wind	971	4.6	0	90
2021-01-26 02:00:00	-12.2	86	0.8	1	326	northwest wind	970	6.6	0	90
2021-01-26 03:00:00	-12.3	88	0.2	0			970	6.2	0	90
2021-01-26 04:00:00	-12.4	88	0.3	1	44	northeast wind	970	6.2	0	90

2021-01-26 05:00:00	-13	88	0.8	1	244	southwest wind	970	6	0	90
2021-01-26 06:00:00	-13.6	88	1.1	1	244	southwest wind	969	5.7	0	90
2021-01-26 07:00:00	-15.8	86	0.7	1	261	west wind	970	10.4	0	90
2021-01-26 08:00:00	-16.3	87	1.6	2	255	west wind	970	13.1	0	90
2021-01-26 09:00:00	-17.9	84	1.2	1	251	west wind	970	9	0	82
2021-01-26 10:00:00	-18.3	83	1.6	2	253	west wind	971	10.7	0	82
2021-01-26 11:00:00	-16.9	80	1.7	2	263	west wind	971	8.6	0	82
2021-01-26 12:00:00	-14.4	75	1.4	1	242	southwest wind	971	10.4	0	82
2021-01-26 13:00:00	-12.3	68	1.8	2	251	west wind	971	12.3	0	82
2021-01-26 14:00:00	-10.1	62	2	2	254	west wind	970	16.9	0	82
2021-01-26 15:00:00	-8.3	57	1.4	1	254	west wind	969	22.4	0	23
2021-01-26 16:00:00	-6.2	49	0.8	1	238	southwest wind	969	23	0	23
2021-01-26 17:00:00	-7	51	1	1	7	north wind	969	22.8	0	23
2021-01-26 18:00:00	-8.6	64	1.5	1	93	east wind	970	14.1	0	23
2021-01-26 19:00:00	-11	76	1.6	2	55	northeast wind	970	11.6	0	23
2021-01-26 20:00:00	-13.3	81	0.8	1	329	northwest wind	972	6.4	0	23
2021-01-26 21:00:00	-15.7	80	1.4	1	301	northwest wind	972	6.4	0	23
2021-01-26 22:00:00	-16.7	84	1.3	1	278	west wind	973	9.8	0	23
2021-01-26 23:00:00	-17.9	81	1.1	1	269	west wind	973	13.5	0	23
2021-01-27 00:00:00	-19.1	80	0.7	1	267	west wind	973	12.8	0	23
2021-01-27 01:00:00	-19.7	81	1.2	1	268	west wind	973	17.9	0	23
2021-01-27 02:00:00	-19.8	80	1.9	2	258	west wind	973	18.4	0	23
2021-01-27 03:00:00	-19.6	81	1.2	1	271	west wind	973	17.2	0	23
2021-01-27 04:00:00	-19.9	82	1	1	281	west wind	973	16.3	0	23
2021-01-27 05:00:00	-21.5	79	0.9	1	260	west wind	973	16.6	0	23
2021-01-27 06:00:00	-21.5	81	1	1	268	west wind	973	14.1	0	23
2021-01-27 07:00:00	-21.3	82	1.5	1	270	west wind	973	9.3	0	23
2021-01-27 08:00:00	-19.8	83	1.8	2	260	west wind	974	5.3	0	23
2021-01-27 09:00:00	-19	83	1.3	1	254	west wind	974	3.7	0	82
2021-01-27 10:00:00	-17.7	84	1	1	250	west wind	974	2.3	0	82
2021-01-27 11:00:00	-16.2	83	1.3	1	254	west wind	974	7.7	0	82
2021-01-27 12:00:00	-14.6	80	1.8	2	255	west wind	974	6.7	0	10
2021-01-27 13:00:00	-12.7	75	1.5	1	259	west wind	974	8.4	0	10
2021-01-27 14:00:00	-11.2	69	1.5	1	239	southwest wind	973	15.4	0	10
2021-01-27 15:00:00	-10.7	64	1.2	1	275	west wind	973	14.4	0	82
2021-01-27 16:00:00	-10.2	61	1.6	2	251	west wind	973	18.3	0	82
2021-01-27 17:00:00	-9.6	59	1.1	1	310	northwest wind	973	16.4	0	82
2021-01-27 18:00:00	-10.6	64	1.1	1	14	north wind	973	17.6	0	23

2021-01-27 19:00:00	-12.1	71	1	1	344	north wind	973	10.2	0	23
2021-01-27 20:00:00	-15.2	80	1.6	2	252	west wind	974	9.6	0	23
2021-01-27 21:00:00	-16.9	79	1.3	1	262	west wind	974	10.7	0	82
2021-01-27 22:00:00	-18.2	77	1.6	2	263	west wind	974	9.7	0	82
2021-01-27 23:00:00	-18.3	80	2.1	2	265	west wind	974	8.9	0	82
2021-01-28 00:00:00	-18.5	78	2.3	2	264	west wind	973	8.6	0	82
2021-01-28 01:00:00	-18.7	80	1.5	1	273	west wind	973	10.4	0	82
2021-01-28 02:00:00	-18.6	80	2.1	2	280	west wind	973	13.6	0	82
2021-01-28 03:00:00	-20	80	1.2	1	264	west wind	972	12	0	82
2021-01-28 04:00:00	-19.9	79	1.8	2	267	west wind	972	14.8	0	82
2021-01-28 05:00:00	-19.9	80	1.9	2	259	west wind	972	17.9	0	82
2021-01-28 06:00:00	-20.4	78	1.5	1	264	west wind	972	21.1	0	82
2021-01-28 07:00:00	-21.2	78	1.3	1	275	west wind	972	20.6	0	82
2021-01-28 08:00:00	-21.6	79	1	1	258	west wind	972	17.9	0	82
2021-01-28 09:00:00	-22.1	81	1.1	1	267	west wind	973	12.6	0	82
2021-01-28 10:00:00	-20.7	82	1.8	2	266	west wind	973	10.4	0	82
2021-01-28 11:00:00	-18.5	77	1.9	2	264	west wind	973	8.6	0	82
2021-01-28 12:00:00	-14.8	71	1.1	1	309	northwest wind	973	11	0	82
2021-01-28 13:00:00	-11.6	71	1.1	1	296	northwest wind	973	10.8	0	82
2021-01-28 14:00:00	-9.6	65	0.8	1	320	northwest wind	972	11.8	0	82
2021-01-28 15:00:00	-9.2	64	1.1	1	254	west wind	972	11.9	0	23
2021-01-28 16:00:00	-8.3	60	1.2	1	257	west wind	971	13.2	0	23
2021-01-28 17:00:00	-8.6	62	1.4	1	114	southeast wind	972	12.6	0	23
2021-01-28 18:00:00	-10.3	69	1.9	2	76	east wind	972	11.7	0	23
2021-01-28 19:00:00	-12.1	74	0.7	1	86	east wind	972	9.8	0	23
2021-01-28 20:00:00	-13.4	78	0.4	1	336	northwest wind	973	7	0	23
2021-01-28 21:00:00	-15.3	82	0.7	1	270	west wind	973	6.1	0	82
2021-01-28 22:00:00	-17.1	82	1	1	270	west wind	973	7.9	0	82
2021-01-28 23:00:00	-18.3	80	1.3	1	262	west wind	973	8.7	0	82
2021-01-29 00:00:00	-18.6	82	1.8	2	260	west wind	973	10.1	0	82
2021-01-29 01:00:00	-19	81	1.8	2	263	west wind	973	10.8	0	82
2021-01-29 02:00:00	-18.9	81	2	2	264	west wind	973	12.1	0	82
2021-01-29 03:00:00	-19.5	81	1.3	1	277	west wind	973	13.6	0	82
2021-01-29 04:00:00	-19.5	82	1.4	1	271	west wind	973	14.2	0	82
2021-01-29 05:00:00	-20.7	78	1.1	1	275	west wind	973	13.7	0	82
2021-01-29 06:00:00	-21	81	0.7	1	274	west wind	973	14.8	0	82
2021-01-29 07:00:00	-21.7	81	1.3	1	260	west wind	973	14	0	82
2021-01-29 08:00:00	-21.3	82	1	1	258	west wind	973	7.4	0	82

2021-01-29 09:00:00	-20.7	83	1.4	1	265	west wind	974	7.8	0	82
2021-01-29 10:00:00	-20.9	81	1.2	1	258	west wind	975	6.6	0	82
2021-01-29 11:00:00	-19	80	1.5	1	252	west wind	975	7.5	0	82
2021-01-29 12:00:00	-16.1	77	1.2	1	256	west wind	975	6.1	0	82
2021-01-29 13:00:00	-13.1	75	0.9	1	316	northwest wind	975	6.9	0	82
2021-01-29 14:00:00	-11.4	71	0.9	1	329	northwest wind	974	7.8	0	82
2021-01-29 15:00:00	-10.1	67	0.7	1	346	north wind	974	9.6	0	23
2021-01-29 16:00:00	-9.2	62	1.2	1	310	northwest wind	973	10	0	23
2021-01-29 17:00:00	-10.7	70	1.7	2	77	east wind	973	9.8	0	23
2021-01-29 18:00:00	-12.2	76	1.8	2	96	east wind	973	8.2	0	23
2021-01-29 19:00:00	-13.4	80	0.8	1	89	east wind	973	6.5	0	23
2021-01-29 20:00:00	-14.9	86	1	1	330	northwest wind	973	5.3	0	23
2021-01-29 21:00:00	-16.8	84	1.1	1	267	west wind	973	5.9	0	82
2021-01-29 22:00:00	-18.5	83	1.5	1	260	west wind	973	6.4	0	82
2021-01-29 23:00:00	-19.2	82	1.9	2	259	west wind	972	6.4	0	82
2021-01-30 00:00:00	-19.6	82	1.2	1	259	west wind	972	5.5	0	82
2021-01-30 01:00:00	-20	82	1.4	1	265	west wind	972	4.9	0	82
2021-01-30 02:00:00	-19.6	82	1.9	2	263	west wind	971	6.7	0	82
2021-01-30 03:00:00	-20.1	81	1.9	2	262	west wind	971	6.7	0	82
2021-01-30 04:00:00	-20.6	80	1.3	1	268	west wind	971	7.9	0	82
2021-01-30 05:00:00	-21	80	1.6	2	273	west wind	970	9.3	0	82
2021-01-30 06:00:00	-21.7	79	0.9	1	270	west wind	970	9.4	0	82
2021-01-30 07:00:00	-22.4	79	0.9	1	286	west wind	970	5.6	0	82
2021-01-30 08:00:00	-22.1	81	1.1	1	259	west wind	970	5.5	0	82
2021-01-30 09:00:00	-19.8	84	1.2	1	271	west wind	970	3.3	0	82
2021-01-30 10:00:00	-19.7	83	2.3	2	255	west wind	970	2.6	0	82
2021-01-30 11:00:00	-18	85	1.9	2	251	west wind	970	3.6	0	82
2021-01-30 12:00:00	-15.3	81	1.6	2	241	southwest wind	970	4.5	0	82
2021-01-30 13:00:00	-12.7	73	1.1	1	187	south wind	969	5.1	0	82
2021-01-30 14:00:00	-10.1	63	0.8	1	174	south wind	969	5.6	0	82
2021-01-30 15:00:00	-10.1	67	1.4	1	8	north wind	968	5.3	0	23
2021-01-30 16:00:00	-8.9	64	0.8	1	208	southwest wind	968	5.4	0	23
2021-01-30 17:00:00	-9.9	67	1.9	2	126	southeast wind	968	5.5	0	23
2021-01-30 18:00:00	-11.2	74	1.4	1	85	east wind	967	5.1	0	23
2021-01-30 19:00:00	-13.3	80	0.9	1	72	east wind	968	3.4	0	23
2021-01-30 20:00:00	-15.1	86	1.3	1	350	north wind	968	2.5	0	23
2021-01-30 21:00:00	-16.8	85	1	1	301	northwest wind	968	2.9	0	82
2021-01-30 22:00:00	-18.6	82	1.1	1	256	west wind	968	3.4	0	82

2021-01-30 23:00:00	-19.4	82	1.3	1	265	west wind	967	4.7	0	82
2021-01-31 00:00:00	-19.7	81	1.9	2	257	west wind	967	4.5	0	82
2021-01-31 01:00:00	-20.6	79	1	1	299	northwest wind	967	6.3	0	82
2021-01-31 02:00:00	-20	80	1.5	1	280	west wind	966	9.1	0	82
2021-01-31 03:00:00	-21.7	78	0.6	1	275	west wind	966	5	0	82
2021-01-31 04:00:00	-20.6	80	1.3	1	258	west wind	965	5.3	0	82
2021-01-31 05:00:00	-20.9	80	1.4	1	266	west wind	965	5.2	0	82
2021-01-31 06:00:00	-20.7	83	2	2	253	west wind	965	4.9	0	82
2021-01-31 07:00:00	-20.7	82	1.6	2	268	west wind	965	4.8	0	82
2021-01-31 08:00:00	-19.8	87	1.2	1	260	west wind	964	4.6	0	82
2021-01-31 09:00:00	-20.9	82	1.4	1	263	west wind	965	4.7	0	82
2021-01-31 10:00:00	-20.7	82	2.1	2	260	west wind	965	2.3	0	82
2021-01-31 11:00:00	-18.9	82	2.5	2	252	west wind	965	5.8	0	82
2021-01-31 12:00:00	-16	80	2.7	2	255	west wind	965	6.6	0	82
2021-01-31 13:00:00	-13.2	75	2.7	2	253	west wind	964	6.9	0	82
2021-01-31 14:00:00	-10.9	70	1.7	2	279	west wind	964	7.2	0	82
2021-01-31 15:00:00	-9.1	64	1.1	1	331	northwest wind	963	7.5	0	82
2021-01-31 16:00:00	-8.8	61	1.2	1	127	southeast wind	962	6.4	0	82
2021-01-31 17:00:00	-9.7	67	1.6	2	115	southeast wind	963	5.3	0	82
2021-01-31 18:00:00	-11.4	74	2	2	95	east wind	963	4.8	0	23
2021-01-31 19:00:00	-13.6	81	1.2	1	39	northeast wind	963	3.6	0	23
2021-01-31 20:00:00	-15.3	86	1.3	1	344	north wind	964	2.6	0	23
2021-01-31 21:00:00	-16.5	87	1.1	1	317	northwest wind	964	1.3	0	82
2021-01-31 22:00:00	-17.9	86	1.1	1	263	west wind	964	3.2	0	82
2021-01-31 23:00:00	-18.8	85	1.7	2	267	west wind	964	4	0	82
2021-02-01 00:00:00	-19.8	83	1	1	283	west wind	963	5.6	0	82
2021-02-01 01:00:00	-20.9	82	1.3	1	261	west wind	963	5.3	0	82
2021-02-01 02:00:00	-20.7	82	1.5	1	266	west wind	962	6.2	0	82
2021-02-01 03:00:00	-20.9	81	1.3	1	273	west wind	962	7.1	0	82
2021-02-01 04:00:00	-22.2	79	0.5	1	290	west wind	962	13.2	0	82
2021-02-01 05:00:00	-21.7	81	0.9	1	273	west wind	962	11.3	0	82
2021-02-01 06:00:00	-21	82	1.2	1	269	west wind	962	9.1	0	82
2021-02-01 07:00:00	-20.5	81	1.3	1	264	west wind	962	6	0	82
2021-02-01 08:00:00	-19.4	83	0.9	1	252	west wind	963	5.3	0	82
2021-02-01 09:00:00	-18.1	84	0.9	1	248	west wind	963	4.6	0	90
2021-02-01 10:00:00	-17.7	83	1.2	1	264	west wind	964	3.6	0	90
2021-02-01 11:00:00	-16.1	83	1	1	255	west wind	964	3.8	0	90
2021-02-01 12:00:00	-13.3	79	1.2	1	256	west wind	964	4.7	0	82

2021-02-01 13:00:00	-10.3	71	1.3	1	229	southwest wind	963	5.4	0	82
2021-02-01 14:00:00	-8.3	63	0.7	1	299	northwest wind	963	6.1	0	82
2021-02-01 15:00:00	-8.2	61	1.9	2	59	northeast wind	962	6.4	0	23
2021-02-01 16:00:00	-8.5	65	1.7	2	48	northeast wind	963	5.9	0	23
2021-02-01 17:00:00	-8.7	67	1.2	1	94	east wind	963	5.4	0	23
2021-02-01 18:00:00	-9.7	70	0.5	1	345	north wind	964	5.4	0	23
2021-02-01 19:00:00	-10.2	74	0.5	1	225	southwest wind	964	4.5	0	23
2021-02-01 20:00:00	-10.8	77	0.5	1	162	south wind	965	4.8	0	23
2021-02-01 21:00:00	-11.2	78	0.4	1	350	north wind	965	3.5	0	82
2021-02-01 22:00:00	-12	81	1	1	323	northwest wind	965	3.5	0	82
2021-02-01 23:00:00	-12.6	83	0.3	1	265	west wind	965	3.1	0	82
2021-02-02 00:00:00	-11.8	85	1	1	274	west wind	965	3.5	0	82
2021-02-02 01:00:00	-11.5	83	0.4	1	229	southwest wind	964	3.3	0	82
2021-02-02 02:00:00	-11.4	83	0.7	1	137	southeast wind	964	3.2	0	82
2021-02-02 03:00:00	-12.6	84	0.1	0			965	2.7	0	82
2021-02-02 04:00:00	-12.8	87	0.7	1	315	northwest wind	965	2.8	0	82
2021-02-02 05:00:00	-12.2	86	0.5	1	150	southeast wind	965	2.8	0	82
2021-02-02 06:00:00	-12.3	84	1.4	1	100	east wind	965	3.4	0	82
2021-02-02 07:00:00	-13.5	88	1.1	1	30	northeast wind	966	3.2	0	82
2021-02-02 08:00:00	-13.6	88	1	1	33	northeast wind	966	3.2	0	82
2021-02-02 09:00:00	-13.6	88	0.7	1	18	north wind	966	3.2	0	82
2021-02-02 10:00:00	-13.7	88	0.4	1	304	northwest wind	966	3.1	0	82
2021-02-02 11:00:00	-13.4	87	0.6	1	229	southwest wind	967	3.2	0	82
2021-02-02 12:00:00	-11.8	81	0.9	1	3	north wind	967	3.7	0	82
2021-02-02 13:00:00	-10.6	75	1.1	1	90	east wind	967	3.9	0	82
2021-02-02 14:00:00	-10	71	1.5	1	1	north wind	967	4	0	82
2021-02-02 15:00:00	-8.3	66	1.1	1	192	south wind	966	4.1	0	82
2021-02-02 16:00:00	-7	60	0.7	1	172	south wind	966	3.9	0	82
2021-02-02 17:00:00	-9.4	72	2.3	2	80	east wind	966	2.8	0	82
2021-02-02 18:00:00	-10	74	1.3	1	87	east wind	967	2.8	0	23
2021-02-02 19:00:00	-11.1	76	0.8	1	344	north wind	968	2.9	0	23
2021-02-02 20:00:00	-12.5	80	0.5	1	312	northwest wind	968	2.4	0	23
2021-02-02 21:00:00	-13.6	83	0.7	1	303	northwest wind	969	2.3	0	23
2021-02-02 22:00:00	-14.5	84	0.9	1	263	west wind	969	2.4	0	23
2021-02-02 23:00:00	-15.1	85	1.8	2	271	west wind	969	2.6	0	23
2021-02-03 00:00:00	-15.1	85	2.6	2	256	west wind	968	2.7	0	23
2021-02-03 01:00:00	-13.7	85	1.2	1	261	west wind	968	2.8	0	23
2021-02-03 02:00:00	-12.7	84	2.8	2	264	west wind	968	5.9	0	23

2021-02-03 03:00:00	-13.6	81	1.2	1	323	northwest wind	968	9.9	0	23
2021-02-03 04:00:00	-14.7	82	1.2	1	308	northwest wind	968	19.9	0	23
2021-02-03 05:00:00	-15.6	82	0.9	1	251	west wind	968	14.1	0	23
2021-02-03 06:00:00	-15.8	83	0.5	1	248	west wind	968	15.9	0	23
2021-02-03 07:00:00	-16.2	84	0.8	1	260	west wind	969	16.9	0	23
2021-02-03 08:00:00	-16.1	85	1	1	266	west wind	969	10.6	0	23
2021-02-03 09:00:00	-15.4	87	0.9	1	270	west wind	969	13	0	23
2021-02-03 10:00:00	-16.3	86	0.4	1	223	southwest wind	970	8.6	0	23
2021-02-03 11:00:00	-11.8	82	0.8	1	304	northwest wind	970	11.5	0	23
2021-02-03 12:00:00	-8.6	66	1	1	149	southeast wind	970	10.2	0	23
2021-02-03 13:00:00	-6.3	62	1.6	2	114	southeast wind	969	4.7	0	23
2021-02-03 14:00:00	-7.6	67	1.9	2	109	east wind	969	4.4	0	23
2021-02-03 15:00:00	-7.4	66	2	2	104	east wind	969	4.6	0	23
2021-02-03 16:00:00	-6.5	64	0.9	1	121	southeast wind	968	4.4	0	23
2021-02-03 17:00:00	-6.3	65	0.6	1	158	south wind	968	4.6	0	23
2021-02-03 18:00:00	-7.7	76	1.7	2	100	east wind	968	4	0	23
2021-02-03 19:00:00	-8.6	82	1.4	1	103	east wind	968	3.1	0	23
2021-02-03 20:00:00	-9.4	85	0.5	1	354	north wind	968	2.5	0	23
2021-02-03 21:00:00	-9.8	87	0.9	1	352	north wind	968	2.5	0	82
2021-02-03 22:00:00	-10.4	89	1.5	1	23	northeast wind	968	1.9	0	82
2021-02-03 23:00:00	-10.9	91	1.1	1	5	north wind	968	1.6	0	82
2021-02-04 00:00:00	-11.6	92	0.7	1	137	southeast wind	968	1.3	0	82
2021-02-04 01:00:00	-11.9	93	1.2	1	342	north wind	968	0.2	0	82
2021-02-04 02:00:00	-11.5	92	1	1	33	northeast wind	968	0.5	0	82
2021-02-04 03:00:00	-11.7	92	0.8	1	339	north wind	968	0.2	0	82
2021-02-04 04:00:00	-11.9	91	0.8	1	297	northwest wind	967	0.3	0	82
2021-02-04 05:00:00	-12.1	91	0.8	1	305	northwest wind	967	1.5	0	82
2021-02-04 06:00:00	-12.7	90	1	1	268	west wind	967	1.3	0	82
2021-02-04 07:00:00	-12.5	91	1.1	1	256	west wind	966	1.9	0	82
2021-02-04 08:00:00	-12.3	91	1.4	1	307	northwest wind	966	2.4	0	82
2021-02-04 09:00:00	-12.3	90	0.8	1	144	southeast wind	966	1.9	0	82
2021-02-04 10:00:00	-11.9	91	0.6	1	221	southwest wind	966	1.8	0	82
2021-02-04 11:00:00	-11.2	91	0.7	1	351	north wind	966	2	0	82
2021-02-04 12:00:00	-10.5	91	1.3	1	47	northeast wind	966	2.7	0	90
2021-02-04 13:00:00	-9.9	92	1	1	32	northeast wind	966	4.2	0	90
2021-02-04 14:00:00	-8.9	92	1.3	1	16	north wind	965	4.4	0	90
2021-02-04 15:00:00	-7.5	80	1.2	1	101	east wind	965	4.9	0	90
2021-02-04 16:00:00	-7.5	72	1.9	2	69	east wind	964	4.7	0	90

2021-02-04 17:00:00	-6.7	69	0.9	1	332	northwest wind	964	4.7	0	90
2021-02-04 18:00:00	-6.8	69	1.1	1	335	northwest wind	964	4.7	0	82
2021-02-04 19:00:00	-8.1	76	1.3	1	109	east wind	964	3.9	0	82
2021-02-04 20:00:00	-9.5	82	0.7	1	313	northwest wind	964	3.4	0	82
2021-02-04 21:00:00	-10.6	86	0.9	1	348	north wind	964	3.1	0	90
2021-02-04 22:00:00	-11	88	1	1	344	north wind	964	3	0	90
2021-02-04 23:00:00	-11.3	89	0.4	1	287	west wind	963	2.9	0	90
2021-02-05 00:00:00	-11.3	88	0.8	1	271	west wind	963	3	0	90
2021-02-05 01:00:00	-11.5	86	0.9	1	276	west wind	963	3.1	0	90
2021-02-05 02:00:00	-12.2	89	1.1	1	251	west wind	962	2.7	0	90
2021-02-05 03:00:00	-13	89	0.9	1	282	west wind	962	2.9	0	90
2021-02-05 04:00:00	-13.2	86	1	1	301	northwest wind	962	3.9	0	90
2021-02-05 05:00:00	-14.3	86	0.9	1	291	west wind	962	5.1	0	90
2021-02-05 06:00:00	-14.6	86	0.5	1	314	northwest wind	962	5.4	0	90
2021-02-05 07:00:00	-14.7	86	0.7	1	261	west wind	961	5.9	0	90
2021-02-05 08:00:00	-14.2	86	0.9	1	296	northwest wind	961	3.7	0	90
2021-02-05 09:00:00	-14.1	87	0.8	1	257	west wind	962	2.9	0	90
2021-02-05 10:00:00	-14.3	87	1.2	1	256	west wind	962	2.6	0	90
2021-02-05 11:00:00	-12.9	86	1	1	251	west wind	962	2.8	0	90
2021-02-05 12:00:00	-10.7	82	0.7	1	78	east wind	962	2.7	0	82
2021-02-05 13:00:00	-6.6	65	0.9	1	144	southeast wind	961	4	0	82
2021-02-05 14:00:00	-5.3	56	1.1	1	151	southeast wind	960	3.8	0	82
2021-02-05 15:00:00	-4.2	66	1.5	1	106	east wind	960	3.5	0	82
2021-02-05 16:00:00	-4.5	69	2.1	2	88	east wind	960	3.2	0	82
2021-02-05 17:00:00	-5.4	71	1.6	2	108	east wind	960	3.2	0	82
2021-02-05 18:00:00	-5.7	73	1.7	2	112	east wind	961	3.5	0	82
2021-02-05 19:00:00	-7.2	77	1.1	1	106	east wind	961	3.1	0	82
2021-02-05 20:00:00	-8.6	83	0.6	1	77	east wind	962	2.3	0	82
2021-02-05 21:00:00	-10	88	0.8	1	293	northwest wind	962	2.1	0	23
2021-02-05 22:00:00	-11.3	87	1.2	1	263	west wind	962	2.6	0	23
2021-02-05 23:00:00	-12.6	86	1.1	1	266	west wind	961	2.8	0	23
2021-02-06 00:00:00	-13.1	85	1.3	1	258	west wind	961	3.6	0	23
2021-02-06 01:00:00	-13.8	85	1	1	266	west wind	961	3.6	0	23
2021-02-06 02:00:00	-14.3	84	1	1	285	west wind	961	4.7	0	23
2021-02-06 03:00:00	-15.5	85	0.9	1	271	west wind	961	4.7	0	23
2021-02-06 04:00:00	-15.9	84	1	1	260	west wind	960	4.7	0	23
2021-02-06 05:00:00	-16	85	1.8	2	261	west wind	960	1.8	0	23
2021-02-06 06:00:00	-15.2	90	2.1	2	255	west wind	960	3.9	0	23

2021-02-06 07:00:00	-14.4	89	1.4	1	254	west wind	960	3.2	0	23
2021-02-06 08:00:00	-13.2	90	1.9	2	267	west wind	960	2.3	0	23
2021-02-06 09:00:00	-12.5	90	1.1	1	268	west wind	960	2.2	0	90
2021-02-06 10:00:00	-12.3	88	1.1	1	293	northwest wind	961	2.8	0	90
2021-02-06 11:00:00	-11.6	86	0.8	1	329	northwest wind	961	3.3	0	90
2021-02-06 12:00:00	-9.5	83	0.6	1	161	south wind	961	3	0	90
2021-02-06 13:00:00	-7.4	74	1.1	1	163	south wind	961	2.8	0	90
2021-02-06 14:00:00	-5.8	69	0.8	1	223	southwest wind	960	3	0	90
2021-02-06 15:00:00	-5	69	1.7	2	121	southeast wind	959	2.9	0	23
2021-02-06 16:00:00	-5.3	71	2	2	118	southeast wind	959	3	0	23
2021-02-06 17:00:00	-6.1	75	1.9	2	80	east wind	960	2.8	0	23
2021-02-06 18:00:00	-6.9	78	1.4	1	131	southeast wind	961	2.8	0	23
2021-02-06 19:00:00	-7.7	82	0.9	1	209	southwest wind	961	2	0	23
2021-02-06 20:00:00	-9.8	88	0.5	1	141	southeast wind	961	1.6	0	23
2021-02-06 21:00:00	-11.6	89	0.6	1	285	west wind	961	2.1	0	23
2021-02-06 22:00:00	-12.8	88	1.2	1	264	west wind	961	2.5	0	23
2021-02-06 23:00:00	-14.7	86	0.2	0	314	northwest wind	961	2.6	0	23
2021-02-07 00:00:00	-15.2	85	1.8	2	349	north wind	960	2.5	0	23
2021-02-07 01:00:00	-15.9	86	0.7	1	268	west wind	960	2.6	0	23
2021-02-07 02:00:00	-16.1	88	2.5	2	254	west wind	960	1.9	0	23
2021-02-07 03:00:00	-14.9	91	1.7	2	250	west wind	960	1.3	0	23
2021-02-07 04:00:00	-13.1	90	1.8	2	242	southwest wind	960	2.1	0	23
2021-02-07 05:00:00	-13.6	89	1	1	308	northwest wind	960	2.3	0	23
2021-02-07 06:00:00	-13.8	89	1.3	1	307	northwest wind	961	4.2	0	23
2021-02-07 07:00:00	-13.6	89	0.6	1	13	north wind	962	3.8	0	23
2021-02-07 08:00:00	-13.8	90	1.4	1	308	northwest wind	963	2.2	0	23
2021-02-07 09:00:00	-12.3	91	0.9	1	288	west wind	963	1.9	0	90
2021-02-07 10:00:00	-11.8	91	0.8	1	178	south wind	964	1.8	0	90
2021-02-07 11:00:00	-10.7	92	1.2	1	298	northwest wind	964	1.4	0	90
2021-02-07 12:00:00	-9.3	92	1.1	1	231	southwest wind	964	2.2	0	90
2021-02-07 13:00:00	-7.7	84	1.2	1	246	southwest wind	963	2.8	0	90
2021-02-07 14:00:00	-5.9	73	0.9	1	138	southeast wind	963	3	0	90
2021-02-07 15:00:00	-6.2	81	2.4	2	84	east wind	964	2.1	0	90
2021-02-07 16:00:00	-6.6	81	0.9	1	109	east wind	964	2.1	0	90
2021-02-07 17:00:00	-6.3	81	1.4	1	7	north wind	965	2.1	0	90
2021-02-07 18:00:00	-6.4	82	1	1	153	southeast wind	965	2	0	90
2021-02-07 19:00:00	-6.7	85	1	1	125	southeast wind	966	2.2	0	90
2021-02-07 20:00:00	-6.5	85	1.4	1	297	northwest wind	965	2	0	90

2021-02-07 21:00:00	-7.2	86	1.5	1	130	southeast wind	965	1.6	0	90
2021-02-07 22:00:00	-7.7	89	0.4	1	97	east wind	964	1.4	0	90
2021-02-07 23:00:00	-9	91	0.8	1	359	north wind	964	1.5	0	90
2021-02-08 00:00:00	-10.2	91	0.8	1	282	west wind	963	1.4	0	90
2021-02-08 01:00:00	-11.2	90	0.7	1	337	northwest wind	962	1.6	0	90
2021-02-08 02:00:00	-12.2	90	1.1	1	283	west wind	962	1.9	0	90
2021-02-08 03:00:00	-12.8	88	1	1	264	west wind	962	2.1	0	90
2021-02-08 04:00:00	-13.7	88	1.1	1	262	west wind	962	2	0	90
2021-02-08 05:00:00	-13	91	0.5	1	105	east wind	962	0.2	0	90
2021-02-08 06:00:00	-12	92	0.7	1	347	north wind	962	0.2	0	90
2021-02-08 07:00:00	-12	92	0.5	1	276	west wind	961	0.2	0	90
2021-02-08 08:00:00	-12.4	91	0.6	1	172	south wind	961	0.4	0	90
2021-02-08 09:00:00	-12	91	0.6	1	211	southwest wind	961	0.7	0	82
2021-02-08 10:00:00	-11.2	92	0.9	1	308	northwest wind	961	0.2	0	82
2021-02-08 11:00:00	-10.8	92	1.3	1	312	northwest wind	961	1.5	0	82
2021-02-08 12:00:00	-10.7	92	1.5	1	239	southwest wind	960	1.3	0	82
2021-02-08 13:00:00	-10	92	1	1	200	south wind	960	1.8	0	82
2021-02-08 14:00:00	-8.2	92	0.7	1	340	north wind	958	2	0	82
2021-02-08 15:00:00	-6.8	76	1.6	2	128	southeast wind	957	2.2	0	82
2021-02-08 16:00:00	-5.7	70	1.2	1	245	southwest wind	957	2.4	0	82
2021-02-08 17:00:00	-6.2	71	1.3	1	223	southwest wind	956	2.4	0	82
2021-02-08 18:00:00	-6	73	1.4	1	217	southwest wind	956	2.6	0	23
2021-02-08 19:00:00	-6.6	76	1.5	1	277	west wind	956	2.6	0	23
2021-02-08 20:00:00	-6.8	79	0.8	1	291	west wind	956	2.6	0	23
2021-02-08 21:00:00	-7.2	81	1.3	1	228	southwest wind	955	2.4	0	82
2021-02-08 22:00:00	-7.3	83	1	1	267	west wind	955	2.1	0	82
2021-02-08 23:00:00	-8.6	85	0.9	1	251	west wind	954	1.8	0	82
2021-02-09 00:00:00	-9.8	87	0.9	1	240	southwest wind	954	1.8	0	82
2021-02-09 01:00:00	-10.3	89	0.6	1	29	northeast wind	954	2.5	0	82
2021-02-09 02:00:00	-10.8	90	1	1	302	northwest wind	954	4.1	0	82
2021-02-09 03:00:00	-10	87	1.3	1	183	south wind	953	6.7	0	82
2021-02-09 04:00:00	-8.4	86	1.6	2	288	west wind	953	7.9	0	82
2021-02-09 05:00:00	-7.7	86	0.9	1	260	west wind	953	5.1	0	82
2021-02-09 06:00:00	-7.2	85	1.5	1	328	northwest wind	954	7.2	0	82
2021-02-09 07:00:00	-6.6	86	1.6	2	250	west wind	954	7.7	0	82
2021-02-09 08:00:00	-5.1	82	2.3	2	253	west wind	954	8	0	82
2021-02-09 09:00:00	-4	79	2.9	2	255	west wind	954	8.3	0	90
2021-02-09 10:00:00	-3.7	77	1.7	2	232	southwest wind	954	6.9	0	90

2021-02-09 11:00:00	-3.1	74	0.9	1	313	northwest wind	954	7.1	0	90
2021-02-09 12:00:00	-0.7	65	1.3	1	284	west wind	954	11.5	0	82
2021-02-09 13:00:00	1.8	55	1.1	1	74	east wind	954	17.2	0	82
2021-02-09 14:00:00	2.2	63	2.2	2	130	southeast wind	954	10.8	0	82
2021-02-09 15:00:00	1.5	71	3.2	2	132	southeast wind	954	11.7	0	82
2021-02-09 16:00:00	1.9	69	1.4	1	197	south wind	954	12.7	0	10
2021-02-09 17:00:00	2.1	71	1.8	2	170	south wind	954	12.3	0	10
2021-02-09 18:00:00	0.9	76	2	2	120	southeast wind	954	10.2	0	90
2021-02-09 19:00:00	0	78	1.8	2	124	southeast wind	955	10.1	0	90
2021-02-09 20:00:00	-0.8	82	1.5	1	146	southeast wind	955	11.5	0	90
2021-02-09 21:00:00	-1.4	86	2.2	2	122	southeast wind	955	15.8	0	23
2021-02-09 22:00:00	-2.5	87	2.4	2	173	south wind	956	10.9	0	23
2021-02-09 23:00:00	-2.5	86	2.5	2	165	south wind	956	26.8	0	23
2021-02-10 00:00:00	-3.4	86	2.8	2	94	east wind	956	16.6	0	23
2021-02-10 01:00:00	-4.3	87	3	2	97	east wind	957	30	0	23
2021-02-10 02:00:00	-4.9	85	2.2	2	132	southeast wind	957	19	0	23
2021-02-10 03:00:00	-4.8	86	1.8	2	111	east wind	957	16.7	0	23
2021-02-10 04:00:00	-5.2	89	1.5	1	133	southeast wind	958	12.6	0	23
2021-02-10 05:00:00	-5.2	89	2.7	2	148	southeast wind	958	18.3	0	23
2021-02-10 06:00:00	-5.5	85	2.1	2	149	southeast wind	959	22.8	0	23
2021-02-10 07:00:00	-5.4	86	1.7	2	158	south wind	960	8.6	0	23
2021-02-10 08:00:00	-5.4	78	3.4	3	134	southeast wind	962	20.2	0	23
2021-02-10 09:00:00	-6.1	80	3	2	166	south wind	963	9.7	0	82
2021-02-10 10:00:00	-6.4	84	2.6	2	141	southeast wind	964	26.1	0	82
2021-02-10 11:00:00	-5.9	74	2.8	2	122	southeast wind	964	30	0	82
2021-02-10 12:00:00	-5.2	64	3.1	2	148	southeast wind	964	30	0	23
2021-02-10 13:00:00	-4.4	64	3.7	3	155	southeast wind	964	30	0	23
2021-02-10 14:00:00	-3.6	62	3	2	152	southeast wind	964	30	0	23
2021-02-10 15:00:00	-3.5	70	2.4	2	116	southeast wind	963	27.6	0	23
2021-02-10 16:00:00	-4.6	78	1.7	2	75	east wind	963	22.5	0	23
2021-02-10 17:00:00	-3.6	69	0.5	1	195	south wind	963	24.2	0	23
2021-02-10 18:00:00	-4.3	77	1.5	1	218	southwest wind	964	26	0	23
2021-02-10 19:00:00	-4.7	79	1.1	1	256	west wind	964	20.3	0	23
2021-02-10 20:00:00	-4.7	79	1.1	1	259	west wind	964	20.8	0	23
2021-02-10 21:00:00	-5	79	1.3	1	279	west wind	964	19.2	0	82
2021-02-10 22:00:00	-5.5	82	0.7	1	293	northwest wind	964	20.3	0	82
2021-02-10 23:00:00	-7.4	87	1.6	2	241	southwest wind	963	11.5	0	82
2021-02-11 00:00:00	-8.6	89	2.1	2	256	west wind	963	13.3	0	82

2021-02-11 01:00:00	-9.5	87	2.1	2	265	west wind	964	15.3	0	82
2021-02-11 02:00:00	-10.5	84	1.5	1	267	west wind	964	17.2	0	82
2021-02-11 03:00:00	-11.2	80	2	2	262	west wind	964	20.5	0	82
2021-02-11 04:00:00	-11.8	82	2.2	2	264	west wind	964	20.4	0	82
2021-02-11 05:00:00	-12.4	81	1.5	1	268	west wind	964	21.7	0	82
2021-02-11 06:00:00	-12.7	82	1.4	1	267	west wind	963	21	0	82
2021-02-11 07:00:00	-12.9	82	2	2	261	west wind	963	18.8	0	82
2021-02-11 08:00:00	-13	82	1.9	2	263	west wind	963	15.8	0	82
2021-02-11 09:00:00	-13.1	83	1.3	1	285	west wind	963	8.1	0	23
2021-02-11 10:00:00	-12.2	80	1.6	2	267	west wind	964	17.9	0	23
2021-02-11 11:00:00	-10.2	75	2	2	255	west wind	964	17.4	0	23
2021-02-11 12:00:00	-8	70	1.3	1	261	west wind	964	13.6	0	23
2021-02-11 13:00:00	-5.4	74	1.5	1	262	west wind	964	11.2	0	23
2021-02-11 14:00:00	-3.6	71	1.5	1	252	west wind	963	13.1	0	23
2021-02-11 15:00:00	-2.9	67	1.5	1	323	northwest wind	963	13.8	0	10
2021-02-11 16:00:00	-3	69	1.7	2	53	northeast wind	962	15.8	0	10
2021-02-11 17:00:00	-3.1	73	2.4	2	46	northeast wind	962	12.1	0	10
2021-02-11 18:00:00	-4.3	79	1.7	2	83	east wind	962	9.4	0	23
2021-02-11 19:00:00	-5.2	85	1.2	1	80	east wind	962	7.9	0	23
2021-02-11 20:00:00	-6.1	87	0.8	1	32	northeast wind	963	6.6	0	23
2021-02-11 21:00:00	-7.4	90	1	1	259	west wind	964	3.9	0	10
2021-02-11 22:00:00	-8.8	91	0.9	1	268	west wind	964	8	0	10
2021-02-11 23:00:00	-10	89	1.2	1	300	northwest wind	964	4.1	0	10
2021-02-12 00:00:00	-11.3	90	1.2	1	257	west wind	965	4.2	0	10
2021-02-12 01:00:00	-10.5	88	0.9	1	300	northwest wind	965	5.9	0	10
2021-02-12 02:00:00	-9.3	92	1.8	2	303	northwest wind	965	3.5	0	10
2021-02-12 03:00:00	-8.3	94	1.1	1	251	west wind	965	3.8	0	10
2021-02-12 04:00:00	-8.2	94	1.9	2	239	southwest wind	965	3.8	0	10
2021-02-12 05:00:00	-9.1	94	1.6	2	295	northwest wind	964	0.6	0	10
2021-02-12 06:00:00	-10.6	93	1.4	1	309	northwest wind	964	0.4	0	10
2021-02-12 07:00:00	-10.8	93	1	1	262	west wind	964	0.5	0	10
2021-02-12 08:00:00	-11	92	0.8	1	229	southwest wind	964	0.6	0	10
2021-02-12 09:00:00	-11.7	92	1.7	2	254	west wind	964	0.6	0	82
2021-02-12 10:00:00	-11	92	1.1	1	265	west wind	965	2	0	82
2021-02-12 11:00:00	-10.7	92	1.2	1	213	southwest wind	964	4.5	0	82
2021-02-12 12:00:00	-10.1	93	1.6	2	244	southwest wind	964	3.1	0	82
2021-02-12 13:00:00	-8.1	94	0.7	1	122	southeast wind	964	3.7	0	82
2021-02-12 14:00:00	-6.8	94	1.2	1	204	southwest wind	964	4.3	0	82

2021-02-12 15:00:00	-5.6	78	0.7	1	293	northwest wind	963	5.9	0	23
2021-02-12 16:00:00	-5.5	77	2.2	2	10	north wind	963	5.8	0	23
2021-02-12 17:00:00	-5.9	80	2	2	29	northeast wind	963	5.8	0	23
2021-02-12 18:00:00	-6.4	82	1.3	1	21	north wind	963	5.7	0	23
2021-02-12 19:00:00	-6.6	83	1.3	1	6	north wind	964	5.7	0	23
2021-02-12 20:00:00	-6.9	85	1	1	331	northwest wind	964	6.3	0	23
2021-02-12 21:00:00	-7.1	88	1.6	2	296	northwest wind	964	5.1	0	23
2021-02-12 22:00:00	-7.3	88	1	1	301	northwest wind	964	4.2	0	23
2021-02-12 23:00:00	-7.4	89	0.8	1	238	southwest wind	963	3.8	0	23
2021-02-13 00:00:00	-7.9	90	1.4	1	163	south wind	963	3.5	0	23
2021-02-13 01:00:00	-7.9	92	1.2	1	176	south wind	963	3	0	23
2021-02-13 02:00:00	-8.4	94	1.8	2	176	south wind	963	3.2	0	23
2021-02-13 03:00:00	-8.6	94	1.3	1	256	west wind	963	2.3	0	23
2021-02-13 04:00:00	-8.6	95	0.7	1	278	west wind	963	2.1	0	23
2021-02-13 05:00:00	-8.7	95	1.4	1	265	west wind	963	1.6	0	23
2021-02-13 06:00:00	-8.7	95	1.5	1	343	north wind	963	1.9	0	23
2021-02-13 07:00:00	-9	94	1.5	1	304	northwest wind	964	0.9	0	23
2021-02-13 08:00:00	-9.1	95	1.1	1	293	northwest wind	964	2.6	0	23
2021-02-13 09:00:00	-8.6	94	1	1	252	west wind	964	2.7	0	23
2021-02-13 10:00:00	-8.5	94	1.6	2	255	west wind	965	2.6	0	23
2021-02-13 11:00:00	-8.2	94	1.5	1	272	west wind	965	2.8	0	23
2021-02-13 12:00:00	-7.4	94	1.1	1	299	northwest wind	965	2.9	0	90
2021-02-13 13:00:00	-6.4	90	0.7	1	27	northeast wind	965	4.3	0	90
2021-02-13 14:00:00	-5.9	87	1.7	2	74	east wind	965	3.6	0	90
2021-02-13 15:00:00	-5.9	87	1.8	2	64	northeast wind	965	4.6	0	90
2021-02-13 16:00:00	-5.6	85	1.7	2	100	east wind	965	4	0	90
2021-02-13 17:00:00	-5.4	84	0.9	1	60	northeast wind	965	3.9	0	90
2021-02-13 18:00:00	-5.4	83	1.1	1	57	northeast wind	965	5.2	0	90
2021-02-13 19:00:00	-5.5	83	1.3	1	33	northeast wind	966	6.3	0	90
2021-02-13 20:00:00	-5.7	83	0.8	1	29	northeast wind	966	6.3	0	90
2021-02-13 21:00:00	-5.7	84	1.1	1	168	south wind	967	5.4	0	23
2021-02-13 22:00:00	-5.8	84	1.1	1	51	northeast wind	968	6.1	0	23
2021-02-13 23:00:00	-5.8	83	1.4	1	47	northeast wind	968	7.9	0	23
2021-02-14 00:00:00	-6	84	1	1	13	north wind	968	9.2	0	23
2021-02-14 01:00:00	-6.1	84	1	1	281	west wind	968	9.2	0	23
2021-02-14 02:00:00	-6.1	84	1.2	1	305	northwest wind	968	9.5	0	23
2021-02-14 03:00:00	-6	83	1.3	1	286	west wind	968	9.6	0	23
2021-02-14 04:00:00	-6.1	84	0.9	1	237	southwest wind	968	9.1	0	23

2021-02-14 05:00:00	-6.3	86	0.2	0	188	south wind	968	8.3	0	23
2021-02-14 06:00:00	-6.3	87	1.7	2	64	northeast wind	968	5.6	0	23
2021-02-14 07:00:00	-6.6	94	1.1	1	42	northeast wind	969	3	0	23
2021-02-14 08:00:00	-6.7	95	0.9	1	6	north wind	969	2	0	23
2021-02-14 09:00:00	-6.9	96	1	1	6	north wind	970	2.2	0	23
2021-02-14 10:00:00	-6.8	97	1	1	355	north wind	970	2.6	0	23
2021-02-14 11:00:00	-6.5	96	1.2	1	45	northeast wind	970	2.9	0	23
2021-02-14 12:00:00	-6.3	96	1.4	1	115	southeast wind	970	3.5	0	90
2021-02-14 13:00:00	-6	95	1.1	1	88	east wind	970	3.7	0	90
2021-02-14 14:00:00	-5.4	91	1.3	1	75	east wind	970	3.2	0	90
2021-02-14 15:00:00	-5.2	89	1.8	2	68	east wind	969	4.8	0	90
2021-02-14 16:00:00	-5.3	89	2.3	2	69	east wind	969	5	0	90
2021-02-14 17:00:00	-5.4	91	1.6	2	82	east wind	969	3.9	0	90
2021-02-14 18:00:00	-5.7	93	1.6	2	63	northeast wind	970	3.9	0	90
2021-02-14 19:00:00	-5.9	94	1.5	1	124	southeast wind	970	3.2	0	90
2021-02-14 20:00:00	-6	95	1.2	1	54	northeast wind	970	2.5	0	90
2021-02-14 21:00:00	-6.4	96	1.2	1	54	northeast wind	970	2.6	0	23
2021-02-14 22:00:00	-6.4	97	1.1	1	126	southeast wind	970	3	0	23
2021-02-14 23:00:00	-6.5	97	1	1	53	northeast wind	970	2.7	0	23
2021-02-15 00:00:00	-6.7	97	1.2	1	37	northeast wind	970	2.6	0	23
2021-02-15 01:00:00	-6.8	97	0.9	1	30	northeast wind	970	2.8	0	23
2021-02-15 02:00:00	-6.8	97	0.5	1	19	north wind	969	2.9	0	23
2021-02-15 03:00:00	-6.8	96	0.9	1	315	northwest wind	969	2.4	0	23
2021-02-15 04:00:00	-6.8	96	0.9	1	17	north wind	969	2.6	0	23
2021-02-15 05:00:00	-6.7	96	0.3	1	311	northwest wind	968	3.1	0	23
2021-02-15 06:00:00	-6.7	96	0.7	1	338	north wind	968	2.8	0	23
2021-02-15 07:00:00	-6.7	96	0.5	1	51	northeast wind	968	2.6	0	23
2021-02-15 08:00:00	-6.6	96	0.5	1	52	northeast wind	969	2.9	0	23
2021-02-15 09:00:00	-6.7	96	0.5	1	57	northeast wind	969	2.9	0	10
2021-02-15 10:00:00	-6.6	96	0.7	1	121	southeast wind	969	2.7	0	10
2021-02-15 11:00:00	-6.3	96	0.7	1	26	northeast wind	969	3.1	0	10
2021-02-15 12:00:00	-6	94	1.1	1	127	southeast wind	969	3.6	0	23
2021-02-15 13:00:00	-5.6	88	0.9	1	136	southeast wind	969	2.7	0	23
2021-02-15 14:00:00	-4.8	85	0.9	1	135	southeast wind	968	1.6	0	23
2021-02-15 15:00:00	-4.4	80	1.2	1	182	south wind	968	3.8	0	82
2021-02-15 16:00:00	-4.8	85	1.7	2	93	east wind	967	4.1	0	82
2021-02-15 17:00:00	-4.7	84	1.7	2	123	southeast wind	967	6.5	0	82
2021-02-15 18:00:00	-5	84	2	2	149	southeast wind	967	7.6	0	82

2021-02-15 19:00:00	-5.2	86	1.6	2	137	southeast wind	967	5.3	0	82
2021-02-15 20:00:00	-5.6	90	1	1	98	east wind	968	6.9	0	82
2021-02-15 21:00:00	-5.8	92	1.6	2	74	east wind	968	4	0	23
2021-02-15 22:00:00	-6.1	93	0.7	1	6	north wind	968	4.1	0	23
2021-02-15 23:00:00	-6.1	94	1.3	1	68	east wind	968	3.9	0	23
2021-02-16 00:00:00	-6.3	94	1	1	327	northwest wind	968	3.6	0	23
2021-02-16 01:00:00	-6.5	95	1	1	332	northwest wind	967	3.5	0	23
2021-02-16 02:00:00	-6.6	96	1	1	61	northeast wind	967	3.2	0	23
2021-02-16 03:00:00	-6.8	96	0.7	1	334	northwest wind	967	5.6	0	23
2021-02-16 04:00:00	-6.7	93	0.6	1	16	north wind	967	4.9	0	23
2021-02-16 05:00:00	-6.7	93	0.8	1	18	north wind	966	3.9	0	23
2021-02-16 06:00:00	-6.8	94	0.9	1	38	northeast wind	966	3.8	0	23
2021-02-16 07:00:00	-6.9	94	1	1	44	northeast wind	966	3.4	0	23
2021-02-16 08:00:00	-7	94	1.3	1	63	northeast wind	966	3.4	0	23
2021-02-16 09:00:00	-7	95	1.6	2	36	northeast wind	967	3.6	0	23
2021-02-16 10:00:00	-7.1	94	1.3	1	54	northeast wind	967	3.7	0	23
2021-02-16 11:00:00	-7	90	1.2	1	283	west wind	967	2.1	0	23
2021-02-16 12:00:00	-6.7	88	1.5	1	296	northwest wind	967	2.4	0	23
2021-02-16 13:00:00	-6	82	1.8	2	310	northwest wind	967	5.7	0	23
2021-02-16 14:00:00	-5.3	77	1.6	2	274	west wind	966	7.1	0	23
2021-02-16 15:00:00	-5.5	77	1.5	1	290	west wind	965	8	0	90
2021-02-16 16:00:00	-5.3	76	1.4	1	315	northwest wind	965	7.1	0	90
2021-02-16 17:00:00	-5.4	77	1	1	318	northwest wind	965	5.5	0	90
2021-02-16 18:00:00	-5.3	78	1.1	1	297	northwest wind	965	6.6	0	82
2021-02-16 19:00:00	-5.8	79	1.3	1	331	northwest wind	966	10	0	82
2021-02-16 20:00:00	-6	79	0.7	1	54	northeast wind	966	9.1	0	82
2021-02-16 21:00:00	-6.1	86	1	1	20	north wind	966	5.6	0	90
2021-02-16 22:00:00	-6.3	91	1	1	4	north wind	966	5.2	0	90
2021-02-16 23:00:00	-6.6	93	0.7	1	332	northwest wind	966	5.4	0	90
2021-02-17 00:00:00	-7.4	92	0.4	1	29	northeast wind	966	3.9	0	90
2021-02-17 01:00:00	-8.9	94	0.8	1	331	northwest wind	966	1.1	0	90
2021-02-17 02:00:00	-9.2	95	0.9	1	277	west wind	966	3.9	0	90
2021-02-17 03:00:00	-9.3	94	0.9	1	248	west wind	965	4.7	0	90
2021-02-17 04:00:00	-9.5	94	1.2	1	251	west wind	965	1.2	0	90
2021-02-17 05:00:00	-9.5	94	1.1	1	234	southwest wind	965	1.7	0	90
2021-02-17 06:00:00	-9.2	94	1.2	1	246	southwest wind	965	2	0	90
2021-02-17 07:00:00	-9.1	94	0.9	1	250	west wind	965	0.7	0	90
2021-02-17 08:00:00	-9.1	94	1	1	294	northwest wind	966	0.8	0	90

2021-02-17 09:00:00	-8.9	94	1	1	330	northwest wind	966	1.5	0	23
2021-02-17 10:00:00	-9.6	94	1.1	1	315	northwest wind	967	2.1	0	23
2021-02-17 11:00:00	-8.9	94	1.6	2	338	north wind	967	2.5	0	23
2021-02-17 12:00:00	-8.6	94	1.5	1	313	northwest wind	967	3.3	0	23
2021-02-17 13:00:00	-7.9	94	0.9	1	259	west wind	967	3.1	0	23
2021-02-17 14:00:00	-7.2	94	0.9	1	289	west wind	966	4.2	0	23
2021-02-17 15:00:00	-6	77	0.5	1	318	northwest wind	965	4.2	0	82
2021-02-17 16:00:00	-6	78	1	1	13	north wind	965	5	0	82
2021-02-17 17:00:00	-6	80	1.3	1	51	northeast wind	965	4.5	0	82
2021-02-17 18:00:00	-6	79	0.8	1	336	northwest wind	965	5.6	0	82
2021-02-17 19:00:00	-6.5	81	1.1	1	334	northwest wind	965	6.3	0	82
2021-02-17 20:00:00	-7	84	0.6	1	326	northwest wind	966	6	0	82
2021-02-17 21:00:00	-7.2	89	1.5	1	37	northeast wind	966	6	0	23
2021-02-17 22:00:00	-7.5	90	1.1	1	41	northeast wind	966	4.9	0	23
2021-02-17 23:00:00	-7.6	91	0.9	1	311	northwest wind	966	4	0	23
2021-02-18 00:00:00	-7.6	92	1.3	1	212	southwest wind	965	4.1	0	23
2021-02-18 01:00:00	-8	92	0.9	1	253	west wind	965	3.8	0	23
2021-02-18 02:00:00	-8.1	94	1.1	1	225	southwest wind	965	3.4	0	23
2021-02-18 03:00:00	-8.5	94	1.6	2	262	west wind	964	2.6	0	23
2021-02-18 04:00:00	-8.8	95	1.3	1	249	west wind	964	2	0	23
2021-02-18 05:00:00	-9.5	95	1.4	1	181	south wind	964	0.6	0	23
2021-02-18 06:00:00	-10.2	94	1.3	1	277	west wind	965	0.9	0	23
2021-02-18 07:00:00	-10.7	93	2	2	309	northwest wind	965	0.7	0	23
2021-02-18 08:00:00	-11.5	92	1.4	1	294	northwest wind	965	0.6	0	23
2021-02-18 09:00:00	-11.7	92	1.6	2	296	northwest wind	965	1.9	0	23
2021-02-18 10:00:00	-11.3	92	1.4	1	256	west wind	966	0.6	0	23
2021-02-18 11:00:00	-10.6	93	1.4	1	306	northwest wind	966	1.3	0	23
2021-02-18 12:00:00	-10.1	93	1.1	1	292	west wind	965	1.8	0	23
2021-02-18 13:00:00	-8.9	94	1	1	4	north wind	965	2.5	0	23
2021-02-18 14:00:00	-8.5	94	1.7	2	347	north wind	965	4.2	0	23
2021-02-18 15:00:00	-7.6	94	1	1	52	northeast wind	964	3.8	0	82
2021-02-18 16:00:00	-6.8	94	1.2	1	302	northwest wind	963	4.4	0	82
2021-02-18 17:00:00	-6.5	84	1.8	2	36	northeast wind	963	6.4	0	82
2021-02-18 18:00:00	-7.2	83	1.4	1	337	northwest wind	962	5.9	0	82
2021-02-18 19:00:00	-7.5	84	1.2	1	339	north wind	962	6.2	0	82
2021-02-18 20:00:00	-8.6	87	2.1	2	42	northeast wind	963	5.1	0	82
2021-02-18 21:00:00	-9	90	2	2	33	northeast wind	963	4.2	0	23
2021-02-18 22:00:00	-9.1	91	0.9	1	0	north wind	962	3.6	0	23

2021-02-18 23:00:00	-8.8	93	2	2	256	west wind	962	2.5	0	23
2021-02-19 00:00:00	-9.5	95	2	2	248	west wind	961	2.3	0	23
2021-02-19 01:00:00	-9.9	95	0.9	1	217	southwest wind	960	2.4	0	23
2021-02-19 02:00:00	-10.5	94	1	1	237	southwest wind	959	1.7	0	23
2021-02-19 03:00:00	-10.5	94	1	1	110	east wind	958	1.9	0	23
2021-02-19 04:00:00	-10.2	93	1.3	1	316	northwest wind	959	1	0	23
2021-02-19 05:00:00	-9.9	94	0.7	1	144	southeast wind	958	0.9	0	23
2021-02-19 06:00:00	-10.7	93	1.4	1	268	west wind	957	0.7	0	23
2021-02-19 07:00:00	-11	93	1.8	2	264	west wind	956	0.9	0	23
2021-02-19 08:00:00	-11.5	92	0.5	1	160	south wind	955	0.3	0	23
2021-02-19 09:00:00	-11.6	92	1.4	1	327	northwest wind	955	1.3	0	82
2021-02-19 10:00:00	-11.4	92	1.2	1	302	northwest wind	955	0.7	0	82
2021-02-19 11:00:00	-10.2	93	1.2	1	259	west wind	955	1.3	0	82
2021-02-19 12:00:00	-9.3	94	1.2	1	261	west wind	954	3.5	0	90
2021-02-19 13:00:00	-7.7	94	2.1	2	281	west wind	954	6.5	0	90
2021-02-19 14:00:00	-6	94	1	1	126	southeast wind	953	7.6	0	90
2021-02-19 15:00:00	-4.3	75	1.7	2	113	southeast wind	953	4.9	0	90
2021-02-19 16:00:00	-4	72	1.4	1	305	northwest wind	952	7.4	0	90
2021-02-19 17:00:00	-3.3	72	1.9	2	144	southeast wind	951	4.9	0	90
2021-02-19 18:00:00	-4.8	80	1.5	1	80	east wind	952	4.7	0	90
2021-02-19 19:00:00	-5.3	80	1.4	1	319	northwest wind	951	5.1	0	90
2021-02-19 20:00:00	-5.4	85	2	2	87	east wind	952	4	0	90
2021-02-19 21:00:00	-5.5	86	1.2	1	83	east wind	952	3.7	0	90
2021-02-19 22:00:00	-5.8	89	1	1	256	west wind	952	3.7	0	90
2021-02-19 23:00:00	-5.3	85	1.3	1	246	southwest wind	952	4.4	0	90
2021-02-20 00:00:00	-5	82	1.2	1	187	south wind	953	5.9	0	90
2021-02-20 01:00:00	-4.8	82	1.2	1	300	northwest wind	952	5.4	0	90
2021-02-20 02:00:00	-4.7	82	1.3	1	290	west wind	952	4.8	0	90
2021-02-20 03:00:00	-5.5	86	0.9	1	272	west wind	952	5.9	0	90
2021-02-20 04:00:00	-5.7	83	2	2	2	north wind	952	12.6	0	90
2021-02-20 05:00:00	-6.7	83	1.6	2	5	north wind	953	8.1	0	90
2021-02-20 06:00:00	-6.9	86	1.6	2	44	northeast wind	954	3.8	0	90
2021-02-20 07:00:00	-8	91	0.9	1	23	northeast wind	955	3.3	0	90
2021-02-20 08:00:00	-8.2	91	1	1	238	southwest wind	955	3.5	0	90
2021-02-20 09:00:00	-7.8	90	1.8	2	237	southwest wind	956	4.4	0	23
2021-02-20 10:00:00	-6	81	1.5	1	318	northwest wind	957	7.2	0	23
2021-02-20 11:00:00	-2.2	78	1.4	1	300	northwest wind	958	8.9	0	23
2021-02-20 12:00:00	1.1	79	1.7	2	247	southwest wind	958	11.2	0	23

2021-02-20 13:00:00	1.6	73	2.3	2	235	southwest wind	959	11.4	0	23
2021-02-20 14:00:00	3.1	69	1.2	1	291	west wind	959	13.7	0	23
2021-02-20 15:00:00	3.8	69	2.8	2	122	southeast wind	959	16.6	0	90
2021-02-20 16:00:00	3.5	69	1.7	2	118	southeast wind	959	16.3	0	90
2021-02-20 17:00:00	2.9	74	2.2	2	164	south wind	960	15.2	0	90
2021-02-20 18:00:00	2.8	75	0.9	1	85	east wind	962	14.4	0	82
2021-02-20 19:00:00	2.5	78	1.6	2	132	southeast wind	962	25.5	0	82
2021-02-20 20:00:00	1.2	84	3.1	2	122	southeast wind	964	3.9	0	82
2021-02-20 21:00:00	0.5	90	2.4	2	129	southeast wind	964	12.6	0	23
2021-02-20 22:00:00	0.2	90	1.1	1	160	south wind	965	12.6	0	23
2021-02-20 23:00:00	-0.1	92	3.2	2	146	southeast wind	965	8.8	0	23
2021-02-21 00:00:00	-0.9	95	1.1	1	149	southeast wind	965	3	0	23
2021-02-21 01:00:00	-0.9	97	0.9	1	257	west wind	964	9.2	0	23
2021-02-21 02:00:00	-0.7	95	0.9	1	139	southeast wind	964	7.5	0	23
2021-02-21 03:00:00	-0.6	94	0.8	1	219	southwest wind	964	4.7	0	23
2021-02-21 04:00:00	-0.8	95	0.7	1	103	east wind	964	5	0	23
2021-02-21 05:00:00	-0.8	95	0.6	1	25	northeast wind	964	5	0	23
2021-02-21 06:00:00	-1	96	0.9	1	126	southeast wind	964	5.2	0	23
2021-02-21 07:00:00	-1.1	96	0.9	1	109	east wind	964	5.5	0	23
2021-02-21 08:00:00	-1.2	96	0.7	1	25	northeast wind	964	4.4	0	23
2021-02-21 09:00:00	-1.2	96	1.7	2	96	east wind	964	1.4	0	10
2021-02-21 10:00:00	-2.3	98	1.7	2	66	northeast wind	964	0.3	0	10
2021-02-21 11:00:00	-2.7	99	1.8	2	73	east wind	964	0.1	0	10
2021-02-21 12:00:00	-2.9	100	2	2	55	northeast wind	964	0.1	0	10
2021-02-21 13:00:00	-2.6	100	1.3	1	87	east wind	965	0.2	0	10
2021-02-21 14:00:00	-2.4	100	1.2	1	33	northeast wind	964	0.3	0	10
2021-02-21 15:00:00	-1.9	100	0.9	1	160	south wind	963	1.2	0	90
2021-02-21 16:00:00	-1.6	100	1	1	310	northwest wind	964	1.2	0	90
2021-02-21 17:00:00	-1.4	100	0.6	1	276	west wind	964	1.1	0	90
2021-02-21 18:00:00	-1	99	0.8	1	181	south wind	964	2	0	90
2021-02-21 19:00:00	-1.1	98	1.2	1	68	east wind	964	1.1	0	90
2021-02-21 20:00:00	-1.8	98	0.9	1	81	east wind	965	2.1	0	90
2021-02-21 21:00:00	-2	98	0.3	1	40	northeast wind	966	1.5	0	90
2021-02-21 22:00:00	-2.5	98	0.9	1	161	south wind	966	0.9	0	90
2021-02-21 23:00:00	-2.5	99	1.2	1	253	west wind	966	0.2	0	90
2021-02-22 00:00:00	-3	99	1.3	1	259	west wind	965	0.2	0	90
2021-02-22 01:00:00	-3.2	100	1.4	1	296	northwest wind	965	0.2	0	90
2021-02-22 02:00:00	-4.1	100	0.9	1	298	northwest wind	964	0.2	0	90

2021-02-22 03:00:00	-4.1	100	0.8	1	303	northwest wind	964	0.3	0	90
2021-02-22 04:00:00	-4.1	99	1.1	1	309	northwest wind	964	0.9	0	90
2021-02-22 05:00:00	-4.6	99	0.7	1	250	west wind	963	0.1	0	90
2021-02-22 06:00:00	-4.9	99	0.9	1	343	north wind	963	0.2	0	90
2021-02-22 07:00:00	-5.4	98	0.9	1	306	northwest wind	963	4.4	0	90
2021-02-22 08:00:00	-4.8	94	1	1	313	northwest wind	963	4.1	0	90
2021-02-22 09:00:00	-4	99	0.8	1	251	west wind	963	4.1	0	82
2021-02-22 10:00:00	-3.4	99	1	1	257	west wind	964	4.6	0	82
2021-02-22 11:00:00	-2.5	99	1.1	1	218	southwest wind	964	4.7	0	82
2021-02-22 12:00:00	-1.5	98	1.1	1	246	southwest wind	964	4.1	0	90
2021-02-22 13:00:00	-0.5	90	1	1	260	west wind	964	4.8	0	90
2021-02-22 14:00:00	0.9	84	1	1	2	north wind	964	4.2	0	90
2021-02-22 15:00:00	-0.5	91	1.9	2	89	east wind	963	3	0	90
2021-02-22 16:00:00	-0.7	93	1.4	1	84	east wind	963	2.7	0	90
2021-02-22 17:00:00	-1.1	94	0	0	96	east wind	963	2.4	0	90
2021-02-22 18:00:00	-0.8	93	0	0	200	south wind	963	2.9	0	23
2021-02-22 19:00:00	-0.9	92	0	0	247	southwest wind	963	2.3	0	23
2021-02-22 20:00:00	-1.3	95	0	0	255	west wind	964	2.1	0	23
2021-02-22 21:00:00	-1.6	96	0.8	1	336	northwest wind	964	2.2	0	82
2021-02-22 22:00:00	-1.5	97	0.9	1	310	northwest wind	964	1.9	0	82
2021-02-22 23:00:00	-1.3	97	0.3	1	283	west wind	964	1.9	0	82
2021-02-23 00:00:00	-1.3	96	1.1	1	245	southwest wind	964	1.9	0	82
2021-02-23 01:00:00	-1.1	96	1	1	255	west wind	964	2.1	0	82
2021-02-23 02:00:00	-1	96	0.9	1	239	southwest wind	963	2.5	0	82
2021-02-23 03:00:00	-1	96	0.7	1	273	west wind	963	2.2	0	82
2021-02-23 04:00:00	-1.1	96	0.9	1	30	northeast wind	962	2.3	0	82
2021-02-23 05:00:00	-0.9	95	0.6	1	28	northeast wind	961	2.2	0	82
2021-02-23 06:00:00	-1.1	96	0.5	1	103	east wind	961	2.5	0	82
2021-02-23 07:00:00	-1.2	95	1.4	1	63	northeast wind	961	3.6	0	82
2021-02-23 08:00:00	-1.6	95	0.4	1	351	north wind	961	3.1	0	82
2021-02-23 09:00:00	-1.8	96	0.8	1	313	northwest wind	961	3.6	0	10
2021-02-23 10:00:00	-1.5	96	1.2	1	68	east wind	961	3.8	0	10
2021-02-23 11:00:00	-1.2	95	0.3	1	343	north wind	961	5.2	0	10
2021-02-23 12:00:00	-0.4	89	1.4	1	50	northeast wind	961	5.3	0	82
2021-02-23 13:00:00	-0.4	90	1.6	2	90	east wind	960	3.4	0	82
2021-02-23 14:00:00	-0.6	92	1.2	1	91	east wind	960	2.8	0	82
2021-02-23 15:00:00	-0.8	94	1.7	2	86	east wind	959	1.1	0	90
2021-02-23 16:00:00	-1.2	97	1.7	2	90	east wind	959	1.5	0	90

2021-02-23 17:00:00	-1	97	1.2	1	11	north wind	959	1	0	90
2021-02-23 18:00:00	-1	97	1.3	1	37	northeast wind	958	2.2	0	90
2021-02-23 19:00:00	-1	96	1.3	1	69	east wind	959	2.2	0	90
2021-02-23 20:00:00	-1.4	97	1.6	2	77	east wind	959	1.6	0	90
2021-02-23 21:00:00	-1.5	98	1.6	2	85	east wind	959	0.1	0	90
2021-02-23 22:00:00	-1.8	99	1.3	1	64	northeast wind	960	0.2	0	90
2021-02-23 23:00:00	-1.9	100	1.3	1	47	northeast wind	959	1.5	0	90
2021-02-24 00:00:00	-2	100	1.5	1	29	northeast wind	959	0.3	0	90
2021-02-24 01:00:00	-2.1	100	0.7	1	337	northwest wind	958	1.1	0	90
2021-02-24 02:00:00	-2.1	100	0.8	1	76	east wind	958	0.4	0	90
2021-02-24 03:00:00	-2	100	1.3	1	107	east wind	958	0.2	0	90
2021-02-24 04:00:00	-2.1	100	0.4	1	114	southeast wind	957	0.1	0	90
2021-02-24 05:00:00	-2.1	100	1.5	1	70	east wind	956	0.1	0	90
2021-02-24 06:00:00	-2.3	100	1.1	1	11	north wind	956	0.2	0	90
2021-02-24 07:00:00	-2.3	100	1.1	1	352	north wind	956	0.2	0	90
2021-02-24 08:00:00	-2.3	100	1.1	1	345	north wind	957	0.2	0	90
2021-02-24 09:00:00	-2.2	100	1.2	1	356	north wind	957	0.2	0	90
2021-02-24 10:00:00	-2.1	100	0.6	1	345	north wind	957	0.8	0	90
2021-02-24 11:00:00	-1.9	100	0.8	1	43	northeast wind	957	0.4	0	90
2021-02-24 12:00:00	-1.9	100	1.2	1	62	northeast wind	956	0.2	0	90
2021-02-24 13:00:00	-2	100	1.6	2	51	northeast wind	956	0.2	0	90
2021-02-24 14:00:00	-2	100	1.5	1	68	east wind	955	2	0	90
2021-02-24 15:00:00	-2	100	1.7	2	57	northeast wind	954	0.6	0	90
2021-02-24 16:00:00	-1.9	100	1.7	2	72	east wind	954	0.3	0	90
2021-02-24 17:00:00	-2	100	1.4	1	72	east wind	954	0.3	0	90
2021-02-24 18:00:00	-2	100	1.5	1	82	east wind	954	1.9	0	23
2021-02-24 19:00:00	-1.9	100	0.7	1	13	north wind	953	3.3	0	23
2021-02-24 20:00:00	-2	100	1.5	1	179	south wind	953	2.4	0	23
2021-02-24 21:00:00	-1.9	100	0.9	1	242	southwest wind	954	2.8	0	82
2021-02-24 22:00:00	-1.9	100	1.5	1	298	northwest wind	953	4.2	0	82
2021-02-24 23:00:00	-2	100	1	1	161	south wind	953	4	0	82
2021-02-25 00:00:00	-2.1	100	0.9	1	108	east wind	952	3	0	82
2021-02-25 01:00:00	-2.3	100	1	1	294	northwest wind	952	4.1	0	82
2021-02-25 02:00:00	-2.2	100	1.5	1	253	west wind	952	5.1	0	82
2021-02-25 03:00:00	-2.4	98	0.8	1	236	southwest wind	951	6.5	0	82
2021-02-25 04:00:00	-2.4	94	1.8	2	275	west wind	951	6.7	0	82
2021-02-25 05:00:00	-2.3	92	2.3	2	259	west wind	950	8.1	0	82
2021-02-25 06:00:00	-2	90	1.6	2	258	west wind	949	8.9	0	82

2021-02-25 07:00:00	-1.9	89	1.7	2	244	southwest wind	951	9.4	0	82
2021-02-25 08:00:00	-1.5	90	0.7	1	143	southeast wind	951	7.7	0	82
2021-02-25 09:00:00	-1.9	93	1.9	2	49	northeast wind	952	4.5	0	82
2021-02-25 10:00:00	-1.9	94	2.4	2	47	northeast wind	954	8.6	0	82
2021-02-25 11:00:00	-1.8	95	1.3	1	320	northwest wind	955	2.8	0	82
2021-02-25 12:00:00	-1.2	94	3.7	3	135	southeast wind	957	4.8	0	90
2021-02-25 13:00:00	-3.1	75	7	4	120	southeast wind	957	6.9	0	90
2021-02-25 14:00:00	-4.4	73	7.4	4	118	southeast wind	957	12.1	0	90
2021-02-25 15:00:00	-5.6	72	3.5	3	70	east wind	958	14	0	90
2021-02-25 16:00:00	-6	72	3.5	3	102	east wind	960	6.3	0	90
2021-02-25 17:00:00	-6.9	84	2.1	2	94	east wind	961	1.6	0	90
2021-02-25 18:00:00	-7	87	2.3	2	95	east wind	962	1.9	0	90
2021-02-25 19:00:00	-6.9	87	2.4	2	117	southeast wind	962	8.5	0	90
2021-02-25 20:00:00	-6.8	85	0.5	1	97	east wind	963	24.3	0	90
2021-02-25 21:00:00	-6.9	86	1.5	1	293	northwest wind	964	18.3	0	90
2021-02-25 22:00:00	-7.1	86	2	2	161	south wind	964	26.3	0	90
2021-02-25 23:00:00	-7.3	85	3.3	2	157	southeast wind	965	20.9	0	90
2021-02-26 00:00:00	-7.7	84	2	2	158	south wind	965	30	0	90
2021-02-26 01:00:00	-8	78	1.8	2	156	southeast wind	966	28.6	0	90
2021-02-26 02:00:00	-8.3	73	2.5	2	154	southeast wind	966	29.8	0	90
2021-02-26 03:00:00	-8.2	74	2.1	2	123	southeast wind	966	20.8	0	90
2021-02-26 04:00:00	-8.5	73	2	2	138	southeast wind	967	29.1	0	90
2021-02-26 05:00:00	-8.9	72	2.3	2	134	southeast wind	967	27.4	0	90
2021-02-26 06:00:00	-9.7	73	2	2	179	south wind	967	21.2	0	90
2021-02-26 07:00:00	-10	71	1.1	1	173	south wind	968	16.8	0	90
2021-02-26 08:00:00	-10.2	66	1.7	2	139	southeast wind	968	30	0	90
2021-02-26 09:00:00	-10.9	67	1.5	1	164	south wind	968	18.9	0	82
2021-02-26 10:00:00	-10	61	2.3	2	117	southeast wind	969	30	0	82
2021-02-26 11:00:00	-9.4	59	3.1	2	123	southeast wind	970	29	0	82
2021-02-26 12:00:00	-9.6	65	2.8	2	119	southeast wind	969	16	0	82
2021-02-26 13:00:00	-8.5	66	2.5	2	110	east wind	969	9.9	0	82
2021-02-26 14:00:00	-8.5	69	0.8	1	137	southeast wind	969	2.4	0	82
2021-02-26 15:00:00	-8	68	2.5	2	190	south wind	969	15.6	0	23
2021-02-26 16:00:00	-7.2	65	3	2	168	south wind	969	20.2	0	23
2021-02-26 17:00:00	-7	66	2.3	2	177	south wind	970	9.1	0	23
2021-02-26 18:00:00	-6.6	65	2.3	2	173	south wind	970	2.5	0	23
2021-02-26 19:00:00	-7.3	78	1.8	2	324	northwest wind	970	13.6	0	23
2021-02-26 20:00:00	-9.5	80	1	1	312	northwest wind	970	19.8	0	23

2021-02-26 21:00:00	-11.1	79	1.9	2	266	west wind	971	19.4	0	82
2021-02-26 22:00:00	-14	75	1.5	1	256	west wind	971	18.5	0	82
2021-02-26 23:00:00	-16	78	2.2	2	263	west wind	971	23.2	0	82
2021-02-27 00:00:00	-16	73	1.7	2	255	west wind	971	22.5	0	82
2021-02-27 01:00:00	-16.8	74	1.2	1	252	west wind	970	25	0	82
2021-02-27 02:00:00	-17.2	72	2.1	2	267	west wind	970	26.7	0	82
2021-02-27 03:00:00	-17.5	70	2.7	2	275	west wind	969	27.5	0	82
2021-02-27 04:00:00	-17.9	70	2.1	2	268	west wind	968	27.5	0	82
2021-02-27 05:00:00	-18.6	71	3.1	2	261	west wind	968	27.3	0	82
2021-02-27 06:00:00	-18.5	73	2.1	2	258	west wind	968	28.5	0	82
2021-02-27 07:00:00	-18.1	72	3	2	265	west wind	968	28.3	0	82
2021-02-27 08:00:00	-18	70	2.5	2	258	west wind	968	28.7	0	82
2021-02-27 09:00:00	-17.9	70	2.6	2	265	west wind	969	23.2	0	90
2021-02-27 10:00:00	-17.4	68	3.6	3	255	west wind	969	27.5	0	90
2021-02-27 11:00:00	-15.4	65	2.6	2	257	west wind	969	22.6	0	90
2021-02-27 12:00:00	-12.6	61	1.9	2	245	southwest wind	969	27	0	90
2021-02-27 13:00:00	-9.3	55	1	1	316	northwest wind	968	25.6	0	90
2021-02-27 14:00:00	-8.1	59	2	2	24	northeast wind	968	28.9	0	90
2021-02-27 15:00:00	-9.1	69	1.9	2	4	north wind	967	29.9	0	82
2021-02-27 16:00:00	-8.8	66	2	2	354	north wind	967	30	0	82
2021-02-27 17:00:00	-8.4	59	1.2	1	339	north wind	967	25.3	0	82
2021-02-27 18:00:00	-8.8	71	1.4	1	53	northeast wind	967	29.8	0	23
2021-02-27 19:00:00	-9.1	69	1.9	2	51	northeast wind	968	24.4	0	23
2021-02-27 20:00:00	-10.3	79	0.8	1	4	north wind	968	20.1	0	23
2021-02-27 21:00:00	-11.6	81	1.1	1	292	west wind	969	16.4	0	82
2021-02-27 22:00:00	-14.3	76	1.7	2	262	west wind	970	17.2	0	82
2021-02-27 23:00:00	-16.1	75	1.9	2	264	west wind	970	20.9	0	82
2021-02-28 00:00:00	-16.2	77	1.2	1	272	west wind	970	21.9	0	82
2021-02-28 01:00:00	-16.7	77	1.6	2	277	west wind	970	24.5	0	82
2021-02-28 02:00:00	-17.1	77	1.7	2	249	west wind	970	25.3	0	82
2021-02-28 03:00:00	-17	77	2.6	2	271	west wind	970	26.1	0	82
2021-02-28 04:00:00	-17.1	75	2.3	2	268	west wind	970	25.8	0	82
2021-02-28 05:00:00	-19	78	1.7	2	262	west wind	970	28.1	0	82
2021-02-28 06:00:00	-18.8	76	1.3	1	262	west wind	970	27.5	0	82
2021-02-28 07:00:00	-19	77	1.3	1	259	west wind	971	26	0	82
2021-02-28 08:00:00	-18.7	75	2.4	2	267	west wind	971		0	82
2021-02-28 09:00:00	-19.1	76	1.7	2	263	west wind	972		0	90
2021-02-28 10:00:00	-17.9	73	2.2	2	268	west wind	972		0	90

2021-02-28 11:00:00	-14.9	68	1.3	1	254	west wind	973	20.6	0	90
2021-02-28 12:00:00	-11.2	65	1	1	213	southwest wind	972	23.5	0	90
2021-02-28 13:00:00	-7.9	58	0.8	1	193	south wind	972	15.9	0	90
2021-02-28 14:00:00	-7.9	64	2.1	2	28	northeast wind	971	15.7	0	90
2021-02-28 15:00:00	-6.6	65	2.2	2	135	southeast wind	970	13.9	0	82
2021-02-28 16:00:00	-7.1	67	2	2	90	east wind	970	15.3	0	82
2021-02-28 17:00:00	-7.2	68	1.9	2	84	east wind	970	16.2	0	82
2021-02-28 18:00:00	-8	69	1.8	2	48	northeast wind	970	16.5	0	23
2021-02-28 19:00:00	-8.4	69	1.3	1	30	northeast wind	970	15	0	23
2021-02-28 20:00:00	-9.5	78	1	1	353	north wind	970	13.4	0	23
2021-02-28 21:00:00	-10.8	82	1.1	1	266	west wind	971	11	0	23
2021-02-28 22:00:00	-12.6	82	0.9	1	243	southwest wind	971	9	0	23
2021-02-28 23:00:00	-14.8	81	1.2	1	284	west wind	971	4.2	0	23
2021-03-01 00:00:00	-15.4	82	1.4	1	260	west wind	971	10.2	0	23
2021-03-01 01:00:00	-16	81	1.8	2	261	west wind	970	10.7	0	23
2021-03-01 02:00:00	-16.2	83	2.6	2	257	west wind	969	10.6	0	23
2021-03-01 03:00:00	-16.3	84	2.4	2	253	west wind	969	8.8	0	23
2021-03-01 04:00:00	-16.8	83	1.7	2	262	west wind	968	10.9	0	23
2021-03-01 05:00:00	-17.5	83	2.3	2	261	west wind	967	11.5	0	23
2021-03-01 06:00:00	-17.6	81	3	2	258	west wind	967	13	0	23
2021-03-01 07:00:00	-17.7	80	2.9	2	268	west wind	967	12.4	0	23
2021-03-01 08:00:00	-17.4	78	2.9	2	267	west wind	967	11.1	0	23
2021-03-01 09:00:00	-17.5	77	2.1	2	257	west wind	967	10.9	0	82
2021-03-01 10:00:00	-16.6	76	2.4	2	264	west wind	966	13.7	0	82
2021-03-01 11:00:00	-13.8	69	1.7	2	258	west wind	966	15.1	0	82
2021-03-01 12:00:00	-11.1	63	1.1	1	247	southwest wind	965	14.3	0	82
2021-03-01 13:00:00	-7.1	53	0.7	1	112	east wind	964	13	0	82
2021-03-01 14:00:00	-6.3	60	2	2	31	northeast wind	964	11	0	82
2021-03-01 15:00:00	-6.2	58	1.8	2	354	north wind	964	12.4	0	23
2021-03-01 16:00:00	-6.1	60	1.5	1	37	northeast wind	963	11.9	0	23
2021-03-01 17:00:00	-5.6	58	1.3	1	296	northwest wind	963	12.6	0	23
2021-03-01 18:00:00	-5.4	56	1.1	1	254	west wind	963	13.7	0	82
2021-03-01 19:00:00	-5.9	59	1.4	1	285	west wind	963	13.8	0	82
2021-03-01 20:00:00	-6.4	62	1.5	1	256	west wind	963	10.8	0	82
2021-03-01 21:00:00	-7.3	66	1.2	1	236	southwest wind	963	11.4	0	23
2021-03-01 22:00:00	-8.2	73	0.8	1	265	west wind	963	9.9	0	23
2021-03-01 23:00:00	-9.6	73	1.6	2	309	northwest wind	962	21.3	0	23
2021-03-02 00:00:00	-11.2	73	1.1	1	250	west wind	962	13.3	0	23

2021-03-02 01:00:00	-12.4	77	0.1	0	315	northwest wind	962	16.9	0	23
2021-03-02 02:00:00	-13.1	75	1.1	1	277	west wind	961	21	0	23
2021-03-02 03:00:00	-13.6	70	1.3	1	279	west wind	961	22	0	23
2021-03-02 04:00:00	-14.5	76	1.8	2	268	west wind	961	17.5	0	23
2021-03-02 05:00:00	-14.1	74	1.9	2	254	west wind	960	18.7	0	23
2021-03-02 06:00:00	-14.3	71	1.3	1	278	west wind	960	22.7	0	23
2021-03-02 07:00:00	-14.8	71	1.7	2	259	west wind	960	15.2	0	23
2021-03-02 08:00:00	-15	73	1.6	2	266	west wind	960	12.7	0	23
2021-03-02 09:00:00	-15	70	1.2	1	252	west wind	960	13.7	0	82
2021-03-02 10:00:00	-13.6	67	2.1	2	263	west wind	960	15.3	0	82
2021-03-02 11:00:00	-10.1	56	0.9	1	178	south wind	960	14.8	0	82
2021-03-02 12:00:00	-5.8	56	1.3	1	351	north wind	959	8.5	0	23
2021-03-02 13:00:00	-4.8	60	1.1	1	306	northwest wind	959	10.6	0	23
2021-03-02 14:00:00	-3.7	56	1.3	1	336	northwest wind	958	10.3	0	23
2021-03-02 15:00:00	-2.6	56	1.5	1	37	northeast wind	958	10.6	0	23
2021-03-02 16:00:00	-2.7	56	1.3	1	142	southeast wind	957	9.6	0	23
2021-03-02 17:00:00	-1.3	55	0.9	1	92	east wind	957	9.3	0	23
2021-03-02 18:00:00	-3.1	62	2.3	2	87	east wind	957	8.5	0	23
2021-03-02 19:00:00	-3.9	65	1.5	1	80	east wind	957	8.9	0	23
2021-03-02 20:00:00	-4.8	68	0.9	1	349	north wind	957	7.6	0	23
2021-03-02 21:00:00	-5.8	72	1	1	337	northwest wind	958	6.8	0	23
2021-03-02 22:00:00	-6.7	75	0.6	1	253	west wind	958	4.9	0	23
2021-03-02 23:00:00	-7.5	80	1.4	1	281	west wind	957	5.2	0	23
2021-03-03 00:00:00	-8.8	82	1.3	1	260	west wind	958	4.9	0	23
2021-03-03 01:00:00	-8.9	81	1.1	1	268	west wind	957	6.1	0	23
2021-03-03 02:00:00	-9.8	84	2.3	2	257	west wind	957	5.4	0	23
2021-03-03 03:00:00	-9.8	85	0.9	1	277	west wind	956	4.4	0	23
2021-03-03 04:00:00	-10	86	1	1	250	west wind	956	5.5	0	23
2021-03-03 05:00:00	-10.5	85	0.8	1	268	west wind	955	4.6	0	23
2021-03-03 06:00:00	-10.6	87	1.7	2	292	west wind	955	6.6	0	23
2021-03-03 07:00:00	-11.4	86	1.3	1	307	northwest wind	955	5.9	0	23
2021-03-03 08:00:00	-11.4	85	0.5	1	72	east wind	956	4	0	23
2021-03-03 09:00:00	-10.7	85	1	1	288	west wind	956	5.2	0	23
2021-03-03 10:00:00	-9.2	80	1.3	1	251	west wind	956	7.9	0	23
2021-03-03 11:00:00	-5.6	70	1.5	1	275	west wind	956	9.1	0	23
2021-03-03 12:00:00	-1.8	59	1.1	1	303	northwest wind	956	11.3	0	90
2021-03-03 13:00:00	0.8	64	2	2	169	south wind	956	12.9	0	90
2021-03-03 14:00:00	2	63	2.5	2	145	southeast wind	957	14	0	90

2021-03-03 15:00:00	1.6	65	1.3	1	164	south wind	957	19.1	0	23
2021-03-03 16:00:00	2.3	64	2.5	2	167	south wind	957	16.8	0	23
2021-03-03 17:00:00	2.6	64	1.7	2	128	southeast wind	957	18.3	0	23
2021-03-03 18:00:00	2.6	63	1.9	2	165	south wind	958	18.4	0	23
2021-03-03 19:00:00	1.9	64	1.6	2	198	south wind	959	17.5	0	23
2021-03-03 20:00:00	1.2	69	0.9	1	233	southwest wind	960	14.2	0	23
2021-03-03 21:00:00	-0.2	77	0.9	1	223	southwest wind	961	22.2	0	90
2021-03-03 22:00:00	-0.4	77	2	2	184	south wind	961	28.3	0	90
2021-03-03 23:00:00	-0.1	70	4	3	137	southeast wind	962	30	0	90
2021-03-04 00:00:00	-0.6	74	4	3	135	southeast wind	962	30	0	90
2021-03-04 01:00:00	-1.1	78	1.4	1	95	east wind	963	15.4	0	90
2021-03-04 02:00:00	-1.9	87	1.3	1	149	southeast wind	963	14	0	90
2021-03-04 03:00:00	-2	86	1.2	1	187	south wind	963	9.9	0	90
2021-03-04 04:00:00	-2.3	87	2.2	2	136	southeast wind	963	12.1	0	90
2021-03-04 05:00:00	-2.2	85	1.2	1	156	southeast wind	962	11.4	0	90
2021-03-04 06:00:00	-2.4	89	0.5	1	172	south wind	962	13.5	0	90
2021-03-04 07:00:00	-2.3	87	0.5	1	146	southeast wind	962	13.6	0	90
2021-03-04 08:00:00	-2.2	85	0.8	1	8	north wind	963	12.1	0	90
2021-03-04 09:00:00	-2.1	85	0.8	1	100	east wind	963	6.3	0	23
2021-03-04 10:00:00	-2	85	0.7	1	176	south wind	963	8.6	0	23
2021-03-04 11:00:00	-1.8	84	1	1	170	south wind	963	10.3	0	23
2021-03-04 12:00:00	-1.1	76	1.3	1	131	southeast wind	963	10.1	0	82
2021-03-04 13:00:00	-1	75	1.3	1	120	southeast wind	962	8	0	82
2021-03-04 14:00:00	-0.6	76	1.9	2	115	southeast wind	962	7.6	0	82
2021-03-04 15:00:00	-0.6	75	1.5	1	124	southeast wind	962	7.1	0	82
2021-03-04 16:00:00	-0.7	79	1.9	2	57	northeast wind	962	6.5	0	82
2021-03-04 17:00:00	-1	83	2.3	2	68	east wind	962	5.1	0	82
2021-03-04 18:00:00	-1.3	80	2.4	2	61	northeast wind	962	4.1	0	82
2021-03-04 19:00:00	-1.6	83	1.2	1	28	northeast wind	962	3.9	0	82
2021-03-04 20:00:00	-1.9	85	0.6	1	357	north wind	962	3.9	0	82
2021-03-04 21:00:00	-2.2	87	0.1	0	314	northwest wind	963	3.3	0	10
2021-03-04 22:00:00	-2.2	88	0.5	1	170	south wind	963	3.7	0	10
2021-03-04 23:00:00	-2.2	87	0.2	0	349	north wind	962	3.5	0	10
2021-03-05 00:00:00	-2.1	90	0.6	1	5	north wind	962	2.9	0	10
2021-03-05 01:00:00	-2.1	92	0.6	1	15	north wind	962	2.6	0	10
2021-03-05 02:00:00	-2.2	94	0.9	1	38	northeast wind	961	2.6	0	10
2021-03-05 03:00:00	-2.1	95	1.4	1	50	northeast wind	961	2.4	0	10
2021-03-05 04:00:00	-2.3	96	1.3	1	73	east wind	960	2.1	0	10

2021-03-05 05:00:00	-2.3	97	0.8	1	14	north wind	960	2.5	0	10
2021-03-05 06:00:00	-2.4	97	1.1	1	357	north wind	960	2.7	0	10
2021-03-05 07:00:00	-2.5	97	1.1	1	25	northeast wind	960	1.5	0	10
2021-03-05 08:00:00	-2.6	98	1.3	1	29	northeast wind	960	0.2	0	10
2021-03-05 09:00:00	-2.7	99	1.5	1	24	northeast wind	960	0.3	0	10
2021-03-05 10:00:00	-2.9	100	1.5	1	76	east wind	962	3	0	10
2021-03-05 11:00:00	-2.7	100	0.7	1	144	southeast wind	964	2.7	0	10
2021-03-05 12:00:00	-2.6	100	1.9	2	59	northeast wind	965	2.9	0	90
2021-03-05 13:00:00	-2.5	99	0.9	1	349	north wind	965	1.1	0	90
2021-03-05 14:00:00	-2.1	97	1	1	303	northwest wind	967	1.1	0	90
2021-03-05 15:00:00	-1.3	94	1.1	1	334	northwest wind	967	2.5	0	90
2021-03-05 16:00:00	-0.3	85	1.1	1	325	northwest wind	967	4.3	0	90
2021-03-05 17:00:00	-0.4	87	1	1	303	northwest wind	968	6	0	90
2021-03-05 18:00:00	-0.2	83	1.1	1	298	northwest wind	968	6.7	0	90
2021-03-05 19:00:00	-0.4	82	0	0	249	west wind	969	4.7	0	90
2021-03-05 20:00:00	-0.7	89	1.2	1	255	west wind	969	3.7	0	90
2021-03-05 21:00:00	-0.5	89	1.2	1	249	west wind	969	5.7	0	82
2021-03-05 22:00:00	-0.5	87	0.7	1	308	northwest wind	969	6.2	0	82
2021-03-05 23:00:00	-0.4	87	0.7	1	86	east wind	969	4.6	0	82
2021-03-06 00:00:00	-0.9	91	0.7	1	161	south wind	969	4.6	0	82
2021-03-06 01:00:00	-0.8	92	0.5	1	193	south wind	969	6.4	0	82
2021-03-06 02:00:00	-1.4	94	2.7	2	68	east wind	968	0.2	0	82
2021-03-06 03:00:00	-2.5	98	1.5	1	14	north wind	968	0.2	0	82
2021-03-06 04:00:00	-2.5	99	0.8	1	117	southeast wind	967	0.2	0	82
2021-03-06 05:00:00	-2.7	100	1.2	1	322	northwest wind	966	1.3	0	82
2021-03-06 06:00:00	-2.6	100	1	1	256	west wind	966	2	0	82
2021-03-06 07:00:00	-2.3	100	1	1	245	southwest wind	966	2	0	82
2021-03-06 08:00:00	-2.2	100	0.9	1	207	southwest wind	966	1.5	0	82
2021-03-06 09:00:00	-2.1	100	1	1	244	southwest wind	965	1.9	0	23
2021-03-06 10:00:00	-1.6	100	1	1	244	southwest wind	965	2.3	0	23
2021-03-06 11:00:00	-1	98	0.8	1	306	northwest wind	965	4.1	0	23
2021-03-06 12:00:00	-0.7	92	1.6	2	58	northeast wind	965	3.7	0	10
2021-03-06 13:00:00	-0.2	90	1.8	2	111	east wind	964	3.1	0	10
2021-03-06 14:00:00	-1.3	95	2	2	73	east wind	963	0.4	0	10
2021-03-06 15:00:00	-1.9	98	2.4	2	79	east wind	962	0.6	0	23
2021-03-06 16:00:00	-1.9	99	2.3	2	75	east wind	961	1.7	0	23
2021-03-06 17:00:00	-1.9	98	1.4	1	56	northeast wind	961	2.5	0	23
2021-03-06 18:00:00	-1.6	97	1.2	1	35	northeast wind	960	2.7	0	82

2021-03-06 19:00:00	-2	97	1.2	1	9	north wind	960	1.5	0	82
2021-03-06 20:00:00	-2.1	98	0.9	1	326	northwest wind	960	2.9	0	82
2021-03-06 21:00:00	-2.2	99	0.8	1	325	northwest wind	960	4.2	0	10
2021-03-06 22:00:00	-2.2	98	1	1	10	north wind	960	3.6	0	10
2021-03-06 23:00:00	-2.2	98	0.9	1	7	north wind	960	2.5	0	10
2021-03-07 00:00:00	-2.3	98	0.9	1	326	northwest wind	960	4.1	0	10
2021-03-07 01:00:00	-2.4	98	0.3	1	314	northwest wind	959	4.3	0	10
2021-03-07 02:00:00	-2.4	98	0.7	1	36	northeast wind	959	3.9	0	10
2021-03-07 03:00:00	-2.5	97	0.1	0	335	northwest wind	958	3.6	0	10
2021-03-07 04:00:00	-2.5	97	0.9	1	40	northeast wind	957	2.2	0	10
2021-03-07 05:00:00	-2.5	98	0.6	1	359	north wind	957	1.9	0	10
2021-03-07 06:00:00	-2.6	99	0.9	1	35	northeast wind	957	0.9	0	10
2021-03-07 07:00:00	-2.7	99	0.8	1	23	northeast wind	957	1.7	0	10
2021-03-07 08:00:00	-2.8	99	0	0	308	northwest wind	957	1.4	0	10
2021-03-07 09:00:00	-2.8	100	0.9	1	257	west wind	958	2.1	0	90
2021-03-07 10:00:00	-2.2	99	1.1	1	243	southwest wind	958	3.2	0	90
2021-03-07 11:00:00	-1.2	95	0.9	1	210	southwest wind	959	5.7	0	90
2021-03-07 12:00:00	-0.3	81	1	1	311	northwest wind	959	6.9	0	90
2021-03-07 13:00:00	1	74	1.5	1	308	northwest wind	958	7.9	0	90
2021-03-07 14:00:00	1.4	68	1	1	299	northwest wind	958	13	0	90
2021-03-07 15:00:00	2.4	60	1.3	1	226	southwest wind	958	13.6	0	90
2021-03-07 16:00:00	2.1	59	1.8	2	308	northwest wind	957	14.2	0	90
2021-03-07 17:00:00	2.2	59	1.6	2	293	northwest wind	957	13.3	0	90
2021-03-07 18:00:00	2.2	59	1.3	1	333	northwest wind	958	13.6	0	90
2021-03-07 19:00:00	2.6	63	1.2	1	169	south wind	958	13.4	0	90
2021-03-07 20:00:00	0.6	77	0.9	1	24	northeast wind	958	6.6	0	90
2021-03-07 21:00:00	-1.5	87	0.5	1	285	west wind	959	7.5	0	23
2021-03-07 22:00:00	-2.6	85	1	1	140	southeast wind	959	10.4	0	23
2021-03-07 23:00:00	-3.8	90	0.9	1	329	northwest wind	959	4.9	0	23
2021-03-08 00:00:00	-4.4	91	1	1	251	west wind	959	6.7	0	23
2021-03-08 01:00:00	-5.4	92	0.5	1	268	west wind	958	9.2	0	23
2021-03-08 02:00:00	-4.4	93	0.9	1	199	south wind	958	7.8	0	23
2021-03-08 03:00:00	-4.6	93	1.1	1	248	west wind	957	4	0	23
2021-03-08 04:00:00	-4.6	96	1.1	1	242	southwest wind	957	0.3	0	23
2021-03-08 05:00:00	-4	98	1.3	1	225	southwest wind	957	0.3	0	23
2021-03-08 06:00:00	-4.1	99	1.1	1	215	southwest wind	957	1.7	0	23
2021-03-08 07:00:00	-5.1	99	1.6	2	225	southwest wind	957	1.8	0	23
2021-03-08 08:00:00	-5.2	99	1.4	1	215	southwest wind	958	1.3	0	23

2021-03-08 09:00:00	-5.4	99	1.3	1	219	southwest wind	958	1.9	0	23
2021-03-08 10:00:00	-4.8	99	1.6	2	254	west wind	959	1.9	0	23
2021-03-08 11:00:00	-4.6	99	1.5	1	248	west wind	959	2.1	0	23
2021-03-08 12:00:00	-3.7	98	1.2	1	228	southwest wind	959	3.2	0	23
2021-03-08 13:00:00	-2.9	95	0.8	1	173	south wind	959	5.9	0	23
2021-03-08 14:00:00	-2	85	1.2	1	265	west wind	959	6.2	0	23
2021-03-08 15:00:00	-1.4	82	1.3	1	168	south wind	958	7.4	0	90
2021-03-08 16:00:00	-0.3	77	1.1	1	131	southeast wind	958	5	0	90
2021-03-08 17:00:00	-0.4	82	1.9	2	105	east wind	958	4.3	0	90
2021-03-08 18:00:00	-0.8	85	1.6	2	114	southeast wind	959	4.2	0	90
2021-03-08 19:00:00	-0.9	85	1.4	1	314	northwest wind	960	4.2	0	90
2021-03-08 20:00:00	-1.1	88	1.6	2	115	southeast wind	960	3.8	0	90
2021-03-08 21:00:00	-2.1	91	1	1	3	north wind	962	3.1	0	23
2021-03-08 22:00:00	-2.6	94	1.2	1	244	southwest wind	962	2.8	0	23
2021-03-08 23:00:00	-2.7	94	1	1	300	northwest wind	962	2.4	0	23
2021-03-09 00:00:00	-3.3	95	1	1	170	south wind	962	4.1	0	23
2021-03-09 01:00:00	-3.5	95	1.6	2	248	west wind	962	4.5	0	23
2021-03-09 02:00:00	-3.8	94	1.9	2	254	west wind	961	6.5	0	23
2021-03-09 03:00:00	-3.4	91	2	2	260	west wind	961	9.3	0	23
2021-03-09 04:00:00	-3.1	87	2.1	2	265	west wind	962	18.9	0	23
2021-03-09 05:00:00	-4.4	82	1	1	327	northwest wind	962	25.6	0	23
2021-03-09 06:00:00	-5.5	87	0.8	1	294	northwest wind	962	21.8	0	23
2021-03-09 07:00:00	-5.9	90	0.9	1	271	west wind	962	8.1	0	23
2021-03-09 08:00:00	-6.4	91	0.8	1	288	west wind	962	4.8	0	23
2021-03-09 09:00:00	-7.4	91	1.1	1	1	north wind	963	12.6	0	23
2021-03-09 10:00:00	-4.9	80	0.9	1	53	northeast wind	964	3.1	0	23
2021-03-09 11:00:00	-3.6	97	2.2	2	90	east wind	965	0.2	0	23
2021-03-09 12:00:00	-3	99	1.8	2	112	east wind	965	0.1	0	10
2021-03-09 13:00:00	-2.9	100	2	2	73	east wind	965	0.2	0	10
2021-03-09 14:00:00	-2.5	100	0.8	1	138	southeast wind	964	0.2	0	10
2021-03-09 15:00:00	-2	100	1.3	1	289	west wind	963	5.3	0	82
2021-03-09 16:00:00	-1.2	94	1.1	1	327	northwest wind	962	6.4	0	82
2021-03-09 17:00:00	-1.2	89	1.6	2	285	west wind	962	6.8	0	82
2021-03-09 18:00:00	-0.9	89	2	2	237	southwest wind	962	5	0	82
2021-03-09 19:00:00	-1.1	88	1.3	1	265	west wind	962	4.1	0	82
2021-03-09 20:00:00	-1.3	89	1.2	1	308	northwest wind	962	4.7	0	82
2021-03-09 21:00:00	-1.7	90	0.6	1	126	southeast wind	962	4.3	0	10
2021-03-09 22:00:00	-2.6	94	0.8	1	177	south wind	962	2.5	0	10

2021-03-09 23:00:00	-2.6	95	0.7	1	246	southwest wind	961	2.9	0	10
2021-03-10 00:00:00	-3	95	1	1	255	west wind	961	3.3	0	10
2021-03-10 01:00:00	-3	95	1.1	1	195	south wind	960	1.8	0	10
2021-03-10 02:00:00	-3.3	97	1.1	1	274	west wind	959	0.2	0	10
2021-03-10 03:00:00	-3.8	98	0.5	1	220	southwest wind	959	0.2	0	10
2021-03-10 04:00:00	-3.9	99	1.6	2	225	southwest wind	958	0.3	0	10
2021-03-10 05:00:00	-4.5	100	1.5	1	246	southwest wind	957	0.4	0	10
2021-03-10 06:00:00	-5	100	1.1	1	214	southwest wind	957	0.4	0	10
2021-03-10 07:00:00	-4.9	100	1.4	1	242	southwest wind	957	0.3	0	10
2021-03-10 08:00:00	-4.8	100	1.5	1	241	southwest wind	957	0.5	0	10
2021-03-10 09:00:00	-4.6	100	1.7	2	250	west wind	957	0.5	0	10
2021-03-10 10:00:00	-4.6	100	1.4	1	230	southwest wind	957	1.2	0	10
2021-03-10 11:00:00	-4.4	100	1.3	1	256	west wind	957	2.2	0	10
2021-03-10 12:00:00	-3.5	99	1.7	2	230	southwest wind	956	7	0	10
2021-03-10 13:00:00	-1.4	92	1.7	2	298	northwest wind	956	11.1	0	10
2021-03-10 14:00:00	1.2	77	0.8	1	193	south wind	955	12	0	10
2021-03-10 15:00:00	2.3	70	1.5	1	39	northeast wind	955	6.4	0	23
2021-03-10 16:00:00	2.1	74	1.5	1	92	east wind	954	6.2	0	23
2021-03-10 17:00:00	3.2	70	1.2	1	94	east wind	954	6.8	0	23
2021-03-10 18:00:00	1.1	82	1.9	2	90	east wind	954	5	0	82
2021-03-10 19:00:00	-0.1	88	1.4	1	83	east wind	955	3	0	82
2021-03-10 20:00:00	-1.6	94	1	1	10	north wind	955	1.4	0	82
2021-03-10 21:00:00	-1.8	97	1.4	1	170	south wind	956	0.7	0	82
2021-03-10 22:00:00	-1.7	98	1	1	150	southeast wind	956	0.1	0	82
2021-03-10 23:00:00	-1.5	99	1.2	1	98	east wind	956	0.2	0	82
2021-03-11 00:00:00	-1.5	100	0.9	1	109	east wind	956	0.1	0	82
2021-03-11 01:00:00	-1.5	100	0.4	1	44	northeast wind	956	0.1	0	82
2021-03-11 02:00:00	-1.6	100	0.4	1	335	northwest wind	956	0.2	0	82
2021-03-11 03:00:00	-1.4	100	1.2	1	240	southwest wind	956	0.5	0	82
2021-03-11 04:00:00	-1.9	100	1	1	309	northwest wind	956	2.5	0	82
2021-03-11 05:00:00	-1.8	100	0.8	1	179	south wind	956	1.7	0	82
2021-03-11 06:00:00	-1.4	100	1.3	1	102	east wind	955	0.2	0	82
2021-03-11 07:00:00	-1.6	100	1.2	1	22	north wind	956	0.1	0	82
2021-03-11 08:00:00	-1.7	100	1	1	327	northwest wind	956	0.6	0	82
2021-03-11 09:00:00	-1.5	100	1.1	1	114	southeast wind	956	0.2	0	23
2021-03-11 10:00:00	-1.6	100	1.3	1	141	southeast wind	957	0.2	0	23
2021-03-11 11:00:00	-1	100	0.8	1	164	south wind	957	0.5	0	23
2021-03-11 12:00:00	-0.9	100	2.5	2	159	south wind	957	2.1	0	23

2021-03-11 13:00:00	0	100	1	1	168	south wind	958	4.2	0	23
2021-03-11 14:00:00	0.6	96	1	1	76	east wind	957	4.3	0	23
2021-03-11 15:00:00	1.7	85	1.6	2	216	southwest wind	957	5.6	0	82
2021-03-11 16:00:00	2.6	80	0.7	1	89	east wind	956	7.1	0	82
2021-03-11 17:00:00	2.5	82	1.8	2	345	north wind	957	4.5	0	82
2021-03-11 18:00:00	2.7	80	1.1	1	308	northwest wind	957	6.6	0	90
2021-03-11 19:00:00	3	78	0.9	1	331	northwest wind	957	8.7	0	90
2021-03-11 20:00:00	2.7	79	1.2	1	235	southwest wind	958	7.7	0	90
2021-03-11 21:00:00	2.1	81	1.5	1	252	west wind	958	6.6	0	90
2021-03-11 22:00:00	1.6	84	1.7	2	248	west wind	958	6.7	0	90
2021-03-11 23:00:00	1.5	86	1.9	2	268	west wind	958	9.3	0	90
2021-03-12 00:00:00	0.7	88	0.7	1	305	northwest wind	958	6.1	0	90
2021-03-12 01:00:00	-1.2	93	0.9	1	174	south wind	959	3.8	0	90
2021-03-12 02:00:00	-1.7	95	0.8	1	301	northwest wind	958	3.3	0	90
2021-03-12 03:00:00	-2.8	97	0.8	1	272	west wind	958	4.1	0	90
2021-03-12 04:00:00	-3.2	97	0.8	1	300	northwest wind	958	8.4	0	90
2021-03-12 05:00:00	-3.6	97	0.4	1	269	west wind	958	8.8	0	90
2021-03-12 06:00:00	-4.5	97	0.5	1	95	east wind	959	2.1	0	90
2021-03-12 07:00:00	-2.5	98	0.3	1	244	southwest wind	959	0.1	0	90
2021-03-12 08:00:00	-2.1	99	1.2	1	255	west wind	959	0.1	0	90
2021-03-12 09:00:00	-1.8	100	1.4	1	266	west wind	959	0.1	0	23
2021-03-12 10:00:00	-1.4	100	0.6	1	273	west wind	959	0.6	0	23
2021-03-12 11:00:00	-1.1	100	1.6	2	72	east wind	959	0.4	0	23
2021-03-12 12:00:00	-0.8	100	1.5	1	103	east wind	959	1.6	0	23
2021-03-12 13:00:00	-0.6	100	0.9	1	27	northeast wind	958	1.4	0	23
2021-03-12 14:00:00	-0.2	100	1.3	1	9	north wind	957	5.3	0	23
2021-03-12 15:00:00	0.2	100	1.4	1	21	north wind	956	4.1	0	82
2021-03-12 16:00:00	0.8	98	0.8	1	0	north wind	955	6.9	0	82
2021-03-12 17:00:00	0.9	90	1.5	1	264	west wind	954	6.6	0	82
2021-03-12 18:00:00	1	89	1.8	2	258	west wind	954	8.8	0	82
2021-03-12 19:00:00	1	88	1.3	1	247	southwest wind	954	7.5	0	82
2021-03-12 20:00:00	0.9	89	1.9	2	256	west wind	954	7.8	0	82
2021-03-12 21:00:00	0.2	90	0.6	1	263	west wind	954	6	0	23
2021-03-12 22:00:00	-0.8	94	1	1	357	north wind	954	6.7	0	23
2021-03-12 23:00:00	-1.5	96	1	1	331	northwest wind	954	0.3	0	23
2021-03-13 00:00:00	-2.3	98	1.7	2	304	northwest wind	953	0.3	0	23
2021-03-13 01:00:00	-2.2	99	1.3	1	270	west wind	952	0.3	0	23
2021-03-13 02:00:00	-3.1	100	1	1	245	southwest wind	952	5.1	0	23

2021-03-13 03:00:00	-2.5	100	0.7	1	196	south wind	951	5.4	0	23
2021-03-13 04:00:00	-1.8	100	0.6	1	101	east wind	950	0.4	0	23
2021-03-13 05:00:00	-1.7	100	0.6	1	168	south wind	950	0.3	0	23
2021-03-13 06:00:00	-2.2	100	1	1	289	west wind	949	0.3	0	23
2021-03-13 07:00:00	-2.9	100	1.9	2	264	west wind	949	0.5	0	23
2021-03-13 08:00:00	-3.2	100	1.5	1	265	west wind	949	1.7	0	23
2021-03-13 09:00:00	-3.5	100	1.1	1	281	west wind	949	7.1	0	10
2021-03-13 10:00:00	-1.8	100	1.8	2	262	west wind	950	11.5	0	10
2021-03-13 11:00:00	0.2	95	1.4	1	244	southwest wind	950	15.8	0	10
2021-03-13 12:00:00	2	77	1.9	2	89	east wind	950	11.4	0	23
2021-03-13 13:00:00	3.1	75	1	1	143	southeast wind	949	7.4	0	23
2021-03-13 14:00:00	2.8	80	1.5	1	35	northeast wind	948	9.5	0	23
2021-03-13 15:00:00	6.3	63	0.6	1	17	north wind	948	18.3	0	23
2021-03-13 16:00:00	6.8	60	1	1	193	south wind	947	15.6	0	23
2021-03-13 17:00:00	6.8	59	1.2	1	296	northwest wind	947	20	0	23
2021-03-13 18:00:00	6.4	57	1.3	1	295	northwest wind	947	27.9	0	90
2021-03-13 19:00:00	6.1	62	1.1	1	331	northwest wind	947	23.2	0	90
2021-03-13 20:00:00	4.6	67	1	1	280	west wind	948	20.9	0	90
2021-03-13 21:00:00	2.3	79	1.4	1	231	southwest wind	949	7.8	0	90
2021-03-13 22:00:00	2.1	85	2.6	2	156	southeast wind	950	22.4	0	90
2021-03-13 23:00:00	1	76	2.5	2	109	east wind	951	28	0	90
2021-03-14 00:00:00	0.2	76	1.8	2	73	east wind	951	27.5	0	90
2021-03-14 01:00:00	-0.4	78	0.9	1	91	east wind	952	26.2	0	90
2021-03-14 02:00:00	-1.1	81	0.6	1	341	north wind	952	26.5	0	90
2021-03-14 03:00:00	-1.9	87	1	1	174	south wind	952	27	0	90
2021-03-14 04:00:00	-1.6	88	0.7	1	292	west wind	953	24.1	0	90
2021-03-14 05:00:00	-1.8	87	0.5	1	343	north wind	953	24.3	0	90
2021-03-14 06:00:00	-1.4	90	1.2	1	227	southwest wind	953	21.8	0	90
2021-03-14 07:00:00	-1.2	86	0.8	1	71	east wind	954	25.3	0	90
2021-03-14 08:00:00	-1.3	86	0.4	1	311	northwest wind	955	15.7	0	90
2021-03-14 09:00:00	-1.3	90	0.9	1	264	west wind	954	2.7	0	90
2021-03-14 10:00:00	-1.2	96	0.7	1	93	east wind	955	2	0	90
2021-03-14 11:00:00	-1.7	97	3.4	3	62	northeast wind	955	2.8	0	90
2021-03-14 12:00:00	-2.7	98	3.1	2	81	east wind	955	4.2	0	90
2021-03-14 13:00:00	-3	99	2.5	2	66	northeast wind	955	3.1	0	90
2021-03-14 14:00:00	-3.2	99	2.1	2	72	east wind	955	4.3	0	90
2021-03-14 15:00:00	-3.2	98	2.2	2	81	east wind	955	4	0	90
2021-03-14 16:00:00	-3.3	97	1.9	2	64	northeast wind	955	7.5	0	90

2021-03-14 17:00:00	-3.4	94	2.6	2	67	northeast wind	955	15.2	0	90
2021-03-14 18:00:00	-3.4	91	1.5	1	22	north wind	956	8.9	0	90
2021-03-14 19:00:00	-3.4	90	0.9	1	337	northwest wind	957	1.2	0	90
2021-03-14 20:00:00	-3.5	95	0.8	1	279	west wind	957	0.8	0	90
2021-03-14 21:00:00	-3.5	96	0.9	1	160	south wind	959	0.7	0	90
2021-03-14 22:00:00	-3.4	97	0.6	1	273	west wind	959	1.8	0	90
2021-03-14 23:00:00	-3.6	97	1.1	1	120	southeast wind	960	2.5	0	90
2021-03-15 00:00:00	-3.9	97	1.1	1	105	east wind	960	1.2	0	90
2021-03-15 01:00:00	-4	97	1.1	1	172	south wind	961	2.2	0	90
2021-03-15 02:00:00	-4.2	97	0.3	1	113	southeast wind	960	4.8	0	90
2021-03-15 03:00:00	-4.2	97	0.9	1	32	northeast wind	960	7.7	0	90
2021-03-15 04:00:00	-4.4	97	0.8	1	342	north wind	960	8.8	0	90
2021-03-15 05:00:00	-4.4	97	0	0	338	north wind	960	2.4	0	90
2021-03-15 06:00:00	-4.3	97	0.4	1	349	north wind	961	1	0	90
2021-03-15 07:00:00	-4.1	97	0.5	1	348	north wind	961	2.1	0	90
2021-03-15 08:00:00	-4.1	97	0.7	1	82	east wind	962	7.2	0	90
2021-03-15 09:00:00	-3.8	96	0.9	1	330	northwest wind	963	5.8	0	90
2021-03-15 10:00:00	-3.5	95	0.1	0	307	northwest wind	964	2.6	0	90
2021-03-15 11:00:00	-2.7	93	0.7	1	219	southwest wind	964	3.4	0	90
2021-03-15 12:00:00	-1.6	87	0.5	1	171	south wind	965	7.7	0	90
2021-03-15 13:00:00	-1.1	76	2	2	179	south wind	965	11.7	0	90
2021-03-15 14:00:00	-1.2	85	1.6	2	102	east wind	964	5.1	0	90
2021-03-15 15:00:00	-0.8	83	1.6	2	123	southeast wind	964	6.5	0	90
2021-03-15 16:00:00	-1.3	84	2.5	2	126	southeast wind	964	11	0	90
2021-03-15 17:00:00	-1.5	84	0.2	0	126	southeast wind	964	11.8	0	90
2021-03-15 18:00:00	-1.8	88	2.2	2	133	southeast wind	965	8.9	0	82
2021-03-15 19:00:00	-2.1	88	1.8	2	118	southeast wind	965	10.1	0	82
2021-03-15 20:00:00	-2.2	90	1.8	2	138	southeast wind	966	9.2	0	82
2021-03-15 21:00:00	-2.2	90	1.6	2	133	southeast wind	966	7.6	0	90
2021-03-15 22:00:00	-2.4	85	2.8	2	128	southeast wind	966	12.8	0	90
2021-03-15 23:00:00	-2.6	83	1.8	2	88	east wind	966	23.8	0	90
2021-03-16 00:00:00	-3.1	83	1.7	2	97	east wind	966	26.6	0	90
2021-03-16 01:00:00	-3.6	86	2	2	116	southeast wind	966	18.6	0	90
2021-03-16 02:00:00	-4	88	1.3	1	131	southeast wind	966	18.3	0	90
2021-03-16 03:00:00	-4.1	88	1.3	1	164	south wind	966	23.5	0	90
2021-03-16 04:00:00	-4.2	89	0.7	1	163	south wind	966	23.2	0	90
2021-03-16 05:00:00	-4.1	88	0.8	1	70	east wind	965	21.8	0	90
2021-03-16 06:00:00	-4.2	86	1.6	2	77	east wind	965	23.1	0	90

2021-03-16 07:00:00	-4.5	88	1.3	1	36	northeast wind	965	20.4	0	90
2021-03-16 08:00:00	-4.6	89	0.8	1	119	southeast wind	965	16.1	0	90
2021-03-16 09:00:00	-4.5	89	0.9	1	162	south wind	965	15.4	0	10
2021-03-16 10:00:00	-4	87	0.6	1	171	south wind	965	11.6	0	10
2021-03-16 11:00:00	-3.7	86	1.2	1	81	east wind	966	11.1	0	10
2021-03-16 12:00:00	-3.5	86	1.3	1	126	southeast wind	966	10	0	10
2021-03-16 13:00:00	-2.8	82	1.1	1	156	southeast wind	965	10.4	0	10
2021-03-16 14:00:00	-2.8	83	1.5	1	51	northeast wind	965	10.3	0	10
2021-03-16 15:00:00	-2.2	83	1.9	2	133	southeast wind	965	8.5	0	90
2021-03-16 16:00:00	-2.6	84	2.3	2	91	east wind	964	7.9	0	90
2021-03-16 17:00:00	-3.1	86	1.7	2	100	east wind	965	2	0	90
2021-03-16 18:00:00	-3.1	87	1.4	1	98	east wind	965	2.3	0	90
2021-03-16 19:00:00	-3.3	90	1.3	1	98	east wind	965	4.8	0	90
2021-03-16 20:00:00	-3.6	91	1.1	1	120	southeast wind	966	2.7	0	90
2021-03-16 21:00:00	-3.8	94	1.1	1	120	southeast wind	966	3.1	0	90
2021-03-16 22:00:00	-4.1	94	1.4	1	181	south wind	966	4.2	0	90
2021-03-16 23:00:00	-5.2	95	0.1	0	146	southeast wind	966	2.2	0	90
2021-03-17 00:00:00	-6.3	96	0.6	1	205	southwest wind	966	4.1	0	90
2021-03-17 01:00:00	-5.8	97	0.7	1	197	south wind	965	6	0	90
2021-03-17 02:00:00	-6.1	96	0.9	1	178	south wind	965	5.9	0	90
2021-03-17 03:00:00	-5.7	96	0.7	1	136	southeast wind	965	3.5	0	90
2021-03-17 04:00:00	-5.5	97	1.1	1	61	northeast wind	965	2.4	0	90
2021-03-17 05:00:00	-5.6	97	1.2	1	155	southeast wind	964	2.3	0	90
2021-03-17 06:00:00	-6.2	97	1.6	2	226	southwest wind	965	1.7	0	90
2021-03-17 07:00:00	-5.9	98	1.4	1	253	west wind	965	3.6	0	90
2021-03-17 08:00:00	-5.9	97	1.1	1	255	west wind	966	3.8	0	90
2021-03-17 09:00:00	-5.6	95	0.9	1	239	southwest wind	966	2.8	0	23
2021-03-17 10:00:00	-5.2	90	1.3	1	224	southwest wind	966	5.2	0	23
2021-03-17 11:00:00	-3.7	80	1.2	1	236	southwest wind	967	7.1	0	23
2021-03-17 12:00:00	-2.3	73	1.5	1	253	west wind	966	9.9	0	23
2021-03-17 13:00:00	-0.6	64	1.1	1	140	southeast wind	966	8.6	0	23
2021-03-17 14:00:00	-1.4	74	2.3	2	90	east wind	966	7.5	0	23
2021-03-17 15:00:00	-0.9	72	2.3	2	118	southeast wind	966	7.8	0	82
2021-03-17 16:00:00	-1.1	72	2.1	2	113	southeast wind	966	7.4	0	82
2021-03-17 17:00:00	-1.3	72	1.9	2	78	east wind	966	9.2	0	82
2021-03-17 18:00:00	-1	71	1.9	2	119	southeast wind	967	10.3	0	82
2021-03-17 19:00:00	-1.3	72	1.5	1	123	southeast wind	967	8	0	82
2021-03-17 20:00:00	-2.6	79	1.4	1	6	north wind	968	6	0	82

2021-03-17 21:00:00	-3.5	86	0.9	1	360	north wind	969	5.8	0	10
2021-03-17 22:00:00	-3.9	88	0.3	1	250	west wind	969	5.4	0	10
2021-03-17 23:00:00	-4.1	88	0.7	1	349	north wind	970	4.8	0	10
2021-03-18 00:00:00	-4.3	89	0.9	1	346	north wind	970	5.4	0	10
2021-03-18 01:00:00	-5.1	88	0.8	1	359	north wind	970	5.1	0	10
2021-03-18 02:00:00	-6.1	89	1.6	2	263	west wind	970	6.1	0	10
2021-03-18 03:00:00	-7.6	89	1.3	1	257	west wind	970	5.7	0	10
2021-03-18 04:00:00	-8.6	87	1.6	2	267	west wind	970	10.1	0	10
2021-03-18 05:00:00	-9.3	87	1.4	1	270	west wind	970	10.1	0	10
2021-03-18 06:00:00	-10.2	88	0.7	1	261	west wind	971	9.1	0	10
2021-03-18 07:00:00	-10	90	1.6	2	264	west wind	972		0	10
2021-03-18 08:00:00	-9.9	86	2.1	2	267	west wind	973	10.8	0	10
2021-03-18 09:00:00	-9.3	83	1.8	2	271	west wind	973	14.1	0	23
2021-03-18 10:00:00	-7.5	78	2.1	2	272	west wind	974	11.6	0	23
2021-03-18 11:00:00	-4.7	71	2.2	2	254	west wind	974	13.9	0	23
2021-03-18 12:00:00	-1.1	63	1.1	1	260	west wind	974	14.3	0	23
2021-03-18 13:00:00	0.6	54	1.3	1	34	northeast wind	974	20.2	0	23
2021-03-18 14:00:00	2	54	1.2	1	99	east wind	973	21.9	0	23
2021-03-18 15:00:00	2.1	57	2.1	2	121	southeast wind	973	11.3	0	82
2021-03-18 16:00:00	2.5	57	1.6	2	93	east wind	972	10.5	0	82
2021-03-18 17:00:00	2.1	62	2	2	121	southeast wind	973	13	0	82
2021-03-18 18:00:00	1.9	57	1.6	2	105	east wind	973	14.2	0	82
2021-03-18 19:00:00	1.2	63	1.7	2	68	east wind	973	12.9	0	82
2021-03-18 20:00:00	-0.1	68	2	2	47	northeast wind	973	9.8	0	82
2021-03-18 21:00:00	-1.9	79	0.3	1	129	southeast wind	974	6.9	0	10
2021-03-18 22:00:00	-2.7	84	1.5	1	254	west wind	974	5.8	0	10
2021-03-18 23:00:00	-3.9	86	1.2	1	307	northwest wind	973	8.5	0	10
2021-03-19 00:00:00	-5.2	82	2.1	2	256	west wind	973	15.2	0	10
2021-03-19 01:00:00	-5.5	87	0.9	1	116	southeast wind	973	9.9	0	10
2021-03-19 02:00:00	-5.6	90	1	1	353	north wind	973		0	10
2021-03-19 03:00:00	-5.2	94	1.1	1	322	northwest wind	973		0	10
2021-03-19 04:00:00	-4.5	93	1.2	1	297	northwest wind	972		0	10
2021-03-19 05:00:00	-4	88	1.1	1	303	northwest wind	972		0	10
2021-03-19 06:00:00	-3.7	85	1.1	1	268	west wind	972		0	10
2021-03-19 07:00:00	-3.6	82	1	1	294	northwest wind	972		0	10
2021-03-19 08:00:00	-3.8	84	2	2	54	northeast wind	972	6.9	0	10
2021-03-19 09:00:00	-4.1	92	1.9	2	41	northeast wind	972	4.8	0	10
2021-03-19 10:00:00	-3.9	90	1.4	1	31	northeast wind	972	3.7	0	10

2021-03-19 11:00:00	-3.4	85	1.3	1	13	north wind	972	4.1	0	10
2021-03-19 12:00:00	-3.2	81	1.8	2	33	northeast wind	972	4.3	0	10
2021-03-19 13:00:00	-2.9	81	2.3	2	17	north wind	972	4.8	0	10
2021-03-19 14:00:00	-2.5	79	2.7	2	74	east wind	972	13.6	0	10
2021-03-19 15:00:00	-1.9	78	2.4	2	64	northeast wind	971	15.1	0	82
2021-03-19 16:00:00	-1.7	78	1.7	2	44	northeast wind	971	18.1	0	82
2021-03-19 17:00:00	-1.5	75	1.7	2	72	east wind	970	18	0	82
2021-03-19 18:00:00	-1.5	79	1.3	1	8	north wind	970	15.5	0	82
2021-03-19 19:00:00	-1.8	80	1.1	1	349	north wind	970	13.3	0	82
2021-03-19 20:00:00	-2.2	84	1	1	319	northwest wind	970	6	0	82
2021-03-19 21:00:00	-2.5	88	1.1	1	335	northwest wind	970		0	23
2021-03-19 22:00:00	-2.8	86	1.1	1	296	northwest wind	970		0	23
2021-03-19 23:00:00	-2.9	84	0.7	1	326	northwest wind	970	5.6	0	23
2021-03-20 00:00:00	-3	86	0.8	1	298	northwest wind	970	7.6	0	23
2021-03-20 01:00:00	-3.1	88	0.9	1	287	west wind	970	5.8	0	23
2021-03-20 02:00:00	-3.3	90	0.9	1	252	west wind	969	7.5	0	23
2021-03-20 03:00:00	-3.5	90	1.2	1	263	west wind	968	9	0	23
2021-03-20 04:00:00	-3.6	91	0.9	1	306	northwest wind	968	6.9	0	23
2021-03-20 05:00:00	-3.7	89	1.7	2	260	west wind	968	7.2	0	23
2021-03-20 06:00:00	-4.1	90	1.5	1	153	southeast wind	967	6.9	0	23
2021-03-20 07:00:00	-4.3	90	1.3	1	171	south wind	967	9.4	0	23
2021-03-20 08:00:00	-4	89	1.1	1	256	west wind	967	8.1	0	23
2021-03-20 09:00:00	-3.5	87	0.8	1	278	west wind	967	7.4	0	82
2021-03-20 10:00:00	-2.9	87	0.9	1	353	north wind	967	3.8	0	82
2021-03-20 11:00:00	-2.3	84	1	1	136	southeast wind	967	5	0	82
2021-03-20 12:00:00	-2.1	86	2.1	2	83	east wind	967	5.3	0	23
2021-03-20 13:00:00	-1.7	86	1.9	2	44	northeast wind	967	6.2	0	23
2021-03-20 14:00:00	-0.9	76	1.9	2	49	northeast wind	966	7.6	0	23
2021-03-20 15:00:00	-0.8	76	2.4	2	34	northeast wind	966	9.9	0	82
2021-03-20 16:00:00	-0.7	77	2	2	40	northeast wind	965	9.8	0	82
2021-03-20 17:00:00	-1	77	1.6	2	36	northeast wind	965	10.9	0	82
2021-03-20 18:00:00	-0.4	71	1.8	2	330	northwest wind	964	13.7	0	90
2021-03-20 19:00:00	-1.2	74	1.4	1	281	west wind	964	11.4	0	90
2021-03-20 20:00:00	-2	75	0.9	1	305	northwest wind	964	11.8	0	90
2021-03-20 21:00:00	-2.5	76	1.3	1	257	west wind	963	11.6	0	82
2021-03-20 22:00:00	-2.9	76	1.9	2	246	southwest wind	963	11.4	0	82
2021-03-20 23:00:00	-3.2	80	1.7	2	237	southwest wind	962	10.2	0	82
2021-03-21 00:00:00	-2.8	80	2.1	2	251	west wind	961	12.2	0	82

2021-03-21 01:00:00	-2.7	72	3.3	2	259	west wind	960	14.7	0	82
2021-03-21 02:00:00	-3	71	2.6	2	262	west wind	960	18.3	0	82
2021-03-21 03:00:00	-3.4	68	2.2	2	264	west wind	959	23.1	0	82
2021-03-21 04:00:00	-4.1	68	2.1	2	262	west wind	958	26.3	0	82
2021-03-21 05:00:00	-4.3	68	1.8	2	275	west wind	957	26.1	0	82
2021-03-21 06:00:00	-4.8	68	2.7	2	262	west wind	956	25.7	0	82
2021-03-21 07:00:00	-4.6	67	3.4	3	259	west wind	956	25.3	0	82
2021-03-21 08:00:00	-4	66	3.3	2	257	west wind	956	20.7	0	82
2021-03-21 09:00:00	-2.7	64	3.6	3	265	west wind	956	20.7	0	23
2021-03-21 10:00:00	-0.4	56	4.7	3	260	west wind	956	21.6	0	23
2021-03-21 11:00:00	0.9	56	4.2	3	269	west wind	956	23.3	0	23
2021-03-21 12:00:00	3	50	3.4	3	271	west wind	955	23.8	0	23
2021-03-21 13:00:00	4.7	43	3.2	2	261	west wind	955	26.8	0	23
2021-03-21 14:00:00	5.4	37	2.3	2	259	west wind	954	28.6	0	23
2021-03-21 15:00:00	6.2	35	1.7	2	280	west wind	954	25.2	0	90
2021-03-21 16:00:00	6.2	40	1.9	2	295	northwest wind	954	24.2	0	90
2021-03-21 17:00:00	6.1	36	1.4	1	295	northwest wind	954	29.6	0	90
2021-03-21 18:00:00	4.8	55	1.9	2	75	east wind	955	21.4	0	90
2021-03-21 19:00:00	5.4	50	0.5	1	198	south wind	955	21.4	0	90
2021-03-21 20:00:00	4.1	58	0.8	1	336	northwest wind	956	19.2	0	90
2021-03-21 21:00:00	2.9	66	1.2	1	266	west wind	957	14.7	0	23
2021-03-21 22:00:00	2.2	76	1.1	1	177	south wind	958	11.9	0	23
2021-03-21 23:00:00	2.1	77	0.4	1	323	northwest wind	958	11	0	23
2021-03-22 00:00:00	2	76	0.7	1	1	north wind	957	10.1	0	23
2021-03-22 01:00:00	1.5	76	0.7	1	234	southwest wind	957	6.6	0	23
2021-03-22 02:00:00	1	80	1.1	1	356	north wind	957	11.6	0	23
2021-03-22 03:00:00	0.8	81	0.3	1	273	west wind	956	10.6	0	23
2021-03-22 04:00:00	0.5	83	0.9	1	322	northwest wind	956	10.6	0	23
2021-03-22 05:00:00	0.2	86	0.7	1	325	northwest wind	956	12.5	0	23
2021-03-22 06:00:00	0.5	79	1.1	1	298	northwest wind	957	15.5	0	23
2021-03-22 07:00:00	-0.1	84	0.7	1	269	west wind	957	13.9	0	23
2021-03-22 08:00:00	0.1	84	1.5	1	258	west wind	958	9.9	0	23
2021-03-22 09:00:00	0.5	79	1.4	1	277	west wind	959	8.7	0	90
2021-03-22 10:00:00	2.5	72	1.3	1	246	southwest wind	960	18.9	0	90
2021-03-22 11:00:00	5.3	58	1.6	2	248	west wind	960	20.2	0	90
2021-03-22 12:00:00	7.9	46	0.8	1	328	northwest wind	960	19.9	0	10
2021-03-22 13:00:00	9.5	42	0.8	1	94	east wind	959	19.5	0	10
2021-03-22 14:00:00	9.3	45	1.8	2	26	northeast wind	959	22.1	0	10

2021-03-22 15:00:00	10.1	42	2.3	2	355	north wind	958	19.5	0	23
2021-03-22 16:00:00	9.7	45	1.8	2	5	north wind	958	19.5	0	23
2021-03-22 17:00:00	10.1	42	2.1	2	346	north wind	957	22.5	0	23
2021-03-22 18:00:00	10.5	35	1.8	2	346	north wind	957	25.1	0	23
2021-03-22 19:00:00	9.7	40	1	1	31	northeast wind	956	24.8	0	23
2021-03-22 20:00:00	7.8	52	0.7	1	69	east wind	956	17.3	0	23
2021-03-22 21:00:00	5.9	60	1.1	1	301	northwest wind	957	21.5	0	82
2021-03-22 22:00:00	3.7	70	1.1	1	250	west wind	957	13.7	0	82
2021-03-22 23:00:00	2.3	78	2	2	255	west wind	957	15.5	0	82
2021-03-23 00:00:00	1.3	81	1.3	1	281	west wind	957	17.7	0	82
2021-03-23 01:00:00	0.4	84	1.8	2	269	west wind	957	16	0	82
2021-03-23 02:00:00	0.2	86	1.8	2	256	west wind	957	15.2	0	82
2021-03-23 03:00:00	-0.1	85	2	2	260	west wind	956	14	0	82
2021-03-23 04:00:00	-0.2	89	1.6	2	253	west wind	956	13.7	0	82
2021-03-23 05:00:00	0.3	89	2.4	2	257	west wind	956	15.3	0	82
2021-03-23 06:00:00	0.4	87	2	2	269	west wind	956	15.4	0	82
2021-03-23 07:00:00	0.1	88	2.2	2	266	west wind	956	11.1	0	82
2021-03-23 08:00:00	-0.4	86	2.3	2	265	west wind	956	8.3	0	82
2021-03-23 09:00:00	-0.1	89	2.2	2	255	west wind	956	9.3	0	10
2021-03-23 10:00:00	1.9	81	1.5	1	261	west wind	955	9	0	10
2021-03-23 11:00:00	3.9	71	1.5	1	250	west wind	955	10.4	0	10
2021-03-23 12:00:00	5.2	64	1.4	1	276	west wind	954	12	0	82
2021-03-23 13:00:00	7.1	58	1.4	1	296	northwest wind	954	11.5	0	82
2021-03-23 14:00:00	7.7	54	2	2	348	north wind	953	11.5	0	82
2021-03-23 15:00:00	8.2	55	1.8	2	331	northwest wind	952	10.6	0	82
2021-03-23 16:00:00	9	53	1.8	2	327	northwest wind	951	11.8	0	82
2021-03-23 17:00:00	8.4	55	3.1	2	298	northwest wind	951	11.9	0	82
2021-03-23 18:00:00	8.8	52	2.1	2	349	north wind	950	13.5	0	23
2021-03-23 19:00:00	8.5	53	1.7	2	346	north wind	950	13.4	0	23
2021-03-23 20:00:00	7.4	62	1.5	1	283	west wind	951	12.1	0	23
2021-03-23 21:00:00	5.6	70	1.1	1	259	west wind	951	10.8	0	23
2021-03-23 22:00:00	4.1	77	1.8	2	252	west wind	951	10	0	23
2021-03-23 23:00:00	4.1	78	1.5	1	268	west wind	951	9.6	0	23
2021-03-24 00:00:00	4.3	80	1.9	2	261	west wind	951	10.8	0	23
2021-03-24 01:00:00	4.1	79	1.1	1	305	northwest wind	951	13.9	0	23
2021-03-24 02:00:00	5	72	2	2	336	northwest wind	952	14	0	23
2021-03-24 03:00:00	4.6	79	1.9	2	242	southwest wind	952	10.9	0	23
2021-03-24 04:00:00	4.6	79	1.9	2	277	west wind	953	15	0	23

2021-03-24 05:00:00	4.1	76	1.7	2	185	south wind	953	14	0	23
2021-03-24 06:00:00	4.3	77	1.1	1	309	northwest wind	954	15.3	0	23
2021-03-24 07:00:00	4	76	1.1	1	280	west wind	955	17.3	0	23
2021-03-24 08:00:00	3.1	81	1.7	2	252	west wind	955	12.5	0	23
2021-03-24 09:00:00	3.5	84	0.8	1	267	west wind	956	10.6	0	82
2021-03-24 10:00:00	5.4	76	0.7	1	268	west wind	957	15.8	0	82
2021-03-24 11:00:00	7.7	75	3.2	2	150	southeast wind	957	18.3	0	82
2021-03-24 12:00:00	8.6	70	3.7	3	159	south wind	958	28	0	23
2021-03-24 13:00:00	9.7	64	2.8	2	174	south wind	958	26.9	0	23
2021-03-24 14:00:00	10.6	57	3.9	3	122	southeast wind	958	27.6	0	23
2021-03-24 15:00:00	11.3	57	2.9	2	112	east wind	958	18.9	0	23
2021-03-24 16:00:00	11.2	60	4.3	3	123	southeast wind	958	18.9	0	23
2021-03-24 17:00:00	10.5	65	1.8	2	3	north wind	958	19	0	23
2021-03-24 18:00:00	10.6	62	1.2	1	118	southeast wind	958	20.9	0	23
2021-03-24 19:00:00	9.8	58	3.4	3	129	southeast wind	959	13	0	23
2021-03-24 20:00:00	8.2	54	2.7	2	152	southeast wind	959	15.9	0	23
2021-03-24 21:00:00	6.4	52	2.2	2	126	southeast wind	960	22.9	0	23
2021-03-24 22:00:00	5.7	55	2.4	2	128	southeast wind	960	26.8	0	23
2021-03-24 23:00:00	5	57	1.6	2	43	northeast wind	960	23.8	0	23
2021-03-25 00:00:00	4.5	58	1.5	1	64	northeast wind	960	26.7	0	23
2021-03-25 01:00:00	3.3	64	0.9	1	310	northwest wind	960	24.5	0	23
2021-03-25 02:00:00	2.5	74	0.9	1	264	west wind	960	22.9	0	23
2021-03-25 03:00:00	2.3	73	1.5	1	250	west wind	959	22.8	0	23
2021-03-25 04:00:00	2.8	73	1.4	1	254	west wind	959	21.2	0	23
2021-03-25 05:00:00	3.2	72	1.2	1	267	west wind	958	22.3	0	23
2021-03-25 06:00:00	2.8	75	1.2	1	260	west wind	958	20.8	0	23
2021-03-25 07:00:00	1.7	80	1.5	1	258	west wind	959	16.7	0	23
2021-03-25 08:00:00	1.9	83	0.9	1	295	northwest wind	959	16	0	23
2021-03-25 09:00:00	3.4	68	1.1	1	338	north wind	959	24.8	0	82
2021-03-25 10:00:00	5.3	58	1.5	1	29	northeast wind	959	27	0	82
2021-03-25 11:00:00	6.2	55	1.4	1	326	northwest wind	959	27	0	82
2021-03-25 12:00:00	7.4	56	1.3	1	36	northeast wind	958	29.2	0	10
2021-03-25 13:00:00	7.9	59	1.9	2	1	north wind	957	27.5	0	10
2021-03-25 14:00:00	8.9	60	2.3	2	59	northeast wind	956	23.5	0	10
2021-03-25 15:00:00	9.3	60	2.4	2	25	northeast wind	955	19.8	0	23
2021-03-25 16:00:00	9.6	58	2.4	2	82	east wind	954	20.8	0	23
2021-03-25 17:00:00	9.9	57	1.8	2	69	east wind	953	22.4	0	23
2021-03-25 18:00:00	9.9	59	2.2	2	68	east wind	953	19.6	0	10

2021-03-25 19:00:00	9.7	58	1.8	2	74	east wind	953	18.4	0	10
2021-03-25 20:00:00	9.2	61	1.8	2	69	east wind	952	17	0	10
2021-03-25 21:00:00	7.9	68	1.4	1	350	north wind	952	18.8	0	10
2021-03-25 22:00:00	6.3	70	0.7	1	308	northwest wind	953	20.5	0	10
2021-03-25 23:00:00	5	79	0.9	1	251	west wind	953	9.8	0	10
2021-03-26 00:00:00	4.1	83	1	1	251	west wind	952	10.6	0	10
2021-03-26 01:00:00	4.1	86	1	1	260	west wind	951	11.8	0	10
2021-03-26 02:00:00	3.4	88	0.9	1	265	west wind	951	12.1	0	10
2021-03-26 03:00:00	2.8	91	0.6	1	275	west wind	950	11.9	0	10
2021-03-26 04:00:00	2.3	92	1.3	1	251	west wind	949	11.2	0	10
2021-03-26 05:00:00	2.1	93	1.1	1	256	west wind	948	10.6	0	10
2021-03-26 06:00:00	1.6	93	1.4	1	326	northwest wind	948	7.8	0	10
2021-03-26 07:00:00	1.4	94	1	1	247	southwest wind	948	9.6	0	10
2021-03-26 08:00:00	2.2	91	2.1	2	258	west wind	948	9.8	0	10
2021-03-26 09:00:00	2.9	90	2.2	2	258	west wind	948	11.1	0	82
2021-03-26 10:00:00	5.5	81	2.5	2	253	west wind	948	15.3	0	82
2021-03-26 11:00:00	7.5	74	2	2	267	west wind	948	15.7	0	82
2021-03-26 12:00:00	9.7	66	1.6	2	222	southwest wind	947	16.7	0	23
2021-03-26 13:00:00	11.2	56	1.4	1	81	east wind	947	17.1	0	23
2021-03-26 14:00:00	12	56	0.5	1	318	northwest wind	947	17.3	0	23
2021-03-26 15:00:00	13.2	49	1	1	351	north wind	947	18.8	0	90
2021-03-26 16:00:00	13.8	50	2.5	2	132	southeast wind	947	18.5	0	90
2021-03-26 17:00:00	14.1	49	1.9	2	104	east wind	947	18	0	90
2021-03-26 18:00:00	14.2	51	2.1	2	110	east wind	947	21.5	0	90
2021-03-26 19:00:00	13.7	55	0.9	1	135	southeast wind	948	16.4	0	90
2021-03-26 20:00:00	13.2	54	0.8	1	82	east wind	949	13.4	0	90
2021-03-26 21:00:00	11.6	71	1	1	34	northeast wind	950	11.5	0	82
2021-03-26 22:00:00	10.6	78	1	1	296	northwest wind	950	7.8	0	82
2021-03-26 23:00:00	9.7	81	1.2	1	211	southwest wind	950	7.8	0	82
2021-03-27 00:00:00	8.4	87	0.8	1	125	southeast wind	950	6.3	0	82
2021-03-27 01:00:00	7	92	0.8	1	349	north wind	950	7.1	0	82
2021-03-27 02:00:00	6.8	91	1.3	1	25	northeast wind	950	9.2	0	82
2021-03-27 03:00:00	5.8	91	1	1	17	north wind	951	8.5	0	82
2021-03-27 04:00:00	5.5	94	1.3	1	307	northwest wind	952	7.9	0	82
2021-03-27 05:00:00	4.2	93	0.7	1	201	south wind	952	6.4	0	82
2021-03-27 06:00:00	3.5	96	0.9	1	270	west wind	952	5.8	0	82
2021-03-27 07:00:00	3.1	97	1.5	1	269	west wind	953	5.1	0	82
2021-03-27 08:00:00	3	97	1.3	1	296	northwest wind	953	5.7	0	82

2021-03-27 09:00:00	3.8	97	0.4	1	344	north wind	954	6.4	0	23
2021-03-27 10:00:00	5.4	93	1.2	1	168	south wind	954	9.4	0	23
2021-03-27 11:00:00	8.6	77	1	1	66	northeast wind	954	22.7	0	23
2021-03-27 12:00:00	10	67	1.6	2	57	northeast wind	954	21.8	0	10
2021-03-27 13:00:00	12.2	63	1	1	105	east wind	953	17.6	0	10
2021-03-27 14:00:00	13.5	58	1.4	1	81	east wind	953	16.4	0	10
2021-03-27 15:00:00	14.9	56	1.3	1	156	southeast wind	952	17.5	0	82
2021-03-27 16:00:00	15.8	54	2	2	107	east wind	951	19.8	0	82
2021-03-27 17:00:00	16.6	55	2.5	2	128	southeast wind	950	20.8	0	82
2021-03-27 18:00:00	16.4	54	2.3	2	126	southeast wind	950	21.4	0	23
2021-03-27 19:00:00	15.8	57	2.3	2	117	southeast wind	949	18.6	0	23
2021-03-27 20:00:00	14.1	65	2.1	2	92	east wind	950	16.9	0	23
2021-03-27 21:00:00	12.6	72	1.5	1	82	east wind	950	13.4	0	10
2021-03-27 22:00:00	11.3	76	1.8	2	36	northeast wind	950	12.7	0	10
2021-03-27 23:00:00	9.4	85	0.6	1	335	northwest wind	950	9.5	0	10
2021-03-28 00:00:00	7.9	90	1.5	1	263	west wind	950	4.7	0	10
2021-03-28 01:00:00	7	93	1.3	1	262	west wind	949	4.1	0	10
2021-03-28 02:00:00	6.4	95	1.7	2	259	west wind	948	3.9	0	10
2021-03-28 03:00:00	6.8	96	1.6	2	267	west wind	948	5.3	0	10
2021-03-28 04:00:00	7.1	96	2.3	2	256	west wind	947	5.9	0	10
2021-03-28 05:00:00	7.9	92	2.7	2	260	west wind	947	7.5	0	10
2021-03-28 06:00:00	8.2	90	1.3	1	277	west wind	947	7.3	0	10
2021-03-28 07:00:00	8.7	85	1	1	36	northeast wind	948	22.4	0	10
2021-03-28 08:00:00	8.9	76	1.4	1	22	north wind	947	24.4	0	10
2021-03-28 09:00:00	8.6	77	1	1	337	northwest wind	947	23.9	0	90
2021-03-28 10:00:00	9	75	0.9	1	336	northwest wind	946	21.5	0	90
2021-03-28 11:00:00	10.3	69	2	2	351	north wind	946	28.5	0	90
2021-03-28 12:00:00	10.4	69	2.4	2	34	northeast wind	946	29.9	0	23
2021-03-28 13:00:00	10.5	69	2.1	2	19	north wind	945	25	0	23
2021-03-28 14:00:00	11.6	66	1.7	2	80	east wind	945	30	0	23
2021-03-28 15:00:00	12.3	63	1.2	1	16	north wind	944	27	0	82
2021-03-28 16:00:00	12.1	63	1.2	1	98	east wind	944	24.5	0	82
2021-03-28 17:00:00	12	64	1.3	1	76	east wind	943	16.3	0	82
2021-03-28 18:00:00	11.1	73	1.8	2	100	east wind	943	15.9	0	90
2021-03-28 19:00:00	10.6	79	1.6	2	92	east wind	944	18.1	0	90
2021-03-28 20:00:00	9.8	80	2.1	2	107	east wind	944	15.1	0	90
2021-03-28 21:00:00	9.1	84	0.2	0	101	east wind	944	14.4	0	90
2021-03-28 22:00:00	8.5	87	1.1	1	272	west wind	945	11.2	0	90

2021-03-28 23:00:00	7.8	91	1.3	1	280	west wind	946	11.8	0	90
2021-03-29 00:00:00	6.2	93	1.3	1	270	west wind	946	4	0	90
2021-03-29 01:00:00	5.6	96	1.3	1	255	west wind	946	5.1	0	90
2021-03-29 02:00:00	5.3	97	1.2	1	281	west wind	946	5.5	0	90
2021-03-29 03:00:00	4.3	97	1.3	1	237	southwest wind	947	4.8	0	90
2021-03-29 04:00:00	4.4	98	1.4	1	269	west wind	947	5.8	0	90
2021-03-29 05:00:00	3.6	97	0.9	1	204	southwest wind	947	4.7	0	90
2021-03-29 06:00:00	4	98	1.6	2	265	west wind	948	5.7	0	90
2021-03-29 07:00:00	3.3	97	1.3	1	262	west wind	948	5.2	0	90
2021-03-29 08:00:00	2.9	98	0.7	1	302	northwest wind	949	3.6	0	90
2021-03-29 09:00:00	3.7	99	0.8	1	223	southwest wind	950	15	0	10
2021-03-29 10:00:00	7.7	78	0.8	1	65	northeast wind	950	30	0	10
2021-03-29 11:00:00	10.9	57	2.6	2	109	east wind	950	30	0	10
2021-03-29 12:00:00	11.6	55	1.9	2	116	southeast wind	950	30	0	10
2021-03-29 13:00:00	12.3	53	1.6	2	116	southeast wind	950	30	0	10
2021-03-29 14:00:00	13.1	50	1.9	2	151	southeast wind	950	29.5	0	10
2021-03-29 15:00:00	13	53	2.2	2	99	east wind	951	24.9	0	90
2021-03-29 16:00:00	12.8	59	2.7	2	102	east wind	951	23.2	0	90
2021-03-29 17:00:00	13	57	2.3	2	73	east wind	951	22.1	0	90
2021-03-29 18:00:00	12.2	60	3.5	3	113	southeast wind	952	16.8	0	90
2021-03-29 19:00:00	8.9	62	3.6	3	102	east wind	953	13.1	0	90
2021-03-29 20:00:00	7.2	76	2.9	2	246	southwest wind	956	15	0	90
2021-03-29 21:00:00	5.9	87	0.9	1	288	west wind	957	18.9	0	90
2021-03-29 22:00:00	6.1	73	1.6	2	82	east wind	958	30	0	90
2021-03-29 23:00:00	5.3	81	2.1	2	137	southeast wind	959	30	0	90
2021-03-30 00:00:00	4.5	85	3.5	3	138	southeast wind	959	30	0	90
2021-03-30 01:00:00	4.7	82	2.3	2	120	southeast wind	960	30	0	90
2021-03-30 02:00:00	4.7	80	2.7	2	120	southeast wind	960	30	0	90
2021-03-30 03:00:00	4.9	74	2.1	2	86	east wind	960	30	0	90
2021-03-30 04:00:00	4.5	73	4.3	3	123	southeast wind	960	30	0	90
2021-03-30 05:00:00	3.6	78	1.7	2	104	east wind	960	30	0	90
2021-03-30 06:00:00	3.5	76	1.9	2	116	southeast wind	960	30	0	90
2021-03-30 07:00:00	3.7	74	2.3	2	106	east wind	961	30	0	90
2021-03-30 08:00:00	3.8	70	1.6	2	94	east wind	961	30	0	90
2021-03-30 09:00:00	4	68	1	1	91	east wind	962	30	0	23
2021-03-30 10:00:00	4.3	69	1.3	1	87	east wind	962	30	0	23
2021-03-30 11:00:00	4.8	69	1.2	1	111	east wind	962	30	0	23
2021-03-30 12:00:00	7	67	1.1	1	144	southeast wind	962	30	0	23

2021-03-30 13:00:00	7.2	64	2.1	2	175	south wind	961	30	0	23
2021-03-30 14:00:00	7.6	66	1.8	2	161	south wind	961	30	0	23
2021-03-30 15:00:00	8.3	64	3.6	3	126	southeast wind	961	30	0	23
2021-03-30 16:00:00	8.5	64	2.3	2	116	southeast wind	961	30	0	23
2021-03-30 17:00:00	8.7	58	1.8	2	86	east wind	961	30	0	23
2021-03-30 18:00:00	8.5	60	3.1	2	157	southeast wind	961	30	0	82
2021-03-30 19:00:00	8.4	58	3.3	2	145	southeast wind	962	30	0	82
2021-03-30 20:00:00	7.8	57	2	2	133	southeast wind	962	25.7	0	82
2021-03-30 21:00:00	7.4	59	3	2	138	southeast wind	963	30	0	90
2021-03-30 22:00:00	6.9	63	2.5	2	110	east wind	963	30	0	90
2021-03-30 23:00:00	5.8	72	2.9	2	133	southeast wind	964	28.5	0	90
2021-03-31 00:00:00	5.4	75	2.1	2	98	east wind	964	30	0	90
2021-03-31 01:00:00	5.3	76	1.7	2	96	east wind	964	30	0	90
2021-03-31 02:00:00	4.7	83	1.8	2	353	north wind	964	29.6	0	90
2021-03-31 03:00:00	3.5	88	1.4	1	329	northwest wind	964	30	0	90
2021-03-31 04:00:00	3.5	90	1.8	2	219	southwest wind	964	30	0	90
2021-03-31 05:00:00	3.4	91	0.8	1	286	west wind	963	30	0	90
2021-03-31 06:00:00	3.3	91	0.3	1	285	west wind	963	28.5	0	90
2021-03-31 07:00:00	3.2	92	0.6	1	255	west wind	963	29.3	0	90
2021-03-31 08:00:00	2.5	94	0.3	1	0	north wind	963	9.5	0	90
2021-03-31 09:00:00	3	96	0.4	1	154	southeast wind	964	30	0	82
2021-03-31 10:00:00	5	79	1.9	2	127	southeast wind	964	30	0	82
2021-03-31 11:00:00	5.3	78	1.9	2	133	southeast wind	964	30	0	82
2021-03-31 12:00:00	5.3	78	1.9	2	133	southeast wind	964	30	0	23
2021-03-31 13:00:00	5.3	78	1.9	2	133	southeast wind	964	28.4	0	23
2021-03-31 14:00:00	6.2	71	3.2	2	104	east wind	963	30	0	23
2021-03-31 15:00:00	6.3	66	3	2	80	east wind	962	30	0	82
2021-03-31 16:00:00	7.3	67	3.3	2	63	northeast wind	962	30	0	82
2021-03-31 17:00:00	7.7	62	3.7	3	57	northeast wind	962	30	0	82
2021-03-31 18:00:00	7	65	3.3	2	65	northeast wind	962	30	0	82
2021-03-31 19:00:00	7.3	60	3.3	2	81	east wind	962	30	0	82
2021-03-31 20:00:00	6.8	64	2.6	2	87	east wind	962	30	0	82
2021-03-31 21:00:00	6.3	65	2.5	2	88	east wind	963	30	0	82
2021-03-31 22:00:00	4.7	82	2.5	2	15	north wind	964	30	0	82
2021-03-31 23:00:00	4.2	79	1.5	1	32	northeast wind	964	30	0	82
2021-04-01 00:00:00	4.2	77	2.5	2	44	northeast wind	964	30	0	82
2021-04-01 01:00:00	3.8	77	1	1	89	east wind	964	30	0	82
2021-04-01 02:00:00	3.5	79	1	1	72	east wind	964	30	0	82

2021-04-01 03:00:00	3.5	82	1.1	1	46	northeast wind	963	30	0	82
2021-04-01 04:00:00	3.6	82	1.2	1	30	northeast wind	963	30	0	82
2021-04-01 05:00:00	3.4	84	1.3	1	45	northeast wind	963	30	0	82
2021-04-01 06:00:00	3.5	84	0.9	1	93	east wind	963	30	0	82
2021-04-01 07:00:00	3.2	87	0.9	1	307	northwest wind	963	30	0	82
2021-04-01 08:00:00	3.2	87	1.1	1	309	northwest wind	963	27.6	0	82
2021-04-01 09:00:00	3.9	85	1.3	1	305	northwest wind	963	20.8	0	82
2021-04-01 10:00:00	4.6	78	0.7	1	232	southwest wind	963	22.8	0	82
2021-04-01 11:00:00	5.7	73	1.5	1	220	southwest wind	963	26.1	0	82
2021-04-01 12:00:00	7.3	64	0.9	1	124	southeast wind	962	29.5	0	23
2021-04-01 13:00:00	6.6	64	1.5	1	142	southeast wind	962	25.1	0	23
2021-04-01 14:00:00	6.3	67	1.9	2	30	northeast wind	961	30	0	23
2021-04-01 15:00:00	6.6	69	1.7	2	79	east wind	961	28.6	0	93
2021-04-01 16:00:00	6.6	66	1.3	1	55	northeast wind	961	27.9	0	93
2021-04-01 17:00:00	7	62	0.5	1	198	south wind	961	30	0	93
2021-04-01 18:00:00	7.5	60	1.1	1	321	northwest wind	961	30	0	93
2021-04-01 19:00:00	7.5	55	0.9	1	251	west wind	962	26.5	0	93
2021-04-01 20:00:00	7.2	58	0.6	1	48	northeast wind	962	21.8	0	93
2021-04-01 21:00:00	5.6	69	0.8	1	221	southwest wind	962	10	0	23
2021-04-01 22:00:00	4.3	76	1.5	1	326	northwest wind	963	13.2	0	23
2021-04-01 23:00:00	3.3	80	0.9	1	262	west wind	963	12.9	0	23
2021-04-02 00:00:00	3.1	82	1.6	2	267	west wind	963	11.8	0	23
2021-04-02 01:00:00	3.1	85	1.5	1	252	west wind	963	16.4	0	23
2021-04-02 02:00:00	3.5	84	1.5	1	250	west wind	963	14.9	0	23
2021-04-02 03:00:00	3.7	85	1	1	203	southwest wind	964	9.5	0	23
2021-04-02 04:00:00	4	88	1.5	1	242	southwest wind	964	10	0	23
2021-04-02 05:00:00	3.9	91	1.8	2	259	west wind	963	10.2	0	23
2021-04-02 06:00:00	3.6	91	2.4	2	249	west wind	964	11.2	0	23
2021-04-02 07:00:00	2.1	94	2.2	2	255	west wind	965	6.5	0	23
2021-04-02 08:00:00	1.1	96	1.4	1	255	west wind	965	4.5	0	23
2021-04-02 09:00:00	0.6	98	0.7	1	224	southwest wind	966	1.8	0	100
2021-04-02 10:00:00	0.6	98	0.6	1	244	southwest wind	966	2.6	0	100
2021-04-02 11:00:00	1.5	99	1.2	1	271	west wind	967	4	0	100
2021-04-02 12:00:00	1.4	99	1.7	2	236	southwest wind	967	4.6	0	100
2021-04-02 13:00:00	1.1	99	1.3	1	249	west wind	967	4.2	0	100
2021-04-02 14:00:00	1.4	99	2.1	2	241	southwest wind	967	4.7	0	100
2021-04-02 15:00:00	2	99	2.3	2	229	southwest wind	967	10	0	94
2021-04-02 16:00:00	2.5	99	2.3	2	246	southwest wind	967	14.5	0	94

2021-04-02 17:00:00	3.1	98	2.7	2	254	west wind	967	8.1	0	94
2021-04-02 18:00:00	3	96	4.1	3	254	west wind	967	18.1	0	93
2021-04-02 19:00:00	3.2	94	3	2	248	west wind	967	25.6	0	84
2021-04-02 20:00:00	3	93	3.1	2	250	west wind	967	27.2	0	90
2021-04-02 21:00:00	3.1	92	3.3	2	252	west wind	967	27.8	0	91
2021-04-02 22:00:00	3.2	90	3.7	3	250	west wind	967	30	0	83
2021-04-02 23:00:00	2.9	92	3.5	3	256	west wind	968	24.8	0	88
2021-04-03 00:00:00	2.5	94	2.7	2	251	west wind	967	18	0	95
2021-04-03 01:00:00	2.3	95	3.7	3	247	southwest wind	967	30	0	0
2021-04-03 02:00:00	2.3	95	3.5	3	247	southwest wind	966	30	0	74
2021-04-03 03:00:00	2.5	92	3.4	3	248	west wind	966	30	0	90
2021-04-03 04:00:00	2.9	87	3.8	3	250	west wind	965	30	0	41
2021-04-03 05:00:00	3	85	2.3	2	266	west wind	965	30	0	38
2021-04-03 06:00:00	2.8	86	2.6	2	274	west wind	964	30	0	44
2021-04-03 07:00:00	2.9	84	2.1	2	271	west wind	964	30	0	89
2021-04-03 08:00:00	3.1	82	1.9	2	277	west wind	964	30	0	86
2021-04-03 09:00:00	3.3	81	1.6	2	280	west wind	965	30	0	89
2021-04-03 10:00:00	4.2	81	1	1	269	west wind	965	24.1	0	84
2021-04-03 11:00:00	5.3	77	0.9	1	325	northwest wind	964	30	0	80
2021-04-03 12:00:00	6.2	72	1.2	1	94	east wind	964	30	0	86
2021-04-03 13:00:00	6.9	68	1.5	1	163	south wind	964	30	0	90
2021-04-03 14:00:00	8.1	63	0.9	1	228	southwest wind	963	30	0	90
2021-04-03 15:00:00	8.6	62	1.9	2	81	east wind	962	30	0	89
2021-04-03 16:00:00	8.8	62	1.6	2	82	east wind	962	30	0	89
2021-04-03 17:00:00	8.8	54	1.1	1	190	south wind	962	30	0	93
2021-04-03 18:00:00	8.6	51	4.2	3	258	west wind	961	30	0	93
2021-04-03 19:00:00	8.3	53	2	2	71	east wind	961	30	0	81
2021-04-03 20:00:00	7.8	56	0.7	1	67	northeast wind	961	30	0	91
2021-04-03 21:00:00	7.4	60	1.4	1	262	west wind	961	27.7	0	81
2021-04-03 22:00:00	6.1	62	3.8	3	255	west wind	961	30	0	93
2021-04-03 23:00:00	5.6	59	4.3	3	258	west wind	961	30	0	91
2021-04-04 00:00:00	5.3	59	4.2	3	256	west wind	960	30	0	84
2021-04-04 01:00:00	4.8	57	3.8	3	257	west wind	959	30	0	0
2021-04-04 02:00:00	4.3	56	3.1	2	269	west wind	959	30	0	41
2021-04-04 03:00:00	3.6	58	2.9	2	267	west wind	958	30	0	45
2021-04-04 04:00:00	3.3	58	2.9	2	265	west wind	958	30	0	42
2021-04-04 05:00:00	3.3	57	2.5	2	263	west wind	957	30	0	64
2021-04-04 06:00:00	3.1	57	2.8	2	265	west wind	957	30	0	63

2021-04-04 07:00:00	2.6	60	2.7	2	262	west wind	957	30	0	67
2021-04-04 08:00:00	3.1	57	3.6	3	262	west wind	957	30	0	31
2021-04-04 09:00:00	4.3	53	4	3	262	west wind	957	30	0	31
2021-04-04 10:00:00	6.2	48	4.5	3	265	west wind	956	30	0	43
2021-04-04 11:00:00	8.6	43	4.4	3	268	west wind	956	30	0	53
2021-04-04 12:00:00	11	34	3.9	3	257	west wind	955	30	0	0
2021-04-04 13:00:00	12	26	2.7	2	275	west wind	954	30	0	0
2021-04-04 14:00:00	13.1	23	1.6	2	333	northwest wind	953	30	0	0
2021-04-04 15:00:00	13.7	25	1.5	1	56	northeast wind	952	30	0	0
2021-04-04 16:00:00	14.4	28	2.3	2	181	south wind	951	30	0	0
2021-04-04 17:00:00	14.7	25	2.1	2	184	south wind	951	30	0	0
2021-04-04 18:00:00	14.6	22	2.1	2	245	southwest wind	951	30	0	0
2021-04-04 19:00:00	13.9	25	2.1	2	226	southwest wind	950	30	0	0
2021-04-04 20:00:00	12.6	27	1.9	2	224	southwest wind	951	29.1	0	0
2021-04-04 21:00:00	10.2	40	1.8	2	259	west wind	951	21.3	0	0
2021-04-04 22:00:00	7.8	56	1.4	1	237	southwest wind	952	28.5	0	0
2021-04-04 23:00:00	6.8	55	2.2	2	274	west wind	952	30	0	0
2021-04-05 00:00:00	5.8	62	3.1	2	259	west wind	953	30	0	0
2021-04-05 01:00:00	4.7	66	2	2	272	west wind	953	30	0	0
2021-04-05 02:00:00	4.3	65	2	2	256	west wind	953	24.7	0	0
2021-04-05 03:00:00	3.3	70	2.2	2	255	west wind	953	27.1	0	26
2021-04-05 04:00:00	3	68	2.1	2	253	west wind	953	22.4	0	0
2021-04-05 05:00:00	2.6	68	3	2	253	west wind	954	22.9	0	0
2021-04-05 06:00:00	2.5	66	1.8	2	253	west wind	954	21.2	0	0
2021-04-05 07:00:00	3.5	57	2.9	2	263	west wind	955	19.1	0	85
2021-04-05 08:00:00	2.9	64	1.8	2	232	southwest wind	955	19.6	0	69
2021-04-05 09:00:00	3.7	60	2.8	2	254	west wind	956	16.7	0	57
2021-04-05 10:00:00	6.4	56	3	2	261	west wind	956	14.4	0	83
2021-04-05 11:00:00	9.9	45	3.7	3	258	west wind	957	20.3	0	29
2021-04-05 12:00:00	13.2	33	2.7	2	254	west wind	957	23.4	0	0
2021-04-05 13:00:00	14.7	28	2.3	2	264	west wind	957	29.9	0	38
2021-04-05 14:00:00	15.8	22	1.7	2	241	southwest wind	957	21.4	0	67
2021-04-05 15:00:00	16.4	22	1.5	1	219	southwest wind	956	21.2	0	83
2021-04-05 16:00:00	17.6	22	1.7	2	120	southeast wind	956	18.7	0	64
2021-04-05 17:00:00	17.8	19	1.7	2	59	northeast wind	956	18.9	0	85
2021-04-05 18:00:00	18	20	1.6	2	134	southeast wind	956	19.7	0	59
2021-04-05 19:00:00	16.8	28	2.4	2	119	southeast wind	957	17.7	0	0
2021-04-05 20:00:00	15.3	35	2.6	2	107	east wind	957	18	0	57

2021-04-05 21:00:00	13.4	41	1.1	1	107	east wind	958	13.7	0	39
2021-04-05 22:00:00	11.8	49	0.4	1	285	west wind	959	11.8	0	50
2021-04-05 23:00:00	11.8	63	1.5	1	120	southeast wind	960	22	0	52
2021-04-06 00:00:00	10.6	69	0.4	1	312	northwest wind	960	18.8	0	23
2021-04-06 01:00:00	9.8	74	0.8	1	131	southeast wind	961	22.4	0	23
2021-04-06 02:00:00	8.3	81	0.6	1	255	west wind	961	18	0	23
2021-04-06 03:00:00	7.2	75	1.7	2	267	west wind	961		0	23
2021-04-06 04:00:00	7.2	75	1.9	2	262	west wind	961		0	23
2021-04-06 05:00:00	5.4	86	2.6	2	255	west wind	961		0	23
2021-04-06 06:00:00	5.1	83	2.8	2	258	west wind	961		0	23
2021-04-06 07:00:00	5.7	74	2.7	2	256	west wind	961		0	23
2021-04-06 08:00:00	5.7	73	2.7	2	258	west wind	962		0	23
2021-04-06 09:00:00	5.6	74	2.7	2	257	west wind	962		0	25
2021-04-06 10:00:00	6.3	73	3.2	2	255	west wind	962	19.8	0	39
2021-04-06 11:00:00	10.5	57	2.2	2	249	west wind	962	20	0	62
2021-04-06 12:00:00	13.2	53	1.3	1	219	southwest wind	962	27.5	0	71
2021-04-06 13:00:00	14.7	49	1.3	1	174	south wind	961	20.8	0	0
2021-04-06 14:00:00	16.3	36	1.8	2	142	southeast wind	961	22.8	0	0
2021-04-06 15:00:00	17.5	38	1.4	1	214	southwest wind	960	25.3	0	0
2021-04-06 16:00:00	17.4	33	1.7	2	68	east wind	960	14.9	0	0
2021-04-06 17:00:00	17.9	35	2.2	2	144	southeast wind	959	6.5	0	71
2021-04-06 18:00:00	17.7	35	2.2	2	133	southeast wind	959	7.1	0	0
2021-04-06 19:00:00	17.3	37	2.6	2	108	east wind	959	21.7	0	0
2021-04-06 20:00:00	16.3	42	3.2	2	138	southeast wind	960	14.9	0	0
2021-04-06 21:00:00	14.5	46	1	1	151	southeast wind	960	17.5	0	0
2021-04-06 22:00:00	12.9	52	0.8	1	294	northwest wind	961	14.9	0	0
2021-04-06 23:00:00	11.3	64	1.2	1	294	northwest wind	961	14.6	0	0
2021-04-07 00:00:00	9.5	71	1.3	1	251	west wind	961	13.4	0	0
2021-04-07 01:00:00	8.1	77	1.2	1	232	southwest wind	961	13.6	0	0
2021-04-07 02:00:00	7.8	75	0.8	1	250	west wind	961	20.3	0	73
2021-04-07 03:00:00	7.5	71	1.5	1	216	southwest wind	961	23.3	0	0
2021-04-07 04:00:00	7.1	69	2.1	2	266	west wind	961	26.3	0	34
2021-04-07 05:00:00	6.8	68	2.8	2	266	west wind	961	25.9	0	23
2021-04-07 06:00:00	6.2	69	2.7	2	261	west wind	960	25.3	0	52
2021-04-07 07:00:00	5.4	75	2.1	2	261	west wind	960	24.1	0	49
2021-04-07 08:00:00	5.8	73	2.3	2	264	west wind	961	20.4	0	23
2021-04-07 09:00:00	7.3	68	2.2	2	253	west wind	961	21.8	0	63
2021-04-07 10:00:00	10.2	60	2.2	2	257	west wind	961	23.1	0	68

2021-04-07 11:00:00	12.3	55	0.7	1	171	south wind	961	19.4	0	88
2021-04-07 12:00:00	14.1	47	1.3	1	18	north wind	961	20.4	0	74
2021-04-07 13:00:00	15	44	2	2	51	northeast wind	960	24.5	0	87
2021-04-07 14:00:00	16.5	39	1.8	2	63	northeast wind	960	29.1	0	87
2021-04-07 15:00:00	17.2	36	1.9	2	20	north wind	959	27.9	0	72
2021-04-07 16:00:00	17.4	37	2.1	2	96	east wind	959	26.4	0	89
2021-04-07 17:00:00	17.2	36	2	2	55	northeast wind	959	28	0	92
2021-04-07 18:00:00	17	40	1.7	2	113	southeast wind	959	25.9	0	85
2021-04-07 19:00:00	16.8	36	1.2	1	127	southeast wind	959	24.1	0	86
2021-04-07 20:00:00	16	45	1.1	1	158	south wind	960	14.3	0	85
2021-04-07 21:00:00	14.5	49	0.1	0	0	north wind	960	8.8	0	89
2021-04-07 22:00:00	12.9	57	0.7	1	303	northwest wind	961	10.2	0	77
2021-04-07 23:00:00	11.2	67	1.3	1	316	northwest wind	961	7.6	0	0
2021-04-08 00:00:00	9.6	74	0.9	1	277	west wind	961	13.8	0	82
2021-04-08 01:00:00	8.9	77	1.6	2	255	west wind	961	20.3	0	0
2021-04-08 02:00:00	8.9	78	1.6	2	259	west wind	961	20.2	0	61
2021-04-08 03:00:00	8.8	78	1.9	2	259	west wind	961	21.7	0	75
2021-04-08 04:00:00	8.5	76	1.8	2	249	west wind	961	22.6	0	81
2021-04-08 05:00:00	7.7	77	2.1	2	249	west wind	961	23.5	0	30
2021-04-08 06:00:00	7.5	74	2.6	2	260	west wind	961	25.8	0	39
2021-04-08 07:00:00	7.2	74	2.4	2	249	west wind	961	23.9	0	49
2021-04-08 08:00:00	6.9	73	2.7	2	257	west wind	961	23.5	0	0
2021-04-08 09:00:00	8.8	67	2	2	249	west wind	961	24.1	0	0
2021-04-08 10:00:00	12	58	2.3	2	248	west wind	961	18.8	0	0
2021-04-08 11:00:00	15	46	1.7	2	268	west wind	961	22	0	60
2021-04-08 12:00:00	16.8	42	0.9	1	346	north wind	961	20.9	0	0
2021-04-08 13:00:00	17.9	40	2.1	2	56	northeast wind	960	24.5	0	77
2021-04-08 14:00:00	18.6	37	1.8	2	75	east wind	960	23.1	0	0
2021-04-08 15:00:00	19	36	2	2	139	southeast wind	959	26	0	66
2021-04-08 16:00:00	19.5	33	2.1	2	144	southeast wind	959	27.7	0	38
2021-04-08 17:00:00	19.9	32	2.7	2	173	south wind	958	30	0	43
2021-04-08 18:00:00	19.6	31	2	2	108	east wind	958	25.1	0	0
2021-04-08 19:00:00	19.2	33	1.4	1	88	east wind	958	25.8	0	37
2021-04-08 20:00:00	18.4	32	1.6	2	77	east wind	958	14.7	0	0
2021-04-08 21:00:00	16	40	1	1	7	north wind	959	11.5	0	0
2021-04-08 22:00:00	13.6	54	1.2	1	16	north wind	959	7.1	0	50
2021-04-08 23:00:00	11.8	61	1	1	273	west wind	959	10.1	0	33
2021-04-09 00:00:00	10.3	70	0.6	1	272	west wind	959	12.4	0	77

2021-04-09 01:00:00	9.4	74	2	2	257	west wind	959	19.6	0	0
2021-04-09 02:00:00	9.7	73	2.2	2	259	west wind	959	19.3	0	42
2021-04-09 03:00:00	9	75	1.9	2	257	west wind	959	19.2	0	27
2021-04-09 04:00:00	9	74	2.9	2	256	west wind	958	22.6	0	0
2021-04-09 05:00:00	8.1	74	1.6	2	264	west wind	958	23.8	0	25
2021-04-09 06:00:00	7.8	74	1.8	2	263	west wind	958	24.2	0	0
2021-04-09 07:00:00	7.3	75	1.7	2	261	west wind	958	23.1	0	0
2021-04-09 08:00:00	7.1	73	2	2	264	west wind	958	23	0	0
2021-04-09 09:00:00	9.3	66	2.1	2	257	west wind	958	23.3	0	0
2021-04-09 10:00:00	13.2	51	2.5	2	259	west wind	958	28.2	0	0
2021-04-09 11:00:00	16.2	41	1	1	292	west wind	958	27	0	0
2021-04-09 12:00:00	18.2	37	0.9	1	266	west wind	958	26.7	0	0
2021-04-09 13:00:00	19.3	33	1.4	1	139	southeast wind	957	25.1	0	0
2021-04-09 14:00:00	19.9	30	2.3	2	115	southeast wind	957	30	0	0
2021-04-09 15:00:00	20.5	25	2.4	2	55	northeast wind	956	28.9	0	0
2021-04-09 16:00:00	20.8	23	1.6	2	331	northwest wind	956	30	0	0
2021-04-09 17:00:00	20.8	23	1.6	2	331	northwest wind	956	29	0	0
2021-04-09 18:00:00	21.2	21	2.3	2	59	northeast wind	955	27.3	0	0
2021-04-09 19:00:00	21.3	22	1.2	1	42	northeast wind	955	28.7	0	0
2021-04-09 20:00:00	20.3	24	1.9	2	18	north wind	955	30	0	0
2021-04-09 21:00:00	17.6	33	1	1	7	north wind	955	14.9	0	0
2021-04-09 22:00:00	15.3	43	1.1	1	129	southeast wind	956	11.1	0	35
2021-04-09 23:00:00	13.6	52	1.1	1	263	west wind	956	15.2	0	58
2021-04-10 00:00:00	11.4	60	1.3	1	201	south wind	956	10.4	0	65
2021-04-10 01:00:00	11	64	1.1	1	289	west wind	956	20.8	0	0
2021-04-10 02:00:00	10.1	64	1.1	1	327	northwest wind	957	20.6	0	0
2021-04-10 03:00:00	8.6	72	1.8	2	256	west wind	956	24.4	0	0
2021-04-10 04:00:00	8	72	1.5	1	262	west wind	956	27.2	0	0
2021-04-10 05:00:00	7.8	71	2.2	2	250	west wind	956	30	0	34
2021-04-10 06:00:00	7.4	70	2.7	2	252	west wind	956	30	0	53
2021-04-10 07:00:00	7.2	68	2.3	2	257	west wind	956	30	0	21
2021-04-10 08:00:00	7.7	64	2.7	2	255	west wind	956	24.3	0	0
2021-04-10 09:00:00	9.3	59	2.4	2	253	west wind	956	30	0	0
2021-04-10 10:00:00	12.4	50	1.8	2	258	west wind	956	21.6	0	0
2021-04-10 11:00:00	15.8	40	0.9	1	325	northwest wind	956	30	0	0
2021-04-10 12:00:00	18.1	30	1.8	2	101	east wind	956	30	0	0
2021-04-10 13:00:00	19.4	28	2.4	2	110	east wind	955	30	0	0
2021-04-10 14:00:00	20.3	26	2	2	156	southeast wind	955	30	0	0

2021-04-10 15:00:00	20.9	24	2	2	42	northeast wind	954	30	0	0
2021-04-10 16:00:00	21.5	22	1.9	2	161	south wind	954	30	0	0
2021-04-10 17:00:00	21.9	23	2.5	2	119	southeast wind	953	26.7	0	0
2021-04-10 18:00:00	21.9	25	2.9	2	119	southeast wind	953	30	0	0
2021-04-10 19:00:00	21.5	25	2.1	2	94	east wind	953	22.1	0	0
2021-04-10 20:00:00	20.1	28	3.6	3	122	southeast wind	953	26.3	0	0
2021-04-10 21:00:00	18.5	32	2	2	87	east wind	954	30	0	0
2021-04-10 22:00:00	16.4	37	1.2	1	119	southeast wind	955	11.2	0	0
2021-04-10 23:00:00	14	48	1.3	1	247	southwest wind	955	18.7	0	0
2021-04-11 00:00:00	13.4	43	1	1	69	east wind	955	25.7	0	82
2021-04-11 01:00:00	12.9	43	1	1	316	northwest wind	956	26	0	0
2021-04-11 02:00:00	11.2	48	1.3	1	310	northwest wind	956	23.3	0	41
2021-04-11 03:00:00	9.7	58	1.4	1	245	southwest wind	956	21.7	0	0
2021-04-11 04:00:00	9.4	65	1.1	1	204	southwest wind	956	21.8	0	26
2021-04-11 05:00:00	9	68	1.3	1	20	north wind	957	23.2	0	31
2021-04-11 06:00:00	7.8	70	1.8	2	339	north wind	957	23.2	0	0
2021-04-11 07:00:00	6.1	78	0.6	1	270	west wind	958	18	0	0
2021-04-11 08:00:00	5.5	79	0.7	1	268	west wind	958	13.3	0	0
2021-04-11 09:00:00	8.2	69	1.2	1	250	west wind	959	21.3	0	0
2021-04-11 10:00:00	12.5	37	1.3	1	236	southwest wind	958	30	0	0
2021-04-11 11:00:00	14.9	22	1.2	1	113	southeast wind	958	30	0	0
2021-04-11 12:00:00	15.9	22	1.6	2	154	southeast wind	958	30	0	0
2021-04-11 13:00:00	17.2	21	2.3	2	81	east wind	958	30	0	0
2021-04-11 14:00:00	17.7	21	1.3	1	80	east wind	957	30	0	0
2021-04-11 15:00:00	18.4	19	2.9	2	78	east wind	957	30	0	0
2021-04-11 16:00:00	18.7	18	2.3	2	68	east wind	957	29	0	0
2021-04-11 17:00:00	19.1	19	3	2	50	northeast wind	957	23.7	0	0
2021-04-11 18:00:00	19.1	19	2.9	2	82	east wind	957	27.6	0	0
2021-04-11 19:00:00	18.8	19	3.3	2	62	northeast wind	958	30	0	0
2021-04-11 20:00:00	17.7	17	3.5	3	67	northeast wind	959	23.9	0	0
2021-04-11 21:00:00	16.4	21	1.7	2	55	northeast wind	960	18.9	0	0
2021-04-11 22:00:00	15.4	23	3.1	2	63	northeast wind	961	17.4	0	0
2021-04-11 23:00:00	14.4	23	1.8	2	54	northeast wind	962	22	0	0
2021-04-12 00:00:00	12.5	26	1.2	1	334	northwest wind	962	18.3	0	0
2021-04-12 01:00:00	10.6	33	1.1	1	275	west wind	963	22.3	0	91
2021-04-12 02:00:00	8.7	40	0.9	1	260	west wind	963	23.8	0	0
2021-04-12 03:00:00	7.1	46	2	2	265	west wind	963	21.9	0	0
2021-04-12 04:00:00	5.8	56	2.3	2	276	west wind	963	30	0	0

2021-04-12 05:00:00	5.9	50	3.1	2	269	west wind	963	30	0	0
2021-04-12 06:00:00	5.8	49	1.5	1	279	west wind	964	30	0	0
2021-04-12 07:00:00	5	53	2.6	2	257	west wind	964	14.6	0	0
2021-04-12 08:00:00	4.9	58	2.1	2	250	west wind	964	22.5	0	0
2021-04-12 09:00:00	6.8	55	2.2	2	260	west wind	964	18.2	0	0
2021-04-12 10:00:00	11.5	32	1.1	1	220	southwest wind	964	30	0	0
2021-04-12 11:00:00	13.5	17	3.2	2	15	north wind	964	30	0	0
2021-04-12 12:00:00	13.9	17	3.8	3	27	northeast wind	964	30	0	0
2021-04-12 13:00:00	14.7	18	2.6	2	333	northwest wind	964	30	0	0
2021-04-12 14:00:00	16.1	18	2.5	2	7	north wind	963	23.9	0	0
2021-04-12 15:00:00	17.4	17	2.1	2	59	northeast wind	963	22.2	0	0
2021-04-12 16:00:00	17.7	14	3.4	3	44	northeast wind	963	27.4	0	0
2021-04-12 17:00:00	17.7	15	2.8	2	44	northeast wind	962	26.9	0	0
2021-04-12 18:00:00	17.6	15	2.5	2	93	east wind	962	25.2	0	0
2021-04-12 19:00:00	17.4	16	2.5	2	102	east wind	962	27.5	0	0
2021-04-12 20:00:00	16.9	16	1.1	1	82	east wind	962	18	0	0
2021-04-12 21:00:00	15.6	17	1.3	1	66	northeast wind	962	12.4	0	0
2021-04-12 22:00:00	12.3	26	0.8	1	299	northwest wind	963	7.9	0	0
2021-04-12 23:00:00	11.5	36	1	1	268	west wind	963	7.9	0	0
2021-04-13 00:00:00	11.5	36	1	1	268	west wind	963	7.9	0	0
2021-04-13 01:00:00	11.5	36	1	1	268	west wind	963	7.9	0	0
2021-04-13 02:00:00	8.4	40	3.4	3	350	north wind		7.9	0	0
2021-04-13 03:00:00	7.8	40	3.4	3	349	north wind		29	0	0
2021-04-13 04:00:00	6.6	47	2.8	2	257	west wind	962	27.5	0	0
2021-04-13 05:00:00	6.1	50	2.4	2	259	west wind	962	30	0	0
2021-04-13 06:00:00	5.6	51	2.6	2	256	west wind	962	29.5	0	0
2021-04-13 07:00:00	5	53	2	2	258	west wind	961	25.2	0	0
2021-04-13 08:00:00	5.7	48	1.2	1	280	west wind	961	22.7	0	0
2021-04-13 09:00:00	8.4	39	2.1	2	270	west wind	962	24	0	0
2021-04-13 10:00:00	12.1	28	1	1	328	northwest wind	962	20.7	0	0
2021-04-13 11:00:00	14.4	22	2.3	2	33	northeast wind	962	25.1	0	0
2021-04-13 12:00:00	14.8	18	2.4	2	23	northeast wind	961	28.2	0	0
2021-04-13 13:00:00	16.1	17	2.1	2	82	east wind	961	28.1	0	0
2021-04-13 14:00:00	17.1	17	2.3	2	62	northeast wind	960	28.4	0	0
2021-04-13 15:00:00	17.8	14	2.8	2	50	northeast wind	959	25.3	0	0
2021-04-13 16:00:00	18.9	14	2.3	2	80	east wind	958	24.1	0	0
2021-04-13 17:00:00	19.8	13	1.3	1	325	northwest wind	958	23.3	0	0
2021-04-13 18:00:00	19.8	12	1.1	1	153	southeast wind	957	26.8	0	0

2021-04-13 19:00:00	19.7	14	1	1	118	southeast wind	957	23.3	0	0
2021-04-13 20:00:00	19	14	0.9	1	3	north wind	957	16.6	0	0
2021-04-13 21:00:00	16.4	21	0.7	1	41	northeast wind	958	12.3	0	0
2021-04-13 22:00:00	13.3	33	0	0	0	north wind	958	7.2	0	0
2021-04-13 23:00:00	11.2	36	1.5	1	272	west wind	959	10.5	0	0
2021-04-14 00:00:00	9.4	44	1.5	1	231	southwest wind	959	15.1	0	0
2021-04-14 01:00:00	9.9	47	2.3	2	259	west wind	960	19.8	0	0
2021-04-14 02:00:00	9.7	47	3.4	3	267	west wind	960	26.9	0	23
2021-04-14 03:00:00	8.2	53	1.9	2	256	west wind	960	24.3	0	42
2021-04-14 04:00:00	7.4	56	1.5	1	257	west wind	960	24.6	0	28
2021-04-14 05:00:00	7.2	57	2.1	2	255	west wind	960	24.6	0	0
2021-04-14 06:00:00	7.1	57	3.6	3	259	west wind	960	28.4	0	0
2021-04-14 07:00:00	6.9	54	3.1	2	254	west wind	960	26	0	0
2021-04-14 08:00:00	7	54	2.6	2	259	west wind	960	27.5	0	0
2021-04-14 09:00:00	10.4	41	3	2	254	west wind	961	21	0	0
2021-04-14 10:00:00	14	34	1.9	2	269	west wind	961	29.3	0	0
2021-04-14 11:00:00	17.5	24	1.4	1	2	north wind	960	25	0	0
2021-04-14 12:00:00	19.2	18	2.1	2	49	northeast wind	960	20.1	0	0
2021-04-14 13:00:00	19.9	18	2.8	2	81	east wind	960	22.7	0	0
2021-04-14 14:00:00	20.5	17	2.7	2	71	east wind	959	22.2	0	0
2021-04-14 15:00:00	22	15	2.3	2	95	east wind	958	26.5	0	0
2021-04-14 16:00:00	22.8	15	2.5	2	104	east wind	958	22.8	0	0
2021-04-14 17:00:00	22.6	12	3.1	2	78	east wind	957	23.1	0	0
2021-04-14 18:00:00	22.9	13	3.8	3	140	southeast wind	957	18.3	0	0
2021-04-14 19:00:00	22.2	10	2.8	2	114	southeast wind	957	15.4	0	0
2021-04-14 20:00:00	20.9	10	3.1	2	103	east wind	957	19.6	0	0
2021-04-14 21:00:00	18.6	13	1.2	1	104	east wind	957	11.6	0	0
2021-04-14 22:00:00	15.4	18	0.8	1	264	west wind	957	11.2	0	0
2021-04-14 23:00:00	12.6	28	1	1	262	west wind	958	11	0	0
2021-04-15 00:00:00	9.3	39	1.3	1	261	west wind	958	12.7	0	0
2021-04-15 01:00:00	9.3	38	1.8	2	271	west wind	958	19	0	81
2021-04-15 02:00:00	9.2	39	2.1	2	265	west wind	958	22.7	0	0
2021-04-15 03:00:00	8.3	43	2	2	264	west wind	958	22.4	0	0
2021-04-15 04:00:00	7.9	45	1.5	1	275	west wind	958	23.8	0	0
2021-04-15 05:00:00	7.9	45	1.5	1	275	west wind	958	20.9	0	0
2021-04-15 06:00:00	7.9	45	1.5	1	275	west wind	958	24.9	0	0
2021-04-15 07:00:00	4.3	56	1.2	1	306	northwest wind	959	15.6	0	0
2021-04-15 08:00:00	4.1	58	1	1	267	west wind	959	13.2	0	0

2021-04-15 09:00:00	7.5	47	1.2	1	270	west wind	960	17.4	0	0
2021-04-15 10:00:00	13.3	19	1.3	1	235	southwest wind	960	21.6	0	0
2021-04-15 11:00:00	14.7	15	1.4	1	107	east wind	959	21.5	0	0
2021-04-15 12:00:00	15.9	15	1.9	2	23	northeast wind	959	18	0	0
2021-04-15 13:00:00	16	14	3.1	2	71	east wind	959	23.6	0	0
2021-04-15 14:00:00	17.1	16	3	2	98	east wind	958	23.4	0	0
2021-04-15 15:00:00	17.8	14	2.9	2	42	northeast wind	957	24.2	0	0
2021-04-15 16:00:00	18.3	15	2.4	2	93	east wind	956	19.3	0	0
2021-04-15 17:00:00	18.7	13	2.5	2	44	northeast wind	956	20.7	0	0
2021-04-15 18:00:00	19	13	2.8	2	64	northeast wind	956	22.5	0	0
2021-04-15 19:00:00	18.7	14	1.9	2	42	northeast wind	956	17.6	0	0
2021-04-15 20:00:00	18.1	16	1.3	1	38	northeast wind	956	14	0	0
2021-04-15 21:00:00	16.2	20	1.1	1	341	north wind	956	10.1	0	0
2021-04-15 22:00:00	13.4	28	1.1	1	295	northwest wind	956	8.2	0	0
2021-04-15 23:00:00	10.9	35	1.4	1	261	west wind	956	14.1	0	0
2021-04-16 00:00:00	10.4	35	2.9	2	255	west wind	956	18.1	0	0
2021-04-16 01:00:00	9.8	39	2.4	2	254	west wind	956	20.8	0	0
2021-04-16 02:00:00	10.1	38	3.3	2	258	west wind	956	21	0	0
2021-04-16 03:00:00	9.3	40	2.4	2	258	west wind	956	21.5	0	0
2021-04-16 04:00:00	9.1	40	2.6	2	258	west wind	956	23.7	0	0
2021-04-16 05:00:00	8.9	41	2.6	2	262	west wind	955	22	0	0
2021-04-16 06:00:00	8.4	41	1.4	1	260	west wind	956	21.6	0	0
2021-04-16 07:00:00	7.5	44	2.3	2	255	west wind	956	18.4	0	0
2021-04-16 08:00:00	7	47	2	2	260	west wind	956	13.4	0	0
2021-04-16 09:00:00	9.7	42	2.6	2	253	west wind	957	15	0	0
2021-04-16 10:00:00	13.8	31	2.5	2	254	west wind	957	17.4	0	0
2021-04-16 11:00:00	16.9	21	1.5	1	346	north wind	956	16.7	0	0
2021-04-16 12:00:00	18	20	1.2	1	36	northeast wind	956	16.5	0	0
2021-04-16 13:00:00	18.8	20	2.3	2	95	east wind	956	12.4	0	0
2021-04-16 14:00:00	19.2	18	3.5	3	74	east wind	955	18.4	0	0
2021-04-16 15:00:00	20.2	15	2.7	2	66	northeast wind	954	17	0	0
2021-04-16 16:00:00	21.4	16	1.7	2	136	southeast wind	954	22.9	0	46
2021-04-16 17:00:00	21.5	13	2.2	2	38	northeast wind	953	23.6	0	0
2021-04-16 18:00:00	21.2	12	2.4	2	39	northeast wind	953	30	0	0
2021-04-16 19:00:00	20.9	12	1.8	2	84	east wind	953	24.9	0	0
2021-04-16 20:00:00	20.2	12	1.2	1	53	northeast wind	953	18.2	0	0
2021-04-16 21:00:00	17.6	19	0.8	1	349	north wind	953	8.9	0	0
2021-04-16 22:00:00	15.1	28	0.9	1	272	west wind	953	12.9	0	0

2021-04-16 23:00:00	12	38	1	1	239	southwest wind	953	9.5	0	29
2021-04-17 00:00:00	11.2	39	1.5	1	226	southwest wind	953	16.9	0	73
2021-04-17 01:00:00	11.7	38	2.2	2	258	west wind	953	20.2	0	73
2021-04-17 02:00:00	12.1	37	2.8	2	262	west wind	953	20.2	0	89
2021-04-17 04:00:00	12.9	38	2.1	2	280	west wind	952	20.7	0	93
2021-04-17 05:00:00	11.6	42	1.5	1	266	west wind	952	20.9	0	93
2021-04-17 06:00:00	11	45	2	2	255	west wind	952	20.3	0	94
2021-04-17 07:00:00	10.9	46	2.5	2	258	west wind	952	19.8	0	75
2021-04-17 08:00:00	10.2	49	2.1	2	257	west wind	953	17.1	0	90
2021-04-17 09:00:00	11.3	47	2.9	2	257	west wind	953	13.6	0	91
2021-04-17 10:00:00	13.3	42	2.7	2	261	west wind	953	10.1	0	52
2021-04-17 11:00:00	18.4	26	0.9	1	153	southeast wind	953	19.3	0	0
2021-04-17 12:00:00	19.9	18	1.3	1	41	northeast wind	953	19.2	0	0
2021-04-17 13:00:00	19.7	19	2.7	2	222	southwest wind	954	21.6	0	88
2021-04-17 14:00:00	17.4	38	2.1	2	76	east wind	954	25.1	0	94
2021-04-17 15:00:00	19.7	31	3	2	343	north wind	954	28.3	0	91
2021-04-17 16:00:00	19.7	32	2.7	2	37	northeast wind	954	30	0	0
2021-04-17 17:00:00	19.1	34	2	2	113	southeast wind	953	24.1	0	87
2021-04-17 18:00:00	20.1	30	2.6	2	150	southeast wind	953	30	0	45
2021-04-17 19:00:00	20.4	32	1.5	1	167	south wind	953	30	0	45
2021-04-17 20:00:00	19.8	32	0.9	1	27	northeast wind	953	21.1	0	76
2021-04-17 21:00:00	18.6	34	2.5	2	85	east wind	953	26.9	0	86
2021-04-17 22:00:00	18	37	2.8	2	93	east wind	954	25.4	0	76
2021-04-17 23:00:00	16.4	41	2.4	2	47	northeast wind	955	24.1	0	79
2021-04-18 00:00:00	15.9	45	1.6	2	170	south wind	955	23.5	0	76
2021-04-18 01:00:00	14.8	46	0.7	1	260	west wind	955	22.9	0	76
2021-04-18 02:00:00	12.3	57	1	1	197	south wind	955	20.1	0	0
2021-04-18 03:00:00	10.5	63	0.5	1	351	north wind	955	20.5	0	23
2021-04-18 04:00:00	9.6	67	1.2	1	281	west wind	955	21.4	0	0
2021-04-18 05:00:00	8.1	73	1.5	1	250	west wind	955	22.3	0	0
2021-04-18 06:00:00	8	72	1.9	2	263	west wind	955	23.2	0	0
2021-04-18 07:00:00	7.7	73	2.1	2	272	west wind	955	22.7	0	0
2021-04-18 08:00:00	7.9	72	2.4	2	254	west wind	955	19.9	0	0
2021-04-18 09:00:00	10.3	65	1.6	2	256	west wind	956	19.5	0	0
2021-04-18 10:00:00	14.2	52	1.9	2	244	southwest wind	956	22.2	0	0
2021-04-18 11:00:00	17.8	42	1.4	1	263	west wind	956	30	0	0
2021-04-18 12:00:00	19.5	36	1.8	2	105	east wind	955	28	0	0
2021-04-18 13:00:00	21.1	32	1.1	1	125	southeast wind	955	29.9	0	0

2021-04-18 14:00:00	21.6	25	2.4	2	63	northeast wind	954	30	0	66
2021-04-18 15:00:00	22.3	25	2.6	2	107	east wind	953	30	0	0
2021-04-18 16:00:00	23.3	24	2	2	122	southeast wind	953	26.4	0	0
2021-04-18 17:00:00	23.4	23	1.9	2	82	east wind	952	28.7	0	0
2021-04-18 18:00:00	23.2	22	2.3	2	73	east wind	952	23.5	0	0
2021-04-18 19:00:00	23	22	2.3	2	73	east wind	952	21.4	0	42
2021-04-18 20:00:00	22.2	23	2.3	2	73	east wind	952	20.8	0	0
2021-04-18 21:00:00	20.8	26	1.8	2	73	east wind	952	22.4	0	0
2021-04-18 22:00:00	18.9	31	1.4	1	51	northeast wind	953	17.3	0	73
2021-04-18 23:00:00	16.2	40	0.8	1	301	northwest wind	953	12.6	0	68
2021-04-19 00:00:00	14.5	45	1.4	1	246	southwest wind	953	16.7	0	90
2021-04-19 01:00:00	13.1	50	0.9	1	259	west wind	953	23.5	0	90
2021-04-19 02:00:00	13.4	51	1	1	264	west wind	953	28.6	0	77
2021-04-19 03:00:00	14	49	2.6	2	256	west wind	953	29.6	0	85
2021-04-19 04:00:00	13.6	52	1.8	2	266	west wind	952	29.8	0	76
2021-04-19 05:00:00	13	54	2.5	2	258	west wind	953	29.4	0	92
2021-04-19 06:00:00	12.9	53	2.1	2	257	west wind	952	29.7	0	85
2021-04-19 07:00:00	12.8	53	2.5	2	269	west wind	952	21.3	0	49
2021-04-19 08:00:00	12.3	56	1.3	1	271	west wind	953	25.5	0	86
2021-04-19 09:00:00	13.1	54	2	2	250	west wind	954	20.8	0	90
2021-04-19 10:00:00	14.4	52	1.1	1	116	southeast wind	954	23.8	0	91
2021-04-19 11:00:00	16.8	43	3.4	3	70	east wind	954	21.3	0	93
2021-04-19 12:00:00	16.9	44	3.8	3	81	east wind	955	26.6	0	93
2021-04-19 13:00:00	17.4	40	3.8	3	89	east wind	954	30	0	93
2021-04-19 14:00:00	18.4	40	3	2	113	southeast wind	954	30	0	93
2021-04-19 15:00:00	19.7	36	3.3	2	122	southeast wind	953	30	0	91
2021-04-19 16:00:00	19.8	35	3.1	2	56	northeast wind	952	29.6	0	61
2021-04-19 17:00:00	19.1	34	2.7	2	84	east wind	951	27.7	0	0
2021-04-19 18:00:00	19	36	1.4	1	115	southeast wind	951	28.1	0	37
2021-04-19 19:00:00	18.3	40	2.7	2	212	southwest wind	953	30	0	87
2021-04-19 20:00:00	13.9	55	4.7	3	333	northwest wind	953	30	0	82
2021-04-19 21:00:00	13.2	59	1.9	2	11	north wind	953	30	0	0
2021-04-19 22:00:00	12.3	63	1.6	2	254	west wind	953	26.9	0	0
2021-04-19 23:00:00	11.3	70	2.1	2	273	west wind	953	30	0	0
2021-04-20 00:00:00	10.1	77	1.7	2	263	west wind	953	30	0	0
2021-04-20 01:00:00	9.4	81	2	2	261	west wind	952	30	0	0
2021-04-20 02:00:00	9.3	81	2.2	2	261	west wind	952	30	0	29
2021-04-20 03:00:00	9.5	78	2.6	2	253	west wind	951	30	0	74

2021-04-20 04:00:00	9.1	79	2.4	2	262	west wind	950	30	0	72
2021-04-20 05:00:00	8.7	80	1.6	2	260	west wind	950	30	0	37
2021-04-20 06:00:00	8.8	78	1.4	1	237	southwest wind	949	30	0	36
2021-04-20 07:00:00	8.5	78	2.4	2	265	west wind	949	30	0	0
2021-04-20 08:00:00	9.4	72	1.9	2	267	west wind	949	30	0	29
2021-04-20 09:00:00	11.3	63	2.7	2	276	west wind	949	30	0	0
2021-04-20 10:00:00	13.9	54	0.6	1	290	west wind	948	30	0	63
2021-04-20 11:00:00	16.1	47	1.5	1	128	southeast wind	948	28	0	69
2021-04-20 12:00:00	18.7	39	1.5	1	76	east wind	947	29.9	0	63
2021-04-20 13:00:00	20.3	35	1.8	2	66	northeast wind	947	30	0	0
2021-04-20 14:00:00	21.2	33	2.1	2	67	northeast wind	946	27.5	0	56
2021-04-20 15:00:00	21.7	29	2.4	2	96	east wind	945	27.4	0	89
2021-04-20 16:00:00	22.4	27	3.5	3	63	northeast wind	945	26.2	0	60
2021-04-20 17:00:00	22.2	28	2.8	2	82	east wind	944	26.4	0	92
2021-04-20 18:00:00	22	28	2.6	2	74	east wind	944	24.1	0	70
2021-04-20 19:00:00	21.8	29	2.3	2	118	southeast wind	944	24.5	0	76
2021-04-20 20:00:00	20.4	32	1.6	2	355	north wind	944	19.5	0	41
2021-04-20 21:00:00	18.1	40	1.1	1	253	west wind	945	7.7	0	80
2021-04-20 22:00:00	15.9	47	5.1	3	125	southeast wind	946	5.5	0	79
2021-04-20 23:00:00	14.3	55	4.3	3	117	southeast wind	946	11.3	0	85
2021-04-21 00:00:00	13.6	56	1.2	1	8	north wind	946	15.6	0	86
2021-04-21 01:00:00	12.9	58	2	2	27	northeast wind	946	18.8	0	0
2021-04-21 02:00:00	11.7	59	1.8	2	64	northeast wind	947	22.4	0	90
2021-04-21 03:00:00	10.8	63	1.2	1	220	southwest wind	946	16.6	0	92
2021-04-21 04:00:00	9.8	68	1.5	1	290	west wind	946	22	0	90
2021-04-21 05:00:00	8.9	71	1.5	1	257	west wind	945	19.3	0	0
2021-04-21 06:00:00	8.2	75	1.5	1	251	west wind	945	13.8	0	29
2021-04-21 07:00:00	7.8	80	1.3	1	272	west wind	945	15.4	0	0
2021-04-21 08:00:00	8.5	77	1.1	1	96	east wind	946	19.4	0	0
2021-04-21 09:00:00	11.2	66	1.8	2	121	southeast wind	946	20.4	0	36
2021-04-21 10:00:00	11.1	64	2.2	2	107	east wind	946	21.2	0	77
2021-04-21 11:00:00	12.4	59	2	2	108	east wind	946	24.4	0	78
2021-04-21 12:00:00	13.7	53	2.9	2	97	east wind	946	26.7	0	66
2021-04-21 13:00:00	14.4	50	3	2	75	east wind	946	24.2	0	89
2021-04-21 14:00:00	15	49	3	2	106	east wind	945	21.1	0	89
2021-04-21 15:00:00	15.6	49	3.2	2	112	east wind	945	18.4	0	92
2021-04-21 16:00:00	15.3	47	3.8	3	82	east wind	945	20	0	59
2021-04-21 17:00:00	15.3	47	3.9	3	99	east wind	945	14.3	0	75

2021-04-21 18:00:00	13.3	52	4.4	3	133	southeast wind	947	15	0	75
2021-04-21 19:00:00	11.6	61	4.2	3	133	southeast wind	948	6.4	0	86
2021-04-21 20:00:00	9	67	3.1	2	99	east wind	950	6.3	0	85
2021-04-21 21:00:00	7.3	77	3.6	3	82	east wind	952	28.5	0.8	75
2021-04-21 22:00:00	6.9	77	3.1	2	99	east wind	954	30	0	79
2021-04-21 23:00:00	6.5	73	2.9	2	112	east wind	955	11.9	0	88
2021-04-22 00:00:00	5.1	70	5.6	4	127	southeast wind	956	12.4	0	91
2021-04-22 01:00:00	3.6	72	4.6	3	112	east wind	957	8	0	92
2021-04-22 02:00:00	2.5	80	3.4	3	98	east wind	958	25.6	0.9	55
2021-04-22 03:00:00	2.3	81	2.7	2	130	southeast wind	959	30	1.8	74
2021-04-22 04:00:00	2.6	72	2.9	2	146	southeast wind	959	20.2	0.4	65
2021-04-22 05:00:00	1.9	82	3	2	152	southeast wind	960	30	0	71
2021-04-22 06:00:00	2.5	83	2.6	2	113	southeast wind	961	30	0.3	54
2021-04-22 07:00:00	2.3	67	1.8	2	157	southeast wind	961	30	0	65
2021-04-22 08:00:00	3.1	52	0.7	1	151	southeast wind	962	30	0	73
2021-04-22 09:00:00	3.2	59	2.5	2	116	southeast wind	963	30	0	76
2021-04-22 10:00:00	2.9	75	1.7	2	100	east wind	963	30	0	64
2021-04-22 11:00:00	1.9	87	1.7	2	154	southeast wind	964	30	0	95
2021-04-22 12:00:00	2.6	88	1.1	1	220	southwest wind	964	30	0	94
2021-04-22 13:00:00	4.2	79	1.2	1	148	southeast wind	964	30	0	94
2021-04-22 14:00:00	5.9	67	1.5	1	295	northwest wind	963	30	0	94
2021-04-22 15:00:00	6.1	52	2	2	168	south wind	963	30	0	95
2021-04-22 16:00:00	6.6	52	2.3	2	266	west wind	964	30	0	94
2021-04-22 17:00:00	6.2	59	2.7	2	274	west wind	964	30	0	95
2021-04-22 18:00:00	5.7	61	3.1	2	295	northwest wind	964	23.9	0	95
2021-04-22 19:00:00	4.6	74	2.5	2	267	west wind	965	30	0	84
2021-04-22 20:00:00	4.3	79	2.3	2	287	west wind	966	25.6	0	76
2021-04-22 21:00:00	2.9	90	1.9	2	329	northwest wind	967	7.7	0	55
2021-04-22 22:00:00	1.9	94	1.6	2	331	northwest wind	968	8.4	0.4	69
2021-04-22 23:00:00	1.4	95	0.6	1	266	west wind	968	16.3	1.3	86
2021-04-23 00:00:00	1.7	97	1.6	2	341	north wind	968	8.3	1.8	92
2021-04-23 01:00:00	1.8	97	1.2	1	322	northwest wind	968	10.6	0	92
2021-04-23 02:00:00	1.6	97	3.3	2	125	southeast wind	968	5.3	2	69
2021-04-23 03:00:00	0.8	98	0.8	1	55	northeast wind	968	4.1	0.7	89
2021-04-23 04:00:00	0.6	99	2.4	2	128	southeast wind	968	16.7	0.9	80
2021-04-23 05:00:00	0.7	98	3.6	3	125	southeast wind	968	23	0.2	84
2021-04-23 06:00:00	0.3	94	2.5	2	123	southeast wind	968	23.7	0.1	68
2021-04-23 07:00:00	-0.3	93	1.5	1	124	southeast wind	969	30	0	83

2021-04-23 08:00:00	-0.1	77	1	1	105	east wind	969	30	0	35
2021-04-23 09:00:00	0.3	75	3	2	132	southeast wind	969	30	0	52
2021-04-23 10:00:00	1.2	70	1.9	2	113	southeast wind	970	29.1	0	87
2021-04-23 11:00:00	2.1	72	1.7	2	89	east wind	970	23.9	0	68
2021-04-23 12:00:00	2.5	70	1.9	2	323	northwest wind	970	27.8	0	61
2021-04-23 13:00:00	2.9	69	1.2	1	64	northeast wind	970	30	0	75
2021-04-23 14:00:00	3.7	64	1.6	2	115	southeast wind	969	30	0	66
2021-04-23 15:00:00	4.1	62	2.5	2	89	east wind	969	30	0	89
2021-04-23 16:00:00	2.7	72	2.9	2	14	north wind	969	3.1	0	86
2021-04-23 17:00:00	2	84	2.6	2	16	north wind	969	24.3	0	97
2021-04-23 18:00:00	2	76	2.6	2	330	northwest wind	969	21.4	0.2	78
2021-04-23 19:00:00	1.7	83	3	2	296	northwest wind	970	30	0	55
2021-04-23 20:00:00	1.8	79	1.7	2	313	northwest wind	970	30	0	58
2021-04-23 21:00:00	2.5	67	1.5	1	276	west wind	971	30	0	63
2021-04-23 22:00:00	2	71	2.4	2	319	northwest wind	972	25.4	0	74
2021-04-23 23:00:00	1.8	71	0.7	1	314	northwest wind	972	30	0	81
2021-04-24 00:00:00	1.3	78	1.2	1	329	northwest wind	972	30	0	70
2021-04-24 01:00:00	1.4	72	0.4	1	349	north wind	971	24	0	88
2021-04-24 02:00:00	0.9	84	1.1	1	198	south wind	971	23	0	68
2021-04-24 03:00:00	0.7	82	1.3	1	254	west wind	970	20.8	0	81
2021-04-24 04:00:00	0.5	82	1.8	2	269	west wind	969	28.3	0	87
2021-04-24 05:00:00	0.4	86	1.8	2	259	west wind	969	27.9	0	73
2021-04-24 06:00:00	0.1	85	1.9	2	252	west wind	969	30	0	62
2021-04-24 07:00:00	-0.3	82	1.9	2	251	west wind	969	29.2	0	51
2021-04-24 08:00:00	-0.4	76	2.1	2	252	west wind	969	30	0	40
2021-04-24 09:00:00	1.1	63	3	2	258	west wind	969	30	0	0
2021-04-24 10:00:00	2.8	58	3.3	2	260	west wind	969	30	0	0
2021-04-24 11:00:00	4.2	48	2.8	2	253	west wind	969	30	0	0
2021-04-24 12:00:00	5.4	41	2.5	2	245	southwest wind	968	30	0	0
2021-04-24 13:00:00	6.3	33	1.9	2	255	west wind	968	30	0	0
2021-04-24 14:00:00	6.9	31	1.7	2	46	northeast wind	967	30	0	0
2021-04-24 15:00:00	8.2	25	1.2	1	225	southwest wind	967	30	0	0
2021-04-24 16:00:00	8.8	21	1.5	1	223	southwest wind	967	30	0	0
2021-04-24 17:00:00	9.1	21	1.8	2	331	northwest wind	966	30	0	0
2021-04-24 18:00:00	9.2	21	1.7	2	308	northwest wind	967	30	0	0
2021-04-24 19:00:00	9.4	20	1.5	1	224	southwest wind	967	30	0	0
2021-04-24 20:00:00	9.2	22	0.9	1	5	north wind	968	30	0	0
2021-04-24 21:00:00	7.2	28	0.5	1	299	northwest wind	968	21.7	0	0

2021-04-24 22:00:00	4.9	46	0.4	1	268	west wind	969	12.3	0	0
2021-04-24 23:00:00	3.1	58	0.4	1	343	north wind	969	12.4	0	25
2021-04-25 00:00:00	1.7	64	1	1	264	west wind	969	16.1	0	0
2021-04-25 01:00:00	0.8	66	1.4	1	262	west wind	968	24.1	0	0
2021-04-25 02:00:00	0.9	72	1.6	2	257	west wind	968	30	0	25
2021-04-25 03:00:00	0.8	71	1.8	2	263	west wind	967	30	0	24
2021-04-25 04:00:00	0.5	71	1.7	2	264	west wind	967	30	0	23
2021-04-25 05:00:00	0.3	71	1.4	1	250	west wind	967	30	0	28
2021-04-25 06:00:00	-0.2	75	1.5	1	264	west wind	967	30	0	26
2021-04-25 07:00:00	0	75	1.5	1	264	west wind	967	28.7	0	92
2021-04-25 08:00:00	0.7	73	1.4	1	262	west wind	967	23.9	0	84
2021-04-25 09:00:00	1.7	70	1.8	2	255	west wind	968	25	0	90
2021-04-25 10:00:00	3.8	54	1.1	1	269	west wind	968	30	0	85
2021-04-25 11:00:00	7	37	1.1	1	75	east wind	967	30	0	83
2021-04-25 12:00:00	8.4	29	1.4	1	58	northeast wind	967	30	0	83
2021-04-25 13:00:00	9.7	25	2.1	2	134	southeast wind	966	30	0	0
2021-04-25 14:00:00	9.9	21	1.5	1	153	southeast wind	965	30	0	0
2021-04-25 15:00:00	10.8	19	1.7	2	99	east wind	965	30	0	0
2021-04-25 16:00:00	12	22	1.8	2	66	northeast wind	964	30	0	0
2021-04-25 17:00:00	11.6	21	2.6	2	68	east wind	963	30	0	46
2021-04-25 18:00:00	12.2	21	1.8	2	50	northeast wind	963	30	0	46
2021-04-25 19:00:00	11.8	21	2.1	2	84	east wind	963	30	0	71
2021-04-25 20:00:00	11.4	21	1.9	2	81	east wind	963	30	0	87
2021-04-25 21:00:00	10.9	23	1.4	1	86	east wind	963	29.3	0	77
2021-04-25 22:00:00	9.7	29	1.2	1	44	northeast wind	964	19.1	0	51
2021-04-25 23:00:00	7.4	41	0.9	1	354	north wind	964	19.7	0	49
2021-04-26 00:00:00	5.9	54	0.5	1	259	west wind	964	15.1	0	43
2021-04-26 01:00:00	4.9	55	1	1	273	west wind	964	16.8	0	89
2021-04-26 02:00:00	4.5	62	0.9	1	277	west wind	963	22.9	0	82
2021-04-26 03:00:00	4.2	64	1.4	1	252	west wind	963	23.1	0	92
2021-04-26 04:00:00	4.2	70	2.1	2	252	west wind	963	25.7	0	90
2021-04-26 05:00:00	4.5	67	2.1	2	261	west wind	963	26.7	0	91
2021-04-26 06:00:00	4.4	66	1.5	1	261	west wind	963	26.3	0	79
2021-04-26 07:00:00	3.6	72	1.5	1	273	west wind	964	17.3	0	29
2021-04-26 08:00:00	3.6	74	1.7	2	262	west wind	964	18.4	0	23
2021-04-26 09:00:00	5.3	66	2	2	261	west wind	964	24.9	0	0
2021-04-26 10:00:00	4	40	0.7	1	297	northwest wind	964	30	0	0
2021-04-26 11:00:00	11.4	28	1	1	88	east wind	964	30	0	0

2021-04-26 12:00:00	12.3	24	1	1	12	north wind	964	30	0	73
2021-04-26 13:00:00	13.1	26	3	2	69	east wind	963	30	0	0
2021-04-26 14:00:00	13.8	23	2.2	2	59	northeast wind	963	25.9	0	53
2021-04-26 15:00:00	14.6	23	1.6	2	90	east wind	963	23	0	0
2021-04-26 16:00:00	15.4	22	3	2	68	east wind	962	26.7	0	0
2021-04-26 17:00:00	15.9	20	2.5	2	57	northeast wind	962	27.3	0	0
2021-04-26 18:00:00	16.7	20	2.1	2	124	southeast wind	962	23.3	0	0
2021-04-26 19:00:00	16.8	20	2	2	72	east wind	962	26.9	0	0
2021-04-26 20:00:00	16.1	21	1.3	1	89	east wind	962	23.4	0	0
2021-04-26 21:00:00	15.3	23	1.5	1	37	northeast wind	963	10.2	0	0
2021-04-26 22:00:00	13.1	33	0.7	1	35	northeast wind	963	11	0	23
2021-04-26 23:00:00	10.7	48	0.8	1	259	west wind	963	15	0	38
2021-04-27 00:00:00	8.9	54	1.1	1	262	west wind	963	9.7	0	33
2021-04-27 01:00:00	7.6	60	1.2	1	287	west wind	963	18.7	0	0
2021-04-27 02:00:00	7.1	67	1	1	245	southwest wind	963	16	0	18
2021-04-27 03:00:00	6.7	70	1.3	1	265	west wind	963	17.5	0	0
2021-04-27 04:00:00	6.1	73	1.2	1	262	west wind	962	19.3	0	74
2021-04-27 05:00:00	5.7	76	1.4	1	258	west wind	963	19	0	0
2021-04-27 06:00:00	5.4	77	1.6	2	266	west wind	963	17.8	0	27
2021-04-27 07:00:00	4.6	80	1.4	1	265	west wind	963	15.8	0	0
2021-04-27 08:00:00	5.1	78	1.9	2	258	west wind	964	16.2	0	45
2021-04-27 09:00:00	8.1	66	1.5	1	255	west wind	965	20.2	0	0
2021-04-27 10:00:00	12.4	38	0.6	1	34	northeast wind	965	26	0	0
2021-04-27 11:00:00	14.7	25	2	2	70	east wind	964	23.7	0	0
2021-04-27 12:00:00	15.8	25	1.3	1	106	east wind	964	25.2	0	0
2021-04-27 13:00:00	17	23	1.5	1	63	northeast wind	963	27.5	0	0
2021-04-27 14:00:00	17.5	23	2.5	2	69	east wind	962	25.3	0	0
2021-04-27 15:00:00	18.9	22	2.4	2	63	northeast wind	962	23.2	0	0
2021-04-27 16:00:00	19.9	23	2.3	2	95	east wind	961	21.5	0	0
2021-04-27 17:00:00	20	21	2.2	2	50	northeast wind	960	27	0	0
2021-04-27 18:00:00	20	22	1.7	2	64	northeast wind	960	26.1	0	0
2021-04-27 19:00:00	20	21	1.3	1	38	northeast wind	960	18.6	0	0
2021-04-27 20:00:00	19.3	24	1.2	1	138	southeast wind	960	21.5	0	0
2021-04-27 21:00:00	17.4	33	0.7	1	325	northwest wind	960	12.3	0	30
2021-04-27 22:00:00	14.4	43	1.1	1	276	west wind	960	12.3	0	22
2021-04-27 23:00:00	12.7	47	1.3	1	269	west wind	960	13.8	0	20
2021-04-28 00:00:00	10.5	63	1	1	243	southwest wind	960	13.3	0	24
2021-04-28 01:00:00	11.1	56	2.2	2	265	west wind	960	16.8	0	0

2021-04-28 02:00:00	10.6	59	1.6	2	272	west wind	960	18.4	0	0
2021-04-28 03:00:00	10	63	2.1	2	266	west wind	960	18.1	0	42
2021-04-28 04:00:00	10	61	2.2	2	269	west wind	959	17.8	0	0
2021-04-28 05:00:00	9.5	63	2	2	247	southwest wind	959	16.2	0	30
2021-04-28 06:00:00	8.8	66	2.8	2	260	west wind	959	16.6	0	0
2021-04-28 07:00:00	8.6	66	2.7	2	263	west wind	959	16	0	0
2021-04-28 08:00:00	9.4	64	2.7	2	255	west wind	959	16.4	0	0
2021-04-28 09:00:00	12.1	57	2.3	2	262	west wind	959	17.5	0	0
2021-04-28 10:00:00	16	45	1.9	2	254	west wind	958	21	0	0
2021-04-28 11:00:00	19.5	33	0.8	1	282	west wind	958	21.3	0	0
2021-04-28 12:00:00	20.9	26	1.2	1	129	southeast wind	957	23	0	0
2021-04-28 13:00:00	21.9	23	2	2	131	southeast wind	957	23.3	0	0
2021-04-28 14:00:00	22.7	22	2.2	2	69	east wind	956	25.2	0	0
2021-04-28 15:00:00	23.5	21	2.6	2	98	east wind	955	22.3	0	0
2021-04-28 16:00:00	24.1	20	2	2	61	northeast wind	955	30	0	0
2021-04-28 17:00:00	24.7	20	1.6	2	30	northeast wind	954	23.1	0	0
2021-04-28 18:00:00	24.7	20	2	2	95	east wind	954	25.1	0	0
2021-04-28 19:00:00	24.5	20	1.4	1	62	northeast wind	954	23.5	0	0
2021-04-28 20:00:00	24.1	20	1.3	1	56	northeast wind	954	15.5	0	0
2021-04-28 21:00:00	21.5	30	0.9	1	357	north wind	954	14.6	0	0
2021-04-28 22:00:00	18.1	42	1.3	1	314	northwest wind	955	11.1	0	0
2021-04-28 23:00:00	15.8	48	1.6	2	263	west wind	955	14.4	0	0
2021-04-29 00:00:00	15.1	51	1.9	2	255	west wind	955	14.8	0	0
2021-04-29 01:00:00	14.4	54	1.9	2	254	west wind	955	16.9	0	0
2021-04-29 02:00:00	13.9	58	2.3	2	254	west wind	954	17.7	0	0
2021-04-29 03:00:00	13	59	1.2	1	258	west wind	954	18.7	0	0
2021-04-29 04:00:00	12.9	60	1.7	2	255	west wind	953	16.7	0	0
2021-04-29 05:00:00	12.2	64	1.9	2	263	west wind	953	19.5	0	0
2021-04-29 06:00:00	11.9	63	1.3	1	281	west wind	953	20.7	0	0
2021-04-29 07:00:00	11.2	64	1.9	2	265	west wind	953	22.3	0	0
2021-04-29 08:00:00	12.3	61	2	2	263	west wind	953	20	0	0
2021-04-29 09:00:00	15.3	53	2.8	2	257	west wind	954	22	0	0
2021-04-29 10:00:00	19.6	39	1.3	1	229	southwest wind	953	20.6	0	0
2021-04-29 11:00:00	23.6	28	0.7	1	67	northeast wind	953	20.2	0	0
2021-04-29 12:00:00	25.1	23	1.8	2	83	east wind	953	21.4	0	0
2021-04-29 13:00:00	26.2	21	1.8	2	116	southeast wind	952	22.5	0	0
2021-04-29 14:00:00	27.4	18	2.2	2	124	southeast wind	951	23.5	0	0
2021-04-29 15:00:00	28	19	2	2	48	northeast wind	951	22.9	0	0

2021-04-29 16:00:00	28.9	17	2.4	2	89	east wind	950	26.8	0	0
2021-04-29 17:00:00	29.3	16	2.2	2	83	east wind	949	26.3	0	0
2021-04-29 18:00:00	29.6	16	1.8	2	112	east wind	949	21.9	0	0
2021-04-29 19:00:00	29.4	15	2.1	2	77	east wind	948	26.8	0	0
2021-04-29 20:00:00	28.6	18	1.5	1	74	east wind	948	12.9	0	0
2021-04-29 21:00:00	25.2	27	1.3	1	346	north wind	948	11.7	0	0
2021-04-29 22:00:00	21.2	37	1.2	1	295	northwest wind	948	10.8	0	0
2021-04-29 23:00:00	19.1	41	1.6	2	260	west wind	949	17.8	0	0
2021-04-30 00:00:00	18.4	42	1.4	1	258	west wind	949	21.1	0	0
2021-04-30 01:00:00	18.6	42	2.8	2	259	west wind	949	23.3	0	0
2021-04-30 02:00:00	17.9	44	2.2	2	266	west wind	948	27.7	0	0
2021-04-30 03:00:00	16.9	46	2.3	2	252	west wind	948	25	0	0
2021-04-30 04:00:00	15.9	49	2.7	2	255	west wind	948	25.7	0	0
2021-04-30 05:00:00	15.7	49	2.4	2	253	west wind	948	28.6	0	0
2021-04-30 06:00:00	14.7	51	2.3	2	253	west wind	947	29.4	0	0
2021-04-30 07:00:00	14.4	50	2.4	2	252	west wind	948	28.3	0	0
2021-04-30 08:00:00	16	45	2.9	2	256	west wind	948	24.1	0	0
2021-04-30 09:00:00	19.8	36	2.3	2	257	west wind	947	24.7	0	0
2021-04-30 10:00:00	23.8	28	1.4	1	281	west wind	947	24	0	0
2021-04-30 11:00:00	26.8	23	0.8	1	324	northwest wind	946	29.3	0	0
2021-04-30 12:00:00	29.4	18	1.2	1	44	northeast wind	946	20.3	0	0
2021-04-30 13:00:00	30.7	18	1.6	2	97	east wind	945	24.1	0	0
2021-04-30 14:00:00	31.7	16	2.1	2	99	east wind	945	28.1	0	0
2021-04-30 15:00:00	32.2	15	2.8	2	86	east wind	944	21.6	0	0
2021-04-30 16:00:00	32.7	13	3.1	2	87	east wind	943	22.7	0	0
2021-04-30 17:00:00	33	12	3.4	3	67	northeast wind	943	24.4	0	0
2021-04-30 18:00:00	33.2	12	2.7	2	94	east wind	942	28.2	0	0
2021-04-30 19:00:00	32.5	13	2.8	2	53	northeast wind	942	17.2	0	0
2021-04-30 20:00:00	31.3	13	1.2	1	49	northeast wind	942	17.7	0	0
2021-04-30 21:00:00	25.9	36	0.8	1	181	south wind	942	10	0	0
2021-04-30 22:00:00	23	33	1.6	2	262	west wind	942	12.9	0	0
2021-04-30 23:00:00	21.6	35	1.7	2	233	southwest wind	943	20.9	0	0
2021-05-01 00:00:00	21.1	35	1.9	2	258	west wind	943	23.4	0	0
2021-05-01 01:00:00	20.6	37	2.6	2	252	west wind	943	26.2	0	0
2021-05-01 02:00:00	18.6	41	1.5	1	196	south wind	943	25	0	0
2021-05-01 03:00:00	19.5	38	2.7	2	248	west wind	942	27.9	0	0
2021-05-01 04:00:00	19.5	36	2	2	249	west wind	942	26.7	0	0
2021-05-01 05:00:00	20.1	33	2.9	2	234	southwest wind	943	1.8	0	0

2021-05-01 06:00:00	24.4	28	9.2	5	250	west wind	944	19.8	0	63
2021-05-01 07:00:00	22.7	35	1.5	1	213	southwest wind	946	10.8	0	93
2021-05-01 08:00:00	22	35	1.8	2	77	east wind	947	8.4	0	70
2021-05-01 09:00:00	21.6	38	1.6	2	75	east wind	948	10.5	0	75
2021-05-01 10:00:00	22.7	37	4	3	76	east wind	949	10.9	0	88
2021-05-01 11:00:00	23.3	39	3.7	3	99	east wind	949	14.7	0	77
2021-05-01 12:00:00	22.7	41	4.9	3	82	east wind	950	11.1	0	0
2021-05-01 13:00:00	23.1	39	4.9	3	85	east wind	950	15.1	0	0
2021-05-01 14:00:00	22.1	39	4.9	3	72	east wind	952	8.5	0	0
2021-05-01 15:00:00	21.3	37	3.3	2	66	northeast wind	953	8.8	0	0
2021-05-01 16:00:00	20.7	36	3.5	3	96	east wind	954	5.8	0	45
2021-05-01 17:00:00	20.4	35	4.7	3	132	southeast wind	955	6.5	10.8	54
2021-05-01 18:00:00	20.5	33	3.5	3	125	southeast wind	956	7.3	0	75
2021-05-01 19:00:00	19.2	34	4.5	3	115	southeast wind	958	7.8	0	0
2021-05-01 20:00:00	18.1	35	4.3	3	116	southeast wind	959	11.3	0	0
2021-05-01 21:00:00	16.6	36	4	3	126	southeast wind	960	17.6	0	0
2021-05-01 22:00:00	15.5	36	3.1	2	132	southeast wind	961	10.4	0	0
2021-05-01 23:00:00	14.5	36	2.7	2	135	southeast wind	962	11.2	0	0
2021-05-02 00:00:00	13.6	37	1.7	2	141	southeast wind	963	17	0	0
2021-05-02 01:00:00	13.5	38	3.4	3	129	southeast wind	963	18.4	0	0
2021-05-02 02:00:00	13.7	37	3.4	3	107	east wind	963	28.6	0	20
2021-05-02 03:00:00	13.3	38	2.4	2	92	east wind	963	29.1	0	26
2021-05-02 04:00:00	12	43	1.4	1	328	northwest wind	963	30	0	0
2021-05-02 05:00:00	11.7	46	0.9	1	284	west wind	963	29.9	0	29
2021-05-02 06:00:00	10.2	56	0.5	1	78	east wind	963	25.8	0	23
2021-05-02 07:00:00	8.9	60	1	1	289	west wind	963	21	0	0
2021-05-02 08:00:00	9.8	56	0.5	1	351	north wind	963	16.8	0	0
2021-05-02 09:00:00	12	49	1.4	1	233	southwest wind	963	30	0	24
2021-05-02 10:00:00	14.3	38	1.6	2	268	west wind	963	26.7	0	0
2021-05-02 11:00:00	16.8	33	1.1	1	24	northeast wind	962	22.4	0	35
2021-05-02 12:00:00	17.4	32	1.7	2	122	southeast wind	962	18.6	0	0
2021-05-02 13:00:00	18	31	2.1	2	117	southeast wind	962	15.9	0	0
2021-05-02 14:00:00	19.1	29	2.9	2	67	northeast wind	961	16.1	0	0
2021-05-02 15:00:00	20.3	28	2.4	2	49	northeast wind	960	17.1	0	0
2021-05-02 16:00:00	20.2	25	2.8	2	84	east wind	960	17.9	0	0
2021-05-02 17:00:00	20.8	24	3.4	3	123	southeast wind	959	19.9	0	0
2021-05-02 18:00:00	20.8	21	3.7	3	125	southeast wind	959	19.9	0	0
2021-05-02 19:00:00	20.5	20	3	2	104	east wind	959	29.3	0	0

2021-05-02 20:00:00	19.6	19	3.1	2	132	southeast wind	959	22.7	0	0
2021-05-02 21:00:00	18.3	20	3.3	2	123	southeast wind	960	20.3	0	0
2021-05-02 22:00:00	16.8	22	2.4	2	140	southeast wind	960	22.9	0	0
2021-05-02 23:00:00	14.3	38	0.7	1	256	west wind	961	17.3	0	0
2021-05-03 00:00:00	13.1	36	1.1	1	294	northwest wind	961	14.7	0	0
2021-05-03 01:00:00	11.3	40	1	1	281	west wind	961	19.4	0	0
2021-05-03 02:00:00	9.4	51	1.1	1	247	southwest wind	961	28.3	0	24
2021-05-03 03:00:00	13.4	22	2.9	2	68	east wind	962	13.6	0	42
2021-05-03 04:00:00	12.1	21	3.3	2	64	northeast wind	962	14.1	0	0
2021-05-03 05:00:00	10.9	22	1.7	2	48	northeast wind	963	14.5	0	33
2021-05-03 06:00:00	9.4	30	1	1	348	north wind	963	15.7	0	64
2021-05-03 07:00:00	7.7	45	0.9	1	286	west wind	963	14.1	0	82
2021-05-03 08:00:00	8.3	51	0.2	0	0	north wind	964	18.3	0	80
2021-05-03 09:00:00	9.8	33	0.9	1	352	north wind	964	30	0	92
2021-05-03 10:00:00	11	23	1.4	1	55	northeast wind	964	30	0	89
2021-05-03 11:00:00	11.9	22	0.6	1	45	northeast wind	964	26.1	0	75
2021-05-03 12:00:00	12.5	20	1.6	2	100	east wind	964	26.6	0	91
2021-05-03 13:00:00	13.2	21	2.1	2	291	west wind	963	29.4	0	93
2021-05-03 14:00:00	13.9	21	1.1	1	277	west wind	963	30	0	91
2021-05-03 15:00:00	14.6	20	1.1	1	271	west wind	962	27.2	0	92
2021-05-03 16:00:00	15.8	21	0.8	1	308	northwest wind	962	22.1	0	90
2021-05-03 17:00:00	16.4	20	1.5	1	114	southeast wind	961	24.9	0	70
2021-05-03 18:00:00	16.8	20	2.3	2	52	northeast wind	961	25.6	0	0
2021-05-03 19:00:00	17	20	1.2	1	57	northeast wind	960	24.6	0	0
2021-05-03 20:00:00	16.7	21	1.4	1	28	northeast wind	960	22.3	0	0
2021-05-03 21:00:00	14.9	31	1	1	111	east wind	961	12.9	0	0
2021-05-03 22:00:00	12.3	42	1.3	1	328	northwest wind	961	11.1	0	0
2021-05-03 23:00:00	10.5	46	0.7	1	277	west wind	961	14.6	0	0
2021-05-04 00:00:00	9	52	1	1	255	west wind	961	14.8	0	0
2021-05-04 01:00:00	7.6	60	1.2	1	250	west wind	960	15.8	0	0
2021-05-04 02:00:00	8	57	1.2	1	274	west wind	959	16.4	0	0
2021-05-04 03:00:00	7.4	56	1.1	1	267	west wind	959	21.2	0	38
2021-05-04 04:00:00	7.1	60	1.3	1	273	west wind	958	22.9	0	29
2021-05-04 05:00:00	6.6	62	1.5	1	257	west wind	958	24.2	0	22
2021-05-04 06:00:00	6.4	63	2	2	260	west wind	958	21.7	0	56
2021-05-04 07:00:00	6.5	63	2.4	2	257	west wind	958	21.2	0	27
2021-05-04 08:00:00	7.9	60	1.8	2	254	west wind	958	20	0	38
2021-05-04 09:00:00	10.7	49	2.5	2	260	west wind	959	25.3	0	0

2021-05-04 10:00:00	15	33	2	2	252	west wind	958	23.1	0	0
2021-05-04 11:00:00	17.6	27	1.2	1	209	southwest wind	958	21.1	0	0
2021-05-04 12:00:00	18.6	22	1.7	2	112	east wind	958	22.7	0	0
2021-05-04 13:00:00	20	22	2.4	2	142	southeast wind	957	19.9	0	0
2021-05-04 14:00:00	20.8	20	2.1	2	97	east wind	956	26.7	0	0
2021-05-04 15:00:00	22.3	21	1.6	2	47	northeast wind	956	26.4	0	0
2021-05-04 16:00:00	22.8	20	1.9	2	71	east wind	955	29.3	0	0
2021-05-04 17:00:00	23.3	20	1.8	2	84	east wind	955	23.3	0	0
2021-05-04 18:00:00	24.1	17	2.2	2	125	southeast wind	954	22	0	0
2021-05-04 19:00:00	23.7	19	2.2	2	131	southeast wind	954	20.6	0	0
2021-05-04 20:00:00	23.6	20	1	1	131	southeast wind	954	16.4	0	0
2021-05-04 21:00:00	20.6	28	0.8	1	105	east wind	954	10.6	0	0
2021-05-04 22:00:00	16.8	42	0.7	1	312	northwest wind	954	10.7	0	0
2021-05-04 23:00:00	14.3	46	0.9	1	274	west wind	954	14.1	0	0
2021-05-05 00:00:00	12.3	50	1.5	1	258	west wind	954	14.9	0	0
2021-05-05 01:00:00	12	51	1.9	2	253	west wind	954	19.1	0	0
2021-05-05 02:00:00	11.3	55	1.4	1	261	west wind	954	23.1	0	0
2021-05-05 03:00:00	11.2	53	2	2	267	west wind	953	24.8	0	0
2021-05-05 04:00:00	10.3	57	1.2	1	258	west wind	953	28.2	0	0
2021-05-05 05:00:00	9.9	57	1.8	2	259	west wind	953	27.6	0	0
2021-05-05 06:00:00	9.4	58	1.8	2	257	west wind	953	27.5	0	0
2021-05-05 07:00:00	9.6	57	2.6	2	270	west wind	953	24.4	0	0
2021-05-05 08:00:00	10.9	53	1.7	2	260	west wind	954	28.4	0	0
2021-05-05 09:00:00	13.9	45	1.7	2	260	west wind	954	24	0	0
2021-05-05 10:00:00	17.7	35	1.6	2	246	southwest wind	953	21.9	0	0
2021-05-05 11:00:00	21	24	2.2	2	74	east wind	953	26.3	0	0
2021-05-05 12:00:00	22.4	21	2.8	2	60	northeast wind	952	29.8	0	0
2021-05-05 13:00:00	23.6	20	2.6	2	77	east wind	952	26.1	0	0
2021-05-05 14:00:00	24.2	18	2.6	2	74	east wind	951	30	0	0
2021-05-05 15:00:00	25.1	16	2.1	2	90	east wind	950	29.7	0	0
2021-05-05 16:00:00	25.5	14	2.5	2	60	northeast wind	950	30	0	0
2021-05-05 17:00:00	26.3	13	2.1	2	43	northeast wind	949	30	0	0
2021-05-05 18:00:00	26	10	2.3	2	35	northeast wind	949	30	0	0
2021-05-05 19:00:00	25.8	12	1.5	1	58	northeast wind	948	30	0	0
2021-05-05 20:00:00	25.5	11	1.6	2	32	northeast wind	948	24.8	0	0
2021-05-05 21:00:00	22.2	24	0.4	1	301	northwest wind	948	14.2	0	0
2021-05-05 22:00:00	18.2	36	1	1	281	west wind	949	16.2	0	0
2021-05-05 23:00:00	16	38	1.5	1	248	west wind	949	21.6	0	0

2021-05-06 00:00:00	15.7	35	2.1	2	258	west wind	949	23.5	0	0
2021-05-06 01:00:00	15.3	35	2.2	2	263	west wind	949	26.7	0	46
2021-05-06 02:00:00	15.3	35	2.9	2	262	west wind	949	28.4	0	0
2021-05-06 03:00:00	15.5	34	3.3	2	257	west wind	949	30	0	0
2021-05-06 04:00:00	15.1	35	2.6	2	253	west wind	948	30	0	0
2021-05-06 05:00:00	14	39	2.5	2	254	west wind	948	30	0	0
2021-05-06 06:00:00	13.4	41	2	2	265	west wind	948	30	0	0
2021-05-06 07:00:00	12.3	45	1.4	1	266	west wind	948	28.8	0	0
2021-05-06 08:00:00	14	41	2.4	2	258	west wind	949	24.7	0	0
2021-05-06 09:00:00	17.2	36	2.6	2	256	west wind	949	21.8	0	0
2021-05-06 10:00:00	21.6	27	2	2	258	west wind	949	29.2	0	0
2021-05-06 11:00:00	25.2	19	0.8	1	206	southwest wind	948	28.6	0	0
2021-05-06 12:00:00	27.2	16	0.8	1	314	northwest wind	948	22.5	0	0
2021-05-06 13:00:00	27.4	14	1.4	1	57	northeast wind	948	21.4	0	0
2021-05-06 14:00:00	28.4	15	1.8	2	106	east wind	948	27.7	0	0
2021-05-06 15:00:00	28.9	16	2.1	2	79	east wind	947	25.4	0	0
2021-05-06 16:00:00	29.3	14	1.9	2	126	southeast wind	947	25.1	0	0
2021-05-06 17:00:00	29.5	13	2.7	2	83	east wind	946	27.3	0	0
2021-05-06 18:00:00	30.2	13	1.4	1	133	southeast wind	946	24.8	0	0
2021-05-06 19:00:00	29.7	13	1.5	1	98	east wind	946	20.1	0	0
2021-05-06 20:00:00	29.3	14	1.4	1	91	east wind	946	21.9	0	0
2021-05-06 21:00:00	24.7	27	1	1	18	north wind	946	9.6	0	0
2021-05-06 22:00:00	21.3	35	1.1	1	262	west wind	947	14.8	0	0
2021-05-06 23:00:00	18.9	39	1.7	2	258	west wind	948	14.3	0	0
2021-05-07 00:00:00	19	37	1.8	2	257	west wind	948	16.8	0	0
2021-05-07 01:00:00	19	37	1.8	2	257	west wind	948	20.1	0	0
2021-05-07 02:00:00	19	37	1.8	2	257	west wind	948	24.7	0	0
2021-05-07 03:00:00	19	37	1.8	2	257	west wind	948	24.9	0	31
2021-05-07 04:00:00	16.9	42	2	2	262	west wind	948	26.8	0	25
2021-05-07 05:00:00	16.3	43	2	2	269	west wind	948	26.6	0	0
2021-05-07 06:00:00	15.8	43	1.9	2	258	west wind	948	29.2	0	0
2021-05-07 07:00:00	15.9	42	1.8	2	249	west wind	949	27.1	0	27
2021-05-07 08:00:00	16.8	41	1.8	2	253	west wind	949	23.2	0	25
2021-05-07 09:00:00	18.6	39	1.7	2	267	west wind	949	22.1	0	76
2021-05-07 10:00:00	22.3	32	2.4	2	239	southwest wind	949	22.3	0	43
2021-05-07 11:00:00	26.5	20	0.7	1	278	west wind	949	16.8	0	0
2021-05-07 12:00:00	28.9	17	1.3	1	113	southeast wind	949	23.9	0	0
2021-05-07 13:00:00	30.2	17	2.7	2	135	southeast wind	949	22.3	0	35

2021-05-07 14:00:00	30.7	15	2	2	101	east wind	948	30	0	85
2021-05-07 15:00:00	31.3	15	2.4	2	115	southeast wind	948	27.9	0	93
2021-05-07 16:00:00	31.7	17	2.1	2	74	east wind	947	29.8	0	68
2021-05-07 17:00:00	32.5	15	1.7	2	73	east wind	947	22.1	0	0
2021-05-07 18:00:00	31.6	20	3.4	3	21	north wind	946	25.3	0	58
2021-05-07 19:00:00	30.4	23	3	2	306	northwest wind	947	20.4	0	0
2021-05-07 20:00:00	28.3	28	3	2	1	north wind	947	27.1	0	32
2021-05-07 21:00:00	27.2	30	1.4	1	253	west wind	948	21.8	0	46
2021-05-07 22:00:00	25.6	34	1	1	244	southwest wind	949	20	0	0
2021-05-07 23:00:00	24.3	39	2.5	2	147	southeast wind	950	8	0	41
2021-05-08 00:00:00	23.7	39	1.5	1	145	southeast wind	950	5.6	0	37
2021-05-08 01:00:00	23.3	41	1.8	2	152	southeast wind	950	11.9	0	41
2021-05-08 02:00:00	23.7	39	2.6	2	56	northeast wind	950	12.6	0	0
2021-05-08 03:00:00	23.2	40	2.6	2	68	east wind	950	14.2	0	31
2021-05-08 04:00:00	21.6	46	0.9	1	51	northeast wind	950	19.2	0	0
2021-05-08 05:00:00	20.3	51	1.3	1	226	southwest wind	951	19.6	0	32
2021-05-08 06:00:00	19.1	54	1.5	1	263	west wind	951	15	0	46
2021-05-08 07:00:00	18.3	58	1.7	2	256	west wind	951	13.1	0	50
2021-05-08 08:00:00	18.4	59	1.5	1	241	southwest wind	951	12.1	0	68
2021-05-08 09:00:00	19.9	57	1.6	2	261	west wind	952	19.7	0	59
2021-05-08 10:00:00	23.2	43	0.4	1	52	northeast wind	952	16.5	0	65
2021-05-08 11:00:00	23.7	42	0.9	1	101	east wind	952	23.8	0	49
2021-05-08 12:00:00	25.2	40	1.6	2	98	east wind	952	25.4	0	48
2021-05-08 13:00:00	25.6	37	1.5	1	39	northeast wind	951	25.5	0	72
2021-05-08 14:00:00	26.8	35	2.8	2	136	southeast wind	951	23.6	0	64
2021-05-08 15:00:00	27.4	34	2.3	2	62	northeast wind	950	23.4	0	0
2021-05-08 16:00:00	28.1	33	2.9	2	79	east wind	950	26.2	0	62
2021-05-08 17:00:00	27.4	34	3	2	42	northeast wind	949	26.9	0	0
2021-05-08 18:00:00	26.4	36	2.6	2	28	northeast wind	950	30	0	0
2021-05-08 19:00:00	26.9	34	2.8	2	54	northeast wind	950	30	0	55
2021-05-08 20:00:00	26.2	36	2.1	2	120	southeast wind	950	20.1	0	41
2021-05-08 21:00:00	24.9	38	3	2	132	southeast wind	951	19.9	0	0
2021-05-08 22:00:00	24	41	3.6	3	123	southeast wind	951	25	0	0
2021-05-08 23:00:00	22.8	46	1.7	2	105	east wind	952	19.4	0	0
2021-05-09 00:00:00	21.6	50	1.6	2	122	southeast wind	952	26.7	0	0
2021-05-09 01:00:00	20.8	53	1.2	1	224	southwest wind	952	22.7	0	19
2021-05-09 02:00:00	19.8	58	0.7	1	288	west wind	952	22.8	0	21
2021-05-09 03:00:00	18.4	63	0.9	1	344	north wind	952	15.2	0	0

2021-05-09 04:00:00	16.6	70	1.7	2	257	west wind	951	22.4	0	0
2021-05-09 05:00:00	15.9	72	1.3	1	245	southwest wind	951	22.6	0	0
2021-05-09 06:00:00	15.6	74	1.3	1	263	west wind	951	23.7	0	0
2021-05-09 07:00:00	15.1	77	1.5	1	248	west wind	951	18.9	0	0
2021-05-09 08:00:00	16.3	74	1.6	2	259	west wind	952	22.8	0	0
2021-05-09 09:00:00	19	65	1.8	2	249	west wind	952	24.9	0	0
2021-05-09 10:00:00	21.7	50	1.1	1	206	southwest wind	952	29	0	0
2021-05-09 11:00:00	23.4	45	1.4	1	241	southwest wind	951	26.8	0	0
2021-05-09 12:00:00	25.3	39	1.6	2	70	east wind	950	22	0	25
2021-05-09 13:00:00	26.3	39	2.2	2	109	east wind	949	28.1	0	55
2021-05-09 14:00:00	26.9	35	2.1	2	66	northeast wind	949	29.2	0	30
2021-05-09 15:00:00	27.7	35	2.2	2	85	east wind	948	29.9	0	0
2021-05-09 16:00:00	29	28	1.9	2	78	east wind	947	27.3	0	0
2021-05-09 17:00:00	28.9	28	2.8	2	75	east wind	946	26.3	0	0
2021-05-09 18:00:00	29	28	2	2	96	east wind	946	25.2	0	0
2021-05-09 19:00:00	29	28	2.1	2	102	east wind	946	27.9	0	0
2021-05-09 20:00:00	27.3	32	1.7	2	140	southeast wind	946	13.7	0	0
2021-05-09 21:00:00	25.3	41	0.7	1	145	southeast wind	946	11.3	0	0
2021-05-09 22:00:00	23.3	50	0.9	1	286	west wind	947	12	0	30
2021-05-09 23:00:00	22.1	51	0.5	1	340	north wind	947	14.3	0	50
2021-05-10 00:00:00	21	57	0.9	1	298	northwest wind	947	19.2	0	32
2021-05-10 01:00:00	21	54	1.2	1	253	west wind	947	25	0	0
2021-05-10 02:00:00	20.3	57	1.9	2	275	west wind	947	29.1	0	0
2021-05-10 03:00:00	19.6	58	1.5	1	273	west wind	947	30	0	0
2021-05-10 04:00:00	20	56	2.5	2	265	west wind	947	30	0	0
2021-05-10 05:00:00	18.8	60	1.6	2	262	west wind	946	30	0	1
2021-05-10 06:00:00	18.3	62	1.5	1	252	west wind	947	30	0	47
2021-05-10 07:00:00	18.2	63	1.4	1	245	southwest wind	947	30	0	38
2021-05-10 08:00:00	18.5	62	1.6	2	251	west wind	948	30	0	84
2021-05-10 09:00:00	19	65	1.5	1	276	west wind	948	17.8	0	66
2021-05-10 10:00:00	19.4	72	1.7	2	305	northwest wind	949	21.6	0	82
2021-05-10 11:00:00	20.6	66	0.9	1	197	south wind	950	21.9	0	93
2021-05-10 12:00:00	23.7	43	2.5	2	83	east wind	950	30	0	93
2021-05-10 13:00:00	24.4	39	2.1	2	99	east wind	949	23	0	93
2021-05-10 14:00:00	24.8	41	2.1	2	106	east wind	949	19.2	0	78
2021-05-10 15:00:00	24.4	40	4.7	3	121	southeast wind	948	25.1	0	94
2021-05-10 16:00:00	24.9	43	3.7	3	112	east wind	948	20	0	91
2021-05-10 17:00:00	25.1	40	4.4	3	114	southeast wind	947	29.8	0	64

2021-05-10 18:00:00	25.6	37	3.6	3	118	southeast wind	947	28.4	0	41
2021-05-10 19:00:00	24.9	40	3.6	3	127	southeast wind	947	30	0	66
2021-05-10 20:00:00	23.8	43	3.3	2	137	southeast wind	947	26.2	0	0
2021-05-10 21:00:00	22.9	47	2.2	2	157	southeast wind	947	30	0	0
2021-05-10 22:00:00	22.5	46	3.4	3	139	southeast wind	948	30	0	0
2021-05-10 23:00:00	21	53	0.7	1	189	south wind	948	30	0	58
2021-05-11 00:00:00	19.2	60	1	1	303	northwest wind	948	30	0	22
2021-05-11 01:00:00	18.6	62	1.6	2	302	northwest wind	948	30	0	29
2021-05-11 02:00:00	18.4	64	1.3	1	312	northwest wind	948	30	0	61
2021-05-11 03:00:00	17.5	69	0.8	1	242	southwest wind	947	30	0	74
2021-05-11 04:00:00	17.7	67	1.2	1	263	west wind	947	30	0	87
2021-05-11 05:00:00	17.4	68	1.1	1	307	northwest wind	947	30	0	42
2021-05-11 06:00:00	16.3	73	1.4	1	254	west wind	947	21.3	0	60
2021-05-11 07:00:00	16.4	74	1.3	1	271	west wind	947	18.6	0	91
2021-05-11 08:00:00	17.2	71	0.4	1	261	west wind	948	29.2	0	90
2021-05-11 09:00:00	19.5	60	0.4	1	214	southwest wind	948	30	0	71
2021-05-11 10:00:00	21.1	50	2	2	90	east wind	948	28.7	0	73
2021-05-11 11:00:00	21.9	39	2.8	2	111	east wind	948	24.3	0	80
2021-05-11 12:00:00	22.1	38	3.5	3	115	southeast wind	948	27.6	0	93
2021-05-11 13:00:00	23	39	3.6	3	96	east wind	948	30	0	91
2021-05-11 14:00:00	22.4	43	3.2	2	85	east wind	947	30	0	93
2021-05-11 15:00:00	24	35	2.2	2	74	east wind	947	30	0	91
2021-05-11 16:00:00	23.7	36	1.2	1	355	north wind	948	30	0	91
2021-05-11 17:00:00	23.1	39	2.6	2	323	northwest wind	948	30	0	93
2021-05-11 18:00:00	22.7	38	2.9	2	23	northeast wind	948	30	0	93
2021-05-11 19:00:00	19.9	46	3.1	2	340	north wind	949	10.3	0	88
2021-05-11 20:00:00	15.4	86	2	2	295	northwest wind	951	19.3	0.1	79
2021-05-11 21:00:00	15.4	91	0.9	1	263	west wind	951	5.7	2.4	83
2021-05-11 22:00:00	14.6	94	2.4	2	261	west wind	953	11.5	1.3	0
2021-05-11 23:00:00	14.1	95	1.1	1	271	west wind	953	21	9.9	19
2021-05-12 00:00:00	14.1	97	0.4	1	302	northwest wind	953	9.1	1.5	0
2021-05-12 01:00:00	14.3	98	0.5	1	296	northwest wind	953	24.6	0.1	0
2021-05-12 02:00:00	14.1	96	1.2	1	305	northwest wind	952	12.2	0.5	0
2021-05-12 03:00:00	13.5	97	1.3	1	279	west wind	951	15.6	0	0
2021-05-12 04:00:00	13	97	0.7	1	299	northwest wind	951	7.2	0	0
2021-05-12 05:00:00	12.3	98	1	1	281	west wind	950	3.6	0	0
2021-05-12 06:00:00	12.1	99	0.6	1	257	west wind	950	6.9	0	0
2021-05-12 07:00:00	12	100	0.9	1	347	north wind	950	3.8	0.1	40

2021-05-12 08:00:00	12.7	100	0.7	1	255	west wind	950	11.5	0	66
2021-05-12 09:00:00	15.1	95	0.6	1	152	southeast wind	950	17.2	0	0
2021-05-12 10:00:00	16.5	78	2.1	2	81	east wind	950	22.1	0	0
2021-05-12 11:00:00	16.4	80	2.6	2	113	southeast wind	950	30	0	0
2021-05-12 12:00:00	16.2	66	3.8	3	128	southeast wind	951	30	0	57
2021-05-12 13:00:00	15.6	70	1.7	2	181	south wind	952	26.1	0	79
2021-05-12 14:00:00	14.6	83	2.3	2	91	east wind	952	30	1.5	75
2021-05-12 15:00:00	15.5	66	2.8	2	80	east wind	953	30	0.1	94
2021-05-12 16:00:00	14.5	70	5.6	4	135	southeast wind	953	30	0	96
2021-05-12 17:00:00	13.8	69	6.1	4	126	southeast wind	954	30	0	95
2021-05-12 18:00:00	13.8	67	4.2	3	112	east wind	955	30	0	94
2021-05-12 19:00:00	13.5	65	2.9	2	115	southeast wind	956	30	0	80
2021-05-12 20:00:00	13.6	65	2.9	2	124	southeast wind	956	30	0	90
2021-05-12 21:00:00	13.5	67	1.3	1	311	northwest wind	957	27.7	0	89
2021-05-12 22:00:00	13.1	70	1.1	1	324	northwest wind	958	14.9	0	76
2021-05-12 23:00:00	12.3	81	0.9	1	222	southwest wind	958	30	0	78
2021-05-13 00:00:00	12.2	81	0.9	1	225	southwest wind	959	30	0	55
2021-05-13 01:00:00	12.9	66	2	2	215	southwest wind	959	30	0	50
2021-05-13 02:00:00	12.8	63	1.7	2	231	southwest wind	958	30	0	90
2021-05-13 03:00:00	11.9	75	1.1	1	251	west wind	958	30	0	50
2021-05-13 04:00:00	11.4	76	1.6	2	271	west wind	957	30	0	45
2021-05-13 05:00:00	10.2	84	1.4	1	234	southwest wind	957	30	0	55
2021-05-13 06:00:00	10	84	2.4	2	260	west wind	957	30	0	62
2021-05-13 07:00:00	10	79	2.2	2	252	west wind	957	27.2	0	56
2021-05-13 08:00:00	11.4	69	2.1	2	261	west wind	957	30	0	58
2021-05-13 09:00:00	13.3	61	2.1	2	244	southwest wind	957	30	0	0
2021-05-13 10:00:00	15.6	52	2.3	2	250	west wind	957	30	0	0
2021-05-13 11:00:00	16.3	49	2.5	2	254	west wind	956	30	0	0
2021-05-13 12:00:00	18	32	1.8	2	160	south wind	956	30	0	0
2021-05-13 13:00:00	19	25	1.8	2	256	west wind	955	30	0	0
2021-05-13 14:00:00	19.2	30	1.2	1	13	north wind	955	30	0	0
2021-05-13 15:00:00	20.2	30	1.9	2	116	southeast wind	954	30	0	0
2021-05-13 16:00:00	20.6	28	1.6	2	20	north wind	954	30	0	0
2021-05-13 17:00:00	20.6	27	2.5	2	53	northeast wind	954	30	0	0
2021-05-13 18:00:00	20.6	27	2.5	2	42	northeast wind	953	30	0	42
2021-05-13 19:00:00	21	26	1.8	2	344	north wind	953	30	0	0
2021-05-13 20:00:00	20.6	26	1.6	2	20	north wind	953	30	0	32
2021-05-13 21:00:00	19.1	37	0.7	1	40	northeast wind	953	27	0	0

2021-05-13 22:00:00	18	42	0.9	1	279	west wind	954	16.9	0	32
2021-05-13 23:00:00	16.8	49	0.4	1	240	southwest wind	954	21.7	0	63
2021-05-14 00:00:00	15.7	58	1.1	1	278	west wind	954	28.1	0	42
2021-05-14 01:00:00	16	51	2.2	2	267	west wind	954	30	0	40
2021-05-14 02:00:00	15.8	51	0.5	1	80	east wind	953	30	0	57
2021-05-14 03:00:00	15.6	50	1.3	1	354	north wind	952	30	0	75
2021-05-14 04:00:00	14.8	61	1	1	108	east wind	952	30	0	0
2021-05-14 05:00:00	14.3	62	1.4	1	234	southwest wind	952	17.6	0	63
2021-05-14 06:00:00	13.7	57	2.3	2	149	southeast wind	952	17.3	0	17
2021-05-14 07:00:00	12.9	61	1	1	132	southeast wind	952	16.6	0	66
2021-05-14 08:00:00	14.1	55	1.7	2	94	east wind	952	17.8	0	68
2021-05-14 09:00:00	15	52	1.9	2	85	east wind	952	15.8	0	53
2021-05-14 10:00:00	15.9	54	1.6	2	123	southeast wind	952	17.4	0	0
2021-05-14 11:00:00	17	46	1.2	1	74	east wind	952	20	0	64
2021-05-14 12:00:00	17.3	44	1.4	1	321	northwest wind	952	20.1	0	77
2021-05-14 13:00:00	18	42	2.1	2	55	northeast wind	952	21.1	0	88
2021-05-14 14:00:00	17.6	43	2.1	2	295	northwest wind	952	25.4	0	93
2021-05-14 15:00:00	14.6	60	4.5	3	243	southwest wind	953	20.9	0	93
2021-05-14 16:00:00	14.4	71	3	2	332	northwest wind	953	30	0.6	93
2021-05-14 17:00:00	15.3	60	3.3	2	357	north wind	953	30	0.1	40
2021-05-14 18:00:00	16	51	2.5	2	8	north wind	954	30	0	83
2021-05-14 19:00:00	16.7	49	1.2	1	27	northeast wind	954	30	0	79
2021-05-14 20:00:00	16	49	1.3	1	40	northeast wind	955	30	0	57
2021-05-14 21:00:00	15.5	56	1	1	263	west wind	956	30	0	81
2021-05-14 22:00:00	15.2	56	3.5	3	255	west wind	956	18.7	0	78
2021-05-14 23:00:00	12.2	87	1.6	2	185	south wind	957	25.1	0.3	89
2021-05-15 00:00:00	12.3	86	2.3	2	185	south wind	958	8.2	1	61
2021-05-15 01:00:00	10.9	92	3.7	3	256	west wind	958	12.9	1.7	62
2021-05-15 02:00:00	10.2	92	1.9	2	247	southwest wind	959	30	0.8	89
2021-05-15 03:00:00	10.1	88	2.2	2	214	southwest wind	959	24.8	1.3	88
2021-05-15 04:00:00	10	88	1.3	1	215	southwest wind	959	28.6	0.5	83
2021-05-15 05:00:00	9.7	90	2.1	2	233	southwest wind	959	30	0.6	93
2021-05-15 06:00:00	9.6	89	2.2	2	261	west wind	959	30	0.1	90
2021-05-15 07:00:00	9.7	85	2.2	2	257	west wind	959	30	0.1	93
2021-05-15 08:00:00	9.9	81	2.2	2	257	west wind	960	30	0	70
2021-05-15 09:00:00	10.5	79	2.6	2	258	west wind	960	30	0	78
2021-05-15 10:00:00	11.9	70	2.6	2	251	west wind	960	30	0	89
2021-05-15 11:00:00	14	56	3.2	2	255	west wind	960	30	0	86

2021-05-15 12:00:00	14.3	57	2.2	2	250	west wind	960	30	0	91
2021-05-15 13:00:00	16	50	2.3	2	271	west wind	959	30	0	63
2021-05-15 14:00:00	16.7	47	1.6	2	213	southwest wind	959	30	0	50
2021-05-15 15:00:00	17.9	45	1.1	1	182	south wind	958	30	0	75
2021-05-15 16:00:00	18.4	46	1.2	1	351	north wind	958	30	0	94
2021-05-15 17:00:00	18.6	38	1.5	1	129	southeast wind	958	30	0	45
2021-05-15 18:00:00	19.2	38	0.8	1	348	north wind	958	30	0	67
2021-05-15 19:00:00	19.2	37	1.6	2	56	northeast wind	958	30	0	65
2021-05-15 20:00:00	18.4	40	1.4	1	94	east wind	958	30	0	40
2021-05-15 21:00:00	17.2	50	1.4	1	125	southeast wind	958	28.7	0	27
2021-05-15 22:00:00	15.3	59	0.5	1	219	southwest wind	958	24.1	0	66
2021-05-15 23:00:00	14.8	63	1	1	306	northwest wind	959	21.3	0	41
2021-05-16 00:00:00	14.3	69	1	1	285	west wind	958	24.3	0	41
2021-05-16 01:00:00	13.1	78	0.9	1	272	west wind	958	10	0	0
2021-05-16 02:00:00	12.5	80	1.4	1	254	west wind	958	23.3	0	27
2021-05-16 03:00:00	12	86	1.5	1	249	west wind	957	23.5	0	68
2021-05-16 04:00:00	12.4	85	1.5	1	269	west wind	957	30	0	61
2021-05-16 05:00:00	13	79	1.3	1	263	west wind	957	27.3	0	37
2021-05-16 06:00:00	12.1	82	1.1	1	286	west wind	957	28.5	0	42
2021-05-16 07:00:00	11.9	81	1.1	1	3	north wind	957	27.9	0	0
2021-05-16 08:00:00	13.5	77	1.1	1	255	west wind	957	20	0	70
2021-05-16 09:00:00	15.4	63	1.1	1	316	northwest wind	957	28.7	0	0
2021-05-16 10:00:00	17.2	56	1.1	1	173	south wind	957	30	0	64
2021-05-16 11:00:00	18.1	49	0.3	1	123	southeast wind	957	30	0	81
2021-05-16 12:00:00	19.3	45	1	1	113	southeast wind	957	30	0	91
2021-05-16 13:00:00	19.6	40	1.2	1	346	north wind	957	30	0	89
2021-05-16 14:00:00	20.1	45	1.7	2	273	west wind	956	28.3	0	69
2021-05-16 15:00:00	20.8	41	2.3	2	260	west wind	956	30	0	75
2021-05-16 16:00:00	21.1	41	1.4	1	229	southwest wind	955	30	0	0
2021-05-16 17:00:00	21.7	41	2.1	2	223	southwest wind	955	30	0	0
2021-05-16 18:00:00	22.5	37	2.6	2	262	west wind	955	30	0	45
2021-05-16 19:00:00	22.4	36	1	1	258	west wind	955	30	0	69
2021-05-16 20:00:00	18.4	54	5.3	3	29	northeast wind	956	28.1	0	73
2021-05-16 21:00:00	16	85	1.3	1	258	west wind	956	17.3	3.1	41
2021-05-16 22:00:00	16.1	85	0.9	1	293	northwest wind	957	30	0	28
2021-05-16 23:00:00	16.9	58	3.6	3	242	southwest wind	957	30	0	34
2021-05-17 00:00:00	16.3	57	4	3	261	west wind	956	30	0	0
2021-05-17 01:00:00	15.9	56	3.6	3	261	west wind	956	30	0	28

2021-05-17 02:00:00	15.4	59	3	2	258	west wind	956	30	0	32
2021-05-17 03:00:00	15.7	58	2.8	2	247	southwest wind	955	30	0	63
2021-05-17 04:00:00	15.3	59	2.9	2	246	southwest wind	955	30	0	54
2021-05-17 05:00:00	14.2	62	1.9	2	228	southwest wind	955	30	0	45
2021-05-17 06:00:00	13.6	62	1.4	1	256	west wind	955	30	0	45
2021-05-17 07:00:00	13.1	60	2.7	2	238	southwest wind	956	30	0	41
2021-05-17 08:00:00	14.7	51	2.5	2	242	southwest wind	956	30	0	0
2021-05-17 09:00:00	16.8	45	3	2	255	west wind	956	30	0	0
2021-05-17 10:00:00	18.6	39	2.4	2	259	west wind	956	30	0	0
2021-05-17 11:00:00	21.6	32	2.3	2	231	southwest wind	956	30	0	0
2021-05-17 12:00:00	23.4	27	1.4	1	167	south wind	956	30	0	1
2021-05-17 13:00:00	24.7	27	0.8	1	31	northeast wind	955	30	0	23
2021-05-17 14:00:00	24.7	27	4.9	3	298	northwest wind	955	30	0	23
2021-05-17 15:00:00	24.5	25	4.3	3	296	northwest wind	955	30	0	23
2021-05-17 16:00:00	24.1	29	3.1	2	306	northwest wind	954	30	0	23
2021-05-17 17:00:00	25.4	24	1.4	1	275	west wind	954	30	0	23
2021-05-17 18:00:00	25.8	21	2.4	2	292	west wind	954	30	0	23
2021-05-17 19:00:00	23.9	29	1.9	2	280	west wind	953	30	0	23
2021-05-17 20:00:00	25.4	25	1.1	1	281	west wind	953	27.7	0	23
2021-05-17 21:00:00	22	33	1.6	2	270	west wind	954	19.7	0	0
2021-05-17 22:00:00	18.6	48	1.3	1	250	west wind	954	17.1	0	0
2021-05-17 23:00:00	16.5	58	1.5	1	245	southwest wind	954	30	0	0
2021-05-18 00:00:00	16.7	53	1.9	2	257	west wind	954	30	0	0
2021-05-18 01:00:00	15.8	57	1.9	2	261	west wind	954	30	0	0
2021-05-18 02:00:00	15.3	58	1.6	2	247	southwest wind	954	30	0	0
2021-05-18 03:00:00	14.4	60	2	2	251	west wind	954	30	0	0
2021-05-18 04:00:00	13.8	61	2.1	2	252	west wind	953	30	0	0
2021-05-18 05:00:00	13.4	59	2.4	2	250	west wind	953	30	0	0
2021-05-18 06:00:00	13.3	61	2	2	251	west wind	953	30	0	0
2021-05-18 07:00:00	13.1	56	2.5	2	255	west wind	953	30	0	0
2021-05-18 08:00:00	15.7	49	2.9	2	256	west wind	953	30	0	0
2021-05-18 09:00:00	18.6	46	2.4	2	256	west wind	953	30	0	0
2021-05-18 10:00:00	22.1	33	2.8	2	261	west wind	953	30	0	0
2021-05-18 11:00:00	24.4	28	2.3	2	250	west wind	952	30	0	0
2021-05-18 12:00:00	26	23	1.5	1	183	south wind	952	30	0	0
2021-05-18 13:00:00	26.8	20	0.9	1	340	north wind	951	30	0	0
2021-05-18 14:00:00	27.4	20	1.5	1	317	northwest wind	951	30	0	0
2021-05-18 15:00:00	28.1	17	2.4	2	142	southeast wind	950	30	0	0

2021-05-18 16:00:00	28.6	20	1.9	2	357	north wind	949	30	0	0
2021-05-18 17:00:00	28.7	21	1.1	1	35	northeast wind	949	30	0	0
2021-05-18 18:00:00	29.1	17	1.2	1	282	west wind	949	30	0	0
2021-05-18 19:00:00	28.6	17	1.3	1	21	north wind	948	30	0	0
2021-05-18 20:00:00	28.1	18	1.5	1	84	east wind	948	30	0	0
2021-05-18 21:00:00	26.1	24	1.7	2	80	east wind	948	30	0	0
2021-05-18 22:00:00	23.5	34	1.1	1	84	east wind	948	24	0	0
2021-05-18 23:00:00	19.3	53	1.3	1	259	west wind	949	20.3	0	0
2021-05-19 00:00:00	18.8	52	1.6	2	258	west wind	949	24.7	0	0
2021-05-19 01:00:00	17.2	62	0.7	1	232	southwest wind	949	28.4	0	0
2021-05-19 02:00:00	16.7	59	1.6	2	266	west wind	948	30	0	0
2021-05-19 03:00:00	15.8	59	1.1	1	238	southwest wind	948	30	0	0
2021-05-19 04:00:00	16.1	55	1.7	2	258	west wind	948	30	0	0
2021-05-19 05:00:00	14.8	59	1.1	1	264	west wind	948	30	0	0
2021-05-19 06:00:00	15.3	55	1.9	2	258	west wind	947	30	0	0
2021-05-19 07:00:00	15.5	54	2.7	2	255	west wind	947	30	0	0
2021-05-19 08:00:00	17.4	50	2.1	2	257	west wind	947	30	0	0
2021-05-19 10:00:00	17.4	50	2.1	2	257	west wind	947	30	0	0
2021-05-19 11:00:00	25.2	30	1.5	1	234	southwest wind	945	30	0	0
2021-05-19 12:00:00	27.3	27	0.9	1	49	northeast wind	945	30	0	0
2021-05-19 13:00:00	27.7	25	1.6	2	303	northwest wind	944	30	0	0
2021-05-19 14:00:00	29.2	23	1.6	2	198	south wind	943	30	0	0
2021-05-19 15:00:00	29.6	20	1.3	1	320	northwest wind	942	30	0	0
2021-05-19 16:00:00	30.2	16	2.6	2	360	north wind	941	30	0	0
2021-05-19 17:00:00	30.5	16	1.8	2	55	northeast wind	940	30	0	0
2021-05-19 18:00:00	30.5	18	2.1	2	43	northeast wind	940	30	0	0
2021-05-19 19:00:00	29.5	19	1.8	2	334	northwest wind	940	23.6	0	0
2021-05-19 20:00:00	27.7	21	7.2	4	291	west wind	940	30	0	36
2021-05-19 21:00:00	25.7	23	1.6	2	316	northwest wind	941	30	0	27
2021-05-19 22:00:00	24.1	27	0.8	1	259	west wind	941	30	0	0
2021-05-19 23:00:00	23.7	29	2.2	2	209	southwest wind	942	30	0	67
2021-05-20 00:00:00	22.2	34	1.2	1	270	west wind	941	23.2	0	44
2021-05-20 01:00:00	20.1	40	1.6	2	265	west wind	941	30	0	0
2021-05-20 02:00:00	18.3	46	1	1	244	southwest wind	941	30	0	0
2021-05-20 03:00:00	18.3	45	1.6	2	247	southwest wind	942	30	0	60
2021-05-20 04:00:00	20.5	36	1.9	2	206	southwest wind	942	30	0	0
2021-05-20 05:00:00	19.4	41	1.4	1	188	south wind	942	30	0	33
2021-05-20 06:00:00	19	42	1.8	2	154	southeast wind	942	30	0	82

2021-05-20 07:00:00	17	51	0.8	1	44	northeast wind	942	26.9	0	32
2021-05-20 08:00:00	18.6	53	1.8	2	4	north wind	942	26.1	0	0
2021-05-20 09:00:00	20.5	50	0.9	1	52	northeast wind	942	30	0	0
2021-05-20 10:00:00	22.4	43	1.3	1	350	north wind	943	26.5	0	0
2021-05-20 11:00:00	23.5	38	1.6	2	96	east wind	943	30	0	36
2021-05-20 12:00:00	22.8	40	2.7	2	146	southeast wind	944	30	0	91
2021-05-20 13:00:00	22	41	3.7	3	301	northwest wind	945	26.5	0	95
2021-05-20 14:00:00	17	67	4.3	3	156	southeast wind	946	30	0	95
2021-05-20 15:00:00	17	70	3.4	3	140	southeast wind	947	30	0.1	93
2021-05-20 16:00:00	18.1	60	4.3	3	44	northeast wind	947	30	0	94
2021-05-20 17:00:00	16.5	76	6.1	4	68	east wind	948	30	0	93
2021-05-20 18:00:00	17	65	5.3	3	79	east wind	949	30	0	73
2021-05-20 19:00:00	18.2	53	2.9	2	114	southeast wind	949	30	0	0
2021-05-20 20:00:00	17.9	55	3.1	2	156	southeast wind	949	30	0	30
2021-05-20 21:00:00	16.9	58	1.4	1	161	south wind	950	30	0	32
2021-05-20 22:00:00	15.8	66	1.1	1	244	southwest wind	950	30	0	18
2021-05-20 23:00:00	16	50	2.9	2	124	southeast wind	952	30	0	71
2021-05-21 00:00:00	14.3	48	3.8	3	137	southeast wind	952	30	0	30
2021-05-21 01:00:00	13.7	40	4.4	3	134	southeast wind	953	30	0	38
2021-05-21 02:00:00	13.4	41	1.3	1	63	northeast wind	954	30	0	56
2021-05-21 03:00:00	12.9	43	2.2	2	148	southeast wind	954	30	0	44
2021-05-21 04:00:00	13.2	39	3	2	124	southeast wind	954	28.3	0	72
2021-05-21 05:00:00	12.3	44	4.3	3	111	east wind	955	30	0	71
2021-05-21 06:00:00	11.6	44	1.8	2	340	north wind	955	30	0	65
2021-05-21 07:00:00	10.9	49	1.3	1	8	north wind	956	30	0	35
2021-05-21 08:00:00	13.6	43	1	1	356	north wind	956	30	0	33
2021-05-21 09:00:00	14.6	38	1.4	1	356	north wind	956	30	0	0
2021-05-21 10:00:00	15.6	37	2.6	2	87	east wind	957	30	0	0
2021-05-21 11:00:00	16.3	38	3.1	2	128	southeast wind	956	30	0	0
2021-05-21 12:00:00	16.8	38	3	2	122	southeast wind	956	30	0	0
2021-05-21 13:00:00	18.3	36	2.1	2	102	east wind	956	30	0	47
2021-05-21 14:00:00	18.5	31	3.2	2	119	southeast wind	956	30	0	74
2021-05-21 15:00:00	19.4	32	3	2	130	southeast wind	956	30	0	58
2021-05-21 16:00:00	20.1	30	2.8	2	129	southeast wind	956	25.1	0	0
2021-05-21 17:00:00	20.5	27	1.8	2	92	east wind	956	30	0	0
2021-05-21 18:00:00	20.8	28	2.8	2	143	southeast wind	955	30	0	0
2021-05-21 19:00:00	20.6	26	3.2	2	118	southeast wind	956	21.6	0	0
2021-05-21 20:00:00	19.9	25	3.8	3	124	southeast wind	956	28.9	0	0

2021-05-21 21:00:00	18.7	24	3.5	3	115	southeast wind	957	30	0	0
2021-05-21 22:00:00	17.4	27	2.4	2	127	southeast wind	957	29.5	0	0
2021-05-21 23:00:00	15.5	32	0.9	1	195	south wind	958	23.1	0	61
2021-05-22 00:00:00	13.9	36	0.9	1	112	east wind	959	30	0	0
2021-05-22 01:00:00	14.7	28	2.1	2	116	southeast wind	959	27.5	0	0
2021-05-22 02:00:00	14.5	27	3.4	3	127	southeast wind	959	30	0	30
2021-05-22 03:00:00	14.4	27	2.7	2	112	east wind	959	25.8	0	59
2021-05-22 04:00:00	12.1	36	1.2	1	314	northwest wind	960	30	0	40
2021-05-22 05:00:00	11.2	45	1.3	1	237	southwest wind	960	30	0	0
2021-05-22 06:00:00	10.7	47	1.7	2	247	southwest wind	960	30	0	25
2021-05-22 07:00:00	10.7	49	1.4	1	272	west wind	960	28.4	0	26
2021-05-22 08:00:00	11.8	47	1.6	2	249	west wind	960	30	0	26
2021-05-22 09:00:00	15.8	36	1.5	1	252	west wind	960	30	0	70
2021-05-22 10:00:00	17.3	29	1.2	1	195	south wind	960	30	0	0
2021-05-22 11:00:00	18.6	27	2	2	91	east wind	960	30	0	0
2021-05-22 12:00:00	19.2	25	2.3	2	117	southeast wind	960	30	0	0
2021-05-22 13:00:00	20.3	25	2.5	2	106	east wind	960	30	0	0
2021-05-22 14:00:00	21.3	24	2.3	2	111	east wind	959	30	0	0
2021-05-22 15:00:00	22.1	23	2.7	2	27	northeast wind	959	30	0	0
2021-05-22 16:00:00	22.5	22	2.4	2	104	east wind	959	30	0	0
2021-05-22 17:00:00	23.1	22	2	2	92	east wind	958	30	0	0
2021-05-22 18:00:00	23.8	22	1.3	1	175	south wind	958	30	0	0
2021-05-22 19:00:00	23.5	24	1.6	2	97	east wind	958	30	0	0
2021-05-22 20:00:00	23.4	24	1.5	1	96	east wind	958	30	0	0
2021-05-22 21:00:00	22.2	29	0.6	1	36	northeast wind	958	29.7	0	0
2021-05-22 22:00:00	18.9	40	1	1	305	northwest wind	958	9.1	0	0
2021-05-22 23:00:00	16.7	51	0.8	1	268	west wind	959	22.8	0	0
2021-05-23 00:00:00	14.9	56	0.6	1	217	southwest wind	959	26.8	0	0
2021-05-23 01:00:00	14.9	53	1.5	1	252	west wind	959	25.1	0	0
2021-05-23 02:00:00	14.6	55	1.2	1	246	southwest wind	958	30	0	0
2021-05-23 03:00:00	14.5	54	1.8	2	263	west wind	958	30	0	0
2021-05-23 04:00:00	13.9	55	1.6	2	256	west wind	958	30	0	0
2021-05-23 05:00:00	12.6	60	0.9	1	256	west wind	958	30	0	0
2021-05-23 06:00:00	12.3	62	1.5	1	251	west wind	958	30	0	0
2021-05-23 07:00:00	12.4	62	1.7	2	267	west wind	958	25.7	0	0
2021-05-23 08:00:00	14.4	58	1.9	2	250	west wind	958	25.4	0	0
2021-05-23 09:00:00	18.1	49	0.8	1	297	northwest wind	959	30	0	0
2021-05-23 10:00:00	20.4	37	1.4	1	307	northwest wind	959	30	0	0

2021-05-23 11:00:00	22.5	35	1.9	2	83	east wind	958	30	0	0
2021-05-23 12:00:00	23.2	31	2.9	2	63	northeast wind	958	30	0	0
2021-05-23 13:00:00	24.2	28	3.2	2	75	east wind	958	30	0	0
2021-05-23 14:00:00	24.4	28	2.4	2	68	east wind	958	30	0	0
2021-05-23 15:00:00	25.2	26	3	2	58	northeast wind	957	30	0	0
2021-05-23 16:00:00	26.1	23	2.8	2	72	east wind	956	30	0	0
2021-05-23 17:00:00	26.5	23	2.1	2	106	east wind	956	30	0	0
2021-05-23 18:00:00	26.5	21	2.2	2	69	east wind	955	30	0	0
2021-05-23 19:00:00	26.9	21	1.3	1	83	east wind	955	30	0	0
2021-05-23 20:00:00	26.2	21	1.7	2	50	northeast wind	954	30	0	0
2021-05-23 21:00:00	24.7	27	1.2	1	7	north wind	955	12.9	0	0
2021-05-23 22:00:00	21.4	37	1.1	1	298	northwest wind	955	8.6	0	0
2021-05-23 23:00:00	18.9	46	0.9	1	294	northwest wind	955	21.9	0	0
2021-05-24 00:00:00	17.4	50	1.2	1	245	southwest wind	956	28.1	0	0
2021-05-24 01:00:00	17.6	46	1.7	2	264	west wind	956	30	0	0
2021-05-24 02:00:00	17	49	1.7	2	262	west wind	955	30	0	0
2021-05-24 03:00:00	16.6	48	1.9	2	260	west wind	955	30	0	0
2021-05-24 04:00:00	16.1	47	1.4	1	262	west wind	955	30	0	0
2021-05-24 05:00:00	15.4	51	1.9	2	258	west wind	955	30	0	0
2021-05-24 06:00:00	14.7	52	1.3	1	253	west wind	955	30	0	0
2021-05-24 07:00:00	13.9	56	1.2	1	263	west wind	956	28	0	0
2021-05-24 08:00:00	16.6	50	1.8	2	248	west wind	956	30	0	0
2021-05-24 09:00:00	20	42	2.2	2	257	west wind	956	30	0	0
2021-05-24 10:00:00	22.7	34	2	2	249	west wind	956	30	0	0
2021-05-24 11:00:00	25	26	1.5	1	348	north wind	956	30	0	0
2021-05-24 12:00:00	25.6	25	2	2	70	east wind	955	30	0	0
2021-05-24 13:00:00	26.6	23	1.8	2	56	northeast wind	955	30	0	0
2021-05-24 14:00:00	27.2	22	2.1	2	72	east wind	954	30	0	0
2021-05-24 15:00:00	27.8	23	2	2	39	northeast wind	954	30	0	0
2021-05-24 16:00:00	28.5	22	1.9	2	9	north wind	953	30	0	0
2021-05-24 17:00:00	28.6	23	2.2	2	332	northwest wind	953	30	0	0
2021-05-24 18:00:00	28.8	19	1.4	1	334	northwest wind	952	30	0	0
2021-05-24 19:00:00	29.7	19	0.8	1	324	northwest wind	952	30	0	0
2021-05-24 20:00:00	27.9	24	1.3	1	355	north wind	952	30	0	0
2021-05-24 21:00:00	25.8	33	0.7	1	337	northwest wind	952	12.6	0	0
2021-05-24 22:00:00	21.9	44	0.6	1	278	west wind	952	8.7	0	0
2021-05-24 23:00:00	19	53	1.1	1	253	west wind	952	16.5	0	0
2021-05-25 00:00:00	19.5	47	1.8	2	241	southwest wind	953	29.9	0	0

2021-05-25 01:00:00	19	46	1.7	2	255	west wind	953	30	0	0
2021-05-25 02:00:00	18.7	44	2.1	2	260	west wind	953	30	0	0
2021-05-25 03:00:00	18.3	46	2.3	2	252	west wind	953	30	0	0
2021-05-25 04:00:00	18.1	46	3	2	258	west wind	952	30	0	0
2021-05-25 05:00:00	17.4	48	2.6	2	258	west wind	952	30	0	0
2021-05-25 06:00:00	16.4	51	1.5	1	262	west wind	952	30	0	0
2021-05-25 07:00:00	16.3	53	1.8	2	251	west wind	952	30	0	0
2021-05-25 08:00:00	18.4	49	2.4	2	254	west wind	952	30	0	0
2021-05-25 09:00:00	22.2	39	2.7	2	249	west wind	952	30	0	0
2021-05-25 10:00:00	25.4	29	2.7	2	262	west wind	952	30	0	0
2021-05-25 11:00:00	28.1	25	1.9	2	238	southwest wind	951	30	0	0
2021-05-25 12:00:00	30.1	18	1.1	1	109	east wind	951	30	0	0
2021-05-25 13:00:00	31	15	1.3	1	51	northeast wind	951	30	0	0
2021-05-25 14:00:00	31.8	15	2.7	2	119	southeast wind	950	30	0	0
2021-05-25 15:00:00	32.5	14	1.8	2	165	south wind	950	30	0	0
2021-05-25 16:00:00	33.1	13	1.2	1	3	north wind	949	30	0	0
2021-05-25 17:00:00	33.1	12	2	2	91	east wind	948	30	0	0
2021-05-25 18:00:00	33.1	12	2	2	96	east wind	948	30	0	0
2021-05-25 19:00:00	33.6	11	1.1	1	326	northwest wind	948	30	0	0
2021-05-25 20:00:00	32.6	12	1.3	1	90	east wind	948	14.4	0	0
2021-05-25 21:00:00	29.5	23	0.7	1	204	southwest wind	948	18.9	0	0
2021-05-25 22:00:00	27.5	24	1.2	1	254	west wind	949	27.5	0	0
2021-05-25 23:00:00	24	34	0.9	1	246	southwest wind	949	24.6	0	0
2021-05-26 00:00:00	22.1	36	1	1	268	west wind	949	30	0	0
2021-05-26 01:00:00	20.4	43	1.2	1	236	southwest wind	949	30	0	0
2021-05-26 02:00:00	21	41	1.5	1	241	southwest wind	949	30	0	0
2021-05-26 03:00:00	19.8	43	1.8	2	253	west wind	949	30	0	0
2021-05-26 04:00:00	19.3	43	1.7	2	250	west wind	949	30	0	0
2021-05-26 05:00:00	18.1	47	1.6	2	231	southwest wind	949	30	0	0
2021-05-26 06:00:00	17.5	47	1.4	1	252	west wind	949	30	0	0
2021-05-26 07:00:00	17.3	48	2.4	2	260	west wind	949	23.4	0	0
2021-05-26 08:00:00	19.1	46	2.4	2	254	west wind	949	25.6	0	0
2021-05-26 09:00:00	22.4	40	2.5	2	253	west wind	949	28.6	0	0
2021-05-26 10:00:00	25.5	34	1.3	1	267	west wind	949	21.7	0	0
2021-05-26 11:00:00	28.7	28	0.6	1	281	west wind	949	27	0	0
2021-05-26 12:00:00	30.5	19	1.6	2	111	east wind	949	30	0	0
2021-05-26 13:00:00	30.9	18	2.5	2	100	east wind	948	30	0	0
2021-05-26 14:00:00	31.7	18	3	2	87	east wind	948	30	0	0

2021-05-26 15:00:00	32.1	16	2.9	2	82	east wind	947	30	0	0
2021-05-26 16:00:00	32.7	16	3	2	86	east wind	946	30	0	0
2021-05-26 17:00:00	33.1	15	2.9	2	61	northeast wind	946	30	0	0
2021-05-26 18:00:00	33.2	14	2.2	2	57	northeast wind	945	30	0	0
2021-05-26 19:00:00	32.8	13	1.9	2	29	northeast wind	944	30	0	0
2021-05-26 20:00:00	32.7	15	1.5	1	45	northeast wind	945	20.7	0	0
2021-05-26 21:00:00	29.6	25	0.9	1	113	southeast wind	945	14.1	0	0
2021-05-26 22:00:00	25	38	1	1	300	northwest wind	945	9	0	0
2021-05-26 23:00:00	21.9	42	1.2	1	255	west wind	945	19.2	0	0
2021-05-27 00:00:00	21.6	41	1.8	2	258	west wind	945	28.5	0	0
2021-05-27 01:00:00	21.9	37	2.3	2	257	west wind	946	30	0	0
2021-05-27 02:00:00	21.7	36	2.5	2	262	west wind	945	30	0	0
2021-05-27 03:00:00	21.5	35	2.3	2	255	west wind	945	30	0	0
2021-05-27 04:00:00	20.7	37	2.2	2	255	west wind	945	30	0	0
2021-05-27 05:00:00	20.2	38	2	2	265	west wind	945	30	0	0
2021-05-27 06:00:00	19.3	40	1.3	1	256	west wind	945	30	0	0
2021-05-27 07:00:00	19.2	40	2.2	2	244	southwest wind	945	28.1	0	0
2021-05-27 08:00:00	21.5	40	1.8	2	251	west wind	945	27.3	0	0
2021-05-27 09:00:00	24.8	32	2.3	2	257	west wind	945	28.1	0	0
2021-05-27 10:00:00	27.3	31	1.7	2	252	west wind	945	25.5	0	0
2021-05-27 11:00:00	30.9	22	1.5	1	99	east wind	945	22.5	0	0
2021-05-27 12:00:00	31.8	19	1.9	2	80	east wind	945	30	0	0
2021-05-27 13:00:00	33.1	17	1.7	2	91	east wind	944	30	0	0
2021-05-27 14:00:00	33.9	15	1.5	1	91	east wind	944	29.6	0	0
2021-05-27 15:00:00	34.6	15	1.6	2	74	east wind	943	30	0	0
2021-05-27 16:00:00	34.4	15	1.7	2	50	northeast wind	943	27	0	0
2021-05-27 17:00:00	34.1	18	2.9	2	131	southeast wind	943	25.6	0	93
2021-05-27 18:00:00	32.9	17	2.2	2	238	southwest wind	944	9.8	0	79
2021-05-27 19:00:00	31.3	18	5.7	4	244	southwest wind	944	26.9	0	86
2021-05-27 20:00:00	30.1	23	5.8	4	259	west wind	945	30	0	89
2021-05-27 21:00:00	28.6	27	5	3	264	west wind	946	28.1	0	85
2021-05-27 22:00:00	27.3	26	4.4	3	266	west wind	946	30	0	73
2021-05-27 23:00:00	26.6	27	4.1	3	264	west wind	947	30	0	83
2021-05-28 00:00:00	26.1	29	3.2	2	263	west wind	947	30	0	89
2021-05-28 01:00:00	26.7	27	4.4	3	268	west wind	946	30	0	44
2021-05-28 02:00:00	24.9	32	2.6	2	248	west wind	946	30	0	58
2021-05-28 03:00:00	24	35	2.3	2	256	west wind	946	30	0	46
2021-05-28 04:00:00	22	42	1.1	1	208	southwest wind	946	30	0	42

2021-05-28 05:00:00	23.9	35	2	2	240	southwest wind	946	30	0	26
2021-05-28 06:00:00	22.3	40	1.6	2	121	southeast wind	946	17.5	0	47
2021-05-28 07:00:00	21.7	47	1.2	1	264	west wind	947	20.1	0	29
2021-05-28 08:00:00	21.8	53	0.9	1	258	west wind	948	21	0	87
2021-05-28 09:00:00	25.8	43	1.3	1	133	southeast wind	949	25.5	0	89
2021-05-28 10:00:00	26.6	33	1.9	2	135	southeast wind	949	23.7	0	94
2021-05-28 11:00:00	28.3	29	3.4	3	116	southeast wind	949	21.7	0	38
2021-05-28 12:00:00	30.4	27	2.7	2	103	east wind	950	30	0	0
2021-05-28 13:00:00	30.5	26	2.7	2	30	northeast wind	950	21.4	0	42
2021-05-28 14:00:00	28.8	27	3.7	3	304	northwest wind	950	16.9	0	83
2021-05-28 15:00:00	23.1	51	4	3	224	southwest wind	951	30	0	94
2021-05-28 16:00:00	23.6	55	3.6	3	218	southwest wind	951	30	0	94
2021-05-28 17:00:00	25	45	1.1	1	165	south wind	951	25	0	94
2021-05-28 18:00:00	25.5	46	0.9	1	98	east wind	951	30	0	93
2021-05-28 19:00:00	26	45	2.7	2	78	east wind	951	30	0	69
2021-05-28 20:00:00	26.2	39	1.8	2	72	east wind	951	30	0	35
2021-05-28 21:00:00	24.4	45	1.2	1	337	northwest wind	951	30	0	0
2021-05-28 22:00:00	22	54	1.5	1	247	southwest wind	951	29.1	0	77
2021-05-28 23:00:00	21.3	58	1.7	2	272	west wind	952	26.5	0	56
2021-05-29 00:00:00	20.7	60	1.8	2	246	southwest wind	951	30	0	0
2021-05-29 01:00:00	20.3	61	1.9	2	257	west wind	951	30	0	85
2021-05-29 02:00:00	20	61	2.5	2	263	west wind	951	30	0	28
2021-05-29 03:00:00	20.7	56	2.8	2	255	west wind	950	30	0	0
2021-05-29 04:00:00	19.6	58	1.9	2	246	southwest wind	950	30	0	38
2021-05-29 05:00:00	18	63	1.5	1	248	west wind	950	30	0	0
2021-05-29 06:00:00	17.3	63	1.7	2	255	west wind	950	30	0	75
2021-05-29 07:00:00	17.2	62	1.9	2	250	west wind	950	30	0	0
2021-05-29 08:00:00	18.7	57	2.2	2	259	west wind	950	30	0	0
2021-05-29 09:00:00	21.7	49	1.9	2	252	west wind	950	30	0	53
2021-05-29 10:00:00	25	41	1.5	1	305	northwest wind	950	27.4	0	0
2021-05-29 11:00:00	27.4	36	0.7	1	130	southeast wind	950	30	0	0
2021-05-29 12:00:00	28.9	30	1.6	2	153	southeast wind	950	30	0	0
2021-05-29 13:00:00	30.2	26	2.7	2	145	southeast wind	949	30	0	0
2021-05-29 14:00:00	31.1	26	3.3	2	146	southeast wind	948	30	0	0
2021-05-29 15:00:00	31.1	25	2.9	2	101	east wind	948	24.3	0	93
2021-05-29 16:00:00	30.7	26	4.4	3	138	southeast wind	948	30	0	94
2021-05-29 17:00:00	30.3	27	3.2	2	124	southeast wind	948	30	0	93
2021-05-29 18:00:00	30.6	21	1.5	1	256	west wind	948	30	0	93

2021-05-29 19:00:00	30	27	2.7	2	261	west wind	949	27.3	0	0
2021-05-29 20:00:00	26.5	30	5.8	4	256	west wind	950	30	0	33
2021-05-29 21:00:00	25.5	34	3	2	274	west wind	950	30	0	0
2021-05-29 22:00:00	24.6	36	2.7	2	275	west wind	950	30	0	36
2021-05-29 23:00:00	23.2	39	2	2	247	southwest wind	950	30	0	0
2021-05-30 00:00:00	22	44	2	2	249	west wind	951	30	0	50
2021-05-30 01:00:00	20.8	48	2.1	2	257	west wind	951	30	0	73
2021-05-30 02:00:00	19.9	51	1.4	1	254	west wind	951	30	0	68
2021-05-30 03:00:00	19.3	52	2.2	2	252	west wind	950	30	0	0
2021-05-30 04:00:00	18.4	55	2.3	2	264	west wind	950	30	0	0
2021-05-30 05:00:00	17.8	56	2	2	264	west wind	950	30	0	0
2021-05-30 06:00:00	17	56	1.7	2	261	west wind	950	30	0	0
2021-05-30 07:00:00	16.8	55	1.9	2	262	west wind	951	30	0	0
2021-05-30 08:00:00	18.9	50	2.4	2	264	west wind	951	30	0	0
2021-05-30 09:00:00	21.9	44	2.1	2	232	southwest wind	951	30	0	0
2021-05-30 10:00:00	24.6	40	2.1	2	259	west wind	951	30	0	0
2021-05-30 11:00:00	27.6	28	1	1	111	east wind	951	29.1	0	0
2021-05-30 12:00:00	28.9	27	1.5	1	193	south wind	950	30	0	0
2021-05-30 13:00:00	29.8	23	2.7	2	177	south wind	950	30	0	0
2021-05-30 14:00:00	31.2	20	2.5	2	93	east wind	949	30	0	0
2021-05-30 15:00:00	31.3	18	2.2	2	67	northeast wind	949	30	0	0
2021-05-30 16:00:00	32.6	20	1.6	2	105	east wind	948	30	0	0
2021-05-30 17:00:00	33.1	17	1.5	1	26	northeast wind	948	30	0	0
2021-05-30 18:00:00	33.1	16	1.4	1	173	south wind	947	30	0	0
2021-05-30 19:00:00	32.6	17	1.7	2	49	northeast wind	947	30	0	0
2021-05-30 20:00:00	32.3	23	0.8	1	65	northeast wind	947	28.9	0	0
2021-05-30 21:00:00	30.4	26	0.9	1	79	east wind	947	24.4	0	0
2021-05-30 22:00:00	25.7	37	1	1	357	north wind	948	13.1	0	0
2021-05-30 23:00:00	23.2	45	0.9	1	267	west wind	948	12.9	0	0
2021-05-31 00:00:00	21.2	50	0.9	1	317	northwest wind	949	18.4	0	0
2021-05-31 01:00:00	19.7	50	1.3	1	252	west wind	949	23.7	0	0
2021-05-31 02:00:00	18.5	52	1	1	293	northwest wind	949	30	0	0
2021-05-31 03:00:00	17.6	57	1.8	2	245	southwest wind	949	30	0	0
2021-05-31 04:00:00	17.6	55	1.8	2	257	west wind	948	30	0	37
2021-05-31 05:00:00	17.8	51	1.7	2	265	west wind	948	30	0	0
2021-05-31 06:00:00	16.9	57	1.7	2	257	west wind	948	30	0	0
2021-05-31 07:00:00	16.9	56	1.5	1	269	west wind	948	27	0	0
2021-05-31 08:00:00	18.7	51	1.9	2	244	southwest wind	948	30	0	0

2021-05-31 09:00:00	21.4	49	2.3	2	258	west wind	948	28.7	0	0
2021-05-31 10:00:00	26.1	30	1.2	1	288	west wind	948	25.1	0	0
2021-05-31 11:00:00	28.5	25	1.2	1	329	northwest wind	948	29.9	0	0
2021-05-31 12:00:00	29.3	21	1.3	1	114	southeast wind	948	30	0	0
2021-05-31 13:00:00	30	22	1.8	2	76	east wind	947	30	0	38
2021-05-31 14:00:00	31.2	21	1.7	2	32	northeast wind	946	30	0	0
2021-05-31 15:00:00	31.9	20	1.6	2	340	north wind	946	30	0	70
2021-05-31 16:00:00	32.2	19	1.8	2	84	east wind	945	30	0	94
2021-05-31 17:00:00	32.7	21	1.9	2	139	southeast wind	945	25.2	0	93
2021-05-31 18:00:00	32.3	21	2.7	2	133	southeast wind	945	23.8	0	94
2021-05-31 19:00:00	31.5	20	1.9	2	107	east wind	945	23.6	0	90
2021-05-31 20:00:00	29.6	24	5.3	3	267	west wind	946	23.7	0	85
2021-05-31 21:00:00	27.1	28	4.4	3	259	west wind	946	30	0	62
2021-05-31 22:00:00	26.1	30	3.7	3	274	west wind	946	30	0	0
2021-05-31 23:00:00	25.7	31	2.6	2	272	west wind	947	30	0	0
2021-06-01 00:00:00	25.2	33	3.2	2	272	west wind	947	30	0	64
2021-06-01 01:00:00	23.9	36	1.7	2	261	west wind	946	30	0	58
2021-06-01 02:00:00	21.4	43	1.6	2	198	south wind	946	30	0	41
2021-06-01 03:00:00	20.4	47	1.3	1	227	southwest wind	946	30	0	0
2021-06-01 04:00:00	19.6	49	1.1	1	218	southwest wind	946	30	0	0
2021-06-01 05:00:00	19.1	49	1.2	1	264	west wind	946	30	0	0
2021-06-01 06:00:00	17.3	57	0.8	1	302	northwest wind	946	30	0	0
2021-06-01 07:00:00	19.1	47	2.9	2	244	southwest wind	947	30	0	0
2021-06-01 08:00:00	20.5	46	2.3	2	254	west wind	947	30	0	0
2021-06-01 09:00:00	23.3	41	2.2	2	250	west wind	947	30	0	0
2021-06-01 10:00:00	26.3	34	2	2	261	west wind	947	30	0	0
2021-06-01 11:00:00	29.7	24	1.3	1	239	southwest wind	947	30	0	0
2021-06-01 12:00:00	31.4	19	2	2	62	northeast wind	946	30	0	0
2021-06-01 13:00:00	31.6	19	2.9	2	85	east wind	946	30	0	0
2021-06-01 14:00:00	32.3	19	2.8	2	82	east wind	946	30	0	0
2021-06-01 15:00:00	33	15	1.7	2	309	northwest wind	945	30	0	0
2021-06-01 16:00:00	33.9	17	1.6	2	288	west wind	945	30	0	0
2021-06-01 17:00:00	33.8	16	2.3	2	9	north wind	945	30	0	0
2021-06-01 18:00:00	34	13	3	2	71	east wind	944	30	0	0
2021-06-01 19:00:00	33.7	16	3	2	42	northeast wind	944	22.2	0	0
2021-06-01 20:00:00	32	19	3.1	2	5	north wind	945	30	0	0
2021-06-01 21:00:00	29.9	22	1.1	1	22	north wind	945	22.5	0	0
2021-06-01 22:00:00	26.9	27	1	1	19	north wind	945	21.8	0	0

2021-06-01 23:00:00	25.2	32	1.5	1	233	southwest wind	946	30	0	75
2021-06-02 00:00:00	24.2	34	1.2	1	252	west wind	946	23.1	0	73
2021-06-02 01:00:00	22	38	1.6	2	214	southwest wind	947	30	0	65
2021-06-02 02:00:00	22.3	37	1.8	2	262	west wind	947	30	0	47
2021-06-02 03:00:00	21.6	41	1.8	2	257	west wind	947	30	0	36
2021-06-02 04:00:00	20.6	43	1.9	2	254	west wind	947	30	0	0
2021-06-02 05:00:00	19.7	47	1.5	1	251	west wind	947	30	0	44
2021-06-02 06:00:00	19.4	47	1.7	2	244	southwest wind	947	30	0	0
2021-06-02 07:00:00	19.3	47	2.5	2	256	west wind	947	23.5	0	0
2021-06-02 08:00:00	21.3	45	2.6	2	253	west wind	948	27.7	0	0
2021-06-02 09:00:00	24.9	38	2.8	2	254	west wind	948	30	0	0
2021-06-02 10:00:00	28.4	29	2.2	2	261	west wind	948	30	0	0
2021-06-02 11:00:00	31.2	22	1.1	1	262	west wind	948	30	0	0
2021-06-02 12:00:00	33.1	20	0.9	1	158	south wind	947	30	0	0
2021-06-02 13:00:00	33.6	18	1.6	2	33	northeast wind	947	30	0	0
2021-06-02 14:00:00	33.9	17	2.3	2	37	northeast wind	947	30	0	0
2021-06-02 15:00:00	34.5	16	2.2	2	79	east wind	946	30	0	0
2021-06-02 16:00:00	35.1	15	1.9	2	53	northeast wind	945	30	0	0
2021-06-02 17:00:00	35.1	16	2.3	2	41	northeast wind	945	30	0	0
2021-06-02 18:00:00	35.7	13	1.2	1	118	southeast wind	944	30	0	0
2021-06-02 19:00:00	35.4	15	2	2	101	east wind	944	30	0	0
2021-06-02 20:00:00	34.7	14	2	2	66	northeast wind	945	25.8	0	0
2021-06-02 21:00:00	30.6	29	0.9	1	14	north wind	945	16.4	0	0
2021-06-02 22:00:00	27.2	38	1	1	270	west wind	945	13.9	0	0
2021-06-02 23:00:00	24.7	41	1.2	1	284	west wind	946	24.5	0	53
2021-06-03 00:00:00	23.1	44	1.4	1	252	west wind	946	26.1	0	0
2021-06-03 01:00:00	23.1	41	2	2	251	west wind	946	28.4	0	0
2021-06-03 02:00:00	22.5	42	1.2	1	263	west wind	946	30	0	0
2021-06-03 03:00:00	22.3	40	2.3	2	256	west wind	946	30	0	0
2021-06-03 04:00:00	21.9	39	1.9	2	260	west wind	946	30	0	0
2021-06-03 05:00:00	21	43	2.2	2	257	west wind	946	30	0	0
2021-06-03 06:00:00	22.1	38	2.6	2	265	west wind	946	30	0	0
2021-06-03 07:00:00	21.1	42	2.4	2	258	west wind	946	30	0	0
2021-06-03 08:00:00	23.3	40	1.9	2	259	west wind	946	30	0	0
2021-06-03 09:00:00	26.4	36	2.6	2	262	west wind	946	30	0	39
2021-06-03 10:00:00	28.6	31	2.6	2	255	west wind	946	26.8	0	0
2021-06-03 11:00:00	31.2	26	2.1	2	328	northwest wind	946	25.9	0	0
2021-06-03 12:00:00	31.9	26	2.7	2	55	northeast wind	946	30	0	0

2021-06-03 13:00:00	32.4	24	2.7	2	69	east wind	945	30	0	0
2021-06-03 14:00:00	33.9	22	2	2	110	east wind	945	30	0	0
2021-06-03 15:00:00	35.1	17	1.7	2	106	east wind	944	27.8	0	0
2021-06-03 16:00:00	34.2	20	1.6	2	154	southeast wind	943	30	0	92
2021-06-03 17:00:00	35.8	16	1.5	1	91	east wind	943	30	0	87
2021-06-03 18:00:00	35.7	17	2.2	2	355	north wind	942	30	0	81
2021-06-03 19:00:00	34.8	19	2.4	2	97	east wind	942	21.2	0	0
2021-06-03 20:00:00	33.7	20	2.8	2	84	east wind	943	30	0	72
2021-06-03 21:00:00	32.2	22	2	2	1	north wind	943	12.3	0	49
2021-06-03 22:00:00	28.7	32	0.9	1	77	east wind	944	21.7	0	78
2021-06-03 23:00:00	29.9	26	2.3	2	260	west wind	945	30	0	90
2021-06-04 00:00:00	27.8	32	1.5	1	228	southwest wind	945	30	0	61
2021-06-04 01:00:00	27.8	32	1.8	2	263	west wind	945	30	0	82
2021-06-04 02:00:00	27.1	34	1.3	1	339	north wind	945	30	0	65
2021-06-04 03:00:00	25.5	39	1.6	2	190	south wind	945	30	0	86
2021-06-04 04:00:00	24.6	40	1.1	1	241	southwest wind	945	30	0	74
2021-06-04 05:00:00	23.3	45	1.5	1	258	west wind	945	30	0	69
2021-06-04 06:00:00	22.2	48	2	2	262	west wind	945	30	0	73
2021-06-04 07:00:00	21.7	48	1.9	2	248	west wind	946	30	0	61
2021-06-04 08:00:00	22.7	45	1.8	2	247	southwest wind	946	30	0	52
2021-06-04 09:00:00	25.3	43	1.5	1	244	southwest wind	946	25.5	0	0
2021-06-04 10:00:00	29.8	30	1.8	2	245	southwest wind	946	30	0	0
2021-06-04 11:00:00	32.3	25	0.8	1	53	northeast wind	945	30	0	0
2021-06-04 12:00:00	32.3	23	2.2	2	113	southeast wind	945	30	0	29
2021-06-04 13:00:00	33	22	2.9	2	90	east wind	945	30	0	0
2021-06-04 14:00:00	31.7	24	2.9	2	350	north wind	945	30	0	0
2021-06-04 15:00:00	26.4	42	1.1	1	144	southeast wind	945	26.4	0.3	65
2021-06-04 16:00:00	27	52	0.7	1	134	southeast wind	945	26.6	0	38
2021-06-04 17:00:00	28.7	42	0.6	1	137	southeast wind	945	30	0	94
2021-06-04 18:00:00	30.7	32	1.9	2	67	northeast wind	944	30	0	94
2021-06-04 19:00:00	29	36	2	2	272	west wind	945	30	0	88
2021-06-04 20:00:00	25.1	51	3.7	3	233	southwest wind	945	26.3	0.2	43
2021-06-04 21:00:00	25.6	54	1.5	1	277	west wind	946	30	0	84
2021-06-04 22:00:00	24.5	56	0.6	1	340	north wind	946	30	0	88
2021-06-04 23:00:00	23.1	63	0.8	1	200	south wind	946	25.2	0	54
2021-06-05 00:00:00	21.4	71	0.4	1	300	northwest wind	946	30	0	0
2021-06-05 01:00:00	20.3	72	0.9	1	268	west wind	946	30	0	0
2021-06-05 02:00:00	19.3	79	1.9	2	256	west wind	946	30	0	71

2021-06-05 03:00:00	19.1	72	1.5	1	253	west wind	946	30	0	0
2021-06-05 04:00:00	19.3	65	1.7	2	251	west wind	945	30	0	0
2021-06-05 05:00:00	20.1	56	1.9	2	260	west wind	945	30	0	69
2021-06-05 06:00:00	19.6	58	1.7	2	245	southwest wind	945	30	0	74
2021-06-05 07:00:00	18.1	68	1.4	1	253	west wind	946	30	0	60
2021-06-05 08:00:00	20.3	60	1.4	1	305	northwest wind	945	30	0	39
2021-06-05 09:00:00	24.2	48	1.3	1	282	west wind	946	30	0	45
2021-06-05 10:00:00	26.5	44	0.9	1	275	west wind	946	27	0	82
2021-06-05 11:00:00	26.1	40	1.4	1	82	east wind	946	30	0	33
2021-06-05 12:00:00	26.7	39	2	2	104	east wind	945	30	0	92
2021-06-05 13:00:00	28.5	32	2.5	2	94	east wind	945	30	0	87
2021-06-05 14:00:00	29.1	33	3	2	85	east wind	945	17.6	0	86
2021-06-05 15:00:00	25.1	49	2.7	2	108	east wind	945	28.9	0.1	95
2021-06-05 16:00:00	26.4	38	4.3	3	93	east wind	945	26.9	0.2	94
2021-06-05 17:00:00	26.6	37	5	3	113	southeast wind	945	25.6	0	60
2021-06-05 18:00:00	25.5	38	3.1	2	96	east wind	946	22.4	0	0
2021-06-05 19:00:00	24.6	38	4.1	3	125	southeast wind	946	21.2	0	0
2021-06-05 20:00:00	23.6	42	3.7	3	157	southeast wind	947	21.6	0	0
2021-06-05 21:00:00	22.3	42	3.3	2	147	southeast wind	948	15.1	0	32
2021-06-05 22:00:00	20.8	41	3.5	3	118	southeast wind	948	14.8	0	0
2021-06-05 23:00:00	19.4	38	3.2	2	103	east wind	949	17.6	0	0
2021-06-06 00:00:00	18.5	39	1.3	1	153	southeast wind	950	25.9	0	0
2021-06-06 01:00:00	17.7	40	2	2	80	east wind	951	30	0	0
2021-06-06 02:00:00	17.1	40	3.4	3	151	southeast wind	951	24.2	0	24
2021-06-06 03:00:00	16.4	42	1.6	2	155	southeast wind	951	21.5	0	0
2021-06-06 04:00:00	15.5	43	2.2	2	154	southeast wind	951	17.8	0	33
2021-06-06 05:00:00	14.2	51	0.5	1	149	southeast wind	951	18.4	0	0
2021-06-06 06:00:00	14.7	49	1.4	1	121	southeast wind	952	20.7	0	0
2021-06-06 07:00:00	14.9	50	1.1	1	199	south wind	952	20	0	0
2021-06-06 08:00:00	15.1	53	1.1	1	247	southwest wind	953	19	0	55
2021-06-06 09:00:00	15.8	50	1.4	1	182	south wind	953	18.2	0	0
2021-06-06 10:00:00	17.4	42	0.8	1	84	east wind	953	24.4	0	0
2021-06-06 11:00:00	18.7	38	1.4	1	337	northwest wind	953	22.6	0	0
2021-06-06 12:00:00	20.2	38	1.8	2	85	east wind	953	21.3	0	0
2021-06-06 13:00:00	21.3	37	3.1	2	111	east wind	952	22.6	0	0
2021-06-06 14:00:00	22.2	32	1.8	2	17	north wind	951	26.3	0	0
2021-06-06 15:00:00	23.6	33	3	2	137	southeast wind	950	30	0	0
2021-06-06 16:00:00	23.8	31	2.9	2	80	east wind	950	24.2	0	0

2021-06-06 17:00:00	24.1	28	2.3	2	108	east wind	949	25.4	0	0
2021-06-06 18:00:00	25	28	1.8	2	102	east wind	949	26.7	0	0
2021-06-06 19:00:00	24.8	26	2.6	2	94	east wind	949	22.9	0	0
2021-06-06 20:00:00	24.3	27	1.8	2	96	east wind	949	24	0	0
2021-06-06 21:00:00	23.4	29	1.8	2	91	east wind	949	25.7	0	0
2021-06-06 22:00:00	21.2	37	0.8	1	341	north wind	949	17.3	0	0
2021-06-06 23:00:00	21	38	0.9	1	280	west wind	950	18.6	0	0
2021-06-07 00:00:00	18.9	52	0.2	0	0	north wind	950	20.8	0	0
2021-06-07 01:00:00	17	60	0.7	1	252	west wind	950	23.7	0	0
2021-06-07 02:00:00	15.8	64	0.7	1	250	west wind	949	26.7	0	0
2021-06-07 03:00:00	15.3	62	1.2	1	261	west wind	949	26.3	0	0
2021-06-07 04:00:00	14.9	61	1	1	257	west wind	949	25.6	0	0
2021-06-07 05:00:00	14	68	1.2	1	257	west wind	949	25.9	0	0
2021-06-07 06:00:00	13.5	70	1.5	1	262	west wind	949	24.3	0	0
2021-06-07 07:00:00	13.6	70	2.1	2	248	west wind	950	24.6	0	28
2021-06-07 08:00:00	16	62	2.5	2	257	west wind	950	22.8	0	33
2021-06-07 09:00:00	18.7	54	2	2	254	west wind	950	26	0	0
2021-06-07 10:00:00	21.9	33	1.8	2	31	northeast wind	950	30	0	0
2021-06-07 11:00:00	22.6	34	2.2	2	76	east wind	950	30	0	0
2021-06-07 12:00:00	23.3	32	2.8	2	1	north wind	949	30	0	68
2021-06-07 13:00:00	24.5	31	2.8	2	37	northeast wind	949	30	0	0
2021-06-07 14:00:00	24.4	31	2.5	2	78	east wind	948	24	0	0
2021-06-07 15:00:00	26.1	32	2.1	2	61	northeast wind	947	25.7	0	0
2021-06-07 16:00:00	25.8	33	2.1	2	91	east wind	947	25.8	0	74
2021-06-07 17:00:00	26.5	31	1.8	2	82	east wind	947	27.1	0	45
2021-06-07 18:00:00	26.1	33	1.5	1	116	southeast wind	947	25.6	0	91
2021-06-07 19:00:00	25.6	37	1.2	1	191	south wind	947	27.4	0	91
2021-06-07 20:00:00	26.3	34	1.4	1	187	south wind	946	27.6	0	79
2021-06-07 21:00:00	25.4	43	0.5	1	140	southeast wind	946	20.8	0	0
2021-06-07 22:00:00	22.5	50	0.5	1	156	southeast wind	946	5.5	0	34
2021-06-07 23:00:00	21.2	52	1.3	1	323	northwest wind	946	17.1	0	0
2021-06-08 00:00:00	19.5	59	1.1	1	273	west wind	946	24.9	0	0
2021-06-08 01:00:00	17.7	69	0.7	1	221	southwest wind	945	23.8	0	0
2021-06-08 02:00:00	17.7	67	1.2	1	258	west wind	945	23	0	0
2021-06-08 03:00:00	17.5	68	1.6	2	252	west wind	945	27.4	0	0
2021-06-08 04:00:00	17.3	68	2.1	2	268	west wind	944	29	0	0
2021-06-08 05:00:00	16.8	67	1.2	1	258	west wind	945	28.8	0	0
2021-06-08 06:00:00	16.3	71	2	2	246	southwest wind	945	27.5	0	77

2021-06-08 07:00:00	16.1	73	1.9	2	255	west wind	945	25.6	0	87
2021-06-08 08:00:00	17.4	68	2.1	2	269	west wind	946	24.5	0	82
2021-06-08 09:00:00	18.7	60	1.1	1	255	west wind	946	23.5	0	91
2021-06-08 10:00:00	21.5	56	1.7	2	257	west wind	945	19.5	0	56
2021-06-08 11:00:00	25.6	30	1.2	1	44	northeast wind	945	30	0	0
2021-06-08 12:00:00	26.5	23	2.9	2	76	east wind	944	28.5	0	0
2021-06-08 13:00:00	27	29	3.8	3	67	northeast wind	944	25.2	0	0
2021-06-08 14:00:00	27.5	30	3.7	3	79	east wind	943	27.3	0	0
2021-06-08 15:00:00	28.7	30	3.2	2	80	east wind	943	27.2	0	0
2021-06-08 16:00:00	29.3	29	2.8	2	65	northeast wind	942	25.6	0	92
2021-06-08 17:00:00	29.9	26	2.2	2	58	northeast wind	943	30	0	0
2021-06-08 18:00:00	30.3	24	2.3	2	120	southeast wind	942	30	0	0
2021-06-08 19:00:00	30.8	27	1.3	1	118	southeast wind	942	24.8	0	50
2021-06-08 20:00:00	29.9	26	2.4	2	132	southeast wind	943	30	0	36
2021-06-08 21:00:00	29.2	28	1	1	3	north wind	943	24.7	0	76
2021-06-08 22:00:00	25.8	41	0.6	1	280	west wind	943	11.7	0	0
2021-06-08 23:00:00	23.5	48	1.1	1	297	northwest wind	944	20.6	0	59
2021-06-09 00:00:00	22	56	0.5	1	27	northeast wind	944	20.7	0	65
2021-06-09 01:00:00	21.4	57	1.2	1	263	west wind	945	25.3	0	49
2021-06-09 02:00:00	21.2	54	1.3	1	246	southwest wind	945	29.9	0	54
2021-06-09 03:00:00	23.3	43	2	2	268	west wind	945	29.1	0	46
2021-06-09 04:00:00	23	42	2	2	241	southwest wind	946	30	0	87
2021-06-09 05:00:00	22.6	42	1.2	1	140	southeast wind	946	30	0	88
2021-06-09 06:00:00	23.2	41	1	1	207	southwest wind	946	15	0	37
2021-06-09 07:00:00	24.5	33	2.8	2	262	west wind	947	30	0	88
2021-06-09 08:00:00	23.9	35	3.5	3	268	west wind	947	30	0	68
2021-06-09 09:00:00	24.6	37	2.8	2	243	southwest wind	947	30	0	36
2021-06-09 10:00:00	25.1	39	1.7	2	251	west wind	946	30	0	91
2021-06-09 11:00:00	28.2	30	1	1	159	south wind	946	30	0	0
2021-06-09 12:00:00	29.4	25	1.5	1	171	south wind	945	30	0	0
2021-06-09 13:00:00	28.4	32	2.6	2	215	southwest wind	946	30	0	0
2021-06-09 14:00:00	30.5	25	1.7	2	349	north wind	945	30	0	88
2021-06-09 15:00:00	29.2	24	5.9	4	279	west wind	945	30	0	0
2021-06-09 16:00:00	28.8	25	2.8	2	345	north wind	945	30	0	90
2021-06-09 17:00:00	29.4	25	2.4	2	244	southwest wind	945	30	0	54
2021-06-09 18:00:00	29.8	26	1.6	2	221	southwest wind	945	27.9	0	78
2021-06-09 19:00:00	27.5	34	2.9	2	326	northwest wind	946	30	0	32
2021-06-09 20:00:00	27	39	2.2	2	357	north wind	946	10	0	58

2021-06-09 21:00:00	24.2	40	5.8	4	162	south wind	947	30	0	37
2021-06-09 22:00:00	23.7	43	2.7	2	268	west wind	947	30	0	88
2021-06-09 23:00:00	21.7	53	2.3	2	180	south wind	946	30	0	77
2021-06-10 00:00:00	23.8	34	3.9	3	240	southwest wind	946	30	0	61
2021-06-10 01:00:00	21.1	45	1.7	2	250	west wind	946	30	0	0
2021-06-10 02:00:00	22.3	39	2.6	2	253	west wind	946	30	0	38
2021-06-10 03:00:00	20.1	48	1.8	2	258	west wind	946	30	0	45
2021-06-10 04:00:00	19.5	46	3	2	266	west wind	945	30	0	0
2021-06-10 05:00:00	17.3	54	1.5	1	197	south wind	945	30	0	0
2021-06-10 06:00:00	17.3	53	1.6	2	263	west wind	945	30	0	0
2021-06-10 07:00:00	16.8	56	1.4	1	230	southwest wind	945	30	0	0
2021-06-10 08:00:00	18.4	55	1.6	2	251	west wind	945	30	0	59
2021-06-10 09:00:00	20.8	52	1.7	2	248	west wind	945	27.4	0	63
2021-06-10 10:00:00	21.9	55	0.7	1	280	west wind	945	30	0	66
2021-06-10 11:00:00	23	58	0.7	1	309	northwest wind	945	24.5	0	71
2021-06-10 12:00:00	24.7	47	1.3	1	33	northeast wind	945	26.5	0	93
2021-06-10 13:00:00	27.5	37	2.3	2	69	east wind	944	30	0	0
2021-06-10 14:00:00	29	30	2.4	2	72	east wind	943	30	0	0
2021-06-10 15:00:00	29.3	30	2	2	98	east wind	943	30	0	0
2021-06-10 16:00:00	29.6	27	2	2	63	northeast wind	943	24.4	0	94
2021-06-10 17:00:00	29.7	32	0.6	1	334	northwest wind	943	30	0	94
2021-06-10 18:00:00	28.5	31	2.1	2	359	north wind	943	30	0	94
2021-06-10 19:00:00	28.8	29	2	2	51	northeast wind	944	23.5	0	89
2021-06-10 20:00:00	27.4	33	1.1	1	129	southeast wind	944	23.1	0	90
2021-06-10 21:00:00	25	39	1.8	2	354	north wind	944	24.5	0	80
2021-06-10 22:00:00	23.9	42	1	1	316	northwest wind	945	30	0	89
2021-06-10 23:00:00	23.4	43	0.7	1	245	southwest wind	945	30	0	83
2021-06-11 00:00:00	23.3	43	3.3	2	228	southwest wind	946	30	0	88
2021-06-11 01:00:00	20.2	63	1.7	2	308	northwest wind	946	30	0	87
2021-06-11 02:00:00	20.4	68	1.1	1	42	northeast wind	946	30	0.1	89
2021-06-11 03:00:00	19.6	65	1.3	1	274	west wind	946	30	0	89
2021-06-11 04:00:00	19.1	67	1.7	2	222	southwest wind	946	30	0	90
2021-06-11 05:00:00	19	69	1.9	2	276	west wind	946	30	0	91
2021-06-11 06:00:00	18.1	71	0.9	1	264	west wind	945	30	0	67
2021-06-11 07:00:00	17.2	75	1.3	1	265	west wind	945	30	0	84
2021-06-11 08:00:00	18	74	1.7	2	266	west wind	946	30	0	0
2021-06-11 09:00:00	19.4	70	1.6	2	250	west wind	946	30	0	0
2021-06-11 10:00:00	22.9	52	1	1	222	southwest wind	946	30	0	0

2021-06-11 11:00:00	24.7	42	1.8	2	135	southeast wind	945	30	0	0
2021-06-11 12:00:00	25.9	38	1.6	2	105	east wind	944	2.3	0	0
2021-06-11 13:00:00	26.2	40	2.4	2	76	east wind	944	30	0	0
2021-06-11 14:00:00	27	38	2.3	2	79	east wind	943	30	0	0
2021-06-11 15:00:00	25.7	38	1	1	235	southwest wind	944	19.9	0	89
2021-06-11 16:00:00	22.4	56	1.7	2	1	north wind	944	30	0	93
2021-06-11 17:00:00	24	42	3.5	3	77	east wind	945	30	0	58
2021-06-11 18:00:00	24.4	42	2.5	2	89	east wind	945	30	0	0
2021-06-11 19:00:00	23.3	50	4.3	3	121	southeast wind	945	30	0	26
2021-06-11 20:00:00	22.4	51	1.8	2	177	south wind	946	30	0	0
2021-06-11 21:00:00	20.4	57	1.4	1	284	west wind	948	30	0	87
2021-06-11 22:00:00	19.5	63	1.8	2	257	west wind	948	19.9	0	83
2021-06-11 23:00:00	18	70	1.3	1	329	northwest wind	949	30	0	83
2021-06-12 00:00:00	17.5	73	1.3	1	345	north wind	949	27.2	0	35
2021-06-12 01:00:00	16.5	76	1.2	1	265	west wind	949	30	0	0
2021-06-12 02:00:00	15.8	79	1.4	1	260	west wind	948	30	0	0
2021-06-12 03:00:00	15.1	81	1.2	1	256	west wind	948	30	0	0
2021-06-12 04:00:00	13.7	87	0.5	1	190	south wind	948	30	0	0
2021-06-12 05:00:00	13.5	86	1.4	1	245	southwest wind	948	30	0	0
2021-06-12 06:00:00	13.5	85	1.5	1	254	west wind	949	30	0	0
2021-06-12 07:00:00	13.2	86	1.4	1	259	west wind	949	30	0	0
2021-06-12 08:00:00	15.4	80	0.9	1	294	northwest wind	949	26.9	0	0
2021-06-12 09:00:00	19.1	66	1.1	1	154	southeast wind	949	25	0	0
2021-06-12 10:00:00	20.3	52	2.4	2	137	southeast wind	949	30	0	0
2021-06-12 11:00:00	22.1	35	2.5	2	91	east wind	949	30	0	0
2021-06-12 12:00:00	23	37	1.8	2	99	east wind	949	28.8	0	0
2021-06-12 13:00:00	23.6	39	2.4	2	106	east wind	949	30	0	31
2021-06-12 14:00:00	23.8	37	3	2	103	east wind	948	30	0	0
2021-06-12 15:00:00	24.5	34	3.8	3	67	northeast wind	948	30	0	0
2021-06-12 16:00:00	24	35	2	2	125	southeast wind	947	19.2	0	91
2021-06-12 17:00:00	24.2	33	1.7	2	151	southeast wind	947	30	0	42
2021-06-12 18:00:00	25.3	31	1.2	1	319	northwest wind	947	30	0.1	76
2021-06-12 19:00:00	24.1	32	2.1	2	284	west wind	947	30	0	46
2021-06-12 20:00:00	23.3	37	1.5	1	281	west wind	947	30	0	0
2021-06-12 21:00:00	21.9	43	1.6	2	255	west wind	948	30	0	25
2021-06-12 22:00:00	18	70	1.7	2	325	northwest wind	949	30	0.8	38
2021-06-12 23:00:00	17.3	72	1.1	1	114	southeast wind	950	30	0.3	82
2021-06-13 00:00:00	17	76	1.4	1	266	west wind	950	30	0.1	90

2021-06-13 01:00:00	17.9	46	3.4	3	110	east wind	950	30	0	43
2021-06-13 02:00:00	16.8	49	3.5	3	118	southeast wind	950	30	0	0
2021-06-13 03:00:00	16.2	49	2.1	2	135	southeast wind	950	30	0	82
2021-06-13 04:00:00	15.8	56	1	1	330	northwest wind	951	30	0	82
2021-06-13 05:00:00	16.2	46	1.4	1	4	north wind	951	30	0	82
2021-06-13 06:00:00	16.3	40	2.2	2	86	east wind	951	30	0	82
2021-06-13 07:00:00	16.2	40	2.1	2	96	east wind	951	30	0	0
2021-06-13 08:00:00	16.7	35	2.4	2	99	east wind	952	30	0	0
2021-06-13 09:00:00	17.4	33	3.9	3	106	east wind	952	30	0	0
2021-06-13 10:00:00	18.3	36	2.9	2	105	east wind	952	30	0	33
2021-06-13 11:00:00	20.1	31	3.3	2	94	east wind	952	30	0	38
2021-06-13 12:00:00	20.2	31	3	2	119	southeast wind	952	30	0	0
2021-06-13 13:00:00	20.8	31	2.7	2	87	east wind	951	30	0	0
2021-06-13 14:00:00	21.8	32	2	2	84	east wind	950	30	0	0
2021-06-13 15:00:00	22.3	31	1.8	2	102	east wind	950	30	0	0
2021-06-13 16:00:00	23.6	32	2.7	2	353	north wind	949	30	0	0
2021-06-13 17:00:00	22.1	29	2.4	2	24	northeast wind	949	30	0	0
2021-06-13 18:00:00	22.4	29	1.2	1	331	northwest wind	949	30	0	0
2021-06-13 19:00:00	21.1	33	4.3	3	297	northwest wind	950	30	0	28
2021-06-13 20:00:00	20.3	32	4.3	3	297	northwest wind	950	30	0	51
2021-06-13 21:00:00	18.2	41	3.9	3	293	northwest wind	952	30	0	0
2021-06-13 22:00:00	16.9	45	2.4	2	256	west wind	953	30	0	0
2021-06-13 23:00:00	15.8	48	2.3	2	246	southwest wind	954	30	0	0
2021-06-14 00:00:00	15.7	48	2.2	2	257	west wind	955	30	0	0
2021-06-14 01:00:00	15.6	47	1.5	1	310	northwest wind	955	30	0	0
2021-06-14 02:00:00	14.6	54	0.8	1	355	north wind	955	30	0	0
2021-06-14 03:00:00	13.9	60	1	1	120	southeast wind	955	30	0	26
2021-06-14 04:00:00	14.1	58	1.5	1	256	west wind	955	30	0	43
2021-06-14 05:00:00	13.1	61	1.3	1	271	west wind	955	30	0	0
2021-06-14 06:00:00	11.4	72	1.5	1	255	west wind	955	30	0	0
2021-06-14 07:00:00	10.7	82	2	2	262	west wind	955	30	0	0
2021-06-14 08:00:00	12.7	74	2.6	2	262	west wind	955	30	0	0
2021-06-14 09:00:00	15.5	61	2.8	2	254	west wind	955	30	0	0
2021-06-14 10:00:00	17.8	50	2.1	2	259	west wind	955	30	0	0
2021-06-14 11:00:00	19.6	36	1.3	1	154	southeast wind	954	30	0	0
2021-06-14 12:00:00	21.1	31	1.6	2	123	southeast wind	954	30	0	0
2021-06-14 13:00:00	22.2	31	2.6	2	130	southeast wind	954	30	0	0
2021-06-14 14:00:00	22.8	25	1.7	2	108	east wind	953	30	0	0

2021-06-14 15:00:00	23.7	24	1.4	1	291	west wind	953	30	0	0
2021-06-14 16:00:00	23.7	24	2.6	2	60	northeast wind	952	30	0	0
2021-06-14 17:00:00	24.4	23	2	2	346	north wind	952	30	0	0
2021-06-14 18:00:00	24.9	23	1.3	1	282	west wind	951	30	0	0
2021-06-14 19:00:00	24.5	22	2.1	2	27	northeast wind	951	30	0	0
2021-06-14 20:00:00	24.6	22	1.3	1	84	east wind	951	30	0	0
2021-06-14 21:00:00	23.8	20	2	2	94	east wind	952	30	0	0
2021-06-14 22:00:00	22.5	23	1.2	1	81	east wind	952	20.6	0	0
2021-06-14 23:00:00	19.4	34	1.2	1	316	northwest wind	953	30	0	0
2021-06-15 00:00:00	17.8	39	1.2	1	282	west wind	953	30	0	0
2021-06-15 01:00:00	16.9	41	1.4	1	270	west wind	953	30	0	0
2021-06-15 02:00:00	14.1	55	0.9	1	206	southwest wind	953	30	0	0
2021-06-15 03:00:00	14.3	60	1.6	2	247	southwest wind	952	30	0	0
2021-06-15 04:00:00	13.2	67	1.8	2	263	west wind	952	30	0	0
2021-06-15 05:00:00	13	66	1.8	2	258	west wind	952	30	0	0
2021-06-15 06:00:00	12.2	68	2.2	2	256	west wind	952	30	0	0
2021-06-15 07:00:00	12.5	66	2.4	2	251	west wind	952	30	0	0
2021-06-15 08:00:00	14.5	61	2.4	2	252	west wind	952	30	0	0
2021-06-15 09:00:00	18.2	50	2.3	2	245	southwest wind	952	30	0	0
2021-06-15 10:00:00	21.5	35	2	2	249	west wind	952	30	0	0
2021-06-15 11:00:00	23.4	28	1.6	2	238	southwest wind	952	30	0	0
2021-06-15 12:00:00	25.2	20	1.1	1	246	southwest wind	951	30	0	0
2021-06-15 13:00:00	25.8	23	1.7	2	89	east wind	951	30	0	0
2021-06-15 14:00:00	26.3	22	2.2	2	64	northeast wind	951	30	0	0
2021-06-15 15:00:00	27.2	20	1.7	2	36	northeast wind	950	30	0	0
2021-06-15 16:00:00	28.4	19	1	1	348	north wind	950	30	0	0
2021-06-15 17:00:00	28.4	19	1	1	348	north wind	950	30	0	0
2021-06-15 18:00:00	28.7	16	1.6	2	125	southeast wind	949	30	0	0
2021-06-15 19:00:00	28.4	15	2.3	2	105	east wind	948	30	0	0
2021-06-15 20:00:00	28.1	14	1.6	2	114	southeast wind	948	28.9	0	0
2021-06-15 21:00:00	26.8	15	1.4	1	98	east wind	949	17.8	0	0
2021-06-15 22:00:00	23.3	24	0.5	1	210	southwest wind	949	16.3	0	0
2021-06-15 23:00:00	19.8	36	1.4	1	313	northwest wind	949	30	0	0
2021-06-16 00:00:00	17.8	47	1	1	276	west wind	949	30	0	0
2021-06-16 01:00:00	16.1	54	1.7	2	256	west wind	950	30	0	0
2021-06-16 02:00:00	16.3	53	2.1	2	259	west wind	950	30	0	0
2021-06-16 03:00:00	15.5	55	2.1	2	253	west wind	950	30	0	0
2021-06-16 04:00:00	15.1	54	2	2	256	west wind	950	30	0	0

2021-06-16 05:00:00	14.3	57	2.1	2	254	west wind	950	30	0	0
2021-06-16 06:00:00	13.8	58	1.5	1	257	west wind	950	30	0	0
2021-06-16 07:00:00	13.6	60	1.8	2	246	southwest wind	951	30	0	0
2021-06-16 08:00:00	16.1	56	1.5	1	252	west wind	951	28.2	0	0
2021-06-16 09:00:00	19.2	50	1.5	1	248	west wind	951	30	0	0
2021-06-16 10:00:00	24.1	32	1	1	330	northwest wind	951	30	0	0
2021-06-16 11:00:00	26.2	26	2.2	2	73	east wind	951	30	0	0
2021-06-16 12:00:00	27.3	23	2.1	2	58	northeast wind	951	30	0	0
2021-06-16 13:00:00	27.4	20	2.6	2	78	east wind	950	30	0	0
2021-06-16 14:00:00	28.2	17	2.8	2	43	northeast wind	950	30	0	0
2021-06-16 15:00:00	28.8	19	2.6	2	59	northeast wind	950	30	0	0
2021-06-16 16:00:00	29.1	18	2.7	2	58	northeast wind	949	30	0	0
2021-06-16 17:00:00	29.4	19	2.7	2	100	east wind	949	30	0	0
2021-06-16 18:00:00	29.6	19	2.6	2	92	east wind	948	30	0	0
2021-06-16 19:00:00	29.5	16	2.3	2	83	east wind	948	28.6	0	0
2021-06-16 20:00:00	29.3	19	2.3	2	81	east wind	948	30	0	0
2021-06-16 21:00:00	28.4	19	1.4	1	80	east wind	948	25.2	0	0
2021-06-16 22:00:00	23.9	35	1.2	1	344	north wind	949	27.5	0	0
2021-06-16 23:00:00	20.7	47	0.5	1	280	west wind	949	23.2	0	0
2021-06-17 00:00:00	19.1	52	1.5	1	249	west wind	949	28	0	59
2021-06-17 01:00:00	18.5	48	1.7	2	256	west wind	949	30	0	62
2021-06-17 02:00:00	17.9	50	1.7	2	258	west wind	949	30	0	42
2021-06-17 03:00:00	17.2	50	1.9	2	260	west wind	949	30	0	55
2021-06-17 04:00:00	17.4	47	2.5	2	255	west wind	949	30	0	52
2021-06-17 05:00:00	16.9	48	1.9	2	248	west wind	949	30	0	29
2021-06-17 06:00:00	16.8	48	1.8	2	249	west wind	949	30	0	83
2021-06-17 07:00:00	16.2	52	2	2	254	west wind	949	30	0	53
2021-06-17 08:00:00	17	52	2.6	2	254	west wind	949	29.6	0	76
2021-06-17 09:00:00	19.9	50	1.8	2	241	southwest wind	949	30	0	65
2021-06-17 10:00:00	22.8	46	1.3	1	260	west wind	949	24.1	0	66
2021-06-17 11:00:00	26.3	34	1.1	1	290	west wind	949	30	0	75
2021-06-17 12:00:00	29.3	20	1.8	2	77	east wind	948	26	0	90
2021-06-17 13:00:00	28.5	26	3.3	2	78	east wind	948	30	0	80
2021-06-17 14:00:00	28.8	24	3.7	3	72	east wind	948	30	0	80
2021-06-17 15:00:00	29.2	22	4	3	72	east wind	947	30	0	80
2021-06-17 16:00:00	29.7	25	2.8	2	96	east wind	947	30	0	85
2021-06-17 17:00:00	29.7	21	2.8	2	69	east wind	946	30	0	75
2021-06-17 18:00:00	30.5	20	1.7	2	123	southeast wind	946	30	0	25

2021-06-17 19:00:00	30.9	18	1.3	1	122	southeast wind	946	30	0	0
2021-06-17 20:00:00	30.7	19	1.1	1	337	northwest wind	946	30	0	80
2021-06-17 21:00:00	28.6	26	1.3	1	255	west wind	946	22.3	0	80
2021-06-17 22:00:00	25.1	37	1.2	1	261	west wind	946	21.2	0	55
2021-06-17 23:00:00	21.2	52	1.2	1	182	south wind	947	18.7	0	40
2021-06-18 00:00:00	20.2	55	1.6	2	240	southwest wind	947	22.1	0	40
2021-06-18 01:00:00	19.8	53	1.7	2	266	west wind	947	30	0	40
2021-06-18 02:00:00	19	53	2.1	2	252	west wind	947	30	0	0
2021-06-18 03:00:00	17.9	54	1.9	2	252	west wind	946	30	0	35
2021-06-18 04:00:00	17.7	52	2.5	2	253	west wind	946	30	0	75
2021-06-18 05:00:00	18.4	47	2.9	2	260	west wind	946	30	0	80
2021-06-18 06:00:00	17.3	51	1.5	1	267	west wind	946	30	0	55
2021-06-18 07:00:00	16.6	56	1.5	1	234	southwest wind	947	30	0	60
2021-06-18 08:00:00	18.7	53	2.2	2	256	west wind	947	30	0	55
2021-06-18 09:00:00	21.2	47	2.4	2	252	west wind	947	28.3	0	0
2021-06-18 10:00:00	25.4	42	1.6	2	252	west wind	946	22.9	0	0
2021-06-18 11:00:00	29	25	0.9	1	12	north wind	946	30	0	0
2021-06-18 12:00:00	31.1	22	1.7	2	68	east wind	945	30	0	70
2021-06-18 13:00:00	30.9	20	1.8	2	68	east wind	945	30	0	70
2021-06-18 14:00:00	32.1	19	1.6	2	87	east wind	944	30	0	70
2021-06-18 15:00:00	32.1	20	2.7	2	102	east wind	944	30	0	90
2021-06-18 16:00:00	31.4	21	2.4	2	103	east wind	943	28.7	0	85
2021-06-18 17:00:00	31	22	1.9	2	99	east wind	943	30	0	85
2021-06-18 18:00:00	31.5	24	2.5	2	146	southeast wind	943	26.3	0	80
2021-06-18 19:00:00	32.1	25	1.1	1	144	southeast wind	943	30	0	80
2021-06-18 20:00:00	31.1	25	0.9	1	96	east wind	943	21.5	0	80
2021-06-18 21:00:00	27.8	39	1.3	1	7	north wind	943	26.6	0	40
2021-06-18 22:00:00	25.6	44	1.5	1	297	northwest wind	944	21.6	0	50
2021-06-18 23:00:00	23.4	53	1.1	1	289	west wind	944	25.5	0	45
2021-06-19 00:00:00	23	47	1.8	2	255	west wind	944	30	0	60
2021-06-19 01:00:00	24.1	36	3	2	260	west wind	944	30	0	75
2021-06-19 02:00:00	23.8	36	3.2	2	260	west wind	944	30	0	90
2021-06-19 03:00:00	23.9	35	2.6	2	255	west wind	943	30	0	75
2021-06-19 04:00:00	23.6	35	2.3	2	264	west wind	943	30	0	70
2021-06-19 05:00:00	24.7	31	2.7	2	258	west wind	943	30	0	60
2021-06-19 06:00:00	21.2	47	1.4	1	206	southwest wind	943	30	0	85
2021-06-19 07:00:00	22	41	1.5	1	228	southwest wind	943	30	0	80
2021-06-19 08:00:00	22.4	44	1.3	1	249	west wind	943	28.9	0	80

2021-06-19 09:00:00	23.2	46	1.7	2	238	southwest wind	943	22.5	0	70
2021-06-19 10:00:00	26.1	39	1.8	2	248	west wind	943	24	0	80
2021-06-19 11:00:00	26.7	47	1.4	1	212	southwest wind	943	30	0	85
2021-06-19 12:00:00	28.6	35	2.4	2	247	southwest wind	943	30	0	80
2021-06-19 13:00:00	29.5	35	1.9	2	259	west wind	943	30	0	70
2021-06-19 14:00:00	30	29	0.9	1	181	south wind	942	30	0	85
2021-06-19 15:00:00	28.4	30	4.6	3	136	southeast wind	942	30	0	85
2021-06-19 16:00:00	27.7	35	2.6	2	157	southeast wind	942	30	0	85
2021-06-19 17:00:00	27	34	5.1	3	247	southwest wind	943	30	0	85
2021-06-19 18:00:00	25.8	36	3.4	3	243	southwest wind	943	30	0	75
2021-06-19 19:00:00	26.5	33	2.8	2	247	southwest wind	943	30	0	90
2021-06-19 20:00:00	26	40	1.3	1	279	west wind	942	30	0	85
2021-06-19 21:00:00	25.4	51	0.4	1	209	southwest wind	942	25.5	0	85
2021-06-19 22:00:00	24	52	1.7	2	254	west wind	943	30	0	75
2021-06-19 23:00:00	23.7	47	2	2	252	west wind	943	30	0	85
2021-06-20 00:00:00	23.3	43	1.7	2	306	northwest wind	943	30	0	85
2021-06-20 01:00:00	21.8	44	1.3	1	327	northwest wind	943	30	0	50
2021-06-20 02:00:00	20.3	51	1.2	1	262	west wind	943	30	0	40
2021-06-20 03:00:00	19.6	53	2.2	2	262	west wind	944	5.4	0	55
2021-06-20 04:00:00	23.9	36	7.8	4	239	southwest wind	944	30	0	55
2021-06-20 05:00:00	24	34	3.3	2	199	south wind	943	30	0	65
2021-06-20 06:00:00	21.5	41	4.1	3	264	west wind	944	30	0	40
2021-06-20 07:00:00	21.4	42	3.3	2	259	west wind	944	30	0	95
2021-06-20 08:00:00	23	40	3.1	2	255	west wind	944	30	0	80
2021-06-20 09:00:00	21.8	48	1	1	141	southeast wind	945	30	0	80
2021-06-20 10:00:00	23.4	48	1.5	1	161	south wind	945	30	0	75
2021-06-20 11:00:00	25	40	1.5	1	146	southeast wind	945	30	0	65
2021-06-20 12:00:00	23.9	40	1.2	1	101	east wind	946	30	0	95
2021-06-20 13:00:00	24.1	40	1.9	2	99	east wind	945	24.4	0	80
2021-06-20 14:00:00	22.2	52	5.2	3	80	east wind	946	30	0	85
2021-06-20 15:00:00	23.5	45	4.1	3	89	east wind	946	22.5	0	85
2021-06-20 16:00:00	24	40	3.9	3	87	east wind	946	30	0	40
2021-06-20 17:00:00	24.1	35	3.7	3	81	east wind	947	30	0	35
2021-06-20 18:00:00	24	37	2.1	2	102	east wind	948	30	0	45
2021-06-20 19:00:00	23.5	38	2.5	2	112	east wind	948	30	0	30
2021-06-20 20:00:00	22.7	37	2.9	2	126	southeast wind	949	30	0	0
2021-06-20 21:00:00	20.9	41	3.8	3	124	southeast wind	951	28.6	0	0
2021-06-20 22:00:00	19.1	45	4.3	3	145	southeast wind	952	30	0	0

2021-06-20 23:00:00	17.7	42	3.2	2	147	southeast wind	953	30	0	45
2021-06-21 00:00:00	16.9	37	2.7	2	135	southeast wind	954	30	0	0
2021-06-21 01:00:00	16.6	37	2.1	2	115	southeast wind	954	30	0	0
2021-06-21 02:00:00	15.6	41	1.3	1	275	west wind	954	30	0	0
2021-06-21 03:00:00	13.6	53	0.9	1	256	west wind	954	30	0	15
2021-06-21 04:00:00	11.8	63	0.8	1	275	west wind	954	30	0	45
2021-06-21 05:00:00	10.8	67	1.8	2	253	west wind	953	30	0	45
2021-06-21 06:00:00	10.7	66	1.8	2	284	west wind	953	30	0	45
2021-06-21 07:00:00	10.6	69	1.7	2	249	west wind	953	26.3	0	0
2021-06-21 08:00:00	13.2	61	1.4	1	247	southwest wind	953	24	0	0
2021-06-21 09:00:00	16.2	53	1.3	1	244	southwest wind	953	29	0	0
2021-06-21 10:00:00	19.5	37	0.8	1	133	southeast wind	953	30	0	0
2021-06-21 11:00:00	20.1	35	2.8	2	84	east wind	952	30	0	0
2021-06-21 12:00:00	20.8	35	2.5	2	58	northeast wind	952	24.9	0	0
2021-06-21 13:00:00	21.5	33	2.3	2	41	northeast wind	951	28.8	0	0
2021-06-21 14:00:00	22.1	31	2.6	2	81	east wind	950	30	0	0
2021-06-21 15:00:00	23.1	30	2.9	2	55	northeast wind	949	25.7	0	0
2021-06-21 16:00:00	23.7	31	3.4	3	70	east wind	948	23.6	0	0
2021-06-21 17:00:00	24.5	30	2.6	2	113	southeast wind	947	27.2	0	0
2021-06-21 18:00:00	24.4	32	2.5	2	62	northeast wind	947	26.5	0	0
2021-06-21 19:00:00	24.6	32	1.8	2	69	east wind	946	27.7	0	0
2021-06-21 20:00:00	24	30	1.7	2	62	northeast wind	946	25.4	0	55
2021-06-21 21:00:00	23.1	35	1	1	322	northwest wind	946	26.1	0	0
2021-06-21 22:00:00	20.7	47	1	1	332	northwest wind	946	7	0	0
2021-06-21 23:00:00	18.8	53	1.7	2	262	west wind	947	22.1	0	45
2021-06-22 00:00:00	17.4	59	1.5	1	246	southwest wind	947	24.5	0	50
2021-06-22 01:00:00	17	60	1.7	2	252	west wind	946	22.9	0	55
2021-06-22 02:00:00	16.7	63	1.6	2	266	west wind	946	27	0	90
2021-06-22 03:00:00	17.1	57	1.8	2	275	west wind	946	27.8	0	80
2021-06-22 04:00:00	16.5	63	1.5	1	252	west wind	946	29.4	0	90
2021-06-22 05:00:00	16.4	62	1.8	2	256	west wind	947	29.3	0	90
2021-06-22 06:00:00	15.6	65	1.5	1	257	west wind	946	30	0	90
2021-06-22 07:00:00	15.9	65	2.1	2	251	west wind	947	27.5	0	85
2021-06-22 08:00:00	17	62	2.1	2	236	southwest wind	947	28.1	0	90
2021-06-22 09:00:00	17.9	61	1.7	2	218	southwest wind	948	30	0	80
2021-06-22 10:00:00	20.6	54	1	1	250	west wind	948	30	0	80
2021-06-22 11:00:00	22	53	1.5	1	15	north wind	948	22.3	0	85
2021-06-22 12:00:00	21	49	2.3	2	118	southeast wind	948	30	0	90

2021-06-22 13:00:00	21.7	48	2.3	2	54	northeast wind	948	30	0	85
2021-06-22 14:00:00	22.5	45	2.7	2	107	east wind	948	23.3	0	80
2021-06-22 15:00:00	24	39	4.5	3	79	east wind	947	30	0	80
2021-06-22 16:00:00	24.1	38	4.9	3	79	east wind	948	11.8	0	0
2021-06-22 17:00:00	24.9	36	4.8	3	66	northeast wind	948	23.2	0	70
2021-06-22 18:00:00	24.5	35	3.6	3	62	northeast wind	948	30	0	65
2021-06-22 19:00:00	23.9	35	3.7	3	80	east wind	949	18.4	0	65
2021-06-22 20:00:00	23.2	35	3	2	84	east wind	950	30	0	75
2021-06-22 21:00:00	21.6	40	1.5	1	103	east wind	951	30	0	65
2021-06-22 22:00:00	20.5	43	1.7	2	97	east wind	951	30	0	45
2021-06-22 23:00:00	19.4	47	2.8	2	119	southeast wind	952	27.9	0	40
2021-06-23 00:00:00	18.2	51	3.4	3	127	southeast wind	952	26.8	0	45
2021-06-23 01:00:00	17.8	50	2.6	2	127	southeast wind	952	30	0	45
2021-06-23 02:00:00	17.4	47	3.3	2	123	southeast wind	952	14.4	0	40
2021-06-23 03:00:00	17	47	2.6	2	131	southeast wind	953	30	0	75
2021-06-23 04:00:00	16.3	50	2.3	2	126	southeast wind	953	30	0	100
2021-06-23 05:00:00	15.2	55	1.4	1	139	southeast wind	953	30	0	55
2021-06-23 06:00:00	13.5	63	1.8	2	321	northwest wind	953	30	0	75
2021-06-23 07:00:00	12.3	70	1.1	1	313	northwest wind	954	30	0	65
2021-06-23 08:00:00	14.7	65	0.9	1	301	northwest wind	954	30	0	70
2021-06-23 09:00:00	17.6	51	1	1	47	northeast wind	954	30	0	60
2021-06-23 10:00:00	19.1	48	1.3	1	270	west wind	954	30	0	0
2021-06-23 11:00:00	20.2	43	2.1	2	354	north wind	954	30	0	0
2021-06-23 12:00:00	20.5	44	3.4	3	66	northeast wind	953	30	0	0
2021-06-23 13:00:00	21.3	45	2.3	2	83	east wind	953	30	0	0
2021-06-23 14:00:00	21.4	42	3.2	2	126	southeast wind	953	30	0	85
2021-06-23 15:00:00	22.9	40	3.1	2	63	northeast wind	952	30	0	0
2021-06-23 16:00:00	23.1	39	3.2	2	84	east wind	951	30	0	0
2021-06-23 17:00:00	23.9	35	3.2	2	71	east wind	951	30	0	75
2021-06-23 18:00:00	23.9	33	2.7	2	79	east wind	951	30	0	85
2021-06-23 19:00:00	24.2	34	2.2	2	103	east wind	951	30	0	65
2021-06-23 20:00:00	24	34	2.4	2	104	east wind	951	29.7	0	55
2021-06-23 21:00:00	23.1	35	2.5	2	124	southeast wind	951	28.1	0	0
2021-06-23 22:00:00	22	38	1.4	1	118	southeast wind	951	29.8	0	0
2021-06-23 23:00:00	19.2	52	0.8	1	283	west wind	952	18.9	0	0
2021-06-24 00:00:00	17.7	55	1.1	1	301	northwest wind	952	30	0	60
2021-06-24 01:00:00	17.1	58	1.3	1	280	west wind	951	30	0	80
2021-06-24 02:00:00	15.5	66	0.7	1	253	west wind	951	30	0	50

2021-06-24 03:00:00	14.9	66	1.9	2	263	west wind	951	30	0	75
2021-06-24 04:00:00	14	69	1.1	1	299	northwest wind	950	30	0	75
2021-06-24 05:00:00	13.5	71	1.6	2	241	southwest wind	950	30	0	45
2021-06-24 06:00:00	12.1	80	1.9	2	254	west wind	950	30	0	75
2021-06-24 07:00:00	12.1	79	1.8	2	254	west wind	949	30	0	85
2021-06-24 08:00:00	12.5	76	2.1	2	269	west wind	949	28	0	95
2021-06-24 09:00:00	16.1	65	1.8	2	252	west wind	949	24.7	0	45
2021-06-24 10:00:00	20.8	48	0.9	1	280	west wind	948	30	0	0
2021-06-24 11:00:00	22.6	37	1.7	2	38	northeast wind	947	30	0	0
2021-06-24 12:00:00	23.4	40	2.5	2	80	east wind	947	30	0	60
2021-06-24 13:00:00	23.6	41	3.2	2	77	east wind	946	30	0	50
2021-06-24 14:00:00	23.9	40	3.2	2	75	east wind	945	30	0	85
2021-06-24 15:00:00	25.6	35	2.9	2	90	east wind	944	30	0	85
2021-06-24 16:00:00	26.3	32	3.1	2	102	east wind	944	30	0	82
2021-06-24 17:00:00	27.2	31	3	2	103	east wind	943	27.5	0	82
2021-06-24 18:00:00	27.7	29	3.2	2	109	east wind	942	23.3	0	23
2021-06-24 19:00:00	28	26	2.9	2	86	east wind	942	30	0	23
2021-06-24 20:00:00	27.8	27	2.3	2	102	east wind	942	26.9	0	23
2021-06-24 21:00:00	25.6	33	1.6	2	150	southeast wind	943	12.9	0	1
2021-06-24 22:00:00	24.6	34	1.7	2	106	east wind	943	12.8	0	1
2021-06-24 23:00:00	21.3	50	0.4	1	266	west wind	944	21.1	0	1
2021-06-25 00:00:00	22	44	3.8	3	120	southeast wind	944	28.6	0	1
2021-06-25 01:00:00	20.6	50	3.7	3	107	east wind	944	30	0	1
2021-06-25 02:00:00	19.6	56	2.1	2	58	northeast wind	944	30	0	1
2021-06-25 03:00:00	18.7	61	1.5	1	279	west wind	944	30	0	1
2021-06-25 04:00:00	18.4	64	0.4	1	190	south wind	945	30	0	1
2021-06-25 05:00:00	18.6	66	1.2	1	129	southeast wind	945	30	0	1
2021-06-25 06:00:00	16.6	69	2.2	2	106	east wind	946	30	0	1
2021-06-25 07:00:00	15.4	73	1.5	1	227	southwest wind	947	30	0.1	1
2021-06-25 08:00:00	14.7	79	1.3	1	177	south wind	947	30	0	1
2021-06-25 09:00:00	15.1	75	2.8	2	152	southeast wind	948	30	0.1	93
2021-06-25 10:00:00	16.2	70	4.6	3	139	southeast wind	949	30	0	93
2021-06-25 11:00:00	16.4	67	4.1	3	132	southeast wind	949	29.2	0	93
2021-06-25 12:00:00	18.6	59	3.3	2	120	southeast wind	950	30	0	82
2021-06-25 13:00:00	18.1	57	2.9	2	106	east wind	950	30	0	82
2021-06-25 14:00:00	20.2	54	3.1	2	120	southeast wind	949	30	0	82
2021-06-25 15:00:00	20.4	50	3.1	2	83	east wind	949	30	0	93
2021-06-25 16:00:00	21	52	2.3	2	53	northeast wind	949	30	0	93

2021-06-25 17:00:00	21.3	53	2	2	76	east wind	949	30	0	93
2021-06-25 18:00:00	21.5	49	2.5	2	93	east wind	949	30	0	82
2021-06-25 19:00:00	22.2	44	1.6	2	88	east wind	949	30	0	82
2021-06-25 20:00:00	21	40	3.3	2	113	southeast wind	949	30	0	82
2021-06-25 21:00:00	20.4	42	2.7	2	122	southeast wind	950	30	0	23
2021-06-25 22:00:00	19.8	43	2.2	2	113	southeast wind	950	30	0	23
2021-06-25 23:00:00	19.3	42	1.9	2	116	southeast wind	950	30	0	23
2021-06-26 00:00:00	18	50	1.4	1	244	southwest wind	950	30	0	23
2021-06-26 01:00:00	16.3	60	1.1	1	331	northwest wind	949	30	0	23
2021-06-26 02:00:00	15.3	66	0.7	1	266	west wind	949	30	0	23
2021-06-26 03:00:00	14.2	75	0.8	1	255	west wind	949	30	0	23
2021-06-26 04:00:00	13.8	77	0.9	1	278	west wind	949	30	0	23
2021-06-26 05:00:00	14.7	69	1.3	1	275	west wind	949	30	0	23
2021-06-26 06:00:00	13.4	80	0.7	1	236	southwest wind	949	30	0	23
2021-06-26 07:00:00	13.5	81	1.4	1	267	west wind	949	24	0	23
2021-06-26 08:00:00	13.9	80	1	1	247	southwest wind	949	22.7	0	23
2021-06-26 09:00:00	18.3	64	0.8	1	291	west wind	949	30	0	93
2021-06-26 10:00:00	20.3	59	2	2	73	east wind	949	30	0	93
2021-06-26 11:00:00	20.5	53	2.7	2	59	northeast wind	949	30	0	93
2021-06-26 12:00:00	20.6	52	2.9	2	87	east wind	949	28.1	0	1
2021-06-26 13:00:00	21.7	52	3.5	3	72	east wind	948	30	0	1
2021-06-26 14:00:00	21.7	49	2.2	2	76	east wind	948	30	0	1
2021-06-26 15:00:00	22.2	49	2.7	2	76	east wind	947	30	0	23
2021-06-26 16:00:00	22.3	49	2.6	2	102	east wind	947	30	0	23
2021-06-26 17:00:00	23.4	42	2.5	2	99	east wind	947	30	0	23
2021-06-26 18:00:00	23.3	41	2.4	2	93	east wind	947	30	0	82
2021-06-26 19:00:00	23	40	1.1	1	143	southeast wind	948	30	0	82
2021-06-26 20:00:00	21.1	51	1.7	2	311	northwest wind	948	30	0	82
2021-06-26 21:00:00	19.9	56	1.2	1	329	northwest wind	949	30	0	82
2021-06-26 22:00:00	18.9	64	0.9	1	169	south wind	949	30	0	82
2021-06-26 23:00:00	18.7	64	1	1	69	east wind	949	30	0	82
2021-06-27 00:00:00	17.5	70	1	1	251	west wind	949	27.7	0	82
2021-06-27 01:00:00	16.5	77	1.1	1	322	northwest wind	949	29.2	0	82
2021-06-27 02:00:00	15.1	82	1.1	1	242	southwest wind	949	30	0	82
2021-06-27 03:00:00	14.9	84	2	2	259	west wind	949	30	0	82
2021-06-27 04:00:00	15.4	80	1.2	1	265	west wind	949	30	0	82
2021-06-27 05:00:00	15.5	87	3.2	2	271	west wind	951	16	0	82
2021-06-27 06:00:00	14.8	91	1.1	1	24	northeast wind	950	30	3.1	82

2021-06-27 07:00:00	13.6	93	1	1	92	east wind	951	30	0.2	82
2021-06-27 08:00:00	15.5	76	2.6	2	82	east wind	952	30	0.1	82
2021-06-27 09:00:00	16.1	71	1.9	2	151	southeast wind	953	30	0	100
2021-06-27 10:00:00	17.3	66	2.4	2	181	south wind	953	30	0	100
2021-06-27 11:00:00	19.2	61	2.6	2	130	southeast wind	954	30	0	100
2021-06-27 12:00:00	19.7	58	3.1	2	122	southeast wind	954	30	0	1
2021-06-27 13:00:00	21	54	3.7	3	140	southeast wind	954	30	0	1
2021-06-27 14:00:00	22.1	49	2.7	2	136	southeast wind	954	30	0	1
2021-06-27 15:00:00	22.5	41	2.7	2	95	east wind	953	30	0	1
2021-06-27 16:00:00	23.5	43	2.7	2	80	east wind	953	30	0	1
2021-06-27 17:00:00	24	42	1.6	2	83	east wind	953	30	0	1
2021-06-27 18:00:00	24	41	2	2	113	southeast wind	953	30	0	1
2021-06-27 19:00:00	24.5	36	1.9	2	101	east wind	953	30	0	1
2021-06-27 20:00:00	24.3	37	1.8	2	92	east wind	953	30	0	1
2021-06-27 21:00:00	23.3	43	2.4	2	142	southeast wind	954	30	0	1
2021-06-27 22:00:00	21.5	48	1.4	1	184	south wind	954	30	0	1
2021-06-27 23:00:00	19.3	61	0.8	1	272	west wind	955	18.7	0	1
2021-06-28 00:00:00	17.6	65	1.2	1	319	northwest wind	954	30	0	1
2021-06-28 01:00:00	16.3	72	0.8	1	124	southeast wind	954	30	0	1
2021-06-28 02:00:00	15.4	78	1.1	1	270	west wind	954	30	0	1
2021-06-28 03:00:00	15	82	1.6	2	271	west wind	954	30	0	1
2021-06-28 04:00:00	14.1	85	1	1	288	west wind	953	30	0	1
2021-06-28 05:00:00	13.3	89	1.2	1	257	west wind	953	29.4	0	1
2021-06-28 06:00:00	12.6	90	1.3	1	253	west wind	953	28.8	0	1
2021-06-28 07:00:00	12.6	89	1	1	262	west wind	954	27.2	0	1
2021-06-28 08:00:00	15.1	79	1.7	2	249	west wind	954	30	0	1
2021-06-28 09:00:00	18.3	66	0.8	1	265	west wind	953	30	0	1
2021-06-28 10:00:00	21.7	45	1.3	1	34	northeast wind	953	30	0	1
2021-06-28 11:00:00	22.8	41	2.2	2	63	northeast wind	953	30	0	1
2021-06-28 12:00:00	23.1	44	2.6	2	79	east wind	952	30	0	1
2021-06-28 13:00:00	24.1	42	2.4	2	97	east wind	952	30	0	1
2021-06-28 14:00:00	25.1	42	1.9	2	75	east wind	951	30	0	1
2021-06-28 15:00:00	25.5	39	2	2	61	northeast wind	951	30	0	1
2021-06-28 16:00:00	26.5	38	1.9	2	118	southeast wind	950	30	0	1
2021-06-28 17:00:00	26.5	34	2.3	2	77	east wind	950	30	0	1
2021-06-28 18:00:00	27.1	32	1.5	1	28	northeast wind	950	30	0	1
2021-06-28 19:00:00	27.3	31	1.4	1	96	east wind	949	30	0	1
2021-06-28 20:00:00	27.2	28	1.7	2	104	east wind	950	30	0	1

2021-06-28 21:00:00	26.5	35	1.1	1	333	northwest wind	950	24.6	0	1
2021-06-28 22:00:00	23.6	46	1.2	1	4	north wind	950	26.6	0	1
2021-06-28 23:00:00	20.8	58	1.3	1	314	northwest wind	950	24.1	0	1
2021-06-29 00:00:00	19.8	60	0.9	1	284	west wind	950	30	0	1
2021-06-29 01:00:00	18.6	65	1.6	2	254	west wind	950	30	0	1
2021-06-29 02:00:00	17.5	74	2	2	258	west wind	950	30	0	1
2021-06-29 03:00:00	17.5	73	1.5	1	274	west wind	950	30	0	1
2021-06-29 04:00:00	16.7	76	1.6	2	245	southwest wind	950	30	0	1
2021-06-29 05:00:00	15.7	81	1.7	2	258	west wind	950	30	0	1
2021-06-29 06:00:00	15.6	82	2.4	2	261	west wind	950	30	0	1
2021-06-29 07:00:00	15.4	82	2.3	2	257	west wind	950	30	0	1
2021-06-29 08:00:00	17.6	75	1.5	1	250	west wind	950	30	0	1
2021-06-29 09:00:00	20.7	67	1.4	1	243	southwest wind	950	27.2	0	1
2021-06-29 10:00:00	25.2	47	0.9	1	1	north wind	950	30	0	1
2021-06-29 11:00:00	26.9	37	1.7	2	97	east wind	950	30	0	1
2021-06-29 12:00:00	27.7	32	2.1	2	88	east wind	950	30	0	1
2021-06-29 13:00:00	28.9	32	2.2	2	113	southeast wind	949	30	0	1
2021-06-29 14:00:00	29.7	31	2.1	2	58	northeast wind	949	30	0	1
2021-06-29 15:00:00	29.9	31	2.6	2	114	southeast wind	949	30	0	1
2021-06-29 16:00:00	30.2	25	2.2	2	97	east wind	948	30	0	1
2021-06-29 17:00:00	31.5	25	2.2	2	103	east wind	947	30	0	1
2021-06-29 18:00:00	31.4	23	2.3	2	80	east wind	947	30	0	1
2021-06-29 19:00:00	31.5	23	2.6	2	88	east wind	947	30	0	1
2021-06-29 20:00:00	31.3	21	1.7	2	120	southeast wind	947	11.8	0	1
2021-06-29 21:00:00	30	27	0.9	1	139	southeast wind	947	25.1	0	1
2021-06-29 22:00:00	26.2	37	1.4	1	307	northwest wind	947	30	0	1
2021-06-29 23:00:00	26.5	26	1.8	2	292	west wind	948	30	0	1
2021-06-30 00:00:00	23.9	34	1.2	1	242	southwest wind	948	30	0	1
2021-06-30 01:00:00	21.7	47	2	2	236	southwest wind	948	30	0	1
2021-06-30 02:00:00	21	50	1.6	2	274	west wind	948	30	0	1
2021-06-30 03:00:00	19.9	56	2.1	2	262	west wind	947	30	0	1
2021-06-30 04:00:00	18.5	63	1.8	2	249	west wind	947	30	0	1
2021-06-30 05:00:00	18.2	64	2	2	256	west wind	947	30	0	1
2021-06-30 06:00:00	17.6	64	2.4	2	251	west wind	947	30	0	1
2021-06-30 07:00:00	17.1	66	2.2	2	260	west wind	947	30	0	1
2021-06-30 08:00:00	18.6	63	3.1	2	254	west wind	948	30	0	1
2021-06-30 09:00:00	23.7	48	2.2	2	247	southwest wind	947	30	0	1
2021-06-30 10:00:00	27.8	37	2.2	2	263	west wind	947	30	0	1

2021-06-30 11:00:00	30.2	27	1.1	1	313	northwest wind	947	30	0	1
2021-06-30 12:00:00	31.9	24	1.4	1	30	northeast wind	947	29.4	0	1
2021-06-30 13:00:00	32	25	3.4	3	64	northeast wind	946	30	0	1
2021-06-30 14:00:00	32.7	27	2.9	2	74	east wind	946	28.3	0	1
2021-06-30 15:00:00	33.1	23	3.1	2	108	east wind	945	30	0	1
2021-06-30 16:00:00	33.3	21	3.7	3	77	east wind	945	30	0	1
2021-06-30 17:00:00	33.5	22	3.8	3	90	east wind	945	29	0	1
2021-06-30 18:00:00	33.8	23	3.3	2	104	east wind	945	30	0	1
2021-06-30 19:00:00	33.4	25	2.9	2	114	southeast wind	945	30	0	1
2021-06-30 20:00:00	32.6	24	3.2	2	124	southeast wind	945	17.6	0	1
2021-06-30 21:00:00	31.5	24	2.4	2	117	southeast wind	945	29.5	0	1
2021-06-30 22:00:00	29.8	33	1.6	2	105	east wind	946	30	0	1
2021-06-30 23:00:00	28.2	30	1.2	1	110	east wind	947	30	0	1
2021-07-01 00:00:00	27.3	28	1.5	1	37	northeast wind	947	30	0	1
2021-07-01 01:00:00	24.2	36	1.7	2	338	north wind	947	30	0	1
2021-07-01 02:00:00	23.2	39	1.1	1	312	northwest wind	947	30	0	1
2021-07-01 03:00:00	21.2	46	1.7	2	260	west wind	947	30	0	1
2021-07-01 04:00:00	18.6	63	2.1	2	251	west wind	947	30	0	1
2021-07-01 05:00:00	17.6	68	1.6	2	257	west wind	947	30	0	1
2021-07-01 06:00:00	16.9	71	1.9	2	261	west wind	947	30	0	1
2021-07-01 07:00:00	16.3	73	1.5	1	243	southwest wind	947	30	0	1
2021-07-01 08:00:00	17.8	67	2.1	2	251	west wind	947	30	0	1
2021-07-01 09:00:00	21.8	56	1.5	1	236	southwest wind	947	30	0	1
2021-07-01 10:00:00	26	39	1.4	1	276	west wind	947	30	0	1
2021-07-01 11:00:00	28	30	1.4	1	282	west wind	947	30	0	1
2021-07-01 12:00:00	28	30	1.4	1	282	west wind	947	29.6	0	1
2021-07-01 13:00:00	28	30	1.4	1	282	west wind	947	30	0	1
2021-07-01 14:00:00	28	30	1.4	1	282	west wind	947	28.2	0	
2021-07-01 15:00:00	32.4	24	0.6	1	117	southeast wind	945	30	0	
2021-07-01 16:00:00	32.4	24	0.6	1	117	southeast wind	945	30	0	
2021-07-01 17:00:00	33.6	19	2	2	136	southeast wind	944	30	0	
2021-07-01 18:00:00	33.8	22	1.4	1	37	northeast wind	944	30	0	
2021-07-01 19:00:00	34	18	1.6	2	154	southeast wind	944	30	0	
2021-07-01 20:00:00	33.7	20	1	1	115	southeast wind	943	30	0	
2021-07-01 21:00:00	33.1	19	0.4	1	152	southeast wind	944	29.3	0	
2021-07-01 22:00:00	29.2	33	0.6	1	314	northwest wind	944	16.5	0	
2021-07-01 23:00:00	26.3	46	1.5	1	316	northwest wind	944	16.5	0	
2021-07-02 00:00:00	23.5	57	1.9	2	318	northwest wind	945	16.5	0	

2021-07-02 01:00:00	22.1	55	1.9	2	304	northwest wind	945	16.5	0	
2021-07-02 02:00:00	21.9	54	3	2	224	southwest wind	945	16.5	0	
2021-07-02 03:00:00	20.3	62	2.1	2	213	southwest wind	945	16.5	0	
2021-07-02 04:00:00	19.3	64	2.9	2	215	southwest wind	944	16.5	0	
2021-07-02 05:00:00	18.6	64	0.6	1	225	southwest wind	944	16.5	0	
2021-07-02 06:00:00	18.1	65	2.6	2	213	southwest wind	944	16.5	0	
2021-07-02 07:00:00	17.4	68	2.4	2	237	southwest wind	944	16.5	0	
2021-07-02 08:00:00	18.9	63	0.2	0	221	southwest wind	944	30	0	
2021-07-02 09:00:00	21.8	57	2.7	2	228	southwest wind	944	26.3	0	
2021-07-02 10:00:00	25.5	48	0.7	1	237	southwest wind	944	26.3	0	
2021-07-02 11:00:00	29	36	2.8	2	313	northwest wind	943	26.3	0	0
2021-07-02 12:00:00	25	29	0.7	1	0	north wind	943	26.3	0	0
2021-07-02 13:00:00	25	26	0.5	1	34	northeast wind	942	30	0	0
2021-07-02 14:00:00	33.1	27	1.4	1	40	northeast wind	942	30	0	0
2021-07-02 15:00:00	33.5	25	2.3	2	133	southeast wind	941	30	0	0
2021-07-02 16:00:00	34.3	25	2.6	2	144	southeast wind	941	30	0	0
2021-07-02 17:00:00	34.4	22	2.9	2	39	northeast wind	940	30	0	0
2021-07-02 18:00:00	34.9	19	1.2	1	127	southeast wind	940	30	0	0
2021-07-02 19:00:00	35.1	23	1	1	128	southeast wind	940	30	0	0
2021-07-02 20:00:00	34.9	25	2.4	2	154	southeast wind	940	30	0	0
2021-07-02 21:00:00	33.8	26	1.9	2	139	southeast wind	940	30	0	0
2021-07-02 22:00:00	31.3	33	1.5	1	118	southeast wind	940	17.5	0	0
2021-07-02 23:00:00	27.5	50	0.6	1	314	northwest wind	941	17.5	0	0
2021-07-03 00:00:00	26.7	50	2.8	2	303	northwest wind	941	17.5	0	0
2021-07-03 01:00:00	25.7	50	3	2	337	northwest wind	942	17.5	0	0
2021-07-03 02:00:00	24.2	54	2.5	2	298	northwest wind	942	17.5	0	0
2021-07-03 03:00:00	22.7	61	3.1	2	240	southwest wind	942	17.5	0	0
2021-07-03 04:00:00	21	68	1.4	1	229	southwest wind	941	17.5	0	0
2021-07-03 05:00:00	20.2	69	0.6	1	217	southwest wind	941	17.5	0	0
2021-07-03 06:00:00	19.7	70	2.2	2	209	southwest wind	941	17.5	0	0
2021-07-03 07:00:00	19.4	70	1.7	2	204	southwest wind	941	17.5	0	0
2021-07-03 08:00:00	20.7	65	2	2	0	north wind	942	17.5	0	0
2021-07-03 09:00:00	23.1	61	2.8	2	240	southwest wind	942	17.5	0	0
2021-07-03 10:00:00	26.9	53	1.1	1	246	southwest wind	942	17.2	0	0
2021-07-03 11:00:00	30.8	33	0.2	0	115	southeast wind	942	30	0	0
2021-07-03 12:00:00	32.1	26	2.5	2	25	northeast wind	942	30	0	0
2021-07-03 13:00:00	32.7	28	1.1	1	132	southeast wind	942	30	0	0
2021-07-03 14:00:00	33.8	27	2.8	2	118	southeast wind	941	30	0	0

2021-07-03 15:00:00	34.3	25	1.8	2	40	northeast wind	941	30	0	0
2021-07-03 16:00:00	35.1	25	1	1	147	southeast wind	940	30	0	0
2021-07-03 17:00:00	35.7	21	3.2	2	38	northeast wind	940	30	0	0
2021-07-03 18:00:00	36.2	20	2	2	116	southeast wind	940	27.1	0	0
2021-07-03 19:00:00	36	21	2.9	2	126	southeast wind	940	27.1	0	0
2021-07-03 20:00:00	35.4	19	1.1	1	129	southeast wind	940	21	0	0
2021-07-03 21:00:00	33.6	24	0.2	0	144	southeast wind	940	21	0	0
2021-07-03 22:00:00	30.2	35	2.1	2	332	northwest wind	941	21	0	0
2021-07-03 23:00:00	27.5	44	1.1	1	332	northwest wind	941	21	0	0
2021-07-04 00:00:00	25.2	51	1.8	2	305	northwest wind	942	21	0	0
2021-07-04 01:00:00	25.3	43	0.5	1	66	northeast wind	942	3	0	0
2021-07-04 02:00:00	23.9	45	0.6	1	293	northwest wind	942	30	0	0
2021-07-04 03:00:00	21.8	58	2.2	2	208	southwest wind	942	30	0	0
2021-07-04 04:00:00	20.6	63	0.2	0	242	southwest wind	942	30	0	0
2021-07-04 05:00:00	19.9	65	2.4	2	204	southwest wind	942	30	0	0
2021-07-04 06:00:00	19.6	65	1	1	245	southwest wind	942	30	0	0
2021-07-04 07:00:00	20.4	60	0.9	1	216	southwest wind	943	30	0	0
2021-07-04 08:00:00	20.9	60	3.1	2	210	southwest wind	943	30	0	0
2021-07-04 09:00:00	24	52	3.1	2	212	southwest wind	943	30	0	0
2021-07-04 10:00:00	27.3	46	2	2	214	southwest wind	943	30	0	0
2021-07-04 11:00:00	31.3	38	1.6	2	244	southwest wind	943	30	0	0
2021-07-04 12:00:00	32.7	27	2	2	125	southeast wind	943	29.4	0	0
2021-07-04 13:00:00	33.4	25	0.8	1	57	northeast wind	943	30	0	0
2021-07-04 14:00:00	33.9	23	2.4	2	116	southeast wind	942	30	0	0
2021-07-04 15:00:00	35.1	19	1.5	1	127	southeast wind	942	30	0	0
2021-07-04 16:00:00	35.4	21	2.7	2	154	southeast wind	941	28.2	0	0
2021-07-04 17:00:00	36.1	21	2.2	2	139	southeast wind	941	30	0	40
2021-07-04 18:00:00	36.6	18	2.3	2	119	southeast wind	941	24.8	0	80
2021-07-04 19:00:00	35.7	19	0.8	1	132	southeast wind	941	30	0	0
2021-07-04 20:00:00	35.3	24	2.8	2	133	southeast wind	941	30	0	85
2021-07-04 21:00:00	31.9	38	0.8	1	318	northwest wind	942	30	0	90
2021-07-04 22:00:00	30.3	32	4.3	3	206	southwest wind	942	30	0	85
2021-07-04 23:00:00	29.5	34	4.5	3	234	southwest wind	943	30	0	70
2021-07-05 00:00:00	28.9	33	4.2	3	206	southwest wind	943	30	0	70
2021-07-05 01:00:00	27.6	36	2.1	2	220	southwest wind	943	30	0	65
2021-07-05 02:00:00	27.3	36	4.2	3	293	northwest wind	942	30	0	45
2021-07-05 03:00:00	25.7	44	2.5	2	207	southwest wind	942	30	0	45
2021-07-05 04:00:00	25	44	0.4	1	237	southwest wind	942	30	0	85

2021-07-05 05:00:00	25.3	43	0.8	1	243	southwest wind	942	30	0	90
2021-07-05 06:00:00	24.8	46	1.8	2	206	southwest wind	942	30	0	55
2021-07-05 07:00:00	25.1	44	3	2	0	north wind	942	30	0	90
2021-07-05 08:00:00	24.5	48	1.7	2	233	southwest wind	942	30	0	90
2021-07-05 09:00:00	25.3	49	2.6	2	239	southwest wind	942	30	0	65
2021-07-05 10:00:00	26.2	51	0.4	1	215	southwest wind	942	30	0	75
2021-07-05 11:00:00	28.1	50	1.2	1	215	southwest wind	942	22	0	0
2021-07-05 12:00:00	33.5	36	1.8	2	323	northwest wind	942	26.2	0	0
2021-07-05 13:00:00	35.3	28	2	2	142	southeast wind	942	26.2	0	30
2021-07-05 14:00:00	35.8	24	2	2	117	southeast wind	942	30	0	65
2021-07-05 15:00:00	36	22	2.3	2	152	southeast wind	941	30	0	80
2021-07-05 16:00:00	36.1	25	1.8	2	125	southeast wind	941	22.8	0	80
2021-07-05 17:00:00	35.6	24	2.8	2	135	southeast wind	940	30	0	85
2021-07-05 18:00:00	36.7	21	0.9	1	135	southeast wind	940	30	0	55
2021-07-05 19:00:00	36.1	22	1	1	128	southeast wind	940	30	0	65
2021-07-05 20:00:00	35.5	22	0.3	1	157	southeast wind	940	15.4	0	85
2021-07-05 21:00:00	26.3	57	2.8	2	56	northeast wind	942	30	1.6	75
2021-07-05 22:00:00	25.7	61	3	2	211	southwest wind	941	30	0.2	45
2021-07-05 23:00:00	25.2	59	2.5	2	232	southwest wind	942	30	0	0
2021-07-06 00:00:00	24.6	61	2.3	2	218	southwest wind	942	30	0	0
2021-07-06 01:00:00	23.7	63	3	2	220	southwest wind	942	30	0	0
2021-07-06 02:00:00	24.1	59	2.5	2	207	southwest wind	942	30	0	35
2021-07-06 03:00:00	23.5	60	2.1	2	226	southwest wind	941	30	0	35
2021-07-06 04:00:00	21.9	68	0	0	247	southwest wind	941	30	0	0
2021-07-06 05:00:00	22.2	64	1.8	2	226	southwest wind	941	30	0	55
2021-07-06 06:00:00	22.3	61	2.4	2	227	southwest wind	941	30	0	50
2021-07-06 07:00:00	21.1	66	0.6	1	243	southwest wind	941	30	0	75
2021-07-06 08:00:00	22.4	61	1.4	1	213	southwest wind	941	30	0	45
2021-07-06 09:00:00	25.2	53	0.8	1	239	southwest wind	942	30	0	30
2021-07-06 10:00:00	27.8	48	1.3	1	229	southwest wind	941	29.6	0	0
2021-07-06 11:00:00	30.4	46	2.3	2	228	southwest wind	941	30	0	0
2021-07-06 12:00:00	33.2	42	2.3	2	120	southeast wind	941	22.8	0	0
2021-07-06 13:00:00	36.1	26	3.2	2	324	northwest wind	941	30	0	0
2021-07-06 14:00:00	37.8	17	1.3	1	41	northeast wind	940	30	0	0
2021-07-06 15:00:00	38.3	18	0.3	1	52	northeast wind	940	30	0	0
2021-07-06 16:00:00	38.4	18	4.9	3	28	northeast wind	939	30	0	55
2021-07-06 17:00:00	38.7	19	1.5	1	133	southeast wind	939	30	0	50
2021-07-06 18:00:00	38.3	19	4	3	117	southeast wind	939	27.8	0	60

2021-07-06 19:00:00	38.8	18	1.7	2	35	northeast wind	939	27.8	0	80
2021-07-06 20:00:00	37	22	0.2	0	141	southeast wind	940	27.6	0	75
2021-07-06 21:00:00	35.3	23	2.5	2	241	southwest wind	941	26.9	0	85
2021-07-06 22:00:00	33.7	23	0.6	1	208	southwest wind	942	29.2	0	90
2021-07-06 23:00:00	29.3	37	4	3	217	southwest wind	944	30	0	80
2021-07-07 00:00:00	29.8	31	5.1	3	235	southwest wind	944	30	0	85
2021-07-07 01:00:00	29.3	32	4.1	3	335	northwest wind	945	30	0	85
2021-07-07 02:00:00	28.6	33	0.7	1	245	southwest wind	945	30	0	75
2021-07-07 03:00:00	26.5	43	1.4	1	333	northwest wind	945	30	0	90
2021-07-07 04:00:00	25.8	47	2.9	2	204	southwest wind	944	30	0	45
2021-07-07 05:00:00	25.4	48	0.7	1	210	southwest wind	944	30	0	90
2021-07-07 06:00:00	23.8	57	2.7	2	204	southwest wind	944	30	0	85
2021-07-07 07:00:00	23.6	58	1.4	1	232	southwest wind	945	30	0	85
2021-07-07 08:00:00	24.8	53	2.7	2	216	southwest wind	945	30	0	85
2021-07-07 09:00:00	26.2	52	0.3	1	210	southwest wind	945	30	0	80
2021-07-07 10:00:00	27	53	3.1	2	239	southwest wind	946	30	0	85
2021-07-07 11:00:00	27.4	59	2.6	2	138	southeast wind	946	30	0	85
2021-07-07 12:00:00	27.9	59	3.1	2	308	northwest wind	947	30	0	85
2021-07-07 13:00:00	30.1	43	2.1	2	25	northeast wind	947	27.6	0	85
2021-07-07 14:00:00	31.5	33	1.5	1	148	southeast wind	947	27.6	0	
2021-07-07 15:00:00	31.2	35	2.3	2	37	northeast wind	947	30	0	90
2021-07-07 16:00:00	32.7	33	1.9	2	145	southeast wind	947	30	0	85
2021-07-07 17:00:00	33.3	30	4.3	3	35	northeast wind	947	30	0	75
2021-07-07 18:00:00	32.6	29	3.6	3	37	northeast wind	947	30	0	75
2021-07-07 19:00:00	32.4	31	2.5	2	150	southeast wind	948	30	0	80
2021-07-07 20:00:00	30.6	43	1.7	2	129	southeast wind	948	30	0	80
2021-07-07 21:00:00	30	44	3	2	329	northwest wind	948	28.7	0	0
2021-07-07 22:00:00	27.4	56	1	1	54	northeast wind	948	22.1	0	0
2021-07-07 23:00:00	25.7	63	2.3	2	240	southwest wind	949	19.2	0	0
2021-07-08 00:00:00	24.8	64	3.2	2	210	southwest wind	949	19.2	0	55
2021-07-08 01:00:00	23.6	66	2.3	2	212	southwest wind	949	19.2	0	55
2021-07-08 02:00:00	23.1	62	0.6	1	213	southwest wind	949	19.2	0	60
2021-07-08 03:00:00	22.4	63	1.6	2	221	southwest wind	948	19.2	0	40
2021-07-08 04:00:00	22.6	60	4.2	3	247	southwest wind	948	19.2	0	35
2021-07-08 05:00:00	22.2	59	0.4	1	223	southwest wind	948	19.2	0	45
2021-07-08 06:00:00	20.9	63	2.4	2	227	southwest wind	948	30	0	50
2021-07-08 07:00:00	20.9	60	1.7	2	243	southwest wind	948	30	0	50
2021-07-08 08:00:00	20.7	61	0.8	1	203	southwest wind	949	30	0	70

2021-07-08 09:00:00	23.9	53	0.5	1	212	southwest wind	949	30	0	0
2021-07-08 10:00:00	25.7	51	3	2	236	southwest wind	949	29.3	0	0
2021-07-08 11:00:00	28.6	47	0.3	1	206	southwest wind	948	29.3	0	0
2021-07-08 12:00:00	32.7	32	2.4	2	29	northeast wind	948	29.3	0	60
2021-07-08 13:00:00	35.7	24	1.9	2	47	northeast wind	948	29.3	0	50
2021-07-08 14:00:00	35.3	22	1.6	2	25	northeast wind	947	30	0	60
2021-07-08 15:00:00	36.4	23	1.1	1	157	southeast wind	947	30	0	60
2021-07-08 16:00:00	36.6	21	0.7	1	42	northeast wind	946	30	0	75
2021-07-08 17:00:00	36.9	21	2.1	2	135	southeast wind	946	30	0	50
2021-07-08 18:00:00	37.6	16	0.3	1	38	northeast wind	945	30	0	75
2021-07-08 19:00:00	36.9	18	0.8	1	57	northeast wind	945	30	0	80
2021-07-08 20:00:00	35.5	24	0.1	0	30	northeast wind	945	28	0	85
2021-07-08 21:00:00	35.3	23	1.2	1	124	southeast wind	946	28	0	70
2021-07-08 22:00:00	31.6	35	2.5	2	294	northwest wind	946	28	0	0
2021-07-08 23:00:00	29.6	42	2.1	2	295	northwest wind	946	20.5	0	0
2021-07-09 00:00:00	26.9	50	1.9	2	214	southwest wind	946	25	0	0
2021-07-09 01:00:00	25.3	55	0.6	1	207	southwest wind	946	25	0	0
2021-07-09 02:00:00	24.3	58	2	2	210	southwest wind	946	25	0	0
2021-07-09 03:00:00	23.3	58	0.5	1	222	southwest wind	946	25	0	0
2021-07-09 04:00:00	23.5	54	3.1	2	230	southwest wind	945	25	0	0
2021-07-09 05:00:00	22.8	54	0.3	1	236	southwest wind	945	25	0	0
2021-07-09 06:00:00	23	51	0.9	1	327	northwest wind	945	25	0	0
2021-07-09 07:00:00	21.8	56	0.8	1	225	southwest wind	944	25	0	0
2021-07-09 08:00:00	22.4	55	1.8	2	244	southwest wind	945	25	0	0
2021-07-09 09:00:00	23.5	55	0.5	1	227	southwest wind	945	17.3	0	0
2021-07-09 10:00:00	25	56	2.7	2	334	northwest wind	944	20	0	0
2021-07-09 11:00:00	30.1	46	1.1	1	29	northeast wind	944	20	0	0
2021-07-09 12:00:00	33.2	31	0.1	0	113	southeast wind	944	20	0	0
2021-07-09 13:00:00	34.1	25	4.8	3	122	southeast wind	944	20	0	0
2021-07-09 14:00:00	35.1	26	3.1	2	145	southeast wind	943	20	0	0
2021-07-09 15:00:00	35.6	25	2.2	2	44	northeast wind	943	20	0	0
2021-07-09 16:00:00	35.5	26	0.5	1	138	southeast wind	942	20	0	0
2021-07-09 17:00:00	33.2	35	0.4	1	125	southeast wind	942	20	0	75
2021-07-09 18:00:00	22.3	80	1.6	2	209	southwest wind	945	20	5.5	75
2021-07-09 19:00:00	26.6	68	0.1	0	239	southwest wind	944	30	0	80
2021-07-09 20:00:00	28.5	59	1.3	1	334	northwest wind	943	30	0	0
2021-07-09 21:00:00	28	56	1.8	2	155	southeast wind	943	30	0	0
2021-07-09 22:00:00	26.2	63	0.8	1	221	southwest wind	943	30	0	0

2021-07-09 23:00:00	25.1	66	0.4	1	303	northwest wind	943	30	0	0
2021-07-10 00:00:00	23.9	72	0	0	23	northeast wind	944	30	0	0
2021-07-10 01:00:00	22.6	80	0.3	1	235	southwest wind	944	22.1	0	0
2021-07-10 02:00:00	22.1	81	2	2	294	northwest wind	943	30	0	0
2021-07-10 03:00:00	20.9	81	3.2	2	230	southwest wind	943	27.4	0	0
2021-07-10 04:00:00	20.1	83	2.6	2	243	southwest wind	943	30	0	0
2021-07-10 05:00:00	19.9	79	2	2	213	southwest wind	942	30	0	0
2021-07-10 06:00:00	19.3	82	3.2	2	208	southwest wind	942	24.1	0	60
2021-07-10 07:00:00	19	83	2.7	2	233	southwest wind	942	26.9	0	65
2021-07-10 08:00:00	19.3	82	2.1	2	325	northwest wind	942	26.9	0	55
2021-07-10 09:00:00	21.9	73	1.5	1	210	southwest wind	943	26.9	0	0
2021-07-10 10:00:00	25.5	62	0.4	1	239	southwest wind	943	26.9	0	0
2021-07-10 11:00:00	28.9	49	1.5	1	115	southeast wind	943	26.9	0	0
2021-07-10 12:00:00	29.7	43	2.4	2	128	southeast wind	943	26.9	0	0
2021-07-10 13:00:00	31.6	38	0.6	1	154	southeast wind	943	26.9	0	0
2021-07-10 14:00:00	31.3	44	2.5	2	40	northeast wind	943	26.9	0	50
2021-07-10 15:00:00	32.2	36	0	0	29	northeast wind	942	26.9	0	0
2021-07-10 16:00:00	32.2	36	3.2	2	33	northeast wind	941	28.4	0	0
2021-07-10 17:00:00	31.3	39	3.2	2	117	southeast wind	941	28.4	0	70
2021-07-10 18:00:00	29.2	57	2	2	240	southwest wind	942	28.4	0	90
2021-07-10 19:00:00	27.1	62	2.4	2	215	southwest wind	942	28.4	0	85
2021-07-10 20:00:00	21.8	82	2.3	2	203	southwest wind	943	28.4	1	85
2021-07-10 21:00:00	22.6	80	0.5	1	243	southwest wind	943	28.4	0.9	85
2021-07-10 22:00:00	23	75	0.1	0	240	southwest wind	942	28.4	0.1	90
2021-07-10 23:00:00	22.2	78	0.9	1	213	southwest wind	943	28.4	0	85
2021-07-11 00:00:00	23.3	54	5.9	4	227	southwest wind	945	28.4	0	80
2021-07-11 01:00:00	22.1	60	4.1	3	211	southwest wind	946	17.4	0	90
2021-07-11 02:00:00	19.4	79	7.4	4	215	southwest wind	947	30	0.4	85
2021-07-11 03:00:00	18.9	82	3.4	3	204	southwest wind	945	30	0.3	90
2021-07-11 04:00:00	18.8	80	3.2	2	247	southwest wind	945	30	0	60
2021-07-11 05:00:00	19.5	75	2.6	2	226	southwest wind	944	30	0	85
2021-07-11 06:00:00	19.5	76	3.8	3	222	southwest wind	944	30	0	85
2021-07-11 07:00:00	20.2	70	2.4	2	212	southwest wind	944	30	0	90
2021-07-11 08:00:00	19.8	76	2.7	2	332	northwest wind	944	30	0	90
2021-07-11 09:00:00	20.3	75	0.9	1	120	southeast wind	944	30	0	85
2021-07-11 10:00:00	22.2	70	2.8	2	307	northwest wind	944	24.1	0	90
2021-07-11 11:00:00	24.3	58	2.9	2	329	northwest wind	944	30	0	50
2021-07-11 12:00:00	26.1	50	3.2	2	244	southwest wind	944	30	0	50

2021-07-11 13:00:00	27.6	44	1.3	1	299	northwest wind	943	30	0	55
2021-07-11 14:00:00	28.4	45	1.3	1	231	southwest wind	943	30	0	80
2021-07-11 15:00:00	29.6	41	0.1	0	326	northwest wind	943	30	0	0
2021-07-11 16:00:00	31.2	32	1	1	323	northwest wind	942	30	0	0
2021-07-11 17:00:00	31.7	30	0.1	0	242	southwest wind	942	30	0	0
2021-07-11 18:00:00	30.5	38	0.6	1	114	southeast wind	941	25.7	0	75
2021-07-11 19:00:00	30.7	33	0.1	0	135	southeast wind	942	30	0	75
2021-07-11 20:00:00	28.2	38	1.4	1	314	northwest wind	942	30	0	80
2021-07-11 21:00:00	26.6	42	3.9	3	230	southwest wind	944	30	0	95
2021-07-11 22:00:00	23.8	59	4	3	150	southeast wind	945	30	0	90
2021-07-11 23:00:00	22.7	65	1.4	1	25	northeast wind	947	30	0	90
2021-07-12 00:00:00	21.1	75	2.7	2	300	northwest wind	947	30	0	90
2021-07-12 01:00:00	20.2	79	0.3	1	208	southwest wind	947	30	0	85
2021-07-12 02:00:00	19.3	84	0.5	1	217	southwest wind	947	22.8	0	0
2021-07-12 03:00:00	18.4	87	0.9	1	205	southwest wind	947	22.8	0	0
2021-07-12 04:00:00	18.3	87	2.2	2	247	southwest wind	947	29	0	0
2021-07-12 05:00:00	17.4	89	1.6	2	227	southwest wind	947	26.8	0	0
2021-07-12 06:00:00	17.1	90	2.9	2	218	southwest wind	946	27.2	0	0
2021-07-12 07:00:00	17.2	88	0.6	1	203	southwest wind	946	25.9	0	0
2021-07-12 08:00:00	17.4	87	1	1	273	west wind	946	30	0	0
2021-07-12 09:00:00	20	71	1.7	2	227	southwest wind	946	30	0	0
2021-07-12 10:00:00	22.4	62	1.1	1	203	southwest wind	946	30	0	0
2021-07-12 11:00:00	24.5	55	1.5	1	27	northeast wind	946	30	0	0
2021-07-12 12:00:00	25.3	52	2.9	2	40	northeast wind	946	30	0	0
2021-07-12 13:00:00	26.6	47	0.3	1	47	northeast wind	946	30	0	0
2021-07-12 14:00:00	27.3	42	1.4	1	34	northeast wind	945	30	0	0
2021-07-12 15:00:00	27.9	45	1.1	1	29	northeast wind	944	30	0	0
2021-07-12 16:00:00	28.9	42	2.1	2	144	southeast wind	944	30	0	0
2021-07-12 17:00:00	29.7	39	1.7	2	51	northeast wind	943	30	0	0
2021-07-12 18:00:00	29.9	36	1.6	2	27	northeast wind	943	29.8	0	0
2021-07-12 19:00:00	29.7	36	4.4	3	28	northeast wind	943	28.2	0	0
2021-07-12 20:00:00	29.5	30	1.7	2	144	southeast wind	943	28.2	0	0
2021-07-12 21:00:00	28	43	2.6	2	0	north wind	943	28.2	0	0
2021-07-12 22:00:00	25.9	53	0.5	1	63	northeast wind	943	28.2	0	35
2021-07-12 23:00:00	24.3	55	2	2	337	northwest wind	944	28.2	0	40
2021-07-13 00:00:00	22.8	65	2.4	2	211	southwest wind	944	28.2	0	0
2021-07-13 01:00:00	22	64	0.9	1	208	southwest wind	944	30	0	90
2021-07-13 02:00:00	22.2	61	1.6	2	294	northwest wind	944	30	0	45

2021-07-13 03:00:00	22.4	64	1.2	1	214	southwest wind	944	30	0	50
2021-07-13 04:00:00	20.9	72	2.8	2	233	southwest wind	944	30	0.1	45
2021-07-13 05:00:00	20.2	76	2.7	2	218	southwest wind	943	30	0	85
2021-07-13 06:00:00	20.1	70	1.7	2	239	southwest wind	943	30	0	35
2021-07-13 07:00:00	19.1	75	0.1	0	247	southwest wind	943	30	0	40
2021-07-13 08:00:00	19.9	71	2.6	2	244	southwest wind	944	30	0	85
2021-07-13 09:00:00	22.1	61	2	2	214	southwest wind	944	30	0	85
2021-07-13 10:00:00	21.7	60	2.1	2	205	southwest wind	944	30	0	75
2021-07-13 11:00:00	21.5	65	1.5	1	242	southwest wind	943	30	0	80
2021-07-13 12:00:00	23.7	57	1.8	2	217	southwest wind	943	30	0	0
2021-07-13 13:00:00	21.5	37	2.7	2	235	southwest wind	942	30	0	0
2021-07-13 14:00:00	28.6	37	1.9	2	26	northeast wind	942	30	0	0
2021-07-13 15:00:00	29.1	39	4.6	3	124	southeast wind	941	25.2	0	0
2021-07-13 16:00:00	29.7	40	0.2	0	45	northeast wind	941	25.2	0	0
2021-07-13 17:00:00	29.5	42	2.3	2	60	northeast wind	940	25.2	0	0
2021-07-13 18:00:00	30.1	42	0.8	1	157	southeast wind	940	25.2	0	0
2021-07-13 19:00:00	29.9	40	2.6	2	64	northeast wind	940	28.9	0	40
2021-07-13 20:00:00	29	40	2.1	2	151	southeast wind	940	21.5	0	70
2021-07-13 21:00:00	28	45	2.3	2	240	southwest wind	941	30	0	0
2021-07-13 22:00:00	26.8	38	0.7	1	316	northwest wind	941	22.6	0	75
2021-07-13 23:00:00	20.1	75	5.1	3	221	southwest wind	945	30	2.6	85
2021-07-14 00:00:00	18.9	85	0	0	315	northwest wind	945	30	2.3	95
2021-07-14 01:00:00	19.5	82	3.2	2	230	southwest wind	946	30	0.4	95
2021-07-14 02:00:00	19.6	85	0.4	1	239	southwest wind	945	30	0	95
2021-07-14 03:00:00	19	89	0.5	1	224	southwest wind	945	30	0	75
2021-07-14 04:00:00	18.5	91	2.1	2	222	southwest wind	945	30	0	55
2021-07-14 05:00:00	18.7	89	1.6	2	326	northwest wind	944	30	0	75
2021-07-14 06:00:00	18.8	86	2.6	2	223	southwest wind	944	30	0	80
2021-07-14 07:00:00	19	83	1.5	1	307	northwest wind	944	30	0	80
2021-07-14 08:00:00	19.7	81	2.2	2	215	southwest wind	945	30	0	85
2021-07-14 09:00:00	19.2	86	2.5	2	46	northeast wind	945	30	0.2	85
2021-07-14 10:00:00	20.9	79	1	1	296	northwest wind	945	30	0	55
2021-07-14 11:00:00	23.8	53	1.4	1	322	northwest wind	945	30	0	57
2021-07-14 12:00:00	24.7	52	2.4	2	124	southeast wind	945	30	0	75
2021-07-14 13:00:00	25.8	46	2.1	2	40	northeast wind	945	30	0	0
2021-07-14 14:00:00	26.4	47	3.6	3	24	northeast wind	944	30	0	0
2021-07-14 15:00:00	27.5	46	0.4	1	133	southeast wind	944	30	0	0
2021-07-14 16:00:00	28	45	0.4	1	125	southeast wind	943	30	0	0

2021-07-14 17:00:00	28.7	40	2.2	2	33	northeast wind	943	30	0	0
2021-07-14 18:00:00	29.7	39	1.9	2	330	northwest wind	943	30	0	0
2021-07-14 19:00:00	29	41	3.2	2	131	southeast wind	943	19.9	0	81
2021-07-14 20:00:00	24.6	58	2	2	322	northwest wind	945	30	0	96
2021-07-14 21:00:00	23.3	66	1.7	2	126	southeast wind	946	30	0.1	98
2021-07-14 22:00:00	22.5	65	2.6	2	230	southwest wind	946	30	0	66
2021-07-14 23:00:00	21.8	71	2.8	2	215	southwest wind	947	30	0	81
2021-07-15 00:00:00	20.5	76	2.9	2	224	southwest wind	948	30	0	50
2021-07-15 01:00:00	20.2	76	3	2	231	southwest wind	947	30	0	55
2021-07-15 02:00:00	20.2	74	1.4	1	302	northwest wind	947	30	0	54
2021-07-15 03:00:00	20.1	74	2.1	2	235	southwest wind	947	30	0	82
2021-07-15 04:00:00	19.7	75	0.8	1	217	southwest wind	947	30	0	75
2021-07-15 05:00:00	19	77	2.6	2	226	southwest wind	946	30	0	59
2021-07-15 06:00:00	18.3	81	0.9	1	298	northwest wind	947	30	0	61
2021-07-15 07:00:00	17.5	86	3.1	2	121	southeast wind	947	30	0	53
2021-07-15 08:00:00	18	84	1.7	2	297	northwest wind	947	30	0	61
2021-07-15 09:00:00	20	76	1.2	1	212	southwest wind	947	30	0	58
2021-07-15 10:00:00	23.5	59	3.2	2	130	southeast wind	947	30	0	1
2021-07-15 11:00:00	25	55	3	2	115	southeast wind	947	30	0	22
2021-07-15 12:00:00	26	46	0	0	122	southeast wind	947	30	0	0
2021-07-15 13:00:00	26.9	43	0.1	0	121	southeast wind	947	30	0	61
2021-07-15 14:00:00	28	40	1.8	2	128	southeast wind	946	30	0	56
2021-07-15 15:00:00	28.6	40	0.2	0	115	southeast wind	946	30	0	85
2021-07-15 16:00:00	29.2	39	1.2	1	133	southeast wind	946	30	0	78
2021-07-15 17:00:00	27.2	42	2.9	2	227	southwest wind	947	20.4	0	90
2021-07-15 18:00:00	20.9	80	3.2	2	308	northwest wind	948	30	0.8	90
2021-07-15 19:00:00	25.7	64	0.4	1	215	southwest wind	947	30	0	98
2021-07-15 20:00:00	25.8	56	0.7	1	217	southwest wind	947	30	0	78
2021-07-15 21:00:00	25.5	62	0.4	1	242	southwest wind	947	26.3	0	2
2021-07-15 22:00:00	22.7	77	1.7	2	246	southwest wind	948	25.9	0	42
2021-07-15 23:00:00	21.8	75	0.7	1	222	southwest wind	949	21.1	0	48
2021-07-16 00:00:00	21.6	75	2.2	2	245	southwest wind	948	21.1	0	50
2021-07-16 01:00:00	20.8	76	2.8	2	204	southwest wind	948	21.1	0	50
2021-07-16 02:00:00	20.6	72	1.3	1	211	southwest wind	948	30	0	22
2021-07-16 03:00:00	19.8	71	2.9	2	239	southwest wind	948	30	0	46
2021-07-16 04:00:00	19.2	70	1.2	1	290	west wind	948	30	0	77
2021-07-16 05:00:00	19	69	1.8	2	227	southwest wind	947	30	0	47
2021-07-16 06:00:00	18.8	68	1.4	1	205	southwest wind	947	30	0	68

2021-07-16 07:00:00	18.7	68	0.9	1	213	southwest wind	947	30	0	49
2021-07-16 08:00:00	18.7	74	1.7	2	227	southwest wind	947	30	0	10
2021-07-16 09:00:00	21.2	64	1	1	227	southwest wind	948	30	0	80
2021-07-16 10:00:00	23.9	57	2.7	2	333	northwest wind	947	30	0	50
2021-07-16 11:00:00	27	44	0.5	1	52	northeast wind	947	30	0	12
2021-07-16 12:00:00	28.1	39	0.8	1	136	southeast wind	947	30	0	72
2021-07-16 13:00:00	27.2	45	1.2	1	157	southeast wind	947	30	0	80
2021-07-16 14:00:00	27.4	44	2.1	2	115	southeast wind	948	30	0	85
2021-07-16 15:00:00	26.7	52	1.4	1	223	southwest wind	948	30	0	80
2021-07-16 16:00:00	27.1	43	3.8	3	321	northwest wind	948	30	0	62
2021-07-16 17:00:00	28.4	40	1.3	1	297	northwest wind	948	30	0	69
2021-07-16 18:00:00	27.4	43	1.8	2	314	northwest wind	948	26.5	0	100
2021-07-16 19:00:00	26.7	43	0.1	0	115	southeast wind	949	29.2	0	99
2021-07-16 20:00:00	25.4	49	4.5	3	122	southeast wind	950	26.2	0	90
2021-07-16 21:00:00	24.6	49	4.3	3	144	southeast wind	950	30	0	80
2021-07-16 22:00:00	22.6	56	2.6	2	229	southwest wind	951	29.3	0	78
2021-07-16 23:00:00	21.5	63	0	0	0	north wind	952	24.6	0	90
2021-07-17 00:00:00	20.9	68	0.4	1	233	southwest wind	952	24.6	0	90
2021-07-17 01:00:00	20	73	2.7	2	226	southwest wind	952	24.6	0	92
2021-07-17 02:00:00	19.2	76	0	0	219	southwest wind	952	24.6	0	71
2021-07-17 03:00:00	18.9	77	0	0	231	southwest wind	952	24.6	0	80
2021-07-17 04:00:00	18.4	78	1.1	1	235	southwest wind	952	12.2	0	70
2021-07-17 05:00:00	17.8	81	0.2	0	204	southwest wind	952	30	0	52
2021-07-17 06:00:00	17.4	84	2.2	2	242	southwest wind	952	30	0	96
2021-07-17 07:00:00	17	86	2.7	2	216	southwest wind	953	30	0	62
2021-07-17 08:00:00	17.6	83	2	2	215	southwest wind	953	30	0	73
2021-07-17 09:00:00	19.6	75	1.4	1	243	southwest wind	953	30	0	88
2021-07-17 10:00:00	22.4	66	3.2	2	239	southwest wind	953	30	0	79
2021-07-17 11:00:00	25	59	0.1	0	336	northwest wind	953	30	0	83
2021-07-17 12:00:00	28	40	2.7	2	117	southeast wind	953	23.3	0	61
2021-07-17 13:00:00	28.1	42	1.4	1	29	northeast wind	953	30	0	19
2021-07-17 14:00:00	28.3	36	3.6	3	38	northeast wind	953	30	0	0
2021-07-17 15:00:00	29.3	36	0.1	0	23	northeast wind	952	30	0	0
2021-07-17 16:00:00	29.8	32	1.9	2	51	northeast wind	952	30	0	0
2021-07-17 17:00:00	30.1	30	1.6	2	116	southeast wind	951	30	0	55
2021-07-17 18:00:00	30	28	0.4	1	29	northeast wind	951	30	0	50
2021-07-17 19:00:00	29.7	30	2.7	2	157	southeast wind	951	30	0	50
2021-07-17 20:00:00	28.5	35	1.6	2	145	southeast wind	950	30	0	55

2021-07-17 21:00:00	28	36	3.1	2	44	northeast wind	951	30	0	90
2021-07-17 22:00:00	26.4	39	0.7	1	293	northwest wind	951	16.7	0	88
2021-07-17 23:00:00	21.9	75	0.5	1	301	northwest wind	952	30	0	80
2021-07-18 00:00:00	21.5	70	1.5	1	207	southwest wind	954	22.5	1.2	78
2021-07-18 01:00:00	19.8	79	2.6	2	326	northwest wind	954	22.5	0.4	100
2021-07-18 02:00:00	19.5	80	1.5	1	220	southwest wind	953	22.5	0.2	94
2021-07-18 03:00:00	19	85	0.1	0	226	southwest wind	953	22.5	0	91
2021-07-18 04:00:00	18.8	86	0.6	1	242	southwest wind	952	23.5	0.3	100
2021-07-18 05:00:00	18.7	86	2.6	2	245	southwest wind	953	22.1	0.1	100
2021-07-18 06:00:00	17.9	92	2.3	2	218	southwest wind	952	30	0.3	96
2021-07-18 07:00:00	17.4	92	2.7	2	220	southwest wind	952	30	0.1	9
2021-07-18 08:00:00	18.2	91	1.6	2	230	southwest wind	952	30	0	93
2021-07-18 09:00:00	20.2	79	2	2	242	southwest wind	952	30	0	14
2021-07-18 10:00:00	22.6	69	1.9	2	120	southeast wind	952	30	0	0
2021-07-18 11:00:00	24.4	57	0.3	1	237	southwest wind	951	30	0	0
2021-07-18 12:00:00	25.9	46	2.3	2	142	southeast wind	951	30	0	0
2021-07-18 13:00:00	26.9	47	1	1	148	southeast wind	951	30	0	0
2021-07-18 14:00:00	27	40	0.9	1	135	southeast wind	950	30	0	0
2021-07-18 15:00:00	27.7	43	2.1	2	32	northeast wind	949	30	0	0
2021-07-18 16:00:00	27.6	42	1	1	58	northeast wind	948	30	0	0
2021-07-18 17:00:00	28.8	37	2.6	2	64	northeast wind	948	30	0	0
2021-07-18 18:00:00	29.1	39	0.6	1	115	southeast wind	947	30	0	2
2021-07-18 19:00:00	28.9	36	1.1	1	140	southeast wind	947	30	0	31
2021-07-18 20:00:00	28.6	32	2.6	2	142	southeast wind	946	30	0	73
2021-07-18 21:00:00	27.7	33	2.8	2	123	southeast wind	947	27.8	0	84
2021-07-18 22:00:00	25.6	46	1.3	1	295	northwest wind	947	20.4	0	100
2021-07-18 23:00:00	24.1	52	0.8	1	313	northwest wind	947	20.4	0	100
2021-07-19 00:00:00	22.9	50	2.6	2	212	southwest wind	947	26.8	0	90
2021-07-19 01:00:00	21	66	2.6	2	219	southwest wind	947	30	0	89
2021-07-19 02:00:00	20.7	65	2.3	2	319	northwest wind	947	30	0	50
2021-07-19 03:00:00	19.7	69	1.7	2	204	southwest wind	947	30	0	31
2021-07-19 04:00:00	18.7	78	3.1	2	220	southwest wind	947	30	0	69
2021-07-19 05:00:00	18.1	82	0.8	1	216	southwest wind	947	30	0	67
2021-07-19 06:00:00	17.8	82	2.5	2	230	southwest wind	947	30	0	60
2021-07-19 07:00:00	17.2	84	0.8	1	243	southwest wind	947	29.4	0	0
2021-07-19 08:00:00	17.9	81	3.1	2	236	southwest wind	947	30	0	4
2021-07-19 09:00:00	20.6	71	0.1	0	238	southwest wind	947	30	0	0
2021-07-19 10:00:00	24.2	56	2.3	2	226	southwest wind	947	30	0	0

2021-07-19 11:00:00	27.2	48	1.1	1	212	southwest wind	946	30	0	0
2021-07-19 12:00:00	29	39	0	0	206	southwest wind	946	30	0	0
2021-07-19 13:00:00	30.5	32	3	2	41	northeast wind	945	30	0	0
2021-07-19 14:00:00	30.9	33	3.2	2	123	southeast wind	945	30	0	0
2021-07-19 15:00:00	31.1	36	1.3	1	125	southeast wind	944	30	0	0
2021-07-19 16:00:00	31.9	30	4.1	3	66	northeast wind	944	30	0	7
2021-07-19 17:00:00	32.7	28	3	2	141	southeast wind	943	30	0	0
2021-07-19 18:00:00	32.8	20	1.8	2	325	northwest wind	943	30	0	55
2021-07-19 19:00:00	33	30	2.4	2	300	northwest wind	942	30	0	0
2021-07-19 20:00:00	31.3	33	0.9	1	139	southeast wind	942	30	0	28
2021-07-19 21:00:00	29.9	34	0.5	1	205	southwest wind	942	30	0	57
2021-07-19 22:00:00	30	23	3.8	3	246	southwest wind	943	30	0	62
2021-07-19 23:00:00	28.4	30	8.5	5	228	southwest wind	943	30	0	63
2021-07-20 00:00:00	26.9	34	3.9	3	310	northwest wind	944	30	0	72
2021-07-20 01:00:00	25.7	40	4.1	3	218	southwest wind	944	30	0	88
2021-07-20 02:00:00	25	42	5.3	3	246	southwest wind	944	30	0	74
2021-07-20 03:00:00	24.3	41	3.7	3	237	southwest wind	943	30	0	58
2021-07-20 04:00:00	23.3	45	4	3	227	southwest wind	943	30	0	0
2021-07-20 05:00:00	22.3	47	2.7	2	229	southwest wind	943	30	0	0
2021-07-20 06:00:00	21.5	48	1.8	2	235	southwest wind	943	30	0	0
2021-07-20 07:00:00	19.7	57	2.4	2	206	southwest wind	943	30	0	0
2021-07-20 08:00:00	19.7	58	0.3	1	206	southwest wind	943	30	0	0
2021-07-20 09:00:00	21.8	54	0.3	1	213	southwest wind	943	30	0	0
2021-07-20 10:00:00	24.6	49	2.3	2	203	southwest wind	943	30	0	0
2021-07-20 11:00:00	28.2	40	1.4	1	223	southwest wind	943	30	0	0
2021-07-20 12:00:00	30.9	31	0.5	1	235	southwest wind	942	30	0	0
2021-07-20 13:00:00	33.2	23	1.1	1	125	southeast wind	942	30	0	0
2021-07-20 14:00:00	33.8	22	0.7	1	47	northeast wind	941	30	0	0
2021-07-20 15:00:00	33.6	23	0.8	1	61	northeast wind	941	30	0	0
2021-07-20 16:00:00	33.8	23	1.2	1	50	northeast wind	941	30	0	70
2021-07-20 17:00:00	33.7	23	4.5	3	111	east wind	940	30	0	60
2021-07-20 18:00:00	34.1	22	0.1	0	33	northeast wind	940	30	0	0
2021-07-20 19:00:00	34.1	19	1.2	1	34	northeast wind	940	14.1	0	60
2021-07-20 20:00:00	33.9	20	2.1	2	24	northeast wind	939	30	0	60
2021-07-20 21:00:00	32.9	26	2.1	2	48	northeast wind	940	21.1	0	70
2021-07-20 22:00:00	28.1	40	0.6	1	330	northwest wind	940	21.1	0	0
2021-07-20 23:00:00	25.8	44	1.9	2	307	northwest wind	941	22.8	0	0
2021-07-21 00:00:00	23.5	57	1.5	1	237	southwest wind	941	26.8	0	0

2021-07-21 01:00:00	22.5	57	1.6	2	212	southwest wind	941	26.3	0	0
2021-07-21 02:00:00	20.7	64	0.2	0	208	southwest wind	941	30	0	0
2021-07-21 03:00:00	20	62	1.2	1	219	southwest wind	941	30	0	0
2021-07-21 04:00:00	19.7	62	1	1	211	southwest wind	941	30	0	0
2021-07-21 05:00:00	20.1	57	0.6	1	223	southwest wind	941	30	0	60
2021-07-21 06:00:00	20.1	55	1.8	2	209	southwest wind	941	30	0	60
2021-07-21 07:00:00	20.1	54	3.2	2	218	southwest wind	941	30	0	0
2021-07-21 08:00:00	20.6	53	0	0	211	southwest wind	941	30	0	0
2021-07-21 09:00:00	23.6	47	0.9	1	213	southwest wind	941	30	0	0
2021-07-21 10:00:00	26.4	40	4.3	3	228	southwest wind	941	28.6	0	0
2021-07-21 11:00:00	30.1	37	1	1	235	southwest wind	941	24.6	0	80
2021-07-21 12:00:00	32.6	23	2.7	2	37	northeast wind	941	30	0	80
2021-07-21 13:00:00	33.1	27	1.2	1	37	northeast wind	941	30	0	70
2021-07-21 14:00:00	32.9	30	5.3	3	47	northeast wind	940	30	0	60
2021-07-21 15:00:00	32.1	27	0.5	1	56	northeast wind	940	30	0	70
2021-07-21 16:00:00	32.8	26	3.2	2	53	northeast wind	940	30	0	70
2021-07-21 17:00:00	33.6	24	3	2	37	northeast wind	939	30	0	90
2021-07-21 18:00:00	33.1	26	1.2	1	309	northwest wind	939	30	0	100
2021-07-21 19:00:00	31.5	34	0.5	1	219	southwest wind	939	30	0	100
2021-07-21 20:00:00	31	41	0.2	0	208	southwest wind	939	18.9	0	100
2021-07-21 21:00:00	29.9	42	1.4	1	330	northwest wind	939	23.2	0	100
2021-07-21 22:00:00	28.4	36	0.3	1	273	west wind	939	30	0	100
2021-07-21 23:00:00	27	54	1.5	1	231	southwest wind	940	22.3	0	100
2021-07-22 00:00:00	26.5	56	0.4	1	331	northwest wind	940	15.8	0	100
2021-07-22 01:00:00	26.9	45	2.7	2	210	southwest wind	940	15.8	0	100
2021-07-22 02:00:00	26.7	42	1	1	222	southwest wind	940	30	0	100
2021-07-22 03:00:00	27.3	39	0.2	0	46	northeast wind	940	30	0	100
2021-07-22 04:00:00	26.8	40	1.6	2	44	northeast wind	940	30	0	90
2021-07-22 05:00:00	27.1	40	0.1	0	325	northwest wind	940	30	0	100
2021-07-22 06:00:00	26.2	46	0.6	1	337	northwest wind	940	30	0	100
2021-07-22 07:00:00	24.4	54	0.2	0	238	southwest wind	941	30	0	100
2021-07-22 08:00:00	23.6	58	2.9	2	241	southwest wind	941	30	0	100
2021-07-22 09:00:00	23.3	62	2	2	216	southwest wind	942	30	0	100
2021-07-22 10:00:00	25.2	50	3	2	138	southeast wind	942	30	0	100
2021-07-22 11:00:00	24.4	59	1.3	1	57	northeast wind	943	30	0	100
2021-07-22 12:00:00	24.9	53	1.3	1	27	northeast wind	943	30	0	100
2021-07-22 13:00:00	24.3	57	0.1	0	156	southeast wind	943	30	0	100
2021-07-22 14:00:00	24.1	59	1	1	36	northeast wind	944	30	0	100

2021-07-22 15:00:00	24.3	63	0	0	28	northeast wind	943	30	0	100
2021-07-22 16:00:00	24.3	65	2.2	2	28	northeast wind	943	30	0	100
2021-07-22 17:00:00	24.2	63	1.3	1	144	southeast wind	943	30	0	100
2021-07-22 18:00:00	24.8	57	1.9	2	56	northeast wind	942	30	0	100
2021-07-22 19:00:00	25	59	3.1	2	125	southeast wind	942	30	0	100
2021-07-22 20:00:00	25.2	61	1.7	2	319	northwest wind	942	30	0	100
2021-07-22 21:00:00	24.9	62	2.7	2	302	northwest wind	942	30	0	100
2021-07-22 22:00:00	24.5	62	1.4	1	334	northwest wind	943	30	0	90
2021-07-22 23:00:00	23.1	70	2.9	2	204	southwest wind	943	30	0	90
2021-07-23 00:00:00	22.5	69	1.1	1	240	southwest wind	943	30	0	90
2021-07-23 01:00:00	22	72	0.3	1	207	southwest wind	943	30	0	86
2021-07-23 02:00:00	21.1	76	1.4	1	235	southwest wind	942	30	0	70
2021-07-23 03:00:00	20.1	80	1.6	2	234	southwest wind	942	30	0	60
2021-07-23 04:00:00	19.6	83	1.8	2	213	southwest wind	942	30	0	60
2021-07-23 05:00:00	19.1	84	1.8	2	224	southwest wind	941	30	0	60
2021-07-23 06:00:00	18.9	81	1.4	1	203	southwest wind	941	30	0	60
2021-07-23 07:00:00	18.8	79	2.6	2	215	southwest wind	942	30	0	60
2021-07-23 08:00:00	18.8	79	0.9	1	208	southwest wind	942	30	0	60
2021-07-23 09:00:00	21.7	65	3.1	2	241	southwest wind	942	30	0	0
2021-07-23 10:00:00	24.8	56	1.3	1	223	southwest wind	942	30	0	0
2021-07-23 11:00:00	27.8	45	2.4	2	229	southwest wind	942	30	0	0
2021-07-23 12:00:00	30	39	2.9	2	309	northwest wind	942	30	0	0
2021-07-23 13:00:00	32.2	28	1.7	2	123	southeast wind	942	30	0	0
2021-07-23 14:00:00	33.1	27	3.1	2	29	northeast wind	941	30	0	0
2021-07-23 15:00:00	33.3	21	1.8	2	148	southeast wind	941	30	0	0
2021-07-23 16:00:00	33.7	19	1.9	2	28	northeast wind	941	30	0	0
2021-07-23 17:00:00	35	17	2.5	2	146	southeast wind	940	30	0	0
2021-07-23 18:00:00	35.3	15	2.9	2	212	southwest wind	940	30	0	70
2021-07-23 19:00:00	35.4	16	2.6	2	326	northwest wind	940	30	0	70
2021-07-23 20:00:00	34.5	23	0.8	1	115	southeast wind	940	30	0	70
2021-07-23 21:00:00	32.3	31	0.9	1	216	southwest wind	941	16.8	0	70
2021-07-23 22:00:00	27.9	35	1.2	1	234	southwest wind	942	16.8	0	70
2021-07-23 23:00:00	26.1	42	1.2	1	212	southwest wind	943	30	0	0
2021-07-24 00:00:00	24.6	48	3	2	243	southwest wind	943	30	0	0
2021-07-24 01:00:00	23.1	56	2.7	2	220	southwest wind	943	30	0	70
2021-07-24 02:00:00	22	60	0.8	1	234	southwest wind	944	23.7	0	70
2021-07-24 03:00:00	21.2	63	1.5	1	241	southwest wind	944	30	0	0
2021-07-24 04:00:00	20.6	63	0.2	0	220	southwest wind	944	30	0	0

2021-07-24 05:00:00	19.6	67	0.2	0	212	southwest wind	944	30	0	70
2021-07-24 06:00:00	18.7	70	1.8	2	215	southwest wind	944	30	0	70
2021-07-24 07:00:00	19	65	2.7	2	203	southwest wind	944	30	0	70
2021-07-24 08:00:00	20	59	2.5	2	238	southwest wind	945	30	0	0
2021-07-24 09:00:00	22.8	49	3.5	3	259	west wind	945	30	0	70
2021-07-24 10:00:00	25.1	45	0.1	0	209	southwest wind	945	30	0	0
2021-07-24 11:00:00	28.6	38	2	2	218	southwest wind	945	29.7	0	0
2021-07-24 12:00:00	31.6	34	2.3	2	44	northeast wind	945	28.7	0	0
2021-07-24 13:00:00	32.6	31	1.4	1	38	northeast wind	944	29.3	0	0
2021-07-24 14:00:00	33.4	29	0.7	1	117	southeast wind	944	30	0	0
2021-07-24 15:00:00	34.2	27	2	2	144	southeast wind	943	30	0	0
2021-07-24 16:00:00	34.9	22	2.9	2	48	northeast wind	943	30	0	0
2021-07-24 17:00:00	35.6	22	1.3	1	141	southeast wind	943	30	0	0
2021-07-24 18:00:00	35.8	22	1.5	1	130	southeast wind	943	30	0	0
2021-07-24 19:00:00	35.8	22	0	0	156	southeast wind	943	30	0	0
2021-07-24 20:00:00	35.4	22	0.2	0	84	east wind	943	27.9	0	70
2021-07-24 21:00:00	34.4	25	1.6	2	118	southeast wind	943	25.3	0	0
2021-07-24 22:00:00	29.7	40	2.8	2	334	northwest wind	944	25.3	0	0
2021-07-24 23:00:00	27.7	43	1.4	1	301	northwest wind	945	25.3	0	0
2021-07-25 00:00:00	26	47	3	2	258	west wind	945	24.8	0	0
2021-07-25 01:00:00	24	57	0.3	1	313	northwest wind	945	26	0	70
2021-07-25 02:00:00	24.5	49	1.3	1	212	southwest wind	946	29.1	0	0
2021-07-25 03:00:00	22.7	56	0	0	209	southwest wind	946	29.1	0	70
2021-07-25 04:00:00	21	67	0.1	0	214	southwest wind	946	30	0	70
2021-07-25 05:00:00	20.6	66	2.2	2	228	southwest wind	946	30	0	70
2021-07-25 06:00:00	19.7	70	0.8	1	246	southwest wind	946	30	0	70
2021-07-25 07:00:00	19.3	69	2.4	2	242	southwest wind	946	30	0	70
2021-07-25 08:00:00	19.9	66	2.2	2	207	southwest wind	947	27.1	0	70
2021-07-25 09:00:00	22.1	60	1.4	1	207	southwest wind	947	30	0	70
2021-07-25 10:00:00	25.7	54	2.7	2	238	southwest wind	947	30	0	0
2021-07-25 11:00:00	30.6	35	1	1	311	northwest wind	947	26.3	0	0
2021-07-25 12:00:00	32.4	28	1.6	2	26	northeast wind	947	24.7	0	0
2021-07-25 13:00:00	33	31	2.9	2	24	northeast wind	947	30	0	0
2021-07-25 14:00:00	33.9	28	2.2	2	124	southeast wind	946	26.4	0	0
2021-07-25 15:00:00	34.2	24	2.3	2	24	northeast wind	945	28.2	0	0
2021-07-25 16:00:00	35.1	23	1.1	1	116	southeast wind	945	30	0	0
2021-07-25 17:00:00	35.4	20	0.8	1	31	northeast wind	944	30	0	0
2021-07-25 18:00:00	35.7	23	0.1	0	35	northeast wind	944	30	0	0

2021-07-25 19:00:00	35.9	22	2.7	2	142	southeast wind	944	30	0	0
2021-07-25 20:00:00	35.1	26	3.2	2	114	southeast wind	944	30	0	0
2021-07-25 21:00:00	33.7	29	0.2	0	135	southeast wind	945	30	0	0
2021-07-25 22:00:00	30.7	37	0	0	302	northwest wind	945	30	0	0
2021-07-25 23:00:00	29.3	34	1.9	2	225	southwest wind	946	21	0	0
2021-07-26 00:00:00	27.1	43	1.3	1	330	northwest wind	946	13.2	0	0
2021-07-26 01:00:00	26.5	37	2.7	2	299	northwest wind	946	17	0	70
2021-07-26 02:00:00	24.2	50	2.7	2	316	northwest wind	946	23.2	0	0
2021-07-26 03:00:00	21.9	60	0.6	1	241	southwest wind	946	24.5	0	70
2021-07-26 04:00:00	21	65	0.9	1	208	southwest wind	946	25.2	0	70
2021-07-26 05:00:00	20.9	65	3.2	2	222	southwest wind	946	28.9	0	70
2021-07-26 06:00:00	21.1	61	0.7	1	222	southwest wind	946	30	0	70
2021-07-26 07:00:00	20.4	64	2	2	244	southwest wind	946	30	0	70
2021-07-26 08:00:00	20.4	65	2.2	2	219	southwest wind	946	30	0	70
2021-07-26 09:00:00	23.5	55	1.8	2	230	southwest wind	946	28	0	70
2021-07-26 10:00:00	26.6	50	2.9	2	234	southwest wind	946	24.4	0	70
2021-07-26 11:00:00	30.5	39	1.1	1	309	northwest wind	946	25.2	0	0
2021-07-26 12:00:00	33.1	28	0.5	1	320	northwest wind	946	25.2	0	0
2021-07-26 13:00:00	35	22	0.4	1	316	northwest wind	945	30	0	0
2021-07-26 14:00:00	35.8	20	1	1	215	southwest wind	944	30	0	0
2021-07-26 15:00:00	36.4	22	1.8	2	55	northeast wind	944	30	0	0
2021-07-26 16:00:00	36.5	20	0.1	0	116	southeast wind	943	30	0	0
2021-07-26 17:00:00	37.3	21	0.2	0	149	southeast wind	943	30	0	0
2021-07-26 18:00:00	37.2	18	0.8	1	116	southeast wind	942	30	0	0
2021-07-26 19:00:00	37	18	2.3	2	61	northeast wind	942	30	0	0
2021-07-26 20:00:00	36.2	21	0.3	1	29	northeast wind	942	30	0	70
2021-07-26 21:00:00	34	28	0	0	125	southeast wind	942	25.9	0	0
2021-07-26 22:00:00	29.5	41	1.4	1	324	northwest wind	942	12.6	0	0
2021-07-26 23:00:00	27.1	53	3.2	2	328	northwest wind	942	7.6	0	0
2021-07-27 00:00:00	25.3	57	0.4	1	224	southwest wind	942	7.6	0	0
2021-07-27 01:00:00	24.1	57	2	2	204	southwest wind	942	24.2	0	0
2021-07-27 02:00:00	24.7	49	2.7	2	230	southwest wind	942	25.3	0	70
2021-07-27 03:00:00	25	45	0.5	1	238	southwest wind	942	30	0	0
2021-07-27 04:00:00	24.1	46	1.8	2	215	southwest wind	941	30	0	70
2021-07-27 05:00:00	23.2	48	1.7	2	219	southwest wind	941	30	0	70
2021-07-27 06:00:00	23.1	46	3.1	2	233	southwest wind	941	30	0	70
2021-07-27 07:00:00	22.6	48	2	2	216	southwest wind	941	30	0	70
2021-07-27 08:00:00	23	47	2.5	2	237	southwest wind	941	30	0	70

2021-07-27 09:00:00	23	53	1.7	2	211	southwest wind	941	30	0	80
2021-07-27 10:00:00	26.6	50	2.9	2	226	southwest wind	941	24.2	0	90
2021-07-27 11:00:00	31.6	33	2.9	2	203	southwest wind	941	24.2	0	70
2021-07-27 12:00:00	34.1	25	2.7	2	73	east wind	940	24.2	0	0
2021-07-27 13:00:00	33.5	30	3.6	3	40	northeast wind	940	18.3	0	0
2021-07-27 14:00:00	34.1	28	4.2	3	29	northeast wind	940	18.3	0	0
2021-07-27 15:00:00	34.4	27	4.2	3	129	southeast wind	940	12.6	0	70
2021-07-27 16:00:00	34.3	30	3.9	3	149	southeast wind	939	12.6	0	70
2021-07-27 17:00:00	34.9	28	3.8	3	138	southeast wind	939	12.6	0	70
2021-07-27 18:00:00	34.4	28	2.4	2	126	southeast wind	939	12.6	0	80
2021-07-27 19:00:00	33.5	33	1.8	2	135	southeast wind	939	12.6	0	80
2021-07-27 20:00:00	32.5	36	1.3	1	135	southeast wind	939	12.6	0.1	80
2021-07-27 21:00:00	34.5	53	0.9	1	304	northwest wind	940	12.6	0	70
2021-07-27 22:00:00	25.7	70	2.2	2	330	northwest wind	940	12.6	0.9	70
2021-07-27 23:00:00	26.2	59	0.2	0	244	southwest wind	941	12.6	0	70
2021-07-28 00:00:00	28	43	0.7	1	25	northeast wind	941	12.6	0	70
2021-07-28 01:00:00	27	40	0.1	0	150	southeast wind	941	12.6	0	70
2021-07-28 02:00:00	25	49	2.6	2	300	northwest wind	942	12.6	0	70
2021-07-28 03:00:00	23.2	52	0.2	0	128	southeast wind	942	12.6	0	0
2021-07-28 04:00:00	23.6	46	1.9	2	171	south wind	942	12.6	0	70
2021-07-28 05:00:00	24.1	42	2.9	2	142	southeast wind	942	12.6	0	0
2021-07-28 06:00:00	23.8	43	0.3	1	115	southeast wind	942	12.6	0	70
2021-07-28 07:00:00	22.8	47	1.5	1	129	southeast wind	943	12.6	0	0
2021-07-28 08:00:00	22.7	43	2.4	2	127	southeast wind	944	12.6	0	0
2021-07-28 09:00:00	23.7	41	1.6	2	55	northeast wind	944	12.6	0	70
2021-07-28 10:00:00	24.6	37	1	1	119	southeast wind	944	12.6	0	70
2021-07-28 11:00:00	25.6	37	2.1	2	151	southeast wind	944	12.6	0	0
2021-07-28 12:00:00	27.2	35	0.9	1	63	northeast wind	944	12.6	0	60
2021-07-28 13:00:00	28	40	0.8	1	130	southeast wind	943	12.6	0	60
2021-07-28 14:00:00	29.6	40	1.4	1	135	southeast wind	943	12.6	0	60
2021-07-28 15:00:00	30.3	38	2	2	146	southeast wind	942	12.6	0	60
2021-07-28 16:00:00	31	32	3.2	2	35	northeast wind	942	12.6	0	0
2021-07-28 17:00:00	31.5	32	2.1	2	122	southeast wind	941	12.6	0	0
2021-07-28 18:00:00	31.4	30	3.8	3	134	southeast wind	941	12.6	0	60
2021-07-28 19:00:00	31.2	29	2.1	2	147	southeast wind	941	12.6	0	60
2021-07-28 20:00:00	31.1	28	0.2	0	121	southeast wind	941	12.6	0	60
2021-07-28 21:00:00	30.4	29	2.3	2	43	northeast wind	941	12.6	0	60
2021-07-28 22:00:00	29.1	33	2.6	2	135	southeast wind	942	12.6	0	60

2021-07-28 23:00:00	26.7	43	1.3	1	309	northwest wind	942	12.6	0	60
2021-07-29 00:00:00	26.7	43	1.3	1	309	northwest wind	942	12.6	0	60
2021-07-29 01:00:00	26.7	43	1.3	1	309	northwest wind	942	12.6	0	0
2021-07-29 02:00:00	26.7	43	1.3	1	309	northwest wind	942	12.6	0	60
2021-07-29 03:00:00	21.2	52	3	2	326	northwest wind		12.6	0	0
2021-07-29 04:00:00	20.3	53	3.3	2	327	northwest wind		12.6	0	0
2021-07-29 05:00:00	19	62	0	0	307	northwest wind	943	12.6	0	60
2021-07-29 06:00:00	17.7	71	0.9	1	210	southwest wind	943	12.6	0	60
2021-07-29 07:00:00	16.7	78	3.1	2	204	southwest wind	944	12.6	0	0
2021-07-29 08:00:00	18.3	69	2.8	2	252	west wind	944	12.6	0	0
2021-07-29 09:00:00	21.3	55	0.2	0	216	southwest wind	944	12.6	0	0
2021-07-29 10:00:00	25	35	0	0	203	southwest wind	944	12.6	0	0
2021-07-29 11:00:00	27.1	28	2.5	2	120	southeast wind	944	12.6	0	0
2021-07-29 12:00:00	28.3	24	1.6	2	116	southeast wind	944	12.6	0	0
2021-07-29 13:00:00	29.1	22	3.2	2	113	southeast wind	943	12.6	0	0
2021-07-29 14:00:00	29.3	23	3.1	2	27	northeast wind	943	12.6	0	0
2021-07-29 15:00:00	29.8	25	1.4	1	42	northeast wind	942	12.6	0	0
2021-07-29 16:00:00	30.5	26	2.8	2	134	southeast wind	941	12.6	0	0
2021-07-29 17:00:00	30.8	26	0.3	1	135	southeast wind	942	12.6	0	0
2021-07-29 18:00:00	31.4	25	1.4	1	0	north wind	941	12.6	0	70
2021-07-29 19:00:00	31.1	27	2.3	2	128	southeast wind	940	12.6	0	70
2021-07-29 20:00:00	30.4	28	0.3	1	49	northeast wind	940	12.6	0	70
2021-07-29 21:00:00	29.3	30	1.5	1	329	northwest wind	941	12.6	0	70
2021-07-29 22:00:00	26.7	40	0.1	0	236	southwest wind	941	12.6	0	60
2021-07-29 23:00:00	25.1	50	0.2	0	218	southwest wind	941	12.6	0	60
2021-07-30 00:00:00	24.3	55	2	2	240	southwest wind	942	12.6	0	70
2021-07-30 01:00:00	23.9	54	1	1	227	southwest wind	941	12.6	0	90
2021-07-30 02:00:00	22.8	56	2	2	231	southwest wind	942	12.6	0	90
2021-07-30 03:00:00	22.3	57	0.1	0	208	southwest wind	941	12.6	0	100
2021-07-30 04:00:00	22.1	58	2.1	2	245	southwest wind	941	12.6	0	100
2021-07-30 05:00:00	21.4	62	0.7	1	303	northwest wind	941	12.6	0	100
2021-07-30 06:00:00	20.5	68	1	1	203	southwest wind	942	12.6	0	100
2021-07-30 07:00:00	20.8	65	3	2	211	southwest wind	942	12.6	0	100
2021-07-30 08:00:00	21.1	65	1.7	2	209	southwest wind	942	12.6	0	100
2021-07-30 09:00:00	21.2	67	2.1	2	335	northwest wind	943	12.6	0	100
2021-07-30 10:00:00	21.9	68	0.5	1	243	southwest wind	944	12.6	0	100
2021-07-30 11:00:00	24	68	1.4	1	40	northeast wind	944	12.6	0	100
2021-07-30 12:00:00	25.5	46	0	0	145	southeast wind	945	12.6	0	100

2021-07-30 13:00:00	26.6	43	2.2	2	138	southeast wind	945	12.6	0	100
2021-07-30 14:00:00	25.3	43	1	1	36	northeast wind	946	12.6	0	100
2021-07-30 15:00:00	23	59	2.1	2	212	southwest wind	947	12.6	0.2	100
2021-07-30 16:00:00	22.5	61	3.5	3	229	southwest wind	948	12.6	0	100
2021-07-30 17:00:00	23.1	61	1.4	1	179	south wind	948	12.6	0.3	100
2021-07-30 18:00:00	23.4	58	3.2	2	50	northeast wind	948	12.6	0	100
2021-07-30 19:00:00	20.7	66	1.4	1	314	northwest wind	950	12.6	0	100
2021-07-30 20:00:00	18.6	79	1.1	1	302	northwest wind	950	12.6	0.1	100
2021-07-30 21:00:00	17.2	88	1.9	2	222	southwest wind	952	12.6	0.5	100
2021-07-30 22:00:00	16.7	94	1.9	2	239	southwest wind	952	12.6	1.9	100
2021-07-30 23:00:00	16.8	95	1.5	1	240	southwest wind	953	12.6	0	100
2021-07-31 00:00:00	17.1	94	1.1	1	238	southwest wind	953	12.6	0	100
2021-07-31 01:00:00	17.1	94	0.1	0	113	southeast wind	953	12.6	0	100
2021-07-31 02:00:00	17.2	94	2.5	2	227	southwest wind	953	12.6	0	90
2021-07-31 03:00:00	16.9	94	0.4	1	296	northwest wind	953	12.6	0.6	100
2021-07-31 04:00:00	17.3	92	3.2	2	227	southwest wind	953	12.6	0.4	100
2021-07-31 05:00:00	17.1	90	1.8	2	236	southwest wind	953	12.6	0	90
2021-07-31 06:00:00	17	91	2.6	2	228	southwest wind	953	12.6	0	100
2021-07-31 07:00:00	17.1	90	1.2	1	244	southwest wind	953	12.6	0	80
2021-07-31 08:00:00	17.7	84	3.2	2	204	southwest wind	953	12.6	0	70
2021-07-31 09:00:00	19.2	78	2.4	2	214	southwest wind	953	12.6	0	70
2021-07-31 10:00:00	20.2	75	2	2	218	southwest wind	953	12.6	0	70
2021-07-31 11:00:00	21.1	75	0.1	0	203	southwest wind	953	12.6	0	86
2021-07-31 12:00:00	23.5	59	0.4	1	220	southwest wind	952	12.6	0	90
2021-07-31 13:00:00	25.6	37	1.1	1	226	southwest wind	952	12.6	0	60
2021-07-31 14:00:00	26.9	32	0.1	0	150	southeast wind	951	12.6	0	0
2021-07-31 15:00:00	28	30	2	2	293	northwest wind	951	12.6	0	0
2021-07-31 16:00:00	28.7	27	1.7	2	39	northeast wind	951	12.6	0	0
2021-07-31 17:00:00	29	29	0.8	1	126	southeast wind	950	12.6	0	60
2021-07-31 18:00:00	27.7	29	0.2	0	43	northeast wind	950	12.6	0	60
2021-07-31 19:00:00	27.8	30	1.8	2	156	southeast wind	951	12.6	0	60
2021-07-31 20:00:00	28	28	2.1	2	28	northeast wind	951	12.6	0	60
2021-07-31 21:00:00	26.9	32	0.8	1	138	southeast wind	951	12.6	0	60
2021-07-31 22:00:00	23.4	49	3	2	140	southeast wind	952	12.6	0	0
2021-07-31 23:00:00	20.9	66	1.4	1	303	northwest wind	952	12.6	0	0
2021-08-01 00:00:00	19.6	65	3.2	2	302	northwest wind	952	12.6	0	0
2021-08-01 01:00:00	17.2	77	1	1	245	southwest wind	953	12.6	0	0
2021-08-01 02:00:00	17.3	69	2.5	2	212	southwest wind	953	12.6	0	0

2021-08-01 03:00:00	16.2	78	3	2	208	southwest wind	953	12.6	0	0
2021-08-01 04:00:00	15.4	81	1.8	2	218	southwest wind	952	12.6	0	0
2021-08-01 05:00:00	14.6	84	0.3	1	239	southwest wind	952	12.6	0	0
2021-08-01 06:00:00	14.2	85	1.8	2	227	southwest wind	952	12.6	0	0
2021-08-01 07:00:00	14	83	1.5	1	243	southwest wind	952	12.6	0	0
2021-08-01 08:00:00	14.9	77	1.2	1	239	southwest wind	952	12.6	0	0
2021-08-01 09:00:00	17.9	65	0.5	1	239	southwest wind	952	12.6	0	0
2021-08-01 10:00:00	21.1	56	3.1	2	217	southwest wind	952	12.6	0	0
2021-08-01 11:00:00	24.2	38	1	1	157	southeast wind	951	12.6	0	0
2021-08-01 12:00:00	25.5	35	2.4	2	113	southeast wind	951	12.6	0	0
2021-08-01 13:00:00	27.2	32	0.1	0	23	northeast wind	950	12.6	0	0
2021-08-01 14:00:00	28.3	30	2.2	2	127	southeast wind	949	12.6	0	0
2021-08-01 15:00:00	29.2	31	2.4	2	131	southeast wind	948	12.6	0	0
2021-08-01 16:00:00	29.4	27	1.2	1	216	southwest wind	948	12.6	0	0
2021-08-01 17:00:00	29.9	27	0.8	1	309	northwest wind	947	12.6	0	0
2021-08-01 18:00:00	30.5	25	2.9	2	34	northeast wind	947	12.6	0	0
2021-08-01 19:00:00	30.8	24	1	1	141	southeast wind	947	12.6	0	0
2021-08-01 20:00:00	30.8	25	0.5	1	141	southeast wind	946	12.6	0	0
2021-08-01 21:00:00	28.2	30	1.7	2	212	southwest wind	946	12.6	0	0
2021-08-01 22:00:00	29.4	42	2.7	2	310	northwest wind	947	12.6	0	0
2021-08-01 23:00:00	21.4	52	0.4	1	308	northwest wind	947	12.6	0	0
2021-08-02 00:00:00	19.3	62	0.7	1	304	northwest wind	947	12.6	0	0
2021-08-02 01:00:00	18.6	66	1.7	2	227	southwest wind	947	12.6	0	0
2021-08-02 02:00:00	17.3	68	0.7	1	233	southwest wind	948	12.6	0	0
2021-08-02 03:00:00	16.3	76	1.4	1	227	southwest wind	947	12.6	0	0
2021-08-02 04:00:00	15.8	79	1.7	2	231	southwest wind	947	12.6	0	0
2021-08-02 05:00:00	15.5	76	0.3	1	208	southwest wind	947	12.6	0	0
2021-08-02 06:00:00	15.5	76	1.3	1	223	southwest wind	947	12.6	0	0
2021-08-02 07:00:00	14.9	78	2.9	2	209	southwest wind	948	12.6	0	0
2021-08-02 08:00:00	15.3	77	2.9	2	233	southwest wind	948	12.6	0	0
2021-08-02 09:00:00	18	67	3	2	217	southwest wind	948	12.6	0	0
2021-08-02 10:00:00	22.1	57	2.1	2	245	southwest wind	948	12.6	0	0
2021-08-02 11:00:00	26.1	37	1.4	1	326	northwest wind	948	12.6	0	0
2021-08-02 12:00:00	29.8	20	0.3	1	115	southeast wind	948	12.6	0	0
2021-08-02 13:00:00	30.8	22	0.2	0	30	northeast wind	948	12.6	0	0
2021-08-02 14:00:00	31.5	16	0.3	1	122	southeast wind	947	12.6	0	0
2021-08-02 15:00:00	31.7	16	1.5	1	37	northeast wind	947	12.6	0	0
2021-08-02 16:00:00	31.9	19	3.2	2	154	southeast wind	946	12.6	0	0

2021-08-02 17:00:00	32.5	15	2.3	2	24	northeast wind	946	12.6	0	0
2021-08-02 18:00:00	32.5	18	0.2	0	139	southeast wind	946	12.6	0	0
2021-08-02 19:00:00	32.5	14	0.7	1	36	northeast wind	946	12.6	0	0
2021-08-02 20:00:00	31.9	13	2.6	2	154	southeast wind	946	12.6	0	60
2021-08-02 21:00:00	29.1	28	1.6	2	0	north wind	946	12.6	0	0
2021-08-02 22:00:00	24.5	37	0.4	1	319	northwest wind	946	12.6	0	0
2021-08-02 23:00:00	22.1	50	0.2	0	203	southwest wind	947	12.6	0	0
2021-08-03 00:00:00	20.4	64	2.8	2	229	southwest wind	947	12.6	0	0
2021-08-03 01:00:00	19.3	64	0	0	213	southwest wind	947	12.6	0	0
2021-08-03 02:00:00	18.3	64	2.4	2	221	southwest wind	947	12.6	0	60
2021-08-03 03:00:00	18.1	60	2.2	2	245	southwest wind	947	12.6	0	60
2021-08-03 04:00:00	17.5	60	3.1	2	227	southwest wind	947	12.6	0	60
2021-08-03 05:00:00	16.7	62	1.6	2	219	southwest wind	947	12.6	0	0
2021-08-03 06:00:00	16.2	65	1	1	229	southwest wind	947	12.6	0	0
2021-08-03 07:00:00	15.7	67	0	0	219	southwest wind	947	12.6	0	60
2021-08-03 08:00:00	16.2	65	1	1	219	southwest wind	948	12.6	0	60
2021-08-03 09:00:00	18.5	59	1.1	1	238	southwest wind	948	12.6	0	60
2021-08-03 10:00:00	22.7	52	3.2	2	226	southwest wind	948	12.6	0	60
2021-08-03 11:00:00	26.8	41	1.3	1	238	southwest wind	948	12.6	0	80
2021-08-03 12:00:00	30.9	22	1.6	2	335	northwest wind	947	12.6	0	80
2021-08-03 13:00:00	30.4	27	0.4	1	31	northeast wind	947	12.6	0	70
2021-08-03 14:00:00	30.7	24	0.5	1	25	northeast wind	947	12.6	0	80
2021-08-03 15:00:00	31.9	21	1.6	2	136	southeast wind	946	12.6	0	90
2021-08-03 16:00:00	31.8	18	2.8	2	31	northeast wind	946	12.6	0	100
2021-08-03 17:00:00	33.1	17	0.2	0	148	southeast wind	946	12.6	0	80
2021-08-03 18:00:00	32.8	17	2.5	2	44	northeast wind	945	12.6	0	70
2021-08-03 19:00:00	31.7	22	0.8	1	118	southeast wind	945	12.6	0	70
2021-08-03 20:00:00	30.1	34	2.9	2	229	southwest wind	945	12.6	0	60
2021-08-03 21:00:00	28	36	2.9	2	310	northwest wind	945	12.6	0	90
2021-08-03 22:00:00	26.8	42	2.1	2	317	northwest wind	946	12.6	0	60
2021-08-03 23:00:00	24.9	42	0.8	1	204	southwest wind	946	12.6	0	70
2021-08-04 00:00:00	22.9	53	2.2	2	204	southwest wind	946	12.6	0	60
2021-08-04 01:00:00	22.2	54	1.2	1	210	southwest wind	946	12.6	0	60
2021-08-04 02:00:00	21.2	52	0.7	1	243	southwest wind	946	12.6	0	60
2021-08-04 03:00:00	20.1	57	1.7	2	220	southwest wind	946	12.6	0	60
2021-08-04 04:00:00	19.6	57	3	2	214	southwest wind	946	12.6	0	
2021-08-04 05:00:00	20.1	50	1	1	232	southwest wind	946	12.6	0	
2021-08-04 06:00:00	20.4	52	0.4	1	238	southwest wind	946	12.6	0	

2021-08-04 07:00:00	20.4	55	3.2	2	206	southwest wind	946	12.6	0	
2021-08-04 08:00:00	20.8	54	2.8	2	237	southwest wind	946	12.6	0	
2021-08-04 09:00:00	21.8	52	2.4	2	215	southwest wind	946	12.6	0	
2021-08-04 10:00:00	22.9	52	3	2	311	northwest wind	946	12.6	0	80
2021-08-04 11:00:00	26	52	1.2	1	304	northwest wind	946	12.6	0	70
2021-08-04 12:00:00	29.4	33	3.1	2	244	southwest wind	946	12.6	0	80
2021-08-04 13:00:00	31.6	27	2.4	2	306	northwest wind	946	12.6	0	70
2021-08-04 14:00:00	30.5	41	0.1	0	124	southeast wind	945	12.6	0	90
2021-08-04 15:00:00	33.7	24	1	1	26	northeast wind	944	12.6	0	100
2021-08-04 16:00:00	33.4	24	1.1	1	29	northeast wind	944	12.6	0	100
2021-08-04 17:00:00	35	20	3.2	2	141	southeast wind	944	12.6	0	100
2021-08-04 18:00:00	32.7	25	2.2	2	306	northwest wind	944	12.6	0	100
2021-08-04 19:00:00	30.5	31	0.7	1	326	northwest wind	944	12.6	0	100
2021-08-04 20:00:00	30.6	32	1.6	2	297	northwest wind	944	12.6	0	100
2021-08-04 21:00:00	29.2	37	2.5	2	231	southwest wind	944	12.6	0	80
2021-08-04 22:00:00	28.5	32	0.9	1	243	southwest wind	945	12.6	0	70
2021-08-04 23:00:00	27.2	36	1.9	2	206	southwest wind	945	12.6	0	70
2021-08-05 00:00:00	27.4	34	1.5	1	207	southwest wind	945	12.6	0	86
2021-08-05 01:00:00	26.3	37	1.9	2	229	southwest wind	945	12.6	0	86
2021-08-05 02:00:00	24	45	0.5	1	213	southwest wind	945	12.6	0	70
2021-08-05 03:00:00	22.8	48	0.9	1	215	southwest wind	945	12.6	0	60
2021-08-05 04:00:00	20.9	55	3.1	2	242	southwest wind	945	12.6	0	0
2021-08-05 05:00:00	19.9	57	3.1	2	223	southwest wind	945	12.6	0	0
2021-08-05 06:00:00	18.6	61	1.1	1	226	southwest wind	944	12.6	0	60
2021-08-05 07:00:00	18.8	58	2.9	2	211	southwest wind	944	12.6	0	80
2021-08-05 08:00:00	19.8	53	2	2	295	northwest wind	945	12.6	0	70
2021-08-05 09:00:00	21.3	54	2.5	2	235	southwest wind	945	12.6	0	0
2021-08-05 10:00:00	24.8	45	0.5	1	135	southeast wind	944	12.6	0	0
2021-08-05 11:00:00	27.1	41	0.6	1	205	southwest wind	944	12.6	0	0
2021-08-05 12:00:00	31.2	28	1.6	2	306	northwest wind	944	12.6	0	60
2021-08-05 13:00:00	32.9	28	3.2	2	39	northeast wind	944	12.6	0	60
2021-08-05 14:00:00	32.6	27	3.7	3	48	northeast wind	944	12.6	0	100
2021-08-05 15:00:00	31.6	29	0.4	1	35	northeast wind	944	12.6	0	60
2021-08-05 16:00:00	31.1	29	5.1	3	133	southeast wind	944	12.6	0	100
2021-08-05 17:00:00	31.5	28	4.7	3	120	southeast wind	943	12.6	0	0
2021-08-05 18:00:00	31.6	28	2.9	2	53	northeast wind	943	12.6	0	60
2021-08-05 19:00:00	32.1	24	1.4	1	41	northeast wind	943	12.6	0	70
2021-08-05 20:00:00	29.3	33	2	2	322	northwest wind	944	12.6	0	80

2021-08-05 21:00:00	29.3	33	2	2	322	northwest wind	944	12.6	0	80
2021-08-05 22:00:00	29.3	33	2	2	322	northwest wind	944	12.6	0	80
2021-08-05 23:00:00	23.5	66	1.6	2	231	southwest wind	946	12.6	0	80
2021-08-06 00:00:00	22.1	69	1	1	222	southwest wind	946	12.6	0	60
2021-08-06 01:00:00	22.4	54	0.7	1	114	southeast wind	947	12.6	0	0
2021-08-06 02:00:00	22.6	55	2.2	2	336	northwest wind	947	12.6	0	60
2021-08-06 03:00:00	20	70	2	2	328	northwest wind	946	12.6	0	0
2021-08-06 04:00:00	18.4	74	0.8	1	244	southwest wind	947	12.6	0	60
2021-08-06 05:00:00	18	74	3.1	2	243	southwest wind	947	12.6	0	60
2021-08-06 06:00:00	17.5	76	2.9	2	207	southwest wind	947	12.6	0	0
2021-08-06 07:00:00	16.7	78	1.8	2	237	southwest wind	947	12.6	0	0
2021-08-06 08:00:00	16.8	77	1.9	2	241	southwest wind	947	12.6	0	0
2021-08-06 09:00:00	19	69	0.9	1	210	southwest wind	947	12.6	0	0
2021-08-06 10:00:00	21.6	63	1.7	2	326	northwest wind	947	12.6	0	0
2021-08-06 11:00:00	25.7	43	0.9	1	317	northwest wind	947	12.6	0	0
2021-08-06 12:00:00	28.2	36	1.8	2	24	northeast wind	947	12.6	0	0
2021-08-06 13:00:00	28.7	35	2.8	2	36	northeast wind	946	12.6	0	0
2021-08-06 14:00:00	29.9	33	2.8	2	156	southeast wind	946	12.6	0	0
2021-08-06 15:00:00	30.5	28	3	2	119	southeast wind	945	12.6	0	0
2021-08-06 16:00:00	31.1	31	0.3	1	144	southeast wind	945	12.6	0	0
2021-08-06 17:00:00	31.1	32	2.4	2	129	southeast wind	944	12.6	0	0
2021-08-06 18:00:00	31	30	2.2	2	148	southeast wind	944	12.6	0	0
2021-08-06 19:00:00	31.1	29	1.8	2	24	northeast wind	944	12.6	0	0
2021-08-06 20:00:00	30.8	30	2.9	2	151	southeast wind	944	12.6	0	60
2021-08-06 21:00:00	29.5	33	2.9	2	157	southeast wind	944	12.6	0	70
2021-08-06 22:00:00	26.6	43	3.1	2	313	northwest wind	945	12.6	0	90
2021-08-06 23:00:00	24.7	48	0	0	317	northwest wind	946	12.6	0	0
2021-08-07 00:00:00	23.4	48	2.2	2	142	southeast wind	946	12.6	0	0
2021-08-07 01:00:00	23.8	42	0.1	0	211	southwest wind	946	12.6	0	0
2021-08-07 02:00:00	22.3	46	2.6	2	328	northwest wind	947	12.6	0	60
2021-08-07 03:00:00	20.3	56	0.7	1	299	northwest wind	947	12.6	0	0
2021-08-07 04:00:00	19.4	58	0.9	1	230	southwest wind	947	12.6	0	0
2021-08-07 05:00:00	18.9	61	0.7	1	315	northwest wind	947	12.6	0	0
2021-08-07 06:00:00	17.3	70	2.5	2	245	southwest wind	947	12.6	0	0
2021-08-07 07:00:00	16	78	3.1	2	234	southwest wind	948	12.6	0	0
2021-08-07 08:00:00	16.1	78	2.9	2	240	southwest wind	949	12.6	0	0
2021-08-07 09:00:00	18.5	67	0.1	0	228	southwest wind	949	12.6	0	0
2021-08-07 10:00:00	22.4	57	0.2	0	330	northwest wind	949	12.6	0	0

2021-08-07 11:00:00	25.9	38	1	1	143	southeast wind	949	12.6	0	0
2021-08-07 12:00:00	26.8	35	1.9	2	42	northeast wind	949	12.6	0	0
2021-08-07 13:00:00	28.2	35	2.3	2	138	southeast wind	948	12.6	0	0
2021-08-07 14:00:00	29.2	30	2.9	2	30	northeast wind	947	12.6	0	0
2021-08-07 15:00:00	29.9	29	2.5	2	62	northeast wind	947	12.6	0	70
2021-08-07 16:00:00	30.4	29	3.1	2	116	southeast wind	946	12.6	0	60
2021-08-07 17:00:00	30.8	32	1	1	126	southeast wind	946	12.6	0	90
2021-08-07 18:00:00	31.2	28	0.3	1	149	southeast wind	945	12.6	0	60
2021-08-07 19:00:00	31.2	28	0.6	1	123	southeast wind	946	12.6	0	90
2021-08-07 20:00:00	30.7	28	2.7	2	29	northeast wind	946	12.6	0	60
2021-08-07 21:00:00	29.5	33	1.5	1	118	southeast wind	946	12.6	0	0
2021-08-07 22:00:00	26.1	45	2.5	2	304	northwest wind	947	12.6	0	70
2021-08-07 23:00:00	24.5	50	1.9	2	295	northwest wind	947	12.6	0	100
2021-08-08 00:00:00	22.5	61	0.5	1	0	north wind	947	12.6	0	90
2021-08-08 01:00:00	21.6	62	0.4	1	305	northwest wind	948	12.6	0	90
2021-08-08 02:00:00	21.4	59	0	0	225	southwest wind	948	12.6	0	100
2021-08-08 03:00:00	20.2	63	2.9	2	210	southwest wind	948	12.6	0	90
2021-08-08 04:00:00	19.1	72	1.2	1	214	southwest wind	948	12.6	0	60
2021-08-08 05:00:00	18.5	73	0.5	1	240	southwest wind	948	12.6	0	0
2021-08-08 06:00:00	17.4	77	1.1	1	236	southwest wind	948	12.6	0	0
2021-08-08 07:00:00	16.9	78	3.1	2	225	southwest wind	948	12.6	0	0
2021-08-08 08:00:00	17.2	74	2	2	212	southwest wind	948	12.6	0	0
2021-08-08 09:00:00	19.4	65	2.5	2	320	northwest wind	948	12.6	0	0
2021-08-08 10:00:00	22.4	60	0	0	206	southwest wind	948	12.6	0	0
2021-08-08 11:00:00	25.9	44	3.1	2	240	southwest wind	948	12.6	0	0
2021-08-08 12:00:00	29.6	32	3	2	114	southeast wind	948	12.6	0	0
2021-08-08 13:00:00	30.4	28	2.1	2	55	northeast wind	947	12.6	0	60
2021-08-08 14:00:00	30.5	30	0.4	1	114	southeast wind	947	12.6	0	0
2021-08-08 15:00:00	31.5	26	2.5	2	36	northeast wind	946	12.6	0	0
2021-08-08 16:00:00	32.3	26	2	2	132	southeast wind	946	12.6	0	0
2021-08-08 17:00:00	32.3	25	0.5	1	65	northeast wind	945	12.6	0	0
2021-08-08 18:00:00	32.8	26	1	1	115	southeast wind	945	12.6	0	60
2021-08-08 19:00:00	31	31	2.1	2	297	northwest wind	946	12.6	0	70
2021-08-08 20:00:00	27.9	35	7.5	4	217	southwest wind	948	12.6	0	60
2021-08-08 21:00:00	23.3	50	10.9	6	300	northwest wind	949	12.6	0.4	100
2021-08-08 22:00:00	23	49	4.2	3	322	northwest wind	950	12.6	0.2	100
2021-08-08 23:00:00	22.3	50	4.3	3	211	southwest wind	950	12.6	0	100
2021-08-09 00:00:00	19.7	67	0	0	217	southwest wind	950	12.6	0	0

2021-08-09 01:00:00	20	67	1.8	2	226	southwest wind	949	12.6	0	0
2021-08-09 02:00:00	17.8	79	0.6	1	238	southwest wind	949	12.6	0	0
2021-08-09 03:00:00	17.4	79	0	0	208	southwest wind	949	12.6	0	0
2021-08-09 04:00:00	16.8	82	3.2	2	241	southwest wind	948	12.6	0	60
2021-08-09 05:00:00	16.3	81	1.1	1	232	southwest wind	947	12.6	0	0
2021-08-09 06:00:00	16.6	74	0.1	0	217	southwest wind	947	12.6	0	0
2021-08-09 07:00:00	16.2	73	1.1	1	219	southwest wind	947	12.6	0	0
2021-08-09 08:00:00	16.3	72	1.2	1	235	southwest wind	947	12.6	0	0
2021-08-09 09:00:00	18.1	68	2.7	2	221	southwest wind	946	12.6	0	0
2021-08-09 10:00:00	21.2	60	1.5	1	233	southwest wind	946	12.6	0	0
2021-08-09 11:00:00	24.9	45	0.7	1	320	northwest wind	945	12.6	0	60
2021-08-09 12:00:00	28.2	35	1.1	1	337	northwest wind	945	12.6	0	0
2021-08-09 13:00:00	30.2	27	0.8	1	140	southeast wind	944	12.6	0	60
2021-08-09 14:00:00	31.7	27	0.5	1	205	southwest wind	943	12.6	0	60
2021-08-09 15:00:00	31.3	32	1.9	2	44	northeast wind	943	12.6	0	0
2021-08-09 16:00:00	30.7	36	2.8	2	45	northeast wind	942	12.6	0	60
2021-08-09 17:00:00	31.4	29	2.1	2	64	northeast wind	942	12.6	0	60
2021-08-09 18:00:00	30.6	34	2.6	2	215	southwest wind	943	12.6	0	60
2021-08-09 19:00:00	29.1	36	4.6	3	235	southwest wind	944	12.6	0	90
2021-08-09 20:00:00	30.2	27	1.1	1	222	southwest wind	943	12.6	0	100
2021-08-09 21:00:00	26.9	43	3.5	3	223	southwest wind	945	12.6	0	100
2021-08-09 22:00:00	24.2	53	1.5	1	318	northwest wind	946	12.6	0.8	100
2021-08-09 23:00:00	22.7	61	2.3	2	205	southwest wind	947	12.6	0	100
2021-08-10 00:00:00	21.4	68	7.2	4	218	southwest wind	949	12.6	0	100
2021-08-10 01:00:00	18.5	88	0.6	1	117	southeast wind	948	12.6	1.4	100
2021-08-10 02:00:00	18.8	86	2.7	2	315	northwest wind	948	12.6	0	100
2021-08-10 03:00:00	18.7	88	2.4	2	61	northeast wind	948	12.6	0	100
2021-08-10 04:00:00	18.6	88	2.2	2	308	northwest wind	948	12.6	0	100
2021-08-10 05:00:00	18.2	86	1.9	2	0	north wind	948	12.6	0	100
2021-08-10 06:00:00	17.5	89	0.7	1	238	southwest wind	948	12.6	0	100
2021-08-10 07:00:00	17.4	84	1	1	222	southwest wind	948	12.6	0	100
2021-08-10 08:00:00	16.7	84	0.8	1	221	southwest wind	948	12.6	0	100
2021-08-10 09:00:00	18.3	77	2.3	2	216	southwest wind	948	12.6	0	80
2021-08-10 10:00:00	21.8	63	2.5	2	239	southwest wind	948	12.6	0	0
2021-08-10 11:00:00	24.1	57	1.1	1	210	southwest wind	948	12.6	0	90
2021-08-10 12:00:00	27.3	43	1	1	244	southwest wind	948	12.6	0	100
2021-08-10 13:00:00	28	41	0.6	1	52	northeast wind	947	12.6	0	70
2021-08-10 14:00:00	28.7	36	4.4	3	31	northeast wind	947	12.6	0	60

2021-08-10 15:00:00	29.1	35	1.4	1	34	northeast wind	946	12.6	0	0
2021-08-10 16:00:00	29.8	30	1.6	2	26	northeast wind	946	12.6	0	60
2021-08-10 17:00:00	30.1	34	1.8	2	150	southeast wind	945	12.6	0	60
2021-08-10 18:00:00	30.3	31	0	0	40	northeast wind	946	12.6	0	60
2021-08-10 19:00:00	30.6	29	4.2	3	27	northeast wind	946	12.6	0	70
2021-08-10 20:00:00	29.5	32	2.9	2	308	northwest wind	946	12.6	0	70
2021-08-10 21:00:00	26.2	41	3	2	232	southwest wind	947	12.6	0	80
2021-08-10 22:00:00	23.4	53	0.8	1	231	southwest wind	948	12.6	0	0
2021-08-10 23:00:00	22.4	54	0	0	232	southwest wind	948	12.6	0	0
2021-08-11 00:00:00	21	60	2	2	239	southwest wind	948	12.6	0	0
2021-08-11 01:00:00	19.7	68	1.7	2	326	northwest wind	949	12.6	0	0
2021-08-11 02:00:00	18.7	73	2.3	2	322	northwest wind	949	12.6	0	0
2021-08-11 03:00:00	17.6	78	1.7	2	204	southwest wind	949	12.6	0	0
2021-08-11 04:00:00	17	81	0.8	1	232	southwest wind	949	12.6	0	0
2021-08-11 05:00:00	16.2	81	0	0	220	southwest wind	949	12.6	0	0
2021-08-11 06:00:00	15.7	83	0.7	1	208	southwest wind	949	12.6	0	0
2021-08-11 07:00:00	15.2	85	2.3	2	241	southwest wind	949	12.6	0	0
2021-08-11 08:00:00	15.5	83	2.7	2	241	southwest wind	950	12.6	0	0
2021-08-11 09:00:00	17.9	72	0.5	1	212	southwest wind	949	12.6	0	0
2021-08-11 10:00:00	21.8	59	1.4	1	219	southwest wind	949	12.6	0	0
2021-08-11 11:00:00	25.4	46	3	2	232	southwest wind	949	12.6	0	0
2021-08-11 12:00:00	27.3	33	0.5	1	208	southwest wind	949	12.6	0	0
2021-08-11 13:00:00	29	32	0.2	0	143	southeast wind	948	12.6	0	0
2021-08-11 14:00:00	29.3	31	0.1	0	125	southeast wind	947	12.6	0	0
2021-08-11 15:00:00	30.1	28	0.7	1	26	northeast wind	947	12.6	0	60
2021-08-11 16:00:00	29.9	33	1.4	1	37	northeast wind	946	12.6	0	60
2021-08-11 17:00:00	29.8	34	1.5	1	42	northeast wind	946	12.6	0	0
2021-08-11 18:00:00	30.1	34	1.4	1	130	southeast wind	946	12.6	0	60
2021-08-11 19:00:00	30.3	30	0.5	1	127	southeast wind	946	12.6	0	60
2021-08-11 20:00:00	30.2	29	0.3	1	45	northeast wind	946	12.6	0	60
2021-08-11 21:00:00	28.1	35	1.5	1	326	northwest wind	946	12.6	0	0
2021-08-11 22:00:00	24.6	45	0.6	1	306	northwest wind	946	12.6	0	60
2021-08-11 23:00:00	23	54	0.4	1	246	southwest wind	947	12.6	0	0
2021-08-12 00:00:00	21.3	61	2.6	2	219	southwest wind	947	12.6	0	60
2021-08-12 01:00:00	21	61	1.3	1	241	southwest wind	947	12.6	0	0
2021-08-12 02:00:00	20.6	63	1.1	1	240	southwest wind	947	12.6	0	0
2021-08-12 03:00:00	20	66	0.3	1	221	southwest wind	947	12.6	0	0
2021-08-12 04:00:00	18.8	69	0.6	1	234	southwest wind	947	12.6	0	0

2021-08-12 05:00:00	18.4	69	0.9	1	223	southwest wind	947	12.6	0	0
2021-08-12 06:00:00	17.3	75	2.5	2	218	southwest wind	947	12.6	0	0
2021-08-12 07:00:00	17	75	0.7	1	214	southwest wind	947	12.6	0	0
2021-08-12 08:00:00	17	75	0	0	221	southwest wind	947	12.6	0	60
2021-08-12 09:00:00	19.2	67	0.6	1	240	southwest wind	947	12.6	0	0
2021-08-12 10:00:00	22.2	58	0.6	1	233	southwest wind	947	12.6	0	60
2021-08-12 11:00:00	26.4	46	0.8	1	231	southwest wind	947	12.6	0	0
2021-08-12 12:00:00	28.1	36	2.9	2	64	northeast wind	946	12.6	0	0
2021-08-12 13:00:00	29.3	36	4.5	3	39	northeast wind	946	12.6	0	0
2021-08-12 14:00:00	30.5	34	1.9	2	151	southeast wind	945	12.6	0	0
2021-08-12 15:00:00	30.9	32	0	0	36	northeast wind	945	12.6	0	60
2021-08-12 16:00:00	31.5	29	2.7	2	37	northeast wind	944	12.6	0	0
2021-08-12 17:00:00	31.7	29	1.4	1	121	southeast wind	944	12.6	0	0
2021-08-12 18:00:00	31.4	29	0	0	38	northeast wind	943	12.6	0	80
2021-08-12 19:00:00	31.2	32	0.8	1	129	southeast wind	943	12.6	0	86
2021-08-12 20:00:00	29.8	41	2.2	2	300	northwest wind	943	12.6	0	90
2021-08-12 21:00:00	28.4	41	2.2	2	135	southeast wind	944	12.6	0	100
2021-08-12 22:00:00	27.4	42	3	2	337	northwest wind	944	12.6	0	100
2021-08-12 23:00:00	25.8	46	1.1	1	229	southwest wind	944	12.6	0	100
2021-08-13 00:00:00	25	51	2.1	2	222	southwest wind	944	12.6	0	100
2021-08-13 01:00:00	23.8	58	0.4	1	205	southwest wind	944	12.6	0	100
2021-08-13 02:00:00	24.4	55	1.6	2	249	west wind	944	12.6	0	100
2021-08-13 03:00:00	22.9	60	1.5	1	226	southwest wind	944	12.6	0	100
2021-08-13 04:00:00	22.9	54	1.3	1	313	northwest wind	944	12.6	0	100
2021-08-13 05:00:00	22.9	52	0.8	1	220	southwest wind	944	12.6	0	0
2021-08-13 06:00:00	24.4	35	3.4	3	219	southwest wind	944	12.6	0	90
2021-08-13 07:00:00	20.5	59	7.9	4	229	southwest wind	946	12.6	0	100
2021-08-13 08:00:00	20.7	54	1.6	2	207	southwest wind	947	12.6	0	90
2021-08-13 09:00:00	18.4	78	0.9	1	302	northwest wind	946	12.6	0.1	100
2021-08-13 10:00:00	19.3	75	0.1	0	233	southwest wind	946	12.6	0.1	100
2021-08-13 11:00:00	22.6	61	2.1	2	123	southeast wind	946	12.6	0	100
2021-08-13 12:00:00	24.9	43	0.5	1	46	northeast wind	946	12.6	0	0
2021-08-13 13:00:00	26.9	41	0.9	1	156	southeast wind	945	12.6	0	60
2021-08-13 14:00:00	27.3	42	5.2	3	59	northeast wind	944	12.6	0	100
2021-08-13 15:00:00	27.5	42	0.7	1	123	southeast wind	944	12.6	0	80
2021-08-13 16:00:00	26.2	49	2.7	2	151	southeast wind	945	12.6	0	90
2021-08-13 17:00:00	28.8	32	2.8	2	306	northwest wind	943	12.6	0	70
2021-08-13 18:00:00	29.4	33	2	2	28	northeast wind	943	12.6	0	90

2021-08-13 19:00:00	29.5	35	1	1	28	northeast wind	943	12.6	0	100
2021-08-13 20:00:00	27.9	40	0.2	0	183	south wind	943	12.6	0	100
2021-08-13 21:00:00	26.7	41	1.9	2	204	southwest wind	944	12.6	0	100
2021-08-13 22:00:00	24.6	51	1.8	2	309	northwest wind	945	12.6	0	100
2021-08-13 23:00:00	22.5	54	0.5	1	208	southwest wind	945	12.6	0	80
2021-08-14 00:00:00	19.9	67	2.5	2	246	southwest wind	945	30	0	80
2021-08-14 01:00:00	20.5	56	1	1	238	southwest wind	946	30	0	100
2021-08-14 02:00:00	20.1	58	1.6	2	319	northwest wind	945	30	0	60
2021-08-14 03:00:00	18.3	66	2.3	2	218	southwest wind	945	30	0	60
2021-08-14 04:00:00	17.8	70	0.7	1	204	southwest wind	945	30	0	60
2021-08-14 05:00:00	16.9	74	0.7	1	246	southwest wind	945	30	0	0
2021-08-14 06:00:00	17.4	71	0.4	1	297	northwest wind	945	30	0	100
2021-08-14 07:00:00	17.5	70	1.9	2	330	northwest wind	945	30	0	100
2021-08-14 08:00:00	16.4	79	3.1	2	241	southwest wind	946	30	0	100
2021-08-14 09:00:00	17.3	78	0.3	1	121	southeast wind	946	30	0	100
2021-08-14 10:00:00	18.3	76	3.2	2	225	southwest wind	946	30	0	100
2021-08-14 11:00:00	19.5	79	2.9	2	54	northeast wind	946	30	0	100
2021-08-14 12:00:00	21.1	57	2.2	2	24	northeast wind	946	30	0	100
2021-08-14 13:00:00	22.3	54	2.1	2	121	southeast wind	946	30	0	100
2021-08-14 14:00:00	22.8	55	0.3	1	211	southwest wind	946	30	0	100
2021-08-14 15:00:00	22.9	53	3	2	203	southwest wind	946	30	0	100
2021-08-14 16:00:00	21.7	63	1	1	229	southwest wind	946	30	0.1	100
2021-08-14 17:00:00	18.5	86	1.2	1	326	northwest wind	947	9	0.9	100
2021-08-14 18:00:00	17.7	92	2.6	2	305	northwest wind	948	7.6	3.6	100
2021-08-14 19:00:00	18.2	88	0.1	0	325	northwest wind	948	10.8	1	100
2021-08-14 20:00:00	18.4	85	0.9	1	134	southeast wind	948	15	0	100
2021-08-14 21:00:00	18.3	86	4.2	3	124	southeast wind	949	21	0	100
2021-08-14 22:00:00	17.4	91	2.9	2	136	southeast wind	949	13.8	0	100
2021-08-14 23:00:00	17	92	2.7	2	36	northeast wind	950	10.5	0.2	100
2021-08-15 00:00:00	16.4	91	0.5	1	228	southwest wind	949	8.1	0.4	100
2021-08-15 01:00:00	15.8	93	1.5	1	205	southwest wind	949	25.4	0	60
2021-08-15 02:00:00	14.8	96	2.1	2	218	southwest wind	949	11.6	0	0
2021-08-15 03:00:00	14.9	98	2.6	2	212	southwest wind	948	7.4	0	0
2021-08-15 04:00:00	14.4	97	2.2	2	213	southwest wind	948	6.3	0	0
2021-08-15 05:00:00	14.2	98	1.7	2	222	southwest wind	948	6.3	0	0
2021-08-15 06:00:00	13.6	96	1.4	1	205	southwest wind	947	9.2	0.1	0
2021-08-15 07:00:00	13.1	95	1.8	2	230	southwest wind	947	15.7	0	0
2021-08-15 08:00:00	12.8	95	0.9	1	247	southwest wind	948	15.9	0	0

2021-08-15 09:00:00	14.3	91	0	0	211	southwest wind	948	19.7	0	0
2021-08-15 10:00:00	17.1	80	1.3	1	328	northwest wind	948	26.8	0	0
2021-08-15 11:00:00	20	68	1.4	1	27	northeast wind	948	20.3	0	0
2021-08-15 12:00:00	21.8	57	3	2	146	southeast wind	948	25.3	0	0
2021-08-15 13:00:00	23.6	48	0.6	1	311	northwest wind	948	30	0	60
2021-08-15 14:00:00	24.2	47	2.4	2	26	northeast wind	948	30	0	60
2021-08-15 15:00:00	24.6	45	0.3	1	140	southeast wind	948	30	0	60
2021-08-15 16:00:00	24.3	46	0.5	1	32	northeast wind	948	30	0	100
2021-08-15 17:00:00	24.2	46	3.1	2	114	southeast wind	948	30	0	100
2021-08-15 18:00:00	24	47	3.2	2	126	southeast wind	948	29.5	0	100
2021-08-15 19:00:00	24.8	44	2.3	2	43	northeast wind	948	29.8	0	100
2021-08-15 20:00:00	23.1	50	0.1	0	41	northeast wind	948	30	0	100
2021-08-15 21:00:00	22	63	0.6	1	204	southwest wind	949	27.2	0	90
2021-08-15 22:00:00	19.9	61	0.8	1	232	southwest wind	950	28.1	0	86
2021-08-15 23:00:00	18.5	71	2.2	2	233	southwest wind	951	29.3	0	90
2021-08-16 00:00:00	17.6	76	0.4	1	223	southwest wind	951	28.9	0	100
2021-08-16 01:00:00	17.5	78	1.8	2	221	southwest wind	951	28.9	0	90
2021-08-16 02:00:00	17.8	74	2.5	2	229	southwest wind	951	30	0	100
2021-08-16 03:00:00	17.7	73	0.8	1	209	southwest wind	951	30	0	100
2021-08-16 04:00:00	17.6	71	1.3	1	230	southwest wind	951	30	0	100
2021-08-16 05:00:00	18.6	63	0.1	0	275	west wind	951	30	0	86
2021-08-16 06:00:00	18.4	64	1.3	1	214	southwest wind	951	30	0	86
2021-08-16 07:00:00	16.6	90	0	0	207	southwest wind	952	12	1.1	90
2021-08-16 08:00:00	15.9	94	1.8	2	315	northwest wind	952	9.1	3.5	90
2021-08-16 09:00:00	15.9	95	0.6	1	337	northwest wind	952	9.6	0.7	100
2021-08-16 10:00:00	16.5	94	2.1	2	223	southwest wind	952	24.1	0.5	100
2021-08-16 11:00:00	17.3	91	1.9	2	221	southwest wind	952	19.9	0	100
2021-08-16 12:00:00	19	82	2.4	2	0	north wind	952	25.9	0	100
2021-08-16 13:00:00	20.7	70	0.8	1	56	northeast wind	952	30	0	100
2021-08-16 14:00:00	21.1	68	2.5	2	27	northeast wind	952	30	0	100
2021-08-16 15:00:00	21	70	1.1	1	36	northeast wind	952	30	0	100
2021-08-16 16:00:00	21.1	74	1.2	1	28	northeast wind	951	30	0	100
2021-08-16 17:00:00	20.7	67	0.2	0	122	southeast wind	951	30	0	100
2021-08-16 18:00:00	20.4	75	0	0	37	northeast wind	952	30	0	100
2021-08-16 19:00:00	20	78	0.1	0	328	northwest wind	952	30	0	100
2021-08-16 20:00:00	20	71	2.4	2	55	northeast wind	952	30	0	100
2021-08-16 21:00:00	19.2	82	1.4	1	240	southwest wind	953	30	0	100
2021-08-16 22:00:00	18.3	87	2	2	148	southeast wind	953	30	0	100

2021-08-16 23:00:00	18.8	79	2.6	2	241	southwest wind	954	30	0	100
2021-08-17 00:00:00	18.7	77	1.1	1	215	southwest wind	954	24.2	0	100
2021-08-17 01:00:00	18.5	74	2.5	2	213	southwest wind	954	30	0	100
2021-08-17 02:00:00	18.1	75	2.8	2	311	northwest wind	954	30	0	100
2021-08-17 03:00:00	17.5	80	2.2	2	212	southwest wind	954	30	0	100
2021-08-17 04:00:00	16.3	90	0.9	1	206	southwest wind	954	30	0.2	100
2021-08-17 05:00:00	15.9	94	0	0	219	southwest wind	954	17.8	0.4	100
2021-08-17 06:00:00	15.8	96	0.5	1	214	southwest wind	954	9.2	0.3	100
2021-08-17 07:00:00	15.8	96	1.1	1	231	southwest wind	954	11.9	0.4	100
2021-08-17 08:00:00	15.7	96	2.1	2	204	southwest wind	955	12.8	0.6	100
2021-08-17 09:00:00	15.5	96	0	0	219	southwest wind	954	17.1	0.6	100
2021-08-17 10:00:00	15.5	94	0.5	1	217	southwest wind	954	17.1	0.2	100
2021-08-17 11:00:00	16.1	93	2.3	2	219	southwest wind	954	17.1	0.3	100
2021-08-17 12:00:00	17.2	87	0.1	0	221	southwest wind	954	17.1	0	100
2021-08-17 13:00:00	18	83	2.4	2	220	southwest wind	954	17.1	0	100
2021-08-17 14:00:00	18.4	69	1.8	2	337	northwest wind	954	17.1	0	100
2021-08-17 15:00:00	18.6	70	1.2	1	325	northwest wind	954	17.1	0	100
2021-08-17 16:00:00	19.4	63	0.6	1	30	northeast wind	954	17.1	0.1	100
2021-08-17 17:00:00	18.9	66	1.7	2	223	southwest wind	954	17.1	0	100
2021-08-17 18:00:00	18.6	64	2.3	2	321	northwest wind	955	30	0	100
2021-08-17 19:00:00	18.7	70	0.1	0	48	northeast wind	955	30	0	100
2021-08-17 20:00:00	18.3	72	1	1	35	northeast wind	955	30	0	100
2021-08-17 21:00:00	18	68	1.9	2	141	southeast wind	955	30	0	100
2021-08-17 22:00:00	17.3	78	2.5	2	337	northwest wind	956	30	0	100
2021-08-17 23:00:00	15.9	90	0.6	1	245	southwest wind	956	30	0	100
2021-08-18 00:00:00	15.3	91	0.4	1	305	northwest wind	956	30	0	100
2021-08-18 01:00:00	14.8	92	2.9	2	330	northwest wind	955	30	0	100
2021-08-18 02:00:00	14.1	92	0.8	1	242	southwest wind	955	30	0	90
2021-08-18 03:00:00	13.1	95	2	2	218	southwest wind	954	30	0	60
2021-08-18 04:00:00	12.9	94	0.3	1	234	southwest wind	954	30	0	60
2021-08-18 05:00:00	12.9	94	2.4	2	207	southwest wind	953	30	0	60
2021-08-18 06:00:00	13.1	90	3.2	2	228	southwest wind	953	30	0	60
2021-08-18 07:00:00	12.7	91	0	0	239	southwest wind	953	30	0	0
2021-08-18 08:00:00	13.1	89	2.8	2	216	southwest wind	953	30	0	0
2021-08-18 09:00:00	15	82	3	2	230	southwest wind	953	30	0	0
2021-08-18 10:00:00	17.8	71	4.7	3	216	southwest wind	953	30	0	0
2021-08-18 11:00:00	20.6	63	1.8	2	317	northwest wind	953	30	0	0
2021-08-18 12:00:00	22.6	49	2.5	2	328	northwest wind	952	30	0	60

2021-08-18 13:00:00	24	41	1.4	1	40	northeast wind	952	30	0	0
2021-08-18 14:00:00	24.7	42	1.4	1	36	northeast wind	952	30	0	0
2021-08-18 15:00:00	25.4	41	0.7	1	215	southwest wind	951	30	0	0
2021-08-18 16:00:00	26.1	37	1.7	2	133	southeast wind	951	30	0	0
2021-08-18 17:00:00	26.4	38	1	1	118	southeast wind	950	30	0	0
2021-08-18 18:00:00	27.1	39	1.8	2	143	southeast wind	950	30	0	0
2021-08-18 19:00:00	27.1	36	0.7	1	133	southeast wind	950	30	0	0
2021-08-18 20:00:00	26.4	32	2.7	2	43	northeast wind	950	30	0	60
2021-08-18 21:00:00	24.2	48	3.2	2	126	southeast wind	950	30	0	0
2021-08-18 22:00:00	21	63	3	2	295	northwest wind	951	30	0	0
2021-08-18 23:00:00	19	75	0	0	327	northwest wind	951	27.1	0	0
2021-08-19 00:00:00	17.6	81	0	0	213	southwest wind	951	21.9	0	0
2021-08-19 01:00:00	16.8	81	1.2	1	208	southwest wind	951	27.7	0	0
2021-08-19 02:00:00	16.7	81	2.5	2	235	southwest wind	951	28	0	60
2021-08-19 03:00:00	15.9	85	2.3	2	214	southwest wind	950	30	0	0
2021-08-19 04:00:00	15.9	82	0.4	1	247	southwest wind	950	30	0	0
2021-08-19 05:00:00	15.3	86	3	2	207	southwest wind	950	30	0	0
2021-08-19 06:00:00	15.1	87	1.1	1	241	southwest wind	950	30	0	0
2021-08-19 07:00:00	14.8	87	1.8	2	223	southwest wind	950	30	0	0
2021-08-19 08:00:00	14.2	89	1.6	2	220	southwest wind	950	28.1	0	0
2021-08-19 09:00:00	16.2	82	3.2	2	235	southwest wind	951	26.3	0	0
2021-08-19 10:00:00	19	74	1.5	1	214	southwest wind	951	29.8	0	0
2021-08-19 11:00:00	23.1	55	2.4	2	140	southeast wind	951	30	0	0
2021-08-19 12:00:00	25.5	47	1.7	2	153	southeast wind	951	30	0	0
2021-08-19 13:00:00	26	45	1.9	2	37	northeast wind	950	30	0	0
2021-08-19 14:00:00	26.7	47	1.5	1	146	southeast wind	950	30	0	0
2021-08-19 15:00:00	26.9	42	0.5	1	27	northeast wind	949	30	0	4
2021-08-19 16:00:00	27.6	37	2.2	2	33	northeast wind	949	26.2	0	0
2021-08-19 17:00:00	27.7	36	0.3	1	52	northeast wind	948	29.1	0	0
2021-08-19 18:00:00	28.1	34	2	2	133	southeast wind	948	29.6	0	0
2021-08-19 19:00:00	27.3	41	1.1	1	133	southeast wind	948	30	0	53
2021-08-19 20:00:00	25.7	46	0.3	1	237	southwest wind	949	30	0	100
2021-08-19 21:00:00	23.4	57	3.2	2	230	southwest wind	949	26.9	0	90
2021-08-19 22:00:00	22.1	68	1.7	2	293	northwest wind	950	30	0	100
2021-08-19 23:00:00	21.5	67	0.7	1	228	southwest wind	950	30	0	100
2021-08-20 00:00:00	19.3	80	1.6	2	245	southwest wind	949	28.3	0	60
2021-08-20 01:00:00	18.9	81	2.6	2	227	southwest wind	949	28.1	0	9
2021-08-20 02:00:00	19	77	3.2	2	244	southwest wind	949	30	0	45

2021-08-20 03:00:00	19.2	71	2.9	2	321	northwest wind	950	30	0	26
2021-08-20 04:00:00	17.5	79	0.8	1	203	southwest wind	949	30	0	26
2021-08-20 05:00:00	17	80	2.2	2	243	southwest wind	950	30	0	
2021-08-20 06:00:00	15.9	85	0.3	1	218	southwest wind	950	30	0	
2021-08-20 07:00:00	15.8	83	0.2	0	219	southwest wind	950	30	0	
2021-08-20 08:00:00	15.9	80	0.1	0	308	northwest wind	951	30	0	
2021-08-20 09:00:00	17.5	74	1.8	2	219	southwest wind	951	30	0	0
2021-08-20 10:00:00	20.6	64	1.1	1	230	southwest wind	951	30	0	0
2021-08-20 11:00:00	24.1	54	2.5	2	234	southwest wind	951	30	0	0
2021-08-20 12:00:00	27.8	41	2.8	2	141	southeast wind	951	30	0	0
2021-08-20 13:00:00	28.9	35	1.8	2	125	southeast wind	950	30	0	0
2021-08-20 14:00:00	29.1	32	1.3	1	53	northeast wind	950	30	0	0
2021-08-20 15:00:00	30	32	2.1	2	156	southeast wind	950	30	0	0
2021-08-20 16:00:00	30.4	29	0.6	1	116	southeast wind	949	30	0	0
2021-08-20 17:00:00	30.6	28	1.9	2	139	southeast wind	949	30	0	0
2021-08-20 18:00:00	30.2	28	1.2	1	52	northeast wind	949	30	0	86
2021-08-20 19:00:00	29.8	27	1.4	1	137	southeast wind	949	30	0	60
2021-08-20 20:00:00	29.1	31	1.7	2	60	northeast wind	949	30	0	65
2021-08-20 21:00:00	26.6	40	2	2	154	southeast wind	950	30	0	45
2021-08-20 22:00:00	23.6	57	1.6	2	204	southwest wind	950	15.9	0	62
2021-08-20 23:00:00	21.9	61	2.5	2	223	southwest wind	951	30	0	55
2021-08-21 00:00:00	20.8	60	1.5	1	333	northwest wind	951	29.1	0	57
2021-08-21 01:00:00	20.9	60	1.8	2	336	northwest wind	952	29.8	0	43
2021-08-21 02:00:00	19.5	67	0.1	0	238	southwest wind	952	30	0	100
2021-08-21 03:00:00	18	78	0.5	1	212	southwest wind	952	29.9	0	100
2021-08-21 04:00:00	17.3	81	1.3	1	236	southwest wind	952	27.7	0	85
2021-08-21 05:00:00	16.9	83	0.5	1	289	west wind	952	29.5	0	65
2021-08-21 06:00:00	16.2	84	0.8	1	301	northwest wind	952	29	0	7
2021-08-21 07:00:00	15.4	88	2	2	204	southwest wind	952	28.7	0	0
2021-08-21 08:00:00	16.1	83	1	1	234	southwest wind	953	29.1	0	0
2021-08-21 09:00:00	17.8	77	0.9	1	329	northwest wind	953	27.1	0	0
2021-08-21 10:00:00	21.7	57	0.5	1	241	southwest wind	953	27.6	0	0
2021-08-21 11:00:00	24.2	43	2.2	2	122	southeast wind	953	30	0	2
2021-08-21 12:00:00	25.6	38	1.6	2	40	northeast wind	953	30	0	2
2021-08-21 13:00:00	26.2	37	2.8	2	122	southeast wind	952	29.6	0	0
2021-08-21 14:00:00	27.3	34	0.2	0	30	northeast wind	952	30	0	0
2021-08-21 15:00:00	27.9	30	2.2	2	316	northwest wind	951	30	0	0
2021-08-21 16:00:00	29.2	32	0.1	0	124	southeast wind	951	30	0	0

2021-08-21 17:00:00	29.3	30	2.2	2	325	northwest wind	950	30	0	0
2021-08-21 18:00:00	29.5	30	3	2	136	southeast wind	949	30	0	0
2021-08-21 19:00:00	29.5	29	2	2	143	southeast wind	949	30	0	28
2021-08-21 20:00:00	28.8	31	0.3	1	143	southeast wind	949	30	0	48
2021-08-21 21:00:00	25.4	50	1.4	1	25	northeast wind	950	30	0	0
2021-08-21 22:00:00	22.6	56	2.9	2	314	northwest wind	950	30	0	0
2021-08-21 23:00:00	22	46	3.2	2	312	northwest wind	951	23.9	0	0
2021-08-22 00:00:00	20.2	53	0.3	1	225	southwest wind	951	30	0	0
2021-08-22 01:00:00	19.3	57	0.2	0	214	southwest wind	951	30	0	0
2021-08-22 02:00:00	18.6	71	1.7	2	204	southwest wind	951	30	0	0
2021-08-22 03:00:00	17.4	77	2.7	2	236	southwest wind	950	30	0	0
2021-08-22 04:00:00	17.9	72	0.2	0	223	southwest wind	950	30	0	0
2021-08-22 05:00:00	16.8	77	3	2	213	southwest wind	950	30	0	0
2021-08-22 06:00:00	16.1	83	3	2	219	southwest wind	950	29.9	0	0
2021-08-22 07:00:00	15.7	85	1.9	2	206	southwest wind	950	29.8	0	0
2021-08-22 08:00:00	16.4	79	1.4	1	226	southwest wind	951	29.8	0	0
2021-08-22 09:00:00	17.7	73	0.1	0	214	southwest wind	951	23.3	0	0
2021-08-22 10:00:00	20.9	65	0	0	227	southwest wind	951	29.5	0	0
2021-08-22 11:00:00	24.6	45	1.9	2	124	southeast wind	952	30	0	0
2021-08-22 12:00:00	26.1	32	1.8	2	125	southeast wind	952	30	0	0
2021-08-22 13:00:00	26.7	35	1.4	1	133	southeast wind	952	30	0	0
2021-08-22 14:00:00	27.5	33	1.7	2	139	southeast wind	951	17	0	0
2021-08-22 15:00:00	28.3	35	2.2	2	33	northeast wind	951	30	0	0
2021-08-22 16:00:00	29.5	29	1.8	2	29	northeast wind	951	30	0	0
2021-08-22 17:00:00	30.1	27	1	1	34	northeast wind	950	30	0	0
2021-08-22 18:00:00	30	27	2.5	2	91	east wind	950	30	0	0
2021-08-22 19:00:00	29.6	25	2.5	2	25	northeast wind	950	30	0	0
2021-08-22 20:00:00	28.9	31	0.3	1	147	southeast wind	950	30	0	0
2021-08-22 21:00:00	25.9	44	1.8	2	42	northeast wind	951	30	0	0
2021-08-22 22:00:00	22.9	52	0.2	0	312	northwest wind	952	30	0	0
2021-08-22 23:00:00	20.7	66	2.5	2	300	northwest wind	952	30	0	0
2021-08-23 00:00:00	22.3	45	0.3	1	48	northeast wind	953	30	0	0
2021-08-23 01:00:00	19.3	63	0.4	1	246	southwest wind	953	30	0	2
2021-08-23 02:00:00	17.7	70	1.5	1	217	southwest wind	953	30	0	19
2021-08-23 03:00:00	16.4	77	1.2	1	226	southwest wind	953	30	0	0
2021-08-23 04:00:00	16.1	74	0.8	1	218	southwest wind	953	30	0	0
2021-08-23 05:00:00	15.2	78	0.8	1	243	southwest wind	953	30	0	0
2021-08-23 06:00:00	15.1	74	1.8	2	218	southwest wind	953	30	0	0

2021-08-23 07:00:00	14.6	75	1.4	1	209	southwest wind	953	30	0	0
2021-08-23 08:00:00	14.3	76	3.2	2	223	southwest wind	953	30	0	0
2021-08-23 09:00:00	16.3	69	2	2	208	southwest wind	954	30	0	0
2021-08-23 10:00:00	19.3	59	0.3	1	240	southwest wind	954	10.6	0	0
2021-08-23 11:00:00	23.8	35	0	0	150	southeast wind	954	7.9	0	0
2021-08-23 12:00:00	25.4	28	0.5	1	122	southeast wind	953	30	0	0
2021-08-23 13:00:00	25.8	27	0.9	1	220	southwest wind	953	30	0	0
2021-08-23 14:00:00	27	26	2.5	2	320	northwest wind	952	30	0	0
2021-08-23 15:00:00	28.2	23	0.5	1	44	northeast wind	952	30	0	0
2021-08-23 16:00:00	28.5	23	4.2	3	51	northeast wind	951	30	0	0
2021-08-23 17:00:00	28.9	24	2.6	2	130	southeast wind	950	30	0	0
2021-08-23 18:00:00	28.7	26	2.5	2	152	southeast wind	950	30	0	0
2021-08-23 19:00:00	28.5	28	0.3	1	132	southeast wind	950	30	0	0
2021-08-23 20:00:00	27.9	28	0.2	0	122	southeast wind	950	30	0	0
2021-08-23 21:00:00	24.9	41	0.4	1	331	northwest wind	950	30	0	0
2021-08-23 22:00:00	22	54	0.3	1	313	northwest wind	950	30	0	0
2021-08-23 23:00:00	20.2	60	1.5	1	237	southwest wind	951	30	0	0
2021-08-24 00:00:00	18.8	63	2.5	2	220	southwest wind	951	30	0	0
2021-08-24 01:00:00	17.7	69	3.1	2	206	southwest wind	951	27.8	0	0
2021-08-24 02:00:00	17.2	69	3	2	206	southwest wind	951	27.8	0	12
2021-08-24 03:00:00	16.3	71	1.2	1	222	southwest wind	950	30	0	0
2021-08-24 04:00:00	15.8	74	0.6	1	217	southwest wind	950	30	0	0
2021-08-24 05:00:00	15.5	74	0.9	1	209	southwest wind	950	30	0	0
2021-08-24 06:00:00	15.2	72	2.4	2	227	southwest wind	950	30	0	0
2021-08-24 07:00:00	15	72	2	2	214	southwest wind	949	30	0	0
2021-08-24 08:00:00	15.1	72	2	2	233	southwest wind	950	30	0	0
2021-08-24 09:00:00	17.1	64	2.4	2	210	southwest wind	950	30	0	0
2021-08-24 10:00:00	20.2	60	2.7	2	243	southwest wind	950	21.3	0	0
2021-08-24 11:00:00	24.9	44	2.8	2	114	southeast wind	950	30	0	5
2021-08-24 12:00:00	27.1	30	0.9	1	52	northeast wind	951	30	0	0
2021-08-24 13:00:00	28.2	30	2	2	42	northeast wind	950	30	0	0
2021-08-24 14:00:00	28.3	30	0.5	1	64	northeast wind	950	30	0	9
2021-08-24 15:00:00	29	29	1	1	123	southeast wind	950	30	0	0
2021-08-24 16:00:00	29.7	29	2.8	2	133	southeast wind	949	30	0	26
2021-08-24 17:00:00	30.3	29	1.8	2	37	northeast wind	949	30	0	36
2021-08-24 18:00:00	29.7	30	1.9	2	114	southeast wind	949	30	0	4
2021-08-24 19:00:00	29.4	29	0.3	1	28	northeast wind	949	30	0	86
2021-08-24 20:00:00	29.3	28	2	2	125	southeast wind	949	30	0	60

2021-08-24 21:00:00	25.6	41	1.4	1	294	northwest wind	950	30	0	12
2021-08-24 22:00:00	23.6	44	2.4	2	334	northwest wind	950	30	0	0
2021-08-24 23:00:00	21.1	56	2.9	2	213	southwest wind	951	30	0	4
2021-08-25 00:00:00	19.6	63	0.5	1	228	southwest wind	951	30	0	24
2021-08-25 01:00:00	19.1	65	2.9	2	240	southwest wind	951	30	0	63
2021-08-25 02:00:00	18.1	65	0.8	1	245	southwest wind	951	30	0	55
2021-08-25 03:00:00	17.3	72	0.8	1	223	southwest wind	951	30	0	20
2021-08-25 04:00:00	17	72	0	0	223	southwest wind	951	30	0	71
2021-08-25 05:00:00	16.8	71	1.4	1	237	southwest wind	951	30	0	100
2021-08-25 06:00:00	17	68	2.6	2	241	southwest wind	951	30	0	100
2021-08-25 07:00:00	17.1	63	3	2	239	southwest wind	951	30	0	100
2021-08-25 08:00:00	16.9	66	1.7	2	215	southwest wind	951	30	0	82
2021-08-25 09:00:00	18.2	58	2	2	307	northwest wind	951	30	0	94
2021-08-25 10:00:00	19.7	60	0.7	1	240	southwest wind	951	30	0	100
2021-08-25 11:00:00	24	41	1.2	1	62	northeast wind	952	30	0	70
2021-08-25 12:00:00	25.4	37	2.1	2	23	northeast wind	952	30	0	90
2021-08-25 13:00:00	27.2	31	1.7	2	43	northeast wind	951	30	0	48
2021-08-25 14:00:00	28.6	23	1.1	1	123	southeast wind	950	30	0	60
2021-08-25 15:00:00	29.1	20	3.2	2	73	east wind	950	30	0	63
2021-08-25 16:00:00	29.3	24	2	2	132	southeast wind	950	30	0	79
2021-08-25 17:00:00	29.3	27	2.1	2	157	southeast wind	949	30	0	90
2021-08-25 18:00:00	29.6	22	0.4	1	116	southeast wind	949	30	0	100
2021-08-25 19:00:00	30	22	2	2	50	northeast wind	949	30	0	100
2021-08-25 20:00:00	27.9	29	1.5	1	326	northwest wind	949	30	0	62
2021-08-25 21:00:00	26.4	37	1.7	2	216	southwest wind	949	30	0	90
2021-08-25 22:00:00	24.8	40	0.9	1	240	southwest wind	949	30	0	60
2021-08-25 23:00:00	24.5	36	4.5	3	217	southwest wind	949	30	0	66
2021-08-26 00:00:00	24.1	37	2.4	2	222	southwest wind	949	30	0	100
2021-08-26 01:00:00	24.8	34	0.8	1	211	southwest wind	949	30	0	60
2021-08-26 02:00:00	23.1	39	1.9	2	227	southwest wind	949	30	0	60
2021-08-26 03:00:00	20.5	50	0.3	1	219	southwest wind	948	30	0	55
2021-08-26 04:00:00	18.5	59	2.8	2	226	southwest wind	948	30	0	60
2021-08-26 05:00:00	19.1	54	1	1	233	southwest wind	948	30	0	33
2021-08-26 06:00:00	18.5	58	0.3	1	221	southwest wind	947	30	0	61
2021-08-26 07:00:00	17.8	59	0.8	1	219	southwest wind	947	30	0	63
2021-08-26 08:00:00	18.4	57	2.9	2	234	southwest wind	947	30	0	63
2021-08-26 09:00:00	19.7	51	0.3	1	219	southwest wind	948	30	0	48
2021-08-26 10:00:00	20.3	51	1	1	241	southwest wind	947	30	0	56

2021-08-26 11:00:00	24.1	47	1	1	215	southwest wind	947	30	0	52
2021-08-26 12:00:00	28.4	41	1.1	1	225	southwest wind	947	30	0	2
2021-08-26 13:00:00	30.8	27	1.9	2	118	southeast wind	946	30	0	2
2021-08-26 14:00:00	32.5	17	0.4	1	236	southwest wind	946	30	0	0
2021-08-26 15:00:00	32.8	20	3.1	2	24	northeast wind	945	30	0	0
2021-08-26 16:00:00	32.4	21	2.5	2	144	southeast wind	945	30	0	0
2021-08-26 17:00:00	31.5	20	1.6	2	23	northeast wind	945	29.3	0	33
2021-08-26 18:00:00	32.2	22	3.5	3	55	northeast wind	945	29.3	0	63
2021-08-26 19:00:00	30.4	23	1.6	2	35	northeast wind	945	29.3	0	69
2021-08-26 20:00:00	27.6	29	3.6	3	243	southwest wind	947	29.3	0	86
2021-08-26 21:00:00	25.3	36	1.5	1	218	southwest wind	947	29.3	0	100
2021-08-26 22:00:00	25.1	34	3.7	3	43	northeast wind	947	29.3	0	90
2021-08-26 23:00:00	23	44	1.1	1	209	southwest wind	947	29.3	0	100
2021-08-27 00:00:00	21.8	50	1.2	1	209	southwest wind	947	30	0	75
2021-08-27 01:00:00	19.7	58	5.1	3	203	southwest wind	947	30	0	48
2021-08-27 02:00:00	19.3	58	2	2	203	southwest wind	946	30	0	0
2021-08-27 03:00:00	19.3	54	2.2	2	221	southwest wind	946	30	0	33
2021-08-27 04:00:00	18.7	55	1	1	222	southwest wind	945	30	0	60
2021-08-27 05:00:00	17.7	60	2.1	2	223	southwest wind	945	30	0	57
2021-08-27 06:00:00	17.5	58	2.9	2	225	southwest wind	945	30	0	50
2021-08-27 07:00:00	16.9	59	2.4	2	213	southwest wind	945	30	0	60
2021-08-27 08:00:00	16.5	57	2.5	2	220	southwest wind	945	30	0	33
2021-08-27 09:00:00	18.1	54	0.3	1	207	southwest wind	945	30	0	0
2021-08-27 10:00:00	21.3	47	2.3	2	246	southwest wind	945	30	0	0
2021-08-27 11:00:00	25	46	2.3	2	245	southwest wind	945	30	0	0
2021-08-27 12:00:00	29.9	23	2.9	2	305	northwest wind	945	30	0	0
2021-08-27 13:00:00	32	19	0.2	0	297	northwest wind	945	30	0	0
2021-08-27 14:00:00	31.9	18	2	2	32	northeast wind	944	30	0	0
2021-08-27 15:00:00	31.9	18	2	2	32	northeast wind	944	30	0	0
2021-08-27 16:00:00	33.1	18	2.2	2	36	northeast wind	944	30	0	0
2021-08-27 17:00:00	33.3	16	2.4	2	118	southeast wind	944	30	0	0
2021-08-27 18:00:00	33	15	0.6	1	33	northeast wind	944	30	0	0
2021-08-27 19:00:00	32.8	16	0.8	1	126	southeast wind	944	30	0	0
2021-08-27 20:00:00	30.6	22	2.5	2	33	northeast wind	944	30	0	0
2021-08-27 21:00:00	25.5	41	2.2	2	297	northwest wind	944	30	0	0
2021-08-27 22:00:00	23	44	1.7	2	242	southwest wind	945	24.1	0	50
2021-08-27 23:00:00	20.7	57	2.7	2	258	west wind	945	16.4	0	0
2021-08-28 00:00:00	20.3	53	2.7	2	234	southwest wind	946	27.6	0	31

2021-08-28 01:00:00	20.6	49	1	1	214	southwest wind	946	30	0	64
2021-08-28 02:00:00	20.5	48	2.7	2	208	southwest wind	946	30	0	65
2021-08-28 03:00:00	20.2	49	3.1	2	220	southwest wind	946	30	0	66
2021-08-28 04:00:00	20.7	46	0.6	1	332	northwest wind	946	30	0	67
2021-08-28 05:00:00	19.4	54	2.9	2	236	southwest wind	946	30	0	100
2021-08-28 06:00:00	19.1	53	3.2	2	209	southwest wind	946	30	0	100
2021-08-28 07:00:00	18	55	1.2	1	211	southwest wind	946	30	0	90
2021-08-28 08:00:00	17.9	54	2.7	2	266	west wind	946	30	0	90
2021-08-28 09:00:00	18.7	52	1.8	2	207	southwest wind	946	30	0	64
2021-08-28 10:00:00	21.1	51	2.7	2	239	southwest wind	946	30	0	100
2021-08-28 11:00:00	22.7	49	2.3	2	225	southwest wind	946	29.6	0	55
2021-08-28 12:00:00	22.9	54	0.9	1	225	southwest wind	947	29.6	0	90
2021-08-28 13:00:00	25	46	0.8	1	229	southwest wind	947	29.6	0	90
2021-08-28 14:00:00	27	40	1	1	224	southwest wind	946	29.6	0	100
2021-08-28 15:00:00	27.1	35	4.3	3	225	southwest wind	947	29.6	0	100
2021-08-28 16:00:00	24.8	42	5.2	3	231	southwest wind	948	29.6	0	100
2021-08-28 17:00:00	24.6	47	1	1	205	southwest wind	948	29.6	0	100
2021-08-28 18:00:00	25.5	42	4.7	3	234	southwest wind	948	29.6	0	64
2021-08-28 19:00:00	25.9	39	4.3	3	246	southwest wind	947	29.6	0	100
2021-08-28 20:00:00	24.3	41	2.8	2	205	southwest wind	948	29.6	0	60
2021-08-28 21:00:00	23.7	42	2.3	2	205	southwest wind	948	30	0	90
2021-08-28 22:00:00	23	44	2.5	2	231	southwest wind	948	30	0	100
2021-08-28 23:00:00	22.3	47	0.5	1	208	southwest wind	949	30	0	100
2021-08-29 00:00:00	21.1	54	3.2	2	229	southwest wind	949	26.2	0	100
2021-08-29 01:00:00	19.9	57	1.3	1	220	southwest wind	949	26.2	0	100
2021-08-29 02:00:00	18.7	62	1.1	1	222	southwest wind	948	30	0	86
2021-08-29 03:00:00	16.9	70	2.4	2	209	southwest wind	948	30	0	58
2021-08-29 04:00:00	16.1	72	1.5	1	225	southwest wind	948	30	0	2
2021-08-29 05:00:00	15.4	73	1.8	2	247	southwest wind	948	30	0	2
2021-08-29 06:00:00	15	72	0.5	1	226	southwest wind	947	30	0	0
2021-08-29 07:00:00	14.8	71	2.6	2	209	southwest wind	947	30	0	0
2021-08-29 08:00:00	14.3	72	2.3	2	230	southwest wind	948	30	0	0
2021-08-29 09:00:00	16.1	66	2.4	2	323	northwest wind	948	30	0	0
2021-08-29 10:00:00	19.6	55	2.9	2	203	southwest wind	948	30	0	0
2021-08-29 11:00:00	22.5	53	2	2	221	southwest wind	948	29.7	0	9
2021-08-29 12:00:00	26.2	35	3.1	2	302	northwest wind	947	30	0	2
2021-08-29 13:00:00	28	31	2.7	2	30	northeast wind	947	30	0	55
2021-08-29 14:00:00	28.9	30	0.3	1	129	southeast wind	947	30	0	29

2021-08-29 15:00:00	29.3	31	0.9	1	66	northeast wind	946	30	0	75
2021-08-29 16:00:00	29.6	27	1.7	2	41	northeast wind	945	30	0	30
2021-08-29 17:00:00	28	34	1.3	1	133	southeast wind	946	30	0	100
2021-08-29 18:00:00	27.4	38	3.2	2	129	southeast wind	946	30	0	100
2021-08-29 19:00:00	26.5	42	1.3	1	129	southeast wind	947	30	0	100
2021-08-29 20:00:00	24.8	42	3.5	3	206	southwest wind	948	30	0	100
2021-08-29 21:00:00	22.1	49	7.4	4	205	southwest wind	949	30	0	100
2021-08-29 22:00:00	20.3	60	5.4	3	247	southwest wind	949	30	0	0
2021-08-29 23:00:00	20.2	59	3.9	3	203	southwest wind	949	30	0	55
2021-08-30 00:00:00	19	66	1.7	2	222	southwest wind	949	30	0	16
2021-08-30 01:00:00	19.9	57	4.7	3	203	southwest wind	948	30	0	0
2021-08-30 02:00:00	19.9	56	0.8	1	223	southwest wind	948	30	0	0
2021-08-30 03:00:00	17.3	69	1.9	2	229	southwest wind	948	30	0	0
2021-08-30 04:00:00	18.3	62	2.7	2	245	southwest wind	947	30	0	0
2021-08-30 05:00:00	17.5	64	4.3	3	224	southwest wind	947	30	0	0
2021-08-30 06:00:00	15.4	73	1.9	2	239	southwest wind	947	30	0	55
2021-08-30 07:00:00	14.9	73	2.7	2	212	southwest wind	947	30	0	2
2021-08-30 08:00:00	14.4	75	1.9	2	227	southwest wind	948	30	0	0
2021-08-30 09:00:00	16.2	67	5.1	3	208	southwest wind	948	30	0	0
2021-08-30 10:00:00	19.6	59	2.6	2	246	southwest wind	948	30	0	0
2021-08-30 11:00:00	23.6	47	1.3	1	323	northwest wind	948	30	0	0
2021-08-30 12:00:00	27.6	31	1.8	2	318	northwest wind	948	30	0	0
2021-08-30 13:00:00	28.6	35	0.2	0	116	southeast wind	947	30	0	0
2021-08-30 14:00:00	28.5	32	1.8	2	67	northeast wind	947	30	0	0
2021-08-30 15:00:00	29.2	30	0.7	1	41	northeast wind	947	30	0	0
2021-08-30 16:00:00	29.9	28	2.4	2	28	northeast wind	947	30	0	0
2021-08-30 17:00:00	30.8	28	1.1	1	115	southeast wind	946	30	0	26
2021-08-30 18:00:00	27.1	32	6.9	4	229	southwest wind	947	30	0	69
2021-08-30 19:00:00	29.4	29	3.2	2	311	northwest wind	947	30	0	90
2021-08-30 20:00:00	27.8	33	2.7	2	233	southwest wind	947	30	0	90
2021-08-30 21:00:00	26	34	0.1	0	247	southwest wind	948	30	0	75
2021-08-30 22:00:00	24.2	41	2.4	2	226	southwest wind	948	30	0	100
2021-08-30 23:00:00	23.1	45	3.1	2	223	southwest wind	948	30	0	31
2021-08-31 00:00:00	21.3	50	0.4	1	240	southwest wind	948	30	0	77
2021-08-31 01:00:00	20.2	53	0.1	0	236	southwest wind	948	30	0	60
2021-08-31 02:00:00	18.3	62	0	0	207	southwest wind	948	30	0	16
2021-08-31 03:00:00	17.7	63	2	2	215	southwest wind	947	30	0	0
2021-08-31 04:00:00	17.8	60	5.2	3	213	southwest wind	947	30	0	60

2021-08-31 05:00:00	16.8	63	1.4	1	217	southwest wind	947	30	0	72
2021-08-31 06:00:00	16.8	62	2.8	2	232	southwest wind	947	30	0	45
2021-08-31 07:00:00	17	60	3.2	2	220	southwest wind	947	30	0	28
2021-08-31 08:00:00	16.8	61	3.1	2	220	southwest wind	948	30	0	62
2021-08-31 09:00:00	17.8	58	0.7	1	204	southwest wind	948	30	0	36
2021-08-31 10:00:00	20.2	54	1.3	1	241	southwest wind	948	30	0	28
2021-08-31 11:00:00	23.3	47	3.2	2	117	southeast wind	948	30	0	0
2021-08-31 12:00:00	27.4	32	1.6	2	113	southeast wind	948	30	0	0
2021-08-31 13:00:00	28.5	30	0.7	1	25	northeast wind	948	30	0	0
2021-08-31 14:00:00	29	30	2.4	2	39	northeast wind	947	30	0	0
2021-08-31 15:00:00	29.8	31	1.9	2	121	southeast wind	947	30	0	0
2021-08-31 16:00:00	30.2	30	0.6	1	27	northeast wind	946	30	0	0
2021-08-31 17:00:00	29.6	32	0.3	1	141	southeast wind	946	30	0	0
2021-08-31 18:00:00	28.9	40	1.2	1	229	southwest wind	946	30	0	55
2021-08-31 19:00:00	28.5	40	0.5	1	324	northwest wind	946	30	0	50
2021-08-31 20:00:00	27.6	51	2.2	2	240	southwest wind	946	30	0	70
2021-08-31 21:00:00	25.1	53	2.3	2	232	southwest wind	947	30	0	43
2021-08-31 22:00:00	25.7	36	0.3	1	219	southwest wind	948	30	0	100
2021-08-31 23:00:00	24.7	38	0.1	0	247	southwest wind	948	30	0	43
2021-09-01 00:00:00	23	43	1	1	238	southwest wind	948	30	0	55
2021-09-01 01:00:00	21.7	44	2.1	2	235	southwest wind	948	30	0	60
2021-09-01 02:00:00	19.7	53	1.4	1	243	southwest wind	948	30	0	63
2021-09-01 03:00:00	18.5	60	3.1	2	223	southwest wind	948	30	0	100
2021-09-01 04:00:00	19	58	0.2	0	210	southwest wind	948	30	0	90
2021-09-01 05:00:00	19.3	58	0.8	1	214	southwest wind	948	30	0	100
2021-09-01 06:00:00	19.3	59	2.2	2	219	southwest wind	948	30	0	90
2021-09-01 07:00:00	18.8	63	2.1	2	222	southwest wind	949	30	0	86
2021-09-01 08:00:00	19.3	58	2.7	2	306	northwest wind	949	30	0	100
2021-09-01 09:00:00	20	57	2	2	294	northwest wind	950	30	0	100
2021-09-01 10:00:00	20.7	60	0.2	0	133	southeast wind	951	20.4	0	100
2021-09-01 11:00:00	24.3	43	2.4	2	213	southwest wind	951	30	0	54
2021-09-01 12:00:00	27	35	3	2	45	northeast wind	951	30	0	38
2021-09-01 13:00:00	28.2	33	1.8	2	31	northeast wind	950	30	0	57
2021-09-01 14:00:00	28	36	1.8	2	29	northeast wind	950	30	0	36
2021-09-01 15:00:00	28.3	37	4.8	3	31	northeast wind	949	30	0	100
2021-09-01 16:00:00	27.1	38	3.8	3	37	northeast wind	949	30	0	100
2021-09-01 17:00:00	26.5	39	1.1	1	210	southwest wind	951	30	0	100
2021-09-01 18:00:00	17.6	79	3.5	3	312	northwest wind	952	21.5	0.4	100

2021-09-01 19:00:00	21.1	64	1.8	2	226	southwest wind	952	30	0.4	100
2021-09-01 20:00:00	20.1	69	0.7	1	232	southwest wind	952	30	0	100
2021-09-01 21:00:00	19.7	74	1.9	2	332	northwest wind	953	30	0	100
2021-09-01 22:00:00	19.4	74	1	1	311	northwest wind	954	30	0	100
2021-09-01 23:00:00	18.8	85	2.2	2	240	southwest wind	954	15	0	100
2021-09-02 00:00:00	17.7	85	0.7	1	319	northwest wind	954	17	0.4	86
2021-09-02 01:00:00	17.2	90	1.1	1	243	southwest wind	954	23.9	0	86
2021-09-02 02:00:00	16.4	88	2.3	2	310	northwest wind	953	30	0	43
2021-09-02 03:00:00	15.6	91	1	1	334	northwest wind	953	30	0.1	33
2021-09-02 04:00:00	15.2	93	2.4	2	243	southwest wind	952	29.7	0	21
2021-09-02 05:00:00	14.8	93	1.2	1	206	southwest wind	952	30	0	12
2021-09-02 06:00:00	14.3	93	1.7	2	250	west wind	952	30	0	12
2021-09-02 07:00:00	13.7	93	1	1	217	southwest wind	952	30	0	0
2021-09-02 08:00:00	13.4	92	0.6	1	218	southwest wind	952	30	0	0
2021-09-02 09:00:00	14.8	86	3	2	212	southwest wind	952	28.9	0	0
2021-09-02 10:00:00	17.8	77	2.4	2	239	southwest wind	952	30	0	0
2021-09-02 11:00:00	21	68	1.4	1	217	southwest wind	952	30	0	0
2021-09-02 12:00:00	24.1	53	1.8	2	301	northwest wind	952	30	0	0
2021-09-02 13:00:00	25.7	42	2.9	2	43	northeast wind	951	30	0	0
2021-09-02 14:00:00	26.5	42	1.2	1	327	northwest wind	950	30	0	0
2021-09-02 15:00:00	27	41	1.3	1	126	southeast wind	950	30	0	0
2021-09-02 16:00:00	27	39	3.1	2	34	northeast wind	950	30	0	0
2021-09-02 17:00:00	27.4	40	2.7	2	150	southeast wind	949	30	0	0
2021-09-02 18:00:00	27.2	38	0.3	1	138	southeast wind	949	30	0	0
2021-09-02 19:00:00	26.9	41	2.3	2	34	northeast wind	949	30	0	0
2021-09-02 20:00:00	25.2	47	2	2	130	southeast wind	950	30	0	0
2021-09-02 21:00:00	23.9	54	2.7	2	204	southwest wind	951	30	0	79
2021-09-02 22:00:00	18.3	78	1.1	1	293	northwest wind	953	9.7	2.9	100
2021-09-02 23:00:00	17.9	80	2.8	2	239	southwest wind	954	9.7	0	84
2021-09-03 00:00:00	16.7	88	1	1	324	northwest wind	953	30	0.1	59
2021-09-03 01:00:00	16.8	86	0	0	137	southeast wind	953	30	0	0
2021-09-03 02:00:00	16.8	82	0.9	1	221	southwest wind	953	30	0	40
2021-09-03 03:00:00	15.3	89	3	2	216	southwest wind	952	30	0	0
2021-09-03 04:00:00	15	86	1.5	1	232	southwest wind	951	30	0	60
2021-09-03 05:00:00	14.4	87	0	0	217	southwest wind	950	30	0	86
2021-09-03 06:00:00	14.9	84	2.3	2	219	southwest wind	950	30	0	71
2021-09-03 07:00:00	14.7	81	1.2	1	234	southwest wind	950	30	0	73
2021-09-03 08:00:00	14.2	85	2.1	2	49	northeast wind	950	30	0	16

2021-09-03 09:00:00	16.1	78	3.1	2	312	northwest wind	951	30	0	0
2021-09-03 10:00:00	18.5	73	2.1	2	294	northwest wind	951	30	0	26
2021-09-03 11:00:00	20.9	60	1.8	2	299	northwest wind	951	30	0	60
2021-09-03 12:00:00	23.4	47	1.1	1	28	northeast wind	951	30	0	21
2021-09-03 13:00:00	24.2	46	2.1	2	24	northeast wind	951	30	0	40
2021-09-03 14:00:00	25.2	41	1.5	1	38	northeast wind	950	30	0	9
2021-09-03 15:00:00	25.8	39	2.6	2	57	northeast wind	950	30	0	62
2021-09-03 16:00:00	26.4	38	1.9	2	139	southeast wind	950	30	0	69
2021-09-03 17:00:00	26.6	40	0.8	1	118	southeast wind	949	30	0	56
2021-09-03 18:00:00	25.8	42	2.5	2	136	southeast wind	950	30	0	22
2021-09-03 19:00:00	24.6	49	1.7	2	41	northeast wind	950	30	0	68
2021-09-03 20:00:00	21.8	71	2.9	2	217	southwest wind	950	20.1	0.3	65
2021-09-03 21:00:00	21.2	61	2.3	2	241	southwest wind	951	30	0	31
2021-09-03 22:00:00	19.7	67	3	2	315	northwest wind	951	30	0	100
2021-09-03 23:00:00	17.7	78	1.9	2	237	southwest wind	952	30	0	55
2021-09-04 00:00:00	17.8	78	1.6	2	226	southwest wind	952	28	0	16
2021-09-04 01:00:00	17.3	77	1.8	2	243	southwest wind	952	28.6	0	0
2021-09-04 02:00:00	17	75	2.1	2	214	southwest wind	952	30	0	0
2021-09-04 03:00:00	16.9	73	1.4	1	236	southwest wind	952	30	0	0
2021-09-04 04:00:00	16.1	75	3	2	219	southwest wind	952	30	0	0
2021-09-04 05:00:00	15.9	73	1.4	1	215	southwest wind	952	30	0	4
2021-09-04 06:00:00	15.3	75	0.7	1	206	southwest wind	952	30	0	0
2021-09-04 07:00:00	15.4	73	2.8	2	239	southwest wind	952	30	0	0
2021-09-04 08:00:00	15.3	72	0.9	1	204	southwest wind	952	30	0	0
2021-09-04 09:00:00	16.7	69	1.5	1	130	southeast wind	953	30	0	0
2021-09-04 10:00:00	19.9	60	2.3	2	209	southwest wind	953	30	0	0
2021-09-04 11:00:00	23.2	48	1.4	1	295	northwest wind	953	30	0	0
2021-09-04 12:00:00	26.2	44	0.6	1	47	northeast wind	953	30	0	0
2021-09-04 13:00:00	27.3	41	1.6	2	155	southeast wind	953	30	0	0
2021-09-04 14:00:00	27.8	34	0.6	1	319	northwest wind	953	29.7	0	0
2021-09-04 15:00:00	28	31	3	2	37	northeast wind	953	30	0	0
2021-09-04 16:00:00	28.4	33	3.2	2	141	southeast wind	953	30	0	0
2021-09-04 17:00:00	28.8	31	0.9	1	131	southeast wind	952	30	0	0
2021-09-04 18:00:00	28.5	31	2.2	2	67	northeast wind	952	30	0	0
2021-09-04 19:00:00	28.1	33	2.6	2	133	southeast wind	953	30	0	0
2021-09-04 20:00:00	27.3	38	0.8	1	40	northeast wind	953	30	0	0
2021-09-04 21:00:00	23.4	54	1.3	1	57	northeast wind	953	26.3	0	0
2021-09-04 22:00:00	20.5	68	0.6	1	0	north wind	954	9.4	0	100

2021-09-04 23:00:00	19	73	3	2	215	southwest wind	955	14.7	0	0
2021-09-05 00:00:00	18.3	75	2.2	2	242	southwest wind	955	14.7	0	0
2021-09-05 01:00:00	17.1	80	0	0	243	southwest wind	955	19.9	0	0
2021-09-05 02:00:00	17	74	0.9	1	232	southwest wind	955	26.9	0	0
2021-09-05 03:00:00	17	72	1	1	213	southwest wind	955	30	0	0
2021-09-05 04:00:00	16.5	72	0.9	1	244	southwest wind	955	30	0	0
2021-09-05 05:00:00	15.7	74	3	2	206	southwest wind	955	30	0	0
2021-09-05 06:00:00	15.1	76	0.7	1	216	southwest wind	955	30	0	0
2021-09-05 07:00:00	14.4	79	0.7	1	234	southwest wind	955	30	0	0
2021-09-05 08:00:00	14.4	76	0.5	1	233	southwest wind	955	30	0	0
2021-09-05 09:00:00	15.9	70	1.6	2	204	southwest wind	956	30	0	0
2021-09-05 10:00:00	18.8	61	2.1	2	230	southwest wind	956	29.3	0	0
2021-09-05 11:00:00	22.3	52	0.8	1	211	southwest wind	956	29.7	0	0
2021-09-05 12:00:00	26.4	40	3	2	301	northwest wind	956	24.5	0	0
2021-09-05 13:00:00	28.9	33	0.6	1	151	southeast wind	956	29.6	0	0
2021-09-05 14:00:00	29.6	27	3.1	2	27	northeast wind	955	30	0	0
2021-09-05 15:00:00	29.7	32	1	1	57	northeast wind	954	29.1	0	0
2021-09-05 16:00:00	30.3	30	1.3	1	45	northeast wind	954	30	0	0
2021-09-05 17:00:00	29.9	30	2.5	2	119	southeast wind	954	30	0	0
2021-09-05 18:00:00	29.9	31	2.6	2	139	southeast wind	953	26.4	0	0
2021-09-05 19:00:00	29.6	28	2.4	2	39	northeast wind	953	30	0	0
2021-09-05 20:00:00	28.7	34	1	1	225	southwest wind	953	30	0	0
2021-09-05 21:00:00	23.9	52	2.7	2	325	northwest wind	954	17	0	0
2021-09-05 22:00:00	22.2	54	0.4	1	317	northwest wind	954	16.6	0	100
2021-09-05 23:00:00	20	63	1.8	2	209	southwest wind	954	13.3	0	0
2021-09-06 00:00:00	19.6	68	2.1	2	204	southwest wind	954	20.2	0	0
2021-09-06 01:00:00	18.6	70	0.8	1	221	southwest wind	954	24.5	0	0
2021-09-06 02:00:00	18.8	67	3.8	3	222	southwest wind	954	27.1	0	0
2021-09-06 03:00:00	17.8	69	2	2	245	southwest wind	954	30	0	0
2021-09-06 04:00:00	17	72	2.2	2	233	southwest wind	954	30	0	0
2021-09-06 05:00:00	16.1	74	0.3	1	204	southwest wind	954	30	0	0
2021-09-06 06:00:00	16	73	0.4	1	222	southwest wind	954	30	0	0
2021-09-06 07:00:00	15.9	72	0.7	1	227	southwest wind	954	30	0	0
2021-09-06 08:00:00	15.7	71	0.8	1	204	southwest wind	954	30	0	0
2021-09-06 09:00:00	17.3	66	2.7	2	236	southwest wind	954	19.5	0	0
2021-09-06 10:00:00	20.9	56	2.6	2	239	southwest wind	954	29.9	0	0
2021-09-06 11:00:00	24.8	45	2.2	2	243	southwest wind	954	29.9	0	0
2021-09-06 12:00:00	27.8	36	0.7	1	223	southwest wind	954	30	0	0

2021-09-06 13:00:00	29.6	28	1.8	2	302	northwest wind	953	26.4	0	0
2021-09-06 14:00:00	30.5	27	0.9	1	25	northeast wind	953	27.4	0	0
2021-09-06 15:00:00	30.7	28	0.3	1	45	northeast wind	952	27.4	0	0
2021-09-06 16:00:00	31.7	27	0.5	1	142	southeast wind	952	26.7	0	0
2021-09-06 17:00:00	31.7	25	2.7	2	42	northeast wind	951	29.3	0	0
2021-09-06 18:00:00	31.7	27	0.7	1	36	northeast wind	951	30	0	0
2021-09-06 19:00:00	31.4	28	0.7	1	131	southeast wind	951	23.1	0	0
2021-09-06 20:00:00	29.8	35	2.8	2	34	northeast wind	951	25	0	0
2021-09-06 21:00:00	25.1	48	1.2	1	309	northwest wind	952	12	0	0
2021-09-06 22:00:00	22.9	58	0.5	1	227	southwest wind	953	11.8	0	100
2021-09-06 23:00:00	21.2	66	0.3	1	204	southwest wind	953	13.5	0	0
2021-09-07 00:00:00	20.2	67	0.1	0	209	southwest wind	953	17.1	0	0
2021-09-07 01:00:00	18.9	68	3.2	2	317	northwest wind	953	23.4	0	0
2021-09-07 02:00:00	18.6	65	0.1	0	227	southwest wind	953	28.4	0	0
2021-09-07 03:00:00	17.4	69	2.6	2	210	southwest wind	953	29.8	0	0
2021-09-07 04:00:00	16.8	70	0.6	1	301	northwest wind	953	30	0	0
2021-09-07 05:00:00	16.5	69	1.9	2	234	southwest wind	952	30	0	0
2021-09-07 06:00:00	16.3	67	2.4	2	207	southwest wind	952	30	0	8
2021-09-07 07:00:00	15.9	67	1.7	2	219	southwest wind	953	30	0	16
2021-09-07 08:00:00	16.1	65	0.3	1	243	southwest wind	953	30	0	0
2021-09-07 09:00:00	17.1	62	2.2	2	241	southwest wind	953	30	0	0
2021-09-07 10:00:00	20.3	55	1.9	2	203	southwest wind	953	30	0	0
2021-09-07 11:00:00	24.5	49	1.1	1	239	southwest wind	953	27	0	0
2021-09-07 12:00:00	29.2	37	2	2	0	north wind	953	22.8	0	0
2021-09-07 13:00:00	31.6	28	0.6	1	208	southwest wind	952	24.7	0	0
2021-09-07 14:00:00	33.1	25	1.6	2	114	southeast wind	951	24.7	0	0
2021-09-07 15:00:00	33.6	23	1.3	1	117	southeast wind	951	27.2	0	0
2021-09-07 16:00:00	33.8	20	1.2	1	123	southeast wind	950	30	0	0
2021-09-07 17:00:00	33.8	22	2.6	2	153	southeast wind	950	30	0	0
2021-09-07 18:00:00	34	22	2.8	2	126	southeast wind	949	26.9	0	0
2021-09-07 19:00:00	33.4	25	0.6	1	123	southeast wind	949	27.1	0	0
2021-09-07 20:00:00	31	31	1.3	1	42	northeast wind	949	25.4	0	0
2021-09-07 21:00:00	25.5	51	1.4	1	325	northwest wind	950	18.5	0	0
2021-09-07 22:00:00	22.4	60	1.9	2	295	northwest wind	950	7.4	0	100
2021-09-07 23:00:00	21.1	63	2.1	2	311	northwest wind	951	19.2	0	2
2021-09-08 00:00:00	19.6	65	2.2	2	297	northwest wind	951	16.5	0	11
2021-09-08 01:00:00	20.7	51	5	3	314	northwest wind	951	16.5	0	0
2021-09-08 02:00:00	20.3	49	0.6	1	301	northwest wind	951	30	0	0

2021-09-08 03:00:00	19.4	50	2.8	2	251	west wind	950	30	0	0
2021-09-08 04:00:00	17.8	56	0.7	1	313	northwest wind	950	30	0	0
2021-09-08 05:00:00	16.9	59	0.3	1	301	northwest wind	949	30	0	14
2021-09-08 06:00:00	17.2	54	1.4	1	322	northwest wind	949	30	0	0
2021-09-08 07:00:00	16.7	54	0.8	1	294	northwest wind	949	30	0	14
2021-09-08 08:00:00	16.3	54	0.5	1	310	northwest wind	950	30	0	0
2021-09-08 09:00:00	17.7	50	0.2	0	327	northwest wind	950	30	0	0
2021-09-08 10:00:00	20.8	46	2.6	2	336	northwest wind	950	28.8	0	0
2021-09-08 11:00:00	25.4	44	1.3	1	321	northwest wind	950	28.8	0	0
2021-09-08 12:00:00	30.3	28	2.3	2	126	southeast wind	949	22.3	0	0
2021-09-08 13:00:00	31.8	23	0.3	1	137	southeast wind	949	24.2	0	0
2021-09-08 14:00:00	32.4	21	2.7	2	147	southeast wind	948	24.2	0	0
2021-09-08 15:00:00	33	21	2.8	2	146	southeast wind	948	24.2	0	0
2021-09-08 16:00:00	33.7	22	0.1	0	141	southeast wind	947	27.6	0	0
2021-09-08 17:00:00	34.2	21	1	1	113	southeast wind	946	25.4	0	0
2021-09-08 18:00:00	34.3	18	0.2	0	118	southeast wind	946	23.2	0	0
2021-09-08 19:00:00	33.6	20	2.1	2	137	southeast wind	946	23.2	0	0
2021-09-08 20:00:00	29.3	30	2.5	2	130	southeast wind	946	23.2	0	0
2021-09-08 21:00:00	26.1	41	0.1	0	336	northwest wind	946	12.2	0	0
2021-09-08 22:00:00	24.2	47	1.8	2	316	northwest wind	947	15	0	100
2021-09-08 23:00:00	21.5	56	0.7	1	0	north wind	947	15	0	16
2021-09-09 00:00:00	20.3	63	1.7	2	325	northwest wind	947	19.2	0	60
2021-09-09 01:00:00	20.8	50	1.6	2	295	northwest wind	948	26.2	0	65
2021-09-09 02:00:00	19.1	54	0.7	1	299	northwest wind	948	28.7	0	38
2021-09-09 03:00:00	18.3	54	0.3	1	302	northwest wind	948	30	0	33
2021-09-09 04:00:00	17.8	53	0.3	1	203	southwest wind	947	30	0	19
2021-09-09 05:00:00	16.8	55	0.8	1	230	southwest wind	947	30	0	12
2021-09-09 06:00:00	16.8	53	0.3	1	336	northwest wind	947	30	0	52
2021-09-09 07:00:00	16.6	50	0.5	1	311	northwest wind	947	30	0	57
2021-09-09 08:00:00	15.9	52	1.1	1	332	northwest wind	948	30	0	2
2021-09-09 09:00:00	17.7	50	3.1	2	307	northwest wind	948	30	0	36
2021-09-09 10:00:00	20.7	47	1.3	1	309	northwest wind	948	30	0	31
2021-09-09 11:00:00	25	42	1.5	1	120	southeast wind	948	30	0	48
2021-09-09 12:00:00	30.1	26	2.5	2	118	southeast wind	947	16.3	0	58
2021-09-09 13:00:00	29	23	0	0	147	southeast wind	947	20.2	0	83
2021-09-09 14:00:00	28.6	24	4	3	123	southeast wind	946	20.2	0	83
2021-09-09 15:00:00	28.9	26	0.2	0	156	southeast wind	946	20.2	0	69
2021-09-09 16:00:00	28.4	30	1.8	2	119	southeast wind	946	24.5	0	70

2021-09-09 17:00:00	27.1	42	2.1	2	142	southeast wind	946	24.5	0	90
2021-09-09 18:00:00	28.2	28	1.5	1	151	southeast wind	946	13.3	0	100
2021-09-09 19:00:00	28.6	24	0.9	1	127	southeast wind	946	13.3	0	73
2021-09-09 20:00:00	26.1	32	2.2	2	126	southeast wind	947	13.3	0	45
2021-09-09 21:00:00	23.4	36	2.2	2	227	southwest wind	948	19	0	26
2021-09-09 22:00:00	23.2	33	0	0	146	southeast wind	949	17.5	0	61
2021-09-09 23:00:00	22.2	35	0.1	0	138	southeast wind	949	23.3	0	36
2021-09-10 00:00:00	20.9	39	2.5	2	134	southeast wind	949	25.8	0	7
2021-09-10 01:00:00	19.1	49	3.1	2	294	northwest wind	949	25.8	0	7
2021-09-10 02:00:00	17.8	53	1.7	2	227	southwest wind	950	29.6	0	60
2021-09-10 03:00:00	19.3	48	1.4	1	217	southwest wind	950	30	0	100
2021-09-10 04:00:00	19.1	48	1.6	2	113	southeast wind	950	30	0	100
2021-09-10 05:00:00	18.9	51	0.8	1	24	northeast wind	950	30	0	100
2021-09-10 06:00:00	18.5	52	2.8	2	136	southeast wind	950	30	0	100
2021-09-10 07:00:00	16.8	60	1.5	1	40	northeast wind	950	29.6	0	81
2021-09-10 08:00:00	15.2	67	2.6	2	304	northwest wind	951	30	0	48
2021-09-10 09:00:00	14.9	72	0.8	1	308	northwest wind	951	25.6	0	83
2021-09-10 10:00:00	15.8	69	1.7	2	315	northwest wind	951	25.6	0	70
2021-09-10 11:00:00	17.3	66	0.4	1	325	northwest wind	951	25.6	0	83
2021-09-10 12:00:00	19.9	58	2.5	2	239	southwest wind	951	21.3	0	100
2021-09-10 13:00:00	21.3	49	4	3	113	southeast wind	951	21.8	0	90
2021-09-10 14:00:00	21.2	45	3.8	3	153	southeast wind	951	28.7	0	100
2021-09-10 15:00:00	20.2	46	3.1	2	115	southeast wind	951	29.7	0	90
2021-09-10 16:00:00	19.2	49	2.6	2	149	southeast wind	951	30	0	100
2021-09-10 17:00:00	20	44	2.5	2	128	southeast wind	951	30	0	100
2021-09-10 18:00:00	19.9	46	0.9	1	138	southeast wind	951	24.3	0	90
2021-09-10 19:00:00	19.2	49	1.6	2	221	southwest wind	952	29.4	0	100
2021-09-10 20:00:00	15.2	85	2.9	2	336	northwest wind	953	11.5	1.3	90
2021-09-10 21:00:00	14.8	91	1.5	1	205	southwest wind	954	21	0.5	100
2021-09-10 22:00:00	14.8	90	2	2	311	northwest wind	955	9.3	0.6	96
2021-09-10 23:00:00	14.5	94	1.3	1	318	northwest wind	956	7.6	1.2	96
2021-09-11 00:00:00	14	88	2.6	2	254	west wind	956	16.8	0.8	100
2021-09-11 01:00:00	14	85	2.8	2	311	northwest wind	956	30	0	100
2021-09-11 02:00:00	13.8	86	1.2	1	332	northwest wind	957	28	0	39
2021-09-11 03:00:00	13.5	86	3.1	2	323	northwest wind	957	29.8	0	100
2021-09-11 04:00:00	12.6	89	2.6	2	293	northwest wind	956	29.5	0	68
2021-09-11 05:00:00	11.9	90	2	2	314	northwest wind	956	28.7	0	0
2021-09-11 06:00:00	10.7	93	1.8	2	317	northwest wind	956	15.9	0	0

2021-09-11 07:00:00	10.1	93	1	1	296	northwest wind	956	23.8	0	0
2021-09-11 08:00:00	9.4	92	0.6	1	309	northwest wind	956	25.3	0	0
2021-09-11 09:00:00	10.6	89	0.1	0	320	northwest wind	956	21.2	0	0
2021-09-11 10:00:00	13.3	83	0.2	0	307	northwest wind	956	21.2	0	0
2021-09-11 11:00:00	17.2	59	1.6	2	63	northeast wind	956	29	0	0
2021-09-11 12:00:00	18.5	52	1.5	1	121	southeast wind	956	30	0	0
2021-09-11 13:00:00	19.8	49	0.7	1	154	southeast wind	955	26.8	0	0
2021-09-11 14:00:00	20.7	44	2.2	2	220	southwest wind	954	28.5	0	0
2021-09-11 15:00:00	21.9	42	3.1	2	130	southeast wind	953	30	0	0
2021-09-11 16:00:00	21.8	39	0.8	1	154	southeast wind	952	30	0	0
2021-09-11 17:00:00	22.3	41	1.6	2	133	southeast wind	951	30	0	0
2021-09-11 18:00:00	22.3	42	1.6	2	120	southeast wind	951	30	0	0
2021-09-11 19:00:00	21.9	40	1.8	2	128	southeast wind	951	30	0	0
2021-09-11 20:00:00	21	42	1.7	2	121	southeast wind	951	30	0	0
2021-09-11 21:00:00	18.1	57	0.3	1	319	northwest wind	951	23.3	0	0
2021-09-11 22:00:00	15.7	69	2.4	2	324	northwest wind	952	21.5	0	0
2021-09-11 23:00:00	14.3	74	1.8	2	294	northwest wind	952	19.6	0	0
2021-09-12 00:00:00	13.8	76	2.5	2	296	northwest wind	952	21.4	0	0
2021-09-12 01:00:00	13.1	79	2.2	2	328	northwest wind	952	22.5	0	0
2021-09-12 02:00:00	12.8	79	1.9	2	317	northwest wind	952	25.5	0	0
2021-09-12 03:00:00	12.5	79	1.5	1	319	northwest wind	951	28.9	0	0
2021-09-12 04:00:00	12.3	79	0.1	0	294	northwest wind	951	30	0	0
2021-09-12 05:00:00	11.9	81	0.6	1	293	northwest wind	951	29.1	0	0
2021-09-12 06:00:00	11.2	83	3.2	2	298	northwest wind	951	28.7	0	0
2021-09-12 07:00:00	11	83	2.6	2	306	northwest wind	951	30	0	0
2021-09-12 08:00:00	11	81	1.2	1	298	northwest wind	951	30	0	0
2021-09-12 09:00:00	12.2	78	2.3	2	298	northwest wind	951	28.4	0	0
2021-09-12 10:00:00	15.2	67	0.2	0	299	northwest wind	951	30	0	0
2021-09-12 11:00:00	18.6	60	2.7	2	301	northwest wind	951	29.7	0	0
2021-09-12 12:00:00	21.5	47	0.8	1	312	northwest wind	951	30	0	0
2021-09-12 13:00:00	22.8	44	0.7	1	219	southwest wind	950	30	0	0
2021-09-12 14:00:00	24	40	0.8	1	167	south wind	950	30	0	60
2021-09-12 15:00:00	23.4	47	1.2	1	27	northeast wind	950	30	0	83
2021-09-12 16:00:00	22	56	0.5	1	49	northeast wind	950	30	0	100
2021-09-12 17:00:00	21	55	2.9	2	227	southwest wind	951	30	0	100
2021-09-12 18:00:00	22.1	50	2.1	2	27	northeast wind	951	30	0	90
2021-09-12 19:00:00	23.1	44	1.6	2	113	southeast wind	951	30	0	14
2021-09-12 20:00:00	21.9	46	2.1	2	129	southeast wind	952	30	0	45

2021-09-12 21:00:00	19.7	57	1.1	1	141	southeast wind	953	30	0	16
2021-09-12 22:00:00	17	70	0.8	1	312	northwest wind	953	17.4	0	21
2021-09-12 23:00:00	16.3	71	2.5	2	325	northwest wind	953	21.5	0	7
2021-09-13 00:00:00	14.2	81	1.7	2	233	southwest wind	953	20.7	0	0
2021-09-13 01:00:00	14.6	79	2.8	2	335	northwest wind	953	25.1	0	0
2021-09-13 02:00:00	14.1	79	2.2	2	309	northwest wind	953	26.5	0	0
2021-09-13 03:00:00	13.6	79	2.3	2	311	northwest wind	953	28.3	0	0
2021-09-13 04:00:00	12.4	82	0.9	1	307	northwest wind	953	30	0	0
2021-09-13 05:00:00	12	83	1.8	2	309	northwest wind	953	30	0	3
2021-09-13 06:00:00	11.5	83	0.7	1	298	northwest wind	953	30	0	30
2021-09-13 07:00:00	11.2	82	1.8	2	323	northwest wind	953	30	0	46
2021-09-13 08:00:00	10.7	82	3.2	2	306	northwest wind	953	30	0	0
2021-09-13 09:00:00	12.2	76	3	2	321	northwest wind	954	30	0	0
2021-09-13 10:00:00	15.4	65	1.3	1	296	northwest wind	954	30	0	0
2021-09-13 11:00:00	18.6	58	0.8	1	296	northwest wind	954	30	0	0
2021-09-13 12:00:00	22	43	2.5	2	322	northwest wind	953	30	0	0
2021-09-13 13:00:00	23.5	40	1.3	1	124	southeast wind	953	30	0	0
2021-09-13 14:00:00	24.4	35	2.1	2	223	southwest wind	952	28.1	0	0
2021-09-13 15:00:00	25.1	32	1	1	149	southeast wind	952	30	0	0
2021-09-13 16:00:00	25.4	33	0.9	1	147	southeast wind	951	28.9	0	0
2021-09-13 17:00:00	25.5	31	2.8	2	116	southeast wind	951	28.9	0	0
2021-09-13 18:00:00	25.5	30	4	3	135	southeast wind	951	30	0	0
2021-09-13 19:00:00	25.1	28	2.5	2	113	southeast wind	951	30	0	0
2021-09-13 20:00:00	23.7	30	0.2	0	120	southeast wind	952	30	0	0
2021-09-13 21:00:00	20.5	42	0.6	1	54	northeast wind	952	28.2	0	0
2021-09-13 22:00:00	17.4	56	0.1	0	310	northwest wind	953	28.2	0	0
2021-09-13 23:00:00	16	63	0.3	1	31	northeast wind	954	28.2	0	0
2021-09-14 00:00:00	15.1	68	3	2	299	northwest wind	954	24.8	0	0
2021-09-14 01:00:00	16.6	50	2.6	2	30	northeast wind	954	27.3	0	0
2021-09-14 02:00:00	13.8	70	1.6	2	297	northwest wind	955	24.7	0	0
2021-09-14 03:00:00	12.4	76	2.2	2	315	northwest wind	955	26	0	0
2021-09-14 04:00:00	11.6	80	3	2	294	northwest wind	955	28.7	0	0
2021-09-14 05:00:00	11	80	3	2	308	northwest wind	955	29.5	0	0
2021-09-14 06:00:00	10.4	80	1.3	1	324	northwest wind	955	30	0	0
2021-09-14 07:00:00	9.8	84	0.2	0	327	northwest wind	956	30	0	0
2021-09-14 08:00:00	9.5	84	0.5	1	318	northwest wind	956	30	0	0
2021-09-14 09:00:00	10.6	80	2.9	2	294	northwest wind	957	24	0	0
2021-09-14 10:00:00	14.1	70	2.4	2	307	northwest wind	957	24	0	0

2021-09-14 11:00:00	17.9	53	2.8	2	217	southwest wind	957	22.5	0	0
2021-09-14 12:00:00	20.6	42	0.2	0	233	southwest wind	958	27.2	0	0
2021-09-14 13:00:00	21.6	34	2	2	115	southeast wind	957	28.6	0	0
2021-09-14 14:00:00	22.4	32	0.1	0	96	east wind	957	30	0	0
2021-09-14 15:00:00	23.6	34	0.3	1	211	southwest wind	956	30	0	0
2021-09-14 16:00:00	23.8	31	0.2	0	319	northwest wind	956	30	0	4
2021-09-14 17:00:00	23.8	32	0.2	0	143	southeast wind	955	23.8	0	72
2021-09-14 18:00:00	23.5	32	3	2	49	northeast wind	955	30	0	100
2021-09-14 19:00:00	24	33	0.8	1	209	southwest wind	955	30	0	68
2021-09-14 20:00:00	22	44	1.3	1	223	southwest wind	956	30	0	32
2021-09-14 21:00:00	18.1	56	3.1	2	320	northwest wind	956	17.1	0	0
2021-09-14 22:00:00	16	65	2.7	2	308	northwest wind	957	10.6	0	0
2021-09-14 23:00:00	13.7	75	2.9	2	229	southwest wind	957	17.3	0	12
2021-09-15 00:00:00	13.6	73	2.9	2	305	northwest wind	957	17.1	0	0
2021-09-15 01:00:00	12.9	75	1.6	2	293	northwest wind	957	21.6	0	0
2021-09-15 02:00:00	12.2	76	1.1	1	331	northwest wind	957	28.5	0	0
2021-09-15 03:00:00	11.9	75	2.5	2	323	northwest wind	956	28.2	0	0
2021-09-15 04:00:00	11.3	79	1.6	2	309	northwest wind	956	30	0	0
2021-09-15 05:00:00	11	77	2.9	2	295	northwest wind	956	30	0	0
2021-09-15 06:00:00	10.9	75	2.9	2	324	northwest wind	956	30	0	0
2021-09-15 07:00:00	11	75	0.1	0	331	northwest wind	956	30	0	0
2021-09-15 08:00:00	10.4	75	1.2	1	299	northwest wind	957	30	0	0
2021-09-15 09:00:00	11	74	2.4	2	321	northwest wind	957	27.7	0	0
2021-09-15 10:00:00	14.9	66	1.3	1	219	southwest wind	957	21.9	0	0
2021-09-15 11:00:00	18.7	39	3.2	2	234	southwest wind	957	21.9	0	0
2021-09-15 12:00:00	20.8	32	0.5	1	113	southeast wind	957	30	0	0
2021-09-15 13:00:00	21.6	29	3	2	295	northwest wind	956	30	0	0
2021-09-15 14:00:00	23	29	3	2	113	southeast wind	955	30	0	0
2021-09-15 15:00:00	24.1	30	2.2	2	134	southeast wind	954	30	0	0
2021-09-15 16:00:00	24.6	29	1.5	1	114	southeast wind	953	30	0	0
2021-09-15 17:00:00	24.8	30	0.9	1	40	northeast wind	953	30	0	28
2021-09-15 18:00:00	24.8	28	0.1	0	116	southeast wind	953	30	0	0
2021-09-15 19:00:00	24.9	32	1.6	2	55	northeast wind	953	30	0	41
2021-09-15 20:00:00	22.2	42	0.6	1	226	southwest wind	953	30	0	0
2021-09-15 21:00:00	18.7	54	1.4	1	308	northwest wind	953	30	0	0
2021-09-15 22:00:00	15.7	69	2.4	2	306	northwest wind	954	11.3	0	4
2021-09-15 23:00:00	14.6	71	1	1	231	southwest wind	954	11.3	0	0
2021-09-16 00:00:00	14.4	70	0.5	1	296	northwest wind	953	18.7	0	0

2021-09-16 01:00:00	14.6	66	1.8	2	335	northwest wind	953	21.5	0	0
2021-09-16 02:00:00	14.1	67	2.3	2	301	northwest wind	952	28.9	0	0
2021-09-16 03:00:00	13.6	67	1.1	1	319	northwest wind	952	29.7	0	0
2021-09-16 04:00:00	12.6	72	2.9	2	312	northwest wind	951	30	0	14
2021-09-16 05:00:00	12.3	73	2.2	2	336	northwest wind	951	30	0	43
2021-09-16 06:00:00	11.7	75	2.4	2	312	northwest wind	951	30	0	21
2021-09-16 07:00:00	11.4	75	0.4	1	325	northwest wind	950	30	0	33
2021-09-16 08:00:00	10.9	77	0.4	1	320	northwest wind	951	30	0	52
2021-09-16 09:00:00	11.8	74	3.1	2	297	northwest wind	951	27.8	0	63
2021-09-16 10:00:00	15.1	65	0.3	1	332	northwest wind	951	27.8	0	0
2021-09-16 11:00:00	14	51	0.5	1	327	northwest wind	951	27.8	0	2
2021-09-16 12:00:00	23.8	37	2.2	2	121	southeast wind	950	27.8	0	4
2021-09-16 13:00:00	25.8	26	0.1	0	120	southeast wind	950	27.8	0	68
2021-09-16 14:00:00	26.5	28	1.3	1	236	southwest wind	949	27.8	0	0
2021-09-16 15:00:00	27.4	27	1.5	1	157	southeast wind	948	27.8	0	0
2021-09-16 16:00:00	27.6	27	0.2	0	155	southeast wind	948	27.6	0	0
2021-09-16 17:00:00	27.4	29	0	0	241	southwest wind	948	30	0	91
2021-09-16 18:00:00	26.4	35	2.9	2	231	southwest wind	948	26.8	0	90
2021-09-16 19:00:00	25.4	39	0.3	1	0	north wind	948	19.4	0	100
2021-09-16 20:00:00	22.5	53	1.6	2	223	southwest wind	949	19.9	0	90
2021-09-16 21:00:00	22.7	41	7.6	4	148	southeast wind	950	5.9	0	100
2021-09-16 22:00:00	20.6	55	2.1	2	24	northeast wind	951	30	0.2	100
2021-09-16 23:00:00	18.7	60	1.6	2	203	southwest wind	952	30	0	90
2021-09-17 00:00:00	17.5	64	0.7	1	335	northwest wind	952	30	0	68
2021-09-17 01:00:00	17.4	65	2.5	2	294	northwest wind	952	30	0	83
2021-09-17 02:00:00	18.2	58	2.1	2	304	northwest wind	951	20.4	0.1	72
2021-09-17 03:00:00	15.9	68	1.3	1	313	northwest wind	951	30	0	12
2021-09-17 04:00:00	14.2	73	1.9	2	315	northwest wind	951	30	0	0
2021-09-17 05:00:00	13.4	74	2.5	2	294	northwest wind	951	30	0	19
2021-09-17 06:00:00	13.7	70	4.3	3	319	northwest wind	950	30	0	16
2021-09-17 07:00:00	14.3	66	1.2	1	327	northwest wind	950	30	0	40
2021-09-17 08:00:00	13.4	69	1.3	1	308	northwest wind	951	30	0	0
2021-09-17 09:00:00	13.6	70	1.5	1	309	northwest wind	951	30	0	0
2021-09-17 10:00:00	17	57	0.8	1	326	northwest wind	951	30	0	0
2021-09-17 11:00:00	20.3	52	1.7	2	327	northwest wind	951	30	0	0
2021-09-17 12:00:00	24.4	38	3	2	209	southwest wind	950	30	0	0
2021-09-17 13:00:00	26.7	24	2.3	2	131	southeast wind	950	30	0	0
2021-09-17 14:00:00	27.1	24	1.4	1	56	northeast wind	949	30	0	0

2021-09-17 15:00:00	27.8	22	3.5	3	93	east wind	948	30	0	0
2021-09-17 16:00:00	28.7	22	3	2	138	southeast wind	947	30	0	0
2021-09-17 17:00:00	28.8	22	1.6	2	153	southeast wind	947	30	0	0
2021-09-17 18:00:00	28.5	24	1.1	1	117	southeast wind	946	30	0	0
2021-09-17 19:00:00	28	24	0.7	1	114	southeast wind	946	30	0	0
2021-09-17 20:00:00	25.9	31	1.2	1	150	southeast wind	946	30	0	0
2021-09-17 21:00:00	21.4	45	0	0	308	northwest wind	947	30	0	0
2021-09-17 22:00:00	19.1	54	0.2	0	316	northwest wind	947	30	0	0
2021-09-17 23:00:00	17.1	60	1.2	1	296	northwest wind	948	30	0	0
2021-09-18 00:00:00	16.3	59	2.7	2	309	northwest wind	948	30	0	0
2021-09-18 01:00:00	16.8	55	0.9	1	310	northwest wind	948	30	0	0
2021-09-18 02:00:00	16.4	55	1.6	2	304	northwest wind	948	30	0	0
2021-09-18 03:00:00	15.6	57	2.8	2	325	northwest wind	948	30	0	0
2021-09-18 04:00:00	14.6	60	3.2	2	327	northwest wind	948	30	0	16
2021-09-18 05:00:00	14.9	57	0.4	1	337	northwest wind	947	30	0	0
2021-09-18 06:00:00	14.8	54	3	2	332	northwest wind	947	30	0	19
2021-09-18 07:00:00	13.4	60	2.3	2	299	northwest wind	948	30	0	7
2021-09-18 08:00:00	12.8	62	1.8	2	332	northwest wind	948	30	0	7
2021-09-18 09:00:00	13.9	58	3.1	2	336	northwest wind	948	30	0	0
2021-09-18 10:00:00	16.8	51	1.6	2	303	northwest wind	948	30	0	50
2021-09-18 11:00:00	20.4	43	1.8	2	303	northwest wind	948	30	0	0
2021-09-18 12:00:00	25.2	35	2.4	2	300	northwest wind	948	30	0	0
2021-09-18 13:00:00	27.6	29	2.3	2	120	southeast wind	947	30	0	0
2021-09-18 14:00:00	28.1	25	1.5	1	136	southeast wind	947	26.5	0	20
2021-09-18 15:00:00	29	24	1.7	2	141	southeast wind	946	29.6	0	20
2021-09-18 16:00:00	29.2	23	0.8	1	147	southeast wind	946	28	0	20
2021-09-18 17:00:00	29.3	24	0	0	50	northeast wind	946	28.8	0	20
2021-09-18 18:00:00	29.6	23	1.8	2	134	southeast wind	946	27.9	0	20
2021-09-18 19:00:00	29.2	27	2.7	2	237	southwest wind	947	28.5	0	60
2021-09-18 20:00:00	25.3	37	0.5	1	234	southwest wind	947	23.5	0	20
2021-09-18 21:00:00	21.5	54	0.1	0	296	northwest wind	948	11.1	0	60
2021-09-18 22:00:00	18.7	66	0.3	1	334	northwest wind	948	8.8	0	60
2021-09-18 23:00:00	17.7	66	2.9	2	247	southwest wind	949	14.3	0	60
2021-09-19 00:00:00	16.8	64	3.2	2	330	northwest wind	949	17.8	0	20
2021-09-19 01:00:00	16.1	68	0.1	0	328	northwest wind	949	16.9	0	20
2021-09-19 02:00:00	15.6	65	2	2	309	northwest wind	949	22.8	0	60
2021-09-19 03:00:00	15	62	1.4	1	321	northwest wind	949	24.6	0	20
2021-09-19 04:00:00	14.2	63	0	0	333	northwest wind	948	27.3	0	20

2021-09-19 05:00:00	13.8	64	1.2	1	315	northwest wind	948	27.9	0	60
2021-09-19 06:00:00	12.9	66	3.2	2	321	northwest wind	948	30	0	60
2021-09-19 07:00:00	12.5	66	0.2	0	299	northwest wind	948	29.8	0	90
2021-09-19 08:00:00	12.6	64	2.5	2	302	northwest wind	948	30	0	90
2021-09-19 09:00:00	13.4	61	1.1	1	315	northwest wind	948	20	0	90
2021-09-19 10:00:00	13.7	65	2.4	2	312	northwest wind	949	21.1	0	90
2021-09-19 11:00:00	18.1	54	1.1	1	206	southwest wind	949	16.3	0	60
2021-09-19 12:00:00	22.7	35	0.3	1	325	northwest wind	949	24	0	60
2021-09-19 13:00:00	26	26	1.6	2	156	southeast wind	948	21.4	0	60
2021-09-19 14:00:00	27.8	22	2.2	2	117	southeast wind	947	22.8	0	20
2021-09-19 15:00:00	28.5	22	1.1	1	155	southeast wind	947	23.5	0	60
2021-09-19 16:00:00	29.2	22	0	0	129	southeast wind	946	23.4	0	20
2021-09-19 17:00:00	29.2	23	2.2	2	113	southeast wind	945	20.6	0	20
2021-09-19 18:00:00	28.8	23	1.1	1	113	southeast wind	946	24.7	0	60
2021-09-19 19:00:00	28.1	32	0.1	0	247	southwest wind	946	27	0	20
2021-09-19 20:00:00	24.1	37	1.1	1	131	southeast wind	946	27	0	20
2021-09-19 21:00:00	21.6	45	1.1	1	30	northeast wind	947	7	0	60
2021-09-19 22:00:00	20.3	45	0	0	296	northwest wind	948	15.5	0	60
2021-09-19 23:00:00	18	55	0.4	1	321	northwest wind	948	15.5	0	20
2021-09-20 00:00:00	18	51	0.1	0	298	northwest wind	948	15.5	0	60
2021-09-20 01:00:00	17.1	55	1.1	1	294	northwest wind	947	15.5	0	20
2021-09-20 02:00:00	16.3	58	0.1	0	294	northwest wind	948	15.5	0	60
2021-09-20 03:00:00	15.8	60	0.8	1	243	southwest wind	948	15.5	0	60
2021-09-20 04:00:00	16.2	56	1.4	1	216	southwest wind	947	15.5	0	90
2021-09-20 05:00:00	15.7	58	2.7	2	256	west wind	948	15.5	0	90
2021-09-20 06:00:00	15.1	64	2.7	2	256	west wind	948	15.5	0	90
2021-09-20 07:00:00	14	67	1.3	1	293	northwest wind	948	15.5	0	60
2021-09-20 08:00:00	13	74	0.4	1	304	northwest wind	949	15.5	0	90
2021-09-20 09:00:00	14.3	67	2.6	2	318	northwest wind	950	14.9	0	90
2021-09-20 10:00:00	17.8	41	0.4	1	153	southeast wind	950	11.6	0	90
2021-09-20 11:00:00	18.9	41	1.2	1	116	southeast wind	951	11.2	0	60
2021-09-20 12:00:00	19.9	38	0.3	1	147	southeast wind	951	14.8	0	20
2021-09-20 13:00:00	21.6	34	2.3	2	145	southeast wind	951	21.3	0	20
2021-09-20 14:00:00	21.8	32	4.1	3	139	southeast wind	951	26.5	0	20
2021-09-20 15:00:00	22.5	30	3.9	3	117	southeast wind	951	17.7	0	20
2021-09-20 16:00:00	22.7	29	1.3	1	116	southeast wind	950	16.6	0	20
2021-09-20 17:00:00	23.8	27	1.6	2	141	southeast wind	950	17	0	20
2021-09-20 18:00:00	23.4	27	2.2	2	134	southeast wind	950	17	0	20

2021-09-20 19:00:00	21.2	29	4.5	3	141	southeast wind	950	18.6	0	20
2021-09-20 20:00:00	18.9	31	5.4	3	136	southeast wind	951	12.2	0	20
2021-09-20 21:00:00	17.3	32	2.6	2	124	southeast wind	952	10.4	0	20
2021-09-20 22:00:00	16.6	32	2.6	2	132	southeast wind	953	19.4	0	20
2021-09-20 23:00:00	15.2	37	0.2	0	326	northwest wind	954	23.2	0	20
2021-09-21 00:00:00	13.5	43	2.8	2	314	northwest wind	954	22.6	0	20
2021-09-21 01:00:00	12	49	1	1	31	northeast wind	954	15.9	0	20
2021-09-21 02:00:00	10.8	57	0.7	1	302	northwest wind	954	18.9	0	20
2021-09-21 03:00:00	9.4	69	0.8	1	321	northwest wind	954	25.4	0	20
2021-09-21 04:00:00	8.1	74	2.2	2	323	northwest wind	953	25.4	0	20
2021-09-21 05:00:00	7.8	76	1.7	2	293	northwest wind	953	25.4	0	20
2021-09-21 06:00:00	6.6	81	3	2	221	southwest wind	953	25.4	0	20
2021-09-21 07:00:00	6.6	77	0.3	1	336	northwest wind	953	25.4	0	20
2021-09-21 08:00:00	5.5	83	0.7	1	306	northwest wind	954	25.4	0	20
2021-09-21 09:00:00	6.7	78	1.5	1	301	northwest wind	954	17.2	0	20
2021-09-21 10:00:00	10.1	63	3.1	2	204	southwest wind	954	20.2	0	60
2021-09-21 11:00:00	14.2	41	2.3	2	145	southeast wind	954	15.8	0	60
2021-09-21 12:00:00	16.7	35	0	0	154	southeast wind	954	24.2	0	60
2021-09-21 13:00:00	17.1	29	0.3	1	140	southeast wind	953	30	0	60
2021-09-21 14:00:00	17.4	26	2.3	2	136	southeast wind	952	30	0	90
2021-09-21 15:00:00	18.1	24	1.7	2	324	northwest wind	952	30	0	60
2021-09-21 16:00:00	18.6	24	4.4	3	139	southeast wind	951	30	0	60
2021-09-21 17:00:00	18	24	1.1	1	147	southeast wind	951	30	0	90
2021-09-21 18:00:00	18.3	24	0.7	1	156	southeast wind	951	30	0	60
2021-09-21 19:00:00	18.1	27	1.3	1	206	southwest wind	951	30	0	60
2021-09-21 20:00:00	17	28	0.7	1	137	southeast wind	952	30	0	20
2021-09-21 21:00:00	14.3	43	1.6	2	115	southeast wind	952	30	0	20
2021-09-21 22:00:00	11.8	49	0.5	1	311	northwest wind	953	20.7	0	20
2021-09-21 23:00:00	10	59	1.6	2	336	northwest wind	953	15	0	20
2021-09-22 00:00:00	8.6	63	1.2	1	313	northwest wind	953	18.8	0	20
2021-09-22 01:00:00	7.9	65	2.5	2	310	northwest wind	953	24.8	0	20
2021-09-22 02:00:00	7.2	68	0.2	0	299	northwest wind	953	21.8	0	20
2021-09-22 03:00:00	7	68	1	1	297	northwest wind	953	29.3	0	20
2021-09-22 04:00:00	5.9	73	1.8	2	313	northwest wind	953	29.3	0	20
2021-09-22 05:00:00	5.5	76	1	1	330	northwest wind	953	30	0	20
2021-09-22 06:00:00	5.3	73	2.9	2	322	northwest wind	953	30	0	20
2021-09-22 07:00:00	4.9	74	1.4	1	319	northwest wind	954	30	0	20
2021-09-22 08:00:00	4.6	75	2.7	2	316	northwest wind	954	30	0	20

2021-09-22 09:00:00	5.4	74	2.6	2	317	northwest wind	954	30	0	20
2021-09-22 10:00:00	9	62	1.2	1	327	northwest wind	955	25.8	0	20
2021-09-22 11:00:00	13.9	44	0.7	1	30	northeast wind	955	19.6	0	20
2021-09-22 12:00:00	15.8	34	0.5	1	131	southeast wind	955	21.3	0	20
2021-09-22 13:00:00	16.8	33	1.2	1	146	southeast wind	955	26.1	0	60
2021-09-22 14:00:00	17.8	32	2.6	2	140	southeast wind	954	30	0	60
2021-09-22 15:00:00	18.7	29	1.1	1	143	southeast wind	953	30	0	20
2021-09-22 16:00:00	19.3	25	0.4	1	149	southeast wind	953	30	0	60
2021-09-22 17:00:00	18.9	24	4.6	3	129	southeast wind	953	30	0	60
2021-09-22 18:00:00	18.8	23	2.5	2	113	southeast wind	953	30	0	60
2021-09-22 19:00:00	18.4	26	1.9	2	131	southeast wind	953	30	0	20
2021-09-22 20:00:00	17.2	29	0.6	1	144	southeast wind	954	30	0	20
2021-09-22 21:00:00	13.5	48	1.4	1	318	northwest wind	954	29.9	0	20
2021-09-22 22:00:00	11.6	50	2.9	2	315	northwest wind	954	11.2	0	20
2021-09-22 23:00:00	9.5	61	1.7	2	331	northwest wind	954	16.9	0	20
2021-09-23 00:00:00	9	62	0.5	1	323	northwest wind	954	19.9	0	20
2021-09-23 01:00:00	8.5	63	1.4	1	307	northwest wind	954	20.9	0	20
2021-09-23 02:00:00	8.1	61	1.1	1	329	northwest wind	954	29	0	20
2021-09-23 03:00:00	7.7	63	1.3	1	319	northwest wind	953	30	0	60
2021-09-23 04:00:00	7.1	65	1.7	2	295	northwest wind	953	29	0	90
2021-09-23 05:00:00	6.9	67	2.5	2	293	northwest wind	953	29.8	0	90
2021-09-23 06:00:00	6.4	71	1	1	315	northwest wind	953	30	0	90
2021-09-23 07:00:00	6.6	70	1.1	1	319	northwest wind	953	30	0	90
2021-09-23 08:00:00	6.4	69	1.6	2	335	northwest wind	954	30	0	90
2021-09-23 09:00:00	6.4	70	1.1	1	305	northwest wind	954	20.4	0	90
2021-09-23 10:00:00	8.2	66	2.3	2	326	northwest wind	955	20.9	0	60
2021-09-23 11:00:00	13.6	38	1.5	1	216	southwest wind	955	22.2	0	20
2021-09-23 12:00:00	16.4	30	0.5	1	153	southeast wind	955	28.1	0	20
2021-09-23 13:00:00	17.1	27	2.7	2	34	northeast wind	954	30	0	20
2021-09-23 14:00:00	17.5	25	1.1	1	134	southeast wind	954	30	0	20
2021-09-23 15:00:00	18	23	3.1	2	62	northeast wind	953	30	0	90
2021-09-23 16:00:00	18.4	23	1.4	1	37	northeast wind	953	30	0	90
2021-09-23 17:00:00	19.6	22	1.5	1	121	southeast wind	952	30	0	60
2021-09-23 18:00:00	19.4	24	1.4	1	140	southeast wind	952	30	0	20
2021-09-23 19:00:00	18.7	24	2.4	2	150	southeast wind	952	30	0	20
2021-09-23 20:00:00	17.1	26	1	1	145	southeast wind	952	30	0	20
2021-09-23 21:00:00	14	41	0.2	0	27	northeast wind	953	30	0	20
2021-09-23 22:00:00	11.6	48	3.2	2	298	northwest wind	953	30	0	20

2021-09-23 23:00:00	9.9	57	2.8	2	209	southwest wind	953	30	0	60
2021-09-24 00:00:00	9.2	59	0.6	1	295	northwest wind	953	30	0	60
2021-09-24 01:00:00	8.7	61	1.8	2	292	west wind	953	30	0	90
2021-09-24 02:00:00	7.8	66	3	2	335	northwest wind	953	30	0	90
2021-09-24 03:00:00	7.6	67	1.4	1	297	northwest wind	952	30	0	90
2021-09-24 04:00:00	7.3	66	0.1	0	206	southwest wind	952	30	0	90
2021-09-24 05:00:00	6.7	71	0.5	1	308	northwest wind	951	30	0	90
2021-09-24 06:00:00	6.9	71	0.1	0	317	northwest wind	951	30	0	90
2021-09-24 07:00:00	6.3	73	1	1	319	northwest wind	952	30	0	60
2021-09-24 08:00:00	5.7	75	1	1	318	northwest wind	952	30	0	20
2021-09-24 09:00:00	6.2	73	0.9	1	311	northwest wind	953	30	0	60
2021-09-24 10:00:00	9.1	61	1.7	2	336	northwest wind	953	30	0	60
2021-09-24 11:00:00	14.3	39	2.7	2	133	southeast wind	953	30	0	20
2021-09-24 12:00:00	16.4	33	0.9	1	117	southeast wind	954	30	0	20
2021-09-24 13:00:00	17.9	29	1.9	2	134	southeast wind	954	30	0	20
2021-09-24 14:00:00	18.6	28	1.6	2	148	southeast wind	955	30	0	20
2021-09-24 15:00:00	18.7	29	1	1	204	southwest wind	955	30	0	20
2021-09-24 16:00:00	18.2	26	0.3	1	215	southwest wind	956	30	0	20
2021-09-24 17:00:00	19.7	26	1.9	2	302	northwest wind	956	30	0	60
2021-09-24 18:00:00	18.3	24	4.4	3	54	northeast wind	957	12.1	0	60
2021-09-24 19:00:00	17.4	27	1.4	1	43	northeast wind	958	12.1	0	60
2021-09-24 20:00:00	16	31	0.2	0	135	southeast wind	959	12.1	0	20
2021-09-24 21:00:00	14.1	36	0	0	27	northeast wind	960	24.6	0	20
2021-09-24 22:00:00	10.6	51	2.2	2	212	southwest wind	961	20.7	0	20
2021-09-24 23:00:00	10.1	49	0.2	0	135	southeast wind	962	20.7	0	20
2021-09-25 00:00:00	11.1	42	2.3	2	118	southeast wind	962	26.2	0	20
2021-09-25 01:00:00	8.9	54	0.1	0	331	northwest wind	962	28.2	0	20
2021-09-25 02:00:00	6.5	62	3.1	2	313	northwest wind	962	28.2	0	20
2021-09-25 03:00:00	5.2	71	3	2	329	northwest wind	961	27.9	0	20
2021-09-25 04:00:00	4.3	76	3.1	2	294	northwest wind	961	26.1	0	20
2021-09-25 05:00:00	3.7	75	0.5	1	303	northwest wind	961	24.4	0	20
2021-09-25 06:00:00	3.2	77	0.1	0	326	northwest wind	962	25	0	20
2021-09-25 07:00:00	2.8	78	2.2	2	328	northwest wind	962	27	0	20
2021-09-25 08:00:00	2.5	80	1.9	2	315	northwest wind	963	26.9	0	20
2021-09-25 09:00:00	3	78	0.8	1	310	northwest wind	964	22.2	0	20
2021-09-25 10:00:00	6.6	62	0.8	1	302	northwest wind	964	16	0	20
2021-09-25 11:00:00	10.3	46	0.3	1	207	southwest wind	964	23.3	0	20
2021-09-25 12:00:00	12.2	39	1.7	2	116	southeast wind	964	12.3	0	20

2021-09-25 13:00:00	12.9	35	2.6	2	140	southeast wind	963	24.2	0	20
2021-09-25 14:00:00	14.2	33	1.5	1	129	southeast wind	963	27.9	0	20
2021-09-25 15:00:00	15.2	31	1.5	1	154	southeast wind	962	29.7	0	20
2021-09-25 16:00:00	15.8	30	0.6	1	155	southeast wind	962	23.8	0	20
2021-09-25 17:00:00	15.7	30	2	2	125	southeast wind	961	30	0	20
2021-09-25 18:00:00	15.7	31	2.3	2	42	northeast wind	961	18.6	0	20
2021-09-25 19:00:00	15.4	30	3.2	2	117	southeast wind	961	17.1	0	20
2021-09-25 20:00:00	13.8	33	2.9	2	122	southeast wind	961	29.7	0	20
2021-09-25 21:00:00	11.2	49	2.4	2	152	southeast wind	961	25.2	0	20
2021-09-25 22:00:00	8.7	59	1.3	1	319	northwest wind	961	17.4	0	20
2021-09-25 23:00:00	6.9	65	0.7	1	334	northwest wind	961	12.1	0	20
2021-09-26 00:00:00	5.8	66	0.9	1	310	northwest wind	961	14.3	0	20
2021-09-26 01:00:00	4.9	69	2.3	2	326	northwest wind	960	18.1	0	20
2021-09-26 02:00:00	4.2	74	3	2	300	northwest wind	960	18.1	0	20
2021-09-26 03:00:00	4.1	74	0.1	0	298	northwest wind	960	23.6	0	20
2021-09-26 04:00:00	3.6	75	2.3	2	309	northwest wind	959	23	0	20
2021-09-26 05:00:00	3.1	76	1.9	2	295	northwest wind	959	26.3	0	20
2021-09-26 06:00:00	2.8	77	0.6	1	326	northwest wind	958	27	0	20
2021-09-26 07:00:00	2.4	77	0.2	0	299	northwest wind	958	27	0	20
2021-09-26 08:00:00	2	79	1.3	1	293	northwest wind	958	26.5	0	20
2021-09-26 09:00:00	2.5	77	2.5	2	329	northwest wind	958	18.1	0	20
2021-09-26 10:00:00	5.3	67	3	2	284	west wind	958	22.4	0	20
2021-09-26 11:00:00	10.2	49	3	2	210	southwest wind	957	22.3	0	20
2021-09-26 12:00:00	13	36	0.3	1	325	northwest wind	957	29.4	0	20
2021-09-26 13:00:00	14.8	32	2.8	2	237	southwest wind	956	29.9	0	20
2021-09-26 14:00:00	16.2	32	2.5	2	123	southeast wind	955	21.9	0	20
2021-09-26 15:00:00	16.9	31	0.5	1	210	southwest wind	954	27	0	20
2021-09-26 16:00:00	17	29	0.6	1	116	southeast wind	954	27	0	20
2021-09-26 17:00:00	17.5	27	3.2	2	53	northeast wind	954	27	0	90
2021-09-26 18:00:00	17.7	28	0.1	0	140	southeast wind	953	27	0	90
2021-09-26 19:00:00	17.3	28	1.9	2	145	southeast wind	953	27	0	90
2021-09-26 20:00:00	14.6	42	0.1	0	151	southeast wind	953	27	0	60
2021-09-26 21:00:00	11.4	56	2.9	2	332	northwest wind	953	27	0	60
2021-09-26 22:00:00	9.4	66	2.4	2	318	northwest wind	953	27	0	20
2021-09-26 23:00:00	8.2	63	1.2	1	323	northwest wind	953	15.2	0	20
2021-09-27 00:00:00	8	63	1	1	315	northwest wind	952	17	0	90
2021-09-27 01:00:00	7.8	66	2	2	319	northwest wind	952	13.4	0	60
2021-09-27 02:00:00	6.9	70	3	2	334	northwest wind	952	14.1	0	20

2021-09-27 03:00:00	5.9	76	1.9	2	328	northwest wind	951	18.9	0	20
2021-09-27 04:00:00	5.4	77	3	2	333	northwest wind	951	21.4	0	20
2021-09-27 05:00:00	5.1	76	1.8	2	298	northwest wind	951	22.2	0	20
2021-09-27 06:00:00	4.9	76	2	2	312	northwest wind	950	21.5	0	20
2021-09-27 07:00:00	5	74	1.5	1	332	northwest wind	951	24.7	0	60
2021-09-27 08:00:00	4.6	77	1.2	1	299	northwest wind	951	23.6	0	90
2021-09-27 09:00:00	5.3	71	0.1	0	315	northwest wind	951	19.8	0	90
2021-09-27 10:00:00	7.6	68	1.6	2	297	northwest wind	951	19.8	0	90
2021-09-27 11:00:00	12	53	2.6	2	332	northwest wind	951	19.8	0	60
2021-09-27 12:00:00	11	35	0.1	0	134	southeast wind	951	19.8	0	20
2021-09-27 13:00:00	17.5	30	2.7	2	152	southeast wind	951	19.8	0	20
2021-09-27 14:00:00	18.1	28	2.2	2	120	southeast wind	950	19.8	0	20
2021-09-27 15:00:00	19.3	28	1	1	229	southwest wind	949	19.8	0	20
2021-09-27 16:00:00	19.8	26	1.9	2	35	northeast wind	949	19.8	0	20
2021-09-27 17:00:00	20.5	26	1.5	1	139	southeast wind	948	19.8	0	20
2021-09-27 18:00:00	19.7	25	1.9	2	231	southwest wind	949	19.8	0	60
2021-09-27 19:00:00	19.3	30	1.2	1	223	southwest wind	949	18.4	0	90
2021-09-27 20:00:00	18.3	29	0.7	1	154	southeast wind	950	18.4	0	20
2021-09-27 21:00:00	15.3	39	1.7	2	233	southwest wind	951	15.8	0	20
2021-09-27 22:00:00	13.4	45	0.5	1	311	northwest wind	952	9.1	0	60
2021-09-27 23:00:00	11.8	54	3	2	314	northwest wind	953	10.3	0	20
2021-09-28 00:00:00	10.5	57	2	2	267	west wind	953	15	0	20
2021-09-28 01:00:00	10	60	1.8	2	329	northwest wind	953	15.8	0	20
2021-09-28 02:00:00	9.3	62	2.9	2	329	northwest wind	954	15.7	0	20
2021-09-28 03:00:00	8.7	66	3.1	2	319	northwest wind	954	17.5	0	20
2021-09-28 04:00:00	8.3	66	0.2	0	328	northwest wind	954	18.8	0	20
2021-09-28 05:00:00	8.2	64	1.2	1	321	northwest wind	954	20	0	20
2021-09-28 06:00:00	8.1	64	0	0	299	northwest wind	954	20.1	0	20
2021-09-28 07:00:00	7.6	66	2.8	2	293	northwest wind	955	18.8	0	20
2021-09-28 08:00:00	8.1	63	2.5	2	305	northwest wind	955	18.8	0	20
2021-09-28 09:00:00	7.7	65	1.9	2	330	northwest wind	955	17.3	0	20
2021-09-28 10:00:00	11.3	54	1.6	2	327	northwest wind	955	14.9	0	20
2021-09-28 11:00:00	15.9	44	1.5	1	37	northeast wind	955	14.5	0	60
2021-09-28 12:00:00	17.9	41	2.1	2	126	southeast wind	955	17	0	60
2021-09-28 13:00:00	19.4	39	1.9	2	142	southeast wind	954	16	0	20
2021-09-28 14:00:00	20.6	33	2.7	2	151	southeast wind	954	16	0	20
2021-09-28 15:00:00	20.8	31	0.6	1	218	southwest wind	953	16	0	20
2021-09-28 16:00:00	21.6	31	4.4	3	147	southeast wind	953	16	0	20

2021-09-28 17:00:00	21.5	30	2.4	2	128	southeast wind	952	16	0	20
2021-09-28 18:00:00	21.8	32	2.1	2	233	southwest wind	952	16	0	60
2021-09-28 19:00:00	20.9	32	0.3	1	133	southeast wind	952	16	0	20
2021-09-28 20:00:00	18.8	42	0.5	1	218	southwest wind	952	16	0	20
2021-09-28 21:00:00	15.2	55	0.4	1	304	northwest wind	953	10.2	0	60
2021-09-28 22:00:00	13.2	61	1.1	1	293	northwest wind	953	7	0	60
2021-09-28 23:00:00	11.9	64	1.4	1	293	northwest wind	952	10.7	0	20
2021-09-29 00:00:00	11	68	1.5	1	326	northwest wind	952	10	0	20
2021-09-29 01:00:00	10.6	67	2.1	2	302	northwest wind	952	11.9	0	60
2021-09-29 02:00:00	11	62	2	2	332	northwest wind	951	11.9	0	90
2021-09-29 03:00:00	10.8	62	2.1	2	308	northwest wind	951	11.9	0	20
2021-09-29 04:00:00	10.1	64	0.4	1	337	northwest wind	951	11.9	0	20
2021-09-29 05:00:00	8.8	70	1.2	1	213	southwest wind	950	11.9	0	60
2021-09-29 06:00:00	8.6	70	1.9	2	314	northwest wind	950	11.9	0	60
2021-09-29 07:00:00	8.5	71	1.1	1	300	northwest wind	951	11.9	0	90
2021-09-29 08:00:00	8.3	71	2	2	322	northwest wind	952	11.9	0	90
2021-09-29 09:00:00	8.4	71	0.5	1	305	northwest wind	952	11.9	0	60
2021-09-29 10:00:00	11.4	61	3.2	2	294	northwest wind	953	11.9	0	60
2021-09-29 11:00:00	14.4	53	3	2	326	northwest wind	953	11.9	0	60
2021-09-29 12:00:00	19.2	37	0.4	1	136	southeast wind	953	11.9	0	60
2021-09-29 13:00:00	20.1	34	3.2	2	134	southeast wind	952	11.9	0	20
2021-09-29 14:00:00	21.3	32	3	2	145	southeast wind	951	11.9	0	20
2021-09-29 15:00:00	21.6	31	1.8	2	142	southeast wind	951	11.9	0	60
2021-09-29 16:00:00	22.5	29	2.9	2	28	northeast wind	951	14.3	0	60
2021-09-29 17:00:00	22.6	31	2.2	2	134	southeast wind	951	17.5	0	60
2021-09-29 18:00:00	22.3	30	2.9	2	127	southeast wind	951	15.9	0	60
2021-09-29 19:00:00	21.4	31	2.1	2	124	southeast wind	952	16	0	60
2021-09-29 20:00:00	19.3	39	1.1	1	129	southeast wind	952	13.3	0	60
2021-09-29 21:00:00	16.2	49	3	2	324	northwest wind	953	9.3	0	60
2021-09-29 22:00:00	14	59	1.4	1	300	northwest wind	953	5.2	0	20
2021-09-29 23:00:00	12.6	61	2.3	2	336	northwest wind	953	10.2	0	20
2021-09-30 00:00:00	11	71	2.2	2	302	northwest wind	953	12.5	0	60
2021-09-30 01:00:00	11.1	67	1.6	2	333	northwest wind	953	8.5	0	60
2021-09-30 02:00:00	11.4	62	0.7	1	305	northwest wind	953	16.6	0	60
2021-09-30 03:00:00	10.9	65	0.7	1	300	northwest wind	953	17.6	0	60
2021-09-30 04:00:00	10.8	65	2.7	2	311	northwest wind	953	17.5	0	20
2021-09-30 05:00:00	10.9	66	0.2	0	335	northwest wind	953	18.1	0	60
2021-09-30 06:00:00	10.3	68	3	2	322	northwest wind	953	18.3	0	20

2021-09-30 07:00:00	9.4	75	2.2	2	306	northwest wind	953	18.3	0	60
2021-09-30 08:00:00	9.1	74	0.4	1	305	northwest wind	954	17.5	0	20
2021-09-30 09:00:00	9.2	74	1.8	2	208	southwest wind	954	9.2	0	60
2021-09-30 10:00:00	11.9	67	1.3	1	314	northwest wind	955	14.6	0	20
2021-09-30 11:00:00	16.5	50	1.9	2	312	northwest wind	955	16.6	0	20
2021-09-30 12:00:00	18.8	40	0.9	1	129	southeast wind	954	16.5	0	20
2021-09-30 13:00:00	20.6	37	3.1	2	151	southeast wind	954	18.3	0	20
2021-09-30 14:00:00	21.3	33	1.4	1	23	northeast wind	953	17.1	0	20
2021-09-30 15:00:00	22.3	32	3.2	2	153	southeast wind	952	21.2	0	20
2021-09-30 16:00:00	23	33	4.2	3	182	south wind	952	21.2	0	20
2021-09-30 17:00:00	23.2	30	4.9	3	147	southeast wind	952	20	0	20
2021-09-30 18:00:00	22.6	28	0.4	1	128	southeast wind	952	19.8	0	20
2021-09-30 19:00:00	21.8	31	2.4	2	124	southeast wind	951	23	0	20
2021-09-30 20:00:00	18.5	43	1.9	2	313	northwest wind	951	25.6	0	20
2021-09-30 21:00:00	15.5	55	3.1	2	243	southwest wind	951	11.1	0	20
2021-09-30 22:00:00	15.3	54	2.7	2	304	northwest wind	951	13.1	0	90
2021-09-30 23:00:00	15.2	52	0.7	1	294	northwest wind	952	13.7	0	60
2021-10-01 00:00:00	14.6	56	2.4	2	224	southwest wind	952	14.1	0	90
2021-10-01 01:00:00	14.4	57	1.6	2	333	northwest wind	951	18.1	0	90
2021-10-01 02:00:00	14	58	1.8	2	294	northwest wind	950	20.6	0	60
2021-10-01 03:00:00	12.6	63	1.9	2	327	northwest wind	949	20.7	0	20
2021-10-01 04:00:00	11.6	67	1	1	333	northwest wind	949	22.4	0	20
2021-10-01 05:00:00	12.7	59	1.2	1	297	northwest wind	949	24	0	20
2021-10-01 06:00:00	11.2	66	2.9	2	204	southwest wind	948	24.3	0	60
2021-10-01 07:00:00	12.2	64	1.5	1	319	northwest wind	949	24.6	0	90
2021-10-01 08:00:00	12	69	0.2	0	304	northwest wind	949	22.3	0	90
2021-10-01 09:00:00	13.7	63	0.6	1	124	southeast wind	950	16.6	0	60
2021-10-01 10:00:00	13.5	73	5.1	3	217	southwest wind	951	18.5	0	60
2021-10-01 11:00:00	13.5	70	1.1	1	224	southwest wind	951	30	0	90
2021-10-01 12:00:00	13.7	61	5.1	3	132	southeast wind	952	25.6	0	60
2021-10-01 13:00:00	14.6	53	1.7	2	115	southeast wind	952	19.9	0	90
2021-10-01 14:00:00	15	50	4.3	3	215	southwest wind	953	21.7	0	60
2021-10-01 15:00:00	14.9	49	0.1	0	137	southeast wind	953	30	0	90
2021-10-01 16:00:00	14.7	50	0.4	1	130	southeast wind	953	22	0	90
2021-10-01 17:00:00	14	52	4.1	3	148	southeast wind	954	26.2	0	90
2021-10-01 18:00:00	13.1	54	4.2	3	138	southeast wind	954	29	0	60
2021-10-01 19:00:00	12.4	54	4.1	3	214	southwest wind	955	27.2	0	20
2021-10-01 20:00:00	11.2	58	4	3	150	southeast wind	955	30	0	60

2021-10-01 21:00:00	10.5	58	5	3	151	southeast wind	956	30	0	60
2021-10-01 22:00:00	10	56	2.8	2	138	southeast wind	957	30	0	60
2021-10-01 23:00:00	10	54	1.6	2	155	southeast wind	957	30	0	20
2021-10-02 00:00:00	9.5	57	2.5	2	116	southeast wind	957	30	0	20
2021-10-02 01:00:00	9.4	58	1	1	128	southeast wind	957	30	0	60
2021-10-02 02:00:00	9.1	58	2.6	2	126	southeast wind	957	30	0	60
2021-10-02 03:00:00	8.9	58	1.9	2	134	southeast wind	956	30	0	90
2021-10-02 04:00:00	8.6	60	2.2	2	157	southeast wind	956	30	0	60
2021-10-02 05:00:00	8.3	60	2.5	2	154	southeast wind	956	30	0	20
2021-10-02 06:00:00	8.2	65	0.6	1	236	southwest wind	956	30	0	90
2021-10-02 07:00:00	7.4	74	0.6	1	123	southeast wind	957	27.4	0	60
2021-10-02 08:00:00	6.7	84	0.4	1	36	northeast wind	958	15.8	0.5	90
2021-10-02 09:00:00	6.5	90	1.3	1	28	northeast wind	958	15.6	0.6	90
2021-10-02 10:00:00	6.5	91	3.1	2	43	northeast wind	959	11.4	0.8	90
2021-10-02 11:00:00	6.5	93	2.3	2	31	northeast wind	960	7.1	1.2	90
2021-10-02 12:00:00	6.6	91	2.2	2	157	southeast wind	961	7.4	1.7	90
2021-10-02 13:00:00	6.6	90	0.5	1	47	northeast wind	961	28	0.8	90
2021-10-02 14:00:00	6.4	85	2.6	2	132	southeast wind	962	30	0	90
2021-10-02 15:00:00	6.9	81	3	2	148	southeast wind	962	30	0	90
2021-10-02 16:00:00	7.6	76	2.5	2	156	southeast wind	962	30	0	60
2021-10-02 17:00:00	7.5	81	1.4	1	52	northeast wind	963	30	0	90
2021-10-02 18:00:00	7.2	83	0.7	1	31	northeast wind	963	30	0	60
2021-10-02 19:00:00	7.2	83	0.6	1	316	northwest wind	964	20.5	0	90
2021-10-02 20:00:00	7.4	83	1.3	1	326	northwest wind	965	20.8	0	60
2021-10-02 21:00:00	7.2	81	0.3	1	320	northwest wind	965	26.5	0	90
2021-10-02 22:00:00	7	87	1.3	1	245	southwest wind	966	28.4	0	90
2021-10-02 23:00:00	6.8	89	0.3	1	228	southwest wind	966	15.9	0	90
2021-10-03 00:00:00	6.8	88	0.8	1	219	southwest wind	966	24.5	0	60
2021-10-03 01:00:00	6.6	80	0.6	1	120	southeast wind	966	22	0	60
2021-10-03 02:00:00	6	80	2.2	2	244	southwest wind	966	30	0	60
2021-10-03 03:00:00	6	80	2	2	299	northwest wind	966	30	0	60
2021-10-03 04:00:00	6	78	2.7	2	134	southeast wind	966	30	0	60
2021-10-03 05:00:00	5.9	77	2.1	2	0	north wind	966	30	0	90
2021-10-03 06:00:00	6.2	74	0.6	1	31	northeast wind	966	30	0	60
2021-10-03 07:00:00	6	74	3.2	2	41	northeast wind	966	30	0	60
2021-10-03 08:00:00	5.3	80	3.2	2	29	northeast wind	966	25.9	0	20
2021-10-03 09:00:00	6	72	0.2	0	51	northeast wind	966	28.5	0	20
2021-10-03 10:00:00	8.3	60	0	0	331	northwest wind	966	28.4	0	20

2021-10-03 11:00:00	9.3	51	0.9	1	120	southeast wind	966	30	0	20
2021-10-03 12:00:00	9.5	52	2.9	2	145	southeast wind	966	30	0	20
2021-10-03 13:00:00	10.8	47	3	2	125	southeast wind	965	30	0	20
2021-10-03 14:00:00	11.1	43	0.5	1	240	southwest wind	965	30	0	20
2021-10-03 15:00:00	11.5	42	2	2	42	northeast wind	964	30	0	20
2021-10-03 16:00:00	10.8	44	2.6	2	123	southeast wind	963	30	0	20
2021-10-03 17:00:00	12.2	45	3.2	2	135	southeast wind	963	30	0	20
2021-10-03 18:00:00	11.7	44	1.3	1	129	southeast wind	963	30	0	20
2021-10-03 19:00:00	11.1	43	1	1	156	southeast wind	963	30	0	20
2021-10-03 20:00:00	9.9	51	1.6	2	113	southeast wind	963	30	0	20
2021-10-03 21:00:00	8.8	57	2.9	2	75	east wind	963	30	0	20
2021-10-03 22:00:00	6.3	74	2.4	2	307	northwest wind	964	20.7	0	20
2021-10-03 23:00:00	4.7	81	2.5	2	311	northwest wind	964	8.6	0	20
2021-10-04 00:00:00	5.1	78	0.6	1	299	northwest wind	963	14.7	0	20
2021-10-04 01:00:00	5.5	78	0.9	1	316	northwest wind	963	18.6	0	20
2021-10-04 02:00:00	6.2	76	3.2	2	295	northwest wind	963	22.4	0	20
2021-10-04 03:00:00	5.9	82	1	1	275	west wind	963	21	0	20
2021-10-04 04:00:00	6.1	83	0.7	1	307	northwest wind	962	20.2	0	20
2021-10-04 05:00:00	5.2	84	3.2	2	312	northwest wind	963	22	0	60
2021-10-04 06:00:00	4.3	85	0.7	1	204	southwest wind	962	21.1	0	90
2021-10-04 07:00:00	4.4	86	2.1	2	323	northwest wind	962	18.4	0	90
2021-10-04 08:00:00	4.9	82	2.7	2	293	northwest wind	962	19.8	0	90
2021-10-04 09:00:00	5.4	81	0.3	1	38	northeast wind	962	15.6	0	90
2021-10-04 10:00:00	6.2	79	3.1	2	242	southwest wind	963	17.2	0	90
2021-10-04 11:00:00	7.7	56	0.5	1	121	southeast wind	963	15.6	0	60
2021-10-04 12:00:00	8.4	48	2.4	2	49	northeast wind	963	29.5	0	90
2021-10-04 13:00:00	8.8	47	0.1	0	59	northeast wind	962	30	0	90
2021-10-04 14:00:00	9.2	47	2.1	2	135	southeast wind	962	30	0	90
2021-10-04 15:00:00	9.9	45	2.8	2	36	northeast wind	961	30	0	90
2021-10-04 16:00:00	10.8	44	0.5	1	126	southeast wind	960	27	0	60
2021-10-04 17:00:00	11.3	45	1.5	1	44	northeast wind	960	30	0	60
2021-10-04 18:00:00	11.3	45	2.9	2	151	southeast wind	961	28.4	0	60
2021-10-04 19:00:00	10.9	49	1.7	2	122	southeast wind	961	27.4	0	60
2021-10-04 20:00:00	9.7	54	0.2	0	133	southeast wind	961	25.1	0	20
2021-10-04 21:00:00	6.8	73	1.3	1	30	northeast wind	962	10.6	0	20
2021-10-04 22:00:00	5.3	80	2.3	2	313	northwest wind	962	9	0	20
2021-10-04 23:00:00	3.9	83	0.1	0	319	northwest wind	962	10.5	0	20
2021-10-05 00:00:00	3.5	84	0.6	1	319	northwest wind	962	10.7	0	20

2021-10-05 01:00:00	2.7	86	2.1	2	303	northwest wind	961	10.7	0	20
2021-10-05 02:00:00	2.5	87	1.7	2	298	northwest wind	961	10.7	0	20
2021-10-05 03:00:00	2.1	88	1.1	1	303	northwest wind	961	13	0	20
2021-10-05 04:00:00	1.9	87	0.6	1	337	northwest wind	960	13.3	0	20
2021-10-05 05:00:00	1.1	89	2.9	2	295	northwest wind	960	11.7	0	20
2021-10-05 06:00:00	0.5	90	2.7	2	296	northwest wind	959	10.9	0	20
2021-10-05 07:00:00	0.4	89	2.8	2	332	northwest wind	959	11.5	0	20
2021-10-05 08:00:00	0.2	89	1.1	1	330	northwest wind	959	9.8	0	20
2021-10-05 09:00:00	0.5	89	3.2	2	306	northwest wind	959	9	0	20
2021-10-05 10:00:00	3	82	0.9	1	295	northwest wind	959	11.4	0	20
2021-10-05 11:00:00	6.6	72	3	2	335	northwest wind	959	13.1	0	20
2021-10-05 12:00:00	9.9	54	1.6	2	113	southeast wind	959	16.1	0	20
2021-10-05 13:00:00	10.7	43	2.7	2	50	northeast wind	958	19.3	0	20
2021-10-05 14:00:00	11.7	42	1	1	310	northwest wind	957	24.8	0	60
2021-10-05 15:00:00	11.9	40	0.7	1	129	southeast wind	956	21.2	0	60
2021-10-05 16:00:00	12.6	38	1.2	1	134	southeast wind	956	18.7	0	60
2021-10-05 17:00:00	12.7	35	1.6	2	43	northeast wind	956	20.6	0	60
2021-10-05 18:00:00	12.3	37	1.8	2	40	northeast wind	956	20.6	0	60
2021-10-05 19:00:00	11.7	37	2.7	2	153	southeast wind	956	11.6	0	60
2021-10-05 20:00:00	10.3	46	1.5	1	151	southeast wind	956	11.9	0	60
2021-10-05 21:00:00	8.3	63	0	0	311	northwest wind	957	8.1	0	90
2021-10-05 22:00:00	7.6	63	2.8	2	294	northwest wind	957	6.9	0	90
2021-10-05 23:00:00	6.6	75	0.1	0	233	southwest wind	957	8.8	0	60
2021-10-06 00:00:00	6.8	69	0.9	1	314	northwest wind	957	8.8	0	90
2021-10-06 01:00:00	6.8	70	1.7	2	310	northwest wind	956	8.8	0	90
2021-10-06 02:00:00	6.8	74	0.4	1	320	northwest wind	956	8.8	0	90
2021-10-06 03:00:00	6.5	76	0.5	1	318	northwest wind	956	8.8	0	90
2021-10-06 04:00:00	6.2	78	1.1	1	324	northwest wind	956	8.8	0	60
2021-10-06 05:00:00	6	81	2.6	2	317	northwest wind	956	8.8	0	90
2021-10-06 06:00:00	5.8	84	0.7	1	220	southwest wind	956	8.3	0	90
2021-10-06 07:00:00	5.8	82	0.1	0	228	southwest wind	956	9	0	90
2021-10-06 08:00:00	5.6	84	1.1	1	28	northeast wind	957	8.7	0	90
2021-10-06 09:00:00	6.8	76	0.6	1	148	southeast wind	958	7.4	0	60
2021-10-06 10:00:00	7	67	1.8	2	125	southeast wind	959	29.7	0	60
2021-10-06 11:00:00	7	63	3.4	3	121	southeast wind	960	29.7	0	90
2021-10-06 12:00:00	7.6	57	3	2	123	southeast wind	960	29.7	0	60
2021-10-06 13:00:00	8.1	55	2.4	2	147	southeast wind	960	21.9	0	60
2021-10-06 14:00:00	8	54	0.5	1	150	southeast wind	961	21	0	60

2021-10-06 15:00:00	8.5	52	0.9	1	136	southeast wind	961	22.5	0	60
2021-10-06 16:00:00	8.1	51	0.8	1	195	south wind	961	20.8	0	90
2021-10-06 17:00:00	8.9	48	3.2	2	234	southwest wind	962	15.4	0	90
2021-10-06 18:00:00	7.3	49	3.6	3	127	southeast wind	963	18.3	0	60
2021-10-06 19:00:00	6.2	50	4.8	3	153	southeast wind	964	9.7	0	60
2021-10-06 20:00:00	5.6	52	1.9	2	128	southeast wind	965	17.1	0	60
2021-10-06 21:00:00	5.4	51	0.3	1	311	northwest wind	967	29.1	0	60
2021-10-06 22:00:00	5	52	0	0	150	southeast wind	967	30	0	60
2021-10-06 23:00:00	4.7	55	2.2	2	152	southeast wind	967	30	0	60
2021-10-07 00:00:00	4.2	59	2.3	2	207	southwest wind	968	30	0	20
2021-10-07 01:00:00	3.7	60	0	0	29	northeast wind	967	30	0	20
2021-10-07 02:00:00	2.8	62	2.4	2	0	north wind	967	30	0	20
2021-10-07 03:00:00	2.6	67	2.1	2	41	northeast wind	967	30	0	20
2021-10-07 04:00:00	2.9	68	0.9	1	332	northwest wind	966	30	0	60
2021-10-07 05:00:00	3.3	64	2	2	142	southeast wind	966	30	0	90
2021-10-07 06:00:00	3.1	67	1.5	1	0	north wind	966	30	0	60
2021-10-07 07:00:00	3.2	59	2.9	2	63	northeast wind	966	30	0	90
2021-10-07 08:00:00	2.9	62	0.9	1	37	northeast wind	967	30	0	90
2021-10-07 09:00:00	2.5	66	1.4	1	42	northeast wind	967	29.3	0	60
2021-10-07 10:00:00	4.8	51	2	2	150	southeast wind	967	18	0	60
2021-10-07 11:00:00	5.8	48	0.4	1	242	southwest wind	968	30	0	60
2021-10-07 12:00:00	6.2	48	4.5	3	218	southwest wind	967	30	0	60
2021-10-07 13:00:00	6.9	45	0.9	1	239	southwest wind	967	30	0	60
2021-10-07 14:00:00	6.9	44	2.9	2	139	southeast wind	967	30	0	60
2021-10-07 15:00:00	7.1	45	1.8	2	42	northeast wind	966	30	0	60
2021-10-07 16:00:00	6.8	47	1.1	1	25	northeast wind	966	30	0	90
2021-10-07 17:00:00	6.6	51	2.3	2	57	northeast wind	967	28.4	0	90
2021-10-07 18:00:00	6.9	48	0.2	0	26	northeast wind	967	30	0	90
2021-10-07 19:00:00	6.7	50	0.2	0	131	southeast wind	968	30	0	90
2021-10-07 20:00:00	6	52	2.6	2	227	southwest wind	968	26.8	0	90
2021-10-07 21:00:00	5.4	58	0.9	1	204	southwest wind	970	30	0	90
2021-10-07 22:00:00	4.3	70	0	0	332	northwest wind	971	30	0	90
2021-10-07 23:00:00	3.2	86	3	2	302	northwest wind	972	14.4	0	90
2021-10-08 00:00:00	2.9	87	0.8	1	335	northwest wind	973	22	0	90
2021-10-08 01:00:00	2.2	89	0.7	1	330	northwest wind	973	22.3	0.3	90
2021-10-08 02:00:00	2.5	89	2.7	2	209	southwest wind	973	25.5	0	90
2021-10-08 03:00:00	2.4	90	0.9	1	147	southeast wind	973	30	0	60
2021-10-08 04:00:00	1.6	87	1.3	1	11	north wind	973	29.1	0	20

2021-10-08 05:00:00	0.7	91	2.5	2	337	northwest wind	973	30	0	20
2021-10-08 06:00:00	-0.4	92	1.9	2	317	northwest wind	972	9.1	0	20
2021-10-08 07:00:00	-1.5	93	0.1	0	0	north wind	972	20.1	0	20
2021-10-08 08:00:00	-2	95	2.2	2	313	northwest wind	972	10.6	0	20
2021-10-08 09:00:00	-1.5	95	1.8	2	309	northwest wind	973	7.7	0	20
2021-10-08 10:00:00	0.5	86	0.2	0	277	west wind	973	7.7	0	20
2021-10-08 11:00:00	3.2	73	1.7	2	236	southwest wind	973	7.7	0	20
2021-10-08 12:00:00	5.5	61	2.1	2	309	northwest wind	972	7.7	0	20
2021-10-08 13:00:00	7.2	51	0.3	1	332	northwest wind	972	7.7	0	20
2021-10-08 14:00:00	8.5	45	0.5	1	219	southwest wind	971	7.7	0	20
2021-10-08 15:00:00	9	40	1.6	2	232	southwest wind	970	7.7	0	20
2021-10-08 16:00:00	9	40	1.6	2	232	southwest wind	970	7.7	0	20
2021-10-08 17:00:00	10.6	31	2.4	2	154	southeast wind	969	7.7	0	20
2021-10-08 18:00:00	10.4	33	1.3	1	139	southeast wind	969	7.7	0	20
2021-10-08 19:00:00	9.6	36	3.1	2	143	southeast wind	969	7.7	0	20
2021-10-08 20:00:00	8.1	42	0.5	1	117	southeast wind	969	7.7	0	20
2021-10-08 21:00:00	6.9	45	0.6	1	152	southeast wind	970	7.7	0	20
2021-10-08 22:00:00	4.5	57	0.3	1	36	northeast wind	971	7.7	0	20
2021-10-08 23:00:00	3.1	64	1	1	296	northwest wind	971	7.7	0	20
2021-10-09 00:00:00	1.3	76	0.3	1	328	northwest wind	971	7.7	0	20
2021-10-09 01:00:00	-0.1	79	1.3	1	296	northwest wind	971	7.7	0	20
2021-10-09 02:00:00	-0.4	80	0.5	1	314	northwest wind	971	7.7	0	20
2021-10-09 03:00:00	-0.7	82	2.9	2	297	northwest wind	970	7.7	0	20
2021-10-09 04:00:00	-0.6	81	2.4	2	313	northwest wind	970	7.7	0	20
2021-10-09 05:00:00	-1.2	81	1.9	2	311	northwest wind	970	7.7	0	20
2021-10-09 06:00:00	-1.6	82	2.6	2	296	northwest wind	969	7.7	0	20
2021-10-09 07:00:00	-2.1	84	0.5	1	299	northwest wind	969	7.7	0	20
2021-10-09 08:00:00	-2.4	85	1.1	1	305	northwest wind	969	7.7	0	20
2021-10-09 09:00:00	-2.4	84	0.4	1	335	northwest wind	970	7.7	0	20
2021-10-09 10:00:00	0.1	76	3.2	2	332	northwest wind	970	7.7	0	20
2021-10-09 11:00:00	4.5	54	0.7	1	36	northeast wind	970	7.7	0	20
2021-10-09 12:00:00	6.5	44	3	2	154	southeast wind	969	7.7	0	20
2021-10-09 13:00:00	7.1	43	1	1	25	northeast wind	969	7.7	0	20
2021-10-09 14:00:00	8.3	39	1.1	1	244	southwest wind	968	7.7	0	20
2021-10-09 15:00:00	9.2	38	0.3	1	207	southwest wind	966	7.7	0	20
2021-10-09 16:00:00	9.6	36	0.7	1	24	northeast wind	966	7.7	0	20
2021-10-09 17:00:00	9.8	36	0.7	1	42	northeast wind	965	7.7	0	20
2021-10-09 18:00:00	9.3	36	2.6	2	151	southeast wind	965	7.7	0	20

2021-10-09 19:00:00	8.7	38	1.3	1	145	southeast wind	965	7.7	0	20
2021-10-09 20:00:00	6.8	49	0.8	1	30	northeast wind	965	7.7	0	20
2021-10-09 21:00:00	4.4	57	1.8	2	36	northeast wind	965	7.7	0	20
2021-10-09 22:00:00	2.8	68	1.7	2	297	northwest wind	965	7.7	0	20
2021-10-09 23:00:00	1.2	72	0.1	0	307	northwest wind	965	7.7	0	20
2021-10-10 00:00:00	0.4	75	1.1	1	322	northwest wind	965	7.7	0	20
2021-10-10 01:00:00	-0.3	76	2.5	2	325	northwest wind	965	7.7	0	20
2021-10-10 02:00:00	-0.5	79	0.3	1	304	northwest wind	964	7.7	0	20
2021-10-10 03:00:00	-0.7	79	2	2	298	northwest wind	964	7.7	0	20
2021-10-10 04:00:00	-1	80	2.3	2	329	northwest wind	963	7.7	0	20
2021-10-10 05:00:00	-1	81	2.2	2	337	northwest wind	963	7.7	0	20
2021-10-10 06:00:00	-1.3	80	0.4	1	204	southwest wind	963	7.7	0	20
2021-10-10 07:00:00	-1.4	81	0.5	1	314	northwest wind	962	7.7	0	20
2021-10-10 08:00:00	-1.4	80	0.2	0	325	northwest wind	962	7.7	0	20
2021-10-10 09:00:00	-1.2	79	1.5	1	304	northwest wind	962	7.7	0	20
2021-10-10 10:00:00	0.9	73	0.7	1	319	northwest wind	962	7.7	0	20
2021-10-10 11:00:00	4.1	61	0.8	1	294	northwest wind	962	7.7	0	20
2021-10-10 12:00:00	7.2	42	0.2	0	242	southwest wind	961	7.7	0	20
2021-10-10 13:00:00	9.3	38	3	2	336	northwest wind	961	7.7	0	20
2021-10-10 14:00:00	9.5	35	0.1	0	117	southeast wind	960	7.7	0	20
2021-10-10 15:00:00	10.6	35	2.1	2	128	southeast wind	959	7.7	0	20
2021-10-10 16:00:00	10.5	35	1.5	1	121	southeast wind	959	7.7	0	60
2021-10-10 17:00:00	10.1	35	2.6	2	148	southeast wind	959	7.7	0	60
2021-10-10 18:00:00	10	36	1.5	1	33	northeast wind	959	7.7	0	60
2021-10-10 19:00:00	9.5	39	1.3	1	65	northeast wind	959	7.7	0	90
2021-10-10 20:00:00	8.1	52	1	1	234	southwest wind	960	7.7	0	90
2021-10-10 21:00:00	6.8	58	1.6	2	326	northwest wind	961	7.7	0	90
2021-10-10 22:00:00	5	68	2.3	2	311	northwest wind	961	7.7	0	60
2021-10-10 23:00:00	3.3	74	3	2	265	west wind	961	7.7	0	20
2021-10-11 00:00:00	3.2	68	2.2	2	247	southwest wind	960	7.7	0	20
2021-10-11 01:00:00	2.9	70	0.5	1	309	northwest wind	960	7.7	0	90
2021-10-11 02:00:00	3.9	67	1.9	2	321	northwest wind	960	10.4	0	90
2021-10-11 03:00:00	4.3	68	3.1	2	321	northwest wind	960	10.4	0	60
2021-10-11 04:00:00	4.8	66	2.3	2	310	northwest wind	959	10.4	0	90
2021-10-11 05:00:00	5.1	65	0.5	1	321	northwest wind	959	14.7	0	90
2021-10-11 06:00:00	6.2	62	0	0	298	northwest wind	960	16.2	0	90
2021-10-11 07:00:00	9.5	49	2.2	2	298	northwest wind	960	17.1	0	90
2021-10-11 08:00:00	6.3	67	1.8	2	226	southwest wind	960	15.7	0	90

2021-10-11 09:00:00	5.8	73	2.3	2	156	southeast wind	961	12.1	0	90
2021-10-11 10:00:00	5.9	81	2.9	2	300	northwest wind	962	12.1	0	90
2021-10-11 11:00:00	6.7	84	0.8	1	0	north wind	962	12.1	0	90
2021-10-11 12:00:00	9.1	69	0.9	1	152	southeast wind	962	12.1	0.3	90
2021-10-11 13:00:00	10.4	51	0.5	1	204	southwest wind	962	12.1	0	60
2021-10-11 14:00:00	11	50	1.4	1	24	northeast wind	961	12.1	0	90
2021-10-11 15:00:00	11.7	43	1.1	1	82	east wind	961	19.8	0	60
2021-10-11 16:00:00	13	39	1.8	2	123	southeast wind	960	18	0	60
2021-10-11 17:00:00	12.4	39	1.7	2	113	southeast wind	960	18.4	0	60
2021-10-11 18:00:00	12.4	40	2	2	147	southeast wind	960	19.6	0	60
2021-10-11 19:00:00	11.7	46	2.4	2	58	northeast wind	960	19.7	0	20
2021-10-11 20:00:00	9.4	57	2	2	55	northeast wind	961	12.1	0	20
2021-10-11 21:00:00	6.9	71	1.1	1	334	northwest wind	961	14.5	0	20
2021-10-11 22:00:00	4.8	76	0.5	1	312	northwest wind	962	8.3	0	20
2021-10-11 23:00:00	3.8	79	0	0	334	northwest wind	962	9.8	0	20
2021-10-12 00:00:00	2	85	2.3	2	331	northwest wind	962	7.4	0	20
2021-10-12 01:00:00	2	82	2.6	2	334	northwest wind	962	13	0	20
2021-10-12 02:00:00	2.1	80	0.3	1	327	northwest wind	962	16.2	0	20
2021-10-12 03:00:00	1.8	80	3.2	2	222	southwest wind	963	15.2	0	20
2021-10-12 04:00:00	1.6	82	1.2	1	315	northwest wind	962	12.9	0	20
2021-10-12 05:00:00	0.8	83	2.1	2	204	southwest wind	963	12.2	0	20
2021-10-12 06:00:00	1.1	85	0.8	1	33	northeast wind	963	9.4	0	20
2021-10-12 07:00:00	0.4	87	3	2	327	northwest wind	963	10.9	0	20
2021-10-12 08:00:00	0.2	88	1.8	2	295	northwest wind	963	9.3	0	60
2021-10-12 09:00:00	1	86	1.7	2	221	southwest wind	964	8.5	0	60
2021-10-12 10:00:00	3.4	81	1.4	1	296	northwest wind	965	8.6	0	20
2021-10-12 11:00:00	6.6	70	0.9	1	138	southeast wind	965	8.6	0	60
2021-10-12 12:00:00	10.8	50	1.8	2	115	southeast wind	965	16.6	0	20
2021-10-12 13:00:00	12.2	41	2	2	55	northeast wind	965	30	0	60
2021-10-12 14:00:00	11.9	42	0.6	1	139	southeast wind	964	30	0	20
2021-10-12 15:00:00	12.4	40	1	1	35	northeast wind	964	30	0	60
2021-10-12 16:00:00	12.1	42	0.8	1	55	northeast wind	964	30	0	60
2021-10-12 17:00:00	12.3	41	4	3	27	northeast wind	964	30	0	59
2021-10-12 18:00:00	11.1	49	0.9	1	148	southeast wind	965	30	0	90
2021-10-12 19:00:00	9.9	51	3.2	2	43	northeast wind	966	28.3	0	77
2021-10-12 20:00:00	6.1	84	1.8	2	299	northwest wind	968	14.6	4.8	100
2021-10-12 21:00:00	5.4	76	1.4	1	53	northeast wind	969	30	0	44
2021-10-12 22:00:00	5.2	76	1.6	2	221	southwest wind	971	30	0	20

2021-10-12 23:00:00	4.1	83	3.1	2	155	southeast wind	971	30	0	12
2021-10-13 00:00:00	2.9	90	1.9	2	317	northwest wind	972	30	0	1
2021-10-13 01:00:00	2.2	92	2.6	2	60	northeast wind	972	25	0	0
2021-10-13 02:00:00	2.2	94	3	2	303	northwest wind	972	18.5	0	0
2021-10-13 03:00:00	1.5	91	3.1	2	329	northwest wind	972	26.2	0	0
2021-10-13 04:00:00	1.5	88	2.5	2	335	northwest wind	973	30	0	13
2021-10-13 05:00:00	0.9	90	0	0	333	northwest wind	973	30	0	3
2021-10-13 06:00:00	-0.1	91	2.5	2	314	northwest wind	973	30	0	0
2021-10-13 07:00:00	-0.6	92	1.5	1	298	northwest wind	973	29.4	0	9
2021-10-13 08:00:00	-0.7	92	1.8	2	301	northwest wind	973	23.8	0	1
2021-10-13 09:00:00	-0.5	87	2.6	2	336	northwest wind	974	24	0	15
2021-10-13 10:00:00	1.2	82	0.1	0	296	northwest wind	974	24.3	0	4
2021-10-13 11:00:00	4.6	70	0.2	0	330	northwest wind	974	28.4	0	6
2021-10-13 12:00:00	8	55	0.1	0	332	northwest wind	974	30	0	16
2021-10-13 13:00:00	9.2	49	0.2	0	147	southeast wind	974	30	0	0
2021-10-13 14:00:00	10.1	47	1	1	51	northeast wind	973	30	0	0
2021-10-13 15:00:00	11.3	42	2.4	2	237	southwest wind	972	30	0	0
2021-10-13 16:00:00	11.6	41	2.7	2	61	northeast wind	972	30	0	0
2021-10-13 17:00:00	11.8	37	1.7	2	152	southeast wind	971	30	0	0
2021-10-13 18:00:00	11.7	38	1.9	2	119	southeast wind	971	30	0	0
2021-10-13 19:00:00	10.7	39	0.3	1	152	southeast wind	972	30	0	0
2021-10-13 20:00:00	8.6	53	1.7	2	144	southeast wind	972	25.2	0	0
2021-10-13 21:00:00	7.3	53	2.2	2	40	northeast wind	973	22	0	0
2021-10-13 22:00:00	4.2	71	3	2	310	northwest wind	973	14.2	0	0
2021-10-13 23:00:00	2.4	76	0.6	1	315	northwest wind	973	11.3	0	0
2021-10-14 00:00:00	1.2	80	0.1	0	328	northwest wind	973	13.7	0	0
2021-10-14 01:00:00	0.6	80	1.7	2	307	northwest wind	973	14.4	0	0
2021-10-14 02:00:00	0.8	81	2.9	2	306	northwest wind	973	20.5	0	0
2021-10-14 03:00:00	0.4	84	3.1	2	324	northwest wind	972	20.8	0	0
2021-10-14 04:00:00	0.5	82	2.5	2	335	northwest wind	972	22.4	0	0
2021-10-14 05:00:00	0	83	1.6	2	329	northwest wind	972	23	0	0
2021-10-14 06:00:00	-0.3	85	1.6	2	312	northwest wind	971	21.6	0	0
2021-10-14 07:00:00	-0.5	85	1.1	1	297	northwest wind	971	20.2	0	0
2021-10-14 08:00:00	-0.6	84	0.3	1	324	northwest wind	972	8.8	0	0
2021-10-14 09:00:00	0	83	1.9	2	304	northwest wind	972	17	0	0
2021-10-14 10:00:00	1.8	82	2.6	2	333	northwest wind	972	14.9	0	39
2021-10-14 11:00:00	4.6	71	1.4	1	317	northwest wind	972	12.3	0	45
2021-10-14 12:00:00	5.5	66	0.7	1	48	northeast wind	971	24.7	0	71

2021-10-14 13:00:00	6.2	63	2.1	2	115	southeast wind	971	22.7	0	46
2021-10-14 14:00:00	7.5	61	3.2	2	142	southeast wind	970	25.8	0	11
2021-10-14 15:00:00	8.8	54	1.8	2	28	northeast wind	969	20.4	0	5
2021-10-14 16:00:00	9.8	48	0.4	1	121	southeast wind	968	25.4	0	8
2021-10-14 17:00:00	10.2	41	0.7	1	25	northeast wind	967	25.3	0	7
2021-10-14 18:00:00	10.4	42	2.6	2	325	northwest wind	967	27.7	0	18
2021-10-14 19:00:00	9.2	46	2.9	2	38	northeast wind	967	26.8	0	0
2021-10-14 20:00:00	6	65	0.4	1	300	northwest wind	967	21.2	0	0
2021-10-14 21:00:00	4.1	74	1.3	1	298	northwest wind	968	5.8	0	0
2021-10-14 22:00:00	2.4	77	2.5	2	326	northwest wind	968	7.3	0	0
2021-10-14 23:00:00	1.5	78	0.4	1	316	northwest wind	968	9.1	0	0
2021-10-15 00:00:00	1.9	77	0.3	1	334	northwest wind	967	9.1	0	0
2021-10-15 01:00:00	2	77	0.2	0	320	northwest wind	967	10.7	0	0
2021-10-15 02:00:00	1.7	81	0.5	1	312	northwest wind	966	9.8	0	0
2021-10-15 03:00:00	1.1	88	2.1	2	333	northwest wind	966	9.4	0	0
2021-10-15 04:00:00	0.8	87	0.9	1	325	northwest wind	965	11	0	0
2021-10-15 05:00:00	0.3	87	2.4	2	329	northwest wind	965	11.1	0	0
2021-10-15 06:00:00	-0.4	90	2.3	2	313	northwest wind	965	9.6	0	0
2021-10-15 07:00:00	-0.4	91	1.6	2	297	northwest wind	965	9.4	0	0
2021-10-15 08:00:00	-0.2	88	1.4	1	305	northwest wind	965	9.4	0	0
2021-10-15 09:00:00	-0.6	86	1.4	1	334	northwest wind	966	9.4	0	0
2021-10-15 10:00:00	1.8	81	2.7	2	304	northwest wind	966	9.4	0	13
2021-10-15 11:00:00	6.6	62	2.7	2	327	northwest wind	966	9.4	0	39
2021-10-15 12:00:00	7.9	57	1.3	1	229	southwest wind	966	9.4	0	8
2021-10-15 13:00:00	9.9	45	0	0	318	northwest wind	965	9.4	0	0
2021-10-15 14:00:00	10.5	45	2.8	2	140	southeast wind	965	9.4	0	0
2021-10-15 15:00:00	11.5	41	2.6	2	146	southeast wind	964	9.4	0	0
2021-10-15 16:00:00	11.6	41	2.7	2	324	northwest wind	964	9.4	0	1
2021-10-15 17:00:00	11.6	42	2.7	2	35	northeast wind	964	9.4	0	0
2021-10-15 18:00:00	11.2	42	2	2	143	southeast wind	964	9.4	0	18
2021-10-15 19:00:00	10.4	43	2.7	2	42	northeast wind	964	9.4	0	0
2021-10-15 20:00:00	7.2	62	0	0	309	northwest wind	965	10.6	0	0
2021-10-15 21:00:00	4.8	72	1.9	2	303	northwest wind	965	5.6	0	0
2021-10-15 22:00:00	3	79	1.2	1	310	northwest wind	966	5.5	0	0
2021-10-15 23:00:00	2	77	0.8	1	309	northwest wind	965	6.3	0	16
2021-10-16 00:00:00	1.9	78	1.6	2	320	northwest wind	965	4.6	0	12
2021-10-16 01:00:00	1.9	81	0.1	0	300	northwest wind	965	4.8	0	52
2021-10-16 02:00:00	2.1	79	1.7	2	304	northwest wind	964	7.2	0	83

2021-10-16 03:00:00	2	79	2.3	2	332	northwest wind	964	9.2	0	83
2021-10-16 04:00:00	2	78	2.3	2	314	northwest wind	964	10.8	0	90
2021-10-16 05:00:00	1.4	80	1.7	2	296	northwest wind	963	13.4	0	0
2021-10-16 06:00:00	0.9	80	1.1	1	328	northwest wind	963	12.3	0	0
2021-10-16 07:00:00	0.4	81	1.3	1	335	northwest wind	963	13.6	0	0
2021-10-16 08:00:00	0.2	80	0.4	1	328	northwest wind	963	13.6	0	0
2021-10-16 09:00:00	0.4	79	1.8	2	303	northwest wind	964	8.1	0	0
2021-10-16 10:00:00	2.2	74	1.8	2	298	northwest wind	964	10.3	0	1
2021-10-16 11:00:00	5.8	62	2.7	2	225	southwest wind	964	13.8	0	0
2021-10-16 12:00:00	9.6	50	1.2	1	63	northeast wind	964	13.2	0	0
2021-10-16 13:00:00	11.7	42	2.5	2	133	southeast wind	963	15.8	0	0
2021-10-16 14:00:00	13.1	36	2.7	2	120	southeast wind	962	17.6	0	0
2021-10-16 15:00:00	13.7	36	2.1	2	240	southwest wind	961	16.1	0	0
2021-10-16 16:00:00	13.7	34	0.4	1	115	southeast wind	960	17.2	0	4
2021-10-16 17:00:00	14	33	1	1	60	northeast wind	960	16.7	0	0
2021-10-16 18:00:00	14.1	34	0.6	1	35	northeast wind	960	16.3	0	0
2021-10-16 19:00:00	12.7	38	0	0	47	northeast wind	960	15.5	0	0
2021-10-16 20:00:00	8.7	58	2	2	296	northwest wind	960	11.2	0	0
2021-10-16 21:00:00	6.3	67	1.3	1	324	northwest wind	961	5	0	0
2021-10-16 22:00:00	4.8	69	3.2	2	295	northwest wind	962	5.2	0	0
2021-10-16 23:00:00	3.7	70	1.4	1	299	northwest wind	962	7.6	0	0
2021-10-17 00:00:00	3	73	3.1	2	302	northwest wind	961	8.9	0	0
2021-10-17 01:00:00	2.9	75	0.5	1	305	northwest wind	961	9.4	0	0
2021-10-17 02:00:00	2.1	78	1.8	2	326	northwest wind	960	8.9	0	0
2021-10-17 03:00:00	1.1	81	2.7	2	308	northwest wind	960	9.7	0	0
2021-10-17 04:00:00	1.3	80	1.8	2	301	northwest wind	960	9.7	0	0
2021-10-17 05:00:00	1.2	79	0.7	1	311	northwest wind	959	11.1	0	0
2021-10-17 06:00:00	1	80	0.3	1	325	northwest wind	959	10.8	0	0
2021-10-17 07:00:00	0.6	81	2	2	302	northwest wind	960	11.5	0	0
2021-10-17 08:00:00	0.5	81	1.5	1	295	northwest wind	960	10.1	0	2
2021-10-17 09:00:00	0.5	81	2.1	2	303	northwest wind	961	10.2	0	10
2021-10-17 10:00:00	2.5	74	2.5	2	325	northwest wind	961	10.3	0	48
2021-10-17 11:00:00	6.5	62	1.9	2	328	northwest wind	962	12.8	0	2
2021-10-17 12:00:00	11.1	47	0.9	1	240	southwest wind	962	13.6	0	0
2021-10-17 13:00:00	13.6	38	2.9	2	150	southeast wind	961	14.7	0	0
2021-10-17 14:00:00	14.4	34	0.3	1	66	northeast wind	961	14.3	0	0
2021-10-17 15:00:00	15	32	2.8	2	153	southeast wind	960	14.7	0	0
2021-10-17 16:00:00	16.3	29	2.1	2	205	southwest wind	960	14.8	0	3

2021-10-17 17:00:00	16.2	31	3	2	145	southeast wind	960	10.3	0	0
2021-10-17 18:00:00	16	30	2.4	2	137	southeast wind	960	14	0	10
2021-10-17 19:00:00	14.3	37	1.4	1	156	southeast wind	960	13.8	0	0
2021-10-17 20:00:00	10.2	54	0	0	0	north wind	960	12.8	0	0
2021-10-17 21:00:00	7.7	62	2	2	336	northwest wind	961	5	0	0
2021-10-17 22:00:00	5.8	67	0	0	322	northwest wind	961	6.1	0	0
2021-10-17 23:00:00	5.1	65	0.6	1	329	northwest wind	961	6.8	0	0
2021-10-18 00:00:00	4.7	67	0.8	1	317	northwest wind	961	8.3	0	0
2021-10-18 01:00:00	5	66	1.3	1	303	northwest wind	961	9.7	0	0
2021-10-18 02:00:00	4.3	67	2.5	2	321	northwest wind	960	8.2	0	0
2021-10-18 03:00:00	3.6	70	0.1	0	336	northwest wind	960	8.3	0	4
2021-10-18 04:00:00	3.7	68	0.8	1	315	northwest wind	960	12	0	1
2021-10-18 05:00:00	3.5	69	1.6	2	295	northwest wind	960	13.5	0	1
2021-10-18 06:00:00	2.8	70	2.1	2	318	northwest wind	959	14.9	0	0
2021-10-18 07:00:00	2.2	71	1	1	313	northwest wind	960	15.9	0	0
2021-10-18 08:00:00	2	73	2.7	2	322	northwest wind	960	15.9	0	0
2021-10-18 09:00:00	1.9	71	2.3	2	325	northwest wind	960	15.9	0	0
2021-10-18 10:00:00	4	64	0.3	1	314	northwest wind	961	17	0	0
2021-10-18 11:00:00	7.5	56	3	2	300	northwest wind	961	16.4	0	0
2021-10-18 12:00:00	11.9	43	1.2	1	132	southeast wind	961	16.3	0	0
2021-10-18 13:00:00	13.6	37	0.4	1	156	southeast wind	960	14.4	0	0
2021-10-18 14:00:00	15.2	33	2.6	2	155	southeast wind	960	16.2	0	0
2021-10-18 15:00:00	16.2	32	0.5	1	135	southeast wind	959	18	0	0
2021-10-18 16:00:00	16.6	30	0	0	123	southeast wind	959	16.5	0	0
2021-10-18 17:00:00	16.3	29	1.4	1	37	northeast wind	959	16.8	0	0
2021-10-18 18:00:00	15.8	30	0.5	1	126	southeast wind	959	17	0	0
2021-10-18 19:00:00	14.4	34	3	2	126	southeast wind	959	17.5	0	0
2021-10-18 20:00:00	10.2	52	3	2	66	northeast wind	959	9.6	0	0
2021-10-18 21:00:00	7.5	59	0.3	1	334	northwest wind	960	6.8	0	0
2021-10-18 22:00:00	5.5	64	0.5	1	306	northwest wind	960	7.9	0	0
2021-10-18 23:00:00	5.7	61	1.4	1	306	northwest wind	961	9	0	0
2021-10-19 00:00:00	4.6	67	0.7	1	310	northwest wind	960	9.3	0	0
2021-10-19 01:00:00	4.2	68	0.1	0	311	northwest wind	960	9.5	0	0
2021-10-19 02:00:00	4.1	68	0.1	0	302	northwest wind	960	11.9	0	0
2021-10-19 03:00:00	3.3	70	2.8	2	305	northwest wind	960	13.4	0	0
2021-10-19 04:00:00	2.9	71	1.7	2	304	northwest wind	960	13.3	0	0
2021-10-19 05:00:00	2.5	72	0	0	296	northwest wind	959	13.5	0	0
2021-10-19 06:00:00	2.2	73	2.5	2	333	northwest wind	959	14	0	0

2021-10-19 07:00:00	2.1	72	2.5	2	311	northwest wind	960	14.6	0	0
2021-10-19 08:00:00	1.8	72	0.4	1	298	northwest wind	960	14.7	0	0
2021-10-19 09:00:00	1.8	72	2.7	2	295	northwest wind	960	12.3	0	0
2021-10-19 10:00:00	4	64	2	2	295	northwest wind	961	10.6	0	0
2021-10-19 11:00:00	7.4	56	1	1	324	northwest wind	961	12.7	0	0
2021-10-19 12:00:00	11.4	36	0	0	42	northeast wind	960	12.4	0	0
2021-10-19 13:00:00	12.3	34	2	2	27	northeast wind	960	12.6	0	0
2021-10-19 14:00:00	13.1	32	0.4	1	140	southeast wind	959	12.7	0	0
2021-10-19 15:00:00	13.8	32	2.9	2	33	northeast wind	958	12.8	0	0
2021-10-19 16:00:00	14	33	2.2	2	28	northeast wind	958	12.6	0	48
2021-10-19 17:00:00	14.3	32	0.2	0	115	southeast wind	958	11.8	0	40
2021-10-19 18:00:00	14.3	33	0.1	0	149	southeast wind	958	10.2	0	11
2021-10-19 19:00:00	12.7	38	0.1	0	54	northeast wind	958	8.8	0	0
2021-10-19 20:00:00	8.9	51	3	2	296	northwest wind	958	7.5	0	28
2021-10-19 21:00:00	6.7	60	0.3	1	333	northwest wind	958	3.5	0	0
2021-10-19 22:00:00	6	59	2.5	2	316	northwest wind	959	5.5	0	44
2021-10-19 23:00:00	5.6	60	0.7	1	305	northwest wind	959	5.9	0	62
2021-10-20 00:00:00	5.6	61	2.2	2	306	northwest wind	959	6.5	0	53
2021-10-20 01:00:00	5	63	1	1	310	northwest wind	959	8.1	0	57
2021-10-20 02:00:00	3.8	65	0.5	1	299	northwest wind	958	8.5	0	5
2021-10-20 03:00:00	3.1	68	1.3	1	335	northwest wind	958	8.3	0	1
2021-10-20 04:00:00	3.2	64	2.4	2	300	northwest wind	958	10.7	0	0
2021-10-20 05:00:00	3	63	2.2	2	318	northwest wind	958	13.1	0	0
2021-10-20 06:00:00	2.5	63	0.7	1	311	northwest wind	958	16	0	1
2021-10-20 07:00:00	2	67	1.9	2	308	northwest wind	958	15	0	2
2021-10-20 08:00:00	1.8	68	0.4	1	323	northwest wind	959	12.5	0	8
2021-10-20 09:00:00	2.3	66	1.1	1	312	northwest wind	959	11.7	0	6
2021-10-20 10:00:00	4.1	61	2.9	2	293	northwest wind	959	11.6	0	20
2021-10-20 11:00:00	7.8	51	1.3	1	329	northwest wind	960	10.9	0	0
2021-10-20 12:00:00	11.6	39	1.9	2	303	northwest wind	959	12.4	0	0
2021-10-20 13:00:00	14.3	33	2	2	335	northwest wind	959	9.9	0	0
2021-10-20 14:00:00	15.6	30	0.9	1	127	southeast wind	958	8.9	0	0
2021-10-20 15:00:00	16.2	30	0.9	1	114	southeast wind	957	10.6	0	0
2021-10-20 16:00:00	16.5	30	2.2	2	209	southwest wind	957	12.2	0	0
2021-10-20 17:00:00	16.3	28	1.3	1	135	southeast wind	957	10.7	0	1
2021-10-20 18:00:00	15.8	29	1	1	133	southeast wind	957	10.5	0	0
2021-10-20 19:00:00	12.8	42	2.3	2	49	northeast wind	957	10	0	0
2021-10-20 20:00:00	9.9	51	2.2	2	297	northwest wind	957	7	0	0

2021-10-20 21:00:00	8.1	56	1.8	2	332	northwest wind	958	4.5	0	61
2021-10-20 22:00:00	7.2	58	2.1	2	296	northwest wind	958	4.1	0	29
2021-10-20 23:00:00	6.9	59	2.3	2	332	northwest wind	958	5.6	0	35
2021-10-21 00:00:00	6.1	62	2.4	2	299	northwest wind	958	7.3	0	2
2021-10-21 01:00:00	5.7	62	3	2	315	northwest wind	957	8.6	0	0
2021-10-21 02:00:00	5.3	62	2.8	2	312	northwest wind	957	9.9	0	0
2021-10-21 03:00:00	4.4	66	1.2	1	319	northwest wind	957	9	0	0
2021-10-21 04:00:00	4.1	65	2.3	2	297	northwest wind	957	9.8	0	2
2021-10-21 05:00:00	3.5	66	2.1	2	251	west wind	957	9.8	0	0
2021-10-21 06:00:00	2.8	70	3	2	305	northwest wind	957	9.6	0	0
2021-10-21 07:00:00	2.6	69	0.6	1	314	northwest wind	957	10.5	0	0
2021-10-21 08:00:00	2.5	69	1.8	2	299	northwest wind	957	10.5	0	0
2021-10-21 09:00:00	2.3	69	1.8	2	321	northwest wind	958	10.6	0	0
2021-10-21 10:00:00	3.6	66	1.6	2	208	southwest wind	958	9.5	0	0
2021-10-21 11:00:00	8.1	54	2.8	2	233	southwest wind	958	10.5	0	0
2021-10-21 12:00:00	12.5	42	0	0	67	northeast wind	958	10.3	0	0
2021-10-21 13:00:00	15.3	30	1.1	1	118	southeast wind	958	7.9	0	0
2021-10-21 14:00:00	16.2	31	1.9	2	149	southeast wind	957	8.8	0	0
2021-10-21 15:00:00	16.9	29	0.3	1	119	southeast wind	956	8.8	0	0
2021-10-21 16:00:00	17	28	1	1	154	southeast wind	955	9	0	10
2021-10-21 17:00:00	17.1	27	0.8	1	144	southeast wind	955	9.5	0	22
2021-10-21 18:00:00	16.2	30	0.2	0	127	southeast wind	955	9.1	0	1
2021-10-21 19:00:00	14.5	36	2.5	2	133	southeast wind	955	8.4	0	12
2021-10-21 20:00:00	11.6	44	0.2	0	43	northeast wind	955	7	0	3
2021-10-21 21:00:00	8.5	55	0	0	205	southwest wind	956	5.7	0	0
2021-10-21 22:00:00	6.4	61	1.3	1	325	northwest wind	956	4.5	0	0
2021-10-21 23:00:00	5	66	0.8	1	298	northwest wind	956	5.8	0	0
2021-10-22 00:00:00	4.7	66	1	1	305	northwest wind	956	5.5	0	0
2021-10-22 01:00:00	4.1	67	0.3	1	324	northwest wind	955	5.6	0	0
2021-10-22 02:00:00	3.8	69	0.2	0	309	northwest wind	955	6.3	0	0
2021-10-22 03:00:00	3.3	70	3.2	2	293	northwest wind	955	6.9	0	0
2021-10-22 04:00:00	3	71	1.2	1	334	northwest wind	955	7.1	0	0
2021-10-22 05:00:00	2.6	72	2.3	2	294	northwest wind	955	7.1	0	0
2021-10-22 06:00:00	2.4	72	0.2	0	309	northwest wind	955	7.2	0	1
2021-10-22 07:00:00	2.6	71	1.1	1	318	northwest wind	956	6.1	0	50
2021-10-22 08:00:00	3	70	1.2	1	220	southwest wind	956	2.6	0	31
2021-10-22 09:00:00	3.6	65	3.1	2	216	southwest wind	957	9.6	0	58
2021-10-22 10:00:00	4.7	62	2.4	2	304	northwest wind	958	8.7	0	57

2021-10-22 11:00:00	10.2	45	3.2	2	53	northeast wind	959	9	0	12
2021-10-22 12:00:00	12.3	40	2.6	2	147	southeast wind	960	9.5	0	12
2021-10-22 13:00:00	12.5	41	1	1	300	northwest wind	960	10.6	0	0
2021-10-22 14:00:00	12.7	46	2.4	2	330	northwest wind	960	13	0	25
2021-10-22 15:00:00	11.7	52	4.2	3	129	southeast wind	960	13.4	0	41
2021-10-22 16:00:00	10.1	54	2.5	2	201	south wind	961	13.4	0	100
2021-10-22 17:00:00	9.6	53	2.8	2	144	southeast wind	961	8.2	0	100
2021-10-22 18:00:00	9.2	52	1.6	2	122	southeast wind	962	9.7	0	100
2021-10-22 19:00:00	8.4	56	2.4	2	240	southwest wind	963	9.3	0	100
2021-10-22 20:00:00	7.1	71	0.5	1	149	southeast wind	964	7.7	0	100
2021-10-22 21:00:00	5.8	79	0.9	1	38	northeast wind	966	9.9	0.5	100
2021-10-22 22:00:00	4.8	86	1.3	1	113	southeast wind	967	8.3	1.2	100
2021-10-22 23:00:00	4	91	0.9	1	136	southeast wind	968	9.1	1.1	100
2021-10-23 00:00:00	3.5	93	1.2	1	141	southeast wind	968	10.7	1	100
2021-10-23 01:00:00	3.3	94	2.6	2	117	southeast wind	969	6.8	1.2	100
2021-10-23 02:00:00	3	94	0.1	0	153	southeast wind	970	11.4	1.3	100
2021-10-23 03:00:00	3.1	94	2.8	2	177	south wind	970	11.4	0.7	100
2021-10-23 04:00:00	2.9	94	2.7	2	140	southeast wind	970	8.9	0.6	100
2021-10-23 05:00:00	3	95	2.9	2	117	southeast wind	971	8.9	0.5	100
2021-10-23 06:00:00	3.1	94	0.3	1	216	southwest wind	971	12.8	0.6	100
2021-10-23 07:00:00	3.1	93	0.9	1	222	southwest wind	971	11.9	0.2	100
2021-10-23 08:00:00	3.3	92	0	0	217	southwest wind	972	11.9	0	100
2021-10-23 09:00:00	3.3	91	0.1	0	235	southwest wind	972	30	0	100
2021-10-23 10:00:00	3.7	86	2.4	2	208	southwest wind	973	30	0	100
2021-10-23 11:00:00	4	83	0.3	1	247	southwest wind	974	30	0	100
2021-10-23 12:00:00	4.2	81	1.7	2	228	southwest wind	974	30	0	100
2021-10-23 13:00:00	4.8	79	1.3	1	216	southwest wind	974	30	0	100
2021-10-23 14:00:00	6	73	0.9	1	122	southeast wind	973	22.7	0	63
2021-10-23 15:00:00	6.4	70	0.6	1	203	southwest wind	973	30	0	35
2021-10-23 16:00:00	7.3	62	2.6	2	149	southeast wind	972	30	0	53
2021-10-23 17:00:00	7.7	58	2.8	2	59	northeast wind	972	30	0	21
2021-10-23 18:00:00	8.1	55	0.1	0	227	southwest wind	972	30	0	0
2021-10-23 19:00:00	6.6	66	0.6	1	204	southwest wind	972	30	0	0
2021-10-23 20:00:00	4	82	0.8	1	328	northwest wind	973	20.5	0	0
2021-10-23 21:00:00	2.3	88	2.1	2	0	north wind	973	12.2	0	0
2021-10-23 22:00:00	1.5	92	3.2	2	299	northwest wind	974	5.1	0	0
2021-10-23 23:00:00	0.8	93	1.2	1	323	northwest wind	974	5.6	0	0
2021-10-24 00:00:00	0.6	93	0.9	1	310	northwest wind	974	5.6	0	0

2021-10-24 01:00:00	-0.3	91	1.8	2	332	northwest wind	973	9.6	0	0
2021-10-24 02:00:00	-0.3	91	0.8	1	300	northwest wind	973	10.6	0	0
2021-10-24 03:00:00	-0.6	93	2.7	2	307	northwest wind	973	9.5	0	0
2021-10-24 04:00:00	-0.7	92	1.8	2	319	northwest wind	972	10.4	0	0
2021-10-24 05:00:00	-1.4	92	0.3	1	311	northwest wind	972	10.4	0	0
2021-10-24 06:00:00	-1.3	92	1.3	1	337	northwest wind	972	12	0	0
2021-10-24 07:00:00	-1.5	92	1.1	1	315	northwest wind	972	13.8	0	0
2021-10-24 08:00:00	-1.7	91	0.5	1	304	northwest wind	972	13.1	0	0
2021-10-24 09:00:00	-2	91	2.9	2	311	northwest wind	972	12.9	0	0
2021-10-24 10:00:00	-0.6	88	1.2	1	330	northwest wind	972	13.6	0	0
2021-10-24 11:00:00	2.2	82	3.1	2	290	west wind	973	14.2	0	0
2021-10-24 12:00:00	5.2	70	0.3	1	310	northwest wind	972	16.1	0	0
2021-10-24 13:00:00	7.2	62	2.9	2	127	southeast wind	971	18	0	0
2021-10-24 14:00:00	8.2	56	1.3	1	131	southeast wind	970	20.5	0	0
2021-10-24 15:00:00	9.2	54	0.6	1	123	southeast wind	970	19.6	0	0
2021-10-24 16:00:00	9.7	52	1.1	1	140	southeast wind	969	20.1	0	0
2021-10-24 17:00:00	9.5	51	2.7	2	156	southeast wind	969	20.2	0	0
2021-10-24 18:00:00	9.4	51	0.5	1	118	southeast wind	969	23.3	0	0
2021-10-24 19:00:00	7.9	55	2.8	2	132	southeast wind	969	20.5	0	0
2021-10-24 20:00:00	5.4	69	1.2	1	27	northeast wind	969	16	0	0
2021-10-24 21:00:00	3.6	79	2.4	2	297	northwest wind	969	9.1	0	0
2021-10-24 22:00:00	2.2	85	2.7	2	321	northwest wind	969	5.4	0	0
2021-10-24 23:00:00	0.9	89	1.9	2	315	northwest wind	968	5.9	0	0
2021-10-25 00:00:00	0.3	91	1.3	1	306	northwest wind	968	5.2	0	0
2021-10-25 01:00:00	-0.5	91	2.3	2	300	northwest wind	968	7.5	0	0
2021-10-25 02:00:00	-0.6	90	2.2	2	315	northwest wind	967	8.1	0	0
2021-10-25 03:00:00	-0.9	89	1.6	2	324	northwest wind	966	10.2	0	0
2021-10-25 04:00:00	-1.1	90	0.9	1	334	northwest wind	966	8.8	0	0
2021-10-25 05:00:00	-1.1	91	1.8	2	298	northwest wind	965	8.8	0	0
2021-10-25 06:00:00	-1.3	90	2.5	2	300	northwest wind	965	9	0	0
2021-10-25 07:00:00	-1.5	90	0.7	1	333	northwest wind	965	8.9	0	0
2021-10-25 08:00:00	-1.5	90	2.9	2	335	northwest wind	965	8.7	0	0
2021-10-25 09:00:00	-1.5	89	2.3	2	323	northwest wind	965	7.7	0	0
2021-10-25 10:00:00	-0.2	86	0.3	1	299	northwest wind	965	8	0	0
2021-10-25 11:00:00	3	78	0	0	311	northwest wind	965	10.1	0	0
2021-10-25 12:00:00	6.2	58	0.1	0	325	northwest wind	965	10.1	0	0
2021-10-25 13:00:00	7.1	56	3.2	2	293	northwest wind	964	16.5	0	0
2021-10-25 14:00:00	8.1	54	2.6	2	204	southwest wind	963	16.5	0	0

2021-10-25 15:00:00	8.7	51	2.1	2	323	northwest wind	962	16.7	0	0
2021-10-25 16:00:00	9.6	46	1	1	306	northwest wind	962	17.6	0	0
2021-10-25 17:00:00	9.5	46	0.8	1	306	northwest wind	961	17.9	0	0
2021-10-25 18:00:00	9.6	46	1.9	2	308	northwest wind	961	18.9	0	0
2021-10-25 19:00:00	8	52	1.5	1	320	northwest wind	961	15.5	0	0
2021-10-25 20:00:00	6.1	64	2.4	2	329	northwest wind	961	8.8	0	0
2021-10-25 21:00:00	4.5	69	1.1	1	335	northwest wind	961	6.9	0	0
2021-10-25 22:00:00	3.1	74	1.1	1	334	northwest wind	961	4.5	0	0
2021-10-25 23:00:00	2.5	76	0.5	1	302	northwest wind	961	6.5	0	0
2021-10-26 00:00:00	2.1	78	1.7	2	324	northwest wind	961	6.5	0	0
2021-10-26 01:00:00	1.4	81	1.9	2	294	northwest wind	961	7.8	0	0
2021-10-26 02:00:00	0.7	82	2.2	2	234	southwest wind	960	8.4	0	0
2021-10-26 03:00:00	-0.6	88	0.7	1	239	southwest wind	960	8.1	0	0
2021-10-26 04:00:00	-0.4	86	3.1	2	328	northwest wind	959	8.7	0	0
2021-10-26 05:00:00	-0.9	88	2.6	2	336	northwest wind	959	8.5	0	0
2021-10-26 06:00:00	-1.3	87	3.1	2	334	northwest wind	959	9.1	0	0
2021-10-26 07:00:00	-1.7	86	2.9	2	302	northwest wind	959	11.1	0	0
2021-10-26 08:00:00	-1.8	84	1.4	1	297	northwest wind	959	11.5	0	0
2021-10-26 09:00:00	-1.9	82	1	1	316	northwest wind	959	13.5	0	0
2021-10-26 10:00:00	-0.8	78	0.8	1	232	southwest wind	960	13.5	0	0
2021-10-26 11:00:00	1.7	74	1.3	1	321	northwest wind	960	13.5	0	0
2021-10-26 12:00:00	-1.7	65	0.4	1	220	southwest wind	959	13.5	0	0
2021-10-26 13:00:00	-1.7	49	0.2	0	204	southwest wind	959	13.5	0	0
2021-10-26 14:00:00	-1.7	45	0.1	0	124	southeast wind	958	13.5	0	0
2021-10-26 15:00:00	-1.7	42	0.2	0	122	southeast wind	958	13.5	0	0
2021-10-26 16:00:00	-1.7	42	3	2	120	southeast wind	958	13.5	0	0
2021-10-26 17:00:00	-1.7	40	1.8	2	151	southeast wind	958	13.5	0	0
2021-10-26 18:00:00	11.6	40	2.3	2	157	southeast wind	958	12.2	0	0
2021-10-26 19:00:00	10	49	1.1	1	32	northeast wind	958	10	0	0
2021-10-26 20:00:00	6.8	65	1.2	1	28	northeast wind	959	5.7	0	0
2021-10-26 21:00:00	4.5	77	1.7	2	325	northwest wind	959	2.4	0	0
2021-10-26 22:00:00	3.1	79	2.7	2	305	northwest wind	959	4.1	0	0
2021-10-26 23:00:00	2.5	77	0.3	1	298	northwest wind	959	4.7	0	0
2021-10-27 00:00:00	2.3	78	1.6	2	305	northwest wind	960	5.7	0	0
2021-10-27 01:00:00	2	77	2.1	2	332	northwest wind	960	7.6	0	0
2021-10-27 02:00:00	1.4	79	3	2	309	northwest wind	960	9.3	0	0
2021-10-27 03:00:00	1	78	0	0	328	northwest wind	960	9.2	0	0
2021-10-27 04:00:00	0.9	80	3	2	323	northwest wind	960	11.6	0	0

2021-10-27 05:00:00	0.9	79	1.8	2	306	northwest wind	960	14.2	0	0
2021-10-27 06:00:00	1.7	76	2.6	2	327	northwest wind	960	14.2	0	0
2021-10-27 07:00:00	2.2	74	2.1	2	295	northwest wind	960	25.4	0	0
2021-10-27 08:00:00	2.6	72	0.9	1	312	northwest wind	960	25.4	0	13
2021-10-27 09:00:00	3	70	0.8	1	309	northwest wind	961	26.7	0	16
2021-10-27 10:00:00	3.5	69	2.8	2	293	northwest wind	961	22.9	0	64
2021-10-27 11:00:00	6	62	0.4	1	230	southwest wind	961	24.6	0	18
2021-10-27 12:00:00	10.6	47	1.3	1	214	southwest wind	961	23.7	0	0
2021-10-27 13:00:00	13.6	36	0.6	1	143	southeast wind	961	24	0	0
2021-10-27 14:00:00	15.3	31	0.8	1	133	southeast wind	960	24	0	16
2021-10-27 15:00:00	16.4	33	0.4	1	126	southeast wind	960	23.7	0	0
2021-10-27 16:00:00	15.9	34	0	0	142	southeast wind	959	16.8	0	11
2021-10-27 17:00:00	15.3	33	0.1	0	155	southeast wind	959	18.2	0	1
2021-10-27 18:00:00	14.5	37	1.6	2	134	southeast wind	960	14.8	0	22
2021-10-27 19:00:00	12.5	47	0.8	1	51	northeast wind	960	12	0	83
2021-10-27 20:00:00	10.8	53	0.3	1	40	northeast wind	961	6.8	0	100
2021-10-27 21:00:00	9.3	61	1.3	1	0	north wind	961	5.2	0	77
2021-10-27 22:00:00	7.9	69	0.5	1	329	northwest wind	962	5.8	0	90
2021-10-27 23:00:00	7.1	71	3.1	2	276	west wind	962	6.2	0	65
2021-10-28 00:00:00	6.4	70	0	0	297	northwest wind	962	6.7	0	72
2021-10-28 01:00:00	6	69	0.3	1	324	northwest wind	962	9.2	0	14
2021-10-28 02:00:00	5.8	68	2.6	2	337	northwest wind	962	7.9	0	0
2021-10-28 03:00:00	5.7	68	0.3	1	333	northwest wind	961	6.9	0	0
2021-10-28 04:00:00	4.7	71	0.4	1	320	northwest wind	961	8.4	0	0
2021-10-28 05:00:00	3.2	77	0.4	1	325	northwest wind	961	8.5	0	0
2021-10-28 06:00:00	2.5	81	0.3	1	226	southwest wind	961	7.4	0	0
2021-10-28 07:00:00	2.9	78	1.7	2	333	northwest wind	961	9	0	0
2021-10-28 08:00:00	2.6	78	2.9	2	328	northwest wind	962	12.1	0	3
2021-10-28 09:00:00	2.4	78	2.2	2	326	northwest wind	962	16.8	0	67
2021-10-28 10:00:00	2.9	76	0.5	1	337	northwest wind	962	16.8	0	70
2021-10-28 11:00:00	5	70	1.1	1	322	northwest wind	962	16.8	0	72
2021-10-28 12:00:00	8.7	60	0.6	1	242	southwest wind	962	16.9	0	64
2021-10-28 13:00:00	10.6	52	0.5	1	32	northeast wind	962	9.4	0	78
2021-10-28 14:00:00	11.8	48	2.1	2	146	southeast wind	962	11.2	0	83
2021-10-28 15:00:00	12.8	43	3.1	2	41	northeast wind	961	11.2	0	69
2021-10-28 16:00:00	13.5	39	0.3	1	128	southeast wind	961	11.2	0	40
2021-10-28 17:00:00	13.7	36	3	2	28	northeast wind	961	12.9	0	7
2021-10-28 18:00:00	13.1	39	0.9	1	129	southeast wind	961	4.9	0	67

2021-10-28 19:00:00	11.2	51	3.2	2	224	southwest wind	961	7.8	0	61
2021-10-28 20:00:00	9.2	57	2.4	2	299	northwest wind	962	6.4	0	90
2021-10-28 21:00:00	6.7	69	0.8	1	321	northwest wind	962	6.4	0	72
2021-10-28 22:00:00	5.4	70	0	0	309	northwest wind	962	5.4	0	3
2021-10-28 23:00:00	5.2	67	0.7	1	323	northwest wind	962	7.7	0	2
2021-10-29 00:00:00	5.2	68	1.8	2	306	northwest wind	963	7.7	0	2
2021-10-29 01:00:00	4.6	70	0.3	1	303	northwest wind	962	11.6	0	16
2021-10-29 02:00:00	3.9	72	3	2	310	northwest wind	962	11.8	0	0
2021-10-29 03:00:00	3.3	74	0.5	1	306	northwest wind	962	12.1	0	0
2021-10-29 04:00:00	2.9	76	2.4	2	324	northwest wind	962	12.1	0	0
2021-10-29 05:00:00	2.6	77	2.6	2	330	northwest wind	962	13.4	0	0
2021-10-29 06:00:00	2.2	78	2.1	2	311	northwest wind	962	14.2	0	0
2021-10-29 07:00:00	1.8	79	3	2	333	northwest wind	963	13.1	0	0
2021-10-29 08:00:00	1.9	78	2.2	2	303	northwest wind	963	13.1	0	0
2021-10-29 09:00:00	1.9	77	0.2	0	309	northwest wind	964	10.2	0	0
2021-10-29 10:00:00	3.5	71	2	2	318	northwest wind	964	14.2	0	0
2021-10-29 11:00:00	6.6	63	0.5	1	293	northwest wind	964	15.3	0	0
2021-10-29 12:00:00	9.9	53	2.4	2	302	northwest wind	964	16.8	0	0
2021-10-29 13:00:00	13.4	41	0	0	143	southeast wind	964	18.8	0	0
2021-10-29 14:00:00	15	35	0.9	1	142	southeast wind	963	16.1	0	0
2021-10-29 15:00:00	15.3	33	0.9	1	127	southeast wind	963	17	0	0
2021-10-29 16:00:00	15.3	34	2.9	2	117	southeast wind	963	17	0	6
2021-10-29 17:00:00	14.9	37	1	1	126	southeast wind	963	17	0	1
2021-10-29 18:00:00	14.5	37	2.3	2	146	southeast wind	963	15.3	0	10
2021-10-29 19:00:00	12.2	47	1.1	1	0	north wind	964	13.1	0	0
2021-10-29 20:00:00	9.2	59	0.7	1	326	northwest wind	964	9	0	0
2021-10-29 21:00:00	6.9	68	0.7	1	319	northwest wind	965	4.1	0	0
2021-10-29 22:00:00	5.6	71	0.6	1	326	northwest wind	966	4.3	0	0
2021-10-29 23:00:00	5.1	71	1.2	1	276	west wind	966	4.3	0	58
2021-10-30 00:00:00	4.6	71	2.7	2	321	northwest wind	966	4.3	0	65
2021-10-30 01:00:00	3.8	78	0.8	1	253	west wind	966	8.9	0	90
2021-10-30 02:00:00	3.4	80	2.7	2	312	northwest wind	966	8.6	0	64
2021-10-30 03:00:00	3	82	2.4	2	333	northwest wind	966	9.2	0	100
2021-10-30 04:00:00	4.8	75	0.6	1	332	northwest wind	967	9.9	0	100
2021-10-30 05:00:00	5.5	73	0.3	1	335	northwest wind	967	11	0	100
2021-10-30 06:00:00	5.6	73	1.7	2	335	northwest wind	967	11.9	0	100
2021-10-30 07:00:00	5.8	72	2.7	2	328	northwest wind	967	11.9	0	100
2021-10-30 08:00:00	6	72	3.1	2	295	northwest wind	968	11.9	0	100

2021-10-30 09:00:00	6.1	74	0.8	1	307	northwest wind	968	11.9	0	100
2021-10-30 10:00:00	6.4	73	1	1	293	northwest wind	968	8.6	0	83
2021-10-30 11:00:00	8	69	0.1	0	310	northwest wind	969	7.3	0	52
2021-10-30 12:00:00	10.6	56	1.7	2	299	northwest wind	969	11.6	0	14
2021-10-30 13:00:00	12.1	49	1.7	2	136	southeast wind	968	12	0	0
2021-10-30 14:00:00	13.1	45	1.9	2	152	southeast wind	968	11.2	0	2
2021-10-30 15:00:00	13.1	43	0.1	0	146	southeast wind	967	13.7	0	15
2021-10-30 16:00:00	13.8	41	0	0	124	southeast wind	966	16.5	0	16
2021-10-30 17:00:00	13.6	39	1.4	1	127	southeast wind	966	16.5	0	0
2021-10-30 18:00:00	12.7	45	2.6	2	151	southeast wind	966	15.7	0	0
2021-10-30 19:00:00	11.4	50	3.1	2	151	southeast wind	966	15.7	0	0
2021-10-30 20:00:00	8.2	65	3.2	2	297	northwest wind	966	6.1	0	0
2021-10-30 21:00:00	6	70	0.9	1	290	west wind	967	4	0	0
2021-10-30 22:00:00	5.1	75	2.3	2	306	northwest wind	967	5.7	0	0
2021-10-30 23:00:00	4.4	74	1.4	1	301	northwest wind	967	8.2	0	0
2021-10-31 00:00:00	3.5	76	2.1	2	329	northwest wind	966	8.2	0	44
2021-10-31 01:00:00	3.2	77	0.4	1	298	northwest wind	966	9	0	11
2021-10-31 02:00:00	2.7	79	2.7	2	316	northwest wind	965	7	0	1
2021-10-31 03:00:00	2.8	79	2.8	2	308	northwest wind	964	11.1	0	44
2021-10-31 04:00:00	2.6	81	2.4	2	333	northwest wind	964	10.1	0	64
2021-10-31 05:00:00	2.9	79	0.3	1	322	northwest wind	963	11.1	0	52
2021-10-31 06:00:00	2.5	81	0.6	1	227	southwest wind	963	10.5	0	44
2021-10-31 07:00:00	2.4	82	2.7	2	301	northwest wind	964	9.5	0	48
2021-10-31 08:00:00	2.2	82	1.2	1	327	northwest wind	964	9.5	0	100
2021-10-31 09:00:00	2.3	82	2.2	2	270	west wind	964	6.4	0	100
2021-10-31 10:00:00	3	80	0.3	1	316	northwest wind	964	7.7	0	100
2021-10-31 11:00:00	4.4	77	0.5	1	298	northwest wind	964	8.2	0	100
2021-10-31 12:00:00	5.9	73	1.5	1	309	northwest wind	964	8	0	55
2021-10-31 13:00:00	9.3	54	0.9	1	228	southwest wind	963	10.2	0	70
2021-10-31 14:00:00	11.7	47	2	2	316	northwest wind	963	15.1	0	51
2021-10-31 15:00:00	13.1	43	2.8	2	133	southeast wind	962	16.2	0	1
2021-10-31 16:00:00	12.8	43	2.9	2	147	southeast wind	962	14.6	0	3
2021-10-31 17:00:00	13.3	42	1.1	1	224	southwest wind	962	16.8	0	2
2021-10-31 18:00:00	12.5	43	0.6	1	155	southeast wind	962	16.8	0	0
2021-10-31 19:00:00	11.4	49	1.1	1	163	south wind	962	18.6	0	71
2021-10-31 20:00:00	9.2	57	1.9	2	323	northwest wind	962	15.8	0	100
2021-10-31 21:00:00	7.8	63	1.7	2	273	west wind	963	5.9	0	100
2021-10-31 22:00:00	5.6	71	3.1	2	334	northwest wind	963	6.7	0	100

2021-10-31 23:00:00	4.5	75	0.7	1	314	northwest wind	963	8.6	0	100
2021-11-01 00:00:00	4.1	73	2.3	2	330	northwest wind	964	8.6	0	100
2021-11-01 01:00:00	3.3	75	2.2	2	319	northwest wind	964	30	0	70
2021-11-01 02:00:00	2.2	80	1.2	1	301	northwest wind	964	30	0	41
2021-11-01 03:00:00	2.1	81	3	2	242	southwest wind	964	30	0	100
2021-11-01 04:00:00	2.6	78	3.2	2	297	northwest wind	964	30	0	85
2021-11-01 05:00:00	1.8	83	2.9	2	330	northwest wind	965	30	0	87
2021-11-01 06:00:00	1.2	85	0.9	1	324	northwest wind	965	30	0	56
2021-11-01 07:00:00	0.9	87	1.9	2	310	northwest wind	965	30	0	64
2021-11-01 08:00:00	0.9	87	1.9	2	310	northwest wind	965	30	0	100
2021-11-01 09:00:00	2.2	88	3	2	311	northwest wind	966	30	0	100
2021-11-01 10:00:00	2.2	87	2.6	2	335	northwest wind	966	30	0	100
2021-11-01 11:00:00	2.8	83	1.6	2	336	northwest wind	967	30	0	100
2021-11-01 12:00:00	4.9	76	0.7	1	329	northwest wind	967	30	0	67
2021-11-01 13:00:00	8.2	58	1.2	1	207	southwest wind	966	30	0	64
2021-11-01 14:00:00	9.8	53	2.3	2	216	southwest wind	966	30	0	6
2021-11-01 15:00:00	9.2	55	2.1	2	137	southeast wind	965	30	0	90
2021-11-01 16:00:00	8.9	55	2	2	234	southwest wind	964	30	0	70
2021-11-01 17:00:00	9.3	53	1.8	2	237	southwest wind	964	30	0	66
2021-11-01 18:00:00	9.1	54	0.6	1	145	southeast wind	964	30	0	100
2021-11-01 19:00:00	8.1	57	1.1	1	131	southeast wind	964	30	0	100
2021-11-01 20:00:00	6.5	65	3.2	2	208	southwest wind	964	30	0	100
2021-11-01 21:00:00	4.7	73	0.4	1	298	northwest wind	964	30	0	84
2021-11-01 22:00:00	3.1	78	2.9	2	0	north wind	965	30	0	42
2021-11-01 23:00:00	2.1	81	2.3	2	316	northwest wind	964	30	0	55
2021-11-02 00:00:00	1.3	83	0.2	0	245	southwest wind	964	30	0	59
2021-11-02 01:00:00	1.6	80	2.2	2	321	northwest wind	964	30	0	76
2021-11-02 02:00:00	2	77	2.8	2	307	northwest wind	963	30	0	100
2021-11-02 03:00:00	1.1	79	2.6	2	307	northwest wind	963	30	0	100
2021-11-02 04:00:00	0.4	81	2.4	2	294	northwest wind	963	30	0	45
2021-11-02 05:00:00	0	82	2.5	2	317	northwest wind	962	30	0	46
2021-11-02 06:00:00	-0.2	82	2.7	2	308	northwest wind	962	30	0	10
2021-11-02 07:00:00	-0.4	82	1.1	1	299	northwest wind	961	30	0	66
2021-11-02 08:00:00	-0.7	82	1.3	1	235	southwest wind	962	30	0	60
2021-11-02 09:00:00	-0.5	81	2.1	2	321	northwest wind	961	30	0	34
2021-11-02 10:00:00	0.8	76	2.2	2	337	northwest wind	961	30	0	62
2021-11-02 11:00:00	2.7	70	1.6	2	317	northwest wind	961	30	0	90
2021-11-02 12:00:00	5	65	0	0	294	northwest wind	960	30	0	53

2021-11-02 13:00:00	8.1	53	2	2	219	southwest wind	960	30	0	31
2021-11-02 14:00:00	10.7	45	0.3	1	114	southeast wind	959	30	0	0
2021-11-02 15:00:00	11.8	42	2.3	2	183	south wind	958	30	0	0
2021-11-02 16:00:00	12.4	39	1.8	2	130	southeast wind	957	30	0	46
2021-11-02 17:00:00	12	39	2.6	2	132	southeast wind	956	30	0	18
2021-11-02 18:00:00	11.7	40	2.9	2	229	southwest wind	956	30	0	0
2021-11-02 19:00:00	9.1	49	2.9	2	206	southwest wind	956	15.3	0	17
2021-11-02 20:00:00	6.4	62	2.2	2	337	northwest wind	956	15.3	0	61
2021-11-02 21:00:00	4.3	65	0.1	0	330	northwest wind	956	5	0	87
2021-11-02 22:00:00	2.7	74	0	0	325	northwest wind	955	7.8	0	0
2021-11-02 23:00:00	2.9	69	0.2	0	314	northwest wind	955	9.1	0	32
2021-11-03 00:00:00	2.4	71	0	0	333	northwest wind	954	9.1	0	16
2021-11-03 01:00:00	2.2	73	2.2	2	301	northwest wind	954	12.5	0	10
2021-11-03 02:00:00	1.8	72	0.3	1	308	northwest wind	953	11.9	0	28
2021-11-03 03:00:00	1.3	72	1.7	2	305	northwest wind	953	16.4	0	14
2021-11-03 04:00:00	0.3	76	0.5	1	212	southwest wind	952	16.4	0	0
2021-11-03 05:00:00	-0.5	79	1.9	2	293	northwest wind	952	15	0	0
2021-11-03 06:00:00	-0.5	78	0	0	312	northwest wind	951	14.5	0	0
2021-11-03 07:00:00	-0.9	77	2.8	2	334	northwest wind	951	12	0	0
2021-11-03 08:00:00	-0.8	77	0.8	1	213	southwest wind	950	12	0	0
2021-11-03 09:00:00	-1.3	79	0.4	1	308	northwest wind	950	6	0	0
2021-11-03 10:00:00	0.6	73	0	0	305	northwest wind	950	7.7	0	0
2021-11-03 11:00:00	4.2	63	1.8	2	300	northwest wind	950	14.2	0	0
2021-11-03 12:00:00	3.1	49	0.8	1	117	southeast wind	950	14.2	0	0
2021-11-03 13:00:00	10.1	50	3	2	152	southeast wind	950	18.3	0	0
2021-11-03 14:00:00	9.7	52	1.2	1	155	southeast wind	949	17.5	0	1
2021-11-03 15:00:00	10.6	48	2.2	2	119	southeast wind	949	17.3	0	0
2021-11-03 16:00:00	8.7	46	3.9	3	129	southeast wind	949	17.3	0	16
2021-11-03 17:00:00	6.8	47	3.8	3	239	southwest wind	949	8.2	0	70
2021-11-03 18:00:00	4.7	50	3.9	3	115	southeast wind	950	8.2	0	87
2021-11-03 19:00:00	2.9	51	3.6	3	147	southeast wind	951	6.1	0	84
2021-11-03 20:00:00	0.2	53	4.9	3	135	southeast wind	952	4.4	0	69
2021-11-03 21:00:00	-0.4	56	3.7	3	130	southeast wind	953	3.3	0	67
2021-11-03 22:00:00	-1	58	2	2	125	southeast wind	954	4.6	0	100
2021-11-03 23:00:00	-1.8	54	7.7	4	136	southeast wind	955	5.4	0	100
2021-11-04 00:00:00	-2.3	54	3.5	3	116	southeast wind	956	8	0	100
2021-11-04 01:00:00	-2.9	58	4.7	3	130	southeast wind	957	7.4	0	100
2021-11-04 02:00:00	-3.6	57	7	4	125	southeast wind	958	4.3	0	76

2021-11-04 03:00:00	-4.1	58	5.2	3	128	southeast wind	959	7.3	0	100
2021-11-04 04:00:00	-4.7	57	6.9	4	155	southeast wind	960	9.7	0	100
2021-11-04 05:00:00	-5.4	65	4.5	3	113	southeast wind	960	11.6	0	100
2021-11-04 06:00:00	-6.3	75	3.7	3	123	southeast wind	961	6.1	0.3	90
2021-11-04 07:00:00	-6.3	66	1.8	2	143	southeast wind	962	16.4	0	100
2021-11-04 08:00:00	-6.6	58	0	0	245	southwest wind	964	16.4	0	100
2021-11-04 09:00:00	-6.8	56	0.8	1	126	southeast wind	965	28	0	100
2021-11-04 10:00:00	-6.7	58	5.2	3	116	southeast wind	966	21.4	0	64
2021-11-04 11:00:00	-5.6	55	3	2	129	southeast wind	967	25.3	0	100
2021-11-04 12:00:00	-5.8	61	3.9	3	139	southeast wind	968	25.3	0	100
2021-11-04 13:00:00	-4.5	54	5.1	3	223	southwest wind	968	25.3	0	65
2021-11-04 14:00:00	-4.2	59	1.8	2	119	southeast wind	969	30	0	100
2021-11-04 15:00:00	-4.1	54	2.6	2	141	southeast wind	969	30	0	54
2021-11-04 16:00:00	-3	50	0	0	301	northwest wind	969	30	0	0
2021-11-04 17:00:00	-3.6	54	1.7	2	306	northwest wind	969	30	0	10
2021-11-04 18:00:00	-3.2	51	2.3	2	307	northwest wind	969	28.9	0	4
2021-11-04 19:00:00	-4.2	55	0.4	1	331	northwest wind	970	29.6	0	0
2021-11-04 20:00:00	-4.9	58	0.8	1	147	southeast wind	972	29.6	0	0
2021-11-04 21:00:00	-5.6	72	0	0	123	southeast wind	973	21.8	0	0
2021-11-04 22:00:00	-6.2	75	2.3	2	150	southeast wind	973	24.9	0	8
2021-11-04 23:00:00	-6.4	65	2.7	2	127	southeast wind	974	17.7	0	4
2021-11-05 00:00:00	-7.2	59	1.7	2	145	southeast wind	975	17.7	0	6
2021-11-05 01:00:00	-7.5	59	0.5	1	130	southeast wind	976	27.8	0	56
2021-11-05 02:00:00	-8.1	63	1.4	1	150	southeast wind	976	28.6	0	7
2021-11-05 03:00:00	-9.7	72	1.8	2	217	southwest wind	976	28.7	0	0
2021-11-05 04:00:00	-10.6	79	1	1	308	northwest wind	977	28.7	0	0
2021-11-05 05:00:00	-11.1	80	2.6	2	327	northwest wind	977	18.5	0	0
2021-11-05 06:00:00	-11.6	81	2.8	2	48	northeast wind	977	20.5	0	0
2021-11-05 07:00:00	-11.1	79	2.6	2	311	northwest wind	978	29.1	0	0
2021-11-05 08:00:00	-12	80	0	0	325	northwest wind	979	29.1	0	0
2021-11-05 09:00:00	-11.9	79	2.6	2	299	northwest wind	979	11.5	0	0
2021-11-05 10:00:00	-11.1	80	3	2	329	northwest wind	980	13.8	0	0
2021-11-05 11:00:00	-7.7	69	2.5	2	305	northwest wind	980	13.3	0	0
2021-11-05 12:00:00	-5.4	60	0.5	1	305	northwest wind	980	13.3	0	0
2021-11-05 13:00:00	-3.2	53	3.2	2	220	southwest wind	980	23.6	0	0
2021-11-05 14:00:00	-2.5	47	1.3	1	35	northeast wind	980	24.8	0	0
2021-11-05 15:00:00	-1.3	44	0	0	121	southeast wind	980	28.4	0	0
2021-11-05 16:00:00	-1.8	52	0.3	1	125	southeast wind	981	28.4	0	0

2021-11-05 17:00:00	-1.6	51	0.9	1	122	southeast wind	981	18.8	0	0
2021-11-05 18:00:00	-2.5	54	0.7	1	144	southeast wind	982	20.8	0	0
2021-11-05 19:00:00	-3.7	59	1.9	2	148	southeast wind	982	21.4	0	0
2021-11-05 20:00:00	-4.6	62	2.1	2	133	southeast wind	983	21.4	0	0
2021-11-05 21:00:00	-5.1	61	2.4	2	149	southeast wind	983	21.1	0	0
2021-11-05 22:00:00	-5.8	60	0.7	1	132	southeast wind	983	21.7	0	0
2021-11-05 23:00:00	-7.3	66	2.7	2	121	southeast wind	983	25.9	0	0
2021-11-06 00:00:00	-8.4	77	2.9	2	120	southeast wind	983	25.9	0	0
2021-11-06 01:00:00	-8.5	80	3.1	2	135	southeast wind	983	17.5	0	0
2021-11-06 02:00:00	-9.5	84	2.2	2	36	northeast wind	983	13.8	0	0
2021-11-06 03:00:00	-9.4	85	1.5	1	52	northeast wind	983	10.5	0	0
2021-11-06 04:00:00	-10.3	88	0.1	0	54	northeast wind	983	10.5	0	0
2021-11-06 05:00:00	-10.5	88	3.1	2	310	northwest wind	983	10.6	0	0
2021-11-06 06:00:00	-11.3	86	2	2	318	northwest wind	982	11.6	0	0
2021-11-06 07:00:00	-11.5	85	2	2	322	northwest wind	982	12.3	0	0
2021-11-06 08:00:00	-11.5	86	1.5	1	326	northwest wind	983	12.3	0	0
2021-11-06 09:00:00	-11.4	85	1.7	2	295	northwest wind	983	10.5	0	2
2021-11-06 10:00:00	-10.2	84	3	2	322	northwest wind	983	8.4	0	0
2021-11-06 11:00:00	-9	81	1.5	1	26	northeast wind	983	9.4	0	0
2021-11-06 12:00:00	-8.9	78	2.4	2	28	northeast wind	983	9.4	0	17
2021-11-06 13:00:00	-7	69	0.2	0	43	northeast wind	982	18.8	0	0
2021-11-06 14:00:00	-5.6	64	2.6	2	119	southeast wind	981	17.4	0	0
2021-11-06 15:00:00	-5	60	2.6	2	55	northeast wind	980	18.9	0	0
2021-11-06 16:00:00	-4.6	57	3.2	2	56	northeast wind	979	18.9	0	0
2021-11-06 17:00:00	-4.7	53	2	2	59	northeast wind	979	25	0	0
2021-11-06 18:00:00	-5.3	61	2	2	126	southeast wind	979	17.2	0	0
2021-11-06 19:00:00	-6.3	65	0.1	0	123	southeast wind	979	17.2	0	0
2021-11-06 20:00:00	-6.9	69	2.1	2	147	southeast wind	979	17.2	0	0
2021-11-06 21:00:00	-7.9	76	1.9	2	42	northeast wind	979	6.4	0	0
2021-11-06 22:00:00	-8.5	79	3.1	2	50	northeast wind	978	9	0	0
2021-11-06 23:00:00	-9	82	1.2	1	36	northeast wind	978	8.4	0	0
2021-11-07 00:00:00	-8.2	79	0.9	1	37	northeast wind	978	8.4	0	0
2021-11-07 01:00:00	-8.6	82	1.5	1	323	northwest wind	977	10.8	0	0
2021-11-07 02:00:00	-8.9	84	1.4	1	53	northeast wind	976	12.3	0	0
2021-11-07 03:00:00	-9.9	86	2.8	2	312	northwest wind	976	8.9	0	0
2021-11-07 04:00:00	-10.4	86	1.2	1	320	northwest wind	975	8.9	0	0
2021-11-07 05:00:00	-11.1	86	2.6	2	306	northwest wind	975	9.6	0	0
2021-11-07 06:00:00	-11.5	86	1.4	1	310	northwest wind	974	9.6	0	0

2021-11-07 07:00:00	-11.8	85	1.8	2	316	northwest wind	974	10.6	0	0
2021-11-07 08:00:00	-11.8	85	0.2	0	309	northwest wind	974	10.6	0	0
2021-11-07 09:00:00	-12.3	84	1.1	1	329	northwest wind	974	6.6	0	0
2021-11-07 10:00:00	-11.5	85	0.7	1	327	northwest wind	974	5.1	0	0
2021-11-07 11:00:00	-8.7	83	2.5	2	0	north wind	974	4.1	0	0
2021-11-07 12:00:00	-6.4	74	1.4	1	206	southwest wind	974	4.1	0	0
2021-11-07 13:00:00	-4.3	65	1.9	2	124	southeast wind	973	7.6	0	0
2021-11-07 14:00:00	-3.5	58	2.2	2	136	southeast wind	972	9.3	0	0
2021-11-07 15:00:00	-2.4	54	1.2	1	209	southwest wind	972	9.2	0	0
2021-11-07 16:00:00	-2.1	53	1.8	2	145	southeast wind	971	9.2	0	0
2021-11-07 17:00:00	-2.2	51	2.3	2	73	east wind	971	12	0	0
2021-11-07 18:00:00	-2.3	53	0.8	1	144	southeast wind	972	11.7	0	0
2021-11-07 19:00:00	-3.6	61	0.3	1	138	southeast wind	972	11.1	0	0
2021-11-07 20:00:00	-4.7	67	2.5	2	31	northeast wind	972	11.1	0	0
2021-11-07 21:00:00	-6.4	74	3.1	2	301	northwest wind	973	5.8	0	0
2021-11-07 22:00:00	-7.4	79	2.6	2	323	northwest wind	973	4.3	0	0
2021-11-07 23:00:00	-8.2	80	1.5	1	57	northeast wind	973	4.1	0	0
2021-11-08 00:00:00	-8.7	79	1.4	1	311	northwest wind	972	4.1	0	0
2021-11-08 01:00:00	-9.8	83	2.9	2	317	northwest wind	972	4.7	0	2
2021-11-08 02:00:00	-10	84	2.1	2	305	northwest wind	972	5.8	0	1
2021-11-08 03:00:00	-10.5	86	1	1	313	northwest wind	971	3.9	0	2
2021-11-08 04:00:00	-10.8	86	1.8	2	316	northwest wind	971	3.9	0	6
2021-11-08 05:00:00	-11.4	84	2.8	2	333	northwest wind	971	5.6	0	1
2021-11-08 06:00:00	-11.8	84	2	2	308	northwest wind	972	5.9	0	1
2021-11-08 07:00:00	-11.7	86	0.4	1	309	northwest wind	972	5.1	0	0
2021-11-08 08:00:00	-12.2	84	1.5	1	336	northwest wind	972	5.1	0	1
2021-11-08 09:00:00	-12.3	84	0	0	315	northwest wind	972	4.2	0	0
2021-11-08 10:00:00	-11.4	84	1.5	1	297	northwest wind	973	4	0	0
2021-11-08 11:00:00	-7.1	81	1.4	1	315	northwest wind	972	3.6	0	0
2021-11-08 12:00:00	-4.8	67	0.3	1	302	northwest wind	972	3.6	0	0
2021-11-08 13:00:00	-3.2	58	0.8	1	32	northeast wind	972	8.5	0	0
2021-11-08 14:00:00	-1.6	50	0.4	1	241	southwest wind	972	9.4	0	0
2021-11-08 15:00:00	-1.2	50	2.1	2	128	southeast wind	971	9.2	0	0
2021-11-08 16:00:00	-1.1	49	2.1	2	119	southeast wind	971	9.2	0	0
2021-11-08 17:00:00	-1.2	50	1.9	2	122	southeast wind	971	10.2	0	0
2021-11-08 18:00:00	-2	54	2.5	2	138	southeast wind	972	9.7	0	3
2021-11-08 19:00:00	-3.1	56	3.1	2	141	southeast wind	972	9.8	0	0
2021-11-08 20:00:00	-4.3	62	0	0	55	northeast wind	973	9.8	0	0

2021-11-08 21:00:00	-5.5	64	0.7	1	40	northeast wind	973	7.3	0	1
2021-11-08 22:00:00	-7.2	71	2.2	2	297	northwest wind	973	8.4	0	3
2021-11-08 23:00:00	-8.1	75	2.3	2	302	northwest wind	973	1.9	0	4
2021-11-09 00:00:00	-8.9	79	1.7	2	326	northwest wind	973	1.9	0	6
2021-11-09 01:00:00	-9.9	81	3.1	2	205	southwest wind	973	5.1	0	5
2021-11-09 02:00:00	-9.7	82	1.8	2	304	northwest wind	973	5.3	0	3
2021-11-09 03:00:00	-10.7	81	1.4	1	210	southwest wind	973	5.9	0	0
2021-11-09 04:00:00	-10.4	84	0	0	326	northwest wind	973	5.9	0	0
2021-11-09 05:00:00	-10.7	81	3.2	2	326	northwest wind	973	6.1	0	0
2021-11-09 06:00:00	-11.1	82	0.1	0	324	northwest wind	973	5.9	0	0
2021-11-09 07:00:00	-10.9	82	1.5	1	326	northwest wind	973	5.7	0	0
2021-11-09 08:00:00	-11.1	81	1.3	1	316	northwest wind	973	5.7	0	0
2021-11-09 09:00:00	-11.6	81	1.9	2	326	northwest wind	973	4.1	0	0
2021-11-09 10:00:00	-11.2	83	0	0	304	northwest wind	973	4.3	0	0
2021-11-09 11:00:00	-7.1	75	1.8	2	116	southeast wind	973	4.9	0	0
2021-11-09 12:00:00	-4.5	61	0	0	117	southeast wind	973	4.9	0	0
2021-11-09 13:00:00	-3.5	61	2.2	2	90	east wind	973	5.7	0	0
2021-11-09 14:00:00	-2.4	56	0.9	1	129	southeast wind	972	5.6	0	0
2021-11-09 15:00:00	-1.1	52	2	2	150	southeast wind	972	6.4	0	0
2021-11-09 16:00:00	-0.7	50	2.8	2	150	southeast wind	972	6.4	0	0
2021-11-09 17:00:00	-0.4	50	2.9	2	125	southeast wind	971	6.9	0	0
2021-11-09 18:00:00	-1.1	52	1.1	1	145	southeast wind	971	6.4	0	0
2021-11-09 19:00:00	-2.3	57	3.2	2	131	southeast wind	972	6.1	0	0
2021-11-09 20:00:00	-4.5	66	0.9	1	6	north wind	972	6.1	0	0
2021-11-09 21:00:00	-5.7	71	1	1	29	northeast wind	972	4.2	0	2
2021-11-09 22:00:00	-6.6	73	2.9	2	325	northwest wind	972	3.9	0	0
2021-11-09 23:00:00	-7.8	78	0.2	0	325	northwest wind	971	2.7	0	0
2021-11-10 00:00:00	-8.8	82	0.6	1	311	northwest wind	971	2.7	0	0
2021-11-10 01:00:00	-9.2	83	0.3	1	300	northwest wind	971	3.9	0	4
2021-11-10 02:00:00	-9.5	83	1.1	1	327	northwest wind	971	3.6	0	4
2021-11-10 03:00:00	-9.7	83	0.2	0	301	northwest wind	970	3.6	0	0
2021-11-10 04:00:00	-10.4	81	3.2	2	242	southwest wind	970	3.6	0	4
2021-11-10 05:00:00	-10.9	83	2.3	2	240	southwest wind	970	3.9	0	0
2021-11-10 06:00:00	-11.1	84	3.1	2	295	northwest wind	969	3.9	0	0
2021-11-10 07:00:00	-10.7	84	0.4	1	311	northwest wind	970	3.8	0	1
2021-11-10 08:00:00	-11.3	83	1.4	1	293	northwest wind	970	3.8	0	0
2021-11-10 09:00:00	-11.2	82	1.5	1	204	southwest wind	970	3.6	0	2
2021-11-10 10:00:00	-10.7	82	2.3	2	309	northwest wind	970	3.5	0	0

2021-11-10 11:00:00	-7.8	79	0.7	1	331	northwest wind	970	4.3	0	0
2021-11-10 12:00:00	-4.2	67	0.4	1	310	northwest wind	970	4.3	0	0
2021-11-10 13:00:00	-1.5	55	2	2	145	southeast wind	969	5.3	0	0
2021-11-10 14:00:00	-0.6	49	1	1	138	southeast wind	969	6.3	0	0
2021-11-10 15:00:00	0.5	49	2.8	2	135	southeast wind	968	6.4	0	0
2021-11-10 16:00:00	0.6	47	2.3	2	148	southeast wind	968	6.4	0	0
2021-11-10 17:00:00	0.1	50	2.1	2	148	southeast wind	968	6.2	0	0
2021-11-10 18:00:00	-0.4	51	3.1	2	138	southeast wind	968	5.8	0	0
2021-11-10 19:00:00	-1.4	55	2.1	2	128	southeast wind	969	5.4	0	0
2021-11-10 20:00:00	-2.7	64	2.4	2	125	southeast wind	969	5.4	0	0
2021-11-10 21:00:00	-4.8	72	1	1	39	northeast wind	969	3.5	0	0
2021-11-10 22:00:00	-5.8	75	3.1	2	298	northwest wind	969	2.6	0	0
2021-11-10 23:00:00	-6.8	78	2.8	2	297	northwest wind	969	3.1	0	8
2021-11-11 00:00:00	-7.6	80	1.2	1	298	northwest wind	968	3.1	0	0
2021-11-11 01:00:00	-8.1	83	2.6	2	322	northwest wind	968	2.8	0	5
2021-11-11 02:00:00	-8.6	83	1.7	2	204	southwest wind	967	3.1	0	0
2021-11-11 03:00:00	-9.3	84	0	0	316	northwest wind	967	2.8	0	0
2021-11-11 04:00:00	-9	84	1.7	2	297	northwest wind	967	2.8	0	0
2021-11-11 05:00:00	-9.9	84	1.5	1	327	northwest wind	967	3.1	0	0
2021-11-11 06:00:00	-9.8	83	3.1	2	307	northwest wind	967	3.2	0	0
2021-11-11 07:00:00	-9.4	82	1.7	2	296	northwest wind	967	3.9	0	10
2021-11-11 08:00:00	-9.6	83	0.8	1	229	southwest wind	967	3.9	0	0
2021-11-11 09:00:00	-8.8	82	1.4	1	295	northwest wind	967	3.5	0	34
2021-11-11 10:00:00	-7.1	79	0.7	1	218	southwest wind	967	3.3	0	67
2021-11-11 11:00:00	-4.7	75	2.3	2	321	northwest wind	968	3.9	0	22
2021-11-11 12:00:00	-1.6	60	0.5	1	121	southeast wind	967	3.9	0	51
2021-11-11 13:00:00	-1.1	58	1.6	2	144	southeast wind	967	5.4	0	48
2021-11-11 14:00:00	0.9	48	0	0	50	northeast wind	966	5.5	0	0
2021-11-11 15:00:00	1.2	47	0.2	0	123	southeast wind	966	5.4	0	2
2021-11-11 16:00:00	1.4	47	1	1	148	southeast wind	965	5.4	0	3
2021-11-11 17:00:00	1.3	46	2.6	2	123	southeast wind	966	6.2	0	8
2021-11-11 18:00:00	0.6	50	1.9	2	150	southeast wind	966	6.4	0	31
2021-11-11 19:00:00	-0.8	54	0.6	1	58	northeast wind	966	5.4	0	0
2021-11-11 20:00:00	-2.8	63	0.7	1	115	southeast wind	967	5.4	0	0
2021-11-11 21:00:00	-4	67	1.2	1	294	northwest wind	967	3.5	0	0
2021-11-11 22:00:00	-5.4	71	1.1	1	337	northwest wind	967	3.3	0	0
2021-11-11 23:00:00	-6.5	79	1	1	301	northwest wind	967	2.8	0	0
2021-11-12 00:00:00	-7.2	81	1.7	2	330	northwest wind	967	2.8	0	0

2021-11-12 01:00:00	-7.2	81	1.8	2	313	northwest wind	966	3	0	0
2021-11-12 02:00:00	-7.2	80	1.6	2	320	northwest wind	966	3.4	0	0
2021-11-12 03:00:00	-7.2	79	3.1	2	294	northwest wind	966	2.9	0	0
2021-11-12 04:00:00	-7.5	79	0.9	1	336	northwest wind	966	2.9	0	0
2021-11-12 05:00:00	-7.3	80	3.2	2	320	northwest wind	966	4.3	0	0
2021-11-12 06:00:00	-8.6	81	2.5	2	297	northwest wind	966	5	0	0
2021-11-12 07:00:00	-9.5	85	0.3	1	298	northwest wind	966	2.7	0	0
2021-11-12 08:00:00	-9.8	83	0.1	0	304	northwest wind	966	2.7	0	0
2021-11-12 09:00:00	-9.7	84	0.2	0	335	northwest wind	967	3.2	0	0
2021-11-12 10:00:00	-9.1	84	2.2	2	295	northwest wind	967	2.5	0	0
2021-11-12 11:00:00	-6.3	80	1.6	2	209	southwest wind	967	3.4	0	0
2021-11-12 12:00:00	-2.3	65	1.2	1	96	east wind	967	3.4	0	0
2021-11-12 13:00:00	0	55	2.9	2	113	southeast wind	966	4.7	0	0
2021-11-12 14:00:00	0.7	53	2.5	2	138	southeast wind	966	4.8	0	0
2021-11-12 15:00:00	1.4	51	0.3	1	119	southeast wind	965	5	0	0
2021-11-12 16:00:00	1.7	50	0.4	1	124	southeast wind	965	5	0	0
2021-11-12 17:00:00	1.6	50	0.6	1	142	southeast wind	965	4.5	0	0
2021-11-12 18:00:00	0.8	54	2.7	2	146	southeast wind	965	3.2	0	0
2021-11-12 19:00:00	-0.6	59	2.7	2	118	southeast wind	965	4	0	0
2021-11-12 20:00:00	-2	64	2.1	2	135	southeast wind	966	4	0	0
2021-11-12 21:00:00	-3.7	70	0.1	0	315	northwest wind	966	2.6	0	0
2021-11-12 22:00:00	-4.8	72	3.1	2	293	northwest wind	966	2.9	0	0
2021-11-12 23:00:00	-6.1	77	0	0	325	northwest wind	965	2.3	0	0
2021-11-13 00:00:00	-6.8	80	0.1	0	294	northwest wind	965	2.3	0	0
2021-11-13 01:00:00	-6.9	83	0.2	0	325	northwest wind	965	2.2	0	0
2021-11-13 02:00:00	-7.2	83	0.9	1	305	northwest wind	965	2.3	0	0
2021-11-13 03:00:00	-7.2	82	3.2	2	313	northwest wind	964	2.5	0	0
2021-11-13 04:00:00	-8	85	1.7	2	302	northwest wind	964	2.5	0	0
2021-11-13 05:00:00	-8	84	1	1	332	northwest wind	964	2.4	0	0
2021-11-13 06:00:00	-8.4	85	3.1	2	313	northwest wind	964	2.4	0	0
2021-11-13 07:00:00	-7.8	84	2.7	2	329	northwest wind	963	2.6	0	0
2021-11-13 08:00:00	-8.2	84	3.1	2	296	northwest wind	964	2.6	0	0
2021-11-13 09:00:00	-8.8	84	3	2	293	northwest wind	964	2.5	0	0
2021-11-13 10:00:00	-8.6	85	1	1	230	southwest wind	964	2.3	0	0
2021-11-13 11:00:00	-4.6	78	1.5	1	40	northeast wind	964	2.5	0	0
2021-11-13 12:00:00	-1.8	67	1.9	2	62	northeast wind	964	2.5	0	0
2021-11-13 13:00:00	0.8	56	1.4	1	113	southeast wind	964	3.6	0	0
2021-11-13 14:00:00	2.2	49	3	2	124	southeast wind	963	4	0	0

2021-11-13 15:00:00	3.1	47	2.8	2	140	southeast wind	962	4.5	0	0
2021-11-13 16:00:00	2.9	46	1.3	1	121	southeast wind	962	4.5	0	0
2021-11-13 17:00:00	2.8	46	1.2	1	118	southeast wind	961	4.5	0	0
2021-11-13 18:00:00	1.4	53	1.5	1	137	southeast wind	962	4.1	0	16
2021-11-13 19:00:00	-0.2	62	0.1	0	144	southeast wind	962	3.6	0	77
2021-11-13 20:00:00	-1.5	67	0.1	0	57	northeast wind	962	3.6	0	90
2021-11-13 21:00:00	-1.6	65	2.6	2	27	northeast wind	962	3	0	100
2021-11-13 22:00:00	-2.3	66	1.9	2	300	northwest wind	962	2.9	0	90
2021-11-13 23:00:00	-3.4	72	0.1	0	319	northwest wind	962	2.4	0	77
2021-11-14 00:00:00	-4.8	78	2.2	2	293	northwest wind	962	2.4	0	54
2021-11-14 01:00:00	-5.6	80	3.2	2	42	northeast wind	962	2	0	4
2021-11-14 02:00:00	-6.5	82	0.9	1	322	northwest wind	961	1.9	0	16
2021-11-14 03:00:00	-7	85	2.7	2	329	northwest wind	961	1.9	0	0
2021-11-14 04:00:00	-6.2	82	1.7	2	333	northwest wind	960	1.9	0	0
2021-11-14 05:00:00	-6.9	82	0.5	1	210	southwest wind	960	2.2	0	0
2021-11-14 06:00:00	-7.7	85	0.8	1	310	northwest wind	959	2.1	0	0
2021-11-14 07:00:00	-8.1	84	2	2	219	southwest wind	959	2.2	0	0
2021-11-14 08:00:00	-7.8	86	0.1	0	303	northwest wind	959	2.2	0	4
2021-11-14 09:00:00	-7.3	86	3.1	2	296	northwest wind	959	1.8	0	100
2021-11-14 10:00:00	-6.7	87	0.7	1	316	northwest wind	960	1.7	0	90
2021-11-14 11:00:00	-4.7	83	2.6	2	246	southwest wind	960	1.7	0	62
2021-11-14 12:00:00	-1.1	70	0.4	1	41	northeast wind	960	1.7	0	0
2021-11-14 13:00:00	0.8	60	2.5	2	147	southeast wind	959	4.1	0	6
2021-11-14 14:00:00	2.2	54	0	0	119	southeast wind	959	4	0	0
2021-11-14 15:00:00	2.5	49	1.6	2	131	southeast wind	958	4.4	0	2
2021-11-14 16:00:00	3.1	46	2.1	2	142	southeast wind	958	4.4	0	17
2021-11-14 17:00:00	3	46	1.1	1	116	southeast wind	958	4.8	0	29
2021-11-14 18:00:00	2.7	47	0.9	1	141	southeast wind	957	4.8	0	48
2021-11-14 19:00:00	0.4	57	0.3	1	30	northeast wind	957	3.9	0	64
2021-11-14 20:00:00	-1.4	65	1.3	1	296	northwest wind	958	3.9	0	73
2021-11-14 21:00:00	-2.4	66	0.9	1	309	northwest wind	958	2.5	0	100
2021-11-14 22:00:00	-2.6	67	1	1	309	northwest wind	958	3	0	90
2021-11-14 23:00:00	-2.4	66	2.7	2	315	northwest wind	958	3.2	0	100
2021-11-15 00:00:00	-3.1	71	1.4	1	217	southwest wind	957	3.2	0	100
2021-11-15 01:00:00	-3.3	75	0	0	295	northwest wind	957	3.1	0	73
2021-11-15 02:00:00	-3.7	77	0.5	1	308	northwest wind	956	3.2	0	0
2021-11-15 03:00:00	-3.2	77	1	1	313	northwest wind	956	1.9	0	0
2021-11-15 04:00:00	-3.9	80	0.2	0	276	west wind	956	1.9	0	100

2021-11-15 05:00:00	-3.2	79	0.1	0	323	northwest wind	956	3.4	0	100
2021-11-15 06:00:00	-3.2	77	1.1	1	31	northeast wind	956	3.7	0	100
2021-11-15 07:00:00	-3	78	2.8	2	325	northwest wind	956	3.5	0	100
2021-11-15 08:00:00	-2.9	81	0.8	1	336	northwest wind	956	3.5	0	100
2021-11-15 09:00:00	-1.9	76	0.2	0	318	northwest wind	957	3.8	0	100
2021-11-15 10:00:00	-0.8	76	2.3	2	297	northwest wind	958	3.7	0	100
2021-11-15 11:00:00	0.5	71	0.7	1	226	southwest wind	959	7.5	0	100
2021-11-15 12:00:00	1.3	68	1.4	1	298	northwest wind	959	7.5	0	90
2021-11-15 13:00:00	2.7	64	0.9	1	311	northwest wind	960	7.4	0	100
2021-11-15 14:00:00	5.5	49	2.8	2	135	southeast wind	960	7.3	0	24
2021-11-15 15:00:00	6.6	48	1.9	2	115	southeast wind	959	9.4	0	70
2021-11-15 16:00:00	6.1	51	0.2	0	140	southeast wind	960	9.4	0	24
2021-11-15 17:00:00	5.9	51	0.4	1	103	east wind	960	9.1	0	0
2021-11-15 18:00:00	5.9	52	0.8	1	42	northeast wind	961	9.1	0	4
2021-11-15 19:00:00	4	58	1	1	84	east wind	961	7.8	0	100
2021-11-15 20:00:00	2.8	63	0.7	1	38	northeast wind	962	7.8	0	100
2021-11-15 21:00:00	0.5	69	0.7	1	125	southeast wind	963	4.8	0	65
2021-11-15 22:00:00	0.2	75	0.4	1	238	southwest wind	963	4.6	0	72
2021-11-15 23:00:00	-1.1	79	1.4	1	309	northwest wind	963	13.7	0	15
2021-11-16 00:00:00	-2.2	82	0.2	0	152	southeast wind	964	13.7	0	0
2021-11-16 01:00:00	-3.3	85	0.9	1	332	northwest wind	964	15.9	0	0
2021-11-16 02:00:00	-4.9	86	3.2	2	337	northwest wind	964	13.1	0	0
2021-11-16 03:00:00	-4.7	88	3.1	2	298	northwest wind	964	18.8	0	0
2021-11-16 04:00:00	-4.5	86	2.5	2	321	northwest wind	963	18.8	0	0
2021-11-16 05:00:00	-5.8	86	2.8	2	309	northwest wind	963	24.2	0	0
2021-11-16 06:00:00	-6.3	88	1.6	2	329	northwest wind	963	14	0	41
2021-11-16 07:00:00	-6.1	90	0.4	1	264	west wind	963	12.9	0	51
2021-11-16 08:00:00	-5	87	1.8	2	319	northwest wind	963	12.9	0	100
2021-11-16 09:00:00	-4.3	84	0.8	1	311	northwest wind	963	9.7	0	76
2021-11-16 10:00:00	-3.5	83	1.2	1	329	northwest wind	963	12.5	0	67
2021-11-16 11:00:00	-2.6	80	2.2	2	325	northwest wind	963	13.8	0	64
2021-11-16 12:00:00	-1.4	79	1	1	333	northwest wind	962	13.8	0	74
2021-11-16 13:00:00	0.1	73	2.9	2	319	northwest wind	961	13.4	0	83
2021-11-16 14:00:00	1.1	69	2.6	2	318	northwest wind	960	14.3	0	79
2021-11-16 15:00:00	0.2	71	3	2	119	southeast wind	959	19.7	0	67
2021-11-16 16:00:00	-0.4	74	0.4	1	130	southeast wind	959	19.7	0	46
2021-11-16 17:00:00	-0.6	74	2.1	2	129	southeast wind	958	13	0	14
2021-11-16 18:00:00	-0.5	74	0.1	0	25	northeast wind	958	13.2	0	52

2021-11-16 19:00:00	-0.8	76	3.1	2	310	northwest wind	958	10.2	0	90
2021-11-16 20:00:00	-0.8	76	1.8	2	316	northwest wind	958	10.2	0	90
2021-11-16 21:00:00	-0.7	76	0.9	1	312	northwest wind	958	8.6	0	90
2021-11-16 22:00:00	-0.6	75	1	1	294	northwest wind	958	9.6	0	90
2021-11-16 23:00:00	-0.8	76	1.6	2	336	northwest wind	957	9.2	0	90
2021-11-17 00:00:00	-0.7	76	1.9	2	304	northwest wind	957	9.2	0	90
2021-11-17 01:00:00	-0.7	76	1.9	2	304	northwest wind	957	9.2	0	90
2021-11-17 02:00:00	-1	77	2.4	2	28	northeast wind	955	7.6	0	100
2021-11-17 03:00:00	-1.2	79	1.2	1	32	northeast wind	954	10.3	0	100
2021-11-17 04:00:00	-1.5	81	1.5	1	301	northwest wind	954	10.3	0	100
2021-11-17 05:00:00	-1.4	79	1.8	2	319	northwest wind	954	11.8	0	100
2021-11-17 06:00:00	-1.5	78	1.6	2	338	north wind	954	14.1	0	90
2021-11-17 07:00:00	-2.2	81	0.4	1	330	northwest wind	954	7.8	0	72
2021-11-17 08:00:00	-3.8	86	2.2	2	309	northwest wind	954	7.8	0	100
2021-11-17 09:00:00	-4.3	87	1.1	1	299	northwest wind	954	6.7	0	100
2021-11-17 10:00:00	-5.1	89	2	2	225	southwest wind	954	5.3	0	100
2021-11-17 11:00:00	-3.9	90	2.3	2	328	northwest wind	954	5.3	0	62
2021-11-17 12:00:00	-1.5	78	2	2	308	northwest wind	954	5.3	0	90
2021-11-17 13:00:00	0.9	66	1.8	2	118	southeast wind	953	12.5	0	100
2021-11-17 14:00:00	2	59	0.4	1	157	southeast wind	953	15.7	0	90
2021-11-17 15:00:00	2.3	57	0.2	0	203	southwest wind	952	16	0	90
2021-11-17 16:00:00	2.5	58	1.1	1	151	southeast wind	951	16	0	90
2021-11-17 17:00:00	2	59	2.2	2	134	southeast wind	951	14.2	0	100
2021-11-17 18:00:00	1.5	60	1.8	2	151	southeast wind	951	13.9	0	100
2021-11-17 19:00:00	0.9	66	2.1	2	138	southeast wind	952	13.5	0	100
2021-11-17 20:00:00	-0.2	73	1.7	2	132	southeast wind	954	13.5	0	100
2021-11-17 21:00:00	-0.8	77	1.9	2	37	northeast wind	955	6.1	0	100
2021-11-17 22:00:00	-0.5	78	1.7	2	230	southwest wind	957	7.6	0	100
2021-11-17 23:00:00	-1	78	1	1	332	northwest wind	958	6.7	0	100
2021-11-18 00:00:00	-1.6	81	2.7	2	310	northwest wind	959	6.7	0	100
2021-11-18 01:00:00	-1.5	79	1.6	2	186	south wind	959	8.3	0	100
2021-11-18 02:00:00	-1.9	80	1	1	302	northwest wind	959	8.8	0	5
2021-11-18 03:00:00	-2.3	79	1.5	1	217	southwest wind	960	8.8	0	74
2021-11-18 04:00:00	-2.8	82	0.1	0	326	northwest wind	960	8.8	0	54
2021-11-18 05:00:00	-3.5	80	2.3	2	295	northwest wind	961	10.3	0	22
2021-11-18 06:00:00	-3	78	2.2	2	310	northwest wind	961	10	0	48
2021-11-18 07:00:00	-3.6	79	1.2	1	330	northwest wind	961	10	0	100
2021-11-18 08:00:00	-4.5	80	0	0	297	northwest wind	963	10	0	41

2021-11-18 09:00:00	-5.5	84	2.1	2	229	southwest wind	963	4.6	0	0
2021-11-18 10:00:00	-5.1	84	2.5	2	297	northwest wind	965	4.8	0	2
2021-11-18 11:00:00	-4.1	81	0.2	0	224	southwest wind	965	4.7	0	0
2021-11-18 12:00:00	-0.1	66	1	1	229	southwest wind	965	4.7	0	0
2021-11-18 13:00:00	1.7	52	2.4	2	232	southwest wind	965	7	0	0
2021-11-18 14:00:00	2.5	44	0.7	1	223	southwest wind	965	13.8	0	0
2021-11-18 15:00:00	3.2	38	2.9	2	138	southeast wind	965	12.7	0	0
2021-11-18 16:00:00	2.8	38	0.5	1	152	southeast wind	964	12.7	0	0
2021-11-18 17:00:00	2.4	40	3.1	2	147	southeast wind	964	13.6	0	2
2021-11-18 18:00:00	1.6	44	2	2	126	southeast wind	964	14.4	0	54
2021-11-18 19:00:00	-0.3	47	3.7	3	244	southwest wind	965	10.8	0	90
2021-11-18 20:00:00	-1.5	47	4	3	114	southeast wind	965	10.8	0	90
2021-11-18 21:00:00	-2	45	1.1	1	233	southwest wind	966	17.7	0	100
2021-11-18 22:00:00	-2.4	47	2.5	2	142	southeast wind	966	22.4	0	83
2021-11-18 23:00:00	-3.3	52	1.9	2	8	north wind	965	17	0	100
2021-11-19 00:00:00	-4.8	58	1.6	2	11	north wind	965	17	0	0
2021-11-19 01:00:00	-6	64	0	0	324	northwest wind	964	12.1	0	22
2021-11-19 02:00:00	-6.5	68	0.8	1	329	northwest wind	964	13.2	0	50
2021-11-19 03:00:00	-6.7	67	1.9	2	246	southwest wind	964	20.6	0	1
2021-11-19 04:00:00	-7.4	70	0.7	1	300	northwest wind	963	20.6	0	59
2021-11-19 05:00:00	-6.4	67	1.6	2	305	northwest wind	963	20.3	0	90
2021-11-19 06:00:00	-6	70	0.1	0	326	northwest wind	963	20.3	0	90
2021-11-19 07:00:00	-5.8	72	0.2	0	328	northwest wind	963	20.3	0	100
2021-11-19 08:00:00	-5.4	68	1.7	2	316	northwest wind	964	30	0	100
2021-11-19 09:00:00	-5.2	69	3.2	2	217	southwest wind	964	13.5	0	100
2021-11-19 10:00:00	-4.7	66	1.9	2	334	northwest wind	965	13	0	90
2021-11-19 11:00:00	-3.5	64	3	2	320	northwest wind	966	14	0	100
2021-11-19 12:00:00	-2.2	59	2.6	2	295	northwest wind	967	14	0	72
2021-11-19 13:00:00	-0.8	54	2.9	2	212	southwest wind	967	15.8	0	70
2021-11-19 14:00:00	0.4	40	1.6	2	141	southeast wind	967	21.3	0	0
2021-11-19 15:00:00	1.3	39	1.5	1	216	southwest wind	967	20	0	0
2021-11-19 16:00:00	1.2	42	0.3	1	125	southeast wind	967	20	0	1
2021-11-19 17:00:00	0.3	46	1	1	123	southeast wind	968	21.1	0	37
2021-11-19 18:00:00	-0.1	48	2.2	2	133	southeast wind	969	21.4	0	56
2021-11-19 20:00:00	-2.9	66	0.6	1	215	southwest wind	971	22	0	0
2021-11-19 21:00:00	-3.4	69	5.1	3	137	southeast wind	972	22.1	0	0
2021-11-19 22:00:00	-4.2	74	3.9	3	142	southeast wind	973	26	0	6
2021-11-19 23:00:00	-4.5	74	2.5	2	189	south wind	974	30	0	0

2021-11-20 00:00:00	-5.2	76	1.9	2	223	southwest wind	975	30	0	0
2021-11-20 01:00:00	-5.6	76	2.4	2	326	northwest wind	975	30	0	0
2021-11-20 02:00:00	-6.8	80	1.1	1	152	southeast wind	975	28.1	0	0
2021-11-20 03:00:00	-6.3	74	2.8	2	207	southwest wind	975	30	0	0
2021-11-20 04:00:00	-7.3	78	0.5	1	299	northwest wind	975	30	0	0
2021-11-20 05:00:00	-8.3	80	0.8	1	23	northeast wind	975	30	0	0
2021-11-20 06:00:00	-8.5	80	1	1	297	northwest wind	975	30	0	0
2021-11-20 07:00:00	-9.8	82	3.1	2	337	northwest wind	975	30	0	0
2021-11-20 08:00:00	-9.7	86	1.3	1	270	west wind	975	30	0	0
2021-11-20 09:00:00	-10.3	86	2.6	2	314	northwest wind	975	11.8	0	0
2021-11-20 10:00:00	-9.5	85	1.3	1	306	northwest wind	975	11.8	0	0
2021-11-20 11:00:00	-7.2	75	1.5	1	298	northwest wind	976	30	0	0
2021-11-20 12:00:00	-4.1	59	1.7	2	311	northwest wind	976	15.6	0	0
2021-11-20 13:00:00	-2.8	54	0.9	1	128	southeast wind	976	30	0	0
2021-11-20 14:00:00	-2.6	57	2	2	58	northeast wind	975	26.6	0	0
2021-11-20 15:00:00	-2.3	53	0.6	1	155	southeast wind	975	5.5	0	0
2021-11-20 16:00:00	-1.7	50	3	2	127	southeast wind	974	5.5	0	0
2021-11-20 17:00:00	-3.1	62	3	2	127	southeast wind	974	18	0	0
2021-11-20 18:00:00	-3.9	60	3	2	105	east wind	974	23.9	0	0
2021-11-20 19:00:00	-4.9	61	1.6	2	115	southeast wind	974	23.8	0	0
2021-11-20 20:00:00	-5.4	63	3.1	2	142	southeast wind	974	23.8	0	72
2021-11-20 21:00:00	-5.5	63	1.7	2	40	northeast wind	975	24.3	0	83
2021-11-20 22:00:00	-5.6	62	0.8	1	143	southeast wind	975	24.3	0	68
2021-11-20 23:00:00	-5.7	64	3.1	2	27	northeast wind	974	30	0	64
2021-11-21 00:00:00	-5.8	65	0.8	1	28	northeast wind	974	30	0	64
2021-11-21 01:00:00	-5.8	65	2.8	2	307	northwest wind	974	18.1	0	67
2021-11-21 02:00:00	-5.9	61	1.3	1	23	northeast wind	973	30	0	63
2021-11-21 03:00:00	-6.2	61	2.1	2	35	northeast wind	973	30	0	62
2021-11-21 04:00:00	-6.4	62	2.4	2	49	northeast wind	972	30	0	64
2021-11-21 05:00:00	-6.6	61	2.1	2	25	northeast wind	972	30	0	61
2021-11-21 06:00:00	-6.9	64	1	1	37	northeast wind	971	29.3	0	56
2021-11-21 07:00:00	-7.2	66	2.8	2	40	northeast wind	971	29	0	65
2021-11-21 08:00:00	-7.2	63	3.2	2	302	northwest wind	971	29	0	68
2021-11-21 09:00:00	-7	62	1.2	1	38	northeast wind	971	30	0	62
2021-11-21 10:00:00	-7.1	65	2.7	2	66	northeast wind	971	24.8	0	5
2021-11-21 11:00:00	-6.6	64	2.6	2	38	northeast wind	971	21.6	0	0
2021-11-21 12:00:00	-6	64	2.3	2	43	northeast wind	970	21.6	0	0
2021-11-21 13:00:00	-5.4	63	2.3	2	57	northeast wind	970	27.2	0	0

2021-11-21 14:00:00	-4.7	60	0	0	67	northeast wind	969	26.5	0	0
2021-11-21 15:00:00	-4.1	56	1.9	2	54	northeast wind	968	28.1	0	0
2021-11-21 16:00:00	-3.7	56	3.1	2	48	northeast wind	967	28.1	0	0
2021-11-21 17:00:00	-3.8	56	1.9	2	337	northwest wind	967	22.3	0	0
2021-11-21 18:00:00	-4.2	56	2.1	2	300	northwest wind	967	22.8	0	0
2021-11-21 19:00:00	-5.3	60	0.3	1	62	northeast wind	967	22.1	0	0
2021-11-21 20:00:00	-6.5	69	0.7	1	20	north wind	967	22.1	0	0
2021-11-21 21:00:00	-8	73	0.6	1	333	northwest wind	967	7.7	0	0
2021-11-21 22:00:00	-9.3	77	1.8	2	312	northwest wind	967	7.7	0	0
2021-11-21 23:00:00	-10.3	80	2.9	2	329	northwest wind	967	7.1	0	0
2021-11-22 00:00:00	-10	81	3.1	2	304	northwest wind	966	7.1	0	0
2021-11-22 01:00:00	-10	80	2.2	2	297	northwest wind	966	10.2	0	0
2021-11-22 02:00:00	-10.1	80	0	0	307	northwest wind	965	11.5	0	0
2021-11-22 03:00:00	-10.3	79	1.5	1	330	northwest wind	965	12.5	0	0
2021-11-22 04:00:00	-10	79	2.4	2	302	northwest wind	965	12.5	0	0
2021-11-22 05:00:00	-9.3	75	0.2	0	318	northwest wind	965	12.5	0	0
2021-11-22 06:00:00	-9.2	71	2	2	310	northwest wind	965	25.5	0	0
2021-11-22 07:00:00	-9.6	71	1.8	2	307	northwest wind	964	26.8	0	0
2021-11-22 08:00:00	-9.7	70	2.3	2	322	northwest wind	965	26.8	0	0
2021-11-22 09:00:00	-10.4	69	1.8	2	316	northwest wind	965	12.6	0	83
2021-11-22 10:00:00	-9.7	65	2.2	2	322	northwest wind	965	16.4	0	22
2021-11-22 11:00:00	-8.1	63	1.3	1	332	northwest wind	965	13.8	0	22
2021-11-22 12:00:00	-5.2	55	1.7	2	314	northwest wind	964	13.8	0	41
2021-11-22 13:00:00	-3	49	0.8	1	85	east wind	964	16.5	0	40
2021-11-22 14:00:00	-1.3	37	0.8	1	114	southeast wind	963	17.2	0	23
2021-11-22 15:00:00	-1.3	39	2.6	2	48	northeast wind	963	20.2	0	40
2021-11-22 16:00:00	-1.1	40	1.3	1	34	northeast wind	963	20.2	0	18
2021-11-22 17:00:00	-2	47	3.1	2	127	southeast wind	964	21	0	55
2021-11-22 18:00:00	-3.2	55	1.1	1	126	southeast wind	965	16.8	0	100
2021-11-22 19:00:00	-3.8	58	0.5	1	128	southeast wind	965	13.3	0	100
2021-11-22 20:00:00	-4.5	59	1.7	2	134	southeast wind	966	13.3	0	100
2021-11-22 21:00:00	-5.5	64	2.7	2	28	northeast wind	967	10.4	0	100
2021-11-22 22:00:00	-6.2	67	1.7	2	23	northeast wind	967	15.2	0	64
2021-11-22 23:00:00	-7.1	70	0.9	1	302	northwest wind	967	7.4	0	59
2021-11-23 00:00:00	-7.7	73	1.5	1	337	northwest wind	967	7.4	0	64
2021-11-23 01:00:00	-7.9	73	2.6	2	299	northwest wind	966	10.4	0	61
2021-11-23 02:00:00	-8.2	77	2.1	2	209	southwest wind	965	9.9	0	64
2021-11-23 03:00:00	-8.9	76	2.2	2	119	southeast wind	965	9.5	0	7

2021-11-23 04:00:00	-9.1	77	1.9	2	321	northwest wind	965	9.5	0	13
2021-11-23 05:00:00	-9.3	78	2	2	302	northwest wind	964	9.5	0	0
2021-11-23 06:00:00	-9.8	79	0	0	319	northwest wind	964	8.8	0	62
2021-11-23 07:00:00	-9.6	79	2.4	2	301	northwest wind	964	7.9	0	49
2021-11-23 08:00:00	-10.3	82	0.7	1	311	northwest wind	965	7.9	0	62
2021-11-23 09:00:00	-10.1	81	0.5	1	322	northwest wind	965	6.5	0	100
2021-11-23 10:00:00	-10.3	82	2.8	2	324	northwest wind	966	5.4	0	90
2021-11-23 11:00:00	-8.9	79	0.8	1	293	northwest wind	967	7	0	88
2021-11-23 12:00:00	-5.6	67	1.9	2	67	northeast wind	967	7	0	17
2021-11-23 13:00:00	-3.6	56	2.2	2	58	northeast wind	966	10.5	0	0
2021-11-23 14:00:00	-2.1	48	2.3	2	55	northeast wind	965	18.1	0	2
2021-11-23 15:00:00	-1.1	44	2.9	2	117	southeast wind	964	19.9	0	0
2021-11-23 16:00:00	-1.1	45	2.1	2	154	southeast wind	963	19.9	0	16
2021-11-23 17:00:00	-0.6	43	1.7	2	134	southeast wind	962	18.6	0	20
2021-11-23 18:00:00	-1.7	48	3	2	147	southeast wind	962	17.6	0	32
2021-11-23 19:00:00	-2.8	50	0.2	0	129	southeast wind	962	17.3	0	68
2021-11-23 20:00:00	-4.2	56	2.1	2	131	southeast wind	962	17.3	0	65
2021-11-23 21:00:00	-5.1	58	1.7	2	20	north wind	962	9.7	0	61
2021-11-23 22:00:00	-6.1	63	0.9	1	44	northeast wind	962	10.2	0	100
2021-11-23 23:00:00	-7.2	66	3.2	2	301	northwest wind	962	8.2	0	90
2021-11-24 00:00:00	-8.3	71	0.3	1	335	northwest wind	962	8.2	0	72
2021-11-24 01:00:00	-8.6	73	2.8	2	320	northwest wind	961	7.5	0	69
2021-11-24 02:00:00	-8.6	73	2.7	2	300	northwest wind	961	6.8	0	0
2021-11-24 03:00:00	-9.4	76	1.9	2	328	northwest wind	961	9	0	19
2021-11-24 04:00:00	-9.4	79	2.3	2	207	southwest wind	961	9	0	0
2021-11-24 05:00:00	-9.2	78	0	0	312	northwest wind	961	8	0	0
2021-11-24 06:00:00	-9.2	77	3	2	310	northwest wind	960	8.3	0	0
2021-11-24 07:00:00	-10.2	78	1.5	1	298	northwest wind	960	8.3	0	0
2021-11-24 08:00:00	-10.8	81	1.1	1	313	northwest wind	960	8.3	0	0
2021-11-24 09:00:00	-11.3	82	1	1	221	southwest wind	961	6.9	0	0
2021-11-24 10:00:00	-10.2	82	0.8	1	326	northwest wind	961	4.7	0	0
2021-11-24 11:00:00	-7.4	74	0.9	1	302	northwest wind	961	3.7	0	0
2021-11-24 12:00:00	-3.7	62	0.3	1	309	northwest wind	961	3.7	0	0
2021-11-24 13:00:00	-0.8	53	1.4	1	116	southeast wind	961	12.7	0	0
2021-11-24 14:00:00	1.2	47	2.3	2	130	southeast wind	960	14.1	0	0
2021-11-24 15:00:00	2.2	42	1.5	1	127	southeast wind	960	14.4	0	0
2021-11-24 16:00:00	2.6	42	2.3	2	123	southeast wind	960	14.4	0	0
2021-11-24 17:00:00	2.3	41	1.8	2	128	southeast wind	960	12.7	0	0

2021-11-24 18:00:00	1.8	42	1.8	2	39	northeast wind	960	12.2	0	0
2021-11-24 19:00:00	-0.9	51	0.1	0	70	east wind	960	11	0	0
2021-11-24 20:00:00	-2.9	57	0.9	1	23	northeast wind	961	11	0	0
2021-11-24 21:00:00	-4.1	62	2.5	2	42	northeast wind	961	6.6	0	0
2021-11-24 22:00:00	-4.5	62	2.7	2	35	northeast wind	961	8	0	0
2021-11-24 23:00:00	-5.7	67	1.6	2	31	northeast wind	961	7.6	0	0
2021-11-25 00:00:00	-6.5	69	0.3	1	332	northwest wind	961	5.9	0	0
2021-11-25 01:00:00	-7.5	72	3.1	2	235	southwest wind	961	7.1	0	0
2021-11-25 02:00:00	-7.2	76	1.1	1	311	northwest wind	961	6.3	0	0
2021-11-25 03:00:00	-8.1	77	2.1	2	29	northeast wind	961	7.1	0	0
2021-11-25 04:00:00	-8.6	79	1.1	1	306	northwest wind	961	7.1	0	0
2021-11-25 05:00:00	-9.1	79	1.9	2	320	northwest wind	961	7.5	0	0
2021-11-25 06:00:00	-9.4	79	2.7	2	203	southwest wind	960	7.1	0	0
2021-11-25 07:00:00	-9.3	80	0	0	333	northwest wind	960	7.4	0	0
2021-11-25 08:00:00	-9.3	80	1.6	2	293	northwest wind	961	7.4	0	0
2021-11-25 09:00:00	-9.8	79	1.5	1	295	northwest wind	961	6.2	0	0
2021-11-25 10:00:00	-9.9	82	3	2	287	west wind	962	4.8	0	0
2021-11-25 11:00:00	-7.2	74	2.8	2	234	southwest wind	962	5.1	0	0
2021-11-25 12:00:00	-8.1	57	1.8	2	144	southeast wind	962	5.1	0	0
2021-11-25 13:00:00	-0.3	49	3.2	2	227	southwest wind	961	10.6	0	0
2021-11-25 14:00:00	1.5	42	0	0	120	southeast wind	960	10.9	0	0
2021-11-25 15:00:00	1.9	39	1.4	1	148	southeast wind	959	10.4	0	0
2021-11-25 16:00:00	1.8	40	2.3	2	152	southeast wind	959	10.4	0	0
2021-11-25 17:00:00	2.1	42	0.7	1	145	southeast wind	959	10.5	0	0
2021-11-25 18:00:00	1.2	45	1.6	2	122	southeast wind	959	10.4	0	0
2021-11-25 19:00:00	-1.1	52	1.8	2	237	southwest wind	959	8.5	0	45
2021-11-25 20:00:00	-2.8	60	0.1	0	323	northwest wind	959	8.5	0	63
2021-11-25 21:00:00	-3.7	62	0.7	1	309	northwest wind	959	2.8	0	68
2021-11-25 22:00:00	-4.2	62	1.3	1	84	east wind	958	4.7	0	100
2021-11-25 23:00:00	-5.2	66	1.1	1	320	northwest wind	958	4	0	100
2021-11-26 00:00:00	-5.6	68	1.9	2	295	northwest wind	958	4	0	100
2021-11-26 01:00:00	-5.3	67	0.6	1	314	northwest wind	957	5.5	0	90
2021-11-26 02:00:00	-5.5	68	0.5	1	284	west wind	957	6.1	0	100
2021-11-26 03:00:00	-4.9	67	0.3	1	326	northwest wind	956	6.4	0	100
2021-11-26 04:00:00	-5	69	2.8	2	27	northeast wind	955	6.4	0	100
2021-11-26 05:00:00	-5.5	72	0.8	1	298	northwest wind	955	6.4	0	100
2021-11-26 06:00:00	-5.8	74	1.3	1	32	northeast wind	955	6	0	100
2021-11-26 07:00:00	-5.8	74	1.3	1	240	southwest wind	956	6.3	0	100

2021-11-26 08:00:00	-6	74	0.6	1	308	northwest wind	956	6.3	0	100
2021-11-26 09:00:00	-6	76	3	2	209	southwest wind	957	4.5	0	100
2021-11-26 10:00:00	-5	73	0.7	1	35	northeast wind	958	7.5	0	100
2021-11-26 11:00:00	-4	71	1.7	2	222	southwest wind	959	5	0	100
2021-11-26 12:00:00	-2.2	65	1.9	2	311	northwest wind	960	5	0	100
2021-11-26 13:00:00	-0.3	58	0.2	0	318	northwest wind	960	8.2	0	90
2021-11-26 14:00:00	2	51	1.2	1	246	southwest wind	959	10.3	0	83
2021-11-26 15:00:00	3.1	49	0.3	1	213	southwest wind	959	10.4	0	62
2021-11-26 16:00:00	3.9	58	2.9	2	156	southeast wind	960	10.4	0	62
2021-11-26 17:00:00	3.5	60	2.3	2	141	southeast wind	961	13	0	64
2021-11-26 18:00:00	3.1	61	0.3	1	120	southeast wind	962	12.4	0	90
2021-11-26 19:00:00	2	62	2.3	2	116	southeast wind	963	11.9	0	100
2021-11-26 20:00:00	0.3	70	1	1	137	southeast wind	965	11.9	0	100
2021-11-26 21:00:00	-0.3	71	0.9	1	123	southeast wind	966	16.8	0	1
2021-11-26 22:00:00	-1	71	2.5	2	135	southeast wind	967	24.4	0	22
2021-11-26 23:00:00	-1.5	71	2.1	2	124	southeast wind	967	23	0	21
2021-11-27 00:00:00	-2	73	3.7	3	121	southeast wind	968	30	0	2
2021-11-27 01:00:00	-2.4	75	2	2	133	southeast wind	969	30	0	6
2021-11-27 02:00:00	-2.1	72	1.6	2	218	southwest wind	970	30	0	1
2021-11-27 03:00:00	-2.2	69	1.2	1	118	southeast wind	970	30	0	4
2021-11-27 04:00:00	-2.4	69	0.7	1	137	southeast wind	971	30	0	21
2021-11-27 05:00:00	-2.8	70	2.7	2	157	southeast wind	972	30	0	14
2021-11-27 06:00:00	-3	70	2.8	2	147	southeast wind	972	30	0	72
2021-11-27 07:00:00	-3.3	72	1.9	2	113	southeast wind	974	30	0	100
2021-11-27 08:00:00	-3.6	73	0.1	0	204	southwest wind	975	30	0	100
2021-11-27 09:00:00	-4.2	87	0.6	1	332	northwest wind	976	4.5	0	100
2021-11-27 10:00:00	-4.1	91	0.8	1	312	northwest wind	977	1.5	0.8	100
2021-11-27 11:00:00	-3.5	89	1.9	2	220	southwest wind	978	1.9	0.1	40
2021-11-27 12:00:00	-2.8	84	1.5	1	322	northwest wind	979	1.9	0.4	2
2021-11-27 13:00:00	-2.5	77	0.6	1	325	northwest wind	979	20.8	0	46
2021-11-27 14:00:00	-0.6	69	3.2	2	290	west wind	978	30	0	0
2021-11-27 15:00:00	1	60	0	0	337	northwest wind	978	30	0	0
2021-11-27 16:00:00	1.9	52	1.9	2	213	southwest wind	978	30	0	0
2021-11-27 17:00:00	2.4	50	2.6	2	229	southwest wind	978	30	0	0
2021-11-27 18:00:00	0.7	64	0.7	1	41	northeast wind	978	30	0	0
2021-11-27 19:00:00	-1.9	69	0.2	0	207	southwest wind	979	30	0	0
2021-11-27 20:00:00	-3.9	72	1.9	2	328	northwest wind	979	30	0	0
2021-11-27 21:00:00	-5.9	79	2.2	2	221	southwest wind	979	12.6	0	0

2021-11-27 22:00:00	-6.3	79	0.8	1	314	northwest wind	979	9.7	0	0
2021-11-27 23:00:00	-6.7	75	1	1	233	southwest wind	979	19.5	0	0
2021-11-28 00:00:00	-7	71	0.4	1	296	northwest wind	979	19.5	0	0
2021-11-28 01:00:00	-7.6	72	2.8	2	256	west wind	979	30	0	0
2021-11-28 02:00:00	-8.2	72	2.8	2	313	northwest wind	978	30	0	0
2021-11-28 03:00:00	-8.3	72	1.9	2	293	northwest wind	978	30	0	0
2021-11-28 04:00:00	-9.3	74	2.1	2	332	northwest wind	978	30	0	0
2021-11-28 05:00:00	-10.4	79	2.7	2	326	northwest wind	978	30	0	0
2021-11-28 06:00:00	-10.7	80	0.3	1	329	northwest wind	978	30	0	0
2021-11-28 07:00:00	-10.9	78	2.2	2	328	northwest wind	979	30	0	0
2021-11-28 08:00:00	-11.2	78	1.7	2	299	northwest wind	979	30	0	0
2021-11-28 09:00:00	-11.5	79	2.8	2	301	northwest wind	979	17.5	0	0
2021-11-28 10:00:00	-11.4	82	1.9	2	295	northwest wind	979	11.1	0	0
2021-11-28 11:00:00	-9.9	79	0	0	322	northwest wind	979	12.5	0	0
2021-11-28 12:00:00	-6.7	76	0.4	1	333	northwest wind	979	12.5	0	0
2021-11-28 13:00:00	-4.2	70	1.4	1	310	northwest wind	979	19.1	0	0
2021-11-28 14:00:00	-2.6	63	1.5	1	43	northeast wind	978	22.3	0	0
2021-11-28 15:00:00	-2.6	62	1.7	2	35	northeast wind	977	26.1	0	0
2021-11-28 16:00:00	-2	62	0.8	1	42	northeast wind	977	26.1	0	0
2021-11-28 17:00:00	-2.3	67	0.5	1	59	northeast wind	976	20.2	0	0
2021-11-28 18:00:00	-3	68	2.1	2	48	northeast wind	977	21.3	0	0
2021-11-28 19:00:00	-4.9	72	0.2	0	130	southeast wind	977	20.5	0	0
2021-11-28 20:00:00	-6.7	81	2.2	2	56	northeast wind	977	20.5	0	0
2021-11-28 21:00:00	-7.6	83	0.6	1	303	northwest wind	977	14	0	0
2021-11-28 22:00:00	-9.2	86	2.8	2	298	northwest wind	977	7.2	0	0
2021-11-28 23:00:00	-10.5	86	1.3	1	297	northwest wind	977	9.6	0	0
2021-11-29 00:00:00	-10.8	88	0.1	0	294	northwest wind	977	9.6	0	0
2021-11-29 01:00:00	-11.3	84	3	2	323	northwest wind	977	8.5	0	0
2021-11-29 02:00:00	-11.6	84	1.1	1	315	northwest wind	976	9.8	0	0
2021-11-29 03:00:00	-11.7	84	2.4	2	309	northwest wind	975	11.2	0	0
2021-11-29 04:00:00	-12.2	84	1.7	2	294	northwest wind	974	11.2	0	0
2021-11-29 05:00:00	-13.1	83	2.5	2	335	northwest wind	974	14	0	0
2021-11-29 06:00:00	-12.8	84	1.5	1	306	northwest wind	973	14.3	0	0
2021-11-29 07:00:00	-13	83	0.1	0	294	northwest wind	973	12.9	0	0
2021-11-29 08:00:00	-12.3	83	1.1	1	334	northwest wind	973	12.9	0	0
2021-11-29 09:00:00	-12.5	80	2.1	2	296	northwest wind	973	14.1	0	0
2021-11-29 10:00:00	-13.2	82	0.3	1	326	northwest wind	973	14.1	0	0
2021-11-29 11:00:00	-11.2	82	2.5	2	313	northwest wind	973	11.9	0	0

2021-11-29 12:00:00	-9.2	93	1.2	1	329	northwest wind	973	11.9	0	0
2021-11-29 13:00:00	-6.1	87	1.4	1	325	northwest wind	972	5.7	0	0
2021-11-29 14:00:00	-3.2	69	1.5	1	299	northwest wind	971	14.4	0	0
2021-11-29 15:00:00	-1.3	55	3.1	2	119	southeast wind	970	21.1	0	0
2021-11-29 16:00:00	-2	58	3	2	42	northeast wind	970	21.1	0	0
2021-11-29 17:00:00	-1.9	56	0.6	1	137	southeast wind	970	18.9	0	0
2021-11-29 18:00:00	-2.6	59	0.5	1	130	southeast wind	970	19.9	0	0
2021-11-29 19:00:00	-4.5	70	3	2	135	southeast wind	970	11.7	0	0
2021-11-29 20:00:00	-6.6	78	0.3	1	329	northwest wind	970	11.7	0	0
2021-11-29 21:00:00	-8.5	82	0.7	1	303	northwest wind	970	5.6	0	0
2021-11-29 22:00:00	-10.3	83	2.2	2	307	northwest wind	970	4.6	0	0
2021-11-29 23:00:00	-11.1	84	2.7	2	304	northwest wind	969	4.7	0	0
2021-11-30 00:00:00	-12	84	3.2	2	312	northwest wind	969	4.7	0	0
2021-11-30 01:00:00	-12.4	82	0.1	0	330	northwest wind	969	7.2	0	0
2021-11-30 02:00:00	-12.5	78	2.6	2	301	northwest wind	968	14.7	0	0
2021-11-30 03:00:00	-12.7	80	1.4	1	324	northwest wind	968	23.5	0	0
2021-11-30 04:00:00	-10.6	73	1.2	1	329	northwest wind	968	23.5	0	0
2021-11-30 05:00:00	-11.8	73	2.3	2	318	northwest wind	968	30	0	0
2021-11-30 06:00:00	-12.6	77	1.2	1	308	northwest wind	967	30	0	0
2021-11-30 07:00:00	-13.3	77	1.8	2	308	northwest wind	967	29.4	0	0
2021-11-30 08:00:00	-14	80	1.1	1	253	west wind	968	29.4	0	0
2021-11-30 09:00:00	-14.3	80	0.6	1	308	northwest wind	968	15.3	0	0
2021-11-30 10:00:00	-13.7	80	1.4	1	332	northwest wind	968	8.9	0	0
2021-11-30 11:00:00	-10.7	77	1.6	2	297	northwest wind	969	3.4	0	0
2021-11-30 12:00:00	-5.1	68	0.9	1	33	northeast wind	969	11.4	0	0
2021-11-30 13:00:00	-4.1	64	1.6	2	62	northeast wind	969	12.2	0	0
2021-11-30 14:00:00	-2.8	54	3	2	28	northeast wind	969	15.7	0	0
2021-11-30 15:00:00	-2	54	0.3	1	142	southeast wind	968	17.4	0	0
2021-11-30 16:00:00	-1.5	53	2.8	2	30	northeast wind	968	15.9	0	0
2021-11-30 17:00:00	-0.9	48	2.5	2	143	southeast wind	968	19.7	0	0
2021-11-30 18:00:00	-2.1	55	1	1	132	southeast wind	969	19.5	0	0
2021-11-30 19:00:00	-3.8	62	1.5	1	45	northeast wind	969	16.4	0	0
2021-11-30 20:00:00	-6.4	73	3.1	2	141	southeast wind	970	9.6	0	0
2021-11-30 21:00:00	-8.1	79	2.5	2	321	northwest wind	970	7.3	0	0
2021-11-30 22:00:00	-10.1	77	1.7	2	329	northwest wind	970	4.9	0	0
2021-11-30 23:00:00	-11	83	2.1	2	295	northwest wind	970	5.1	0	0
2021-12-01 00:00:00	-11.4	81	3.1	2	319	northwest wind	970	5	0	0
2021-12-01 01:00:00	-12.1	81	0.4	1	328	northwest wind	970	7.9	0	0

2021-12-01 02:00:00	-12.5	81	2.7	2	302	northwest wind	970	9.5	0	0
2021-12-01 03:00:00	-12.8	80	2.3	2	295	northwest wind	969	10.4	0	0
2021-12-01 04:00:00	-13.1	81	2.4	2	320	northwest wind	969	10.2	0	0
2021-12-01 05:00:00	-13.3	80	0.6	1	299	northwest wind	970	12.4	0	8
2021-12-01 06:00:00	-14.1	81	2.4	2	325	northwest wind	970	11.2	0	0
2021-12-01 07:00:00	-14.8	84	0	0	328	northwest wind	970	8.4	0	0
2021-12-01 08:00:00	-14.9	83	2.3	2	332	northwest wind	970	7.7	0	0
2021-12-01 09:00:00	-14.5	83	0.7	1	327	northwest wind	970	5	0	21
2021-12-01 10:00:00	-14.2	84	2.9	2	305	northwest wind	971	3.7	0	62
2021-12-01 11:00:00	-12	82	1.3	1	213	southwest wind	971	4.8	0	0
2021-12-01 12:00:00	-10	80	2.8	2	212	southwest wind	971	4.2	0	70
2021-12-01 13:00:00	-6.4	74	2.4	2	146	southeast wind	971	4.4	0	27
2021-12-01 14:00:00	-5.4	67	2	2	134	southeast wind	970	4.6	0	0
2021-12-01 15:00:00	-4.8	63	2.7	2	129	southeast wind	970	5	0	0
2021-12-01 16:00:00	-3.9	59	2.6	2	127	southeast wind	969	5	0	0
2021-12-01 17:00:00	-4.6	63	1.8	2	60	northeast wind	969	5.8	0	0
2021-12-01 18:00:00	-5.4	65	1.9	2	144	southeast wind	969	8.1	0	0
2021-12-01 19:00:00	-6.6	70	1.8	2	47	northeast wind	969	7.5	0	0
2021-12-01 20:00:00	-8.5	78	1.5	1	66	northeast wind	969	5.3	0	0
2021-12-01 21:00:00	-10.2	80	2.9	2	295	northwest wind	969	3.3	0	0
2021-12-01 22:00:00	-11.4	81	2.6	2	321	northwest wind	969	3.4	0	0
2021-12-01 23:00:00	-12.4	83	0.3	1	331	northwest wind	969	1.6	0	0
2021-12-02 00:00:00	-12.5	84	1.6	2	333	northwest wind	968	2.6	0	0
2021-12-02 01:00:00	-12.6	83	1.1	1	328	northwest wind	968	3.2	0	0
2021-12-02 02:00:00	-11.9	82	0.5	1	314	northwest wind	967	3.8	0	0
2021-12-02 03:00:00	-11.4	81	0.7	1	324	northwest wind	966	4.3	0	0
2021-12-02 04:00:00	-10.8	79	1.8	2	302	northwest wind	966	4.9	0	0
2021-12-02 05:00:00	-10.9	78	0.1	0	305	northwest wind	965	6.5	0	0
2021-12-02 06:00:00	-11.2	78	0.6	1	316	northwest wind	965	7.5	0	28
2021-12-02 07:00:00	-11.7	73	1.2	1	294	northwest wind	965	13.5	0	8
2021-12-02 08:00:00	-12.3	77	1.6	2	316	northwest wind	965	12.8	0	12
2021-12-02 09:00:00	-12.9	77	1.6	2	23	northeast wind	964	12.1	0	68
2021-12-02 10:00:00	-13	78	1.1	1	300	northwest wind	965	8.5	0	100
2021-12-02 11:00:00	-11.4	82	0.8	1	357	north wind	965	7	0	0
2021-12-02 12:00:00	-7.2	73	2.1	2	57	northeast wind	965	5.3	0	0
2021-12-02 13:00:00	-5.8	64	3	2	127	southeast wind	965	6.7	0	15
2021-12-02 14:00:00	-4.2	59	2.4	2	154	southeast wind	964	6.8	0	0
2021-12-02 15:00:00	-2.7	54	0.2	0	317	northwest wind	963	8.1	0	0

2021-12-02 16:00:00	-1.6	51	0.4	1	123	southeast wind	963	8.9	0	0
2021-12-02 17:00:00	-1.8	52	2.5	2	155	southeast wind	964	8.2	0	0
2021-12-02 18:00:00	-2.9	56	0.7	1	157	southeast wind	964	9.4	0	0
2021-12-02 19:00:00	-4.4	63	1	1	140	southeast wind	964	7.4	0	0
2021-12-02 20:00:00	-6.4	71	2.8	2	296	northwest wind	965	5.5	0	0
2021-12-02 21:00:00	-8.4	76	2.6	2	309	northwest wind	965	3.4	0	0
2021-12-02 22:00:00	-9.6	78	1.8	2	295	northwest wind	965	3.6	0	0
2021-12-02 23:00:00	-10.6	82	0.8	1	308	northwest wind	965	3.6	0	0
2021-12-03 00:00:00	-10.8	80	0.5	1	312	northwest wind	964	4.7	0	0
2021-12-03 01:00:00	-10.9	78	2.1	2	313	northwest wind	964	4.4	0	0
2021-12-03 02:00:00	-11.2	79	2.4	2	294	northwest wind	964	5.8	0	0
2021-12-03 03:00:00	-11.6	79	2.9	2	312	northwest wind	964	6.5	0	0
2021-12-03 04:00:00	-12.5	79	1.7	2	0	north wind	964	6.8	0	0
2021-12-03 05:00:00	-12.5	82	1.9	2	334	northwest wind	965	6.1	0	0
2021-12-03 06:00:00	-12.5	82	1.1	1	317	northwest wind	964	6.1	0	0
2021-12-03 07:00:00	-12.5	81	0	0	333	northwest wind	964	6.7	0	0
2021-12-03 08:00:00	-13.6	80	0.8	1	320	northwest wind	965	6.4	0	0
2021-12-03 09:00:00	-14.1	83	1	1	307	northwest wind	965	4.2	0	0
2021-12-03 10:00:00	-14.2	83	0.2	0	204	southwest wind	965	3.9	0	0
2021-12-03 11:00:00	-12	79	2.3	2	224	southwest wind	966	4.5	0	0
2021-12-03 12:00:00	-8.5	74	1.6	2	230	southwest wind	966	4.5	0	0
2021-12-03 13:00:00	-5.2	61	1.4	1	227	southwest wind	965	6.1	0	0
2021-12-03 14:00:00	-4.1	58	2.7	2	136	southeast wind	964	6.5	0	0
2021-12-03 15:00:00	-4	56	1.8	2	41	northeast wind	964	6.8	0	0
2021-12-03 16:00:00	-3.8	57	2.3	2	126	southeast wind	963	6.4	0	0
2021-12-03 17:00:00	-4.1	60	0.7	1	29	northeast wind	964	4.9	0	0
2021-12-03 18:00:00	-4.7	63	1.1	1	81	east wind	964	5.1	0	42
2021-12-03 19:00:00	-6	69	0.7	1	310	northwest wind	965	4.5	0	32
2021-12-03 20:00:00	-7.5	75	0.1	0	40	northeast wind	965	3.3	0	60
2021-12-03 21:00:00	-8.2	77	0	0	303	northwest wind	966	3.2	0	70
2021-12-03 22:00:00	-8.8	78	1.9	2	241	southwest wind	965	2.5	0	90
2021-12-03 23:00:00	-8.8	81	3.1	2	301	northwest wind	965	2.6	0	73
2021-12-04 00:00:00	-7.4	77	0.9	1	293	northwest wind	965	2.6	0	90
2021-12-04 01:00:00	-6.6	75	0.2	0	331	northwest wind	964	2.8	0	100
2021-12-04 02:00:00	-6.6	76	2.7	2	221	southwest wind	965	3.1	0	90
2021-12-04 03:00:00	-6.5	77	0.7	1	311	northwest wind	964	2.9	0	90
2021-12-04 04:00:00	-6.1	77	2.5	2	223	southwest wind	965	3.1	0	76
2021-12-04 05:00:00	-5.8	75	0.9	1	45	northeast wind	965	3.2	0	64

2021-12-04 06:00:00	-6.3	77	1.9	2	295	northwest wind	965	3	0	6
2021-12-04 07:00:00	-6.8	79	0.7	1	318	northwest wind	966	3.1	0	90
2021-12-04 08:00:00	-7.7	82	0.7	1	320	northwest wind	966	2.7	0	100
2021-12-04 09:00:00	-8.2	84	2.6	2	329	northwest wind	967	2.3	0	100
2021-12-04 10:00:00	-8.2	84	0.7	1	319	northwest wind	967	2.4	0	90
2021-12-04 11:00:00	-6.8	80	0	0	222	southwest wind	967	2.5	0	90
2021-12-04 12:00:00	-5.1	76	2.2	2	215	southwest wind	967	3	0	90
2021-12-04 13:00:00	-3.4	65	1.4	1	253	west wind	966	4.3	0	83
2021-12-04 14:00:00	-2.2	62	0.6	1	114	southeast wind	966	4.4	0	64
2021-12-04 15:00:00	-2.3	62	3	2	45	northeast wind	966	3.9	0	69
2021-12-04 16:00:00	-2.4	60	0.9	1	35	northeast wind	966	4.3	0	52
2021-12-04 17:00:00	-2.5	59	2.4	2	124	southeast wind	967	4.6	0	36
2021-12-04 18:00:00	-2.8	60	0.1	0	303	northwest wind	967	4.7	0	65
2021-12-04 19:00:00	-3	62	3.2	2	14	north wind	967	4.3	0	76
2021-12-04 20:00:00	-3.5	65	3.1	2	113	southeast wind	967	3.9	0	35
2021-12-04 21:00:00	-4.7	70	0.8	1	223	southwest wind	968	3.6	0	19
2021-12-04 22:00:00	-5.7	75	3	2	315	northwest wind	967	2.7	0	100
2021-12-04 23:00:00	-5.9	75	0.4	1	67	northeast wind	967	2.8	0	100
2021-12-05 00:00:00	-5.6	71	2.4	2	31	northeast wind	967	3	0	83
2021-12-05 01:00:00	-6.7	75	2.6	2	334	northwest wind	967	3.3	0	67
2021-12-05 02:00:00	-7.8	78	0.4	1	331	northwest wind	967	2.6	0	0
2021-12-05 03:00:00	-8.5	82	2.9	2	311	northwest wind	966	2.4	0	27
2021-12-05 04:00:00	-8.9	82	2.8	2	324	northwest wind	966	2.2	0	63
2021-12-05 05:00:00	-8.6	85	0.8	1	316	northwest wind	966	2	0	90
2021-12-05 06:00:00	-7.9	82	1.9	2	296	northwest wind	965	2.3	0	80
2021-12-05 07:00:00	-8.1	82	2.3	2	318	northwest wind	965	2.4	0	75
2021-12-05 08:00:00	-8.3	85	2.2	2	209	southwest wind	965	2.1	0	0
2021-12-05 09:00:00	-8.6	85	1.2	1	306	northwest wind	965	2.3	0	73
2021-12-05 10:00:00	-7.8	86	1.9	2	331	northwest wind	964	2.3	0	66
2021-12-05 11:00:00	-5.9	80	1.6	2	113	southeast wind	964	2.4	0	40
2021-12-05 12:00:00	-3.6	67	2	2	120	southeast wind	964	3.3	0	24
2021-12-05 13:00:00	-2.8	62	1	1	25	northeast wind	964	3.9	0	19
2021-12-05 14:00:00	-2.5	63	0.2	0	35	northeast wind	963	3.5	0	18
2021-12-05 15:00:00	-1.9	60	1	1	141	southeast wind	962	3.5	0	0
2021-12-05 16:00:00	-1.1	54	1.4	1	208	southwest wind	961	3.8	0	0
2021-12-05 17:00:00	-1.1	55	2.9	2	148	southeast wind	961	4.2	0	22
2021-12-05 18:00:00	-2.5	62	2.2	2	131	southeast wind	961	3.6	0	5
2021-12-05 19:00:00	-4	69	1.7	2	40	northeast wind	961	3	0	40

2021-12-05 20:00:00	-5.1	72	2.2	2	41	northeast wind	961	2.8	0	0
2021-12-05 21:00:00	-6.4	73	1.9	2	332	northwest wind	961	2.4	0	0
2021-12-05 22:00:00	-6.9	78	2.4	2	311	northwest wind	961	2	0	4
2021-12-05 23:00:00	-7.1	78	2.2	2	316	northwest wind	961	1.8	0	68
2021-12-06 00:00:00	-7.1	78	2.2	2	316	northwest wind	961	1.8	0	0
2021-12-06 01:00:00	-7.1	78	2.2	2	316	northwest wind	961	2	0	90
2021-12-06 02:00:00	-7.1	78	2.2	2	316	northwest wind	961	1.8	0	83
2021-12-06 03:00:00	-7.1	78	2.2	2	316	northwest wind	961	2.2	0	68
2021-12-06 04:00:00	-7.1	78	2.2	2	316	northwest wind	961	2.1	0	64
2021-12-06 05:00:00	-7.1	78	2.2	2	316	northwest wind	961	2.3	0	68
2021-12-06 06:00:00	-7.1	78	2.2	2	316	northwest wind	961	2.8	0	0
2021-12-06 07:00:00	-7.1	78	2.2	2	316	northwest wind	961	3	0	17
2021-12-06 08:00:00	-7.1	78	2.2	2	316	northwest wind	961	3	0	49
2021-12-06 09:00:00	-7.1	78	2.2	2	316	northwest wind	961	6	0	77
2021-12-06 10:00:00	-7.1	78	2.2	2	316	northwest wind	961	5	0	0
2021-12-06 11:00:00	-5.7	74	0.7	1	309	northwest wind	961	7.7	0	6
2021-12-06 12:00:00	-2.4	67	1.7	2	323	northwest wind	961	9.9	0	16
2021-12-06 13:00:00	-1.1	58	0.6	1	38	northeast wind	961	8.2	0	14
2021-12-06 14:00:00	-1.6	59	2.2	2	133	southeast wind	961	3.5	0	3
2021-12-06 15:00:00	-0.3	57	2.5	2	144	southeast wind	961	3.8	0	3
2021-12-06 16:00:00	0.3	54	2.4	2	137	southeast wind	960	4.1	0	12
2021-12-06 17:00:00	-0.8	59	2.2	2	149	southeast wind	961	3.8	0	10
2021-12-06 18:00:00	-1.9	63	0.9	1	131	southeast wind	961	3.3	0	62
2021-12-06 19:00:00	-2.9	66	0.1	0	57	northeast wind	962	3.1	0	90
2021-12-06 20:00:00	-3.7	68	1.1	1	66	northeast wind	962	2.6	0	100
2021-12-06 21:00:00	-4.2	71	0.8	1	332	northwest wind	963	2.4	0	100
2021-12-06 22:00:00	-5.2	75	0.6	1	321	northwest wind	962	2	0	90
2021-12-06 23:00:00	-5.5	77	0.3	1	312	northwest wind	962	2	0	90
2021-12-07 00:00:00	-5.3	77	0.6	1	304	northwest wind	962	2.2	0	100
2021-12-07 01:00:00	-5	77	2.6	2	54	northeast wind	962	2.2	0	100
2021-12-07 02:00:00	-4.3	63	2.6	2	144	southeast wind	962	3	0	90
2021-12-07 03:00:00	-4.5	67	1	1	298	northwest wind	962	3.4	0	100
2021-12-07 04:00:00	-4.2	69	2.9	2	304	northwest wind	962	3	0	100
2021-12-07 05:00:00	-4.1	73	2.2	2	312	northwest wind	962	2.8	0	100
2021-12-07 06:00:00	-4.7	76	3	2	335	northwest wind	962	2.5	0	26
2021-12-07 07:00:00	-5.2	78	1	1	323	northwest wind	961	2.5	0	0
2021-12-07 08:00:00	-5.7	78	2.7	2	51	northeast wind	962	2.5	0	0
2021-12-07 09:00:00	-7.4	82	0.9	1	306	northwest wind	962	3	0	0

2021-12-07 10:00:00	-7.8	84	2.2	2	309	northwest wind	963	2.8	0	0
2021-12-07 11:00:00	-6.6	81	1.2	1	326	northwest wind	963	2.8	0	0
2021-12-07 12:00:00	-3.4	71	2.8	2	307	northwest wind	963	2.7	0	0
2021-12-07 13:00:00	-0.8	62	2.5	2	334	northwest wind	963	3.1	0	0
2021-12-07 14:00:00	2.2	50	2.5	2	139	southeast wind	962	4.6	0	0
2021-12-07 15:00:00	3.7	45	3	2	113	southeast wind	961	6.1	0	0
2021-12-07 16:00:00	3	47	2	2	137	southeast wind	961	5.9	0	0
2021-12-07 17:00:00	2.7	50	0.5	1	131	southeast wind	962	6.4	0	1
2021-12-07 18:00:00	1.4	54	0.1	0	113	southeast wind	963	5.9	0	70
2021-12-07 19:00:00	0	57	2.4	2	119	southeast wind	963	4.2	0	100
2021-12-07 20:00:00	-1.7	64	1.3	1	25	northeast wind	964	3.6	0	90
2021-12-07 21:00:00	-2.1	66	1.1	1	305	northwest wind	964	3.3	0	100
2021-12-07 22:00:00	-2.5	69	0.5	1	205	southwest wind	964	3.1	0	100
2021-12-07 23:00:00	-2.6	70	2.5	2	309	northwest wind	964	2.9	0	100
2021-12-08 00:00:00	-3.2	73	2.8	2	301	northwest wind	965	2.9	0	75
2021-12-08 01:00:00	-3.8	76	1.5	1	27	northeast wind	965	2.8	0	32
2021-12-08 02:00:00	-3.8	76	2.7	2	328	northwest wind	965	3	0	90
2021-12-08 03:00:00	-3.7	74	3	2	319	northwest wind	965	3	0	90
2021-12-08 04:00:00	-3.7	76	0.4	1	305	northwest wind	965	3	0	100
2021-12-08 05:00:00	-4.1	77	2.9	2	299	northwest wind	965	3.1	0	90
2021-12-08 06:00:00	-4.4	78	0.6	1	28	northeast wind	965	2.9	0	83
2021-12-08 07:00:00	-5.3	81	2.7	2	67	northeast wind	965	2.7	0	100
2021-12-08 08:00:00	-5.5	81	1.3	1	300	northwest wind	965	2.5	0	100
2021-12-08 09:00:00	-5.5	81	3	2	31	northeast wind	965	2.3	0	100
2021-12-08 10:00:00	-5.4	79	0.2	0	43	northeast wind	966	2.2	0	100
2021-12-08 11:00:00	-5.6	80	0	0	324	northwest wind	966	1.9	0	100
2021-12-08 12:00:00	-5.2	79	3	2	331	northwest wind	966	1.7	0	69
2021-12-08 13:00:00	-2.9	69	0.4	1	115	southeast wind	965	2	0	39
2021-12-08 14:00:00	-0.9	57	2.5	2	123	southeast wind	964	2.1	0	9
2021-12-08 15:00:00	-0.6	56	2.9	2	150	southeast wind	964	2.9	0	21
2021-12-08 16:00:00	-0.1	57	1.9	2	125	southeast wind	963	2.9	0	7
2021-12-08 17:00:00	-0.3	58	1.8	2	118	southeast wind	963	2.8	0	27
2021-12-08 18:00:00	-1.3	61	2.7	2	215	southwest wind	964	2.7	0	25
2021-12-08 19:00:00	-2.1	66	2.6	2	143	southeast wind	964	2.4	0	67
2021-12-08 20:00:00	-3.1	70	1.9	2	150	southeast wind	964	2.2	0	63
2021-12-08 21:00:00	-3.4	73	2	2	56	northeast wind	964	1.9	0	2
2021-12-08 22:00:00	-3	71	0	0	312	northwest wind	965	1.9	0	0
2021-12-08 23:00:00	-2.9	72	3.1	2	321	northwest wind	964	1.8	0	2

2021-12-09 00:00:00	-2.3	69	2.4	2	298	northwest wind	964	1.9	0	3
2021-12-09 01:00:00	-2	68	3.2	2	327	northwest wind	964	2.1	0	10
2021-12-09 02:00:00	-2.1	69	0.7	1	276	west wind	965	2.1	0	11
2021-12-09 03:00:00	-1.5	68	2.3	2	295	northwest wind	965	2.2	0	29
2021-12-09 04:00:00	-1.6	69	3.1	2	37	northeast wind	965	2.2	0	0
2021-12-09 05:00:00	-1.7	71	2.7	2	235	southwest wind	965	2.1	0	54
2021-12-09 06:00:00	-1.3	70	1	1	297	northwest wind	966	2.2	0	100
2021-12-09 07:00:00	-1	74	2	2	224	southwest wind	966	2.3	0	46
2021-12-09 08:00:00	-0.9	74	0.6	1	227	southwest wind	967	2.3	0	27
2021-12-09 09:00:00	-1.2	73	5.2	3	247	southwest wind	968	6.1	0	10
2021-12-09 10:00:00	-1.8	71	1.5	1	115	southeast wind	969	20.7	0	60
2021-12-09 11:00:00	-1.8	66	1.2	1	128	southeast wind	970	14.8	0	46
2021-12-09 12:00:00	-2.3	61	2.6	2	125	southeast wind	971	22.7	0	26
2021-12-09 13:00:00	-2	58	2.5	2	131	southeast wind	972	22.7	0	20
2021-12-09 14:00:00	-0.3	52	1	1	237	southwest wind	972	20.6	0	0
2021-12-09 15:00:00	0.7	49	1.5	1	128	southeast wind	972	23.2	0	0
2021-12-09 16:00:00	1.4	50	1.1	1	155	southeast wind	972	18.3	0	0
2021-12-09 17:00:00	0.1	52	3.2	2	125	southeast wind	973	17.7	0	16
2021-12-09 18:00:00	-0.8	63	2.3	2	140	southeast wind	974	13.8	0	6
2021-12-09 19:00:00	-1	66	1.9	2	215	southwest wind	975	12.9	0	0
2021-12-09 20:00:00	-1	59	3	2	138	southeast wind	976	12.9	0	16
2021-12-09 21:00:00	-2.3	46	2.9	2	155	southeast wind	977	30	0	9
2021-12-09 22:00:00	-3.3	49	1.2	1	129	southeast wind	978	30	0	0
2021-12-09 23:00:00	-3.5	47	0	0	147	southeast wind	978	30	0	0
2021-12-10 00:00:00	-5.2	55	0.1	0	143	southeast wind	978	30	0	0
2021-12-10 01:00:00	-6.7	62	0.9	1	295	northwest wind	978	30	0	0
2021-12-10 02:00:00	-8	68	1.6	2	306	northwest wind	979	17.2	0	0
2021-12-10 03:00:00	-9.2	72	0.5	1	64	northeast wind	979	30	0	0
2021-12-10 04:00:00	-10.1	75	1.9	2	93	east wind	979	30	0	0
2021-12-10 05:00:00	-9	74	1.5	1	125	southeast wind	979	30	0	0
2021-12-10 06:00:00	-10.1	79	3.1	2	128	southeast wind	980	30	0	2
2021-12-10 07:00:00	-11.1	83	1.6	2	293	northwest wind	980	27.6	0	6
2021-12-10 08:00:00	-10.5	82	2	2	41	northeast wind	981	25	0	58
2021-12-10 09:00:00	-10.4	75	2.7	2	25	northeast wind	981	25	0	62
2021-12-10 10:00:00	-10.7	76	0.2	0	126	southeast wind	982	29	0	60
2021-12-10 11:00:00	-10.4	75	3.1	2	121	southeast wind	982	26.1	0	52
2021-12-10 12:00:00	-9.7	74	3	2	115	southeast wind	982	7.9	0	56
2021-12-10 13:00:00	-9.1	74	0.9	1	126	southeast wind	981	11.8	0	69

2021-12-10 14:00:00	-9.1	71	2.8	2	137	southeast wind	981	20.9	0	34
2021-12-10 15:00:00	-9.4	72	1.5	1	37	northeast wind	980	29.7	0	50
2021-12-10 16:00:00	-9.1	71	1.3	1	148	southeast wind	980	26.1	0	51
2021-12-10 17:00:00	-9	70	1.3	1	153	southeast wind	980	22.3	0	54
2021-12-10 18:00:00	-9	73	2	2	58	northeast wind	980	18.9	0	15
2021-12-10 19:00:00	-9.3	76	1.1	1	27	northeast wind	980	7	0	6
2021-12-10 20:00:00	-9.8	80	4	3	126	southeast wind	981	12.7	0	66
2021-12-10 21:00:00	-10.4	80	0	0	107	east wind	981	14.7	0	68
2021-12-10 22:00:00	-10.4	80	2.4	2	113	southeast wind	981	6.4	0	70
2021-12-10 23:00:00	-10.8	82	1	1	115	southeast wind	981	6.4	0	100
2021-12-11 00:00:00	-11.2	82	1.1	1	129	southeast wind	981	5.1	0	100
2021-12-11 01:00:00	-11.4	84	1.2	1	37	northeast wind	981	6.6	0	90
2021-12-11 02:00:00	-11.5	83	1.4	1	57	northeast wind	980	13	0	100
2021-12-11 03:00:00	-11.5	83	0.7	1	65	northeast wind	979	5.8	0	100
2021-12-11 04:00:00	-11.7	82	0.4	1	148	southeast wind	979	5.7	0	90
2021-12-11 05:00:00	-12	85	0.2	0	122	southeast wind	978	8.6	0	90
2021-12-11 06:00:00	-12.2	85	0.1	0	137	southeast wind	978	5.2	0	90
2021-12-11 07:00:00	-12.3	85	2.5	2	58	northeast wind	977	3.2	0	100
2021-12-11 08:00:00	-12.3	84	2.1	2	26	northeast wind	978	2.4	0	100
2021-12-11 09:00:00	-12.2	84	2.9	2	44	northeast wind	978	6.4	0	100
2021-12-11 10:00:00	-12.4	84	1.4	1	60	northeast wind	978	8.1	0	100
2021-12-11 11:00:00	-12.5	83	3.1	2	337	northwest wind	978	2.8	0	0
2021-12-11 12:00:00	-11.6	77	2.8	2	154	southeast wind	978	5.1	0	0
2021-12-11 13:00:00	-11.1	74	3.1	2	307	northwest wind	977	3.7	0	0
2021-12-11 14:00:00	-10.5	72	1.1	1	60	northeast wind	976	5.3	0	0
2021-12-11 15:00:00	-9.2	69	1.4	1	242	southwest wind	975	14	0	0
2021-12-11 16:00:00	-8.5	61	1.2	1	45	northeast wind	974	17	0	0
2021-12-11 17:00:00	-8.9	68	1.3	1	119	southeast wind	974	15.5	0	0
2021-12-11 18:00:00	-9.8	70	0.1	0	213	southwest wind	974	17.7	0	0
2021-12-11 19:00:00	-11	78	2.7	2	143	southeast wind	974	17.3	0	0
2021-12-11 20:00:00	-12	81	1.8	2	33	northeast wind	975	13.8	0	0
2021-12-11 21:00:00	-12.6	83	0	0	48	northeast wind	975	7.1	0	26
2021-12-11 22:00:00	-12.5	86	1.5	1	296	northwest wind	974	3.7	0	90
2021-12-11 23:00:00	-12.2	84	0.2	0	58	northeast wind	973	4.3	0	100
2021-12-12 00:00:00	-12.5	84	1.6	2	16	north wind	973	2.8	0	100
2021-12-12 01:00:00	-12.6	82	0.4	1	241	southwest wind	972	5.9	0	75
2021-12-12 02:00:00	-14.1	83	3	2	214	southwest wind	971	6.6	0	79
2021-12-12 03:00:00	-16	84	2.3	2	204	southwest wind	971	6.5	0	18

2021-12-12 04:00:00	-16.7	85	1.4	1	244	southwest wind	970	6.8	0	19
2021-12-12 05:00:00	-17.7	81	0	0	311	northwest wind	970	8.1	0	49
2021-12-12 06:00:00	-17.9	82	1.7	2	314	northwest wind	970	7.2	0	49
2021-12-12 07:00:00	-18.5	81	0.8	1	329	northwest wind	970	7.4	0	68
2021-12-12 08:00:00	-18.4	82	2.9	2	319	northwest wind	970	6.5	0	70
2021-12-12 09:00:00	-18	81	2.1	2	319	northwest wind	970	6	0	76
2021-12-12 10:00:00	-18.1	80	1.3	1	312	northwest wind	969	7.3	0	100
2021-12-12 11:00:00	-16.6	83	0.7	1	329	northwest wind	969	5	0	2
2021-12-12 12:00:00	-13.1	79	0.8	1	62	northeast wind	969	8	0	0
2021-12-12 13:00:00	-11.9	77	1.9	2	53	northeast wind	969	7.1	0	0
2021-12-12 14:00:00	-10.2	71	0.8	1	48	northeast wind	968	8.6	0	0
2021-12-12 15:00:00	-9.5	69	0.5	1	42	northeast wind	967	10.4	0	60
2021-12-12 16:00:00	-9.5	71	1.7	2	41	northeast wind	967	8.6	0	36
2021-12-12 17:00:00	-9.4	66	3.2	2	45	northeast wind	967	10.9	0	9
2021-12-12 18:00:00	-10	71	1.4	1	23	northeast wind	967	11.1	0	67
2021-12-12 19:00:00	-10.4	75	0.7	1	41	northeast wind	967	9.4	0	90
2021-12-12 20:00:00	-11.2	78	1.3	1	39	northeast wind	967	7.4	0	83
2021-12-12 21:00:00	-11.5	81	2.4	2	296	northwest wind	967	6.6	0	90
2021-12-12 22:00:00	-11.1	79	0.9	1	209	southwest wind	967	5.8	0	100
2021-12-12 23:00:00	-10.7	77	3.2	2	218	southwest wind	967	7.3	0	100
2021-12-13 00:00:00	-10.7	79	1.9	2	33	northeast wind	967	6.2	0	16
2021-12-13 01:00:00	-11.3	80	2.4	2	17	north wind	966	6.2	0	26
2021-12-13 02:00:00	-11.5	83	0.6	1	23	northeast wind	966	5.6	0	25
2021-12-13 03:00:00	-11.8	84	0.3	1	67	northeast wind	966	5.8	0	61
2021-12-13 04:00:00	-12.7	87	3.2	2	26	northeast wind	965	5.8	0	70
2021-12-13 05:00:00	-12.9	88	1.2	1	24	northeast wind	965	4.6	0	100
2021-12-13 06:00:00	-13.1	88	1.4	1	308	northwest wind	965	4.6	0	90
2021-12-13 07:00:00	-13	87	1.4	1	157	southeast wind	965	4.6	0	90
2021-12-13 08:00:00	-13.5	86	0.9	1	36	northeast wind	965	4.8	0	73
2021-12-13 09:00:00	-13.7	85	2.1	2	46	northeast wind	966	5.2	0	74
2021-12-13 10:00:00	-14	86	0.7	1	27	northeast wind	966	2.6	0	69
2021-12-13 11:00:00	-13.4	86	1.8	2	220	southwest wind	966	1.7	0	28
2021-12-13 12:00:00	-12.3	82	0.9	1	117	southeast wind	966	3.1	0	70
2021-12-13 13:00:00	-10.8	76	0.6	1	126	southeast wind	965	3.9	0	16
2021-12-13 14:00:00	-10	72	1.8	2	125	southeast wind	964	4.5	0	42
2021-12-13 15:00:00	-9.5	72	0.5	1	123	southeast wind	964	4.5	0	1
2021-12-13 16:00:00	-9.4	70	0.8	1	116	southeast wind	963	4.5	0	2
2021-12-13 17:00:00	-8.9	68	1.7	2	116	southeast wind	963	5	0	0

2021-12-13 18:00:00	-9.4	70	3.2	2	129	southeast wind	963	5.2	0	0
2021-12-13 19:00:00	-10.6	75	2.8	2	140	southeast wind	964	4.7	0	0
2021-12-13 20:00:00	-11.7	79	3	2	335	northwest wind	964	4.3	0	2
2021-12-13 21:00:00	-12.4	81	2.7	2	54	northeast wind	964	3	0	0
2021-12-13 22:00:00	-13.3	81	2.1	2	329	northwest wind	964	3.2	0	0
2021-12-13 23:00:00	-13.2	84	0.4	1	300	northwest wind	964	2.8	0	0
2021-12-14 00:00:00	-13	82	2.1	2	300	northwest wind	964	3.2	0	2
2021-12-14 01:00:00	-12.2	82	0.3	1	325	northwest wind	964	4.2	0	9
2021-12-14 02:00:00	-12.8	82	0.9	1	318	northwest wind	964	4.5	0	2
2021-12-14 03:00:00	-12.8	82	2.7	2	298	northwest wind	964	4.7	0	0
2021-12-14 04:00:00	-12.7	81	1.8	2	219	southwest wind	964	4.7	0	0
2021-12-14 05:00:00	-13.6	83	2.6	2	240	southwest wind	964	9.8	0	0
2021-12-14 06:00:00	-13.3	85	0.9	1	328	northwest wind	963	12.8	0	0
2021-12-14 07:00:00	-12.4	84	1.4	1	325	northwest wind	963	15.5	0	0
2021-12-14 08:00:00	-13.3	83	2.1	2	311	northwest wind	964	15.5	0	0
2021-12-14 09:00:00	-14	85	1.7	2	311	northwest wind	964	8.8	0	2
2021-12-14 10:00:00	-13.8	86	1	1	294	northwest wind	964	4.5	0	14
2021-12-14 11:00:00	-10.7	85	0.8	1	316	northwest wind	965	11.7	0	0
2021-12-14 12:00:00	-7.5	77	0.7	1	146	southeast wind	965	10	0	0
2021-12-14 13:00:00	-5.9	72	1.6	2	38	northeast wind	965	12.8	0	0
2021-12-14 14:00:00	-4.8	66	1.8	2	141	southeast wind	965	12.8	0	0
2021-12-14 15:00:00	-3.3	62	1	1	117	southeast wind	964	9.2	0	0
2021-12-14 16:00:00	-3	61	0.1	0	123	southeast wind	964	8.9	0	0
2021-12-14 17:00:00	-2.7	59	2.6	2	124	southeast wind	965	9.8	0	0
2021-12-14 18:00:00	-4.3	65	2.3	2	153	southeast wind	965	8.9	0	0
2021-12-14 19:00:00	-5.7	70	1	1	59	northeast wind	966	7.4	0	0
2021-12-14 20:00:00	-7.5	78	1.6	2	56	northeast wind	967	5.8	0	15
2021-12-14 21:00:00	-9.2	81	0.1	0	336	northwest wind	967	4.4	0	50
2021-12-14 22:00:00	-9.5	82	3.1	2	295	northwest wind	968	3.6	0	65
2021-12-14 23:00:00	-10.7	83	2.7	2	296	northwest wind	968	3.5	0	39
2021-12-15 00:00:00	-11	84	3	2	326	northwest wind	968	3.4	0	55
2021-12-15 01:00:00	-11.4	85	1	1	295	northwest wind	968	4	0	31
2021-12-15 02:00:00	-11.8	86	2.9	2	235	southwest wind	968	4	0	71
2021-12-15 03:00:00	-11.7	86	0.6	1	30	northeast wind	968	6	0	100
2021-12-15 04:00:00	-11.1	87	1.2	1	130	southeast wind	969	6	0	100
2021-12-15 05:00:00	-11.5	87	3.2	2	37	northeast wind	969	3	0	100
2021-12-15 06:00:00	-11.6	88	0.5	1	36	northeast wind	969	3	0	100
2021-12-15 07:00:00	-11.6	88	1.7	2	208	southwest wind	970	3	0	100

2021-12-15 08:00:00	-11.4	88	1.7	2	30	northeast wind	971	3	0	100
2021-12-15 09:00:00	-11	88	2.3	2	317	northwest wind	971	2.9	0	100
2021-12-15 10:00:00	-10.5	87	1.7	2	328	northwest wind	971	3.3	0	100
2021-12-15 11:00:00	-10.1	87	1.8	2	333	northwest wind	972	2.5	0	34
2021-12-15 12:00:00	-9.2	86	2.3	2	296	northwest wind	972	2.4	0	70
2021-12-15 13:00:00	-8.3	84	3.1	2	61	northeast wind	972	2.9	0	58
2021-12-15 14:00:00	-7.5	81	3.1	2	120	southeast wind	972	3.3	0	40
2021-12-15 15:00:00	-7.4	80	0.4	1	150	southeast wind	972	3.7	0	50
2021-12-15 16:00:00	-6.9	79	2.9	2	232	southwest wind	972	3.7	0	66
2021-12-15 17:00:00	-6.3	77	2.2	2	116	southeast wind	972	4.7	0	70
2021-12-15 18:00:00	-6.8	78	1.5	1	48	northeast wind	972	5.2	0	90
2021-12-15 19:00:00	-7.6	84	1.2	1	127	southeast wind	974	4	0	76
2021-12-15 20:00:00	-7.9	86	1.7	2	121	southeast wind	974	3.3	0	46
2021-12-15 21:00:00	-8.2	85	3.1	2	141	southeast wind	975	3.3	0	42
2021-12-15 22:00:00	-8.4	86	1.5	1	132	southeast wind	975	3.2	0	100
2021-12-15 23:00:00	-8.5	86	0.7	1	137	southeast wind	975	3	0	90
2021-12-16 00:00:00	-8.5	86	1.2	1	323	northwest wind	975	2.9	0	90
2021-12-16 01:00:00	-8.6	85	1.6	2	308	northwest wind	975	3	0	100
2021-12-16 02:00:00	-8.6	85	0	0	53	northeast wind	975	3	0	90
2021-12-16 03:00:00	-8.4	86	0.6	1	305	northwest wind	976	2.9	0	90
2021-12-16 04:00:00	-8.3	87	2.4	2	155	southeast wind	976	2.8	0	90
2021-12-16 05:00:00	-8.2	87	0.7	1	191	south wind	976	2.8	0	90
2021-12-16 06:00:00	-8.3	88	3.2	2	325	northwest wind	976	2.7	0	100
2021-12-16 07:00:00	-8.4	88	2.2	2	63	northeast wind	976	2.6	0	90
2021-12-16 08:00:00	-8.2	86	2	2	42	northeast wind	976	3.1	0	90
2021-12-16 09:00:00	-8.4	86	0.4	1	42	northeast wind	977	2.8	0	100
2021-12-16 10:00:00	-8.3	86	0.2	0	122	southeast wind	977	2.7	0	100
2021-12-16 11:00:00	-8.1	86	0.8	1	121	southeast wind	978	2.9	0	68
2021-12-16 12:00:00	-7.9	84	3.2	2	31	northeast wind	978	2.9	0	14
2021-12-16 13:00:00	-8	84	2.1	2	67	northeast wind	978	1.6	0	44
2021-12-16 14:00:00	-7.4	81	3.1	2	134	southeast wind	977	1.9	0	2
2021-12-16 15:00:00	-7.5	80	1.7	2	146	southeast wind	977	3.4	0	45
2021-12-16 16:00:00	-7.4	79	3.1	2	148	southeast wind	977	2.2	0	0
2021-12-16 17:00:00	-7.3	81	0.4	1	124	southeast wind	976	2.2	0	12
2021-12-16 18:00:00	-7.4	81	0.2	0	48	northeast wind	976	2.8	0	4
2021-12-16 19:00:00	-7.8	84	2.6	2	119	southeast wind	977	2.7	0	22
2021-12-16 20:00:00	-8.1	84	2.6	2	334	northwest wind	977	4.7	0	90
2021-12-16 21:00:00	-8.1	84	2.7	2	115	southeast wind	977	4	0	27

2021-12-16 22:00:00	-8.1	85	1.5	1	32	northeast wind	977	3.9	0	64
2021-12-16 23:00:00	-8.1	83	0.4	1	137	southeast wind	977	3.9	0	63
2021-12-17 00:00:00	-8.3	84	0.8	1	117	southeast wind	976	3.5	0	70
2021-12-17 01:00:00	-8.9	85	2.5	2	116	southeast wind	976	3.5	0	83
2021-12-17 02:00:00	-9.2	84	0.3	1	141	southeast wind	976	9.5	0	63
2021-12-17 03:00:00	-9.2	84	2.2	2	293	northwest wind	976	9.6	0	58
2021-12-17 04:00:00	-9.1	86	1.1	1	123	southeast wind	975	3.4	0	67
2021-12-17 05:00:00	-9.1	87	2.6	2	53	northeast wind	975	3.7	0	62
2021-12-17 06:00:00	-9.2	88	0.9	1	30	northeast wind	974	3.4	0	57
2021-12-17 07:00:00	-9.2	89	2.3	2	118	southeast wind	974	2.9	0	59
2021-12-17 08:00:00	-9.3	90	0.6	1	226	southwest wind	974	2.9	0	60
2021-12-17 09:00:00	-9.3	90	2.2	2	140	southeast wind	975	2.9	0	64
2021-12-17 10:00:00	-9.4	90	0.1	0	132	southeast wind	975	2.6	0	62
2021-12-17 11:00:00	-9.2	89	0.8	1	155	southeast wind	975	2.2	0	68
2021-12-17 12:00:00	-8.8	86	1.9	2	128	southeast wind	975	1.2	0	74
2021-12-17 13:00:00	-8.7	81	3	2	128	southeast wind	974	2.1	0.3	0
2021-12-17 14:00:00	-8.7	81	0.9	1	155	southeast wind	974	4.8	0	10
2021-12-17 15:00:00	-8.8	81	3.1	2	129	southeast wind	973	3.2	0	43
2021-12-17 16:00:00	-8.3	79	1.5	1	144	southeast wind	973	3	0	55
2021-12-17 17:00:00	-8.1	76	1.2	1	133	southeast wind	972	6.3	0	53
2021-12-17 18:00:00	-8.5	78	0.7	1	153	southeast wind	972	2.6	0	12
2021-12-17 19:00:00	-9.2	80	1.8	2	139	southeast wind	973	5.9	0	90
2021-12-17 20:00:00	-9.1	83	2.2	2	126	southeast wind	973	5.8	0	90
2021-12-17 21:00:00	-10.1	85	3.1	2	237	southwest wind	973	4.7	0	90
2021-12-17 22:00:00	-9.3	82	0.2	0	153	southeast wind	973	4.8	0	69
2021-12-17 23:00:00	-9.4	82	1.2	1	149	southeast wind	973	4.7	0	83
2021-12-18 00:00:00	-9.5	82	2.9	2	37	northeast wind	972	2.8	0	100
2021-12-18 01:00:00	-9.7	82	1.8	2	114	southeast wind	972	3.2	0	68
2021-12-18 02:00:00	-9.8	84	3.2	2	141	southeast wind	971	4	0	71
2021-12-18 03:00:00	-10.5	88	3	2	156	southeast wind	971	4.4	0	83
2021-12-18 04:00:00	-10.5	88	2.8	2	226	southwest wind	970	4.4	0	83
2021-12-18 05:00:00	-10.3	88	0.7	1	86	east wind	970	3.5	0	83
2021-12-18 06:00:00	-10.4	86	1.5	1	218	southwest wind	969	4.1	0	83
2021-12-18 07:00:00	-10.3	85	2	2	145	southeast wind	969	2.5	0	83
2021-12-18 08:00:00	-10.5	84	2.8	2	39	northeast wind	969	3.3	0	90
2021-12-18 09:00:00	-10.6	83	2.9	2	23	northeast wind	968	3.6	0	83
2021-12-18 10:00:00	-10.6	83	2.7	2	155	southeast wind	968	3.2	0	90
2021-12-18 11:00:00	-10.5	81	2.5	2	29	northeast wind	968	3	0	90

2021-12-18 12:00:00	-10.1	79	1.3	1	44	northeast wind	968	3	0	5
2021-12-18 13:00:00	-9.4	76	0.6	1	113	southeast wind	967	1.8	0	1
2021-12-18 14:00:00	-9.4	77	1.2	1	219	southwest wind	967	1.7	0.3	2
2021-12-18 15:00:00	-9.3	78	0.2	0	213	southwest wind	966	1.3	0	15
2021-12-18 16:00:00	-8.8	73	1.1	1	211	southwest wind	966	1.3	0.3	10
2021-12-18 17:00:00	-8.8	75	3	2	262	west wind	964	1.9	0	20
2021-12-18 18:00:00	-9.7	77	2.9	2	29	northeast wind	964	3.2	0	0
2021-12-18 19:00:00	-11	82	1.8	2	56	northeast wind	964	5.3	0	0
2021-12-18 20:00:00	-12.1	84	0	0	295	northwest wind	964	5.3	0	90
2021-12-18 21:00:00	-13.4	85	1.3	1	314	northwest wind	964	3.4	0	90
2021-12-18 22:00:00	-14.6	85	3.2	2	311	northwest wind	964	3.8	0	64
2021-12-18 23:00:00	-14.8	86	3	2	303	northwest wind	964	3.2	0	35
2021-12-19 00:00:00	-14	89	1.7	2	208	southwest wind	964	3.2	0	62
2021-12-19 01:00:00	-15.6	84	0	0	337	northwest wind	963	3.7	0	62
2021-12-19 02:00:00	-16.7	83	2	2	294	northwest wind	963	4.3	0	0
2021-12-19 03:00:00	-17.2	83	0.7	1	294	northwest wind	962	4.7	0	11
2021-12-19 04:00:00	-17.6	82	1.7	2	335	northwest wind	962	4.8	0	65
2021-12-19 05:00:00	-18.1	83	2.4	2	335	northwest wind	960	4.8	0	26
2021-12-19 06:00:00	-18.1	83	2.4	2	335	northwest wind	960	4.4	0	28
2021-12-19 07:00:00	-18.1	83	2.4	2	335	northwest wind	960	4.9	0	57
2021-12-19 08:00:00	-17.6	83	2.4	2	233	southwest wind	960	4.9	0	68
2021-12-19 09:00:00	-15	87	2.9	2	217	southwest wind	960	3.8	0	73
2021-12-19 10:00:00	-13.9	88	0	0	329	northwest wind	961	1.3	0	90
2021-12-19 11:00:00	-13.6	88	0	0	310	northwest wind	961	1	0	90
2021-12-19 12:00:00	-12.8	87	0.2	0	213	southwest wind	961	2	0	100
2021-12-19 13:00:00	-11.5	87	1.2	1	221	southwest wind	961	2.6	0	73
2021-12-19 14:00:00	-9.4	85	0.3	1	191	south wind	960	3.2	0	60
2021-12-19 15:00:00	-8.2	74	2	2	209	southwest wind	959	4.1	0	53
2021-12-19 16:00:00	-9.4	76	0.8	1	145	southeast wind	959	4.6	0	62
2021-12-19 17:00:00	-9.4	78	1	1	125	southeast wind	959	4.5	0	59
2021-12-19 18:00:00	-10.1	79	0.7	1	150	southeast wind	959	4.5	0	74
2021-12-19 19:00:00	-11.1	82	2.7	2	124	southeast wind	959	4.1	0	90
2021-12-19 20:00:00	-11.7	87	0.5	1	32	northeast wind	959	2.9	0	70
2021-12-19 21:00:00	-11.2	88	1	1	32	northeast wind	959	2.9	0	64
2021-12-19 22:00:00	-11.5	88	0.6	1	296	northwest wind	959	1	0	73
2021-12-19 23:00:00	-11.7	89	3.1	2	337	northwest wind	959	1.9	0.2	100
2021-12-20 00:00:00	-11.7	89	2.2	2	214	southwest wind	959	1.9	0	100
2021-12-20 01:00:00	-11.8	89	1.7	2	227	southwest wind	958	2.3	0	90

2021-12-20 02:00:00	-11.9	90	2.9	2	221	southwest wind	958	1.8	0	100
2021-12-20 03:00:00	-11.9	88	3.2	2	278	west wind	958	2.1	0	100
2021-12-20 04:00:00	-11.9	88	1.7	2	245	southwest wind	957	2.1	0	100
2021-12-20 05:00:00	-11.8	88	0.3	1	203	southwest wind	957	2.2	0	100
2021-12-20 06:00:00	-11.7	87	3	2	121	southeast wind	957	2.6	0	100
2021-12-20 07:00:00	-11.5	87	0.5	1	25	northeast wind	957	2.9	0	100
2021-12-20 08:00:00	-11.5	88	0.8	1	324	northwest wind	958	2.9	0	100
2021-12-20 09:00:00	-11.3	87	0.1	0	287	west wind	958	2.6	0	100
2021-12-20 10:00:00	-11.4	86	0.8	1	231	southwest wind	958	2.8	0	100
2021-12-20 11:00:00	-11.4	86	1.4	1	228	southwest wind	959	2.3	0	76
2021-12-20 12:00:00	-9.4	84	2.9	2	11	north wind	959	2.3	0	53
2021-12-20 13:00:00	-7.7	74	1.5	1	137	southeast wind	960	3.2	0	47
2021-12-20 14:00:00	-7.4	72	1.5	1	127	southeast wind	959	3.5	0	66
2021-12-20 15:00:00	-8.4	76	1.9	2	149	southeast wind	959	3.5	0	44
2021-12-20 16:00:00	-7.2	71	0.1	0	246	southwest wind	960	3.4	0	54
2021-12-20 17:00:00	-7.1	71	0.4	1	133	southeast wind	960	3.6	0	63
2021-12-20 18:00:00	-7.4	73	2.3	2	36	northeast wind	960	3.1	0	55
2021-12-20 19:00:00	-8.4	78	1.7	2	157	southeast wind	961	3.3	0	100
2021-12-20 20:00:00	-8.8	81	0.4	1	124	southeast wind	962	3	0	100
2021-12-20 21:00:00	-9.7	82	2.5	2	124	southeast wind	963	3.3	0	100
2021-12-20 22:00:00	-10.7	84	3.2	2	226	southwest wind	963	2.7	0	90
2021-12-20 23:00:00	-11.3	87	2.5	2	62	northeast wind	963	2.3	0	55
2021-12-21 00:00:00	-11.9	86	0.3	1	327	northwest wind	964	2.3	0	74
2021-12-21 01:00:00	-12.7	87	0	0	214	southwest wind	964	2.3	0	83
2021-12-21 02:00:00	-13	88	2.6	2	70	east wind	963	2.3	0	50
2021-12-21 03:00:00	-13.5	86	0.2	0	49	northeast wind	964	2.2	0	0
2021-12-21 04:00:00	-13.1	88	1.1	1	65	northeast wind	964	2.3	0	0
2021-12-21 05:00:00	-13.8	86	2.4	2	301	northwest wind	964	2.3	0	0
2021-12-21 06:00:00	-14.1	86	1.3	1	321	northwest wind	964	2.5	0	0
2021-12-21 07:00:00	-14.8	86	2.5	2	309	northwest wind	964	2.6	0	4
2021-12-21 08:00:00	-15	84	0	0	223	southwest wind	964	2.6	0	100
2021-12-21 09:00:00	-15.4	84	1.1	1	342	north wind	964	2.6	0	90
2021-12-21 10:00:00	-15	85	0.2	0	0	north wind	964	1.9	0	63
2021-12-21 11:00:00	-13.7	86	0.9	1	32	northeast wind	965	2.4	0	6
2021-12-21 12:00:00	-12.2	86	2	2	233	southwest wind	965	1.7	0	30
2021-12-21 13:00:00	-10.7	85	1.5	1	273	west wind	965	1.3	0	33
2021-12-21 14:00:00	-8.5	76	0.6	1	210	southwest wind	964	1.9	0	4
2021-12-21 15:00:00	-7.3	69	1.5	1	117	southeast wind	963	2.3	0	4

2021-12-21 16:00:00	-7.5	73	2.3	2	113	southeast wind	964	2.7	0	3
2021-12-21 17:00:00	-7.7	71	0.1	0	115	southeast wind	964	3.1	0	6
2021-12-21 18:00:00	-8.4	74	2.7	2	142	southeast wind	964	3.3	0	0
2021-12-21 19:00:00	-8.3	73	0.8	1	165	south wind	964	3.4	0	0
2021-12-21 20:00:00	-8.1	74	0.8	1	337	northwest wind	965	3.2	0	0
2021-12-21 21:00:00	-8	75	1.7	2	118	southeast wind	965	3.1	0	0
2021-12-21 22:00:00	-7.6	77	0.2	0	304	northwest wind	965	3	0	72
2021-12-21 23:00:00	-7	79	1.6	2	233	southwest wind	964	2.7	0	0
2021-12-22 00:00:00	-7.1	87	1.3	1	303	northwest wind	963	2.7	0	6
2021-12-22 01:00:00	-8.5	80	0.5	1	39	northeast wind	963	2.4	0	16
2021-12-22 02:00:00	-9.2	81	2.3	2	117	southeast wind	963	2.6	0	0
2021-12-22 03:00:00	-10.1	82	1.3	1	128	southeast wind	963	2.5	0	1
2021-12-22 04:00:00	-10	85	1.9	2	42	northeast wind	963	2.5	0	40
2021-12-22 05:00:00	-10.2	88	1.7	2	331	northwest wind	963	1.5	0	51
2021-12-22 06:00:00	-10.1	91	1.5	1	225	southwest wind	962	1.3	0	51
2021-12-22 07:00:00	-9.8	92	2.9	2	210	southwest wind	962	1	0	2
2021-12-22 08:00:00	-9.6	92	2.4	2	216	southwest wind	962	1	0	0
2021-12-22 09:00:00	-9.9	92	0	0	130	southeast wind	962	1.5	0	21
2021-12-22 10:00:00	-9.8	92	0.9	1	127	southeast wind	962	0.5	0	11
2021-12-22 11:00:00	-9.7	91	0.4	1	316	northwest wind	962	0.6	0	45
2021-12-22 12:00:00	-9.1	91	0.6	1	209	southwest wind	962	1.1	0	53
2021-12-22 13:00:00	-8.2	90	3.2	2	216	southwest wind	961	1.4	0	54
2021-12-22 14:00:00	-7.8	89	0	0	155	southeast wind	960	1.4	0	51
2021-12-22 15:00:00	-7.3	80	2.4	2	318	northwest wind	959	2.1	0	52
2021-12-22 16:00:00	-7	80	1	1	297	northwest wind	959	3.6	0	50
2021-12-22 17:00:00	-6.5	82	0	0	314	northwest wind	959	2.3	0	49
2021-12-22 18:00:00	-6.2	80	2.5	2	311	northwest wind	959	2.7	0	53
2021-12-22 19:00:00	-6	82	2.9	2	316	northwest wind	960	3.1	0	44
2021-12-22 20:00:00	-5.8	84	1.3	1	322	northwest wind	960	2.7	0	60
2021-12-22 21:00:00	-5.4	84	1.3	1	293	northwest wind	961	2.2	0	100
2021-12-22 22:00:00	-5.3	84	2.2	2	116	southeast wind	961	3.1	0	100
2021-12-22 23:00:00	-6.2	87	2.8	2	51	northeast wind	961	3	0	100
2021-12-23 00:00:00	-6.6	90	1.8	2	133	southeast wind	961	3	0	100
2021-12-23 01:00:00	-7.1	91	1.4	1	142	southeast wind	961	1.8	0	70
2021-12-23 02:00:00	-7.4	92	2	2	37	northeast wind	961	1.5	0	90
2021-12-23 03:00:00	-7.5	93	0.5	1	53	northeast wind	961	1.1	0	70
2021-12-23 04:00:00	-7.3	92	0.1	0	37	northeast wind	961	1.3	0	69
2021-12-23 05:00:00	-7.1	91	1.4	1	129	southeast wind	961	1.5	0	69

2021-12-23 06:00:00	-7.1	91	0.7	1	332	northwest wind	961	1.5	0	67
2021-12-23 07:00:00	-7.2	91	2.3	2	42	northeast wind	962	1.6	0	69
2021-12-23 08:00:00	-7.1	90	2.4	2	41	northeast wind	962	1.6	0	90
2021-12-23 09:00:00	-7.3	91	1.1	1	302	northwest wind	963	1.5	0	100
2021-12-23 10:00:00	-7	90	1	1	216	southwest wind	963	1.7	0	100
2021-12-23 11:00:00	-6.3	87	0.4	1	219	southwest wind	964	1.7	0	100
2021-12-23 12:00:00	-5.3	82	0.3	1	216	southwest wind	964	2.1	0	100
2021-12-23 13:00:00	-4.9	80	0.9	1	115	southeast wind	964	2.4	0	83
2021-12-23 14:00:00	-4.5	78	1.3	1	113	southeast wind	964	2.5	0	53
2021-12-23 15:00:00	-3.5	73	0.1	0	149	southeast wind	963	2.5	0	45
2021-12-23 16:00:00	-2.6	73	0.8	1	116	southeast wind	964	4.1	0	45
2021-12-23 17:00:00	-3.1	73	0	0	119	southeast wind	964	6.9	0	58
2021-12-23 18:00:00	-3.6	76	3.1	2	145	southeast wind	965	7.2	0	60
2021-12-23 19:00:00	-5.3	81	3.2	2	120	southeast wind	966	6.1	0	0
2021-12-23 20:00:00	-5.7	86	0.7	1	25	northeast wind	966	4.3	0	4
2021-12-23 21:00:00	-5.4	86	0.7	1	214	southwest wind	967	4.2	0	62
2021-12-23 22:00:00	-5.8	86	1	1	290	west wind	967	3.5	0	63
2021-12-23 23:00:00	-5.7	86	3	2	237	southwest wind	967	3.8	0	64
2021-12-24 00:00:00	-5.8	87	1.4	1	298	northwest wind	967	3.8	0	60
2021-12-24 01:00:00	-6.1	88	1.8	2	65	northeast wind	967	4.2	0	56
2021-12-24 02:00:00	-6.5	90	1.8	2	11	north wind	967	3.7	0	51
2021-12-24 03:00:00	-7	89	1.2	1	244	southwest wind	967	2.9	0	54
2021-12-24 04:00:00	-6.4	91	2.1	2	155	southeast wind	967	4.3	0	59
2021-12-24 05:00:00	-6.6	93	1.5	1	54	northeast wind	968	2.2	0	63
2021-12-24 06:00:00	-6.6	94	2.7	2	305	northwest wind	968	1.5	0	58
2021-12-24 07:00:00	-6.5	93	1.9	2	304	northwest wind	969	1.7	0	59
2021-12-24 08:00:00	-6.3	93	3.1	2	300	northwest wind	969	1.7	0	52
2021-12-24 09:00:00	-6.2	93	1.6	2	229	southwest wind	969	1.7	0	53
2021-12-24 10:00:00	-6.1	92	1.2	1	309	northwest wind	970	1.8	0	50
2021-12-24 11:00:00	-5.7	91	1.7	2	312	northwest wind	971	1.9	0	26
2021-12-24 12:00:00	-5.1	87	3.2	2	140	southeast wind	971	2	0	44
2021-12-24 13:00:00	-4.4	83	2.4	2	46	northeast wind	971	3.3	0	40
2021-12-24 14:00:00	-3.9	82	0.6	1	154	southeast wind	970	2.7	0	38
2021-12-24 15:00:00	-3.6	82	2.3	2	127	southeast wind	969	3	0	43
2021-12-24 16:00:00	-3.6	81	0.8	1	115	southeast wind	969	3.3	0	26
2021-12-24 17:00:00	-3.8	83	1.3	1	121	southeast wind	969	3.4	0	42
2021-12-24 18:00:00	-4.1	84	3.2	2	121	southeast wind	969	3.1	0	39
2021-12-24 19:00:00	-4.3	85	1	1	137	southeast wind	970	2.6	0	52

2021-12-24 20:00:00	-4.4	86	3.1	2	124	southeast wind	970	2.2	0	56
2021-12-24 21:00:00	-4.5	87	1.6	2	140	southeast wind	970	2.2	0	57
2021-12-24 22:00:00	-4.9	89	2.8	2	131	southeast wind	970	1.7	0	60
2021-12-24 23:00:00	-5.2	90	2.6	2	35	northeast wind	970	1.1	0	70
2021-12-25 00:00:00	-5.3	91	3.2	2	27	northeast wind	969	1.1	0	73
2021-12-25 01:00:00	-5.3	91	0.2	0	122	southeast wind	969	1.2	0	71
2021-12-25 02:00:00	-5.6	93	0.8	1	25	northeast wind	969	0.9	0	61
2021-12-25 03:00:00	-5.7	95	3	2	44	northeast wind	969	0.3	0	62
2021-12-25 04:00:00	-6.1	95	3.1	2	34	northeast wind	969	0.3	0	56
2021-12-25 05:00:00	-6.2	94	1.9	2	117	southeast wind	969	0.8	0	67
2021-12-25 06:00:00	-6.4	94	0.1	0	46	northeast wind	968	1.2	0	64
2021-12-25 07:00:00	-6.6	94	3.1	2	56	northeast wind	968	1.7	0	60
2021-12-25 08:00:00	-6.8	93	3.2	2	26	northeast wind	968	1.7	0.3	73
2021-12-25 09:00:00	-7.1	93	0	0	121	southeast wind	969	3	0	90
2021-12-25 10:00:00	-7.2	93	3.2	2	42	northeast wind	969	3	0	100
2021-12-25 11:00:00	-7.4	93	1	1	33	northeast wind	969	2.4	0	100
2021-12-25 12:00:00	-7.7	92	1.5	1	113	southeast wind	969	1.8	0.4	75
2021-12-25 13:00:00	-7.6	92	0.3	1	28	northeast wind	969	2.6	0.1	71
2021-12-25 14:00:00	-7.5	91	0.1	0	35	northeast wind	969	2.7	0	64
2021-12-25 15:00:00	-7.1	91	0.9	1	151	southeast wind	968	2.4	0.2	61
2021-12-25 16:00:00	-7	85	1.7	2	115	southeast wind	968	4.9	0	47
2021-12-25 17:00:00	-6.9	85	3.2	2	39	northeast wind	968	3.8	0	52
2021-12-25 18:00:00	-7.2	86	1.7	2	318	northwest wind	968	4.8	0	34
2021-12-25 19:00:00	-7.6	85	1.3	1	27	northeast wind	968	5.9	0	51
2021-12-25 20:00:00	-7.9	87	2.3	2	38	northeast wind	968	6.7	0	90
2021-12-25 21:00:00	-8.3	87	1.1	1	310	northwest wind	968	4.6	0	90
2021-12-25 22:00:00	-8.5	88	0.4	1	219	southwest wind	968	5.6	0	62
2021-12-25 23:00:00	-9.6	89	2.7	2	304	northwest wind	968	4	0	34
2021-12-26 00:00:00	-11.2	89	0.2	0	302	northwest wind	968	4	0	59
2021-12-26 01:00:00	-12.7	87	2.4	2	331	northwest wind	968	3.8	0	90
2021-12-26 02:00:00	-12.9	88	1	1	296	northwest wind	968	4.8	0	90
2021-12-26 03:00:00	-13.4	87	3.2	2	331	northwest wind	968	4.8	0	68
2021-12-26 04:00:00	-12.4	88	0.2	0	336	northwest wind	968	5	0	70
2021-12-26 05:00:00	-11.4	86	1.2	1	325	northwest wind	968	6.1	0	90
2021-12-26 06:00:00	-10.7	86	3	2	299	northwest wind	968	7.2	0	90
2021-12-26 07:00:00	-10.6	85	0.7	1	303	northwest wind	968	7.6	0	90
2021-12-26 08:00:00	-10.5	86	0.6	1	331	northwest wind	968	7.6	0	100
2021-12-26 09:00:00	-10.2	86	2.1	2	321	northwest wind	968	4.5	0	83

2021-12-26 10:00:00	-9.3	87	0.3	1	300	northwest wind	968	4.4	0	83
2021-12-26 11:00:00	-8.7	86	1.2	1	53	northeast wind	969	4.4	0	68
2021-12-26 12:00:00	-7.2	80	2.3	2	297	northwest wind	969	4.1	0	66
2021-12-26 13:00:00	-6.1	73	3.2	2	304	northwest wind	968	4.4	0	4
2021-12-26 14:00:00	-5	71	0.3	1	63	northeast wind	968	5.6	0	4
2021-12-26 15:00:00	-5.7	72	1.5	1	126	southeast wind	967	6.3	0	7
2021-12-26 16:00:00	-6	77	0.3	1	113	southeast wind	967	6.7	0	0
2021-12-26 17:00:00	-6.5	78	0.1	0	136	southeast wind	968	6.5	0	0
2021-12-26 18:00:00	-7.1	79	1.7	2	311	northwest wind	968	7.1	0	0
2021-12-26 19:00:00	-8.4	84	1.2	1	314	northwest wind	968	7	0	0
2021-12-26 20:00:00	-10	87	3.2	2	31	northeast wind	968	2.2	0	51
2021-12-26 21:00:00	-11.5	89	3.1	2	307	northwest wind	968	2.3	0	55
2021-12-26 22:00:00	-12.7	87	2.2	2	335	northwest wind	968	3.5	0	71
2021-12-26 23:00:00	-12.8	86	1	1	335	northwest wind	969	3.9	0	58
2021-12-27 00:00:00	-13.4	84	2.2	2	319	northwest wind	969	4.2	0	56
2021-12-27 01:00:00	-13.4	85	2.7	2	318	northwest wind	969	3.9	0	64
2021-12-27 02:00:00	-13.2	86	2.7	2	313	northwest wind	969	3.7	0	57
2021-12-27 03:00:00	-13.7	85	1.2	1	307	northwest wind	969	3.7	0	53
2021-12-27 04:00:00	-12.1	87	1.1	1	326	northwest wind	969	3.9	0	53
2021-12-27 05:00:00	-10.7	87	0	0	311	northwest wind	969	3.2	0	69
2021-12-27 06:00:00	-9.7	85	1.8	2	321	northwest wind	969	3.3	0	68
2021-12-27 07:00:00	-10.3	85	3.2	2	303	northwest wind	969	4.6	0	64
2021-12-27 08:00:00	-10.9	87	2.4	2	323	northwest wind	969	4.6	0	100
2021-12-27 09:00:00	-11.1	83	0.3	1	321	northwest wind	969	4.9	0	33
2021-12-27 10:00:00	-11.4	80	1.2	1	327	northwest wind	970	5.4	0	75
2021-12-27 11:00:00	-11	79	0.7	1	316	northwest wind	970	6.2	0	60
2021-12-27 12:00:00	-9.9	78	0.5	1	332	northwest wind	971	6.3	0	83
2021-12-27 13:00:00	-7.8	71	1	1	325	northwest wind	971	7.3	0	18
2021-12-27 14:00:00	-6.4	65	1.9	2	211	southwest wind	970	6.1	0	24
2021-12-27 15:00:00	-5	59	1.1	1	208	southwest wind	970	6.3	0	16
2021-12-27 16:00:00	-6.1	70	0.8	1	120	southeast wind	970	6.8	0	0
2021-12-27 17:00:00	-6.2	69	0.2	0	120	southeast wind	970	5	0	55
2021-12-27 18:00:00	-6.8	73	2.8	2	24	northeast wind	971	5.5	0	56
2021-12-27 19:00:00	-8	78	3	2	214	southwest wind	971	4.7	0	85
2021-12-27 20:00:00	-9.1	83	2	2	330	northwest wind	971	3.1	0	43
2021-12-27 21:00:00	-10.7	82	1.1	1	308	northwest wind	972	2.8	0	59
2021-12-27 22:00:00	-11.3	82	2.9	2	319	northwest wind	972	3.2	0	0
2021-12-27 23:00:00	-12.3	81	0.3	1	302	northwest wind	972	4.7	0	50

2021-12-28 00:00:00	-12.9	82	1.2	1	295	northwest wind	972	2.3	0	60
2021-12-28 01:00:00	-12.2	84	3.1	2	310	northwest wind	971	5.1	0	70
2021-12-28 02:00:00	-13.7	83	1.1	1	209	southwest wind	971	6.6	0	61
2021-12-28 03:00:00	-13.9	83	1	1	299	northwest wind	972	6.6	0	46
2021-12-28 04:00:00	-14.5	83	0.3	1	303	northwest wind	972	7.6	0	56
2021-12-28 05:00:00	-15.2	85	0.9	1	313	northwest wind	972	7.6	0	55
2021-12-28 06:00:00	-15.5	84	2.7	2	310	northwest wind	973	7.5	0	73
2021-12-28 07:00:00	-15.6	84	1.2	1	295	northwest wind	973	7.9	0	39
2021-12-28 08:00:00	-16.2	84	1.4	1	336	northwest wind	973	6.9	0	58
2021-12-28 09:00:00	-15.5	86	1.3	1	321	northwest wind	973	6.4	0	52
2021-12-28 10:00:00	-13.8	89	2.4	2	31	northeast wind	974	3.1	0	58
2021-12-28 11:00:00	-12.7	86	2.3	2	213	southwest wind	974	1.9	0	4
2021-12-28 12:00:00	-12	83	2.1	2	212	southwest wind	974	1.7	0	70
2021-12-28 13:00:00	-10.6	79	2.3	2	237	southwest wind	974	3.7	0	56
2021-12-28 14:00:00	-9.5	74	2.9	2	66	northeast wind	974	3.2	0	42
2021-12-28 15:00:00	-8.6	70	2	2	133	southeast wind	973	4.4	0	22
2021-12-28 16:00:00	-9.1	75	0.1	0	45	northeast wind	972	3.5	0	0
2021-12-28 17:00:00	-9.4	76	0.9	1	32	northeast wind	972	4	0	0
2021-12-28 18:00:00	-9.8	78	1.7	2	34	northeast wind	972	4.1	0	15
2021-12-28 19:00:00	-11.2	82	0.2	0	337	northwest wind	973	4.1	0	45
2021-12-28 20:00:00	-12.3	84	1.8	2	335	northwest wind	973	3.5	0	68
2021-12-28 21:00:00	-13.2	85	2.2	2	320	northwest wind	973	3.5	0	69
2021-12-28 22:00:00	-14.7	83	1.7	2	325	northwest wind	973	3.2	0	66
2021-12-28 23:00:00	-15.2	84	0.5	1	294	northwest wind	973	3.4	0	52
2021-12-29 00:00:00	-14.5	85	2.6	2	331	northwest wind	973	3.4	0	61
2021-12-29 01:00:00	-14.5	83	1.7	2	352	north wind	973	1.8	0	60
2021-12-29 02:00:00	-15	84	0.4	1	346	north wind	972	2.4	0	53
2021-12-29 03:00:00	-15.3	85	0.2	0	278	west wind	972	3.1	0	60
2021-12-29 04:00:00	-16.2	85	2.2	2	46	northeast wind	972	1.3	0	57
2021-12-29 05:00:00	-16.6	83	1.2	1	333	northwest wind	972	3.8	0	58
2021-12-29 06:00:00	-16.4	84	1.5	1	315	northwest wind	972	4	0	66
2021-12-29 07:00:00	-16.1	82	1.1	1	294	northwest wind	971	3.9	0	70
2021-12-29 08:00:00	-15.9	82	0.9	1	326	northwest wind	971	3.9	0	58
2021-12-29 09:00:00	-16.2	81	0.6	1	318	northwest wind	971	4.2	0	46
2021-12-29 10:00:00	-16.1	83	1.8	2	300	northwest wind	972	5	0	36
2021-12-29 11:00:00	-13.7	83	1.9	2	301	northwest wind	972	4.6	0	47
2021-12-29 12:00:00	-11.9	83	2.1	2	38	northeast wind	972	3.1	0	60
2021-12-29 13:00:00	-10.7	81	0.6	1	25	northeast wind	972	4.4	0	0

2021-12-29 14:00:00	-10.2	77	1	1	155	southeast wind	971	3.7	0	68
2021-12-29 15:00:00	-9.3	72	0.1	0	125	southeast wind	971	3.8	0	31
2021-12-29 16:00:00	-9.2	72	0.1	0	124	southeast wind	970	4.4	0	7
2021-12-29 17:00:00	-9.3	72	2.1	2	136	southeast wind	970	4.5	0	2
2021-12-29 18:00:00	-10.2	75	2.1	2	140	southeast wind	970	4.4	0	35
2021-12-29 19:00:00	-11.2	81	2.1	2	214	southwest wind	970	4.2	0	7
2021-12-29 20:00:00	-12	82	2.5	2	262	west wind	971	3.8	0	58
2021-12-29 21:00:00	-12.8	85	0.2	0	295	northwest wind	971	3.5	0	67
2021-12-29 22:00:00	-14.3	85	0.1	0	311	northwest wind	970	2.9	0	62
2021-12-29 23:00:00	-15.8	86	0.7	1	327	northwest wind	970	2.6	0	69
2021-12-30 00:00:00	-16.4	83	3	2	205	southwest wind	970	2.6	0	55
2021-12-30 01:00:00	-16.8	83	1.8	2	314	northwest wind	969	4.4	0	67
2021-12-30 02:00:00	-16.5	88	2.4	2	303	northwest wind	969	5	0	57
2021-12-30 03:00:00	-17.2	84	1.9	2	211	southwest wind	968	1.1	0	90
2021-12-30 04:00:00	-16.6	85	1.3	1	309	northwest wind	968	2.2	0	83
2021-12-30 05:00:00	-16	85	3	2	297	northwest wind	968	1.7	0	56
2021-12-30 06:00:00	-17.1	83	0.4	1	242	southwest wind	968	3	0	62
2021-12-30 07:00:00	-17.6	83	0	0	298	northwest wind	968	3.4	0	48
2021-12-30 08:00:00	-17.6	83	1	1	309	northwest wind	968	3.5	0	52
2021-12-30 09:00:00	-17.3	83	3.2	2	337	northwest wind	968	4.2	0	100
2021-12-30 10:00:00	-17.2	82	1.8	2	218	southwest wind	968	3.8	0	100
2021-12-30 11:00:00	-15.7	84	2.2	2	326	northwest wind	968	3.5	0	95
2021-12-30 12:00:00	-14.3	84	2.4	2	306	northwest wind	968	3.9	0	90
2021-12-30 13:00:00	-11.8	82	2	2	121	southeast wind	968	3.7	0	67
2021-12-30 14:00:00	-9.8	71	1.1	1	222	southwest wind	967	4.5	0	65
2021-12-30 15:00:00	-8.7	68	1.8	2	149	southeast wind	967	5.9	0	68
2021-12-30 16:00:00	-7.9	67	0.1	0	47	northeast wind	966	4.6	0	70
2021-12-30 17:00:00	-7.8	66	1.5	1	126	southeast wind	966	4.3	0	25
2021-12-30 18:00:00	-9.2	71	0.9	1	123	southeast wind	967	4.3	0	5
2021-12-30 19:00:00	-10.7	77	1.1	1	312	northwest wind	967	4.1	0	71
2021-12-30 20:00:00	-11.8	82	1.4	1	334	northwest wind	967	3.3	0	90
2021-12-30 21:00:00	-13.1	84	2.8	2	33	northeast wind	968	2.6	0	95
2021-12-30 22:00:00	-14	84	1.5	1	299	northwest wind	968	2.6	0	100
2021-12-30 23:00:00	-14.3	84	1.8	2	304	northwest wind	968	3.1	0	95
2021-12-31 00:00:00	-14.9	83	0.2	0	313	northwest wind	968	3.1	0	84
2021-12-31 01:00:00	-15.4	84	2.3	2	329	northwest wind	968	3.4	0	70
2021-12-31 02:00:00	-16.5	82	0.2	0	300	northwest wind	968	4.1	0	66
2021-12-31 03:00:00	-17.1	83	2.9	2	278	west wind	968	3.8	0	64

2021-12-31 04:00:00	-17.5	82	0.5	1	303	northwest wind	969	4.6	0	60
2021-12-31 05:00:00	-16.8	84	1.4	1	32	northeast wind	970	4.7	0	57
2021-12-31 06:00:00	-15.9	84	0.5	1	31	northeast wind	970	2.1	0	68
2021-12-31 07:00:00	-16	83	1.1	1	233	southwest wind	971	1.7	0	66
2021-12-31 08:00:00	-15.8	84	0.2	0	314	northwest wind	971	2.4	0	78
2021-12-31 09:00:00	-16.1	84	2.9	2	316	northwest wind	972	2.5	0	63
2021-12-31 10:00:00	-15.8	83	2.5	2	331	northwest wind	972	2.8	0	74
2021-12-31 11:00:00	-15.6	82	0.3	1	307	northwest wind	972	2.4	0	58
2021-12-31 12:00:00	-14.1	81	2.9	2	330	northwest wind	972	2.4	0	4
2021-12-31 13:00:00	-12.3	77	1.1	1	322	northwest wind	972	3.2	0	0
2021-12-31 14:00:00	-10.8	72	2	2	333	northwest wind	971	3.6	0	0
2021-12-31 15:00:00	-9.3	68	1.2	1	227	southwest wind	971	3.6	0	0
2021-12-31 16:00:00	-8	64	0	0	296	northwest wind	970	4.4	0	1
2021-12-31 17:00:00	-7.3	61	1.5	1	131	southeast wind	970	5.3	0	47
2021-12-31 18:00:00	-10.2	74	0.9	1	133	southeast wind	970	4.9	0	14
2021-12-31 19:00:00	-11.7	82	3.1	2	67	northeast wind	970	3.1	0	90
2021-12-31 20:00:00	-13.2	85	0.1	0	24	northeast wind	970	3.1	0	90
2021-12-31 21:00:00	-13.8	87	0.9	1	335	northwest wind	970	3.1	0	75
2021-12-31 22:00:00	-14.3	85	3.2	2	331	northwest wind	969	1.9	0	100
2021-12-31 23:00:00	-14.6	85	2.3	2	297	northwest wind	969	1.9	0	100
